# Selective *endo*-Cyclic α‑Functionalization of Saturated *N*‑Alkyl Piperidines

**DOI:** 10.1021/acs.joc.5c01742

**Published:** 2025-08-18

**Authors:** Rachel C. Phillips, John C. K. Chu, Alex A. Rafaniello, Matthew J. Gaunt

**Affiliations:** Yusuf Hamied Department of Chemistry, Lensfield Road, Cambridge CB2 1EW, United Kingdom

## Abstract

Saturated *N*-alkyl heterocycles are among the most significant structural motifs in natural products, small-molecule biological probes, and pharmaceutical agents, as evidenced by their prevalence in FDA-approved drugs. Substituted derivatives of these cyclic tertiary alkylamine scaffolds often exhibit markedly different physicochemical and biological properties compared to their unsubstituted counterparts. Consequently, methods for the selective functionalization of these scaffolds would greatly facilitate the optimization of biological activity, physicochemical properties, and systematic evaluations of structure–activity relationships. In this work, we present a robust platform for the late-stage α-functionalization of *N*-alkyl piperidines through a sequential process involving iminium ion formation followed by nucleophilic functionalization. Key to this strategy is the selective formation of *endo*-iminium ions from six-membered N-heterocycles, achieved via α-C–H elimination of cyclic tertiary alkylamine *N*-oxides. This approach provides exceptional *endo*-selectivity, enabling efficient further functionalization. The method allows for the *in situ* addition of diverse carbon-based nucleophiles to the iminium intermediates, demonstrated across a range of piperidine-based systems; alkylation, azinylation, and trifluoromethylation are successfully demonstrated through a variety of activation modes. Furthermore, the formal C–H functionalization sequence has been successfully applied to the late-stage modification of complex bioactive molecules, underscoring the potential of this methodology to expand drug-like chemical space.

## Graphical abstract

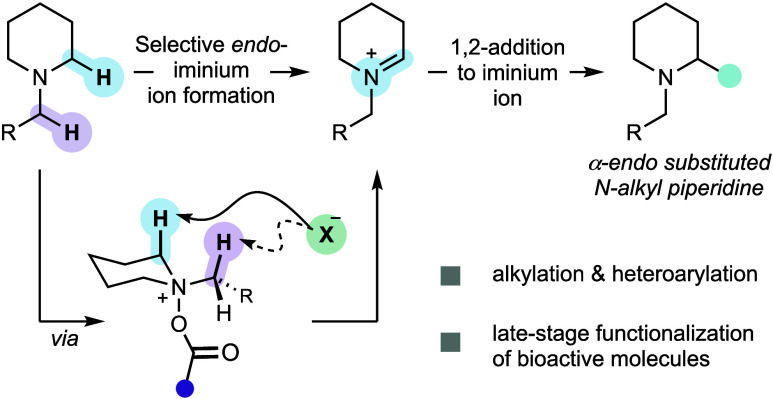

## Introduction

Cyclic tertiary alkylamines are important structural features in many classes of bioactive molecules, especially pharmaceutical agents, with those containing a piperidine motif being most ubiquitous (Figure 1A).^1^ The physicochemical and biological properties of molecules containing *N*-alkyl piperidines, as well as other saturated azacycles, are often influenced by the level of substitution around the sp^3^-hybridized nitrogen atom (Figure 1B). Substituents on the cyclic scaffold can impact the conformational rigidity of the molecule and thus modulate its binding interactions with a target protein, as exemplified by the development of LNP023 (Figure 1C). Additionally, substituents proximal to the heterocyclic nitrogen atom can regulate the molecule’s ionization state under physiological conditions (amine basicity) and influence factors such as lipophilicity, solubility, metabolism, and interference with the hERG ion channel and targeted receptors, among others (Figure 1D).^2^

**Figure 1:**
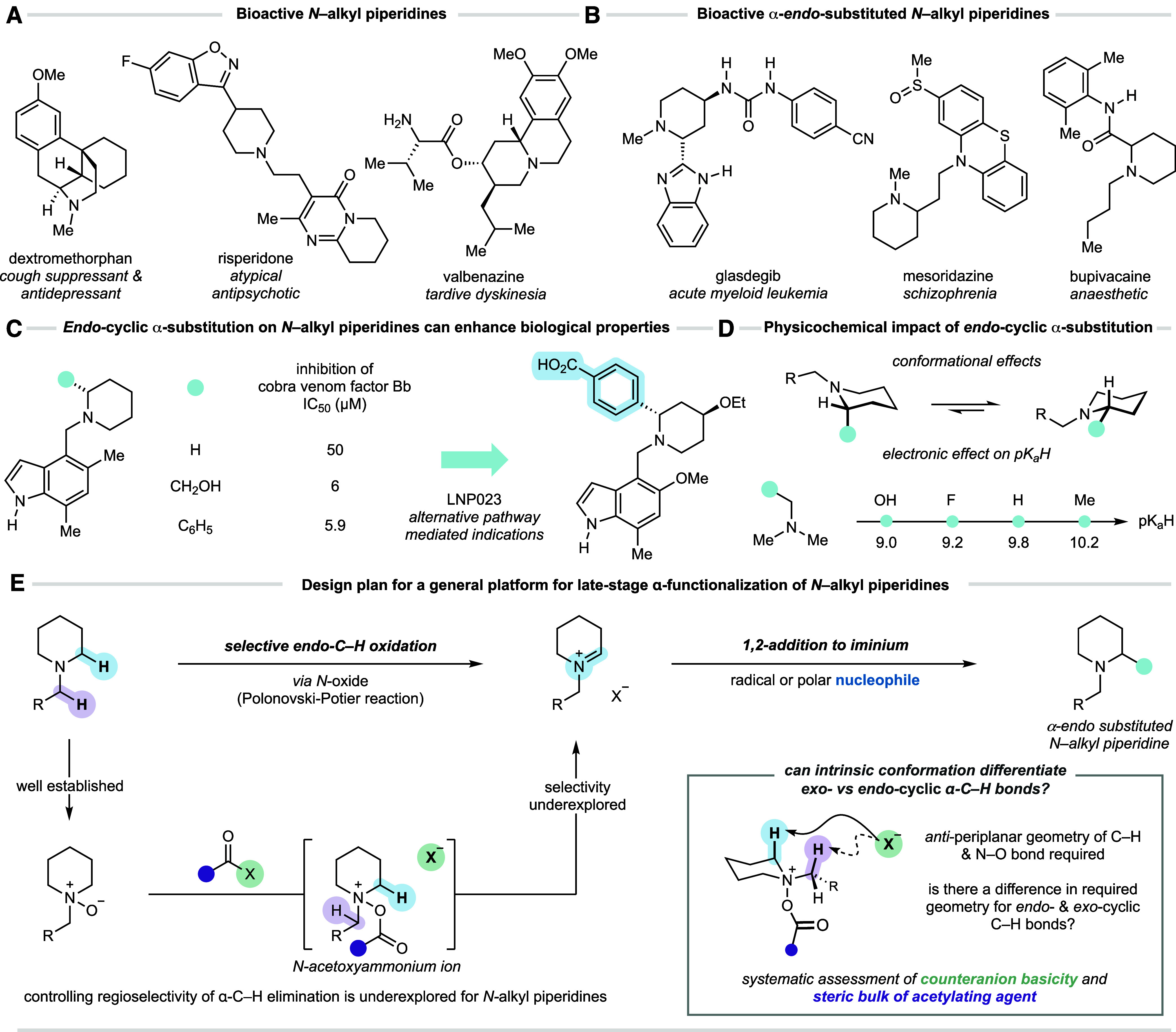
Evolution of a strategy for *endo*-cyclic α-selective C–H functionalization of *N*-alkyl piperidines.

A common design strategy for fine-tuning the properties of cyclic tertiary alkylamine containing drugs has, therefore, been the incorporation of small structural changes at the *endo*-cyclic α-position to the nitrogen atom. To access these analogues, the synthetic practitioner generally leverages the available feedstock pool of α-substituted free­(NH) amines in a *de novo* synthesis of the target cyclic tertiary alkylamines. When the feedstock pool is insufficient, a wide variety of stepwise synthetic strategies exist for the synthesis of α-substituted cyclic tertiary alkylamines; however, these approaches can be laborious or dependent on specific functional group patterns.^3^ Alternatively, several methods for the direct α-functionalization of cyclic secondary amine derivatives have been developed.^4,5^ The most commonly practiced methods involve directed lithiation of *N*-Boc piperidines;^5a,5b^ directed metal-catalyzed C–H activation of auxiliary-derivatized piperidines;^5c,5d^ metallocarbenoid insertion into *N*-Boc piperidines;^5e^ electrochemical oxidation of piperidine carbamates or sulfinamides,^5f,5g^ and intermolecular hydride-transfer approaches to form imines that can be intercepted with organometallic reagents.^5h^ Several photochemical methods have also been developed, including decarboxylative cross-couplings of *N*-Boc pipecolic acid derivatives,^5i,5j^ and 1,5-hydrogen migration of *N*-benzoyl pyrrolidines;^5k^ methods to form and functionalize α-amino radicals via hydrogen atom abstraction^5l^ or single electron oxidation methods;^5m^ and photocatalytic-mediated addition of alkyl radicals to cyclic iminium surrogates.^5n^

While these methods offer a means to elaborate saturated secondary azacycles and their derivatives, most of these transformations cannot be applied to the functionalization of cyclic tertiary alkylamines, particularly *N*-alkyl piperidines, due to the lack of reactivity and selectivity for reaction on the ring (*endo*) or on the substituent (*exo*) position. For example, there is a single report of metal-catalyzed carbenoid insertion into C–H bonds in *N*-alkyl piperidines. Beckwith and co-workers reported that donor–acceptor metallocarbenoids can be used for C–H functionalization at the α-position of *N*-alkyl piperidines in several complex molecules, although functionalization occurred selectively at the *exo*-cyclic position in *N*-methylpiperidine units.^6,6a^

Some success has been reported using metal-catalyzed polar oxidation pathways to form iminium ions *in situ* followed by interception with nucleophiles. These cross dehydrogenative coupling methods have facilitated alkynylation, nitroalkylation, Mannich reactions, Friedel–Crafts processes, and phosphonations, among others.^6b^ While most reports describe the functionalization of *N*-aryl tetrahydroisoquinolines in the *endo*-cyclic α-benzylic position, there are selected examples of α-functionalization on *N*-substituted pyrrolidines and piperidines.^6c–6f^ Linked to cross dehydrogenative couplings are methods based on a hydride shift from the α-position of cyclic tertiary alkylamines to a pendant electrophile, forming a cyclic iminium ion intermediate.^6g^ This activation mode enables the formal addition of hard nucleophiles adjacent to the nitrogen atom of prefunctionalized azacycles.

Hydrogen atom transfer (HAT)-based approaches to form an α-amino radical have also been reported, though examples are limited to very simple cases.^6h,6i^ Several photocatalytic platforms, also involving HAT, which form an α-amino radical on the saturated azacycle that can couple with radical acceptors have been reported but these works do not describe any examples of selective transformations on structurally or functionally unbiased cyclic tertiary alkylamines.^6j,6k^ A notable exception is the work by Rovis and co-workers, which showed that a reversible HAT catalysis manifold was able to deliver the selective *endo*-cyclic α-functionalization of various cyclic tertiary alkylamines.^6l^ The intrinsic selectivity challenges of all these methods are compounded when applied to the late-stage functionalization of *N*-alkyl piperidine motifs in complex molecules due to the presence of several competing functionalities, which complicate the control of regioselectivity. Accordingly, the means to directly install a new α-substituent onto the saturated ring of an *N-*alkyl piperidine at either an early, mid, or late stage of a synthesis campaign remains a challenge but would be an invaluable tool for streamlining access to finely tuned analogs for biological evaluation and other target synthesis applications.

## Reaction Design

We questioned whether a strategy of functionalizing the α-C–H bond on the heterocyclic unit of *N*-alkyl piperidines might be feasible by combining an approach for the selective formation of an iminium ion and its subsequent interception via 1,2-addition with an alkyl nucleophile (Figure 1E). Ideally, such a strategy would provide access to a range of subtly differentiated analogues by coupling the same iminium ion intermediate with a variety of carbon-based nucleophiles that may be of interest to molecular discovery programs. We recognized that oxidation of *N*-alkyl piperidines to iminium ions is well established using many different types of reagents but were conscious that many of these methods offer no obvious means to control the *endo*- vs *exo*-selectivity of iminium ion formation in structurally unbiased substrates.^7^ Even a simple *N*-alkyl piperidine displays at least two subtly different α-C–H bonds. Accordingly, a process by which a level of reagent control could be applied to the positional outcome of iminium ion formation could provide a means to engineer the desired *endo*-selective reaction on structurally and functionally unbiased *N*-alkyl piperidines.

We were drawn to the seminal work of Polonovski and Potier, who showed that iminium ions can be generated via the rearrangement of tertiary alkylamine *N*-oxides in the presence of an acylating agent, typically trifluoroacetic anhydride (TFAA).^8,8a,8b^ While this mild transformation has been frequently employed during the synthesis of complex alkaloids, the regiochemical outcome is highly dependent on the reaction conditions and substrate structure.^8c,8d^ Additionally, these examples often exhibit fused ring systems, thereby eliminating the distinction between *endo*- and *exo*-selectivity.^8d–8f^ The proposed E2 mechanism dictates an *anti*-periplanar relationship with respect to the *N*-acetoxy group and the α-C–H bond being broken, and this preference is typically observed in simple aliphatic systems, although there are exceptions.^8d^ In addition to this, the regioselectivity is typically influenced by the acetylating agent used. Acetic anhydride produces a weak nucleofuge, inhibiting N–O bond cleavage, and a strong base, facilitating C–H bond breaking. As a result, a more E1cB-transition state occurs, favoring elimination of the more kinetically acidic proton. On the other hand, TFAA produces a strong nucleofuge and a weak base, promoting a more E1-like transition state that favors formation of the more thermodynamically stable iminium ion. The complex interplay between kinetic acidity and iminium ion stability makes regioselectivity predictions challenging in more complex systems.^8g^ Moreover, reactions at elevated temperature can have a profound impact on iminium ion distribution, which has been rationalized in terms of enhanced conformational dynamics.^8h^ To the best of our knowledge, the regio-controlled formation of *endo*-cyclic iminium ions from their corresponding tertiary alkylamine *N*-oxides has not been systematically investigated in structurally unbiased substrates, though there are a few isolated examples that demonstrate *endo*- or *exo*-selectivity.^8i–8k^ Furthermore, there are only limited examples of *endo*-selective iminium ion generation in complex systems via the Polonovski–Potier reaction.^8l,8m^ Therefore, we questioned whether the outcome of the E2 elimination could be engineered by the action of the counteranion of the acylating agent, which formally deprotonates the *anti*-periplanar C–H bond, and the steric properties of the acylating reagent itself. Together, these features might enable exploitation of subtle differences between the transition state energies of the elimination pathways with the reactive α-C–H bonds both inside and outside the cyclic architecture of the tertiary alkylamine *N*-oxide. Upon the selective formation of the *endo*-cyclic iminium ion, we proposed that the adaptation of our mild carbonyl alkylative amination manifolds would enable the 1,2-addition of a range of carbon-based nucleophiles^9^ and result in the formation of the α-substituted *N*-alkyl piperidine products in both simple and complex molecules. Here, we report the successful realization of this idea through the development of a practical synthetic workflow that enables the transformation of *N*-alkyl piperidines into substituted variants wherein a carbon group has been added, selectively, at the α-position of the saturated azacycle. The reaction works well across a range of *N*-alkyl piperidines in both simple and complex systems, supporting valuable applications ranging from building block elaboration to late-stage functionalization.

## Results and Discussion

At the outset, the factors controlling the regiochemical outcome of the Polonovski–Potier elimination were evaluated. *N*-Benzyl piperidine **1a** was used as the representative substrate to challenge the selectivity aspects associated with abstracting the *endo* α-proton in the presence of an activated benzylic *exo* α-proton. The corresponding *N*-oxide **2a** was prepared in quantitative yield by oxidation of the amine with *m*CPBA, although it should be noted that there are several methods available for this type of transformation.^10^ Accordingly, the impact of a range of acylating reagents on the elimination step was assessed based on the *endo*:*exo*-selectivity of the resulting iminium ion products (**3a**/**4a**), which were measured using direct ^1^H NMR spectroscopic analysis of the reaction mixture (Figure 2A). These preliminary studies used 2.5 equiv of acetylating agent at 0 °C (condition A). The first round of experiments varied the acylating reagent and reflected a range of p*K*_a_H values of the counteranion released, which mediates the subsequent E2 elimination step.^8d,11,12^ As previously noted, weaker bases lead to a more E1-like transition state that favors formation of the thermodynamically favorable iminium ion. As a comparison to an all-carbon-based system, 1-alkylcyclohexenes (*endo*-isomer) are reported to be thermodynamically more stable than the corresponding alkyldienecyclohexanes (*exo*-isomer). The differences in stability between the *exo*- and *endo*-isomers have been attributed to fewer ring interactions.^13^ We questioned whether these findings could be extrapolated to six-membered N-heterocycles. Gratifyingly, a strong correlation was observed between the desired *endo*-selectivity and the p*K*_a_H of the anion released upon acylation. Acylating reagents that released less basic counter-anions, such as chloride, were found to be highly selective, though yields were modest. Anhydrides with more basic carboxylate leaving groups (from the anhydrides) gave both poor *endo*-selectivity and low assay yields for iminium ion formation. Formation of the hydroxylammonium salt **5** was observed as a major side product, diminishing the yields of the desired iminium ions **3a**/**4a**. This finding was in line with observations made by Volz and Gartner on the acetolysis of *N*-acetoxyammonium salts.^14^ The formation of **5** was minimized (<10%) by adopting an alternative procedure whereby 6.6 equiv of acetylating agent was used at −78 °C (condition B). Again, a clear trend was observed between *endo*-selectivity and the p*K*_a_H of the anion released, with acetyl chloride and bromide delivering the desired *endo*-iminium ion **3a** in excellent assay yields. Interestingly, the commonly used trifluoroacetic anhydride gave a good yield for combined iminium ion formation but reduced selectivity for the *endo*-cyclic isomer under both sets of reaction conditions.

**Figure 2:**
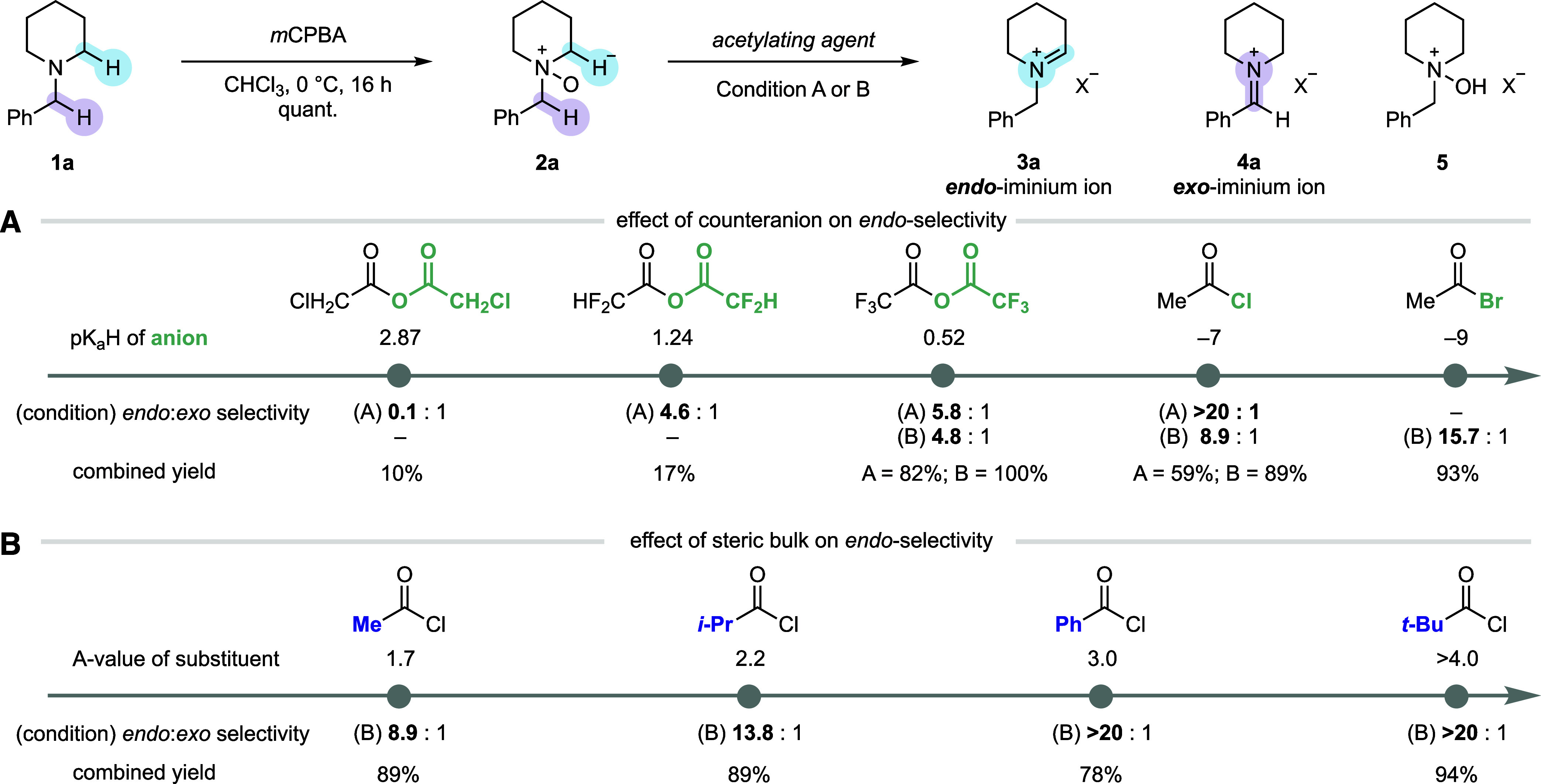
Overview of the optimization for selective *endo*-cyclic iminium ion formation. Condition A: 2.5 equiv acetylating agent, CD_2_Cl_2_ [0.3 M], 0 °C, 3 h. Condition B: 6.6 equiv acetylating agent, CD_2_Cl_2_ [0.3 M], −78 °C to rt, 5 h. (A) Effect of counteranion released from the acylating agent. (B) Effect of steric factors in the acylating reagent.

Next, we focused on varying the nature of the acyl group while maintaining the chloride leaving group. Despite acetyl bromide providing better *endo*-selectivity and assay yield, the acid chloride series was advanced due to their wider availability and hence more practical application (Figure 2B). A clear trend was observed that correlates the steric volume of the acyl group (categorized here by *A*-value) to the selectivity and assay yield of the *endo*-cyclic iminium ion **3a**, such that the use of pivaloyl chloride (PivCl) led to clean formation of the desired intermediate with a >20:1 regioisomeric ratio. Although classical Polonovski–Potier reaction conditions (TFAA at 0 °C)^8b^ gave reasonably good selectivity (5.8:1), the use of PivCl (>20:1) led to a notable advancement, which can be essential during downstream applications such as the late-stage modification of complex bioactive molecules. Notably, the formation of **5** was further minimized (<5%) when using more sterically hindered acylating reagents, such as pivaloyl and benzoyl chlorides. While the basicity of the counteranion is known to influence the pathway of α-C–H elimination in the Polonovski–Potier reaction, this study highlights that the steric bulk on the acylating agent is also important to the regiochemical outcome.^8d^

With the practical reaction conditions in hand, a preliminary assessment of the scope of this modified Polonovski–Potier elimination was explored (Figure 3). Formation of the tertiary amine *N*-oxides (**2**) from the corresponding amines typically occurred in excellent yield (see the Supporting Information). Varying the *N*-alkyl substituent had no impact on the *endo:exo* regioselectivity of the elimination step, with *N*-benzyl (**3a**), linear *N*-alkyl (**3b**), *N*-methyl (**3c**), α-branched *N*-alkyl (**3d**), and *N*-aryl piperidines (**3e**) all undergoing clean conversion to the corresponding *endo*-cyclic iminium ions with good to excellent assay yields. Substitution adjacent to the nitrogen atom of the piperidine ring also led to >20:1 *endo:exo* selectivity, with an approximate 6:1 preference for *endo*-iminium ion formation at the position bearing the methyl group (**3f**/**3f′**). However, while the *endo*-selectivity was still >20:1 for piperidine with a substituent in the 3-position position, there was little preference for the formation of the *endo*-iminium ion at the 2- or 6-positions of the ring (**3g**, **3g′**, respectively). Cyclic tertiary alkylamines other than piperidine were found to be less viable substrates. *N*-Benzyl morpholine (to **3i**), as well as piperazine (to **3j**), failed to deliver any of the desired iminium ion intermediates, possibly due to a documented decomposition pathway of these species.^8d,8h^ Changing the *N*-substituent had little impact on the outcome of iminium ion formation. *N*-Benzyl pyrrolidine and azepane exhibited little *endo:exo* selectivity during iminium ion formation (to **3k**/**4k** and **3l**/**4l**, respectively), although the reactive intermediates were formed in high assay yield. However, we were pleased to observe that *N*-benzyl tetrahydroisoquinoline was smoothly converted to its *endo*-cyclic iminium ion (**3m**) with exquisite selectivity even though this substrate presents a choice of two subtly different benzylic C–H bonds during the elimination process. It is important to note that our later studies (see Table 1) in connection with the subsequent iminium addition step revealed that 2.5 equiv of PivCl could be used instead of the 6.6 equiv used in Figure 3. To calibrate this observation, we subsequently demonstrated that the selectivity in the formation of iminium ion **3a** was not affected when using 2.5 or 6.6 equiv of PivCl. Accordingly, we have not retrospectively investigated the experiments in Figure 3 with 2.5 equiv of PivCl.

**Figure 3:**
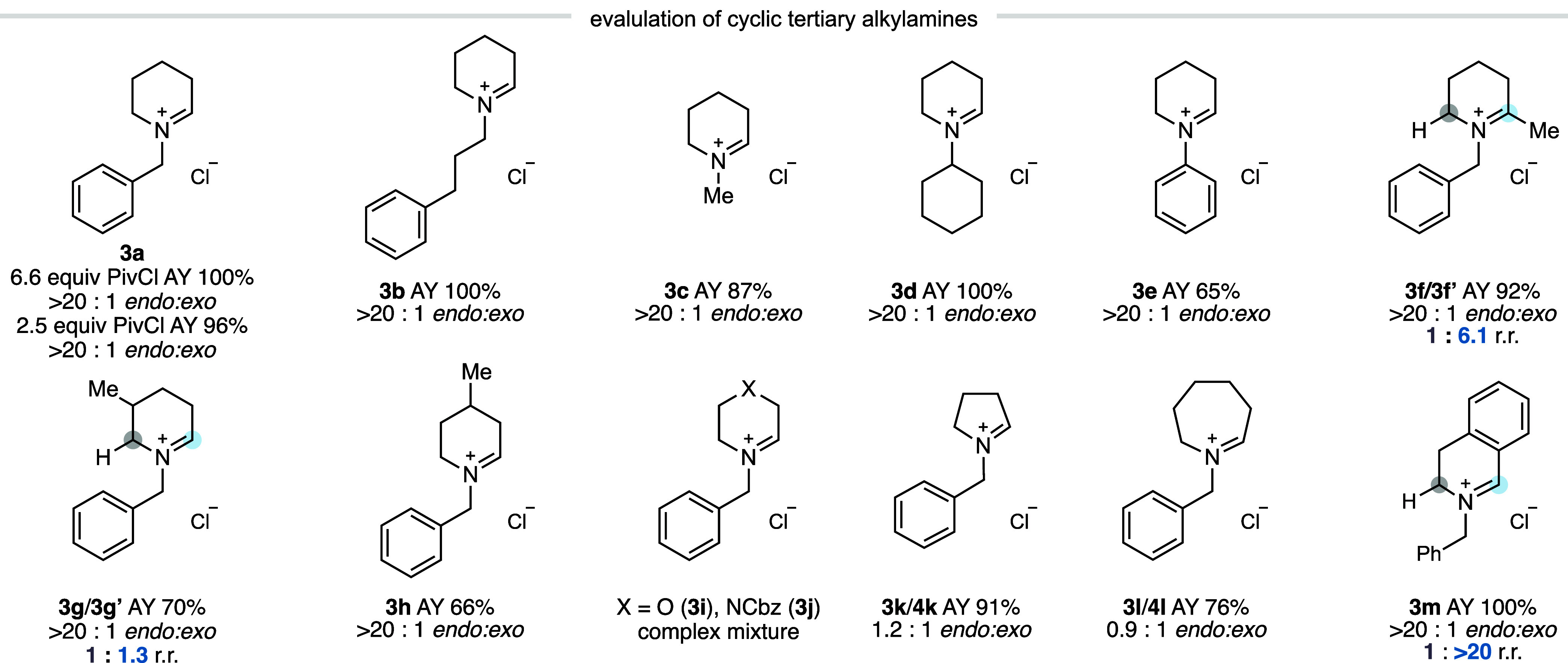
Preliminary evaluation of iminium selectivity across a range of cyclic tertiary alkylamines. Reactions were performed on a 0.2 mmol scale using 6.6 equiv of PivCl unless otherwise specified. AY = assay yield determined by ^1^H NMR using 1,1,2,2-tetrachloroethane as an internal standard.

**Table 1:**
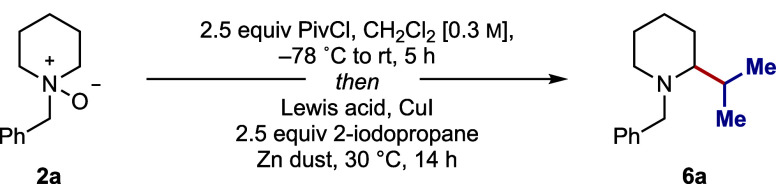
Selected Optimization for the One-Pot **α**-Alkylation of **2a**

entry	Zn equiv	CuI mol %	R_3_SiOTf, equiv	**6a** %^a^
1^d^	2.0	0	TMSOTf, 1.5	3
2^b^	2.0	0	TMSOTf, 1.5	48
3^c^	1.5	25	TMSOTf, 1.5	66
4^c^	1.5	25	TMSOTf, 0.75	70
5^c^	1.5	25	TBSOTf, 0.75	100

a Yield of 6a was determined by ^1^H NMR using 1,1,2,2-tetrachloroethane as an internal standard.

b Order of addition: TMSOTf, alkyl iodide, Zn.

c Order of addition: TMSOTf, CuI, alkyl iodide, Zn.

d Order of addition: Zn, alkyl iodide, TMSOTf.

To better understand the selectivity aspects of this reaction, computational modeling studies were performed to provide a theoretical basis for the experimental results (Figure 4). Using *N*-benzyl piperidine *N*-oxide (**2a**) and PivCl as the model substrates, density functional theory (DFT) calculations were performed at the wB97XD/6-311++g­(d,p) level of theory (see the Supporting Information).^15^ Analysis of the transition states revealed that the lowest energy pathway was, indeed, consistent with elimination from the *endo*-cyclic α-C–H bond (*endo* **-TS**).

**Figure 4:**
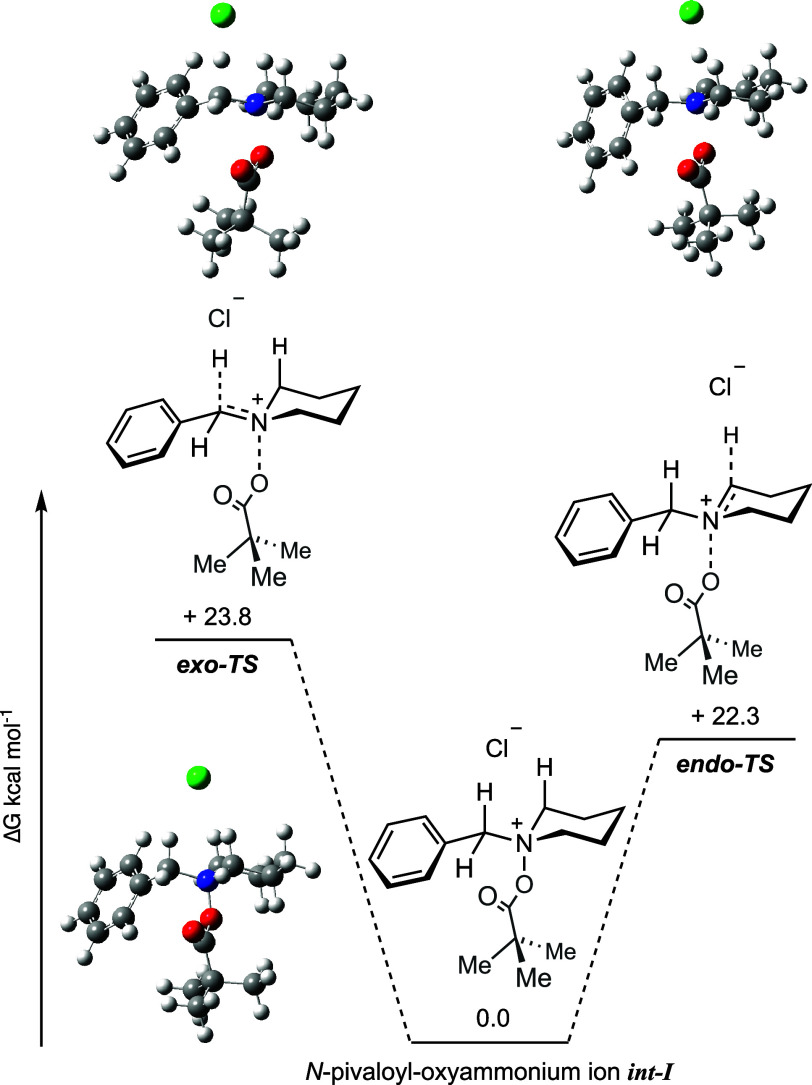
Single-point energies of the *exo*- and *endo*-cyclic α-C–H elimination transition states. Calculations were performed at the wB97XD/6-311++g­(d,p) level of theory.

Furthermore, the difference in the relative energies between the *endo* **-TS** and *exo* **-TS** (1.56 kcal mol^–1^) gave rise to a predicted regioisomeric ratio of >20:1 at 253 K. This value was determined to be the approximate temperature at which iminium ion **3a** was formed, based on ^1^H NMR spectroscopic experiments recorded at 5 °C intervals between −50 and 25 °C. Presumably, the difference in relative energies can be attributed to the *anti*-periplanar alignment of the *endo*- and *exo*-cyclic α-C–H protons, which exhibited dihedral angles with respect to the N–O bond of 175° and 173°, respectively. Interestingly, the *endo*-cyclic transition state in the five-membered ring pyrrolidine system becomes less favorable, resulting in an approximately 1:1 predicted regioselectivity (**3k**/**4k**) in line with our experimental observations (see the Supporting Information).

Next, the 1,2-addition of carbon-based nucleophiles to the *in situ*-generated iminium ion was investigated, completing the overall functionalization process. Our laboratory has developed several broad platforms for the synthesis of α-branched secondary and tertiary alkylamines based on the addition of alkyl radicals,^9a–9c^ acyl radicals,^9d^ alkylzinc reagents,^9e^ and heteroarylindium species^9f^ to *in situ*-generated iminium ions. The zinc-mediated carbonyl alkylative amination (CAA) manifold^9e^ was selected to assess the 1,2-addition process on the representative *N*-alkyl piperidine **1a**, via its *N*-oxide **2a**. Optimization of this transformation began by telescoping the iminium ion formation step (to **3a**) with the CAA step to formulate a one-pot process for the α-alkylation of *N*-alkyl piperidines. Starting with the standard conditions for the Zn-mediated CAA reaction,^9e^ zinc dust (2 equiv), 2-iodopropane (2.5 equiv), and TMSOTf (1.5 equiv) were added sequentially to the *in situ* generated *endo*-cyclic iminium ion **3a**. Under these conditions, the desired α-alkylated product **6a** could not be detected. Control reactions revealed that the excess of PivCl was detrimental to the Zn-mediated CAA reaction (see the Supporting Information). Upon further investigation, it was found that the amount of PivCl could be reduced from 6.6 to 2.5 equiv, while still maintaining complete selectivity and excellent yield of *endo*-cyclic iminium ion **3a** (*vide supra*). Consequently, 2.5 equiv of PivCl was used henceforth when coupling **3a** with any carbon-based nucleophile. Using these modified conditions for iminium ion formation, the Zn-mediated CAA reaction gave the desired α-alkylated product **6a**, albeit in 3% yield (Table 1, entry 1). Further exploration of reaction conditions revealed that the order of addition of the alkylating reagents was of paramount importance for maximizing the yield of **6a** (entries 1 vs 2). In line with previous findings,^9e^ the inclusion of substoichiometric CuI was necessary for alkyl addition to the iminium ion. Decreasing the amount of zinc and TMSOTf further enhanced the yield of **6a** (entries 3 and 4). Finally, the use of TBSOTf as the Lewis acid proved optimal, providing **6a** in 100% assay yield (entry 5).

Optimal conditions for the one-pot α-alkylation with secondary alkyl iodides involved dropwise addition of **2a** to a solution of PivCl (2.5 equiv) in dichloromethane cooled to −78 °C. Upon warming slowly to room temperature over 5 h, the desired *endo*-cyclic iminium ion **3a** was formed selectively in excellent assay yield (as determined by ^1^H NMR). Then, **3a** was treated, sequentially, with TBSOTf (0.75 equiv), CuI (25 mol %), 2-iodopropane (2.5 equiv), and zinc dust (1.5 equiv) and the reaction stirred for 14 h at 30 °C. Interestingly, these conditions were not applicable to the addition of primary or tertiary alkyl iodides (producing low yields of **6b** and **6c**) and so successive rounds of optimization were required for each class of alkyl iodide. This revealed that different stoichiometries of reagents were required (Scheme 1 and see the Supporting Information).

**Scheme 1:**
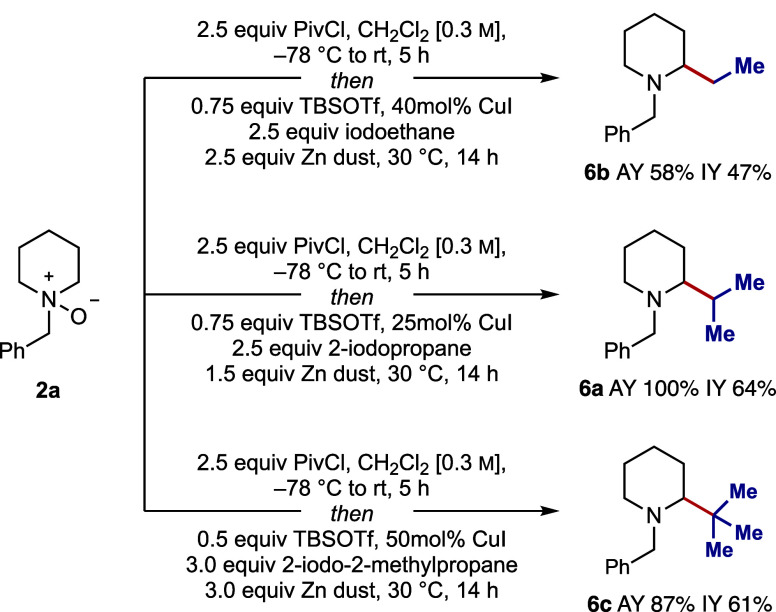
Optimal Conditions for Different Classes of Alkyl Halides^a^

a AY = assay yield determined by ^1^H NMR using 1,1,2,2-tetrachloroethane as an internal standard. IY = isolated yield.

Using *N*-benzyl piperidine *N-*oxide (**2a**), a range of primary, secondary, and tertiary alkyl iodides containing several types of functional groups were shown to efficiently add to the *endo*-cyclic iminium ion (**3a**) and gave α-alkylated products **6a**–**p** in, generally, good yields for the multistep process (Figure 5A). All yields are quoted from the *N*-oxide **2a**, which was formed in quantitative yield from the corresponding amine. It is important to note that isolated yields were typically 5–20% lower than the corresponding assay yields, a discrepancy commonly observed with alkylamines due to challenges associated with recovery during silica gel chromatography. This issue was particularly pronounced in the case of **6a**.

**Figure 5:**
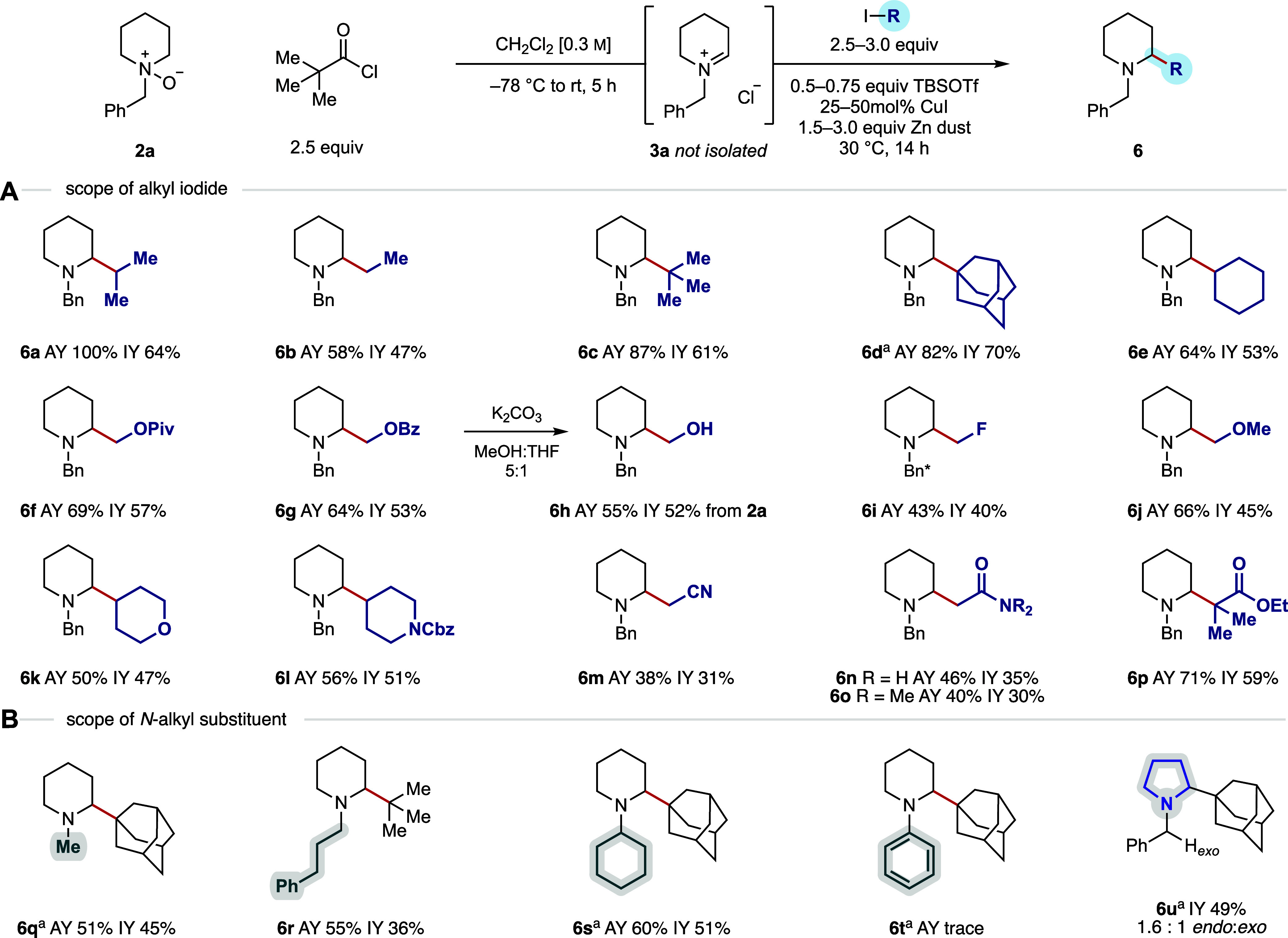
(A) Alkyl iodide and (B) cyclic tertiary alkylamine scope for the one-pot α-alkylation transformation. Reactions were performed on a 0.2 mmol scale using 2.5 equiv PivCl. AY = assay yield determined by ^1^H NMR using 1,1,2,2-tetrachloroethane as an internal standard. IY = isolated yield. *N-(4-Phenylbenzyl) piperidine *N*-oxide **7a** was used as the amine component. ^a^1.5 equiv of copper perchlorate was added to the reaction mixture.

Transformations that introduce small, functionalized alkyl groups directly into lead compounds are desirable for late-stage functionalization applications as these modifications can have a profound impact on biological activity with minimal alteration to the overall structure.^16^ Accordingly, the addition of hydroxymethyl derivatives and a fluoromethyl group to the incipient iminium ion produced the α-substituted piperidines in good yields (**6f**–**i**). Subjecting the crude benzoyl-protected alcohol **6g** to 3 equiv of K_2_CO_3_ furnished the desired α-hydroxymethylated product (**6h**) in good yield across the three-step process from **2a**. Additionally, alkyl iodides bearing an ether (**6j**) or cyano group (**6m**) were well tolerated. Saturated heterocyclic secondary alkyl iodides were also good substrates for the α-functionalization process, producing **6k** and **6l** in good yields. Several α-iodo-carbonyls could be accommodated in the reaction to produce the “aza-Reformatsky”-type products in useful yields (**6n**–**p**).^17^ Throughout this investigation, only the *endo*-cyclic α-functionalized products were observed, highlighting the exceptional regioselectivity of this methodology. However, the assay yields for several scope entries in this two-step process were found to be below 64% (corresponding to an approximate yield of 80% for each individual step). To account for the remaining mass balance, we studied the reaction of **2a** with ethyl iodide (to **6b**); here, 7% of hydroxylammonium salt **5**, 12% of *N*-benzyl-2-(*t-*Bu)-piperidine (**6c**, see the Supporting Information for discussion on its formation), and 12% of a dimeric byproduct (**24**, see the Supporting Information) collectively comprised the remainder of the mass balance.

While the use of *N*-benzyl piperidine provides a means to remove the *exo*-cyclic substituent en route to α-substituted secondary cyclic amines, it is important to note that this reaction can accommodate a range of *N*-alkyl groups. This means that unbiased cyclic tertiary alkylamines are amenable to direct and selective α-functionalization; *N*-methyl (**6q**), *N*-(3-phenyl)­propyl (**6r**), and *N*-cyclohexyl piperidine (**6s**) all gave the desired α-functionalized products selectively in synthetically useful yields, demonstrating a new method for direct modification of these motifs (Figure 5B). Tertiary alkyl iodides were used to explore this set of substrates to ensure the products were not volatile and, therefore, amenable to isolation. Despite the high-yielding formation of the iminium ion derived from *N*-phenylpiperidine (**3e**), the 1,2-addition product **6t** could not be detected and a substantial amount of a dimeric byproduct was isolated (**25**, see the Supporting Information). As expected, *N*-benzyl pyrrolidine *N*-oxide underwent modestly selective α-alkylation to give a mixture of *endo* and *exo* products in a 1.6:1 ratio (**6u**), which could be separated by column chromatography. Owing to the modest selectivity during iminium ion formation, no further examples employing pyrrolidine substrates were pursued. Unfortunately, *N*-alkyl morpholines underwent decomposition regardless of the *exo*-substituent.

The introduction of a methyl group at the α-position of cyclic amines is a highly attractive structural modification to cyclic scaffolds in the context of molecular design. This structural perturbation can affect binding affinity, selectivity, and metabolic stability, without compromising the molecule’s overall properties.^2b,18^ Based on our previous studies, we were surprised to find that attempts to add a methyl group via the Zn-mediated CAA protocol^9e^ delivered the desired product in low and unreliable yields. Instead, other discrete methyl-based organometallic reagents were evaluated using *N*-(4-phenyl)­benzylpiperidine *N*-oxide **7a** as the model substrate (to ensure non-volatile products). Additionally, the lower 2.5 equiv of PivCl was used to minimize any excess interfering with the Grignard-mediated alkylation. Accordingly, the use of methyl magnesium bromide (3 equiv) at 0 °C, in combination with the standard iminium ion formation conditions, furnished the desired α-methylated piperidine derivative (**8**) in good yield (Figure 6A). The trideuterio-methyl group could be introduced in the same way in good yield (to *d*_**3**_ **-8**) and provides a useful tactic for the installation of isotopically labeled groups from readily available feedstocks.^19^

**Figure 6:**
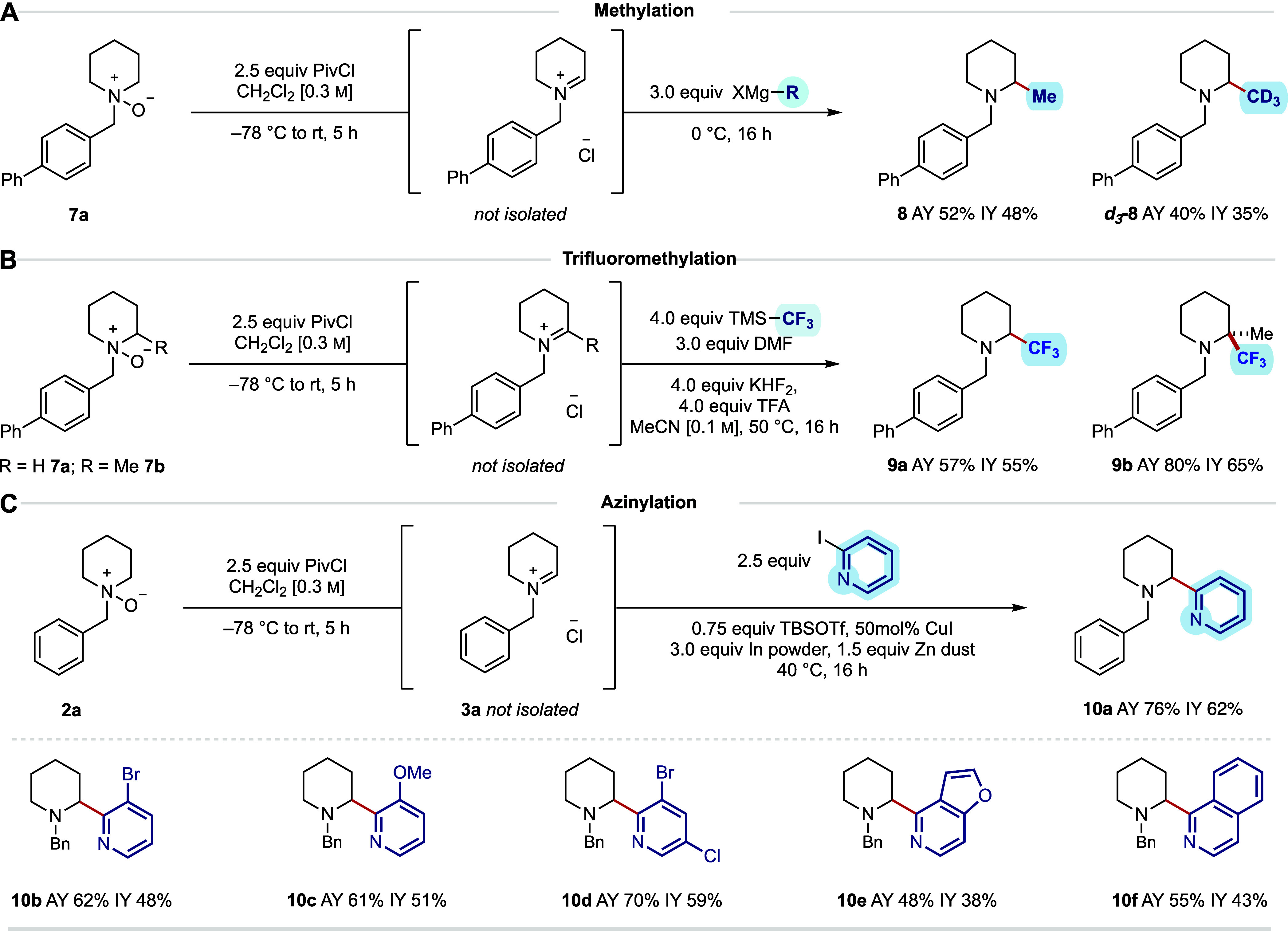
(A) α-Methylation conditions via Grignard addition and scope. (B) α-Trifluoromethylation conditions and scope. (C) α-Heteroarylation conditions and scope with respect to the heteroaryl iodide component. All reactions were performed on a 0.2 mmol scale using 2.5 equiv PivCl. AY = assay yield determined by ^1^H NMR using 1,1,2,2-tetrachloroethane as an internal standard. IY = isolated yield.

Encouraged by the expansion of nucleophile class, we next explored the introduction of trifluoromethyl fragments. The addition of a trifluoromethyl group is widely adopted for late-stage functionalization as it can considerably modulate the basicity and nucleophilicity of the adjacent nitrogen atom and thus influence the molecule’s ionization state *in vivo*.^2e,2f^ While the Zn-mediated CAA protocol was amenable for the introduction of fluoromethyl groups, the addition of the CF_3_ group would not be possible via this method due to the instability of the corresponding organometallic reagent.^2f,20^ However, we reasoned that the use of trimethyl­(trifluoromethyl)­silane (Ruppert–Prakash reagent) would facilitate the nucleophilic addition of trifluoromethyl to the *endo*-cyclic iminium ion under acidic conditions.^21^ Using *N*-oxide **7a**, a process involving application of the now standard iminium formation protocol using 2.5 equiv of PivCl, was followed by *in situ* treatment with TMS-CF_3_, KHF_2_, TFA, and DMF in a solution of acetonitrile, which gave the α-trifluoromethylated tertiary piperidine derivative (**9a**) in excellent yield (Figure 6B). When applied to the *N*-oxide of the 2-methylpiperidine derivative **7b**, α-trifluoromethylation took place at the more substituted position to generate the α-methyl, α-trifluoromethylpiperidine product in excellent yield (**9b**); 12% assay yield of the 2-methyl, 6-trifluoromethylpiperidine product was observed, which reflected a difference in the ratio of the two *endo*-iminium ions. The facile formation of the α,α-disubstituted *N*-alkyl piperidine detailed here would be difficult to accomplish via established protocols from the parent saturated heterocycle and demonstrates the utility of this new approach.

To further extend the range of nucleophiles amenable to this C–H functionalization procedure, its alignment with our recently developed carbonyl azinylative amination (CAzA) manifold was explored.^9f^ While the addition of 2-azinyl groups is perhaps less common in the context of useful late-stage functionalization, its power as a fragment elaboration method or strategically useful carbon–carbon bond formation in the context of target synthesis would make it a useful addition to the synthetic chemist’s tool box of available transformations. Initial investigations into a one-pot α-heteroarylation began with azinylation of **2a** via the *endo*-cyclic iminium ion **3a**, with 2-iodopyridine under the previously developed conditions (1.5 equiv indium powder, 2 equiv 2-iodopyridine, and 1.5 equiv TMSOTf at 70 °C for 16 h). The desired α-azinylated product (**10a**) was formed in a modest 20% yield (Figure 6C). After extensive optimization, the use of substoichiometric amounts of copper­(I) iodide, alongside a reduction in temperature and increase in the equivalency of indium powder, resulted in the formation of **10a** in 55% assay yield (see the Supporting Information). We reasoned that the need for excess metal reductant was due to consumption of indium by the residual pivaloyl chloride from the iminium ion formation step. The impact of other metal reductants was also studied, and zinc dust was found to be effective at promoting the α-heteroarylation of our model substrate. Surprisingly, the combination of indium and zinc led to a reaction furnishing **10a** in 76% assay yield. Here, the protocol required treatment of the intermediate *endo*-cyclic iminium ion with TBSOTf (75 mol %), copper­(I) iodide (50 mol %), heteroaryl iodide (2.5 equiv), indium powder (3.0 equiv), and zinc dust (1.5 equiv) at 40 °C for 16 h. Under these conditions, several heteroaryl iodides were competent substrates in the reaction and generated the corresponding α-azinylated piperidines in good to modest yields (**10b**–**f**).

When considering the reaction of *N*-benzyl-2-methylpiperidine *N*-oxide **7b**, the iminium ion was shown to form at the more substituted *endo*-position in a 86:14 mixture of isomers (see Figure 3 to 3 **f**/**3f′** and Supporting Information). However, when the addition of an ethyl fragment was carried out under the Zn-mediated conditions, none of the expected α,α-(disubstituted) product was detected and the reaction had occurred exclusively at the unsubstituted *endo*-cyclic iminium ion to form 16% assay yield (12% yield of isolated product) of the 2-methyl, 6-ethylpiperidine derivative **11**, with excellent selectivity (Figure 7A, *left-hand side*). In contrast, when **7b** was subjected to the same iminium ion formation conditions and then treated with ethyl magnesium bromide, the 2-methyl, 2-ethylpiperidine derivative **12** was formed in a good assay yield of 76%, accompanied by 16% of **11**. The selective reaction of **7b** with the Grignard reagent was in line with that observed for the corresponding trifluoromethylation to form α-Me, α-CF_3_-piperidine **9b** (see Figure 6B), and in contrast to the Zn/Cu-mediated process forming **11**. Together, these findings suggest that a mechanistic difference may be operating during the 1,2-addition steps of the Zn-mediated and Grignard/trifluoromethylation processes that are possibly the result of radical and polar pathways, respectively. Regardless of the reason, this is a further demonstration of the complementary reactivity exhibited by Grignard reagents compared to the Zn-based alkyl nucleophiles and substantially increases the broader applicability of this C–H alkylation protocol.

**Figure 7:**
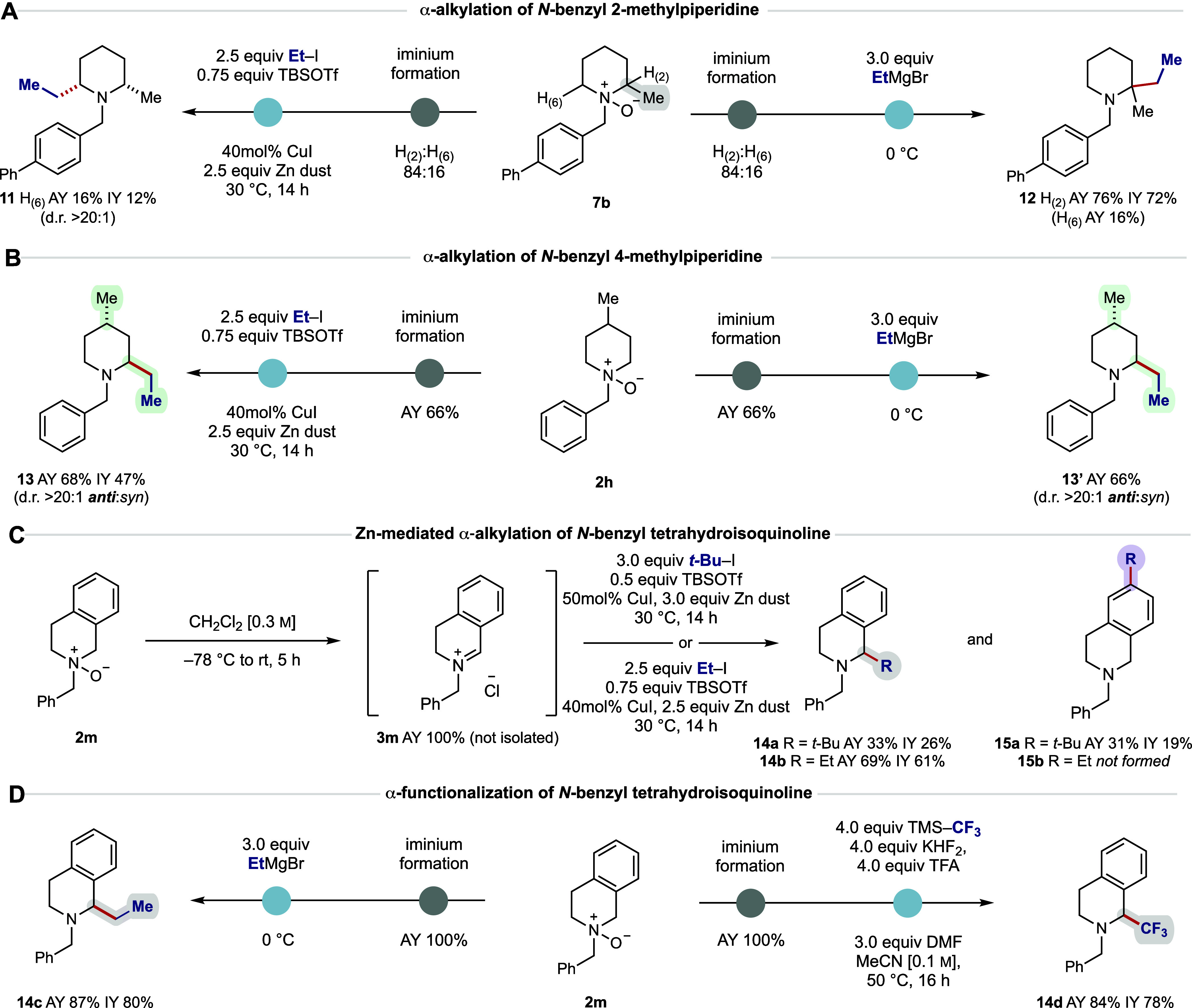
(A) Regioselectivity in the α-functionalization of *N*-benzyl-2-methylpiperidine via Zn-mediated and Grignard-based alkylation. (B) Diastereoselectivity observed in the α-functionalization of *N*-benzyl 4-methylpiperidine via Zn-mediated and Grignard-based alkylation. (C) Poorly selective alkylation of *N*-benzyl tetrahydroisoquinoline under Zn-mediated conditions. (D) Highly selective α-functionalization of *N*-benzyl tetrahydroisoquinoline under Grignard-based alkylation and trifluoromethylation. All reactions were performed on a 0.2 mmol scale using 2.5 equiv PivCl. AY = assay yield determined by ^1^H NMR using 1,1,2,2-tetrachloroethane as an internal standard. IY = isolated yield.

To further test the selectivity differences in reactivity between the Zn-mediated and Grignard-mediated processes, 4-methylpiperidine derivative **2h** was evaluated to probe the reaction’s intrinsic diastereoselectivity (Figure 7B). Zn-mediated addition of an ethyl group to the intermediate iminium ion (from **2h**) generated a good assay yield of the desired α-functionalized product **13** with >20:1 selectivity for the *anti*-diastereomer. In contrast to previous differences, deployment of ethyl magnesium bromide resulted in the selective formation of **13′**, again in good assay yield. These results are consistent with axial attack of the nucleophile to a half chair iminium ion with the remaining substituent occupying an equatorial (or pseudo-equatorial) position.^22^

During our investigations of different piperidine-derived substrates, we found that the reaction of *N*-benzyl tetrahydroisoquinoline *N*-oxide **2m** with *tert*-butyl iodide under the Zn-mediated conditions resulted in a low yield of the desired product **14a**, albeit with the expected *endo*-selectivity (Figure 7C). However, we noted the formation of an isomeric byproduct **15a** (that was not the *exo*-alkylation) that resulted from the net reductive addition of a *t*-Bu group in the *para*-position to the iminium ion **3m**. This outcome could be explained by a steric effect wherein the bulky *t*-Bu nucleophile adds to the more accessible and electrophilically activated “*para*”-position to the iminium ion. To confirm this, we tested the reaction of Et–I, which forms a sterically unencumbered alkyl nucleophile, under the appropriate Zn/Cu CAA conditions and found that only **14b** was formed, suggesting that the unusual selectivity observed for the *t*-Bu system is simply a result of steric effects. In line with previous observations, the alkylation reaction using ethyl magnesium bromide resulted in the desired 1,2-addition product **14c** in excellent yield (Figure 7D). The corresponding C–H trifluoromethylation also worked well to generate the α-functionalized product **14d** in high yield.

Armed with a strategy for the introduction of unactivated carbon-based alkyl groups to the *endo*-α-position of *N-*alkyl piperidines, we questioned whether the reaction could be applied to the late-stage modification of bioactive molecules. Several of the α-functionalization protocols were evaluated using dextromethorphan as a representative example of a complex cyclic tertiary alkylamine (Figure 8A).^6a,6r^ ^23^ Oxidation to dextromethorphan *N*-oxide proceeded in good yield upon treatment with *m*CPBA. Despite having multiple α-C–H bonds around the nitrogen atom, a single *endo*-iminium ion (**16**) was formed in good yield (>20:1 r.r.) using the standard optimized reaction conditions. Several alkyl groups, comprising methyl, *iso*-propyl, hydroxymethyl, and fluoromethyl, could be added to this iminium ion to form the corresponding *endo* α-substituted dextromethorphan products in good yields over this two-step process (**17a**–**f**). Interestingly, methylation (to **17a**), *iso*-propylation (to **17b**), and fluoromethylation (to **17d**) were not only exquisitely regioselective but also diastereoselective, affording single isomers. The hydroxymethylation reaction (to **17c**), which utilized the Zn-mediated protocol, was regioselective but displayed little diastereoselectivity, though the isomers were separable by column chromatography; the α-trifluoromethylation to **17f** displayed a diastereoselectivity of 4:1 with the major isomer depicted . While less of a classical late-stage modification, the α-azinylation (to **17e**) provides a useful fragment building protocol wherein more elaborate groups can be added to a complex framework as a means for a more target-oriented synthesis application. Structures related to dextromethorphan have been reported to undergo the Polonovski–Potier reaction with *endo*- or *exo*-selectivity depending on the reaction conditions employed.^24^ What is significant about this new transformation is the diversity of functional groups that can be added to this scaffold without compromising the *endo*-selectivity.

**Figure 8:**
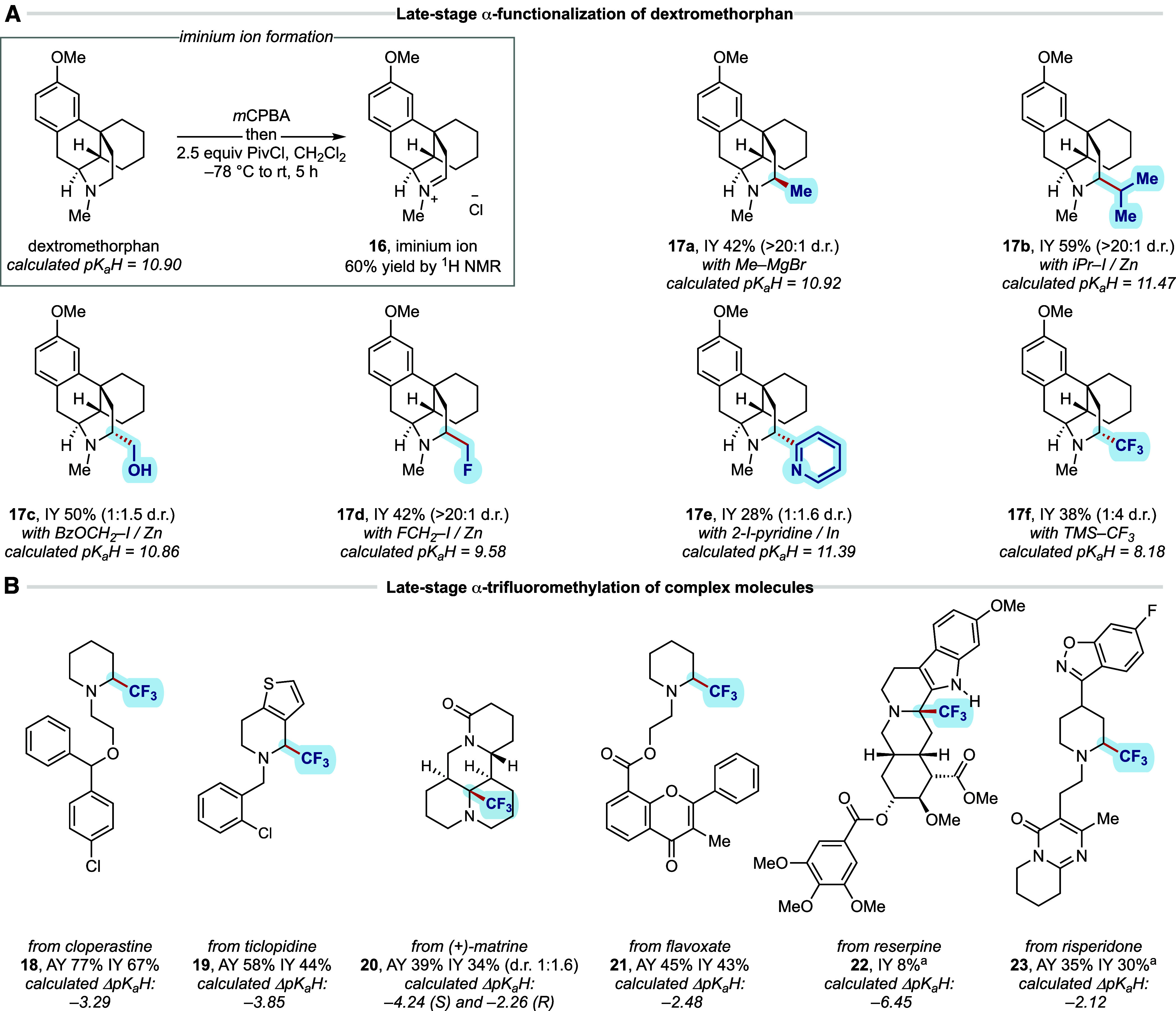
(A) Late-stage α-functionalization of dextromethorphan and (B) late-stage α-trifluoromethylation scope with respect to the complex cyclic tertiary alkylamine component. All reactions were performed on a 0.2 mmol scale using 2.5 equiv PivCl. AY = assay yield determined by ^1^H NMR using 1,1,2,2-tetrachloroethane as an internal standard. IY = isolated yield. ^a^Isolated as the TFA salt.

Many of these structural changes represent common α-modifications made to amine scaffolds in pharmaceutical discovery campaigns. This is often because the p*K*_a_H of the candidate is a key physicochemical parameter that influences many of its biopharmaceutical characteristics.^25^ Therefore, modifications that impact a molecule’s basicity will also impact its associated physicochemical and biological properties. Accordingly, computational modeling using open access software (developed by Rowan Scientific Corporation)^26^ was employed to predict the p*K*_a_H of dextromethorphan (predicted p*K*_a_H 10.90) and its α-functionalized derivatives. As expected, these α-modifications had a significant impact on the predicted p*K*_a_Hs, modulating the amine basicity from 8.18 to 11.47 and highlighting the power of this transformation to fine-tune the physicochemical properties of pharmaceutical candidates.

It was also found that several piperidine-containing natural products and marketed pharmaceuticals underwent the α-trifluoromethylation transformation to form products that would be difficult to obtain by other means, which further highlighted the functional group tolerance of this methodology (Figure 8B). Therein, selective oxidation of the tertiary amines with *m*CPBA proceeded efficiently, producing the corresponding tertiary amine *N*-oxides in good yields (see the Supporting Information). Elimination to form the corresponding iminium ions proceeded as expected under the standard reaction conditions, which were directly subjected to the trifluoromethylation reaction. Accordingly, a selection of piperidine-containing drugs and natural products—cloperastine, ticlopidine, (+)-matrine, and flavoxate—all underwent the α-trifluoromethylation process (to **18**–**21**) from the corresponding *N*-oxides in good yields. The α-trifluoromethylation of the complex indole alkaloid, reserpine, produced a single α-functionalized product (to **22**), albeit in modest yield. The corresponding enamine was instead identified as a major byproduct, suggesting that the iminium ion intermediate was not converted in the functionalization step. While this example does not exhibit typical *endo*- and *exo*-positions, it serves as a valuable illustration of functional group tolerance. Finally, risperidone also delivered the desired α-functionalized product (**23**) in good yield. Even though the yields, in some cases, were modest, this strategy enables streamlined access to the functionalized products in synthetically usable yields, the likes of which would be challenging to generate by established protocols. As expected, each example of α-trifluoromethylation endowed significant changes in the molecule’s predicted p*K*_a_H.

## Conclusions

In summary, we have devised a general platform for the α-functionalization of structurally simple or complex *N*-alkyl piperidines, via sequential iminium ion formation and subsequent 1,2-addition of a range of carbon-based nucleophiles. A set of conditions have been developed for the regioselective formation of *endo*-cyclic iminium ions in piperidine ring systems by controlling α-C–H elimination. Subsequent functionalization of these iminium ions in a one-pot process facilitated the α-alkylation and α-heteroarylation of a wide variety of *N*-alkyl piperidines. Excellent functional group compatibility and regioselectivity allowed for the late-stage modification of complex bioactive molecules, delivering α-methylated, α-trifluoromethylated, α-fluoromethylated, and α-hydroxymethylated analogs, which demonstrates a potentially useful platform for the exploration of drug-like chemical space.

## Supplementary Material



## Data Availability

The data underlying this study are available in the published article and its online Supporting Information

## Notes

All experimental procedures, DFT calculations, and spectroscopic data for novel compounds (PDF)
