# Supplementary material for: Selective endo-Cyclic α‑Functionalization of Saturated N‑Alkyl Piperidines
Source: J Org Chem. 2025 Aug 18;90(34):12226–39. doi: 10.1021/acs.joc.5c01742 (PMC12400421; doi:10.1021/acs.joc.5c01742)

Supplementary Information for

Selective *Endo*-Cyclic α-Functionalization of Saturated *N*-Alkyl Piperidines

Rachel C. Phillips, John C. K. Chu, Alex A. Rafaniello and Matthew J. Gaunt*

Yusuf Hamied Department of Chemistry, University of Cambridge, Lensfield Road, Cambridge, CB2 1EW, United Kingdom.

Correspondence to: mjg32@cam.ac.uk

Table of contents

1. GENERAL INFORMATION SI-3

1.1 Materials and methods ---------- SI-3
1.2 General comments and practical guide for reaction ---------- SI-5

2. GENERAL PROCEDURES SI-7

2.1 General procedure 1: Iminium ion formation ---------- SI-7
2.2 General procedure 2: Zn mediated α-alkylation ---------- SI-8
2.3 General procedure 3: Zn mediated α-alkylation 2 ---------- SI-9
2.4 General procedure 4: Grignard-based α-alkylation ---------- SI-10
2.5 General procedure 5: α-Trifluoromethylation ---------- SI-11
2.6 General procedure 6: α-Azinylation ---------- SI-12
2.7 General procedure 7: *N*-oxide synthesis ---------- SI-13

3. REACTION OPTIMIZATION SI-14

3.1 *Selective endo*-cyclic iminium ion formation ---------- SI-14
3.2 Zn mediated α-alkylation ---------- SI-18
3.3 Grignard mediated α-alkylation ---------- SI-27
3.4 α-Trifluoromethylation ---------- SI-28
3.5 α-Azinylation ---------- SI-29

4. IMINIUM ION SCOPE SI-31

5. α-FUNCTIONALIZED CYCLIC AMINE SCOPE SI-41

5.1 Zn mediated alkylation scope ---------- SI-41
5.2 Grignard mediated α-alkylation scope ---------- SI-58
5.3 Trifluoromethylation scope ---------- SI-61
5.4 Azinylation scope ---------- SI-64
5.5 Late-stage α-derivatisation scope ---------- SI-67
5.6 Additional substrates ---------- SI-83
5.7 Problematic substrates ---------- SI-104

6. STARTING MATERIAL SYNTHESIS SI-107

6.1 Tertiary amines ---------- SI-107
6.2 Amine *N*-oxides ---------- SI-110
6.3 Other ---------- SI-125

7. COMPUTATIONAL CALCULATIONS SI-127

7.1 General information ---------- SI-127
7.2 Predicting regioselectivity using DFT ---------- SI-127
7.3 DFT computed transition states for the regiodetermining step ---------- SI-131
7.4 Predicted pK_aH data ---------- SI-137

8. REFERENCES SI-140

9. NMR SPECTRAL DATA SI-144

1. General information

1.1 Materials and methods

All reactions were fitted with a magnetic stirrer bar and conducted under an inert atmosphere (N_2) unless otherwise specified. Glassware was oven-dried prior to use. Anhydrous solvents (CH_2Cl_2, DMF, MeCN, EtOAc) were purchased from Aros Organics (99.5%, <50 ppm water). Deuterated solvents (Acetone-d_6, CH_2Cl_2-d_2, DMSO-d_6, MeCN-d_3, benzene-d_6, toluene-d_8 and $CHCl_3$-d) were purchased from Sigma Aldrich as 1 mL or 0.75 mL anhydrous ampoules. PE refers to the fraction of petroleum ether collected between 40–60 ˚C. Pivaloyl chloride was distilled under N_2 and stored in a sealed microwave at 5 ˚C under N_2. Zinc dust (<10 µm, 98%) and indium powder (99.99% trace metal basis) were purchased from Sigma-Aldrich, free from anti-caking agents. Copper(I) iodide (synthesis grade) was purchased from Sigma-Aldrich. All other commercial reagents (amines, aldehydes, ketones, etc.) were used as supplied if sufficiently pure, otherwise they were purified either by distillation or flash column chromatography and used immediately.

Analytical thin layer chromatography (TLC) was carried out using Merck silica gel 60 F254 pre-coated glass plates (0.2 nm) and visualised with UV light (λ_{max} = 254 nm) and/or by staining with basic aqueous $KMnO_4$. Flash column chromatography was performed using silica gel (Merck Geduran Si 60 [40–65 µm]) or using a Teledyne CombiFlash NextGen 300+ equipped with RediSep Silver Si Rf columns (40–60 µm, normal phase), RediSep Gold Si Rf (20–40 µm, normal phase) or RediSep Gold C18 Rf columns (20–40 µm, reverse phase) with the indicated solvent system.

Strong cation exchange (SCX) chromatography was performed using HyperSep SCX cartridges (500 – 1000 mg bed weight) from Thermo Scientific. Prior to purification, SCX cartridges were primed by passing through MeOH (15 mL). SCX purification was conducted by loading the crude sample as a solution in MeOH onto the primed cartridge. After washing with MeOH (15 mL), the retained product was eluted using a 7 N solution of NH_3 in MeOH (15 mL). The eluent was concentrated *in vacuo* to yield the desired product.

NMR spectra were recorded in deuterated solvent, using a Bruker AM-400 (400 MHz), Avance 500 (500 MHz) or Avance 700 (700 MHz) spectrometer at 298 K unless otherwise stated. Chemical shifts (δ) are reported in parts per million (ppm) relative to tetramethylsilane and were referenced to the residual protic solvent signals ($CDCl_3$ = 7.26 ppm; CD_2Cl_2 = 5.32 ppm; MeOD = 3.31 ppm). Coupling constants (J) are reported in Hertz (Hz) to the nearest 0.1. Abbreviations for splitting patterns are as follows: singlet (s), doublet (d), triplet (t), quartet (q), multiplet (m) apparent (app.) and broad (br). Data is reported as follows: chemical shift (multiplicity, integration, coupling constant). ^{13}C NMR signals were recorded with complete proton decoupling and are reported in ppm relative to tetramethylsilane. Signals were referenced to the residual solvent signals ($CDCl_3$ = 77.2 ppm; CD_2Cl_2 = 53.8 ppm; MeOD = 49.0). Assay yields were determined by ^1H NMR analysis of crude reaction mixtures with reference to 1,1,2,2-tetrachloroethane as an internal standard.

Melting point (m.p.) were recorded using a Gallenkamp melting point apparatus and are reported uncorrected. IR spectra were recorded on a Thermo Fisher Scientific Nicolet Summit PRO FTIR equipped with a diamond ATR module; absorption maxima (ν_{max}) are reported in wavenumbers (cm^{-1}). Samples were applied as solids or films, either through direct application or deposited as a solution in $CHCl_3$.

High-resolution mass spectrometry (HRMS) experiments were carried out using a Shimadzu LCMS-9030 Q-TOF mass spectrometer using electrospray ionisation (ESI) techniques at the Department of Chemistry. Liquid chromatography mass spectrometry (LCMS) experiments were carried out using a Shimadzu LCMS-2020 single quadrupole spectrometer using ESI techniques.

Preparative high-performance liquid chromatography (HPLC) was carried out using a Shimadzu LC-20AR equipped with a SPD-40 UV-Vis detector and a reverse-phase C18 Luna® column (150 mm x 10 mm, 5 µm), eluting with a gradient of 40-95% solvent A-solvent B over 25 mins (solvent A: 0.05% (v/v) TFA in H_2O, solvent B: 0.05% TFA in MeCN). Product containing fractions were lyophilized to give the pure product.

Compound names are those generated by PerkinElmer ChemDraw Professional v. 22.2.0.3348 according to IUPAC nomenclature.

1.2 General comments and practical guide for reaction

General comments:

- Iminium ion formation is sensitive to moisture and, therefore, glassware should be oven-dried before use and an inert atmosphere should be maintained. The α-alkylation and α-heteroarylation reactions are also sensitive to air, thus an inert atmosphere must be maintained throughout the reaction.

- Vigorous stirring during the α-alkylation and α-heteroarylation reactions (>1200 rpm) is required for successful results.

- The α-alkylation reaction is sensitive to reaction time. Best yields were obtained when reactions were run for 14 hours and no longer than 16 hours. Beyond this, significant decreases in yield were observed.

- Vigorous stirring during the aqueous workup (>1200 rpm), resulting in the aqueous and organic layers becoming 'miscible', is required for successful results in the α-alkylation and α-heteroarylation reactions. Additionally, best results were generally obtained when the reaction mixture was quenched with 40% NaOH for at least 2 hours, or until the aqueous layer appeared blue, whichever was longer.

- It is imperative that the order of addition for the α-alkylation and α-heteroarylation reactions is as follows: i) TBSOTf, ii) copper iodide, iii) alkyl or aryl iodide, iv) zinc dust, and for the α-heteroarylation v) indium powder. Significantly diminished yields were observed when the order of addition deviated from above.

- The reaction is not overly sensitive to the type or size of flask (microwave vial, round bottom flask, etc.), though slightly better yields were obtained in 10 mL round bottom flasks.

- It was noted that with most examples, isolated yields were 5-15% lower than the assay yields. This is commonly encountered with alkylamines and is an issue with recovery from silica gel chromatography.

Step-by-step guide for α-alkylation reaction:

- A 10 mL RBF was charged with a 10 mm magnetic stirrer bar and placed under vacuum. The RBF was dried with a heat gun for 2 minutes and then allowed to cool to rt before being placed under an N_2 atmosphere.
- To the RBF was added pivaloyl chloride (62 µL) and anhydrous CH_2Cl_2 (0.25 mL) and the mixture was stirred (at 600 rpm).

- Simultaneously, the alkylamine *N*-oxide was weighed into a 4 mL screw cap vial. The vial was then sealed, placed under an N_2 atmosphere, and anhydrous CH_2Cl_2 (0.25 mL) was added to dissolve the *N*-oxide.
- Both the stirring RBF (with pivaloyl chloride) and vial (with *N*-oxide) were cooled to –78 ˚C in a dry ice/acetone bath for 15 mins.
- After this time, the vial containing the *N*-oxide solution was removed from the ice bath. The solution was taken up in a 1 mL syringe and added dropwise to the pivaloyl chloride solution in the RBF.
- To the same 4 mL screw gap vial was added anhydrous CH_2Cl_2 (0.2 mL). The vial was shaken and then cooled back to –78 ˚C.
- After 15 mins, the vial was removed from the dry ice/acetone bath, and the solution was taken up in the same 1mL syringe as before and added dropwise to the pivaloyl chloride solution in the RBF.
- The mixture was left stirring for 15 mins at –78 ˚C. After which, the dry ice was removed from the dry ice/acetone bath. The RBF was left in the cold acetone bath to warm to rt.
- After 4 h, water was added to the acetone bath to increase the temperature from approx. 0 ˚C to 10 – 15 ˚C. After a further 30 mins, the RBF was removed from the acetone bath and allowed to warm to rt for another 30 mins.
- After this time, the stirring was increased to 1200 rpm and TBSOTf was added *via* micro syringe.
- The RBF was unsealed, and copper iodide was added quickly to minimise disruption to the N_2 atmosphere, after which the RBF was resealed.
- Next, the alkyl iodide was added *via* micro syringe. The RBF was then unsealed again, zinc dust was added quickly, and the RBF was resealed.
- The reaction mixture was heated to 30 ˚C and vigorous stirring was maintained (>1200 rpm) overnight for 14 h under an N_2 atmosphere.
- After this time, the mixture was transferred to a 50 mL RBF charged with a 24 mm stirrer bar with CH_2Cl_2 (20 mL) and 40% NaOH solution (20 mL). The resulting mixture was stirred vigorously (>1200 rpm, so that the organic and aqueous layers were 'miscible') for a minimum of 2 h.
- The aqueous layer was extracted with CH_2Cl_2 (3 x 20 mL), dried over Na_2SO_4, filtered, and concentrated *in vacuo* to afford the crude α-alkylated cyclic alkylamine.

2. General procedures

2.1 General procedure 1: Iminium ion formation

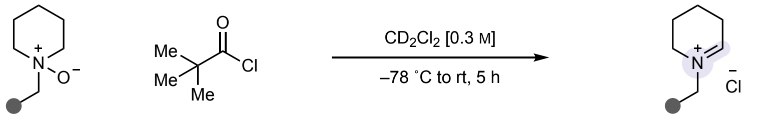

2 6.6 equiv. 3 *endo*-iminium ion

A solution of pivaloyl chloride (163 µL, 1.32 mmol, 6.6 equiv) in anhydrous CD_2Cl_2 (0.25 mL, 0.8 M) was cooled to –78 °C. To this was added dropwise a solution of alkylamine *N*-oxide (0.20 mmol, 1.0 equiv) in anhydrous CD_2Cl_2 (0.25 mL, 0.8 M), which was also cooled to –78 ˚C. The vial containing the *N*-oxide was washed with CD_2Cl_2 (0.20 mL), cooled to –78 ˚C and added dropwise to the pivaloyl chloride solution. After complete addition, the mixture was stirred for a further 15 mins at –78 ˚C before warming slowly to rt over 5 h.

NB: These intermediates are extremely unstable in the presence of moisture; hence they are generated in situ and either used directly in the next step or analysed immediately by NMR.

2.2 General procedure 2: Zn mediated α-alkylation

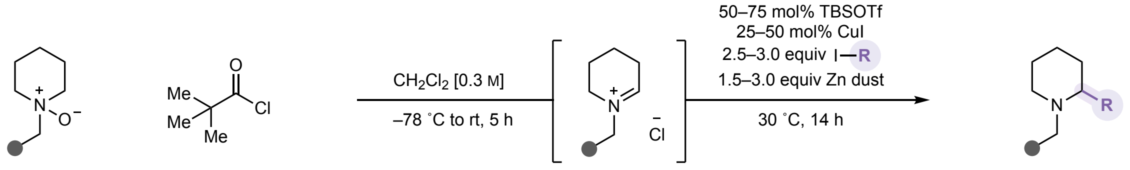

2 2.5 equiv. *not isolated* **6**

A solution of pivaloyl chloride (62 μL, 0.50 mmol, 2.5 equiv) in anhydrous CH_2Cl_2 (0.25 mL, 0.8 M) was cooled to –78 °C. To this was added dropwise a solution of alkylamine *N*-oxide (0.20 mmol, 1.0 equiv) in anhydrous CH_2Cl_2 (0.25 mL, 0.8 M), which was also cooled to –78 ˚C. The vial containing the *N*-oxide was washed with CH_2Cl_2 (0.20 mL), cooled to –78 ˚C and added dropwise to the pivaloyl chloride solution. After complete addition, the mixture was stirred for a further 15 mins at –78 ˚C before warming slowly to rt over 5 h.

To this vigorously stirring solution was added TBSOTf (50–75 mol%) followed by copper iodide (25–50 mol%), alkyl iodide (2.5–3.0 equiv), and zinc dust (1.5–3.0 equiv) in that order. The reaction mixture was heated to 30 ˚C using an oil bath and vigorously stirred for 14 h. The crude reaction mixture was cooled and diluted with CH_2Cl_2 (20 mL), 40% (w/w) NaOH solution (20 mL) was added, and the resultant mixture was stirred vigorously for a further 2 h. The aqueous layer was extracted with CH_2Cl_2 (3 x 20 mL), and the combined organic layers were dried over Na_2SO_4, filtered, and concentrated *in vacuo* to afford the crude α-alkylated cyclic alkylamine.

Condition 1 (primary alkyl iodides):
75 mol% TBSOTf; 40 mol% copper iodide, 2.5 equiv alkyl iodide; 2.5 equiv zinc dust.

Condition 2 (secondary alkyl iodides):
75 mol% TBSOTf; 25 mol% copper iodide, 2.5 equiv alkyl iodide; 1.5 equiv zinc dust.

Condition 3 (tertiary alkyl iodides):
50 mol% TBSOTf; 50 mol% copper iodide, 3.0 equiv alkyl iodide; 3.0 equiv zinc dust.

2.3 General procedure 3: Zn mediated α-alkylation 2

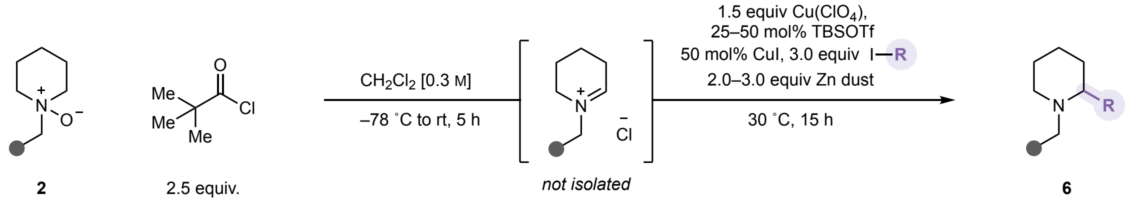

A solution of pivaloyl chloride (62 μL, 0.50 mmol, 2.5 equiv) in anhydrous CH_2Cl_2 (0.25 mL, 0.8 M) was cooled to –78 °C. To this was added dropwise a solution of alkylamine *N*-oxide (0.20 mmol, 1.0 equiv) in anhydrous CH_2Cl_2 (0.25 mL, 0.8 M), which was also cooled to –78 ˚C. The vial containing the *N*-oxide was washed with CH_2Cl_2 (0.20 mL), cooled to –78 ˚C and added dropwise to the pivaloyl chloride solution. After complete addition, the mixture was stirred for a further 15 mins at –78 ˚C before warming slowly to rt over 5 h.

After warming to rt, copper perchlorate (78.2 mg, 0.30 mmol, 1.5 equiv) was added and the mixture was stirred vigorously for 15 mins at rt.

Next, TBSOTf (25–50 mol%) was added, followed by copper iodide (19.0 mg, 0.10 mmol, 50 mol%), alkyl iodide (0.60 mmol, 3.0 equiv), and zinc dust (2.0–3.0 equiv) in that order. The reaction mixture was heated to 30 ˚C using an oil bath and vigorously stirred for 15 h. The crude reaction mixture was cooled and diluted with CH_2Cl_2 (20 mL), 40% (w/w) NaOH solution (20 mL) was added, and the resultant mixture was stirred vigorously for a further 3 h. The aqueous layer was extracted with CH_2Cl_2 (3 x 20 mL), and the combined organic layers were dried over Na_2SO_4, filtered, and concentrated *in vacuo* to afford the crude α-alkylated cyclic alkylamine.

Condition 1 (primary and tertiary alkyl iodides):
50 mol% TBSOTf; 50 mol% copper iodide, 3.0 equiv alkyl iodide; 3.0 equiv zinc dust.

Condition 2 (secondary alkyl iodides):
25 mol% TBSOTf; 50 mol% copper iodide, 3.0 equiv alkyl iodide; 2.0 equiv zinc dust.

2.4 General procedure 4: Grignard-based α-alkylation

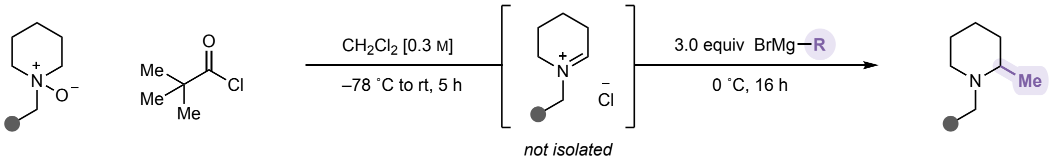

7 2.5 equiv. **8**

A solution of pivaloyl chloride (62 μL, 0.50 mmol, 2.5 equiv) in anhydrous CH_2Cl_2 (0.25 mL, 0.8 M) was cooled to –78 °C. To this was added dropwise a solution of alkylamine *N*-oxide (0.20 mmol, 1.0 equiv) in anhydrous CH_2Cl_2 (0.25 mL, 0.8 M), which was also cooled to –78 ˚C. The vial containing the *N*-oxide was washed with CH_2Cl_2 (0.20 mL), cooled to –78 ˚C and added dropwise to the pivaloyl chloride solution. After complete addition, the mixture was stirred for a further 15 mins at –78 ˚C before warming slowly to rt over 5 h.

The reaction mixture was cooled back to 0 °C and Grignard reagent (3.0 equiv) was added dropwise. The reaction was allowed to warm slowly to rt whilst stirring for 16 h. The crude reaction mixture was diluted with CH_2Cl_2 (20 mL), water (20 mL) was added, and the resultant mixture was stirred vigorously for 20 mins. The aqueous layer was extracted with CH_2Cl_2 (3 x 20 mL), and the combined organic layers were dried over Na_2SO_4, filtered, and concentrated *in vacuo* to afford the crude α-methylated cyclic alkylamine.

2.5 General procedure 5: α-Trifluoromethylation

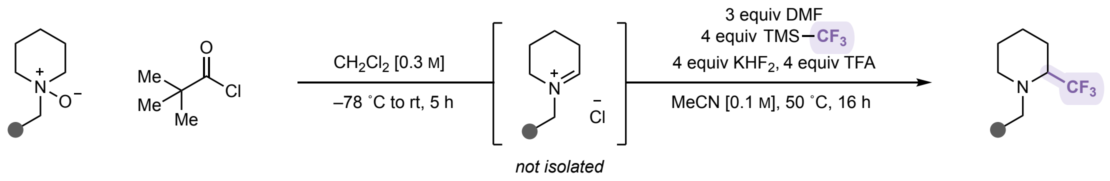

7 2.5 equiv. **9**

A solution of pivaloyl chloride (62 µL, 0.50 mmol, 2.5 equiv) in anhydrous CH_2Cl_2 (0.25 mL, 0.8 M) was cooled to –78 °C. To this was added dropwise a solution of alkylamine *N*-oxide (0.20 mmol, 1.0 equiv) in anhydrous CH_2Cl_2 (0.25 mL, 0.8 M), which was also cooled to –78 ˚C. The vial containing the *N*-oxide was washed with CH_2Cl_2 (0.20 mL), cooled to –78 ˚C and added dropwise to the pivaloyl chloride solution. After complete addition, the mixture was stirred for a further 15 mins at –78 ˚C before warming slowly to rt over 5 h.

To this vigorously stirring solution was added acetonitrile (2 mL, 0.1 M), DMF (47 µL, 0.60 mmol, 3.0 equiv), trimethyl(trifluoromethyl)silane (118 µL, 0.80 mmol, 4.0 equiv) and potassium bifluoride (62.5 mg, 0.80 mmol, 4.0 equiv) followed by trifluoroacetic acid (61 µL, 0.80 mmol, 4.0 equiv). The reaction mixture was heated to 50 ˚C using an oil bath and vigorously stirred for 16 h. The crude reaction mixture was cooled and diluted with CH_2Cl_2 (20 mL), sat. $NaHCO_3$ solution (20 mL) was added, and the resultant mixture was stirred vigorously for 20 mins. The aqueous layer was extracted with CH_2Cl_2 (3 x 20 mL), and the combined organic layers were dried over Na_2SO_4, filtered, and concentrated *in vacuo* to afford the crude α-trifluoromethylated cyclic alkylamine.

2.6 General procedure 6: α-Azinylation

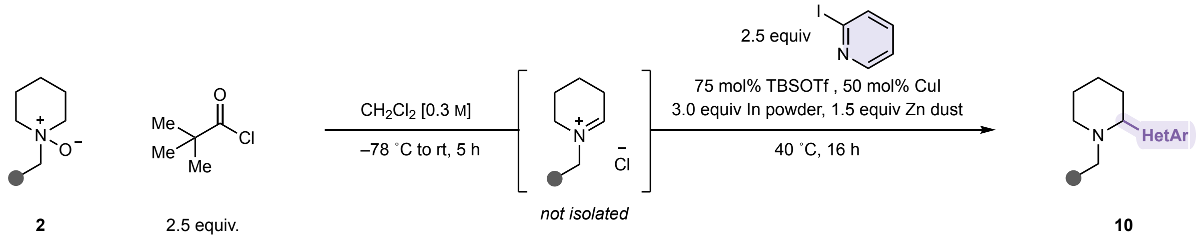

2 2.5 equiv. **10**

A solution of pivaloyl chloride (62 μL, 0.50 mmol, 2.5 equiv) in anhydrous CH_2Cl_2 (0.25 mL, 0.8 M) was cooled to –78 °C. To this was added dropwise a solution of alkylamine *N*-oxide (0.20 mmol, 1.0 equiv) in anhydrous CH_2Cl_2 (0.25 mL, 0.8 M), which was also cooled to –78 ˚C. The vial containing the *N*-oxide was washed with CH_2Cl_2 (0.20 mL), cooled to –78 ˚C and added dropwise to the pivaloyl chloride solution. After complete addition, the mixture was stirred for a further 15 mins at –78 ˚C before warming slowly to rt over 5 h.

To this vigorously stirring solution was added TBSOTf (34 µL, 0.15 mmol, 75 mol%), followed by copper iodide (19.0 mg, 0.10 mmol, 50 mol%), aryl iodide (2.5 equiv) and zinc dust (19.6 mg, 0.30 mmol, 1.5 equiv) in that order. After 10 mins, indium powder (68.9 mg, 0.60 mmol, 3.0 equiv) was added and reaction mixture was heated to 40 ˚C using an oil bath and with vigorous stirring for 16 h. The crude reaction mixture was cooled and diluted with CH_2Cl_2 (20 mL), 40% (w/w) NaOH solution (20 mL) was added, and the resultant mixture was stirred for a further 2 h. The aqueous layer was extracted with CH_2Cl_2 (3 x 20 mL), and the combined organic layers were dried over Na_2SO_4, filtered, and concentrated *in vacuo* to afford the crude α-heteroarylated cyclic alkylamine.

2.7 General procedure 7: *N*-oxide synthesis

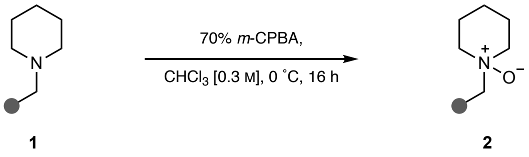

Adapted from the literature procedure by Laksham. [28]

To a stirred solution of amine (1.0 equiv) in $CHCl_3$ [0.15 M] at 0 °C was added 70% *m*-CPBA (1.2 equiv). The resultant mixture was stirred at rt for 16 h. Next, solid anhydrous K_2CO_3 (4.5 equiv) was added, and the resultant mixture was stirred for a further 30 mins. The solid was removed *via* vacuum filtration and the filtrate was dried over Na_2SO_4, filtered, and concentrated *in vacuo* to afford the crude alkylamine *N*-oxide.

3. Reaction optimization

3.1 *Selective endo*-cyclic iminium ion formation

Table S1: Survey of solvents for selective *endo*-cyclic iminium ion formation

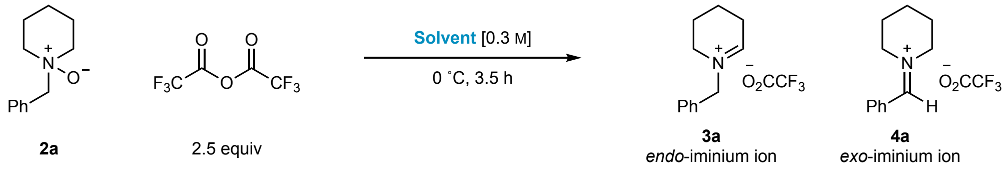

Entry	Solvent	Combined assay yield / %[a]	*Endo* : *Exo* selectivity
**1**	Dichloromethane-d_2	82	5.8 : 1
**2**	Acetone-d_6	65	2.8 : 1
**3**	Acetonitrile-d_3	90	5.0 : 1
**4**[b]	Benzene-d_6	88	5.7 : 1
**5**	Chloroform-*d*	74	5.7 : 1
**6**	Toluene-d_8	96	5.0 : 1

Reactions carried out using 0.20 mmol of *N*-oxide **2a** and 0.50 mmol of TFAA in deuterated solvents. A solution of **2a** in CD_2Cl_2 was cooled to 0 °C before the dropwise addition of TFAA. [a]Assay yields of **3a/4a** were determined by ^{1}H NMR using 1,1,2,2-tetrachloroethane as an internal standard. [b]Reaction was conducted at 10 °C.

Table S2: Survey of acetylating agents for selective *endo*-cyclic iminium ion formation

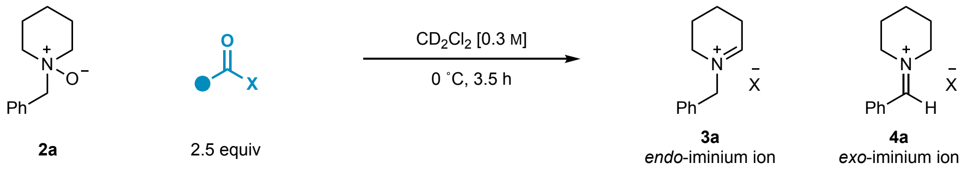

Entry	Acetylating agent	pK_aH of conjugate anion[a]	Combined assay yield / %[b]	*Endo* : *Exo* selectivity
**1**	Acetic anhydride	4.75	0	-
**2**	Chloroacetic anhydride	2.87	10	0.1 : 1
**3**	Difluoroacetic anhydride	1.24	17	4.6 : 1
**4**	Trifluoroacetic anhydride	0.52	82	5.8 : 1
**5**	Acetyl chloride	–7.00	58	>20 : 1

Reactions carried out using 0.20 mmol of *N*-oxide **2a** and 0.50 mmol of acetylating agent. A solution of **2a** in CD_2Cl_2 was cooled to 0 °C before the dropwise addition of acetylating agent. [a]For full references of pK_aH values see [29]. [b]Assay yields of **3a/4a** were determined by ^1H NMR using 1,1,2,2-tetrachloroethane as an internal standard.

Table S3: Further survey of acetylating agents for selective *endo*-cyclic iminium ion formation following adapted procedure by Volz and co-workers.[a]

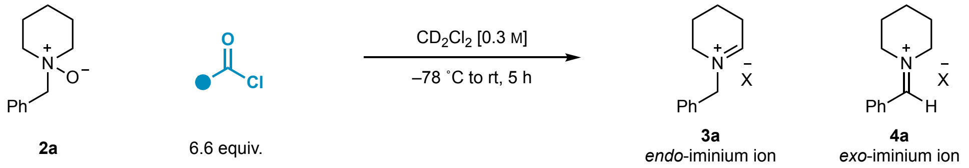

Entry	Acetylating agent	pK_aH of conjugate anion[b]	Combined assay yield / %[c]	*Endo* : *Exo* selectivity
**1**	Trifluoroacetic anhydride	0.52	100	4.8 : 1
**2**	Acetyl chloride	–7.00	89	9.0 : 1
**3**	Acetyl bromide	–9.00	93	15.7 : 1

Reactions carried out by using 0.20 mmol of *N*-oxide **2a** and 1.32 mmol of acetylating agent. A solution of **2a** in CD_2Cl_2 was cooled to –78 °C. Simultaneously, a solution of acetylating agent (6.6 equiv) in CD_2Cl was cooled to –78 °C to which the solution of *N*-oxide **2a** in CD_2Cl_2 was added to dropwise. [a]See reference 89 in manuscript. [b]For full references of pK_aH values see [29]. [c]Assay yields of **3a/4a** were determined by ^{1}H NMR using 1,1,2,2-tetrachloroethane as an internal standard.

Table S4: Further survey of acetylating agents for selective *endo*-cyclic iminium ion formation

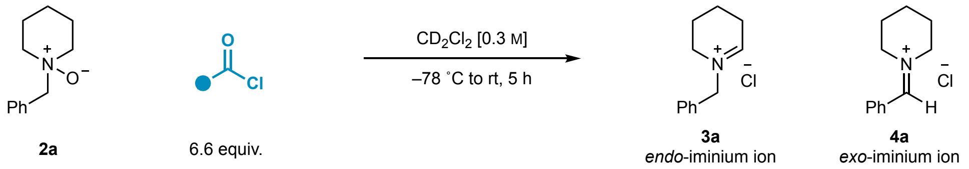

Entry	Acetylating agent	Combined assay yield / %[a]	*Endo* : *Exo* selectivity
**1**	Acetyl chloride	89	9.0 : 1
**2**	Isobutyryl chloride	85	13.2 : 1
**3**	Pivaloyl chloride (PivCl)	90	>20 : 1
**4**	Benzoyl chloride	75	>20 : 1

Reactions carried out following general procedure 1 using 0.20 mmol of *N*-oxide **2a** and 1.32 mmol of acetylating agent. A solution of acetylating agent in CD_2Cl_2 was cooled to –78 °C before the dropwise addition of a solution of *N*-oxide **2a** in CD_2Cl_2. [a]Assay yields of **3a/4a** were determined by ^{1}H NMR using 1,1,2,2-tetrachloroethane as an internal standard.

3.2 Zn mediated α-alkylation

Table S5: Initial results for sequential iminium ion formation and Zn mediated α-alkylation

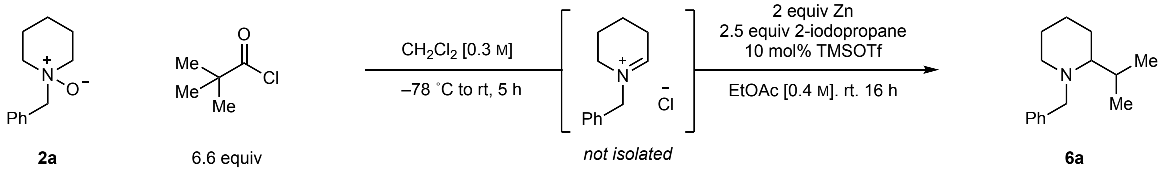

Entry	PivCl equiv	2-iodopropane equiv	TBSOTf mol%	Zn equiv	Assay yield / %[a]
**1**	6.6	2.5	10	2	0

Reaction carried out following general procedure 2 using 0.20 mmol of *N*-oxide **2a** and 1.32 mmol of acetylating agent. [a]Assay yields of **6a** were determined by ^{1}H NMR using 1,1,2,2-tetrachloroethane as an internal standard.

Table S6: Zn CAA control reactions in the presence of pivaloyl chloride under standard conditions

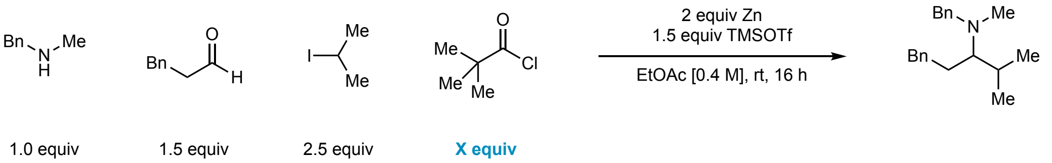

Entry	PivCl equiv	Assay yield / %[a]
**1**	0.0	84
**2**	1.0	27
**3**	1.5	21
**3**	5.6	0

Reactions carried out using 0.20 mmol of amine, 0.30 mmol of aldehyde and 0.50 mmol of 2-iodopropane. [a]Assay yields were determined by ^{1}H NMR using 1,1,2,2-tetrachloroethane as an internal standard.

Table S7: Survey of acetylating agent equivalence against yield and selectivity of *endo*-cyclic iminium ion formation

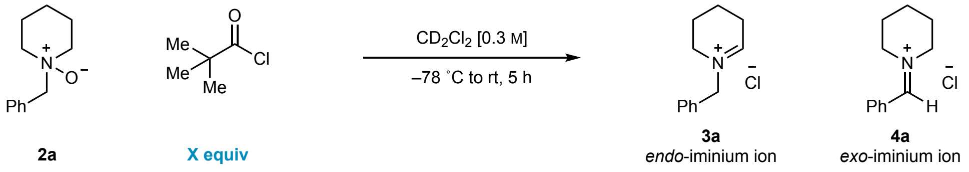

Entry	PivCl equiv	Combined assay yield / %[a]	*Endo* : *Exo* selectivity
**1**	6.6	89	>20 : 1
**2**	4.0	100	>20 : 1
**3**	3.0	94	>20 : 1
**4**	2.5	96	>20 : 1
**5**	2.0	87	>20 : 1
**6**	1.2	75	>20 : 1

Reactions carried out following general procedure 1 using 0.20 mmol of *N*-oxide **2a**. [a]Assay yields of **3a/4a** were determined by ^{1}H NMR using 1,1,2,2-tetrachloroethane as an internal standard.

Table S8: Initial optimisation for Zn mediated α-alkylation reaction

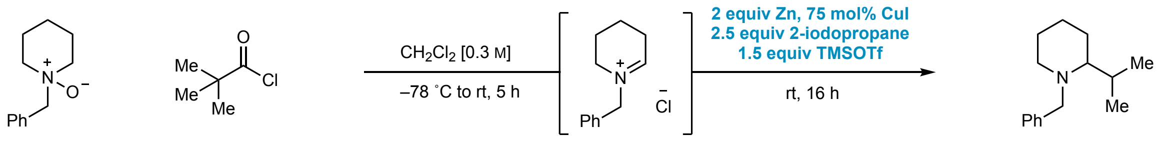

Entry	PivCl equiv	Order of addition	Assay yield / %[a]
**1**	2.5	Zn then alkyl iodide then TMSOTf	3
**2**	2.5	Zn then TMSOTf then alkyl iodide	37
**3**	2.5	Alkyl iodide then Zn then TMSOTf	14
**4**	2.5	Alkyl iodide then TMSOTf then Zn	46
**5**	2.5	TMSOTf then Zn then alkyl iodide	37
**6**	2.5	TMSOTf then alkyl iodide then Zn	48
**7**	2.5	CuI then TMSOTf then alkyl iodide then Zn	33
**8**	2.5	TMSOTf then CuI then alkyl iodide then Zn	57
**9**	2.5	TMSOTf then alkyl iodide then CuI then Zn	35
**10**	2.5	TMSOTf then alkyl iodide then Zn then CuI	45

Reactions carried out following general procedure 2 using 0.20 mmol of *N*-oxide **2a** and 0.50 mmol of PivCl. [a]Assay yields of **6a** were determined by ^1H NMR using 1,1,2,2-tetrachloroethane as an internal standard.

Table S9: Further optimisation of the Zn mediated α-alkylation reaction

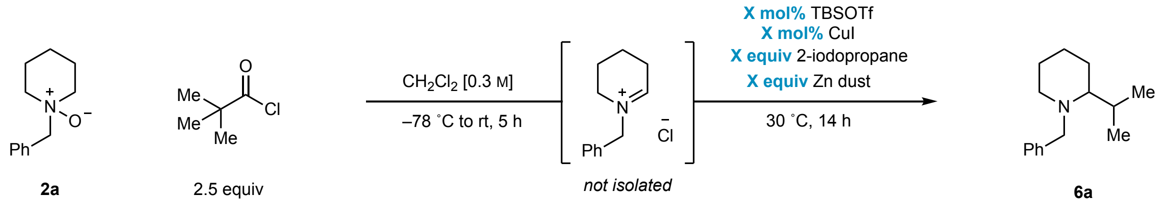

Entry	Zn equiv	2-iodopropane equiv	Time / h	CuI mol%	Lewis base	Assay yield / %[a]
**1**	1.0	2.0	12	40	TMSOTf (150 mol%)	8
**2**	1.5	2.0	12	40	TMSOTf (150 mol%)	38
**3**	2.0	2.0	12	40	TMSOTf (150 mol%)	29
**4**	3.0	2.0	12	40	TMSOTf (150 mol%)	12
**5**	1.5	1.0	12	75	TMSOTf (150 mol%)	40
**6**	1.5	2.0	12	75	TMSOTf (150 mol%)	49
**7**	1.5	2.5	12	75	TMSOTf (150 mol%)	55
**8**	1.5	3.0	12	75	TMSOTf (150 mol%)	52
**9**	1.5	2.5	2	75	TMSOTf (150 mol%)	44
**10**	1.5	2.5	3	75	TMSOTf (150 mol%)	55
**11**	1.5	2.5	14	75	TMSOTf (150 mol%)	59
**12**	1.5	2.5	16	75	TMSOTf (150 mol%)	46
**13**	1.5	2.5	20	75	TMSOTf (150 mol%)	48
**14**	1.5	2.5	14	10	TMSOTf (150 mol%)	57
**15**	1.5	2.5	14	25	TMSOTf (150 mol%)	66
**16**	1.5	2.5	14	50	TMSOTf (150 mol%)	42
**17**	1.5	2.5	14	25	TMSOTf (25 mol%)	55
**18**	1.5	2.5	14	25	TMSOTf (50 mol%)	63
**19**	1.5	2.5	14	25	TMSOTf (75 mol%)	70
**20**	1.5	2.5	14	25	TMSOTf (100 mol%)	59
**21**	1.5	2.5	14	25	TBSOTf (75 mol%)	100
**22**	1.5	2.5	14	25	TMSCl (75 mol%)	51
**23**	1.5	2.5	14	25	TMSBr (75 mol%)	58

Reactions carried out following general procedure 2 using 0.20 mmol of *N*-oxide **2a** and 0.50 mmol of PivCl [a]Assay yields of **6a** were determined by ^{1}H NMR using 1,1,2,2-tetrachloroethane as an internal standard.

Table S10: Preliminary scope of Zn mediated α-alkylation with respect to the alkyl iodide

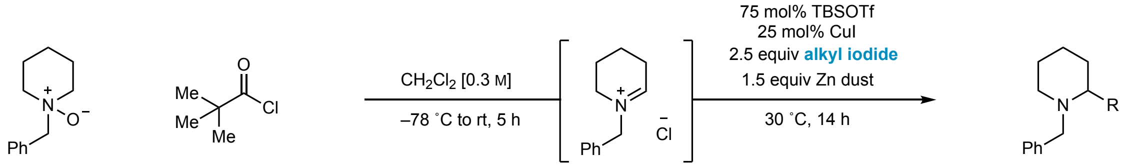

2a 2.5 equiv *not isolated* **6**

Entry	Alkyl iodide	Assay yield / %[a]
**1**	2-iodopropane	100
**2**	1-iodopropane	15
**3**	2-iodo-2-methylpropane	35

Reactions carried out following general procedure 2 using 0.20 mmol of *N*-oxide **2a** and 0.50 mmol of PivCl. Order of addition was as follows: TBSOTf then CuI then alkyl iodide then Zn dust. [a]Assay yields of **6** were determined by ^1H NMR using 1,1,2,2-tetrachloroethane as an internal standard.

Table S11: Optimisation for Zn mediated α-alkylation with primary alkyl iodides

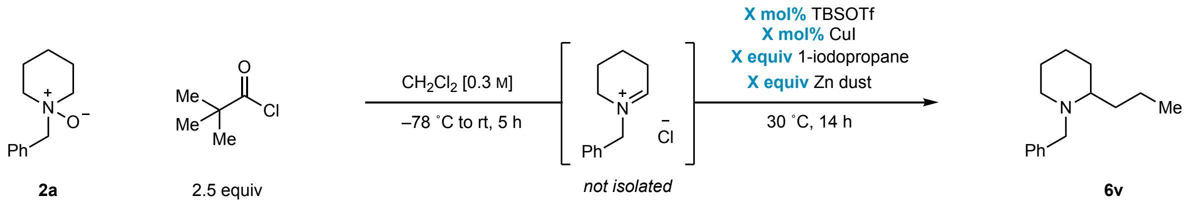

Entry	CuI mol%	TBSOTf mol%	1-iodopropane equiv	Zn equiv	Assay yield / %[a]
**1**	25	75	2.5	1.5	15
**2**	40	75	2.5	1.5	24
**3**	50	75	2.5	1.5	24
**4**	40	25	2.5	1.5	35
**5**	40	50	2.5	1.5	44
**5**	40	75	2.5	1.5	45
**6**	40	100	2.5	1.5	41
**7**	40	75	1.5	1.5	40
**8**	40	75	2.0	1.5	46
**9**	40	75	2.5	1.5	52
**10**	40	75	3.0	1.5	40
**11**	40	75	2.5	2.0	33
**12**	40	75	2.5	2.5	60
**13**	40	75	2.5	3.0	59
**14**	40	75	2.5	5.0	45

Reactions carried out following general procedure 2 using 0.20 mmol of *N*-oxide **2a** and 0.50 mmol of PivCl. [a]Assay yields of **6v** were determined by ^{1}H NMR using 1,1,2,2-tetrachloroethane as an internal standard.

Table S12: Optimisation for Zn mediated α-alkylation with tertiary alkyl iodides

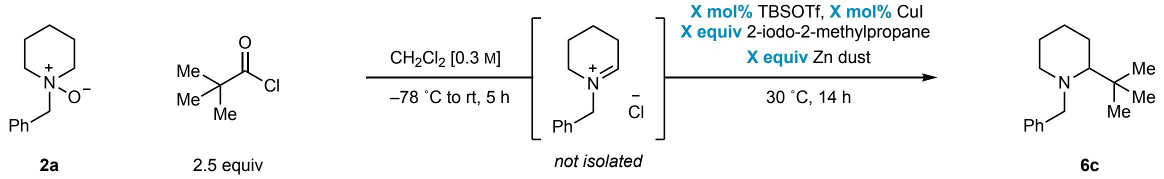

Entry	CuI mol%	Zn equiv	2-iodopropane equiv	TBSOTf mol%	Assay yield / %[a]
**1**	25	1.5	2.5	75	35
**2**	40	1.5	2.5	75	30
**3**	50	1.5	2.5	75	42
**4**	75	1.5	2.5	75	33
**5**	50	2.0	2.5	75	37
**6**	50	2.5	2.5	75	63
**7**	50	3.0	2.5	75	65
**8**	50	5.0	2.5	75	61
**9**	50	3.0	1.5	75	52
**10**	50	3.0	2.0	75	58
**11**	50	3.0	3.0	75	83
**12**	50	3.0	4.0	75	67
**13**	50	3.0	3.0	25	88
**14**	50	3.0	3.0	50	96
**15**	50	5.0	3.0	100	79

Reactions carried out following general procedure 2 using 0.20 mmol of *N*-oxide **2a** and 0.50 mmol of PivCl. [a]Assay yields of **6c** were determined by ^{1}H NMR using 1,1,2,2-tetrachloroethane as an internal standard.

During investigations into the alkyl iodide scope, *N*-benzyl-2-(*tert*-butyl)piperidine (**6c**) was identified and isolated as a side-product of this reaction. Although only minor formation of XX was observed across all scope entries, its separation from closely related alpha-alkylated products proved challenging, with the best isolated yield of **6a** still containing 5% of this impurity. We speculated that the formation of **6c** was the result of fragmentation of residual PivCl during the alkylation step, as **6c** was not observed during the preceding elimination step. Barteau, Ryzhov and Buijs have independently demonstrated the ability of Cu and Zn complexes to promote decarboxylation of aliphatic carboxylic acids, a by-product of the Polonovski-Potier reaction.[30-32] Various approaches were subsequently explored to achieve the removal of PivCl from the reaction prior to the key alpha-functionalisation reaction, including the use of chemical scavengers and iminium ion precipitation with metal perchlorate salts. During the course of these studies, control reactions were undertaken to assess the feasibility of performing the alkylation step in the presence of metal perchlorate salts. To our delight, the perchlorate anion appeared to suppress the formation of **6c**, while $CuClO_4$ simultaneously maintained high yields of product formation. We reasoned that the more weakly coordinating ClO_4 anion increased the electrophilicity, and thus reactivity, of the iminium ion intermediate, thereby promoting reactivity with the alkyl zinc species over the slower forming *tert*-butyl fragment.[33]

Table S13: Optimisation of an alternative Zn mediated α-alkylation procedure for tertiary alkyl iodides

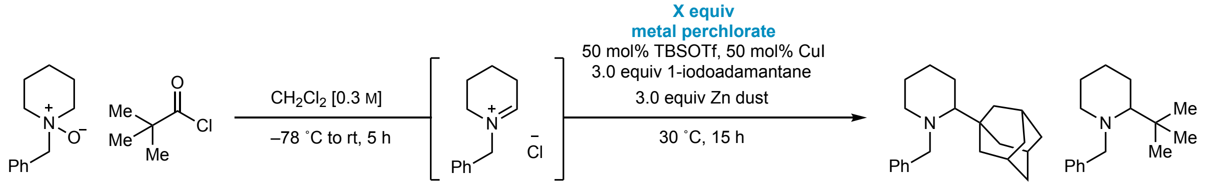

not isolated

2a 2.5 equiv **6d** **6c**

Entry	Metal perchlorate	Equiv	6d / %[a]	6c / %[a]
**1**	$Mg(ClO_4)_2$	1.0	7	<5
**2**	$NaClO_4$	1.0	28	<5
**3**	$Cu(ClO_4)_2$	1.0	66	<5
**4**	$Zn(ClO_4)_2$	1.0	4	<5
**5**	$AgClO_4$	1.0	7	<5
**6**	$Cu(ClO_4)_2$	0.5	67	<5
**7**	$Cu(ClO_4)_2$	1.5	82	<5
**8**	$Cu(ClO_4)_2$	2.0	59	<5

Reactions carried out following general procedure 3 using 0.20 mmol of *N*-oxide **2a** and 0.50 mmol of PivCl. [a]Assay yields of **6c/6d** were determined by ^{1}H NMR using 1,1,2,2-tetrachloroethane as an internal standard.

Table S14: Further optimisation of an alternative Zn mediated α-alkylation procedure for secondary alkyl iodides

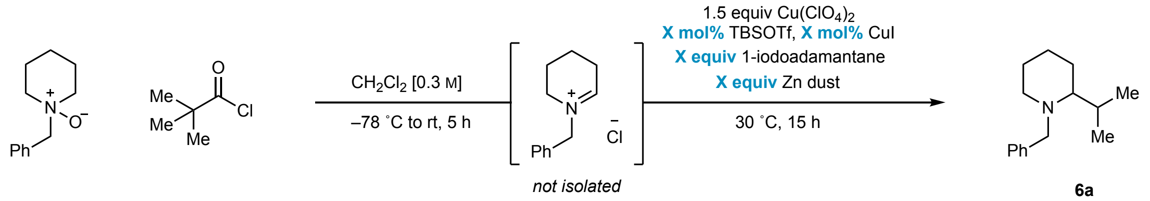

Entry	2-iodopropane equiv	TBSOTf mol%	CuI mol%	Zn equiv	Assay yield / %[a]
**1**	2.0	50	50	3.0	63
**2**	2.5	50	50	3.0	61
**3**	3.0	50	50	3.0	70
**4**	5.0	50	50	3.0	59
**5**	3.0	25	50	3.0	78
**6**	3.0	75	50	3.0	71
**7**	3.0	100	50	3.0	69
**8**	3.0	25	0	3.0	64
**9**	3.0	25	25	3.0	66
**10**	3.0	25	75	3.0	76
**11**	3.0	25	100	3.0	74
**12**	3.0	25	50	1.5	26
**13**	3.0	25	50	2.0	83
**14**	3.0	25	50	2.5	73
**15**	3.0	25	50	5.0	72

Reactions carried out using 0.20 mmol of *N*-oxide **2a** and 0.50 mmol of PivCl. [a]Assay yields of **6d** were determined by ^{1}H NMR using 1,1,2,2-tetrachloroethane as an internal standard.

3.3 Grignard mediated α-alkylation

Table S15: Initial results and optimisation for Grignard mediated α-alkylation

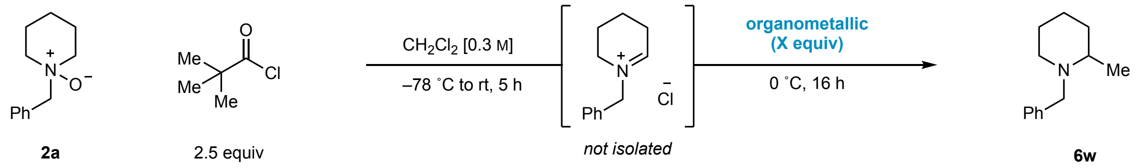

Entry	Organometallic	Assay yield / %[a]
**1**	Methyl magnesium bromide (2.0 equiv)	38
**2**	Methyl magnesium bromide (3.0 equiv)	44
**3**	Methyl magnesium bromide (4.0 equiv)	42
**4**	Methyl magnesium bromide (5.0 equiv)	42
**5**	Methyl lithium (3.0 equiv)	3

Reactions carried out following general procedure 4 using 0.20 mmol of *N*-oxide **2a** and 0.50 mmol of PivCl. [a]Assay yields of **6w** were determined by ^{1}H NMR using 1,1,2,2-tetrachloroethane as an internal standard.

3.4 α-Trifluoromethylation

Table S16: Initial results and optimisation for α-trifluoromethylation

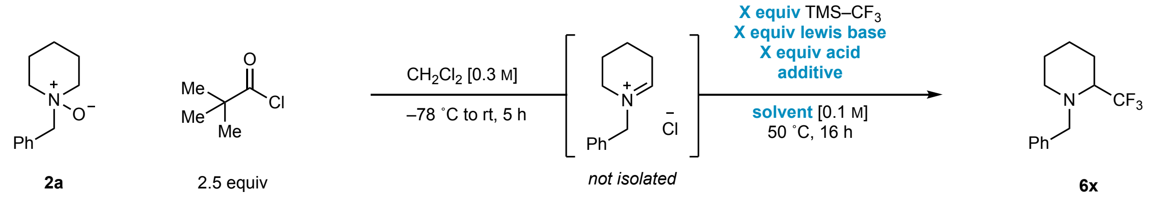

Entry	TMS–CF_3 equiv	Lewis base	Acid	Solvent	Additive (3 equiv)	Assay yield / %[a]
**1**	2.0	KHF_2 (2.0 equiv)	TFA (2.0 equiv)	MeCN	-	14
**2**	2.0	KHF_2 (2.0 equiv)	TFA (2.0 equiv)	DMF	-	0
**3**	2.0	KHF_2 (2.0 equiv)	TFA (2.0 equiv)	MeCN	DMF	30
**4**	2.0	KHF_2 (2.0 equiv)	TfOH (2.0 equiv)	MeCN	DMF	0
**5**	2.0	KF (2.0 equiv)	TFA (2.0 equiv)	MeCN	DMF	14
**6**	2.0	NaOAc (2.0 equiv)	TFA (2.0 equiv)	MeCN	DMF	0
**7**	3.0	KHF_2 (3.0 equiv)	TFA (3.0 equiv)	MeCN	DMF	37
**8**	4.0	KHF_2 (4.0 equiv)	TFA (4.0 equiv)	MeCN	DMF	52
**9**	5.0	KHF_2 (5.0 equiv)	TFA (5.0 equiv)	MeCN	DMF	46

Reactions carried out following general procedure 5 using 0.20 mmol of *N*-oxide **2a** and 0.50 mmol of PivCl. [a]Assay yields of **6x** were determined by ^1H NMR using 1,1,2,2-tetrachloroethane as an internal standard.

3.5 α-Azinylation

Table S17: Initial results and optimisation for one pot α-heteroarylation

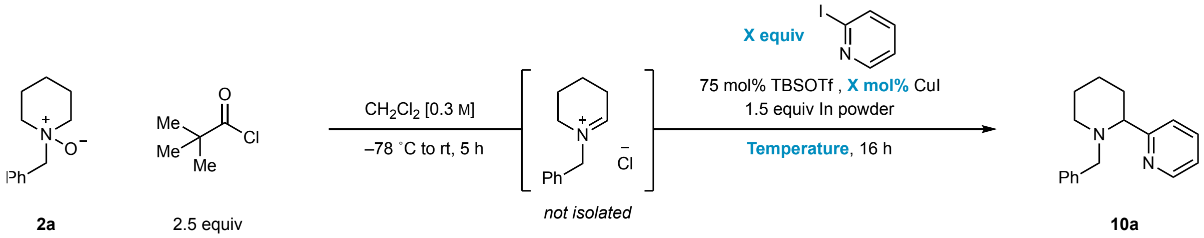

Entry	2-iodopyridine equiv	Temperature / °C	CuI / mol%	Assay yield / %[a]
1[b]	2.0	70	-	20
2[b]	3.0	70	-	21
3	2.0	15	-	32
4	2.0	25	-	28
5	2.0	40	-	44
6	2.0	50	-	22
7	2.0	60	-	25
8	2.0	25	25	26
9	2.0	25	50	45
10	2.0	25	75	24
11	2.0	25	100	18

Reactions carried out following general procedure 6 using 0.20 mmol of *N*-oxide **2a** and 0.50 mmol of PivCl. [a]Assay yields of **10a** were determined by ^{1}H NMR using 1,1,2,2-tetrachloroethane as an internal standard. [b]Reaction run with 1.5 equiv TMSOTf.

Table S18: Further optimisation of the one pot α-heteroarylation reaction

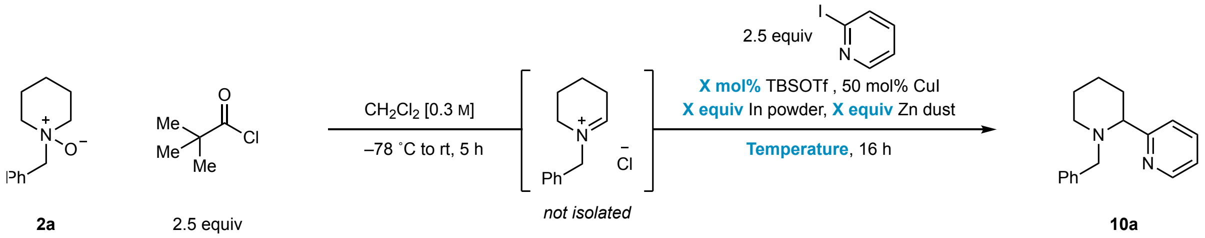

Entry	In equiv	Zn equiv	Temperature / °C	TBSOTf / mol%	Assay yield / %[a]
**1**	1.5	-	25	75	32
**2**	-	1.5	25	75	42
**3**	1.5	1.5	25	75	61
**4**	1.5	1.5	40	75	71
**5**	3.0	-	40	75	55
**6**	3.0	1.0	40	75	62
**7**	3.0	1.5	40	75	76
**8**	3.0	1.5	40	50	55

Reactions carried out following general procedure 6 using 0.20 mmol of *N*-oxide **2a** and 0.50 mmol of PivCl. [a]Assay yields of **10a** were determined by ^{1}H NMR using 1,1,2,2-tetrachloroethane as an internal standard.

4. Iminium ion scope

*1-benzyl-2,3,4,5-tetrahydropyridin-1-ium chloride (**3a**):*

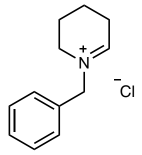

Prepared according to general procedure **1** using 1-benzyl piperidine *N*-oxide (38.2 mg, 0.20 mmol, 1.0 equiv) and pivaloyl chloride (163 μL, 1.32 mmol, 6.6 equiv). After warming to rt, 1,1,2,2-tetrachloroethane (21 μL, 0.20 mmol, 1.0 equiv) was added as an internal standard and the yield was determined *via* quantitative ^{1}H NMR spectroscopy (100%, >20:1 r.r.).

^{1}H NMR (400 MHz, CD_2Cl_2): δ 9.29 (s, 1H), 7.57 – 7.49 (m, 2H), 7.48 – 7.38 (m, 3H), 5.23 (s, 2H), 3.74 – 3.67 (m, 2H), 3.09 – 3.04 (m, 2H), 2.01 – 1.88 (m, 2H), 1.89 – 1.72 (m, 2H).

^{13}C{H} NMR (101 MHz, CD_2Cl_2): δ 180.3, 130.7, 130.3 (2C), 130.2, 129.8 (2C), 65.8, 50.6, 29.4, 20.9, 15.8.

HRMS & IR: *compound unstable.*

*Scale up: 1-benzyl-2,3,4,5-tetrahydropyridin-1-ium chloride (**3a**):*

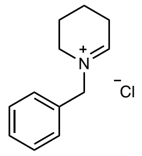

Prepared according to general procedure **1.** A solution of pivaloyl chloride (0.81 mL, 6.6 mmol, 6.6 equiv) in anhydrous CD_2Cl_2 (1.0 mL, 1.0 M) was cooled to –78 °C. To this was added dropwise a solution of 1-benzyl piperidine *N*-oxide (191 mg, 1.0 mmol, 1.0 equiv) in anhydrous CD_2Cl_2 (1.0 mL, 1.0 M), which was also cooled to –78 ˚C. The vial containing the *N*-oxide was washed with CD_2Cl_2 (1.0 mL), cooled to –78 ˚C and added dropwise to the pivaloyl chloride solution. After complete addition, the mixture was stirred for a further 15 mins at –78 ˚C before warming slowly to rt over 5 h. After warming to rt, 1,1,2,2-tetrachloroethane (105 μL, 1.0 mmol, 1.0 equiv) was added as an internal standard and the yield was determined *via* quantitative ^{1}H NMR spectroscopy (80%, >20:1 r.r.).

^{1}H NMR (400 MHz, CD_2Cl_2) δ 9.28 (s, 1H), 7.57 – 7.50 (m, 2H), 7.45 – 7.41 (m, 3H), 5.24 (s, 2H), 3.74 – 3.66 (m, 2H), 3.09 – 3.00 (m, 2H), 2.02 – 1.90 (m, 2H), 1.84 – 1.78 (m, 2H).

^{13}C{H} NMR (101 MHz, CD_2Cl_2) 180.2, 130.6, 130.5 (2C), 130.4, 129.9 (2C), 66.0, 50.8, 29.5, 21.0, 15.9.

HRMS & IR: *compound unstable.*

*1-(3-phenylpropyl)-2,3,4,5-tetrahydropyridin-1-ium chloride (**3b**):*

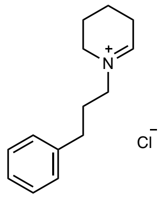

Prepared according to general procedure **1** using 1-(3-phenylpropyl)piperidine *N*-oxide (43.9 mg, 0.20 mmol, 1.0 equiv) and pivaloyl chloride (163 µL, 1.32 mmol, 6.6 equiv). After warming to rt, 1,1,2,2-tetrachloroethane (21 µL, 0.20 mmol, 1.0 equiv) was added as an internal standard and the yield was determined *via* quantitative ^{1}H NMR spectroscopy (100%, >20:1 r.r.).

^{1}H NMR (400 MHz, CD_2Cl_2) δ 9.17 (s, 1H), 7.34 – 7.14 (m, 5H), 4.04 (t, *J* = 7.5 Hz, 2H), 3.68 – 3.61 (m, 2H), 2.95 – 2.88 (m, 2H), 2.76 (t, *J* = 7.5 Hz, 2H), 2.19 (p, *J* = 7.5 Hz, 2H), 1.93 – 1.86 (m, 2H), 1.73 – 1.67 (m, 2H).

^{13}C{H} NMR (101 MHz, CD_2Cl_2) δ 180.4, 140.5, 129.0 (2C), 128.8 (2C), 126.8, 62.3, 50.8, 32.8, 29.2, 28.3, 20.8, 15.6.

HRMS & IR: *compound unstable.*

*1-methyl-2,3,4,5-tetrahydropyridin-1-ium chloride (3**c**):*

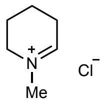

Prepared according to general procedure **1** using 1-methylpiperidine *N*-oxide (23.0 mg, 0.20 mmol, 1.0 equiv) and pivaloyl chloride (163 µL, 1.32 mmol, 6.6 equiv). After warming to rt, 1,1,2,2-tetrachloroethane (21 µL, 0.20 mmol, 1.0 equiv) was added as an internal standard and the yield was determined *via* quantitative ^{1}H NMR spectroscopy (87%, >20:1 r.r.).

^{1}H NMR (400 MHz, CD_2Cl_2) δ 9.08 (s, 1H), 3.75 (s in m, 5H), 2.96 (tt, *J* = 4.3, 2.1 Hz, 2H), 2.01 (td, *J* = 7.5, 4.7 Hz, 2H), 1.88 – 1.74 (m, 2H).

^{13}C{H} NMR (101 MHz, CD_2Cl_2) δ 180.3, 52.9, 49.5, 29.1, 20.8, 15.4.

HRMS & IR: *compound unstable.*

*1-cyclohexyl-2,3,4,5-tetrahydropyridin-1-ium chloride (**3d**):*

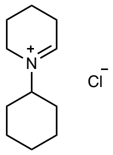

Prepared according to general procedure **1** using 1-cyclohexylpiperidine *N*-oxide (36.6 mg, 0.20 mmol, 1.0 equiv) and pivaloyl chloride (163 µL, 1.32 mmol, 6.6 equiv). After warming to rt, 1,1,2,2-tetrachloroethane (21 µL, 0.20 mmol, 1.0 equiv) was added as an internal standard and the yield was determined *via* quantitative ^{1}H NMR spectroscopy (100%, >20:1 r.r.).

^{1}H NMR (700 MHz, CD_2Cl_2) δ 9.16 (s, 1H), 3.99 (tt, *J* = 12.1, 3.6 Hz, 1H), 3.75 – 3.70 (m, 2H), 3.08 – 3.03 (m, 2H), 2.14 (br d, *J* = 9.7 Hz, 2H), 2.03 – 1.97 (m, 2H), 1.91 (dt, *J* = 11.7, 2.5 Hz, 2H), 1.87 – 1.81 (m, 2H), 1.72 – 1.61 (m, 3H), 1.46 – 1.37 (m, 2H), 1.22 – 1.19 (m, 2H).

^{13}C{H} NMR (176 MHz, CD_2Cl_2) δ 178.7, 72.0, 48.7, 30.6 (2C), 29.4, 25.2 (2C), 25.1, 21.2, 16.3.

HRMS & IR: *compound unstable.*

*1-phenyl-2,3,4,5-tetrahydropyridin-1-ium chloride (**3e**):*

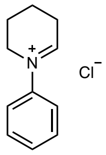

Prepared according to general procedure **1** using 1-phenylpiperidine *N*-oxide (35.5 mg, 0.20 mmol, 1.0 equiv) and pivaloyl chloride (163 µL, 1.32 mmol, 6.6 equiv). After warming to rt, 1,1,2,2-tetrachloroethane (21 µL, 0.20 mmol, 1.0 equiv) was added as an internal standard and the yield was determined *via* quantitative ^{1}H NMR spectroscopy (65%, >20:1 r.r.).

^{1}H NMR (700 MHz, CD_2Cl_2) δ 9.12 (s, 1H), 7.73 – 7.65 (m, 2H), 7.52 – 7.49 (m, 2H), 7.44 – 7.37 (m, 1H), 4.25 (br s, 2H), 3.25 (br s, 2H), 2.26 – 2.16 (m, 2H), 2.06 – 1.97 (m, 2H).

^{13}C{H} NMR (176 MHz, CD_2Cl_2) δ 169.9, 143.3, 132.1 (2C), 131.2, 123.0 (2C), 53.6, 53.0, 25.1, 20.7.

HRMS & IR: *compound unstable.*

*1-benzyl-2-methyl-2,3,4,5-tetrahydropyridin-1-ium chloride (**3f**) and 1-benzyl-6-methyl-2,3,4,5-tetrahydropyridin-1-ium chloride (**3f'**):*

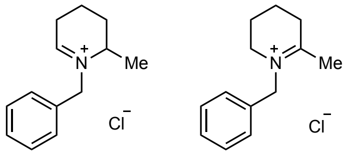

Prepared according to general procedure **1** using 1-benzyl-2-methylpiperidine *N*-oxide (41.1 mg, 0.20 mmol, 1.0 equiv) and pivaloyl chloride (163 µL, 1.32 mmol, 6.6 equiv). After warming to rt, 1,1,2,2-tetrachloroethane (21 µL, 0.20 mmol, 1.0 equiv) was added as an internal standard and the yield was determined *via* quantitative ^{1}H NMR spectroscopy (total 84%, 1:6.1 r.r.)

Major regioisomer (74%).

*1-benzyl-6-methyl-2,3,4,5-tetrahydropyridin-1-ium chloride (**3f'**):*

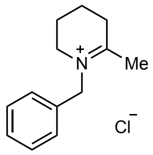

^{1}H NMR (400 MHz, CD_2Cl_2) δ 7.46 – 7.34 (m, 5H), 5.18 (s, 2H), 3.85 – 3.73 (m, 2H), 3.09 (t, *J* = 6.3 Hz, 2H), 2.68 (s, 3H), 2.01 – 1.82 (m, 4H).

^{13}C{H} NMR (101 MHz, CD_2Cl_2) δ 190.4, 131.4 129.8 (2C), 129.5, 2C), 128.3 (2C), 60.4, 53.6, 35.9, 25.2, 21.2, 17.5.

HRMS & IR: *compound unstable.*

Minor regioisomer (13%).

*1-benzyl-2-methyl-2,3,4,5-tetrahydropyridin-1-ium chloride (**3f**):*

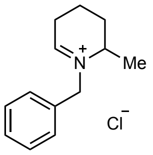

^{1}H NMR (400 MHz, CD_2Cl_2) δ 9.39 (s, 1H), 7.49 – 7.24 (m, 5H), 5.46 (d, *J* = 14.3 Hz, 1H, H_6), 5.04 (d, *J* = 14.4 Hz, 1H), 3.94 (t, *J* = 5.8 Hz, 1H), 3.08 (d, *J* = 6.1 Hz, 2H), 2.03 – 1.78 (m, 2H), 1.47 (d, *J* = 6.7 Hz, 4H).

^{13}C{H} NMR (101 MHz, CD_2Cl_2) δ 181.2, 131.1, 130.8, 129.8 (2C), 129.4 (2C), 63.1, 55.6, 29.5, 18.4, 12.3.

HRMS & IR: *compound unstable*

*1-benzyl-5-methyl-2,3,4,5-tetrahydropyridin-1-ium chloride (**3g**) and 1-benzyl-3-methyl-2,3,4,5-tetrahydropyridin-1-ium chloride (**3g'**):*

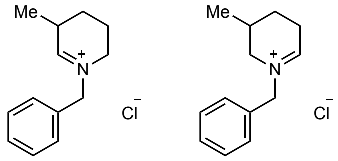

Prepared according to general procedure **1** using 1-benzyl-3-methylpiperidine *N*-oxide (41.0 mg, 0.20 mmol, 1.0 equiv) and pivaloyl chloride (163 μL, 1.32 mmol, 6.6 equiv). After warming to rt, 1,1,2,2-tetrachloroethane (21 μL, 0.20 mmol, 1.0 equiv) was added as an internal standard and the yield was determined *via* quantitative ^{1}H NMR spectroscopy (73%, 1:1.3 r.r.).

<u>*Major regioisomer (41%).*</u>

*1-benzyl-3-methyl-2,3,4,5-tetrahydropyridin-1-ium chloride (**3g'**):*

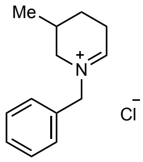

^{1}H NMR (700 MHz, CD_2Cl_2) δ 9.45 – 9.36 (m, 1H), 7.53 – 7.51 (m, 2H), 7.43 – 7.42 (m, 3H), 5.24 (s, 2H), 3.68 (d, *J* = 4.9 Hz, 1H), 3.25 – 3.14 (m, 2H), 3.09 – 2.92 (m, 1H), 2.08 (s, 1H), 1.91 – 1.83 (m, 1H), 1.47 – 1.41 (m, 1H), 0.98 (d, *J* = 6.7 Hz, 3H).

^{13}C{H} NMR (176 MHz, CD_2Cl_2) δ 183.1, 130.8, 130.2, 129.9 (2C), 129.8 (2C), 65.7, 56.2, 29.6, 27.1, 23.8, 18.1.

HRMS & IR: *compound unstable*

Minor regioisomer (32%).

*1-benzyl-5-methyl-2,3,4,5-tetrahydropyridin-1-ium chloride (**3g**)*

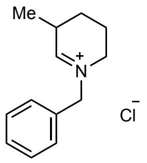

^{1}H NMR (700 MHz, CD_2Cl_2) δ 9.31 (s, 1H), 7.53 – 7.51 (m, 3H), 7.43 (d, *J* = 0.8 Hz, 3H), 5.30 (d, *J* = 14.1 Hz, 1H), 5.22 (s, 1H), 3.69 – 3.65 (m, 2H), 3.13 (d, *J* = 6.0 Hz, 1H), 1.99 – 1.95 (m, 2H), 1.52 – 1.41 (m, 2H), 1.39 (d, *J* = 7.6 Hz, 3H).

^{13}C{H} NMR (176 MHz, CD_2Cl_2) δ 180.2, 130.9, 130.3 (2C), 129.8 (2C), 129.0, 65.7, 50.7, 34.5, 24.0, 23.8, 17.1.

HRMS & IR: *compound unstable*

1-benzyl-4-methyl-2,3,4,5-tetrahydropyridin-1-ium chloride (**3h**):

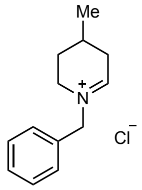

Prepared according to general procedure **1** using 1-benzyl-4-methylpiperidine *N*-oxide (41.0 mg, 0.20 mmol, 1.0 equiv) and pivaloyl chloride (163 μL, 1.32 mmol, 6.6 equiv). After warming to rt, 1,1,2,2-tetrachloroethane (21 μL, 0.20 mmol, 1.0 equiv) was added as an internal standard and the yield was determined *via* quantitative ^{1}H NMR spectroscopy (66%, >20:1 r.r.).

^{1}H NMR (700 MHz, CD_2Cl_2) δ 9.27 (d, *J* = 3.5 Hz, 1H), 7.55 – 7.52 (m, 2H), 7.45 – 7.42 (m, 3H), 5.28 (d, *J* = 14.1 Hz, 1H), 5.20 (d, *J* = 14.1 Hz, 1H), 3.80 – 3.69 (m, 2H), 3.21 (d, *J* = 21.7 Hz, 1H), 2.57 (dd, *J* = 22.0, 9.0 Hz, 1H), 2.05 – 1.95 (m, 2H), 1.68 – 1.58 (m, 1H), 1.05 (d, *J* = 6.6 Hz, 3H).

^{13}C{H} NMR (176 MHz, CD_2Cl_2) δ 179.9, 130.8, 130.4 (2C), 130.3, 129.9 (2C), 65.6, 50.8, 37.0, 28.8, 22.9, 20.4.

HRMS & IR: *compound unstable*

*1-benzyl-3-4-dihydro-2H-pyrrol-1-ium chloride (**3k**) and 1-benzylidenepyrrolidin-1-ium chloride (**4k**):*

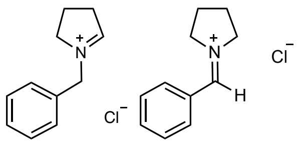

Prepared according to general procedure **1** using 1-benzylpyrrolidine *N*-oxide (35.5 mg, 0.20 mmol, 1.0 equiv) and pivaloyl chloride (163 μL, 1.32 mmol, 6.6 equiv). After warming to rt, 1,1,2,2-tetrachloroethane (21 μL, 0.20 mmol, 1.0 equiv) was added as an internal standard and the yield was determined *via* quantitative ^{1}H NMR spectroscopy (total 91%, 1.2:1 r.r.).

<u>*Major regioisomer (50%).*</u>

*1-benzyl-3-4-dihydro-2H-pyrrol-1-ium chloride (**3k**):*

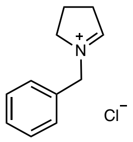

^{1}H NMR (700 MHz, CD_2Cl_2) δ 9.48 (s, 1H), 7.58 – 7.52 (m, 3H), 7.45 – 7.39 (m, 2H), 5.33 (s, 2H), 4.12 (t, *J* = 8.0 Hz, 2H), 3.37 (t, *J* = 7.9 Hz, 2H), 2.36 – 2.29 (m, 2H).

^{13}C{H} NMR (176 MHz, CD_2Cl_2) δ 182.1, 136.6, 130.1 (2C), 129.9, 129.3 (2C), 58.7, 58.4, 36.8, 20.0.

HRMS & IR: *compound unstable*

<u>*Minor regioisomer (41%).*</u>

*1-benzylidenepyrrolidin-1-ium chloride (**4k**):*

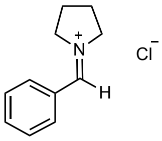

^{1}H NMR (700 MHz, CD_2Cl_2) δ 9.87 (s, 1H), 8.09 (d, *J* = 7.8 Hz, 2H), 7.77 (t, *J* = 7.5 Hz, 1H), 7.66 (t, *J* = 7.5 Hz, 2H), 4.61 (t, *J* = 7.1 Hz, 2H), 4.30 (t, *J* = 7.2 Hz, 2H), 2.36 – 2.32 (m, 2H), 2.24 – 2.19 (m, 2H).

^{13}C{H} NMR (176 MHz, CD_2Cl_2) δ 168.6, 136.3, 133.7 (2C), 129.8 (2C), 128.0, 60.3, 54.4, 25.7, 23.6.

HRMS & IR: *compound unstable.*

*1-benzyl-3,4,5,6-tetrahydrp-2H-azepin-1-ium chloride (**3l**) and 1-benzylideneazepan-1-ium chloride (**4l**):*

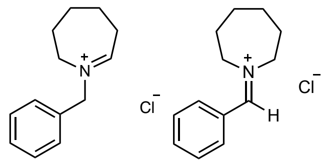

Prepared according to general procedure **1** using 1-benzylazepane *N*-oxide (41.1 mg, 0.20 mmol, 1.0 equiv) and pivaloyl chloride (163 μL, 1.32 mmol, 6.6 equiv). After warming to rt, 1,1,2,2-tetrachloroethane (21 μL, 0.20 mmol, 1.0 equiv) was added as an internal standard and the yield was determined *via* quantitative ^{1}H NMR spectroscopy (total 76%, 0.9:1 r.r.).

Major regioisomer (40%).

*1-benzylideneazepan-1-ium chloride (**4l**):*

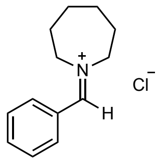

^{1}H NMR (400 MHz, CD_2Cl_2) δ 10.02 (s, 1H), 8.02 (d, *J* = 8.4 Hz, 2H), 7.74 (d, *J* = 7.9 Hz, 1H), 7.65 (d, *J* = 4.7 Hz, 2H), 4.61 – 4.43 (m, 2H), 4.41 – 4.21 (m, 2H), 2.10 (q, *J* = 6.2 Hz, 4H), 1.74 (dd, *J* = 5.9, 3.3 Hz, 4H).

^{13}C{H} NMR (101 MHz, CD_2Cl_2) δ 173.9, 134.8, 133.8 (2C), 130.3 (2C), 130.1, 62.9, 55.5, 27.4, 25.3.

HRMS & IR: *compound unstable.*

Minor regioisomer (36%).

*1-benzyl-3,4,5,6-tetrahydrp-2H-azepin-1-ium chloride (**3l**):*

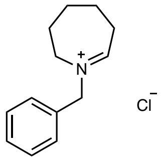

^{1}H NMR (700 MHz, CD_2Cl_2) δ 9.73 (t, *J* = 5.7 Hz, 1H), 7.56 – 7.54 (m, 3H), 7.45 – 7.42 (m, 2H), 5.32 (s, 2H), 3.97 – 3.90 (m, 2H), 3.11 – 2.97 (m, 2H), 1.92 – 1.86 (m, 2H), 1.86 – 1.80 (m, 2H), 1.56 – 1.48 (m, 2H).

^{13}C{H} NMR (101 MHz, CD_2Cl_2) δ 186.0, 136.1, 130.0 (2C), 129.4 (2C), 129.1, 67.4, 55.8, 31.8, 29.7, 24.7, 21.7.

HRMS & IR: *compound unstable.*

2-benzyl-3,4-dihydroisoqinolin-2-ium chloride (**3m**):

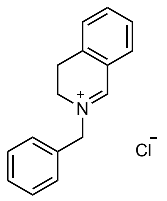

Prepared according to general procedure **1** using 2-benzyl-1,2,3,4-tetrahydroisoquinoline *N*-oxide (47.9 mg, 0.20 mmol, 1.0 equiv) and pivaloyl chloride (163 μL, 1.32 mmol, 6.6 equiv). After warming to rt, 1,1,2,2-tetrachloroethane (21 μL, 0.20 mmol, 1.0 equiv) was added as an internal standard and the yield was determined *via* quantitative ^{1}H NMR spectroscopy (100%, >20:1 r.r.).

^{1}H NMR (500 MHz, CD_2Cl_2) δ 10.26 (s, 1H), 8.04 (dd, *J* = 7.7, 1.3 Hz, 1H), 7.71 (td, *J* = 7.6, 1.4 Hz, 1H), 7.63 – 7.56 (m, 2H), 7.48 – 7.40 (m, 4H), 7.34 (dd, *J* = 7.6, 1.2 Hz, 1H), 5.46 (s, 2H), 3.95 (t, *J* = 8.1 Hz, 2H), 3.19 (t, *J* = 8.1 Hz, 2H).

^{13}C{H} NMR (126 MHz, CD_2Cl_2) δ 168.0, 138.5, 136.4, 135.1, 131.4, 130.2, 130.1 (2C), 129.8 (2C), 128.9, 128.5, 125.1, 64.3, 48.2, 25.7.

HRMS & IR: *compound unstable.*

*1-(4-phenylbenzyl))-2,3,4,5-tetrahydropyridine-1-ium chloride (**3n**):*

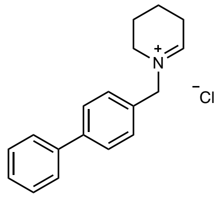

Prepared according to general procedure **1** using 1-(4-phenylbenzyl) piperidine *N*-oxide (38.2 mg, 0.20 mmol, 1.0 equiv) and pivaloyl chloride (163 μL, 1.32 mmol, 6.6 equiv). After warming to rt, 1,1,2,2-tetrachloroethane (21 μL, 0.20 mmol, 1.0 equiv) was added as an internal standard and the yield was determined *via* quantitative ^{1}H NMR spectroscopy (77%, >20:1 r.r.).

^{1}H NMR (500 MHz, CD_2Cl_2) δ 9.53 (s, 1H), 7.67 – 7.62 (m, 4H), 7.59 – 7.56 (m, 2H), 7.45 – 7.41 (m, 2H), 7.38 – 7.34 (m, 1H), 5.34 (s, 2H), 3.73 – 3.66 (m, 2H), 3.11 – 3.05 (m, 2H), 1.99 – 1.92 (m, 2H), 1.83 – 1.78 (m, 2H).

^{13}C{H} NMR (126 MHz, CD_2Cl_2) δ 180.5, 142.9, 140.2, 130.9 (2C), 130.5, 129.3 (2C), 128.3 (2C), 128.2, 127.4 (2C), 65.4, 49.7, 29.4, 20.9, 15.8.

HRMS & IR: *compound unstable.*

*1-(4-phenylbenzyl)-2-methyl-2,3,4,5-tetrahydropyridin-1-ium chloride (**3o**) and 1-(4-phenylbenzyl)-6-methyl-2,3,4,5-tetrahydropyridin-1-ium chloride (**3o'**):*

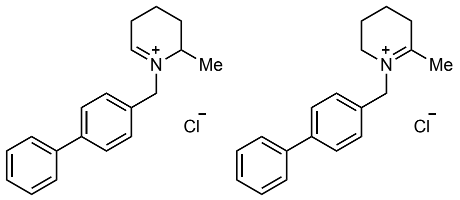

Prepared according to general procedure **1** using 1-(4-phenylbenzyl)-2-methylpiperidine *N*-oxide (56.2 mg, 0.20 mmol, 1.0 equiv) and pivaloyl chloride (163 μL, 1.32 mmol, 6.6 equiv). After warming to rt, 1,1,2,2-tetrachloroethane (21 μL, 0.20 mmol, 1.0 equiv) was added as an internal standard and the yield was determined *via* quantitative ^{1}H NMR spectroscopy (total 100%, 1:5.3 r.r.).

<u>*Major regioisomer (84%).*</u>

*1-(4-phenylbenzyl)-6-methyl-2,3,4,5-tetrahydropyridin-1-ium chloride (**3o'**):*

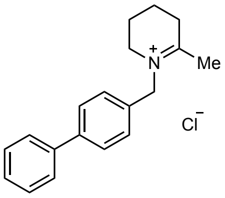

^{1}H NMR (700 MHz, CD_2Cl_2) δ 7.68 – 7.66 (m, 2H), 7.59 (dd, *J* = 7.4, 0.9 Hz, 2H), 7.46 – 7.43 (m, 4H), 7.38 – 7.35 (m, 1H), 5.24 (s, 2H), 3.81 (dt, *J* = 7.6, 3.5 Hz, 2H), 3.11 (t, *J* = 6.3 Hz, 2H), 2.72 (s, 3H), 2.00 – 1.94 (m, 2H), 1.92 – 1.85 (m, 2H).

^{13}C{H} NMR (176 MHz, CD_2Cl_2) δ 190.4, 142.3, 140.2, 130.6, 129.3 (2C), 128.9 (2C), 128.4 (2C), 128.2, 127.3 (2C), 60.2, 53.5, 35.9, 25.2, 21.2, 17.5.

HRMS & IR: *compound unstable.*

Minor regioisomer (16%).

*1-(4-phenylbenzyl)-2-methyl-2,3,4,5-tetrahydropyridin-1-ium chloride (**3o**):*

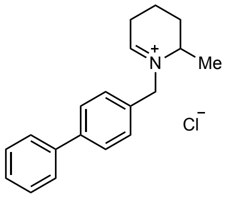

^{1}H NMR (700 MHz, CD_2Cl_2) δ 9.40 (s, 1H), 7.66 – 7.64 (m, 2H), 7.61 (d, *J* = 8.4 Hz, 2H), 7.47 – 7.42 (m, 4H), 7.40 (t, *J* = 7.8 Hz, 1H), 5.50 (d, *J* = 14.4 Hz, 1H), 5.11 (d, *J* = 14.4 Hz, 1H), 3.99 (d, *J* = 7.4 Hz, 1H), 3.16 (d, *J* = 22.9 Hz, 1H), 3.09 – 3.01 (m, 1H), 1.95 – 1.91 (m, 2H), 1.86 – 1.78 (m, 2H), 1.51 (d, *J* = 6.8 Hz, 3H).

^{13}C{H} NMR (176 MHz, CD_2Cl_2) δ 181.3, 142.9, 130.5, 130.3 (2C), 130.1, 128.3 (2C), 128.2, 128.2 (2C), 127.4 (2C), 62.9, 55.6, 29.5, 27.6, 18.4, 12.3.

HRMS & IR: *compound unstable.*

5. α-functionalized cyclic amine scope

5.1 Zn mediated alkylation scope

1-benzyl-2-isopropyl-piperidine (6a):

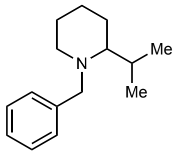

Prepared according to general procedure **3** using 1-benzylpiperidine *N*-oxide (38.2 mg, 0.20 mmol, 1.0 equiv), pivaloyl chloride (62 μL, 0.50 mmol, 2.5 equiv), copper perchlorate (78.2 mg, 0.30 mmol, 1.5 equiv), TBSOTf (12 μL, 0.05 mmol, 25 mol%), copper iodide (19.0 mg, 0.10 mmol, 50 mol%), 2-iodopropane (60 μL, 0.60 mmol, 3.0 equiv) and zinc dust (26.2 mg, 0.4 mmol, 2.0 equiv). Purification by automated flash column chromatography (RediSep Rf Gold Si 4g, 0–30% [10% Et_2O and 3% Et_3N in PE] in PE) delivered the title compound as a pale yellow oil (28.2 mg, 65%, 0.13 mmol).

Spectra are in accordance with literature data. [34]

Scale up: 1-benzyl-2-isopropyl-piperidine (6a):

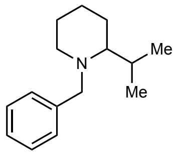

Prepared according to general procedure **3.** A solution of pivaloyl chloride (308 µL, 2.5 mmol, 2.5 equiv) in anhydrous CH_2Cl_2 (1.0 mL, 1.0 M) was cooled to –78 °C. To this was added dropwise a solution of 1-benzylpiperidine *N*-oxide (191 mg, 1.0 mmol, 1.0 equiv), in anhydrous CH_2Cl_2 (1.0 mL, 1.0 M), which was also cooled to –78 ˚C. The vial containing the *N*-oxide was washed with CH_2Cl_2 (1.0 mL), cooled to –78 ˚C and added dropwise to the pivaloyl chloride solution. After complete addition, the mixture was stirred for a further 15 mins at –78 ˚C before warming slowly to rt over 5 h.

After warming to rt, copper perchlorate (391 mg, 1.5 mmol, 1.5 equiv) was added and the mixture was stirred vigorously for 30 mins at rt.

Next, TBSOTf (57 µL, 0.25 mmol, 25 mol%) was added, copper iodide (95 mg, 0.5 mmol, 50 mol%), 2-iodopropane (300 µL, 3.0 mmol, 3.0 equiv) and zinc dust (131 mg, 2.0 mmol, 2.0 equiv) in that order. The reaction mixture was heated to 30 ˚C using an oil bath and vigorously stirred for 15 h. The crude reaction mixture was cooled and diluted with CH_2Cl_2 (75 mL), 40% (w/w) NaOH solution (100 mL) was added, and the resultant mixture was stirred vigorously for a further 3.5 h. The aqueous layer was extracted with CH_2Cl_2 (3 x 75 mL), and the combined organic layers were dried over Na_2SO_4, filtered, and concentrated *in vacuo*. Purification by automated flash column chromatography (RediSep Rf Gold Si 4g, 0–3% EtOAc in PE) delivered the title compound as a pale yellow oil (113 mg, 52%, 0.52 mmol).

Spectra are in accordance with literature data.[34]

1-benzyl-2-ethyl-piperidine (6b):

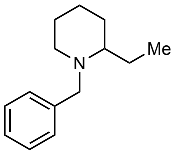

Prepared according to general procedure **2** using 1-benzylpiperidine *N*-oxide (38.2 mg, 0.20 mmol, 1.0 equiv), pivaloyl chloride (62 µL, 0.50 mmol, 2.5 equiv), TBSOTf (35 µL, 0.10 mmol, 75 mol%), copper iodide (15.2 mg, 0.08 mmol, 40 mol%), 1-iodoethane (40 µL, 0.50 mmol, 2.5 equiv) and zinc dust (33.7 mg, 0.50 mmol, 2.5 equiv). Purification by automated flash column chromatography (RediSep Rf Gold Si 4g, 0–40% [10% Et_2O

and 3% Et_3N in PE] in PE) delivered the title compound as a colourless oil (18.9 mg, 47%, 0.09 mmol).

Spectra are in accordance with literature data. [35]

1-benzyl-2-(tert-butyl)-piperidine (6c):

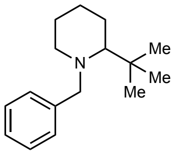

Prepared according to general procedure **2** using 1-benzylpiperidine *N*-oxide (38.2 mg, 0.20 mmol, 1.0 equiv), pivaloyl chloride (62 μL, 0.50 mmol, 2.5 equiv), TBSOTf (23 μL, 0.10 mmol, 50 mol%), copper iodide (19.0 mg, 0.10 mmol, 50 mol%), 2-iodo-2-methylpropane (72 μL, 0.60 mmol, 3.0 equiv) and zinc dust (39.2 mg, 0.60 mmol, 3.0 equiv). Purification by automated flash column chromatography (RediSep Rf Gold Si 4g, 0–30% [10% Et_2O and 3% Et_3N in PE] in PE) followed by SCX delivered the title compound as a colourless oil (28.2 mg, 61%, 0.12 mmol).

Spectra are in accordance with literature data. [36]

2-adamantan-1-yl-1-benzyl-piperidine (6d):

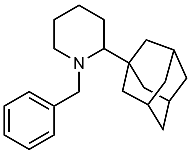

Prepared according to general procedure **3** using 1-benzylpiperidine *N*-oxide (38.2 mg, 0.20 mmol, 1.0 equiv), pivaloyl chloride (62 μL, 0.50 mmol, 2.5 equiv), copper perchlorate (78.2 mg, 0.30 mmol, 1.5 equiv), TBSOTf (23 μL, 0.10 mmol, 50 mol%), copper iodide (19.0 mg, 0.10 mmol, 50 mol%), 1-iodoadamantane (157.3 mg, 0.60 mmol, 3.0 equiv) and zinc dust (39.2 mg, 0.60 mmol, 3.0 equiv). Purification by reverse phase automated flash column chromatography (RediSep Rf 5.5 g C18-column, 0–100% MeCN in H_2O) followed by SCX delivered the title compound as a colourless oil (43.5 mg, 70%, 0.14 mmol).

Spectra are in accordance with literature data. [37]

1-benzyl-2-cyclohexyl-piperidine **(6e):**

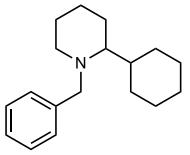

Prepared according to general procedure **2** using 1-benzylpiperidine *N*-oxide (38.2 mg, 0.20 mmol, 1.0 equiv), pivaloyl chloride (62 μL, 0.50 mmol, 2.5 equiv), TBSOTf (12 μL, 0.05 mmol, 25 mol%), copper iodide (19.0 mg, 0.10 mmol, 50 mol%), iodocyclohexane (52 μL, 0.40 mmol, 2.0 equiv) and zinc dust (26.1 mg, 0.40 mmol, 2.0 equiv). Purification by SCX, followed by automated flash column chromatography (RediSepRf Gold Si 4g, 0–40% [10% Et_2O and 3% Et_3N in PE] in PE) delivered the title compound as a colourless oil (27.4 mg, 53%, 0.10 mmol).

^{1}H NMR (500 MHz, $CDCl_3$) δ 7.40 – 7.30 (m, 4H), 7.27 – 7.21 (m, 1H), 4.10 (d, *J* = 13.4 Hz, 1H), 3.17 (d, *J* = 13.4 Hz, 1H), 2.85 (dt, *J* = 12.0, 3.3 Hz, 1H), 2.08 (ddd, *J* = 9.9, 5.1, 2.8 Hz, 1H), 2.00 (ddd, *J* = 12.0, 10.7, 3.3 Hz, 1H), 1.92 – 1.61 (m, 8H), 1.52 – 1.00 (m, 9H).

^{13}C{H} NMR (126 MHz, $CDCl_3$) δ 140.6, 129.0 (2C), 128.2 (2C), 126.6, 66.3, 56.6, 52.5, 39.0, 31.0, 27.4, 27.2, 27.2, 27.0, 24.9, 24.7, 24.5.

HRMS: *m/z* calculated for $C_{18}H_{28}N$ $[M+H]^+$ 258.2216; found 258.2218.

IR (film, cm^{-1}): 3025, 2925, 2851, 2784, 1449, 733, 696.

(1-benzylpiperidin-2-yl) methyl pivalate **(6f):**

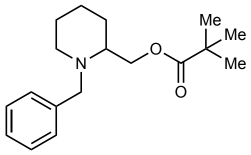

Prepared according to general procedure **2** using 1-benzylpiperidine *N*-oxide (38.2 mg, 0.20 mmol, 1.0 equiv), pivaloyl chloride (62 μL, 0.50 mmol, 2.5 equiv), TBSOTf (35 μL, 0.10 mmol, 75 mol%), copper iodide (15.2 mg, 0.08 mmol, 40 mol%), iodomethyl pivlate (78 μL, 0.50 mmol, 2.5 equiv) and zinc dust (33.2 mg, 0.50 mmol, 2.5 equiv). Purification by SCX, followed by automated flash column chromatography (RediSep Rf Gold Si 4g, 0–5% EtOAc in PE) delivered the title compound as a pink oil (33.2 mg, 57%, 0.11 mmol).

^{1}H NMR (500 MHz, $CDCl_3$) δ 7.36 – 7.28 (m, 4H), 7.26 – 7.21 (m, 1H), 4.29 (dd, *J* = 11.6, 5.0 Hz, 1H), 4.19 (dd, *J* = 11.6, 5.0 Hz, 1H), 4.07 (d, *J* = 13.6 Hz, 1H), 3.32 (d, *J* = 13.6 Hz, 1H), 2.76 (dt, *J* = 11.5, 4.0 Hz, 1H), 2.65 – 2.56 (m, 1H), 2.14 – 2.02 (m, 1H), 1.83 – 1.74 (m, 1H), 1.73 – 1.65 (m, 1H), 1.56 – 1.42 (m, 3H), 1.40 – 1.31 (m, 1H), 1.21 (s, 9H).

^{13}**C{H} NMR** (126 MHz, $CDCl_3$) δ 178.6, 139.4, 128.9 (2C), 128.4 (2C), 127.0, 66.1, 60.5, 59.1, 51.7, 38.9, 29.3, 27.3 (3C), 25.4, 23.3.

HRMS: *m/z* calculated for $C_{18}H_{27}NO_2$ $[M+H]^+$ 290.2115; found 290.2120.

IR (film, cm^{-1}): 2934, 2858, 2794, 1728, 1480, 1453, 1283, 1152, 1065, 1030, 978, 734, 698.

(1-benzylpiperidin-2-yl) methyl benzoate **(6g):**

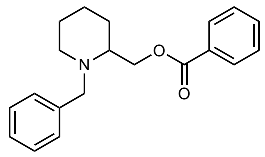

Prepared according to general procedure **2** using 1-benzylpiperidine *N*-oxide (38.2 mg, 0.20 mmol, 1.0 equiv), pivaloyl chloride (62 μL, 0.50 mmol, 2.5 equiv), TBSOTf (35 μL, 0.10 mmol, 75 mol%), copper iodide (15.2 mg, 0.08 mmol, 40 mol%), iodomethyl benzoate (131.0 mg, 0.50 mmol, 2.5 equiv) and zinc dust (33.2 mg, 0.50 mmol, 2.5 equiv). Purification by SCX, followed by automated flash column chromatography (RediSep Rf Gold Si 4g, 0–20% EtOAc in PE) delivered the title compound as a yellow oil (32.7 mg, 53%, 0.11 mmol).

^{1}**H NMR** (500 MHz, $CDCl_3$) δ 8.12 – 8.01 (m, 2H), 7.60 – 7.53 (m, 1H), 7.48 – 7.43 (m, 2H), 7.37 – 7.33 (m, 2H), 7.31 – 7.27 (m, 2H), 7.25 – 7.20 (m, 1H), 4.56 (dd, *J* = 11.5, 5.0 Hz, 1H), 4.45 (dd, *J* = 11.5, 5.2 Hz, 1H), 4.12 (d, *J* = 13.6 Hz, 1H), 3.41 (d, *J* = 13.6 Hz, 1H), 2.85 – 2.72 (m, 2H), 2.18 – 2.11 (m, 1H), 1.90 – 1.83 (m, 1H), 1.76 – 1.69 (m, 1H), 1.64 – 1.47 (m, 3H), 1.45 – 1.36 (m, 1H).

^{13}**C{H} NMR** (126 MHz, $CDCl_3$) δ 166.6, 139.8, 133.1, 130.4, 129.7 (2C), 128.9 (2C), 128.5 (2C), 128.3 (2C), 126.9, 66.5, 60.0, 59.1, 51.7, 29.4, 25.5, 23.2.

HRMS: *m/z* calculated for $C_{20}H_{24}NO_2$ $[M+H]^+$ 310.1802; found 310.1808.

IR (film, cm^{-1}): 2929, 2861, 1703, 1638, 1434, 1390, 1372, 1285, 1129, 1066, 1022, 944, 765, 700.

***(1-benzylpiperidin-2-yl) methanol* (6h):**

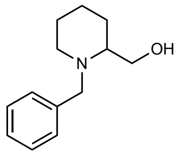

Prepared according to general procedure **2** using 1-benzylpiperidine *N*-oxide (38.2 mg, 0.20 mmol, 1.0 equiv), pivaloyl chloride (62 μL, 0.50 mmol, 2.5 equiv), TBSOTf (35 μL, 0.10 mmol, 75 mol%), copper iodide (15.2 mg, 0.08 mmol, 40 mol%), iodomethyl benzoate (131.0 mg, 0.50 mmol, 2.5 equiv) and zinc dust (33.2 mg, 0.50 mmol, 2.5 equiv). After concentrating *in vacuo*, the crude α-alkylated cyclic amine was dissolved in MeOH/THF (12 mL, 5:1) and K_2CO_3 (82.9 mg, 0.60 mmol, 3.0 equiv) was added. The mixture was stirred for 24 h and then quenched with saturated ammonium chloride solution (20 mL) for 30 mins. The solution was diluted with water and the aqueous layer was washed several times with EtOAc (6 x 20 mL). The combined organic layers were dried over $MgSO_4$, filtered, and concentrated *in vacuo*. Purification by SCX delivered the title compound as a brown oil (21.4 mg, 52%, 0.10 mmol).

Spectra are in accordance with literature data. [38]

1-(4-phenyl-benzyl)-2-(fluoromethyl)piperidine (6i):

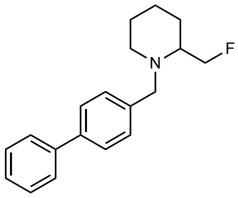

Prepared according to general procedure **2** using 1-(4-phenylbenzyl)piperidine *N*-oxide (53.5 mg, 0.20 mmol, 1.0 equiv), pivaloyl chloride (62 μL, 0.50 mmol, 2.5 equiv), TBSOTf (35 μL, 0.10 mmol, 75 mol%), copper iodide (15.2 mg, 0.08 mmol, 40 mol%), fluoroiodomethane (35 μL, 0.50 mmol, 2.5 equiv) and zinc dust (33.2 mg, 0.50 mmol, 2.5 equiv). Purification by SCX, followed by automated flash column chromatography (RediSep Rf Gold Si 4g, 0–10% EtOAc in PE) delivered the title compound as a colourless oil (23.4 mg, 41%, 0.08 mmol).

^{1}H NMR (500 MHz, $CDCl_3$) δ 7.62 – 7.58 (m, 2H), 7.57 – 7.52 (m, 2H), 7.47 – 7.39 (m, 4H), 7.36 – 7.30 (m, 1H), 4.71 – 4.44 (m, 2H), 4.12 (d, *J* = 13.8 Hz, 1H), 3.40 (d, *J* = 13.8 Hz, 1H), 2.87 – 2.80 (m, 1H), 2.70 – 2.58 (m, 1H), 2.09 (ddd, *J* = 11.7, 10.2, 3.2 Hz, 1H), 1.77 – 1.67 (m, 2H), 1.59 – 1.53 (m, 1H), 1.52 – 1.42 (m, 2H), 1.41 – 1.31 (m, 1H).

^{13}C{H} NMR (126 MHz, $CDCl_3$) δ 141.2, 139.9, 138.71, 129.4 (2C), 128.9 (2C), 127.2, 127.2 (2C), 127.0 (2C), 86.6 (d, *J* = 170.0 Hz), 61.2 (d, *J* = 17.7 Hz), 58.9 (d, *J* = 3.2 Hz), 52.2, 28.4 (d, *J* = 7.2 Hz), 25.5, 23.6 (d, *J* = 0.9 Hz).

^{19}**F{H} NMR** (471 MHz, $CDCl_3$) δ -218.4.

HRMS: *m/z* calculated for $C_{19}H_{23}FN$ $[M+H]^+$ 284.1809; found 284.1816.

IR (film, cm^{-1}): 3027, 2931, 2855, 2794, 1487, 1443, 1338, 1115, 1081, 1055, 1008, 988, 928, 839, 756, 697.

(1-benzyl-2-methoxymethyl) piperidine **(6j):**

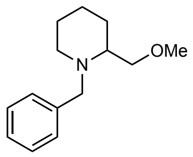

Prepared according to general procedure **2** using 1-benzylpiperidine *N*-oxide (38.2 mg, 0.20 mmol, 1.0 equiv), pivaloyl chloride (62 μL, 0.50 mmol, 2.5 equiv), TBSOTf (35 μL, 0.10 mmol, 75 mol%), copper iodide (15.2 mg, 0.08 mmol, 40 mol%), iodomethyl methylether (42 μL, 0.50 mmol, 2.5 equiv) and zinc dust (33.2 mg, 0.50 mmol, 2.5 equiv). Purification by SCX, followed by automated flash column chromatography (RediSep Rf Gold Si 4g, 0–30% EtOAc in PE) delivered the title compound as a yellow oil (19.5 mg, 45%, 0.09 mmol).
^{1}**H NMR** (400 MHz, $CDCl_3$) δ 7.36 – 7.28 (m, 4H), 7.25 – 7.19 (m, 1H), 4.09 (d, *J* = 13.7 Hz, 1H), 3.57 (dd, *J* = 9.9, 4.7 Hz, 1H), 3.47 (dd, *J* = 9.9, 4.3 Hz, 1H), 3.33 (m, 4H), 2.76 (dt, *J* = 10.7, 3.7 Hz, 1H), 2.49 – 2.38 (m, 1H), 2.00 (ddd, *J* = 11.7, 10.1, 3.6 Hz, 1H), 1.73 – 1.65 (m, 2H), 1.53 – 1.41 (m, 3H), 1.35 – 1.27 (m, 1H).

^{13}**C{H} NMR** (126 MHz, $CDCl_3$) δ 139.9, 129.1 (2C), 128.2 (2C), 126.7, 76.0, 61.3, 59.0, 58.9, 52.5, 29.9, 25.6, 24.0.

HRMS: *m/z* calculated for $C_{14}H_{22}NO$ $[M+H]^+$ 220.1696; found 220.1709.

IR (film, cm^{-1}): 2924, 2851, 1451, 1129, 1106, 733, 697.

1-benzyl-2-(tetrahydro-2H-pyran-4-yl)piperidine **(6k):**

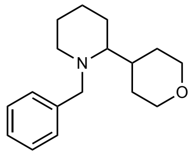

Prepared according to general procedure **2** using 1-benzylpiperidine *N*-oxide (38.2 mg, 0.20 mmol, 1.0 equiv), pivaloyl chloride (62 μL, 0.50 mmol, 2.5 equiv), TBSOTf (34 μL, 0.05 mmol, 75 mol%), copper iodide (9.5 mg, 0.05 mmol, 25 mol%), 4-iodotetrahydro-2H-pyran (60 μL, 0.50 mmol, 2.5 equiv) and zinc dust (19.5 mg, 0.30 mmol, 1.5 equiv). Purification by SCX, followed by two automated flash column

chromatography's (RediSep Rf Gold 4g, 0–40% [10% Et_2O and 3% Et_3N in PE] in PE then RediSep Rf Gold Si 4g, 0–10% EtOAc in PE) delivered the title compound as an orange oil (24.6 mg, 47%, 0.10 mmol).

^{1}H NMR (500 MHz, $CDCl_3$) δ 7.41 – 7.30 (m, 4H), 7.28 – 7.23 (m, 1H), 4.13 – 3.90 (m, 3H), 3.49 – 3.27 (m, 3H), 2.90 (dt, *J* = 12.8, 4.3 Hz, 1H), 2.29 – 2.16 (m, 2H), 2.16 – 2.02 (m, 1H), 1.82 – 1.60 (m, 4H), 1.58 – 1.32 (m, 6H).

^{13}C{H} NMR (126 MHz, $CDCl_3$) δ 139.7, 128.8 (2C), 128.4 (2C), 127.0, 68.8, 68.5, 64.9, 56.1, 51.3, 36.1, 30.6, 28.3, 23.8, 23.7, 23.1.

HRMS: *m/z* calculated for $C_{17}H_{26}NO$ $[M+H]^+$ 260.2009; found 260.2014.

IR (film, cm^{-1}): 2933, 2849, 1648, 1454, 1091, 1064, 987, 746, 700.

Benzyl 1-benzyl-[2,4'-bipiperidine]-1'-carboxylate (6l):

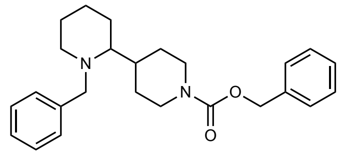

Prepared according to general procedure **2** using 1-benzylpiperidine *N*-oxide (38.2 mg, 0.20 mmol, 1.0 equiv), pivaloyl chloride (62 µL, 0.50 mmol, 2.5 equiv), TBSOTf (23 µL, 0.10 mmol, 50 mol%), copper iodide (19.0 mg, 0.10 mmol, 50 mol%), *N*-Cbz-4-iodopiperidine (129 µL, 0.60 mmol, 3.0 equiv) and zinc dust (39.2 mg, 0.60 mmol, 3.0 equiv). Purification by SCX, followed by automated flash column chromatography (RediSep Rf Gold Si 4g, 0–25% EtOAc in PE) delivered the title compound as a pale yellow oil (39.8 mg, 51%, 0.10 mmol).

^{1}H NMR (500 MHz, $CDCl_3$) δ 7.37 – 7.29 (m, 9H), 7.25 – 7.19 (m, 1H), 5.13 (s, 2H), 4.23 (s, 2H), 4.01 (d, *J* = 13.5 Hz, 1H), 3.29 (d, *J* = 13.5 Hz, 1H), 2.88 – 2.61 (m, 3H), 2.21 – 2.07 (m, 2H), 2.04 – 1.94 (m, 1H), 1.78 – 1.20 (m, 10H).

^{13}C{H} NMR (126 MHz, $CDCl_3$) δ 155.4, 140.4, 137.1, 128.7 (2C), 128.6 (2C), 128.3 (2C), 128.0 (2C), 128.0 (2C), 126.8 (2C), 67.1, 64.8, 56.6, 51.8, 45.0, 44.6, 37.1, 29.8, 27.0, 24.0, 23.7, 23.7.

HRMS: *m/z* calculated for $C_{25}H_{33}N_2O_2$ $[M+H]^+$ 393.2537; found 393.2541.

IR (film, cm^{-1}): 3028, 2932, 2854, 2788, 1697, 1432, 1360, 1278, 1229, 1216, 1136, 1092, 736, 697.

(1-benzyl-2-piperidine)acetonitrile (6m):

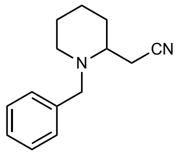

Prepared according to general procedure **2** using 1-benzylpiperidine *N*-oxide (38.2 mg, 0.20 mmol, 1.0 equiv), pivaloyl chloride (62 μL, 0.50 mmol, 2.5 equiv), TBSOTf (35 μL, 0.10 mmol, 75 mol%), copper iodide (15.2 mg, 0.08 mmol, 40 mol%), iodoacetonitrile (36 μL, 0.50 mmol, 2.5 equiv) and zinc dust (33.2 mg, 0.50 mmol, 2.5 equiv). Purification by SCX, followed by automated flash column chromatography (RediSep Rf Gold Si 4g, 0–10% EtOAc in PE) delivered the title compound as a pale pink oil (13.2 mg, 31%, 0.06 mmol).

^{1}H NMR (500 MHz, $CDCl_3$) δ 7.38 – 7.29 (m, 4H), 7.27 – 7.23 (m, 1H), 3.87 (d, *J* = 13.4 Hz, 1H), 3.30 (d, *J* = 13.5 Hz, 1H), 2.74 (tt, *J* = 7.6, 3.7 Hz, 1H), 2.71 – 2.62 (m, 2H), 2.57 (dd, *J* = 16.8, 3.9 Hz, 1H), 2.15 (ddd, *J* = 12.1, 8.5, 3.8 Hz, 1H), 1.88 – 1.80 (m, 1H), 1.72 – 1.63 (m, 2H), 1.57 – 1.37 (m, 3H).

^{13}C{H} NMR (126 MHz, $CDCl_3$) δ 138.8, 128.8 (2C), 128.5 (2C), 127.2, 118.6, 58.9, 57.1, 50.9, 31.2, 25.3, 22.5, 20.2.

HRMS: *m/z* calculated for $C_{14}H_{19}N_2$ $[M+H]^+$ 215.1543; found 215.1551.

IR (film, cm^{-1}): 3024, 2934, 2849, 2798, 2246, 1494, 1451, 1131, 754, 698.

***2-(1-benzylpiperidin-2-yl)acetamide* (6n):**

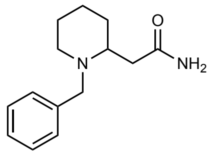

Prepared according to general procedure **2** using 1-benzylpiperidine *N*-oxide (38.2 mg, 0.20 mmol, 1.0 equiv), pivaloyl chloride (62 μL, 0.50 mmol, 2.5 equiv), TBSOTf (35 μL, 0.10 mmol, 75 mol%), copper iodide (15.2 mg, 0.08 mmol, 40 mol%), iodoacetamide (92.5 mg, 0.50 mmol, 2.5 equiv) and zinc dust (33.2 mg, 0.50 mmol, 2.5 equiv). The reaction was quenched with 40% NaOH solution (20 mL) and stirred vigorously for 1.5 h. Purification by automated flash column chromatography (RediSep Rf Gold Si 4g, 0–10% MeOH in EtOAc) followed by SCX delivered the title compound as a yellow oil (16.1 mg, 35%, 0.07 mmol).

^{1}H NMR (400 MHz, $CDCl_3$) δ 8.35 (br s, 1H), 7.36 – 7.25 (m, 5H, H_1), 5.63 (br s, 1H), 4.15 (d, *J* = 12.9 Hz, 1H), 3.23 (d, *J* = 12.9 Hz, 1H), 2.90 – 2.77 (m, 2H), 2.71 – 2.63 (m, 1H), 2.46

(dd, J = 17.0, 4.3 Hz, 1H), 2.09 – 1.97 (m, 1H), 1.79 – 1.62 (m, 3H), 1.62 – 1.52 (m, 1H), 1.47 – 1.31 (m, 2H).

^{13}C{H} NMR (101 MHz, $CDCl_3$) δ 174.3, 138.1, 129.3 (2C), 128.6 (2C), 127.4, 58.4, 57.8, 51.4, 38.5, 30.0, 24.6, 23.8.

HRMS: *m/z* calculated for $C_{14}H_{21}N_2O$ $[M+H]^+$ 233.1648; found 233.1646.

IR (film, cm^{-1}): 3331, 3191, 2931, 2854, 2800, 16662, 1450, 1402, 736, 698.

2-(1-benzylpiperidin-2-yl)-N,N-dimethylacetamide **(6o):**

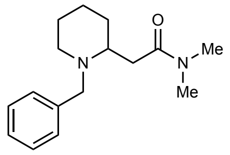

Prepared according to general procedure **2** using 1-benzylpiperidine *N*-oxide (38.2 mg, 0.20 mmol, 1.0 equiv), pivaloyl chloride (62 μL, 0.50 mmol, 2.5 equiv), TBSOTf (35 μL, 0.10 mmol, 75 mol%), copper iodide (15.2 mg, 0.08 mmol, 40 mol%), 2-iodo-*N,N*-dimethylacetamide (59 μL, 0.50 mmol, 2.5 equiv) and zinc dust (33.2 mg, 0.50 mmol, 2.5 equiv). The reaction was quenched with 40% NaOH solution (20 mL) and stirred vigorously for 1.5 h. Purification by automated flash column chromatography (RediSep Rf Gold Si 4g, 0–10% MeOH in EtOAc) followed by SCX delivered the title compound as a yellow oil (15.6 mg, 30%, 0.06 mmol).

^{1}H NMR (700 MHz, $CDCl_3$) δ 7.36 – 7.31 (m, 2H), 7.31 – 7.28 (m, 2H), 7.23 – 7.20 (m, 1H), 3.81 (d, J = 13.7 Hz, 1H), 3.37 (d, J = 13.7 Hz, 1H), 3.11 – 3.05 (m, 1H), 2.96 (s, 3H), 2.93 (s, 3H), 2.74 (dd, J = 15.1, 4.8 Hz, 1H), 2.71 – 2.65 (m, 1H), 2.40 (dd, J = 15.1, 7.6 Hz, 1H), 2.19 (ddd, J = 12.1, 8.8, 3.5 Hz, 1H), 1.81 – 1.75 (m, 1H), 1.67 – 1.62 (m, 1H), 1.54 – 1.39 (m, 4H).

^{13}C{H} NMR (101 MHz, $CDCl_3$) δ 172.1, 139.6, 128.9 (2C), 128.3 (2C), 127.0, 58.8, 57.9, 51.2, 37.6, 35.6, 35.5, 31.4, 25.4, 22.9.

HRMS: *m/z* calculated for $C_{16}H_{25}N_2O$ $[M+H]^+$ 261.1961 found 261.1959.

IR (film, cm^{-1}): 2927, 2853, 1642, 1493, 1451, 1396, 1150.

Ethyl 2-(N-benzylpiperidin-2-yl)-2-methylpropanoate (6p):

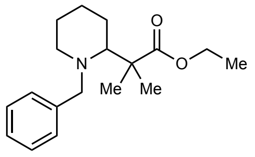

Prepared according to general procedure **2** using 1-benzylpiperidine *N*-oxide (38.2 mg, 0.20 mmol, 1.0 equiv), pivaloyl chloride (62 μL, 0.50 mmol, 2.5 equiv), TBSOTf (23 μL, 0.10 mmol, 50 mol%), copper iodide (19.0 mg, 0.10 mmol, 50 mol%), ethyl 2-iodo-2-methylpropionate (90 μL, 0.60 mmol, 3.0 equiv) and zinc dust (39.0 mg, 0.60 mmol, 3.0 equiv). The reaction was quenched with 40% NaOH solution (20 mL) and stirred vigorously for 1.5 h. Purification by automated flash column chromatography (RediSep Rf Gold Si 4g, 0–10% EtOAc in PE) followed by SCX delivered the title compound as a colourless oil (33.9 mg, 59%, 0.12 mmol).

^{1}H NMR (700 MHz, $CDCl_3$) δ 7.34 – 7.27 (m, 4H), 7.23 – 7.18 (m, 1H), 4.13 (q, *J* = 7.1 Hz, 2H), 3.77 (d, *J* = 13.0 Hz, 1H), 3.23 (d, *J* = 13.0 Hz, 1H), 2.98 – 2.87 (m, 1H), 2.77 – 2.66 (m, 1H), 2.14 – 2.08 (m, 1H), 1.86 – 1.77 (m, 1H), 1.71 – 1.66 (m, 1H), 1.52 – 1.47 (m, 1H), 1.43 – 1.33 (m, 3H), 1.28 (s, 3H), 1.24 (t, *J* = 7.1 Hz, 3H), 1.17 (s, 3H).

^{13}C{H} NMR (101 MHz, $CDCl_3$) δ 179.1, 140.8, 128.5 (2C), 128.3 (2C), 126.7, 66.9, 60.6, 57.9, 50.0, 46.3, 25.7, 24.3, 23.9, 22.8, 19.4, 14.2.

HRMS: *m/z* calculated for $C_{18}H_{28}NO_2$ $[M+H]^+$ 290.2115; found 290.2114.

IR (film, cm^{-1}): 2933, 2857, 2800, 1722, 1451, 1387, 1364, 1249, 1139, 1048, 736, 698.

2-(adamantan-1-yl)-1-methylpiperidine (6q):

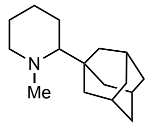

Prepared according to general procedure **3** using 1-methylpiperidine *N*-oxide (23.0 mg, 0.20 mmol, 1.0 equiv), pivaloyl chloride (62 μL, 0.50 mmol, 2.5 equiv), copper perchlorate (78.2 mg, 0.30 mmol, 1.5 equiv), TBSOTf (23 μL, 0.10 mmol, 50 mol%), copper iodide (19.0 mg, 0.10 mmol, 50 mol%), 1-iodoadamantane (157.3 mg, 0.60 mmol, 3.0 equiv) and zinc dust (39.2 mg, 0.60 mmol, 3.0 equiv). Purification by reverse phase automated flash column chromatography (RediSep Rf 5.5 g C18-column, 0–100% MeCN in H_2O) followed SCX delivered the title compound as a colourless oil (20.9 mg, 45%, 0.09 mmol).

^{1}H NMR (700 MHz, $CDCl_3$) δ 2.89 (dt, *J* = 8.9, 4.6 Hz, 1H), 2.81 (ddd, *J* = 13.6, 10.0, 3.5 Hz, 1H), 2.45 (s, 3H), 2.12 (d, *J* = 11.4 Hz, 1H), 2.01 – 1.92 (m, 3H), 1.82 (dq, *J* = 10.8, 4.8 Hz, 1H), 1.74 – 1.56 (m, 13H), 1.55 – 1.49 (m, 1H), 1.38 (tdd, *J* = 13.2, 7.7, 4.5 Hz, 3H).

^{13}C{H} NMR (176 MHz, $CDCl_3$) δ 71.1, 55.8, 40.0 (3C), 39.4, 37.4 (3C), 36.6, 28.9 (3C), 24.6, 19.2, 18.0.

HRMS: *m/z* calculated for $C_{16}H_{27}N$ $[M+H]^+$ 234.2216; found 234.2220.

IR (film, cm^{-1}): 2900, 2846, 1446, 1342, 1259, 1166, 1015.

2-(tert-butyl)-1-(3-phenylpropyl)piperidine (6r):

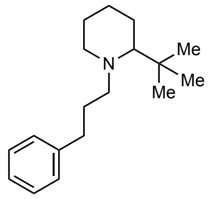

Prepared according to general procedure **2** using 1-(3-phenylpropyl)piperidine *N*-oxide (43.8 mg, 0.20 mmol, 1.0 equiv), pivaloyl chloride (62 μL, 0.50 mmol, 2.5 equiv), TBSOTf (23 μL, 0.10 mmol, 50 mol%), copper iodide (19.0 mg, 0.10 mmol, 50 mol%), 2-iodo-2-methylpropane (72 μL, 0.60 mmol, 3.0 equiv) and zinc dust (39.2 mg, 0.60 mmol, 3.0 equiv). Purification by SCX followed by automated flash column chromatography (RediSep Rf Gold Si 4g, 0–10% EtOAc in PE) delivered the title compound as a yellow oil (18.4 mg, 36%, 0.07 mmol).

^{1}H NMR (700 MHz, $CDCl_3$) δ 7.28 (d, *J* = 7.6 Hz, 2H), 7.21 – 7.15 (m, 3H), 2.91 (ddd, *J* = 14.1, 8.6, 4.4 Hz, 1H), 2.74 – 2.63 (m, 3H), 2.60 (ddd, *J* = 14.6, 9.4, 6.3 Hz, 2H), 2.20 (dd, *J* = 9.6, 4.8 Hz, 1H), 1.85 – 1.74 (m, 3H), 1.51 – 1.37 (m, 5H), 0.89 (s, 9H).

^{13}C{H} NMR (176 MHz, $CDCl_3$) δ 142.9, 128.5 (2C), 128.4 (2C), 125.7, 70.1, 52.3, 46.6, 35.7, 33.9, 30.8, 28.0 (3C), 22.5, 20.6, 18.5.

HRMS: *m/z* calculated for $C_{18}H_{29}N$ $[M+H]^+$ 260.2373; found 260.2375.

IR (film, cm^{-1}): 2928, 2859, 1647, 1453, 1357, 1052, 1017, 743, 696.

2-(adamantan-1-yl)-1-cyclohexylpiperidine (6s):

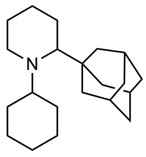

Prepared according to general procedure **3** using 1-cyclohexylpiperidine *N*-oxide (36.6 mg, 0.20 mmol, 1.0 equiv), pivaloyl chloride (62 μL, 0.50 mmol, 2.5 equiv), copper perchlorate (78.2 mg, 0.30 mmol, 1.5 equiv), TBSOTf (23 μL, 0.10 mmol, 50 mol%),

copper iodide (19.0 mg, 0.10 mmol, 50 mol%), 1-iodoadamantane (157.0 mg, 0.60 mmol, 3.0 equiv) and zinc dust (39.2 mg, 0.60 mmol, 3.0 equiv). Purification by reverse phase automated flash column chromatography (RediSep Rf 5.5 g C18-column, 0–100% MeCN in H_2O) followed SCX delivered the title compound as an orange oil (31.0 mg, 51%, 0.10 mmol).

^{1}H NMR (700 MHz, $CDCl_3$) δ 2.82 (dd, *J* = 14.9, 6.2 Hz, 1H), 2.76 – 2.67 (m, 1H), 2.28 (tt, *J* = 11.2, 3.3 Hz, 1H), 2.13 (t, *J* = 7.1 Hz, 1H), 1.96 – 1.89 (m, 4H), 1.86 – 1.80 (m, 1H), 1.80 – 1.72 (m, 2H), 1.69 – 1.50 (m, 15H), 1.48 – 1.39 (m, 3H), 1.32 – 1.19 (m, 3H), 1.18 – 1.10 (m, 2H), 1.05 (qt, *J* = 12.7, 3.6 Hz, 1H).

^{13}C{H} NMR (176 MHz, $CDCl_3$) δ 67.1, 65.3, 41.2, 40.0 (3C), 39.6, 37.7 (3C), 33.5, 32.4, 29.0 (3C), 27.1, 27.0, 26.5, 24.3, 20.9, 20.8.

HRMS: *m/z* calculated for $C_{21}H_{35}N$ $[M+H]^+$ 302.2842; found 302.2840.

IR (film, cm^{-1}): 2898, 2844, 1447, 1343, 1170, 1099, 994, 889.

2-(adamantan-1-yl)-1-benzylpyrrolidine (6u) and 1-((adamantan-1-yl)(phenyl)methyl)pyrrolidine (6u'):

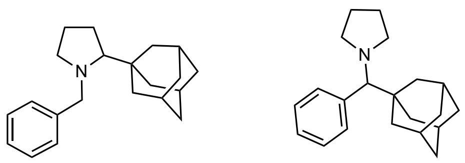

Prepared according to general procedure **3** using 1-benzylpyrrolidine *N*-oxide (35.4 mg, 0.20 mmol, 1.0 equiv), pivaloyl chloride (62 μL, 0.50 mmol, 2.5 equiv), copper perchlorate (78.2 mg, 0.30 mmol, 1.5 equiv), TBSOTf (23 μL, 0.10 mmol, 50 mol%), copper iodide (19.0 mg, 0.10 mmol, 50 mol%), 1-iodoadamantane (157.0 mg, 0.60 mmol, 3.0 equiv) and zinc dust (39.2 mg, 0.60 mmol, 3.0 equiv). Purification by reverse phase automated flash column chromatography (RediSep Rf 5.5 g C18-column, 0–100% MeCN in H_2O) followed by automated flash column chromatography (RediSep Rf Gold Si 4g, 0–5% EtOAc in PE) delivered the two regioisomers (total 28.8 mg, 49%, 0.10 mmol, 2.3:1 r.r.).

<u>*Major regioisomer (20.0 mg, 34%, 0.07 mmol).*</u>

2-(adamantan-1-yl)-1-benzylpyrrolidine (6u):

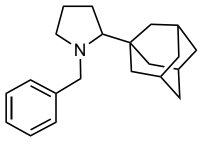

Obtained as an amorphous yellow solid.

^{1}**H NMR** (700 MHz, $CDCl_3$) δ 7.43 (d, *J* = 7.5 Hz, 2H), 7.31 (t, *J* = 7.6 Hz, 2H), 7.22 (t, *J* = 7.3 Hz, 1H), 4.04 (d, *J* = 14.1 Hz, 1H), 3.49 (d, *J* = 14.1 Hz, 1H), 2.81 (ddd, *J* = 10.2, 6.1, 4.5 Hz, 1H), 2.36 (dd, *J* = 9.3, 4.3 Hz, 1H), 2.29 – 2.21 (m, 1H), 1.99 – 1.95 (m, 3H), 1.78 – 1.73 (m, 1H), 1.72 – 1.58 (m, 15H).

^{13}**C{H} NMR** (176 MHz, $CDCl_3$) δ 141.8, 128.2 (2C), 128.2 (2C), 126.5, 73.8, 63.5, 55.5, 39.5 (3C), 38.0, 37.6 (3C), 28.7 (3C), 26.1, 25.6.
HRMS: *m/z* calculated for $C_{21}H_{29}N$ $[M+H]^+$ 296.2373; found 296.2375.

IR (film, cm^{-1}): 2899, 2845, 2792, 1450, 1127, 1103, 769, 703.

Minor regioisomer (8.8 mg, 15%, 0.03 mmol).

1-((adamantan-1-yl)(phenyl)methyl)pyrrolidine (6u'):

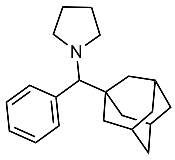

Obtained as a colourless oil.

^{1}**H NMR** (700 MHz, $CDCl_3$) δ 7.29 – 7.26 (m, 2H), 7.23 (d, *J* = 7.3 Hz, 3H), 3.04 (s, 1H), 2.61 – 2.44 (m, 4H), 1.92 (h, *J* = 3.0 Hz, 3H), 1.83 (dd, *J* = 12.6, 3.0 Hz, 3H), 1.66 – 1.57 (m, 10H), 1.51 – 1.41 (m, 3H).

^{13}**C{H} NMR** (176 MHz, $CDCl_3$) δ 140.1, 130.4 (2C), 127.5 (2C), 126.5, 79.2, 53.7 (2C), 41.0 (3C), 37.9, 37.3 (2C), 29.0 (3C), 23.3 (2C).

HRMS: *m/z* calculated for $C_{21}H_{29}N$ $[M+H]^+$ 296.2373; found 296.2375.

IR (film, cm^{-1}): 2899, 2843, 2780, 1493, 1450, 1359, 1256, 1104, 1027, 734, 696.

(2S,6S)-1-(4-phenylbenzyl)-2-ethyl-6-methylpiperidine (11):

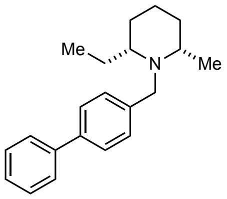

Prepared according to general procedure **2** using 1-(4-phenylbenzyl)-2-methylpiperidine *N*-oxide (56.2 mg, 0.20 mmol, 1.0 equiv), pivaloyl chloride (62 μL, 0.50 mmol, 2.5 equiv), TBSOTf (34 μL, 0.15 mmol, 75 mol%), copper iodide (15.2 mg, 0.08 mmol, 40 mol%),

iodoethane (40 μL, 0.50 mmol, 2.5 equiv) and zinc dust (33.7 mg, 0.50 mmol, 2.5 equiv). Purification by reverse phase automated flash column chromatography (RediSep Rf 5.5 g C18-column, 0–100% MeCN in H_2O) delivered the title compound as a yellow oil and as a single diastereomer (11.6 mg, 12%, 0.02 mmol, >20:1 dr).

^{1}H NMR (700 MHz, $CDCl_3$) δ 7.61 – 7.59 (m, 2H), 7.52 (d, *J* = 7.8 Hz, 2H), 7.46 – 7.42 (m, 4H), 7.33 – 7.31 (m, 1H), 3.84 – 3.74 (m, 2H), 2.62 (br s, 1H), 2.36 (br s, 1H), 1.73 – 1.66 (m, 3H), 1.54 (br s, 1H), 1.34 (d, *J* = 12.9 Hz, 2H), 1.29 (d, *J* = 10.1 Hz, 2H), 1.09 – 1.02 (m, 3H), 0.83 (t, *J* = 7.4 Hz, 3H).

^{13}C{H} NMR (176 MHz, $CDCl_3$) δ 142.3, 141.3, 139.0, 128.8 (2C), 128.3 (2C), 127.1 (2C), 127.1, 126.8 (2C), 64.4, 58.2, 52.8, 33.2, 29.4, 27.8, 24.2, 22.3, 11.0.

HRMS: *m/z* calculated for $C_{21}H_{27}N$ $[M+H]^+$ 294.2216; found 294.2220.

IR (film, cm^{-1}): 2926, 2853, 1643, 1487, 1460, 1190, 1007, 837, 756, 697.

The relative stereochemistry of the title compound was determined by analogy to compound **12’**. See page 57.

(2R,4S)-1-benzyl-2-ethyl-4-methylpiperidine (13):

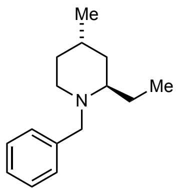

Prepared according to general procedure **2** using 1-benzyl-4-methylpiperidine *N*-oxide (41.0 mg, 0.20 mmol, 1.0 equiv), pivaloyl chloride (62 μL, 0.50 mmol, 2.5 equiv), TBSOTf (35 μL, 0.10 mmol, 75 mol%), copper iodide (15.2 mg, 0.08 mmol, 40 mol%), 1-iodoethane (40 μL, 0.50 mmol, 2.5 equiv) and zinc dust (33.7 mg, 0.50 mmol, 2.5 equiv). Purification by automated flash column chromatography (RediSep Rf Gold Si 4g, 0–20% EtOAc in PE) delivered the title compound as a yellow oil and as a single diastereomer (20.5 mg, 47%, 0.09 mmol, d.r. >20:1).

^{1}H NMR (500 MHz, $CDCl_3$) δ 7.36 (d, *J* = 7.6 Hz, 2H), 7.30 (t, *J* = 7.7 Hz, 2H), 7.22 (t, *J* = 7.3 Hz, 1H), 3.65 (q, *J* = 12.4 Hz, 2H), 2.64 (dq, *J* = 8.7, 4.3 Hz, 1H), 2.56 (td, *J* = 11.8, 3.1 Hz, 1H), 2.49 (dd, *J* = 10.2, 6.2 Hz, 1H), 1.69 – 1.57 (m, 3H), 1.51 (ddd, *J* = 11.5, 8.1, 5.8 Hz, 2H), 1.36 (ddd, *J* = 13.1, 11.0, 4.5 Hz, 1H), 1.25 – 1.19 (m, 1H), 0.91 (d, *J* = 6.6 Hz, 3H), 0.85 (t, *J* = 7.5 Hz, 3H).

^{13}C{H} NMR (126 MHz, $CDCl_3$) δ 140.5, 128.8 (2C), 128.2 (2C), 126.7, 59.0, 58.7, 46.2, 35.3, 33.0, 25.3, 22.2, 18.6, 11.5.

HRMS: *m/z* calculated for $C_{15}H_{23}N$ $[M+H]^+$ 218.1900; found 218.1903.

IR (film, cm^{-1}): 2948, 2921, 2870, 2796, 1493, 1453, 1363, 1122, 1067, 1028, 974, 754, 731, 696.

The relative stereochemistry of the title compound was determined by 2D NMR experimentation and analysis of the *J*-coupling constants. The N-CH-C**H$_2$** proton at 1.36 ppm exhibits a single *J*-coupling constant of 11.0 Hz, which is the result of an axial-axial coupling. Consequently, the two substituents must be on opposite faces of the ring, resulting in the *anti*-configuration.

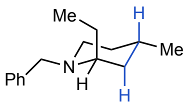

$^3J_{ax\text{-}ax}$ = 11.5 Hz

Additionally, a clear NOE signal between the Me-C**H** at 1.70 ppm and C**H$_2$** of the ethyl group at 1.51 ppm was observed. To obtain the NOE data, the proton signal corresponding to the Me-C**H** group at 1.70 ppm was selectively irradiated using appropriate pulse sequences. Analysis of the 2D NMR data revealed a strong correlation to the Me group, as expected. The next strongest correlation was to the signal just above 1.50 ppm. Very careful inspection of the HSQC spectrum with high resolution indicates that one of the ethyl CH_2 protons contributes a major part of this signal. This spatial proximity is consistent with the *anti*-configuration and suggests that both the Me-C**H** proton and Et group adopt axial positions in the chair conformation.

2-benzyl-1-(tert-butyl)-1,2,3,4-tetrahydroisoquinoline (14a):

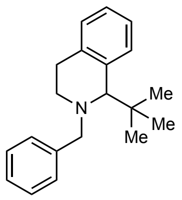

Prepared according to general procedure **2** using 2-benzyl-1,2,3,4-tetrahydroisoquinoline *N*-oxide (47.8 mg, 0.20 mmol, 1.0 equiv), pivaloyl chloride (62 µL, 0.50 mmol, 2.5 equiv), TBSOTf (23 µL, 0.10 mmol, 50 mol%), copper iodide (19.0 mg, 0.10 mmol, 50 mol%), 2-iodo-2-methylpropane (72 µL, 0.60 mmol, 3.0 equiv) and zinc dust (39.2 mg, 0.60 mmol, 3.0 equiv). Purification by automated flash column chromatography (RediSep Rf Gold Si 4g, 0–10% EtOAc in PE) delivered the title compound as an orange oil (14.6 mg, 26%, 0.05 mmol).

^{1}H NMR (400 MHz, $CDCl_3$) δ 7.49 – 7.39 (m, 2H), 7.33 (t, *J* = 7.7 Hz, 2H), 7.26 (dd, *J* = 5.4, 1.8 Hz, 1H), 7.21 – 7.06 (m, 4H), 4.03 (d, *J* = 13.8 Hz, 1H), 3.66 (d, *J* = 13.9 Hz, 1H), 3.46 (s, 1H), 3.21 (ddd, *J* = 10.9, 5.8, 4.1 Hz, 1H), 2.83 (ddd, *J* = 15.3, 10.0, 5.0 Hz, 1H), 2.60 (dt, *J* = 16.0, 4.6 Hz, 1H), 2.29 (ddd, *J* = 11.1, 9.9, 4.2 Hz, 1H), 0.97 (s, 9H).

^{13}C{H} NMR (176 MHz, $CDCl_3$) δ 140.6, 138.3, 136.4, 130.3, 128.4 (2C), 128.4 (2C), 127.8, 126.8, 126.1, 124.8, 72.4, 63.9, 48.5, 38.8, 28.6, 28.2 (3C).

HRMS: *m/z* calculated for $C_{20}H_{25}N$ $[M+H]^+$ 280.2060; found 280.2061.

IR (film, cm^{-1}): 2950, 2864, 1492, 1453, 1358, 1141, 1028, 746, 724, 697.

2-benzyl-1-ethyl-1,2,3,4-tetrahydroisoquinoline (14b):

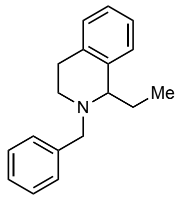

Prepared according to general procedure **2** using 2-benzyl-1,2,3,4-tetrahydroisoquinoline *N*-oxide (47.8 mg, 0.20 mmol, 1.0 equiv), pivaloyl chloride (62 µL, 0.50 mmol, 2.5 equiv), TBSOTf (35 µL, 0.10 mmol, 75 mol%), copper iodide (15.2 mg, 0.08 mmol, 40 mol%), 1-iodoethane (40 µL, 0.50 mmol, 2.5 equiv) and zinc dust (33.7 mg, 0.50 mmol, 2.5 equiv). Purification by automated flash column chromatography (RediSep Rf Gold Si 4g, 0–10% EtOAc in PE) delivered the title compound as a pale-yellow oil (30.5 mg, 61%, 0.12 mmol).

Spectra are in accordance with the literature data.[39]

2-benzyl-6-(tert-butyl)-1,2,3,4-tetrahydroisoquinoline (15a):

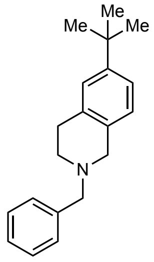

Prepared according to general procedure **2** using 2-benzyl-1,2,3,4-tetrahydroisoquinoline *N*-oxide (47.8 mg, 0.20 mmol, 1.0 equiv), pivaloyl chloride (62 µL, 0.50 mmol, 2.5 equiv), TBSOTf (23 µL, 0.10 mmol, 50 mol%), copper iodide (19.0 mg, 0.10 mmol, 50 mol%), 2-iodo-2-methylpropane (72 µL, 0.60 mmol, 3.0 equiv) and zinc dust (39.2 mg, 0.60 mmol, 3.0 equiv). Purification by automated flash column chromatography (RediSep Rf Gold Si 4g, 0–10% EtOAc in PE) delivered the title compound as an orange oil (10.5 mg, 19%, 0.04 mmol).

^{1}H NMR (700 MHz, $CDCl_3$) δ 7.40 (ddd, *J* = 7.5, 1.3, 0.7 Hz, 2H), 7.35 – 7.31 (m, 2H), 7.27 (d, *J* = 7.3 Hz, 1H), 7.16 – 7.09 (m, 2H), 6.93 (d, *J* = 8.0 Hz, 1H), 3.68 (s, 2H), 3.61 (s, 2H), 2.90 (t, *J* = 5.9 Hz, 2H), 2.75 (t, *J* = 5.9 Hz, 2H), 1.30 (s, 9H).

^{13}C{H} NMR (126 MHz, $CDCl_3$) δ 149.0, 138.8, 133.8, 132.1, 129.1 (2C), 128.3 (2C), 127.0, 126.3, 125.5, 122.7, 62.8, 55.9, 50.8, 34.3, 31.4, 29.4.

HRMS: *m/z* calculated for $C_{20}H_{25}N$ $[M+H]^+$ 280.2060; found 280.2061.

IR (film, cm^{-1}): 2959, 2922, 2796, 2754, 1673, 1453, 1364, 1266, 1113, 937, 819, 738, 698.

5.2 Grignard mediated α-alkylation scope

1-(4-phenylbenzyl)-2-methylpiperidine (8):

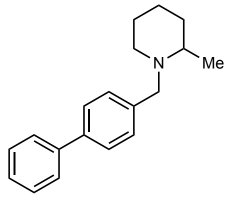

Prepared according to general procedure **4** using 1-(4-phenylbenzyl)piperidine *N*-oxide (53.5 mg, 0.20 mmol, 1.0 equiv), pivaloyl chloride (62 μL, 0.50 mmol, 2.5 equiv) and 3.4 M methyl magnesium bromide in 2-MeTHF (0.27 mL, 0.60 mmol, 3.0 equiv). Purification by SCX followed by automated flash column chromatography (RediSep Rf Gold Si 4 g, 0–50% Et_2O in PE) delivered the title compound as a colourless oil (25.3 mg, 48%, 0.10 mmol).

Spectra are in accordance with the literature data. [40]

1-(4-phenylbenzyl)-2-(methyl-d_3)piperidine (d_3-8):

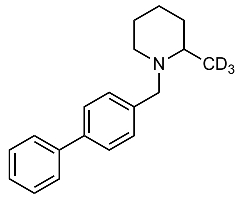

Prepared according to general procedure **4** using 1-(4-phenylbenzyl)piperidine *N*-oxide (53.5 mg, 0.20 mmol, 1.0 equiv), pivaloyl chloride (62 μL, 0.50 mmol, 2.5 equiv) and 1.0 M methyl-d_3 magnesium iodide in Et_2O (0.60 mL, 0.60 mmol, 3 equiv). Purification by reverse phase automated flash column chromatography (RediSep Rf 5.5 g C18-column, 0–100% MeCN in H_2O) delivered the title compound as a colourless oil (19.0 mg, 35%, 0.07 mmol).

^{1}H NMR (500 MHz, $CDCl_3$) δ 7.62 – 7.58 (m, 2H), 7.57 – 7.51 (m, 2H), 7.46 – 7.38 (m, 4H), 7.36 – 7.31 (m, 1H), 4.04 (d, *J* = 13.5 Hz, 1H), 3.25 (d, *J* = 13.5 Hz, 1H), 2.79 (dt, *J* = 11.6, 4.0 Hz, 1H), 2.32 (d, *J* = 9.0 Hz, 1H), 2.00 (td, *J* = 11.0, 3.4 Hz, 1H), 1.66 (ddd, *J* = 12.4, 5.5, 3.1 Hz, 2H), 1.58 – 1.44 (m, 2H), 1.42 – 1.25 (m, 2H).

^{13}C{H} NMR (126 MHz, $CDCl_3$) δ 141.3, 139.7, 138.8, 129.7 (2C), 128.8 (2C), 127.2 (3C), 126.9 (2C), 58.3, 56.4, 52.4, 34.8, 26.3, 24.2, 20.07 – 18.17 (m).

HRMS: *m/z* calculated for $C_{19}H_{20}ND_3$ $[M+H]^+$ 269.2092; found 269.2097.

IR (film, cm^{-1}): 3027, 2853, 2783, 2221, 1727, 1486, 1328, 1132, 1104, 1007, 842, 760, 696.

*1-(4-phenylbenzyl)-2-methyl-2-ethylpiperidine (**12**) and (2R,6S)-1-(4-phenylbenzyl)-2-ethyl-6-ethylpiperidine (**12'**):*

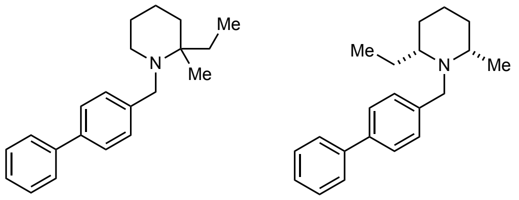

Prepared according to general procedure **4** using 1-(4-phenylbenzyl)-2-methylpiperidine *N*-oxide (56.2 mg, 0.20 mmol, 1.0 equiv), pivaloyl chloride (62 µL, 0.50 mmol, 2.5 equiv) and 3.0 M ethyl magnesium bromide in Et_2O (0.20 mL, 0.60 mmol, 3.0 equiv). Purification by automated flash column chromatography (RediSep Rf Gold Si 4 g, 0–20% EtOAc in PE) delivered the two regioisomers (total 49.7 mg, 85%, 0.17 mmol, 5.6:1 r.r).

<u>*Major regioisomer (42.2 mg, 72%, 0.14 mmol).*</u>

1-(4-phenylbenzyl)-2-methyl-2-ethylpiperidine (12):

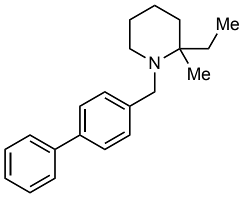

Obtained as a pale yellow oil.

^{1}H NMR (500 MHz, $CDCl_3$) δ 7.63 – 7.60 (m, 2H), 7.57 – 7.53 (m, 2H), 7.48 – 7.43 (m, 4H), 7.38 – 7.31 (m, 1H), 3.76 (d, *J* = 14.3 Hz, 1H), 3.37 (d, *J* = 14.2 Hz, 1H), 2.54 (dt, *J* = 11.9, 4.7 Hz, 1H), 2.35 (ddd, *J* = 12.2, 8.8, 3.7 Hz, 1H), 1.81 (dq, *J* = 14.8, 7.5 Hz, 1H), 1.71 (ddt, *J* = 12.3, 8.0, 4.5 Hz, 1H), 1.57 – 1.39 (m, 5H), 1.36 – 1.30 (m, 1H), 1.09 (s, 3H), 0.95 (t, *J* = 7.5 Hz, 3H).

^{13}C{H} NMR (126 MHz, $CDCl_3$) δ 141.4, 141.0, 139.4, 128.8 (2C), 128.8 (2C), 127.2 (2C), 127.1, 127.0 (2C), 55.7, 53.1, 46.9, 35.6, 30.1, 26.6, 21.2, 19.1, 8.2.

HRMS: *m/z* calculated for $C_{21}H_{27}N$ $[M+H]^+$ 294.2222; found 294.2229.

IR (film, cm^{-1}): 2963, 2931, 2861, 2795, 1486, 1461, 1368, 1126, 1085, 1007, 833, 757, 696.

Minor regioisomer (7.5 mg, 13%, 0.03 mmol, >20:1 dr).

(2R,6S)-1-(4-phenylbenzyl)-2-ethyl-6-ethylpiperidine (12'):

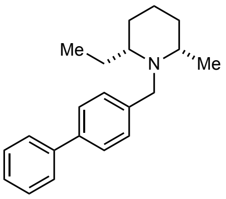

Obtained as a pale yellow oil and as a single diastereomer (>20:1 r.r).

^{1}H NMR (700 MHz, $CDCl_3$) δ 7.61 – 7.57 (m, 2H), 7.54 – 7.50 (m, 2H), 7.47 – 7.40 (m, 4H), 7.34 – 7.29 (m, 1H), 3.84 – 3.73 (m, 2H), 2.61 (s, 1H), 2.36 (t, *J* = 9.9 Hz, 1H), 1.75 – 1.64 (m, 3H), 1.56 – 1.52 (m, 1H), 1.39 – 1.32 (m, 2H), 1.30 – 1.24 (m, 2H), 1.05 (d, *J* = 6.3 Hz, 3H), 0.83 (t, *J* = 7.4 Hz, 3H).

^{13}C{H} NMR (176 MHz, $CDCl_3$) δ 142.2, 141.2, 138.9, 128.7 (2C), 128.1 (2C), 127.0 (2C), 126.9, 126.6 (2C), 64.3, 58.1, 52.7, 33.1, 29.2, 27.7, 24.1, 22.2, 10.9.

HRMS: *m/z* calculated for $C_{21}H_{27}N$ $[M+H]^+$ 294.2222; found 294.2229.

IR (film, cm^{-1}): 2960, 2926, 2853, 1487, 1373, 1189, 1007, 831, 754, 696.

The relative stereochemistry of the title compound was determined by 2D NMR experimentation. A clear NOE signal between the N-C**H** at 2.64 ppm and N-C**H** at 2.38 ppm was observed, thus these protons must be on the same face of the ring.

(*2R,4S*)-1-benzyl-2-ethyl-4-methylpiperidine (13'):

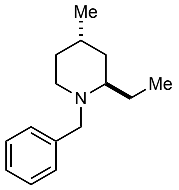

Prepared according to general procedure **4** using 1-benzyl-4-methylpiperidine *N*-oxide (41.0 mg, 0.20 mmol, 1.0 equiv), pivaloyl chloride (62 μL, 0.50 mmol, 2.5 equiv) and 3.0 M ethyl magnesium bromide in Et_2O (0.20 mL, 0.60 mmol, 3.0 equiv). Purification by automated flash column chromatography (RediSep Rf 4 g, 0–20% EtOAc in PE) followed by SCX delivered the title compound as a colourless oil and as a single diastereomer (18.1 mg, 42%, 0.08 mmol, >20:1 r.r)

^{1}H NMR (700 MHz, $CDCl_3$) δ 7.37 (d, *J* = 6.6 Hz, 2H), 7.32 – 7.28 (m, 2H), 7.26 – 7.23 (m, 1H), 3.82 (d, *J* = 13.3 Hz, 1H), 3.70 (d, *J* = 13.3 Hz, 1H), 2.74 (dq, *J* = 8.3, 4.0 Hz, 1H), 2.65 (dt, *J* = 12.4, 4.2 Hz, 1H), 2.59 (td, *J* = 11.9, 3.2 Hz, 1H), 1.72 – 1.65 (m, 2H), 1.61 (dtd, *J* = 13.5, 3.7, 1.9 Hz, 1H), 1.54 (ddt, *J* = 20.7, 9.9, 7.3 Hz, 2H), 1.40 (ddd, *J* = 13.5, 11.1, 4.5 Hz, 1H), 1.28 (ddd, *J* = 17.8, 13.3, 6.8 Hz, 1H), 0.91 (d, *J* = 6.5 Hz, 3H), 0.84 (t, *J* = 7.5 Hz, 3H).

^{13}C{H} NMR (176 MHz, $CDCl_3$) δ 138.3, 129.4 (2C), 128.4 (2C), 127.2, 58.5, 57.9, 46.1, 34.5, 32.3, 24.9, 22.0, 18.2, 11.3.

HRMS: *m/z* calculated for $C_{15}H_{23}N$ $[M+H]^+$ 218.1909; found 218.1916.

IR (film, cm^{-1}): 2948, 2921, 2870, 2796, 1493, 1453, 1363, 1122, 1067, 1028, 974, 754, 731, 696.

The relative stereochemistry of the title compound was attributed by analogy to compound **13**. See page 54.

2-benzyl-1-ethyl-1,2,3,4-tetrahydroisoquinoline (14c):

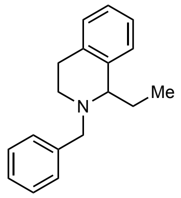

Prepared according to general procedure **4** using 2-benzyl-1,2,3,4-tetrahydroisoquinoline *N*-oxide (47.8 mg, 0.20 mmol, 1.0 equiv), pivaloyl chloride (62 µL, 0.50 mmol, 2.5 equiv) and 3.0 M ethyl magnesium bromide in Et_2O (0.20 mL, 0.60 mmol, 3 equiv). Purification by automated flash column chromatography (RediSep Rf Gold Si 4 g, 0–20% EtOAc in PE) delivered the title compound as a pale-yellow oil (40.0 mg, 80%, 0.16 mmol).

Spectra are in accordance with the literature.[39]

5.3 Trifluoromethylation scope

1-(4-phenylbenzyl)-2-(trifluoromethyl)piperidine (9a):

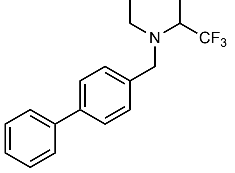

Prepared according to general procedure **5** using 1-(4-phenylbenzyl)piperidine *N*-oxide (53.5 mg, 0.20 mmol, 1.0 equiv), pivaloyl chloride (62 µL, 0.50 mmol, 2.5 equiv), MeCN

(2 mL, 0.1 M), DMF (47 μL, 0.60 mmol, 3.0 equiv), trimethyl(trifluoromethyl)silane (118 μL, 0.80 mmol, 4.0 equiv), potassium bifluoride (62.5 mg, 0.80 mmol, 4.0 equiv) and trifluoroacetic acid (61 μL, 0.80 mmol, 4.0 equiv). Purification by reverse phase automated flash column chromatography (RediSep Rf 5.5 g C18-column, 0-100% MeCN in H_2O) delivered the title compound as a colourless oil (40.0 mg, 55%, 0.11 mmol).

^{1}H NMR (500 MHz, $CDCl_3$) δ 7.63 – 7.58 (m, 2H), 7.58 – 7.53 (m, 2H), 7.47 – 7.40 (m, 4H), 7.37 – 7.31 (m, 1H), 3.96 (d, *J* = 14.1 Hz, 1H), 3.86 (d, *J* = 14.8 Hz, 1H), 3.26 (qt, *J* = 9.2, 4.7 Hz, 1H), 2.94 – 2.83 (m, 1H), 2.59 – 2.48 (m, 1H), 1.94 – 1.82 (m, 2H), 1.74 – 1.64 (m, 1H), 1.60 – 1.54 (m, 3H).

^{13}C{H} NMR (176 MHz, $CDCl_3$) δ 141.2, 140.1, 138.6, 128.9 (2C), 128.9 (2C), 128.1 (q, *J* = 291.8 Hz), 127.3, 127.2 (2C), 127.2 (2C), 59.3 (q, *J* = 24.6 Hz), 59.0 (q, *J* = 1.7 Hz), 47.3, 24.4, 24.4 (q, *J* = 1.9 Hz), 20.4.

^{19}F{H} NMR (471 MHz, $CDCl_3$) δ -64.8.

HRMS: *m/z* calculated for $C_{19}H_{21}NF_3$ $[M+H]^+$ 320.1621; found 320.1631.

IR (film, cm^{-1}): 2945, 2837, 1487, 1446, 1298, 1240, 1187, 1146, 1120, 1076, 997, 838, 759, 696.

1-(4-phenylbenzyl)-2-methyl-2-(trifluoromethyl)piperidine (9b):

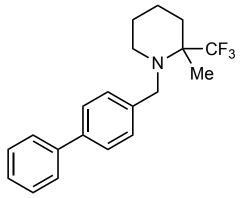

Prepared according to general procedure **5** using 1-(4-phenylbenzyl)-2-methylpiperidine *N*-oxide (56.2 mg, 0.20 mmol, 1.0 equiv), pivaloyl chloride (62 μL, 0.50 mmol, 2.5 equiv), MeCN (2 mL, 0.1 M), DMF (47 μL, 0.60 mmol, 3.0 equiv), trimethyl(trifluoromethyl)silane (118 μL, 0.80 mmol, 4.0 equiv), potassium bifluoride (62.5 mg, 0.80 mmol, 4.0 equiv) and trifluoroacetic acid (61 μL, 0.80 mmol, 4.0 equiv). Purification by reverse phase automated flash column chromatography (RediSep Rf 5.5 g C18-column, 0-100% MeCN in H_2O) followed by automated flash column chromatography (RediSep Rf 4 g, 0–5% EtOAc in PE) delivered the title compound as a colourless oil (43.0 mg, 65%, 0.13 mmol).

^{1}H NMR (500 MHz, $CDCl_3$) δ 7.60 (d, *J* = 6.9 Hz, 2H), 7.55 (d, *J* = 8.2 Hz, 2H), 7.47 – 7.37 (m, 4H), 7.34 (t, *J* = 7.3 Hz, 1H), 4.11 (d, *J* = 14.8 Hz, 1H), 3.61 (d, *J* = 14.9 Hz, 1H), 2.76 – 2.65 (m, 1H), 2.55 (d, *J* = 12.0 Hz, 1H), 2.10 – 1.96 (m, 1H), 1.69 – 1.48 (m, 5H), 1.42 (s, 3H).

^{13}C{H} NMR (126 MHz, $CDCl_3$) δ 141.3, 140.2 (q, *J* = 1.4 Hz), 139.8, 128.9 (2C),129.7 (q, *J* = 296.4 Hz), 128.3 (2C), 127.2, 127.2 (2C), 127.1 (2C), 59.3 (q, *J* = 21.8 Hz), 54.1 (q, *J* = 1.8 Hz), 46.7, 34.0 (d, *J* = 1.2 Hz), 25.7, 20.9, 20.6 (d, *J* = 1.2 Hz).

^{19}**F{H} NMR** (471 MHz, $CDCl_3$) δ -67.8.

HRMS: *m/z* calculated for $C_{20}H_{22}NF_3$ $[M+H]^+$ 334.1777; found 334.1783.

IR (film, cm^{-1}): 2947, 2838, 1487, 1381, 1250, 1123, 759, 697.

2-benzyl-1-(trifluoromethyl)-1,2,3,4-tetrahydroisoquinoline (14d):

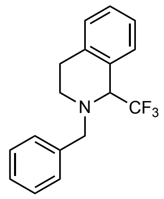

Prepared according to general procedure **5** using 2-benzyl-1,2,3,4-tetrahydroisoquinoline *N*-oxide (47.8 mg, 0.20 mmol, 1.0 equiv), pivaloyl chloride (62 μL, 0.50 mmol, 2.5 equiv), MeCN (2 mL, 0.1 M), DMF (47 μL, 0.60 mmol, 3.0 equiv), trimethyl(trifluoromethyl)silane (118 μL, 0.80 mmol, 4.0 equiv), potassium bifluoride (62.5 mg, 0.80 mmol, 4.0 equiv) and trifluoroacetic acid (61 μL, 0.80 mmol, 4.0 equiv). Purification by reverse phase automated flash column chromatography (RediSep Rf 5.5 g C18-column, 0-100% MeCN in H_2O) delivered the title compound as a colourless oil (45.6 mg, 78%, 0.16 mmol).

^{1}**H NMR** (700 MHz, $CDCl_3$) δ 7.41 (dd, *J* = 7.9, 1.1 Hz, 2H), 7.38 – 7.34 (m, 2H), 7.31 – 7.26 (m, 3H), 7.23 (t, *J* = 7.5 Hz, 1H), 7.19 (d, *J* = 7.5 Hz, 1H), 4.27 (q, *J* = 7.9 Hz, 1H), 4.05 (d, *J* = 13.7 Hz, 1H), 3.92 (d, *J* = 13.6 Hz, 1H), 3.28 (ddd, *J* = 12.0, 7.5, 4.6 Hz, 1H), 2.86 (ddd, *J* = 16.0, 7.5, 4.8 Hz, 1H), 2.83 – 2.77 (m, 1H), 2.75 – 2.66 (m, 1H).

^{13}**C{H} NMR** (176 MHz, $CDCl_3$) δ 138.7, 137.5, 129.5 (q, *J* = 1.8 Hz), 128.7, 128.6 (2C), 128.5 (d, *J* = 1.2 Hz), 128.4 (2C), 128.2, 127.4, 126.3 (d, *J* = 284.9 Hz), 126.0, 62.7 (q, *J* = 27.6 Hz), 60.6 (d, *J* = 1.2 Hz), 45.6 (q, *J* = 1.1 Hz), 26.9.

^{19}**F{H} NMR** (471 MHz, $CDCl_3$) δ -71.9.

HRMS: *m/z* calculated for $C_{17}H_{17}NF_3$ $[M+H]^+$ 292.1313; found 292.1323.

IR (film, cm^{-1}): 2917, 2812, 1494, 1454, 1371, 1331, 1253, 1149, 1110, 1009, 1026, 748, 697.

5.4 Azinylation scope

2-(1-benzylpiperidin-2-yl)pyridine (10a):

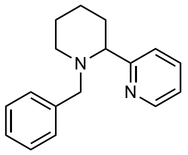

Prepared according to general procedure **6** using 1-benzylpiperidine *N*-oxide (38.2 mg, 0.20 mmol, 1.0 equiv), pivaloyl chloride (62 μL, 0.50 mmol, 2.5 equiv), TBSOTf (35 μL, 0.10 mmol, 75 mol%), copper iodide (19.0 mg, 0.10 mmol, 50 mol%), 2-iodopyridine (53 μL, 0.50 mmol, 2.5 equiv), zinc dust (19.6 mg, 0.30 mmol, 1.5 equiv) and indium powder (69.7 mg, 0.60 mmol, 3.0 equiv). Purification by automated flash column chromatography (RediSep Rf Gold Si 4g, 0–15% EtOAc in [3% Et_3N in PE]) delivered the title product as a colourless oil (31.3 mg, 62%, 0.12 mmol).

HRMS: *m/z* calculated for $C_{17}H_{21}N_2$ $[M+H]^+$ 253.1699; found 253.1701.

Spectra are in accordance with the literature data. [41]

2-(1-benzylpiperidin-2-yl)-3-bromopyridine (10b):

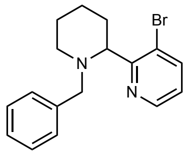

Prepared according to general procedure **6** using 1-benzylpiperidine *N*-oxide (38.2 mg, 0.20 mmol, 1.0 equiv), pivaloyl chloride (62 μL, 0.50 mmol, 2.5 equiv), TBSOTf (35 μL, 0.10 mmol, 75 mol%), copper iodide (19.0 mg, 0.10 mmol, 50 mol%), 3-bromo-2-iodopyridine (141.9 mg, 0.50 mmol, 2.5 equiv), zinc dust (19.6 mg, 0.30 mmol, 1.5 equiv), and indium powder (69.7 mg, 0.60 mmol, 3.0 equiv). Purification by automated flash column chromatography (RediSep Rf Gold Si 4g, 10–30% EtOAc in [3% Et_3N in PE]) delivered the title product as a colourless oil (31.8 mg, 48%, 0.10 mmol).

^{1}H NMR (500 MHz, $CDCl_3$) δ 8.67 (dd, *J* = 4.6, 1.6 Hz, 1H), 7.84 (dd, *J* = 8.0, 1.6 Hz, 1H), 7.27 – 7.22 (m, 2H), 7.21 – 7.17 (m, 3H), 7.04 (dd, *J* = 8.0, 4.5 Hz, 1H), 3.88 (dd, *J* = 7.8, 6.0 Hz, 1H), 3.57 (d, *J* = 13.1 Hz, 1H), 3.12 (d, *J* = 13.1 Hz, 1H), 3.08 – 2.99 (m, 1H), 2.10 (td, *J* = 11.7, 3.0 Hz, 1H), 1.83 – 1.59 (m, 5H), 1.44 – 1.33 (m, 1H).

^{13}C{H} NMR (126 MHz, $CDCl_3$) δ 160.8, 148.9, 140.7, 138.1, 129.8 (2C), 128.1 (2C), 126.9, 123.1, 121.3, 66.2, 60.0, 53.1, 32.4, 25.6, 24.7.

HRMS: *m/z* calculated for $C_{17}H_{20}N_2Br$ $[M+H]^+$ 331.0804; found 331.0806.

IR (film, cm^{-1}): 2931, 2852, 1571, 1494, 1426, 1369, 1129, 1101, 1066, 1011, 792, 761, 737, 698, 625.

2-(1-benzylpiperidin-2-yl)-3-methoxypyridine (10c):

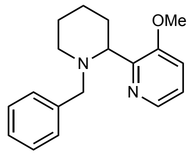

Prepared according to general procedure **6** using 1-benzylpiperidine *N*-oxide (38.2 mg, 0.20 mmol, 1.0 equiv), pivaloyl chloride (62 μL, 0.50 mmol, 2.5 equiv), TBSOTf (35 μL, 0.10 mmol, 75 mol%), copper iodide (19.0 mg, 0.10 mmol, 50 mol%), 2-iodo-3-methoxypyridine (118.0 mg, 0.50 mmol, 2.5 equiv), zinc dust (19.6 mg, 0.30 mmol, 1.5 equiv), and indium powder (69.7 mg, 0.60 mmol, 3.0 equiv). Purification by automated flash column chromatography (RediSep Rf Gold Si 4g, 5–20% EtOAc in [3% Et_3N in PE]) delivered the title product as a pale yellow oil (28.8 mg, 51%, 0.10 mmol).

^{1}H NMR (500 MHz, $CDCl_3$) δ 8.32 (dd, *J* = 4.0, 2.0 Hz, 1H), 7.24 – 7.11 (m, 7H), 3.85 – 3.80 (m, 4H), 3.59 (d, *J* = 13.1 Hz, 1H), 3.09 – 2.97 (m, 2H), 2.05 (td, *J* = 11.4, 2.9 Hz, 1H), 1.93 – 1.83 (m, 1H), 1.79 (dt, *J* = 12.8, 3.5 Hz, 1H), 1.73 – 1.64 (m, 2H), 1.61 – 1.54 (m, 1H), 1.37 (qt, *J* = 12.2, 3.5 Hz, 1H).

^{13}C{H} NMR (126 MHz, $CDCl_3$) δ 153.4, 152.7, 141.7, 138.8, 129.7 (2C), 127.9 (2C), 126.7, 122.2, 117.3, 61.1, 59.9, 55.4, 53.4, 32.5, 25.8, 24.9.

HRMS: *m/z* calculated for $C_{18}H_{23}N_2O$ $[M+H]^+$ 283.1805; found 283.1817.

IR (film, cm^{-1}): 2932, 1449, 1431, 1274, 1257, 1223, 1123, 797, 737, 699.

2-(1-benzylpiperidin-2-yl)-3-bromo-5-chloropyridine (10d):

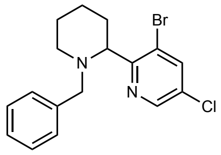

Prepared according to general procedure **6** using 1-benzylpiperidine *N*-oxide (38.2 mg, 0.20 mmol, 1.0 equiv), pivaloyl chloride (62 μL, 0.50 mmol, 2.5 equiv), TBSOTf (35 μL, 0.10 mmol, 75 mol%), copper iodide (19.0 mg, 0.10 mmol, 50 mol%), 3-bromo-5-chloro-2-iodopyridine (159.2 mg, 0.50 mmol, 2.5 equiv), zinc dust (19.6 mg, 0.30 mmol, 1.5 equiv), and indium powder (69.7 mg, 0.60 mmol, 3.0 equiv). Purification by automated flash column chromatography (RediSep Rf Gold Si 4g, 0–10% EtOAc in [3% Et_3N in PE]) delivered the title product as a colourless oil (43.2 mg, 59%, 0.12 mmol).

^{1}H NMR (400 MHz, $CDCl_3$) δ 8.62 (d, *J* = 2.2 Hz, 1H), 7.86 (d, *J* = 2.2 Hz, 1H), 7.27 – 7.16 (m, 5H), 3.88 – 3.82 (m, 1H), 3.55 (d, *J* = 13.1 Hz, 1H), 3.13 (d, *J* = 13.1 Hz, 1H), 3.04 (dt, *J* = 11.8, 3.6 Hz, 1H), 2.11 (td, *J* = 11.4, 3.7 Hz, 1H), 1.83 – 1.62 (m, 6H), 1.41 – 1.32 (m, 1H).

^{13}C{H} NMR (101 MHz, $CDCl_3$) δ 159.1, 147.8, 139.8, 130.0, 129.7 (2C), 128.1 (2C), 127.0, 120.8, 65.7, 60.1, 53.1, 32.5, 25.5, 24.6.

HRMS: *m/z* calculated for $C_{17}H_{19}N_2ClBr$ $[M+H]^+$ 365.0415; found 365.0413.

IR (film, cm^{-1}): 2931, 2853, 1560, 1493, 1428, 1373, 1250, 1127, 1108, 1030, 991, 891, 849, 797, 734, 697.

4-(1-benzylpiperidin-2-yl) furo[3,2-c]pyridine (10e):

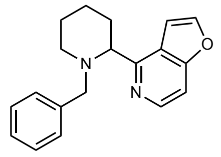

Prepared according to general procedure **6** using 1-benzylpiperidine *N*-oxide (38.2 mg, 0.20 mmol, 1.0 equiv), pivaloyl chloride (62 μL, 0.50 mmol, 2.5 equiv), TBSOTf (35 μL, 0.10 mmol, 75 mol%), copper iodide (19.0 mg, 0.10 mmol, 50 mol%), 4-iodofuro[3,2-*c*]pyridine (123.0 mg, 0.50 mmol, 2.5 equiv), zinc dust (19.6 mg, 0.30 mmol, 1.5 equiv), and indium powder (69.7 mg, 0.60 mmol, 3.0 equiv). Purification by automated flash column chromatography (RediSep Rf Gold Si 4g, 0–10% EtOAc in [3% Et_3N in PE]) delivered the title product as a colourless oil (22.2 mg, 38%, 0.08 mmol).

^{1}H NMR (400 MHz, $CDCl_3$) δ 8.41 (d, *J* = 5.8 Hz, 1H), 7.65 (d, *J* = 2.2 Hz, 1H), 7.45 (d, *J* = 1.2 Hz, 1H), 7.34 (dd, *J* = 5.8, 1.0 Hz, 1H), 7.25 – 7.15 (m, 5H), 3.64 – 3.52 (m, 2H), 3.07 – 2.95 (m, 2H), 2.02 – 1.92 (m, 1H), 1.91 – 1.80 (m, 3H), 1.71 – 1.61 (m, 2H), 1.51 – 1.39 (m, 1H).

^{13}C{H} NMR (101 MHz, $CDCl_3$) δ 160.4, 159.5, 144.5, 143.7, 139.0, 129.1 (2C), 128.1 (2C), 126.8, 122.1, 106.5, 106.3, 71.3, 60.8, 53.2, 33.3, 26.1, 24.9.

HRMS: *m/z* calculated for $C_{19}H_{21}N_2O$ $[M+H]^+$ 293.1648; found 293.1649.

IR (film, cm^{-1}): 2932, 2797, 1602, 1578, 1531, 1495, 1450, 1424, 1381, 1332, 1270, 1135, 1099, 1055, 1006, 847, 816, 794, 754, 698.

1-(1-benzylpiperidin-2-yl) isoquinoline (10f):

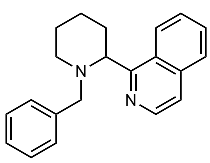

Prepared according to general procedure **6** using 1-benzylpiperidine *N*-oxide (38.2 mg, 0.20 mmol, 1.0 eqzuiv), pivaloyl chloride (62 μL, 0.50 mmol, 2.5 equiv), TBSOTf (35 μL, 0.10 mmol, 75 mol%), copper iodide (19.0 mg, 0.10 mmol, 50 mol%), 1-iodoisoquinoline (128.0 mg, 0.50 mmol, 2.5 equiv), zinc dust (19.6 mg, 0.30 mmol, 1.5 equiv), and indium powder (69.7 mg, 0.60 mmol, 3.0 equiv). Purification by automated flash column chromatography (RediSep Rf Gold Si 4g, 0–5% EtOAc in [3% Et_3N in PE]) delivered the title product as a colourless oil (26.0 mg, 43%, 0.09 mmol).

^{1}H NMR (400 MHz, $CDCl_3$) δ 8.49 (d, *J* = 5.6 Hz, 1H), 7.88 – 7.76 (m, 1H), 7.72 – 7.59 (m, 2H), 7.54 (d, *J* = 5.6 Hz, 1H), 7.30 – 7.09 (m, 5H), 3.99 (d, *J* = 11.4 Hz, 1H), 3.61 (d, *J* = 13.2 Hz, 1H), 3.15 – 2.96 (m, 2H), 2.27 – 1.63 (m, 7H), 1.49 (qt, *J* = 12.7, 4.2 Hz, 1H).

^{13}C{H} NMR (126 MHz, $CDCl_3$) δ 163.1, 142.0, 139.0, 137.1, 130.0, 129.3 (2C), 128.1 (2C), 127.4, 126.8, 126.7, 126.7, 126.5, 120.3, 72.3, 60.7, 53.5, 32.9, 25.9, 25.3.

HRMS: *m/z* calculated for $C_{21}H_{22}N_2$ $[M+H]^+$ 303.1856; found 303.1857.

IR (film, cm^{-1}): 3050, 2932, 2853, 2799, 1585, 1560, 1495, 1451, 1346, 1105, 826, 798, 744, 698, 678, 644.

5.5 Late-stage α-derivatisation scope

*(4bS,8aS,9S)-3-methoxy-11-methyl-6,7,8,8a,9,10-hexahydro-5H-11λ^4-9,4b-(aminediylethan[2]yl[1]ylidene)phenanthrene chloride (**16**):*

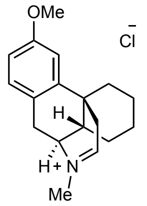

Prepared according to general procedure **1** using dextromethorphan *N*-oxide (57.4 mg, 0.20 mmol, 1.0 equiv) and pivaloyl chloride (163 μL, 1.32 mmol, 6.6 equiv). After warming to rt, 1,1,2,2-tetrachloroethane (21 μL, 0.20 mmol, 1.0 equiv) was added as an internal standard and the yield was determined *via* quantitative ^{1}H NMR spectroscopy (60%, >20:1 r.r.).

^{1}H NMR (700 MHz, CD2Cl2) δ 9.06 (br s, 1H), 7.10 (d, *J* = 8.5 Hz, 1H), 6.87 (d, *J* = 2.6 Hz, 1H), 6.79 (dd, *J* = 8.5, 2.6 Hz, 1H), 4.17 (d, *J* = 4.4 Hz, 1H), 3.88 (s, 3H), 3.77 (s, 3H), 3.26 (dd, *J* = 18.0, 5.1 Hz, 1H), 3.19 (d, *J* = 21.3 Hz, 1H), 3.06 (d, *J* = 18.2 Hz, 1H), 2.96 (d, *J* = 20.8 Hz, 1H), 2.58 (dt, *J* = 14.2, 3.5 Hz, 1H), 2.32 (dt, *J* = 12.5, 3.7 Hz, 1H), 1.71 (t, *J* = 12.2 Hz, 2H), 1.59 (dd, *J* = 13.7, 4.6 Hz, 1H), 1.51 – 1.42 (m, 2H), 1.17 – 1.09 (m, 2H).

^{13}C{H} NMR (176 MHz, CD2Cl2) δ 179.3, 160.1, 140.0, 131.8, 122.0, 113.6, 111.2, 62.5, 55.7, 48.1, 47.8, 38.7, 35.6, 33.6, 29.2, 25.9, 25.4, 22.0.

HRMS & IR: *compound unstable.*

(4bS,8aS,9S,12R)-3-methoxy-11,12-dimethyl-6,7,8,8a,9,10-hexahydro-5H-9,4b-(epiminoethano)phenanthrene (17a):

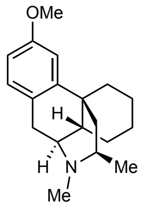

Prepared according to general procedure **4** using dextromethorphan *N*-oxide (57.4 mg, 0.20 mmol, 1.0 equiv), pivaloyl chloride (62 µL, 0.50 mmol, 2.5 equiv) and 3.4 M methyl magnesium bromide in 2-MeTHF (0.27 mL, 0.60 mmol, 3.0 equiv). Purification by reverse phase automated flash column chromatography (RediSep Rf 5.5 g C18-column, 0–100% MeCN in H_2O delivered the title compound as an orange oil and as a single diastereomer (23.9 mg, 42%, 0.08 mmol, d.r. >20:1).

^{1}H NMR (500 MHz, $CDCl_3$) δ 6.94 (d, *J* = 8.4 Hz, 1H), 6.85 (d, *J* = 2.7 Hz, 1H), 6.68 (dd, *J* = 8.3, 2.7 Hz, 1H), 3.78 (s, 3H), 3.09 (pd, *J* = 7.1, 2.2 Hz, 1H), 2.88 – 2.82 (m, 2H), 2.70 (dd, *J* = 17.6, 5.6 Hz, 1H), 2.49 (s, 3H), 2.38 – 2.30 (m, 1H), 1.91 (dd, *J* = 13.2, 7.1 Hz, 1H), 1.80 (dt, *J* = 12.6, 3.4 Hz, 1H), 1.64 – 1.57 (m, 1H), 1.52 (dd, *J* = 13.2, 2.2 Hz, 1H), 1.48 – 1.37 (m, 2H), 1.35 – 1.19 (m, 3H), 1.17 – 1.04 (m, 1H), 0.39 (d, *J* = 7.1 Hz, 3H).

^{13}C{H} NMR (126 MHz, $CDCl_3$) δ 158.3, 143.9, 129.9, 129.2, 111.6, 110.9, 57.6, 55.3, 51.8, 48.7, 44.3, 40.3, 38.0, 36.0, 27.1, 26.7, 26.6, 22.0, 16.7.

HRMS: *m/z* calculated for $C_{19}H_{27}NO$ $[M+H]^+$ 286.2165; found 286.2157.

IR (film, cm^{-1}): 2924, 2853, 1608, 1575, 1497, 1452, 1265, 1240, 1157, 1068, 1041, 866, 797.

The relative stereochemistry of the title compound was determined by 2D NMR experimentation. NOE signals between the methyl group at 0.4 ppm and two aromatic protons can be observed. This is only possible if the methyl group points over the ring.

***(4bS,8aS,9S)-12-isopropyl-3-methoxy-11-methyl-6,7,8,8a,9,10-hexahydro-5H-9,4b-(epiminoethano)phenanthrene* (17b):**

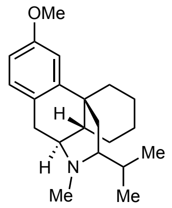

Prepared according to general procedure **2** using dextromethorphan *N*-oxide (57.4 mg, 0.20 mmol, 1.0 equiv), pivaloyl chloride (62 μL, 0.50 mmol, 2.5 equiv), TBSOTf (34 μL, 0.15 mmol, 75 mol%), copper iodide (9.5 mg, 0.05 mmol, 25 mol%), 2-iodopropane (50 μL, 0.50 mmol, 2.5 equiv) and zinc dust (19.6 mg, 0.30 mmol, 1.5 equiv). Purification by reverse phase automated flash column chromatography (RediSep Rf 5.5 g C18-column, 0–100% MeCN in H_2O) delivered the title compound as pink oil (36.7 mg, 59%, 0.12 mmol, d.r. >20:1).

^{1}H NMR (700 MHz, $CDCl_3$) δ 6.90 (d, *J* = 8.3 Hz, 1H), 6.83 (d, *J* = 2.7 Hz, 1H), 6.69 (dd, *J* = 8.3, 2.7 Hz, 1H), 3.78 (s, 3H), 3.06 – 2.97 (m, 1H), 2.77 – 2.66 (m, 2H), 2.59 (s, 3H), 2.31 (dd, *J* = 12.3, 1.8 Hz, 1H), 2.09 (ddd, *J* = 10.0, 7.1, 3.3 Hz, 1H), 1.89 (dt, *J* = 12.6, 3.5 Hz, 1H), 1.72 – 1.58 (m, 3H), 1.48 – 1.43 (m, 1H), 1.35 – 1.17 (m, 4H), 1.14 – 1.01 (m, 2H), 0.78 (d, *J* = 6.6 Hz, 3H), 0.30 (d, *J* = 6.6 Hz, 3H).

^{13}C{H} NMR (176 MHz, $CDCl_3$) δ 158.1, 143.4, 129.4, 128.6, 111.5, 111.1, 64.2, 59.0, 55.4, 46.2, 38.6, 38.0, 37.7, 36.5, 33.6, 30.7, 27.1, 26.6, 22.3, 22.0, 19.4.

HRMS: *m/z* calculated for $C_{21}H_{31}NO$ $[M+H]^+$ 314.2478; found 314.2470.

IR (film, cm^{-1}): 2926, 2855, 1667, 1608, 1575, 1499, 1268, 1238, 1042, 850, 804, 754.

The 2D NMR data did not provide definitive evidence to unambiguously determine the diastereomer formed.

*((4bS,8aS,9S)-3-methoxy-11-methyl-6,7,8,8a,9,10-hexahydro-5H-9,4b-(epiminoethano)phenanthren-12-yl)methanol (**17c & 17c'**):*

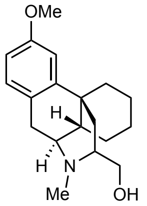

Prepared according to general procedure **2** using dextromethorphan *N*-oxide (57.4 mg, 0.20 mmol, 1.0 equiv), pivaloyl chloride (62 μL, 0.50 mmol, 2.5 equiv), TBSOTf (35 μL, 0.15 mmol, 75 mol%), copper iodide (15.2 mg, 0.08 mmol, 40 mol%), iodomethyl benzoate

(131.0 mg, 0.50 mmol, 2.5 equiv) and zinc dust (33.2 mg, 0.50 mmol, 2.5 equiv). After concentrating *in vacuo*, the crude α-alkylated cyclic alkylamine was dissolved in MeOH/THF (12 mL, 5:1) and K_2CO_3 (82.9 mg, 0.60 mmol, 3.0 equiv) was added. The mixture was stirred for 24 h and then quenched with saturated ammonium chloride solution (20 mL) for 30 mins. The solution was diluted with water and the aqueous layer was washed several times with EtOAc (6 x 20 mL). The combined organic layers were dried over $MgSO_4$, filtered, and concentrated *in vacuo*. Purification by reverse phase automated flash column chromatography (RediSep Rf 5.5 g C18-column, 0–100% MeCN in H_2O) delivered two diastereomers of the title compound (total 29.9 mg, 50%, 0.10 mmol, d.r. 1.5:1).

<u>*Major diastereomer (18.1 mg, 30%, 0.06 mmol).*</u>

***((4bS,8aS,9S,12R)-3-methoxy-11-methyl-6,7,8,8a,9,10-hexahydro-5H-9,4b-(epiminoethano)phenanthren-12-yl)methanol* (17c):**

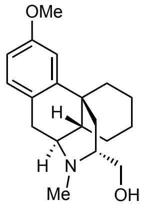

Obtained as a pale yellow oil.

^{1}H NMR (700 MHz, $CDCl_3$) δ 6.95 (d, *J* = 8.4 Hz, 1H), 6.79 (d, *J* = 2.6 Hz, 1H), 6.70 (dd, *J* = 8.4, 2.7 Hz, 1H), 3.77 (s, 3H), 3.04 (dd, *J* = 17.9, 6.7 Hz, 1H), 2.85 – 2.78 (m, 2H), 2.76 (d, *J* = 17.9 Hz, 1H), 2.69 (s, 3H), 2.61 (d, *J* = 7.0 Hz, 2H), 2.32 (d, *J* = 13.9 Hz, 1H), 1.93 – 1.85 (m, 2H), 1.67 – 1.62 (m, 1H), 1.50 – 1.44 (m, 1H), 1.41 – 1.32 (m, 3H), 1.30 – 1.18 (m, 2H), 1.08 (qd, *J* = 13.4, 4.2 Hz, 1H).

^{13}C{H} NMR (176 MHz, $CDCl_3$) δ 158.5, 142.5, 129.7, 128.6, 111.5, 111.4, 62.6, 59.1, 57.8, 55.3, 45.7, 40.5, 39.1, 38.3, 36.2, 32.0, 26.9, 26.6, 22.1.

HRMS: *m/z* calculated for $C_{19}H_{27}NO_2$ $[M+H]^+$ 302.2115; found 302.2111.

IR (film, cm^{-1}): 3379, 2925, 2853, 1608, 1499, 1463, 1452, 1266, 1240, 1040, 866, 809, 752.

The relative stereochemistry of the title compound was determined by computational modelling due to weak NOE correlations. DP4-AI modelling developed by the Goodman group was employed to predict the proton and carbon chemical shifts of each isomer and match this with the experimental data.[42]

These results show the proton and carbon chemical shifts match the predicted (*R*)-isomer.

Results of DP4 using proton chemical shifts:

Computed **17c'**: 26.7%
Computed **17c**: 73.3%

Results of DP4 using carbon chemical shifts:
Computed **17c'**: 1.5%
Computed **17c**: 98.5%

Results of DP4:
Computed **17c'**: 0.6%
Computed **17c**: 99.4%

<u>*Minor diastereomer (11.8 mg, 20%, 0.04 mmol).*</u>

((4bS,8aS,9S,12S)-3-methoxy-11-methyl-6,7,8,8a,9,10-hexahydro-5H-9,4b-(epiminoethano)phenanthren-12-yl)methanol **(17c')**:

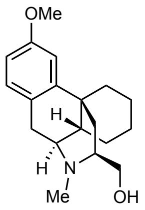

^{1}H NMR (700 MHz, $CDCl_3$) δ 7.02 (d, *J* = 8.4 Hz, 1H), 6.80 (d, *J* = 2.9 Hz, 1H), 6.76 (dd, *J* = 8.7, 2.2 Hz, 1H), 3.78 (s, 3H), 3.39 (d, *J* = 19.1 Hz, 1H), 3.35 – 3.30 (m, 1H), 3.30 – 3.23 (m, 1H), 3.21 – 3.13 (m, 2H), 3.97 (s, 3H), 2.64 (d, *J* = 16.0 Hz, 1H), 2.37 (d, *J* = 13.9 Hz, 1H), 2.06 (dd, *J* = 14.4, 8.0 Hz, 1H), 2.01 – 1.96 (m, 1H), 1.65 (dd, *J* = 20.3, 14.3 Hz, 2H), 1.53 (d, *J* = 13.2 Hz, 1H), 1.44 – 1.34 (m, 2H), 1.30 (td, *J* = 13.6, 3.3 Hz, 1H), 1.24 – 1.18 (m, 1H), 1.14 (td, *J* = 12.6, 4.2 Hz, 1H).

^{13}C{H} NMR (126 MHz, $CDCl_3$) δ 159.0, 140.5, 129.9, 126.3, 112.3, 111.6, 64.4, 63.3, 60.1, 55.4, 44.7, 41.0, 38.3, 37.2, 35.4, 29.7, 26.3, 25.8, 21.7.

HRMS: *m/z* calculated for $C_{19}H_{27}NO_2$ $[M+H]^+$ 302.2115; found 302.2111.

IR (film, cm^{-1}): 3358, 2931, 2857, 1609, 1558, 1501, 1464, 1380, 1270, 1241, 1065, 1040, 865, 809, 753, 722.

The relative stereochemistry of the title compound was determined by computational modelling due to weak NOE correlations. DP4-AI modelling developed by the Goodman group was employed to predict the proton and carbon chemical shifts of each isomer and match this with the experimental data.[42]

These results show the proton and carbon chemical shifts match the predicted (*S*)-isomer.

Results of DP4 using proton chemical shifts:
Computed **17c'**: 100.0%
Computed **17c**: 0.0%

Results of DP4 using carbon chemical shifts:
Computed **17c'**: 99.1%
Computed **17c**: 0.9%

Results of DP4:
Computed **17c'**: 100.0%
Computed **17c**: 0.0%

(4bS,8aS,9S)-12-(fluoromethyl)-3-methoxy-11-methyl-6,7,8,8a,9,10-hexahydro-5H-9,4b-(epiminoethano)phenanthrene **(17d):**

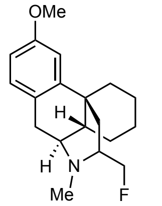

Prepared according to general procedure **2** dextromethorphan *N*-oxide (57.4 mg, 0.20 mmol, 1.0 equiv), pivaloyl chloride (62 μL, 0.50 mmol, 2.5 equiv), TBSOTf (34 μL, 0.15 mmol, 75 mol%), copper iodide (15.2 mg, 0.08 mmol, 40 mol%), fluoroiodomethane (35 μL, 0.50 mmol, 2.5 equiv) and zinc dust (33.2 mg, 0.50 mmol, 2.5 equiv). Purification by reverse phase automated flash column chromatography (RediSep Rf 5.5 g C18-column, 0–100% MeCN in H_2O) followed by automated flash column chromatography (RediSep Rf Gold 4g, 0–40% Et_2O acetate in PE) delivered the title compound as a colourless oil (25.4 mg, 42%, 0.08 mmol, d.r. >20:1).

^{1}H NMR (700 MHz, $CDCl_3$) δ 6.97 (d, *J* = 8.4 Hz, 1H), 6.82 (d, *J* = 2.7 Hz, 1H), 6.72 (dd, *J* = 8.3, 2.6 Hz, 1H), 4.05 – 4.07 – 3.91 (m, 1H), 3.76 (s, 3H), 3.23 – 3.10 (m, 2H), 2.89 – 2.84 (m, 2H), 2.79 – 2.72 (m, 1H), 2.62 (d, *J* = 1.9 Hz, 3H), 2.31 (dq, *J* = 13.5, 2.7 Hz, 1H), 1.97 – 1.91 (m, 1H,), 1.78 (dt, *J* = 12.7, 3.4 Hz, 1H), 1.68 – 1.60 (m, 2H), 1.51 – 1.45 (m, 1H), 1.41 (dd, *J* = 12.9, 3.2 Hz, 1H), 1.34 (qt, *J* = 12.8, 3.7 Hz, 1H), 1.27 (td, *J* = 13.6, 3.2 Hz, 1H), 1.18 (qt, *J* = 13.2, 3.1 Hz, 1H), 1.09 (qd, *J* = 12.8, 3.9 Hz, 1H).

^{13}C{H} NMR (176 MHz, $CDCl_3$) δ 158.6, 142.4, 130.2, 128.2, 111.7, 111.6, 85.5 (d, *J* = 165.8 Hz), 58.5 (d, *J* = 21.5 Hz), 57.2, 55.3, 45.1 (d, *J* = 5.2 Hz), 44.0, 41.2 (d, *J* = 1.2 Hz), 37.4, 35.9, 27.3, 26.7, 26.6, 22.2.

^{19}F{H} NMR (376 MHz, $CDCl_3$) δ -207.4.

HRMS: *m/z* calculated for $C_{19}H_{26}FNO$ $[M+H]^+$ 304.2071; found 304.2065.

IR (film, cm^{-1}): 2924, 2853, 1608, 1499, 1452, 1289, 1266, 1240, 1156, 1120, 1059, 1041, 1002, 982, 866, 809.

The 2D NMR data did not provide definitive evidence to unambiguously determine the diastereomer formed.

*(4bS,8aS,9S)-3-methoxy-11-methyl-12-(pyridin-2-yl)-6,7,8,8a,9,10-hexahydro-5H-9,4b-(epiminoethano)phenanthrene (**17e & 17e'**):*

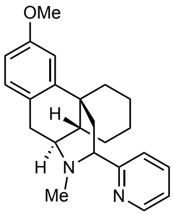

Prepared according to general procedure **6** using dextromethorphan *N*-oxide (57.4 mg, 0.20 mmol, 1.0 equiv), pivaloyl chloride (62 μL, 0.50 mmol, 2.5 equiv), TBSOTf (35 μL, 0.10 mmol, 75 mol%), copper iodide (19.0 mg, 0.10 mmol, 50 mol%), 2-iodopyridine (53 µL, 0.50 mmol, 2.5 equiv), zinc dust (19.6 mg, 0.30 mmol, 1.5 equiv), and indium powder (69.7 mg, 0.60 mmol, 3.0 equiv). Purification by reverse phase automated flash column chromatography (RediSep Rf 5.5 g C18-column, 0–100% MeCN in H_2O) delivered two diastereomers of the title compound (total 19.3 mg, 28%, 0.06 mmol, d.r. 1.6:1).

Major diastereomer (11.6 mg, 17%, 0.03 mmol).

***(4bS,8aS,9S, 12R)-3-methoxy-11-methyl-12-(pyridin-2-yl)-6,7,8,8a,9,10-hexahydro-5H-9,4b-(epiminoethano)phenanthrene* (17e):**

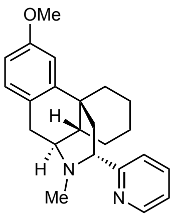

Obtained as a yellow oil.

^{1}H NMR (500 MHz, $CDCl_3$) δ 8.46 (ddd, *J* = 4.9, 1.8, 0.9 Hz, 1H), 7.57 (td, *J* = 7.7, 1.8 Hz, 1H), 7.26 (d, *J* = 7.7 Hz, 1H), 7.11 – 7.02 (m, 2H), 6.80 (d, *J* = 2.7 Hz, 1H), 6.71 (dd, *J* = 8.4, 2.6 Hz, 1H), 3.78 (s, 3H), 3.27 (dd, *J* = 11.6, 3.5 Hz, 1H), 3.15 (d, *J* = 18.2 Hz, 1H), 3.02 (dd, *J* = 5.3, 3.4 Hz, 1H), 2.66 (dd, *J* = 17.8, 6.5 Hz, 1H), 2.34 – 2.28 (m, 1H), 2.14 (s, 3H), 2.08 (dt, *J* = 12.8, 3.4 Hz, 1H), 1.74 (d, *J* = 11.5 Hz, 1H), 1.69 – 1.64 (m, 1H), 1.57 (dd, *J* = 12.7, 3.6 Hz, 1H), 1.54 – 1.46 (m, 2H), 1.43 – 1.26 (m, 3H), 1.20 (qd, *J* = 12.7, 3.8 Hz, 1H).

^{13}C{H} NMR (126 MHz, $CDCl_3$) δ 164.0, 158.4, 149.3, 142.0, 136.5, 129.5, 129.1, 122.2, 122.0, 111.3, 111.1, 63.7, 59.6, 55.3, 51.4, 45.6, 40.7, 38.3, 36.7, 26.8, 26.8, 24.2, 22.4.

HRMS: *m/z* calculated for $C_{23}H_{28}N_2O$ $[M+H]^+$ 349.2274; found 349.2268.

IR (film, cm^{-1}): 2926, 2853, 1608, 1589, 1495, 1464, 1432, 1236, 1070, 1044, 906, 795, 729.

The relative stereochemistry of the title compound was determined by 2D NMR experimentation. NOE correlations were observed between a benzylic C**H₂** proton adjacent to the aromatic ring and the benzylic C**H** proton adjacent to pyridine. This is only possible if the benzylic CH proton adjacent to pyridine points over the ring.

<u>*Minor diastereomer (7.7 mg, 11%, 0.02 mmol).*</u>

(4bS,8aS,9S,12S)-3-methoxy-11-methyl-12-(pyridin-2-yl)-6,7,8,8a,9,10-hexahydro-5H-9,4b-(epiminoethano)phenanthrene **(17e'):**

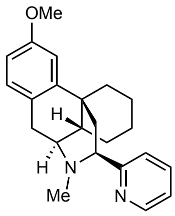

Further purification by automated flash column chromatography (RediSep Rf Gold Si 4g, 0–20% EtOAc in PE) delivered the title compound as the minor diastereomer as a yellow oil.

^{1}H NMR (700 MHz, $CDCl_3$) δ 8.29 – 8.22 (m, 1H), 7.40 (ddd, *J* = 9.6, 6.8, 1.9 Hz, 1H), 7.32 – 7.27 (m, 1H), 6.90 (ddd, *J* = 7.4, 4.9, 1.3 Hz, 1H), 6.79 (d, *J* = 2.6 Hz, 1H), 6.73 (d, *J* = 8.4 Hz, 1H), 6.53 (d, *J* = 8.8 Hz, 1H), 3.90 (s, 1H), 3.75 (s, 3H), 2.92 – 2.79 (m, 2H), 2.72 (d, *J* = 17.1 Hz, 1H), 2.43 (d, *J* = 14.2 Hz, 4H), 2.08 (dd, *J* = 37.8, 10.4 Hz, 2H), 2.02 – 1.95 (m, 1H), 1.65 (d, *J* = 13.1 Hz, 1H), 1.49 (d, *J* = 13.9 Hz, 1H), 1.45 – 1.31 (m, 3H), 1.18 (dt, *J* = 13.3, 3.4 Hz, 1H), 1.09 (qd, *J* = 12.7, 3.9 Hz, 1H).

^{13}C{H} NMR (176 MHz, $CDCl_3$) δ 164.8, 158.2, 148.0, 144.6, 135.9, 130.4, 127.6, 121.6, 121.0, 111.1, 110.8, 62.3, 59.8, 55.4, 46.9, 44.6, 38.4, 37.7, 36.4, 33.1, 26.7, 22.6.

HRMS: *m/z* calculated for $C_{23}H_{28}N_2O$ $[M+H]^+$ 349.2274; found 349.2269.

IR (film, cm^{-1}): 2927, 2852, 1608, 1588, 1500, 1431, 1291, 1267, 1236, 1043, 911, 860, 794, 751, 729.

The relative stereochemistry of the title compound was determined by 2D NMR experimentation. An NOE correlation was observed between the benzylic C**H** proton

adjacent to pyridine and the N-CH-C**H** proton. This is only possible if the pyridine points over the ring.

*(4bS,8aS,9S)-3-methoxy-11-methyl-12-(trifluoromethyl)-6,7,8,8a,9,10-hexahydro-5H-9,4b-(epiminoethano)phenanthrene (**17f & 17f'**):*

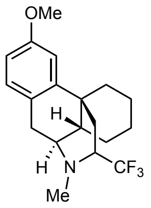

Prepared according to general procedure **5** using dextromethorphan *N*-oxide (57.4 mg, 0.20 mmol, 1.0 equiv), pivaloyl chloride (62 μL, 0.50 mmol, 2.5 equiv), MeCN (2 mL, 0.1 M), DMF (47 μL, 0.60 mmol, 3.0 equiv), trimethyl(trifluoromethyl)silane (118 μL, 0.80 mmol, 4.0 equiv), potassium bifluoride (62.5 mg, 0.80 mmol, 4.0 equiv) and trifluoroacetic acid (61 μL, 0.80 mmol, 4.0 equiv). Purification by reverse phase automated flash column chromatography (RediSep Rf 5.5 g C18-column, 0–100% MeCN in H_2O) followed by automated flash column chromatography (RediSep Rf Gold Si 4 g, 0–10% Et_2O in PE) delivered two diastereomers of the title compound (total 25.9 mg, 38%, 0.08 mmol, d.r. 4:1).

Major diastereomer (20.7 mg, 30%, 0.06 mmol).

***(4bS,8aS,9S,12R)-3-methoxy-11-methyl-12-(trifluoromethyl)-6,7,8,8a,9,10-hexahydro-5H-9,4b-(epiminoethano)phenanthrene* (17f):**

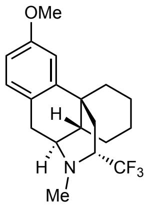

Obtained as a colourless oil.

^{1}H NMR (700 MHz, $CDCl_3$) δ 7.04 (d, *J* = 8.4 Hz, 1H), 6.81 (d, *J* = 2.6 Hz, 1H), 6.73 (dd, *J* = 8.4, 2.6 Hz, 1H), 3.80 (s, 3H), 3.02 (d, *J* = 18.2 Hz, 1H), 2.96 – 2.88 (m, 1H), 2.74 – 2.62 (m, 2H), 2.50 (d, *J* = 1.9 Hz, 3H), 2.37 (d, *J* = 13.7 Hz, 1H), 1.90 (dt, *J* = 12.6, 3.4 Hz, 1H), 1.68 – 1.61 (m, 3H), 1.58 – 1.51 (m, 1H), 1.46 – 1.41 (m, 1H), 1.41 – 1.31 (m, 2H), 1.31 – 1.23 (m, 1H), 1.11 (qd, *J* = 12.8, 3.9 Hz, 1H).

^{13}C{H} NMR (176 MHz, $CDCl_3$) δ 158.7, 140.8, 129.2, 128.5, 126.4 (q, *J* = 282.3 Hz), 111.6, 111.3, 61.2, 58.1 (q, *J* = 26.1 Hz), 55.4, 44.6, 42.1 (q, *J* = 2.5 Hz), 41.4 (q, *J* = 2.3 Hz), 37.0, 36.5, 26.5, 26.3, 24.8, 22.2.

^{19}F{H} NMR (471 MHz, $CDCl_3$) δ -69.8.

HRMS: *m/z* calculated for $C_{19}H_{24}NOF_3$ $[M+H]^+$ 340.1883; found 340.1887.

IR (film, cm^{-1}): 2928, 2856, 1687, 1609, 1496, 1454, 1362, 1268, 1239, 1226, 1151, 1127, 1109, 1043, 970, 907, 753.

The relative stereochemistry of the title compound was determined by 2D NMR experimentation. No NOE correlation was observed between C**H**-CF_3 and the N-CH-C**H**. This is only possible if the C**H**-CF_3 proton points over the aromatic ring.

Minor diastereomer (*5.2 mg, 8%, 0.02 mmol).*

(4bS,8aS,9S,12S)-3-methoxy-11-methyl-12-(trifluoromethyl)-6,7,8,8a,9,10-hexahydro-5H-9,4b-(epiminoethano)phenanthrene (17f')

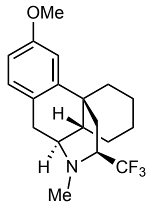

Obtained as a colourless oil.

^{1}H NMR (700 MHz, $CDCl_3$) δ 6.96 (d, *J* = 8.4 Hz, 1H), 6.81 (d, *J* = 2.6 Hz, 1H), 6.71 (dd, *J* = 8.4, 2.6 Hz, 1H), 3.78 (s, 3H), 3.34 (qdd, *J* = 10.0, 7.7, 4.4 Hz, 1H), 2.82 (d, *J* = 1.8 Hz, 3H), 2.66 (q, *J* = 1.5 Hz, 3H), 2.42 (d, *J* = 14.3 Hz, 1H), 1.95 – 1.84 (m, 2H), 1.75 (dt, *J* = 12.6, 3.4 Hz, 1H), 1.63 (d, *J* = 12.9 Hz, 1H), 1.51 – 1.46 (m, 1H), 1.44 – 1.39 (m, 1H), 1.32 (qt, *J* = 13.0, 3.7 Hz, 1H), 1.26 (td, *J* = 13.5, 3.2 Hz, 1H), 1.17 (qt, *J* = 13.2, 3.2 Hz, 1H), 1.09 (qd, *J* = 12.9, 4.0 Hz, 1H).

^{13}C{H} NMR (176 MHz, $CDCl_3$) δ 158.3, 141.3, 130.3, 128.5, 126.4 (q, *J* = 287.6 Hz), 111.4 (d, *J* = 2.0 Hz), 111.3 (d, *J* = 1.7 Hz), 58.4 (d, *J* = 27.5 Hz), 58.1, 55.4, 43.6, 41.5, 40.4, 38.6, 34.7, 30.7, 26.6, 26.5, 22.3.

^{19}F{H} NMR (471 MHz, $CDCl_3$) δ -68.5.

HRMS: *m/z* calculated for $C_{19}H_{24}NOF_3$ $[M+H]^+$ 340.1883; found 340.1886.

IR (film, cm^{-1}): 2929, 2856, 1610, 1577, 1453, 1293, 1269, 1236, 1143, 1110, 1043, 985, 882.

The relative stereochemistry of the title compound was determined by 2D NMR experimentation. A clear NOE signal was observed between C**H**-CF_3 and the N-CH-C**H**. This is only possible if the C**H**-CF_3 proton points over the aliphatic ring.

1-(2-((4-chlorophenyl)(phenyl)methoxy)ethyl)-2-(trifluoromethyl)piperidine (18):

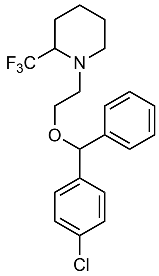

Prepared according to general procedure **5** using cloperastine *N*-oxide (69.2 mg, 0.20 mmol, 1.0 equiv), pivaloyl chloride (62 μL, 0.50 mmol, 2.5 equiv), MeCN (2 mL, 0.1 M), DMF (47 μL, 0.60 mmol, 3.0 equiv), trimethyl(trifluoromethyl)silane (118 μL, 0.80 mmol, 4.0 equiv), potassium bifluoride (62.5 mg, 0.80 mmol, 4.0 equiv) and trifluoroacetic acid (61 μL, 0.80 mmol, 4.0 equiv). Purification by reverse phase automated flash column chromatography (RediSep Rf 5.5 g C18-column, 0–100% MeCN in H_2O) delivered a mixture of diastereomers of the title compound as a colourless oil (53.3 mg, 67%, 0.13 mmol, d.r. 1:1).

^{1}H NMR (700 MHz, $CDCl_3$) δ 7.35 – 7.25 (m, 18H, d_{major} and d_{minor}), 5.35 (s, 2H, d_{major} and d_{minor}), 3.54 (td, *J* = 5.9, 1.8 Hz, 4H, d_{major} and d_{minor}), 3.25 – 3.14 (m, 2H, d_{major} and d_{minor}), 2.99 (t, *J* = 5.9 Hz, 4H, d_{major} and d_{minor}), 2.96 – 2.86 (m, 2H, d_{major} and d_{minor}), 2.64 (dt, *J* = 12.3, 6.2 Hz, 2H, d_{major} and d_{minor}), 1.78 (app. q, *J* = 5.5 Hz, 4H, d_{major} and d_{minor}), 1.69 – 1.62 (m, 2H, d_{major} and d_{minor}), 1.59 – 1.53 (m, 4H, d_{major} and d_{minor}), 1.53 – 1.47 (m, 2H, d_{major} and d_{minor}).

^{13}C{H} NMR (176 MHz, $CDCl_3$) δ 142.0 (d_{major} and d_{minor}), 141.1 (d_{minor}), 141.1 (d_{major}), 133.3 (d_{major}), 133.3 (d_{minor}), 128.7 (2C, d_{major}), 128.6 (2C, d_{minor}), 128.6 (2C, d_{minor}), 128.6 (2C, d_{major}), 128.4 (2C, d_{major} and d_{minor}), 127.8 (d_{major}), 127.8 (d_{minor}), 127.7 (q, *J* = 290.1 Hz, d_{major} and d_{minor}), 127.1 (2C, d_{major}), 127.0 (2C, d_{minor}), 83.3 (d_{major} and d_{minor}), 67.8 (d_{major}), 67.8 (d_{minor}), 59.8 (q, *J* = 26.4 Hz, d_{major}), 59.8 (q, *J* = 24.6 Hz, d_{minor}), 54.8 (dq, *J* = 3.0, 1.5 Hz, d_{major} and d_{minor}), 48.9 (d_{major} and d_{minor}), 24.3 (d_{major}), 24.3 (d_{minor}), 24.1 (q, *J* = 1.8 Hz, d_{major}) , 24.1 (q, *J* = 1.8 Hz, d_{minor}), 20.5 (d_{major} and d_{minor}).

^{19}F{H} NMR (471 MHz, $CDCl_3$) δ -66.2 (F_{minor}), -66.2 (F_{major}).

HRMS: *m/z* calculated for $C_{21}H_{23}NOF_3Cl$ $[M+H]^+$ 398.1493; found 398.1497.

IR (film, cm^{-1}): 2940, 2854, 1489, 1451, 1241, 1189, 1120, 1075, 1013, 850, 796, 754, 698.

5-(2-chlorobenzyl)-4-(trifluoromethyl)-4,5,6,7-tetrahydrothieno[3,2-c]pyridine (19):

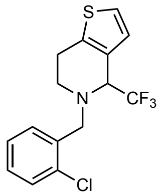

Prepared according to general procedure **5** using ticlopidine *N*-oxide (55.8 mg, 0.20 mmol, 1.0 equiv), pivaloyl chloride (62 µL, 0.50 mmol, 2.5 equiv), MeCN (2 mL, 0.1 M), DMF (47 µL, 0.60 mmol, 3.0 equiv), trimethyl(trifluoromethyl)silane (118 µL, 0.80 mmol, 4.0 equiv), potassium bifluoride (62.5 mg, 0.80 mmol, 4.0 equiv) and trifluoroacetic acid (61 µL, 0.80 mmol, 4.0 equiv). Purification by reverse phase automated flash column chromatography (RediSep Rf 5.5 g C18-column, 0–100% MeCN in H_2O) delivered the title compound as a white solid (29.0 mg, 44%, 0.09 mmol).

^{1}H NMR (700 MHz, $CDCl_3$) δ 7.63 (dd, *J* = 7.7, 1.7 Hz, 1H), 7.36 (d, *J* = 7.9 Hz, 1H), 7.29 (t, *J* = 7.4 Hz, 1H), 7.22 (td, *J* = 7.6, 1.7 Hz, 1H), 7.16 (d, *J* = 5.2 Hz, 1H), 6.95 (d, *J* = 5.2 Hz, 1H), 4.17 (q, *J* = 8.0 Hz, 1H), 4.04 (d, *J* = 14.7 Hz, 1H), 3.93 (d, *J* = 14.7 Hz, 1H), 3.40 – 3.30 (m, 1H), 3.10 – 2.98 (m, 2H), 2.75 – 2.62 (m, 1H).

^{13}C{H} NMR (176 MHz, $CDCl_3$) δ 138.2, 136.2, 134.2, 130.5, 129.6, 128.6, 127.0, 127.0 (q, *J* = 2.1 Hz), 126.2, 125.8 (q, *J* = 284.6 Hz), 122.5, 60.5 (q, *J* = 29.3 Hz), 55.7 (q, *J* = 1.3 Hz), 45.11 (qf, *J* = 1.9 Hz).

^{19}F{H} NMR (471 MHz, $CDCl_3$) δ -71.5.

HRMS: *m/z* calculated for $C_{15}H_{13}NF_3SCl$ $[M+H]^+$ 332.0482; found 332.0487.

IR (solid, cm^{-1}): 2827, 1453, 1349, 1335, 1271, 1259, 1191, 1164, 1150, 1115, 1092, 1033, 1016, 967, 938, 843, 765, 756, 712, 704, 683, 675, 638, 617.

(7a*S*,13a*R*,13b*R*)-41-(trifluoromethyl)dodecahydro-1*H*,5*H*,10H-dipyrido[2,1-f:3',2',1'-ij][1,6]naphthyridin-10-one (**20A & 20B**):

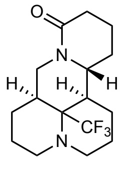

Prepared according to general procedure **5** using Matrine *N*-oxide (52.9 mg, 0.20 mmol, 1.0 equiv), pivaloyl chloride (62 µL, 0.50 mmol, 2.5 equiv), MeCN (2 mL, 0.1 M), DMF (47 µL, 0.60 mmol, 3.0 equiv), trimethyl(trifluoromethyl)silane (118 µL, 0.80 mmol, 4.0 equiv), potassium bifluoride (62.5 mg, 0.80 mmol, 4.0 equiv) and trifluoroacetic acid (62 µL, 0.80 mmol, 4.0 equiv). Purification by reverse phase automated flash column (RediSep Rf 5.5. g C18-column 0–100% MeCN in H_2O) delivered two diastereomers of the title compound (total 21.4 mg, 34%, 0.06 mmol, d.r. 2:1).

Major diastereomer (13.9 mg, 22%, 0.04 mmol).

(4[1]R,7aS,13aR,13bR)-4[1]-(trifluoromethyl)dodecahydro-1H,5H,10H-dipyrido[2,1-f:3',2',1'-ij][1,6]naphthyridin-10-one (20A):

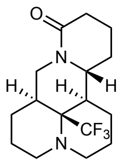

Obtained as a as pale yellow oil.

^{1}H NMR (700 MHz, $CDCl_3$) δ 4.44 (ddd, *J* = 11.6, 5.2, 2.6 Hz, 1H), 3.56 (td, *J* = 10.8, 2.2 Hz, 1H), 3.02 – 2.95 (m, 1H), 2.93 (tt, *J* = 12.1, 3.7 Hz, 1H), 2.88 – 2.80 (m, 1H), 2.61 – 2.53 (m, 2H), 2.49 (ddt, *J* = 17.4, 5.2, 2.4 Hz, 1H), 2.33 (ddd, *J* = 18.3, 12.6, 6.4 Hz, 1H), 2.12 – 2.08 (m, 1H), 1.90 – 1.86 (m, 1H), 1.81 – 1.73 (m, 5H), 1.64 (ddddd, *J* = 21.7, 13.2, 10.4, 5.1, 2.8 Hz, 3H), 1.54 (dq, *J* = 13.6, 2.7 Hz, 1H), 1.49 (dddd, *J* = 15.3, 10.4, 4.9, 2.4 Hz, 2H), 1.31 – 1.23 (m, 1H).

^{13}C{H} NMR (126 MHz, $CDCl_3$) δ 170.3, 130.4 (q, *J* = 306.2 Hz), 61.1 (q, *J* = 17.5 Hz), 56.4 (q, *J* = 3.2 Hz), 51.7, 49.3 (q, *J* = 2.1 Hz), 48.4 (q, *J* = 2.0 Hz), 44.7, 42.7 (q, *J* = 3.0 Hz), 32.6, 29.3, 24.9, 24.8, 23.2 (d, *J* = 2.2 Hz), 21.2 (d, *J* = 2.3 Hz), 19.9.

^{19}F{H} NMR (471 MHz, $CDCl_3$) δ -46.3.

HRMS: *m/z* calculated for $C_{16}H_{23}N_2OF_3$ $[M+H]^+$ 317.1835; found 317.1839.

IR (film, cm^{-1}): 2930, 2856, 1639, 1442, 1412, 1328, 1273, 1210, 1171, 1136, 1116, 876.

The relative stereochemistry of the title compound was determined by 2D NMR experimentation. In the heteronuclear NOESY spectrum, only signals arising from protons close to the 19F are observed. A clear NOE signal from the fluorine to a CH proton at 3.6 ppm can be observed, which is the N-CH proton, thus they must be on the same face of the molecule.

Minor diastereomer (7.5 mg, 12%, 0.02 mmol).

(4[1]S,7aS,13aR,13bR)-4[1]-(trifluoromethyl)dodecahydro-1H,5H,10H-dipyrido[2,1-f:3',2',1'-ij][1,6]naphthyridin-10-one (20B):

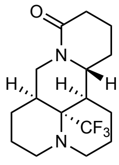

Obtained as a as pale yellow oil.

^{1}H NMR (500 MHz, $CDCl_3$) δ 4.4 (dd, *J* = 13.0, 4.7 Hz, 1H), 3.8 (ddd, *J* = 10.5, 8.7, 6.0 Hz, 1H), 3.1 (t, *J* = 12.7 Hz, 1H), 3.0 – 2.9 (m, 2H), 2.7 – 2.6 (m, 1H), 2.6 – 2.5 (m, 1H), 2.4 (dtd, *J* = 17.2, 4.7, 1.1 Hz, 1H), 2.2 (ddd, *J* = 16.8, 10.8, 5.3 Hz, 1H), 2.2 – 2.1 (m, 1H), 2.1 (ddtd, *J* = 16.3, 10.3, 5.0, 2.4 Hz, 1H), 2.0 – 1.9 (m, 2H), 1.9 – 1.8 (m, 2H), 1.7 – 1.6 (m, 4H), 1.5 – 1.4 (m, 4H).

^{13}C{H} NMR (126 MHz, $CDCl_3$) δ 169.3, 130.3 (q, *J* = 356.2 Hz), 53.2, 50.7 (2C), 42.5, 41.6, 33.6, 32.7, 27.8, 24.3 (d, *J* = 3.4 Hz), 20.8 (d, *J* = 3.1 Hz), 20.1, 19.5, 19.1.

^{19}F{H} NMR (471 MHz, $CDCl_3$) δ -56.5.

HRMS: *m/z* calculated for $C_{16}H_{23}N_2OF_3$ $[M+H]^+$ 317.1835; found 317.1838.

IR (film, cm^{-1}): 2931, 2848, 1642, 1470, 1444, 1302, 1288, 1230, 1156, 1139, 1123, 1113, 1099,1085, 1072, 893.

The relative stereochemistry of the title compound was determined by analogy to **20A** and the absence of an NOE signal from the fluorine to the CH proton at 3.8 ppm, which is the N-CH proton, thus they must be on opposite faces of the molecule.

2-(2-(trifluoromethyl)piperidin-1-yl)ethyl 3-methyl-4-oxo-2-phenyl-4H-chromene-8-carboxylate (21):

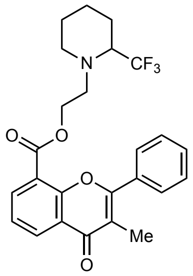

Prepared according to general procedure **5** using flavoxate *N*-oxide (81.4 mg, 0.20 mmol, 1.0 equiv), pivaloyl chloride (62 μL, 0.50 mmol, 2.5 equiv), MeCN (2 mL, 0.1 M), DMF (47 μL, 0.60 mmol, 3.0 equiv), trimethyl(trifluoromethyl)silane (118 μL, 0.80 mmol, 4.0 equiv), potassium bifluoride (62.5 mg, 0.80 mmol, 4.0 equiv) and trifluoroacetic acid (61 μL, 0.80 mmol, 4.0 equiv). Purification by reverse phase automated flash column chromatography (RediSep Rf 5.5 g C18-column, 0–100% MeCN in H_2O) delivered the title compound as a pale yellow oil (39.1 mg, 43%, 0.09 mmol).

^{1}H NMR (700 MHz, $CDCl_3$) δ 8.47 (d, *J* = 7.9, 1H), 8.25 (d, *J* = 7.4, 1H), 7.82 – 7.73 (m, 2H), 7.57 – 7.49 (m, 3H), 7.45 (t, *J* = 7.7, 1H), 4.46 (dt, *J* = 11.3, 5.6 Hz, 1H), 4.35 (dt, *J* = 11.4, 5.8 Hz, 1H), 3.09 – 2.98 (m, 3H), 2.89 – 2.81 (m, 1H), 2.67 – 2.59 (m, 1H), 2.23 (s, 3H), 1.76 – 1.70 (m, 1H), 1.67 – 1.62 (m, 1H), 1.61 – 1.49 (m, 2H), 1.49 – 1.39 (m, 2H).

^{13}C{H} NMR (176 MHz, $CDCl_3$) δ 178.4, 164.5, 161.2, 154.6, 136.2, 133.2, 130.9, 130.7, 129.4 (2C), 128.6 (2C), 127.7 (q, *J* = 290.8 Hz), 124.1, 123.4, 120.9, 117.8, 63.2 (d, *J* = 1.2 Hz), 59.7 (q, *J* = 24.8 Hz), 53.6 (q, *J* = 1.5 Hz), 48.1, 24.3, 24.2 (q, *J* = 1.9 Hz), 20.2, 11.9.

^{19}F{H} NMR (471 MHz, $CDCl_3$) δ -65.9.

HRMS: *m/z* calculated for $C_{25}H_{24}NO_4F_3$ $[M+H]^+$ 460.1730; found 460.1736.

IR (film, cm^{-1}): 2948, 2854, 1726, 1635, 1478, 1439, 1390, 1372, 1261, 1120, 1076, 1023, 756, 697.

(1S,2R,3R,4aS,13bS,14aS)-2,11-dimethoxy-1-(methoxycarbonyl)-13b-(trifluoromethyl)-3-((3,4,5-trimethoxybenzoyl)oxy)-2,3,4,4a,5,6,7,8,13,13b,14,14a-dodecahydro-1H-indolo[2', 3':3,4]pyrido[1,2-b]isoquinolin-6-ium trifluoroacetate (22):

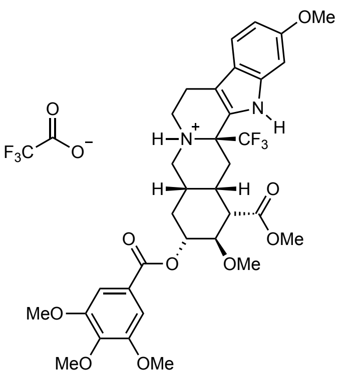

Prepared according to general procedure **5** using reserpine *N*-oxide (124.9 mg, 0.20 mmol, 1.0 equiv), pivaloyl chloride (62 μL, 0.50 mmol, 2.5 equiv), MeCN (2 mL, 0.1 M), DMF (47 μL, 0.60 mmol, 3.0 equiv), trimethyl(trifluoromethyl)silane (118 μL, 0.80 mmol, 4.0 equiv), potassium bifluoride (62.5 mg, 0.80 mmol, 4.0 equiv) and trifluoroacetic acid (61 μL, 0.80 mmol, 4.0 equiv). Purification by reverse phase automated flash column chromatography (RediSep Rf 5.5 g C18-column, 0–100% MeCN in H_2O), followed by preparative HPLC delivered the title compound as an amorphous brown solid as a single diastereomer (13.2 mg, 8%, 0.02 mmol, d.r. >20:1).

^{1}H NMR (500 MHz, $CDCl_3$) δ 7.89 (s, 1H), 7.40 (d, *J* = 8.5 Hz, 1H), 7.28 (s, 2H), 6.90 (d, *J* = 2.2 Hz, 1H), 6.81 (dd, *J* = 8.6, 2.2 Hz, 1H), 5.05 (ddd, *J* = 11.8, 9.4, 5.2 Hz, 1H), 3.89 (s, 3H), 3.87 (s, 6H), 3.86 (s, 3H), 3.81 (s, 3H), 3.72 (dd, *J* = 11.2, 9.4 Hz, 1H), 3.65 (d, *J* = 11.8 Hz, 1H), 3.59 – 3.53 (m, 1H), 3.44 (s, 3H), 2.87 – 2.84 (m, 2H), 2.78 – 2.74 (m, 1H), 2.73 – 2.62 (m, 3H), 2.21 – 2.05 (m, 3H), 2.00 – 1.88 (m, 2H).

^{13}C{H} NMR (126 MHz, $CDCl_3$) δ 172.5, 165.6, 157.2, 153.1 (2C), 142.4, 137.1, 129.3, 128.7 (q, *J* = 302.0 Hz), 125.3, 120.7, 119.5, 111.3, 109.9, 106.9 (2C), 95.0, 77.7, 77.6, 61.1, 61.1, 59.5 (q, J = 22.4 Hz), 56.4 (2C), 56.0, 54.2, 52.2, 52.0, 48.3, 33.9, 33.0, 30.1, 29.1, 21.2.

^{19}F{H} NMR (471 MHz, $CDCl_3$) δ -62.3, -75.7.

HRMS: *m/z* calculated for $C_{34}H_{39}N_2O_9F_3$ $[M+H]^+$ 677.2680; found 677.2681.

IR (film, cm^{-1}): 3007, 2936, 2842, 1712, 1629, 1589, 1503, 1461, 1415, 1330, 1223, 1144, 1124, 1001, 751.

The relative stereochemistry of the title compound was determined by 2D NMR experimentation. In the heteronuclear NOESY spectrum, only signals arising from protons close to the 19F are observed. A clear NOE signal from the fluorine to a CH proton at 2.695 ppm can be observed (looking at the original HSQC there are 3 peaks in the 2.6-2.7ppm region and the CH is the highest shift one, which is what we see the correlation to here). Strong NOE correlations are also observed to two N-C**H**$_2$ protons from separate N-C**H**$_2$ groups at 3.57 and 3.65 ppm (these are unambiguous) and a CH_2 proton at 2.097 ppm. As a result, the CF_3 group must be on the same side as the two C**H** protons.

4-(6-fluorobenzo[d]isoxazol-3-yl)-1-(2-(2-methyl-4-oxo-6,7,8,9-tetrahydro-4H-pyrido[1,2-a]pyrimidin-3-yl)ethyl)-2-(trifluoromethyl)piperidin-1-ium trifluoroacetate (23):

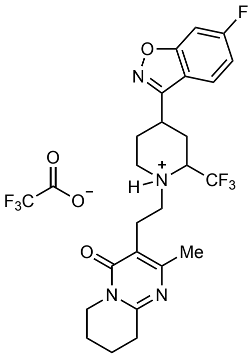

Prepared according to general procedure **5** using risperidone *N*-oxide (85.3 mg, 0.20 mmol, 1.0 equiv), pivaloyl chloride (62 μL, 0.50 mmol, 2.5 equiv), MeCN (2 mL, 0.1 M), DMF (47 μL, 0.60 mmol, 3.0 equiv), trimethyl(trifluoromethyl)silane (118 μL, 0.80 mmol, 4.0 equiv), potassium bifluoride (62.5 mg, 0.80 mmol, 4.0 equiv) and trifluoroacetic acid (61 μL, 0.80 mmol, 4.0 equiv). Purification by reverse phase automated flash column chromatography (RediSep Rf 5.5 g C18-column, 0–100% MeCN in H_2O), followed by preparative HPLC delivered the title compound as a colourless oil (35.5 mg, 30%, 0.06 mmol).

^{1}H NMR (700 MHz, $CDCl_3$) δ 7.67 (dd, *J* = 8.7, 5.0 Hz, 1H), 7.28 (dd, *J* = 8.3, 2.1 Hz, 1H), 7.11 (td, *J* = 8.8, 2.1 Hz, 1H), 4.31 – 4.21 (m, 1H), 3.99 (t, *J* = 6.2 Hz, 2H), 3.63 (tt, *J* = 9.8, 4.4 Hz, 1H), 3.46 (dt, *J* = 12.8, 4.8 Hz, 1H), 3.44 – 3.38 (m, 1H), 3.33 – 3.19 (m, 4H), 2.98 (ddd, *J* = 14.3, 8.5, 6.1 Hz, 1H), 2.90 (ddd, *J* = 14.3, 8.5, 6.7 Hz, 1H), 2.56 – 2.42 (s in m, 5H), 2.39 – 2.32 (m, 1H), 2.28 – 2.18 (m, 1H), 2.12 – 2.05 (m, 2H), 2.02 – 1.95 (m, 2H).

^{13}C{H} NMR (176 MHz, $CDCl_3$) δ 164.5 (d, J = 252.1 Hz), 164.0 (d, J = 13.7 Hz), 161.3, 159.5, 159.2, 150.7, 125.3 (q, J = 286.3 Hz), 122.0 (d, J = 11.0 Hz), 118.9, 116.6 (d, J = 1.2 Hz), 113.2 (d, J = 25.5 Hz), 97.7 (d, J = 26.9 Hz), 58.3 (q, J = 28.1 Hz), 52.4, 47.6, 44.1, 28.2, 27.3, 26.9 (2C), 22.4, 20.7, 17.0, 16.4.

^{19}F{H} NMR (471 MHz, $CDCl_3$) δ -64.3, -75.92, -108.0.

HRMS: *m/z* calculated for $C_{24}H_{27}N_4O_2F_4$ $[M+H]^+$ 479.2065; found 479.2065.

IR (film, cm^{-1}): 2924, 2853, 1697, 1666, 1614, 1553, 1415, 1272, 1177, 1136, 956, 751, 719, 664.

5.6 Additional substrates

1-benzyl-1-hydroxypiperidin-1-ium chloride (5):

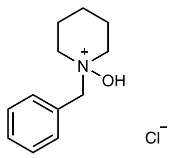

1-benzylpiperidine *N*-oxide (38.5 mg, 0.20 mmol, 1.0 equiv) was added to a solution of 4 N hydrochloric acid in dioxane (50 µL, 0.40 mmol, 2.0 equiv) and stirred for 30 mins. The solvent was removed *in vacuo* to yield the title compound as a white solid (30.4 mg, 79%, 0.16 mmol).

^{1}H NMR (500 MHz, $CDCl_3$) δ 7.73 – 7.64 (m, 2H), 7.44 – 7.38 (m, 1H), 7.38 – 7.31 (m, 2H), 5.11 (s, 2H), 3.84 (br d, J = 11.9 Hz, 2H), 3.63 (td, J = 12.5, 2.8 Hz, 2H), 2.21 – 2.02 (m, 2H), 1.84 – 1.66 (m, 3H), 1.58 – 1.42 (m, 1H).

^{13}C{H} NMR (126 MHz, $CDCl_3$) δ 133.3 (2C), 130.5, 128.8 (2C), 127.0, 71.5, 62.5 (2C), 20.9, 20.3 (2C).

HRMS: *m/z* calculated for $C_{12}H_{18}NO$ $[M]^+$ 192.1383, found 192.1380.

IR (solid, cm^{-1}): 2942, 2862, 2537 (O–H stretch), 1522, 1445, 1381, 1221, 935, 890.

***1-benzyl-2-(fluoromethyl)piperidine* (6y):**

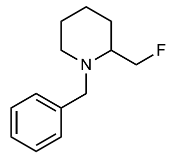

NB: This amine is volatile at room temperature and atmospheric pressure.

Prepared according to general procedure **2** using 1-benzylpiperidine *N*-oxide (38.2 mg, 0.20 mmol, 1.0 equiv), pivaloyl chloride (62 μL, 0.50 mmol, 2.5 equiv), TBSOTf (35 μL, 0.10 mmol, 75 mol%), copper iodide (15.2 mg, 0.08 mmol, 40 mol%), fluoroiodomethane (35 μL, 0.50 mmol, 2.5 equiv) and zinc dust (33.2 mg, 0.50 mmol, 2.5 equiv). Purification by automated flash column chromatography (RediSep Rf Gold Si 4g, 0–20% [10% Et_2O and 3% Et_3N in PE] in PE) delivered the title compound as a pale-yellow oil (7.0 mg, 17%, 0.03 mmol).

Spectra are in accordance with the literature data.[43]

*2-(adamantan-1-yl)-1-benzyl-5-methyl-piperidine (**6z**) and (2R,3R)-2-((1s,3S)-adamantan-1-yl)-1-benzyl-3-methyl-piperidine (**6z'**):*

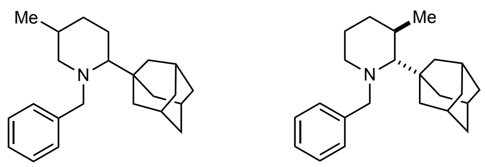

Prepared according to general procedure **3** using 1-benzyl-3-methylpiperidine *N*-oxide (41.0 mg, 0.20 mmol, 1.0 equiv), pivaloyl chloride (62 μL, 0.50 mmol, 2.5 equiv), copper perchlorate (78.2 mg, 0.30 mmol, 1.5 equiv), TBSOTf (23 μL, 0.10 mmol, 50 mol%), copper iodide (19.0 mg, 0.10 mmol, 50 mol%), 1-iodoadamantane (157.0 mg, 0.60 mmol, 3.0 equiv) and zinc dust (39.2 mg, 0.60 mmol, 3.0 equiv). Purification by reverse phase automated flash column chromatography (RediSep Rf 5.5 g C18-column, 0–100% MeCN in H_2O) followed by automated flash column chromatography (RediSep Rf Gold Si 4g, 0–10% EtOAc in PE) delivered the two regioisomers (total 20.0 mg, 31%, 0.06 mmol, 2.4:1 r.r)

<u>*Major regioisomer (14.5 mg, 22%, 0.04 mmol, d.r. 1:1.1)*</u>

2-(adamantan-1-yl)-1-benzyl-5-methyl-piperidine (6z)

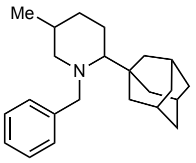

Obtained as a pale yellow oil and as a mixture of diastereomers (1:1.1. d.r).

^{1}H NMR (700 MHz, $CDCl_3$) δ 7.41 (d, *J* = 7.1 Hz, 2H, d_{minor}), 7.38 (d, *J* = 7.1 Hz, 2H, d_{major}), 7.31 (td, *J* = 7.6, 4.5 Hz, 4H, d_{major} and d_{minor}), 7.22 (q, *J* = 7.6 Hz, 2H, d_{major} and d_{minor}), 4.18 (d, *J* = 13.9 Hz, 1H, d_{minor}), 3.95 (d, *J* = 13.7 Hz, 1H, d_{major}), 3.83 (d, *J* = 14.0 Hz, 1H, d_{minor}), 3.69 (d, *J* = 13.7 Hz, 1H, d_{major}), 2.75 (ddd, *J* = 14.8, 11.0, 4.0 Hz, 1H, d_{minor}), 2.57 – 2.52 (m,

1H, d_{minor}), 2.49 (dd, J = 14.4, 5.7 Hz, 1H, d_{major}), 2.20 (dd, J = 14.4, 11.4 Hz, 1H, d_{major}), 2.07 (t, J = 7.4 Hz, 1H, d_{minor}), 2.01 – 1.95 (m, 9H, d_{major} and d_{minor}), 1.80 – 1.77 (m, 5H, d_{major} and d_{minor}), 1.72 – 1.64 (m, 19H, d_{major} and d_{minor}), 1.59 – 1.53 (m, 6H, d_{major} and d_{minor}), 1.46 (dq, J = 8.5, 4.9 Hz, 1H, d_{minor}), 1.34 – 1.29 (m, 1H, d_{major}), 1.19 (d, J = 7.2 Hz, 3H, d_{minor}), 1.09 (dt, J = 12.4, 3.7 Hz, 1H, d_{minor}), 0.71 (d, J = 6.7 Hz, 3H, d_{major}).

^{13}C{H} NMR (176 MHz, $CDCl_3$) δ 142.4 (d_{minor}), 141.6 (d_{major}), 128.7 (2C, d_{major}), 128.6 (2C, d_{minor}), 128.2 (2C, d_{major}), 128.2 (2C, d_{minor}), 126.7 (d_{major}), 126.6 (d_{minor}), 75.9 (d_{minor}), 69.6 (d_{major}), 63.4 (d_{minor}), 62.0 (d_{major}), 51.3 (d_{major}), 45.3 (d_{minor}), 41.6 (3C, d_{minor}), 40.5 (d_{minor}), 40.0 (3C, d_{major}), 39.2 (d_{major}), 37.6 (3C, d_{major}), 37.5 (3C, d_{minor}), 29.6 (d_{minor}), 29.1 (3C, d_{minor}), 28.9 (3C, d_{major}), 28.9 (d_{major}), 25.9 (d_{minor}), 24.4 (d_{minor}), 22.3 (d_{major}), 19.7 (d_{major}), 17.9 (d_{major}), 16.2 (d_{minor}).

HRMS: *m/z* calculated for $C_{23}H_{33}N$ $[M+H]^+$ 324.2686; found 324.2690.

IR (film, cm^{-1}): 2898, 2844, 1449, 1347, 1101, 1057, 991, 731, 697.

<u>*Minor regioisomer (5.5 mg, 9%, 0.02 mmol, d.r. >20:1)*</u>

(2R,3R)-2-((1s,3S)-adamantan-1-yl)-1-benzyl-3-methyl-piperidine (6z'):

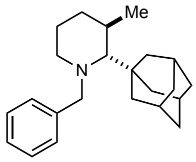

Obtained as a colourless oil and a single diastereomer.

^{1}H NMR (700 MHz, $CDCl_3$) δ 7.39 (d, J = 7.5 Hz, 2H), 7.31 (t, J = 7.6 Hz, 2H), 7.23 (t, J = 7.3 Hz, 1H), 3.98 (d, J = 13.6 Hz, 1H), 3.77 (d, J = 13.6 Hz, 1H), 2.67 (ddd, J = 13.9, 4.3, 2.2 Hz, 1H), 2.30 (dd, J = 11.8, 2.4 Hz, 1H), 2.13 (dd, J = 13.7, 10.9 Hz, 1H), 1.98 – 1.94 (m, 3H), 1.94 – 1.85 (m, 2H), 1.78 (d, J = 12.3 Hz, 3H), 1.72 – 1.68 (m, 6H), 1.61 (dd, J = 12.1, 3.6 Hz, 4H), 1.50 – 1.44 (m, 1H), 1.04 (qd, J = 12.6, 4.1 Hz, 1H), 0.67 (d, J = 6.5 Hz, 3H).

^{13}C{H} NMR (176 MHz, $CDCl_3$) δ 140.9, 128.6 (2C), 128.4 (2C), 126.7, 71.5, 58.1, 52.3, 40.7 (3C), 37.5 (3C), 36.4, 35.2, 29.1 (3C), 23.7, 20.0, 19.5.

HRMS: *m/z* calculated for $C_{23}H_{33}N$ $[M+H]^+$ 324.2686; found 324.2692.

IR (film, cm^{-1}): 2900, 2845, 1449, 1343, 1071, 1018, 756, 727, 696.

The relative stereochemistry of the title compound was determined by *J*-coupling constant analysis. The N-C**H** proton exhibits a *J*-coupling constant of 11.8 Hz, which is the result of axial-axial coupling. Consequently, the two substituents must be on opposite faces of the ring.

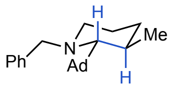

$^3J_{ax\text{-}ax}$ = 11.8 Hz

2-((1s,3s)-adamantan-1-yl)-1-benzyl-4-methylpiperidine (6aa):

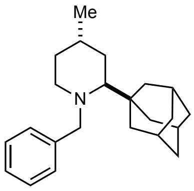

Prepared according to general procedure **3** using 1-benzyl-4-methylpiperidine *N*-oxide (41.0 mg, 0.20 mmol, 1.0 equiv), pivaloyl chloride (62 µL, 0.50 mmol, 2.5 equiv), copper perchlorate (78.2 mg, 0.30 mmol, 1.5 equiv), TBSOTf (23 µL, 0.10 mmol, 50 mol%), copper iodide (19.0 mg, 0.10 mmol, 50 mol%), 1-iodoadamantane (157.0 mg, 0.60 mmol, 3.0 equiv) and zinc dust (39.2 mg, 0.60 mmol, 3.0 equiv). Purification by reverse phase automated flash column chromatography (RediSep Rf 5.5 g C18-column, 0–100% MeCN in H_2O) followed by automated flash column chromatography (RediSep Rf Gold Si 4g, 0–10% EtOAc in PE) delivered a mixture of diastereomers of the title compound as a colourless oil (10.5 mg, 16%, 0.03 mmol, d.r. 2.3:1).

^{1}H NMR (700 MHz, $CDCl_3$) δ 7.45 – 7.40 (m, 2H, d_{minor}), 7.40 – 7.34 (m, 2H, d_{major}), 7.30 (q, *J* = 7.6 Hz, 4H, d_{major} and d_{minor}), 7.22 (td, *J* = 7.2, 5.5 Hz, 2H, d_{major} and d_{minor}), 3.91 (dd, *J* = 13.5, 4.4 Hz, 2H, d_{major} and d_{minor}), 3.68 (dd, *J* = 20.6, 13.5 Hz, 2H, d_{major} and d_{minor}), 2.86 (ddd, *J* = 14.9, 8.8, 6.4 Hz, 1H, d_{major}), 2.69 – 2.61 (m, 2H, d_{major} and d_{minor}), 2.48 (ddd, *J* = 14.2, 7.2, 4.8 Hz, 1H, d_{minor}), 2.26 (dd, *J* = 12.1, 3.9 Hz, 1H, d_{minor}), 2.17 (dd, *J* = 6.9, 2.4 Hz, 1H, d_{major}), 1.96 (dp, *J* = 18.0, 3.3 Hz, 6H, d_{major} and d_{minor}), 1.77 – 1.43 (m, 30H, d_{major} and d_{minor}), 1.28 – 1.06 (m, 4H, d_{major} and d_{minor}), 0.96 (d, *J* = 6.3 Hz, 3H, d_{minor}), 0.91 (d, *J* = 6.5 Hz, 3H, d_{major}).

^{13}C{H} NMR (176 MHz, $CDCl_3$) δ 141.5 (d_{major}), 141.0 (d_{minor}), 128.9 (2C, d_{major}), 128.5 (2C, d_{minor}), 128.2 (2C, d_{minor}), 128.0 (2C, d_{major}), 126.6 (d_{minor}), 126.6 (d_{major}), 71.4 (d_{minor}), 67.4 (d_{major}), 59.9 (d_{major}), 55.6 (d_{minor}), 47.1 (d_{major}), 46.0 (d_{minor}), 41.7 (3C, d_{major}), 39.7 (3C, d_{minor}), 38.8 (d_{major}), 37.5 (3C, d_{minor}), 37.4 (d_{minor}), 37.3 (3C, d_{major}), 29.7 (d_{minor}), 29.0 (3C, d_{major}), 28.8 (3C, d_{minor}), 28.1 (d_{minor}), 28.0 (d_{major}), 27.7 (d_{major}), 27.5 (d_{major}), 27.1 (d_{minor}), 23.5 (d_{major}), 23.2 (d_{minor}).

HRMS: *m/z* calculated for $C_{23}H_{33}N$ $[M+H]^+$ 324.2686; found 324.2689.

IR (film, cm^{-1}): 2899, 2844, 1450, 1347, 1099,987, 736, 697.

The relative stereochemistry of the title compound was determined by 2D NMR experimentation. In the major diastereomer, a clear NOE signal from the methyl signal at 0.91 ppm to the N-C**H** at 2.19 ppm was observed, thus they must be on the same face of

the ring. The corresponding signal in the minor diastereomer from the methyl signal at 0.98 ppm to the N-C**H** at 2.28 ppm was absent.

*2-(adamantan-1-yl)-1-benzylazepane (**6ab**) and 1-((adamantan-1-yl)(phenyl)methyl) azepane (**6ab'**):*

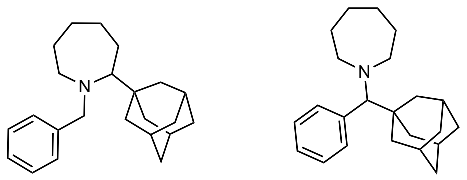

Prepared according to general procedure **3** using 1-benzylazepane *N*-oxide (41.0 mg, 0.20 mmol, 1.0 equiv), pivaloyl chloride (62 μL, 0.50 mmol, 2.5 equiv), copper perchlorate (78.2 mg, 0.30 mmol, 1.5 equiv), TBSOTf (23 μL, 0.10 mmol, 50 mol%), copper iodide (19.0 mg, 0.10 mmol, 50 mol%), 1-iodoadamantane (157.0 mg, 0.60 mmol, 3.0 equiv) and zinc dust (39.2 mg, 0.60 mmol, 3.0 equiv). Purification by reverse phase automated flash column chromatography (RediSep Rf 5.5 g C18-column, 0–100% MeCN in H_2O) followed by automated flash column chromatography (RediSep Rf Gold Si 4g, 0–5% EtOAc in PE) delivered the two regioisomers (total 15.5 mg, 24%, 0.05 mmol, 2.2:1 r.r)

Major regioisomer (11.8 mg, 18%, 0.04 mmol)

2-(adamantan-1-yl)-1-benzylazepane (6ab):

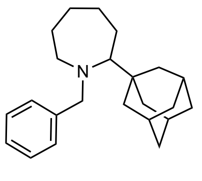

Obtained as an amorphous white solid.

^{1}H NMR (700 MHz, $CDCl_3$) δ 7.45 (d, *J* = 8.3 Hz, 2H), 7.31 (t, *J* = 7.7 Hz, 2H), 7.22 (t, *J* = 7.4 Hz, 1H), 4.28 (d, *J* = 14.2 Hz, 1H), 3.82 (d, *J* = 14.2 Hz, 1H), 2.78 (dd, *J* = 15.4, 5.1 Hz, 1H), 2.68 – 2.54 (m, 1H), 2.21 (dd, *J* = 12.4, 5.2 Hz, 1H), 2.02 – 1.93 (m, 4H), 1.84 (dd, *J* = 13.7, 7.8 Hz, 1H), 1.76 (dq, *J* = 11.8, 2.7 Hz, 4H), 1.74 – 1.68 (m, 4H), 1.68 – 1.63 (m, 3H), 1.56 (dd, *J* = 12.1, 2.8 Hz, 3H), 1.46 (q, *J* = 12.9 Hz, 1H), 1.36 (q, *J* = 12.3 Hz, 1H), 1.19 (d, *J* = 12.5 Hz, 1H), 1.09 (s, 1H).

^{13}C{H} NMR (176 MHz, $CDCl_3$) δ 141.0, 128.5 (2C), 128.2 (2C), 126.6, 74.4, 60.0, 46.5, 39.8, 39.7 (3C), 37.7 (3C), 30.0, 28.9 (3C), 28.3, 26.9, 23.5.

HRMS: *m/z* calculated for $C_{23}H_{33}N$ $[M+H]^+$ 324.2686; found 324.2686.

IR (film, cm^{-1}): 2898, 2844, 2780, 1450, 1348, 1157, 953, 727, 696.

Minor regioisomer (3.7 mg, 6%, 0.01 mmol)

1-((adamantan-1-yl)(phenyl)methyl)azepane (6ab'):

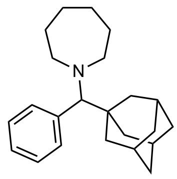

Obtained as a colourless oil.

^{1}H NMR (700 MHz, $CDCl_3$) δ 7.30 – 7.26 (m, 4H), 7.25 – 7.21 (m, 1H), 3.04 (s, 1H), 2.91 – 2.77 (m, 2H), 2.43 (ddd, *J* = 12.0, 7.5, 4.2 Hz, 2H), 1.98 – 1.91 (m, 3H), 1.91 – 1.84 (m, 3H), 1.68 (dd, *J* = 12.0, 2.8 Hz, 3H), 1.64 – 1.61 (m, 3H), 1.58 – 1.50 (m, 11H).

^{13}C{H} NMR (176 MHz, $CDCl_3$) δ 139.1, 130.8 (2C), 127.5 (2C), 126.4, 82.6, 55.2 (2C), 41.2 (3C), 38.7, 37.4 (3C), 30.0 (2C), 29.0 (3C), 27.1 (2C).

HRMS: *m/z* calculated for $C_{23}H_{33}N$ $[M+H]^+$ 324.2686; found 324.2686.

IR (film, cm^{-1}): 2901, 2846, 1311, 1234, 1128, 764, 701, 630.

(2R,4S)-1-benzyl-2,4-dimethylpiperidine (8b):

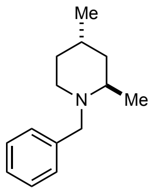

Prepared according to general procedure **4** using 1-benzyl-4-methylpiperidine *N*-oxide (41.0 mg, 0.20 mmol, 1.0 equiv), pivaloyl chloride (62 μL, 0.50 mmol, 2.5 equiv) and 3.4 M methyl magnesium bromide in 2-MeTHF (0.27 mL, 0.60 mmol, 3.0 equiv). Purification by automated flash column chromatography (RediSep Rf Gold Si 4 g, 0–20% EtOAc in PE) delivered the title compound as colourless oil and as a single diastereomer (12.3 mg, 30%, 0.06 mmol, d.r. >20:1).

^{1}H NMR (700 MHz, $CDCl_3$) δ 7.36 – 7.33 (m, 2H), 7.31 – 7.28 (m, 2H), 7.23 – 7.21 (m, 1H), 3.62 (d, *J* = 13.4 Hz, 1H), 3.53 (d, *J* = 13.5 Hz, 1H), 2.96 (dq, *J* = 11.1, 5.4 Hz, 1H), 2.48 (ddd, *J* = 11.9, 10.6, 3.2 Hz, 1H), 2.43 (dt, *J* = 11.9, 4.4 Hz, 1H), 1.71 (tdd, *J* = 10.5, 7.2, 4.0 Hz, 1H), 1.58 – 1.54 (m, 1H), 1.49 – 1.42 (m, 2H), 1.23 – 1.18 (m, 1H), 1.03 (d, *J* = 6.6 Hz, 3H), 0.90 (d, *J* = 6.6 Hz, 3H).

^{13}C{H} NMR (176 MHz, $CDCl_3$) δ 140.1, 128.9 (2C), 128.2 (2C), 126.8, 59.2, 52.0, 45.9, 40.6, 34.1, 25.2, 21.7, 11.9.

HRMS: *m/z* calculated for $C_{14}H_{21}N$ $[M+H]^+$ 204.1747; found 204.1745.

IR (film, cm^{-1}): 2953, 2918, 1849, 1697, 1463, 1377, 1065, 737, 698.

The relative stereochemistry of the title compound was determined by 2D NMR experimentation. No NOE signals were detected between the two Me-C**H** protons (shown in black), nor between the two methyl groups (shown in orange). However, clear NOE signals between the methyl groups and the adjacent C**H** (shown in green), as well as to the other C**H** (shown in purple). This can only be the case if the two methyl groups are on opposite faces. The stronger of the two methyl-opposite C**H** correlations must arise from the two groups being axial-axial and the much weaker correlation is mostly equatorial-equatorial. In the most populated conformation, the N-CH-**Me** must be axial. However, there must also be measurable population of the other conformer or else the other Me-CH correlation (equatorial-equatorial) would be too weak to see.

2-benzyl-1-methyl-1,2,3,4-tetrahydroisoquinoline (8d):

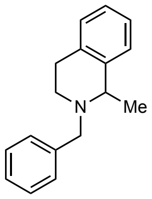

Prepared according to general procedure **4** using 2-benzyl-1,2,3,4-tetrahydroisoquinoline *N*-oxide (47.8 mg, 0.20 mmol, 1.0 equiv), pivaloyl chloride (62 μL, 0.50 mmol, 2.5 equiv) and 3.4 M methyl magnesium bromide in 2-MeTHF (0.27 mL, 0.60 mmol, 3.0 equiv). Purification by automated flash column chromatography (RediSep Rf Gold Si 4 g, 0–20% EtOAc in PE) delivered the title compound as a colourless oil (44.6 mg, 94%, 0.19 mmol).

^{1}H NMR (700 MHz, $CDCl_3$) δ 7.44 – 7.40 (m, 2H), 7.37 – 7.33 (m, 2H), 7.30 – 7.26 (m, 1H), 7.18 – 7.07 (m, 4H), 3.92 (q, *J* = 6.7 Hz, 1H), 3.85 (d, *J* = 13.6 Hz, 1H), 3.73 (d, *J* = 13.6 Hz, 1H), 3.14 – 3.05 (m, 1H), 2.93 (ddd, *J* = 14.9, 9.3, 6.2 Hz, 1H), 2.80 – 2.67 (m, 2H), 1.42 (d, *J* = 6.7 Hz, 3H).

^{13}C{H} NMR (176 MHz, $CDCl_3$) δ 140.5, 139.6, 134.4, 128.9, 128.8 (2C), 128.3 (2C), 127.5, 126.9, 125.8, 125.7, 58.2, 56.3, 43.9, 27.5, 19.8.

HRMS: *m/z* calculated for $C_{17}H_{19}N$ $[M+H]^+$ 238.1590; found 238.1595.

IR (film, cm^{-1}): 3023, 2966, 2920, 2801, 1492, 1451, 1364, 1312, 1136, 1099, 1028, 756, 732, 697.

1-benzyl-4-methyl-2-(trifluoromethyl)piperidine (9c):

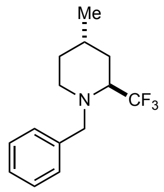

Prepared according to general procedure **5** using 1-benzyl-4-methylpiperidine *N*-oxide (41.0 mg, 0.20 mmol, 1.0 equiv), pivaloyl chloride (62 µL, 0.50 mmol, 2.5 equiv), MeCN (2 mL, 0.1 M), DMF (47 µL, 0.60 mmol, 3.0 equiv), trimethyl(trifluoromethyl)silane (118 µL, 0.80 mmol, 4.0 equiv), potassium bifluoride (62.5 mg, 0.80 mmol, 4.0 equiv) and trifluoroacetic acid (61 µL, 0.80 mmol, 4.0 equiv). Purification by reverse phase automated flash column chromatography (RediSep Rf 5.5 g C18-column, 0-100% MeCN in H_2O) delivered a mixture of diastereomers of the title compound as a pale-yellow oil (26.1 mg, 51%, 0.10 mmol, d.r. 1.5:1).

^{1}H NMR (700 MHz, $CDCl_3$) δ 7.37 – 7.30 (m, 8H, d_{major} and d_{minor}), 7.28 – 7.24 (m, 2H, d_{major} and d_{minor}), 4.19 (d, *J* = 13.4 Hz, 1H, d_{minor}), 3.98 (d, *J* = 14.1 Hz, 1H, d_{major}), 3.84 (dd, *J* = 14.2, 1.8 Hz, 1H, d_{major}), 3.38 – 3.26 (m, 2H, d_{major} and d_{minor}), 2.97 (dqd, *J* = 11.7, 7.0, 3.1 Hz, 1H, d_{minor}), 2.93 – 2.82 (m, 2H, d_{major} and d_{minor}), 2.64 (ddt, *J* = 12.4, 4.4, 1.9 Hz, 1H, d_{major}), 2.07 (td, *J* = 12.3, 2.7 Hz, 1H, d_{minor}), 1.96 – 1.87 (m, 2H, d_{major} and d_{minor}), 1.78 (dddd, *J* = 18.5, 8.1, 4.0, 1.9 Hz, 1H, d_{major}), 1.57 (ddd, *J* = 12.9, 4.2, 2.4 Hz, 1H, d_{major}), 1.53 – 1.39 (m, 3H, d_{major} and d_{minor}), 1.32 (dt, *J* = 13.1, 11.8 Hz, 1H, d_{minor}), 1.24 – 1.12 (m, 2H, d_{major} and d_{minor}), 0.98 (d, *J* = 6.3 Hz, 3H, d_{minor}), 0.93 (d, *J* = 6.5 Hz, 3H, d_{major}).

^{13}C{H} NMR (176 MHz, $CDCl_3$) δ 139.7 (q, *J* = 0.9 Hz, d_{major}), 139.0 (d_{minor}), 129.0 (2C, d_{minor}), 128.5 (q, *J* = 294.1 Hz, d_{major}), 128.4 (2C, d_{major}), 128.4 (2C, d_{minor}), 128.3 (2C, d_{major}), 127.2 (d_{major}), 127.1(d_{minor}), 126.4 (q, *J* = 282.2 Hz, d_{minor}), 63.4 (q, *J* = 26.1 Hz, d_{minor}), 59.3 (q, *J* = 1.4 Hz, d_{major}), 58.4 (q, *J* = 24.5 Hz, d_{major}), 57.7 (q, *J* = 2.9 Hz, d_{minor}), 51.9 (q, *J* = 1.0 Hz, d_{minor}), 46.0 (q, *J* = 1.0 Hz, d_{major}), 33.2 (q, *J* = 3.0 Hz, d_{minor}), 33.0 (d_{major}), 32.3 (d_{minor}), 32.3 (q, *J* = 1.4 Hz, d_{major}), 30.2 (d_{minor}), 25.7 (q, *J* = 1.1 Hz, d_{major}), 22.7 (d_{major}), 22.1 (d_{minor}).

^{19}F{H} NMR (376 MHz, $CDCl_3$) δ -64.1, -68.8.

HRMS: *m/z* calculated for $C_{14}H_{18}F_3N$ $[M+H]^+$ 258.1470; found 258.1480.

IR (film, cm^{-1}): 2954, 2925, 2848, 1495, 1454, 1372, 1259, 1173, 1145, 1123, 1085, 1028, 981, 741, 724, 697, 636.

The relative stereochemistry of the title compound was determined by 2D NMR experimentation. In the heteronuclear NOESY spectrum only signals arising from protons close to the 19F are observed. For the major component (^{19}F at -64 ppm), clear NOE signals from the fluorine to the benzylic protons (3.98 and 3.84 ppm), C**H**-CF3 proton (3.38 ppm), one of the N-C**H**$_2$ protons (2.93 ppm), one of the ring C**H**$_2$-CH-CF_3 protons (1.96 ppm) and to the CH_3-C**H** proton (1.78 ppm) are observed. This is only possible in the anti-configuration with the CF3 group in the axial position. For the minor component

(^{19}F at -69 ppm), as well as the benzylic (4.19 and 3.26 ppm) and C**H**-CF_3 proton (2.97 ppm), only a strong NOE signal to the protons of the neighbouring CH_2 group, C**H**$_2$-CH-CF_3, (1.87 and 1.32 ppm) is observed. No NOE to the methyl protons is observed. This is only possible in the syn-configuration with the CF_3 in the equatorial position.

3-(1-benzylpiperidin-2-yl)pyridine (10g):

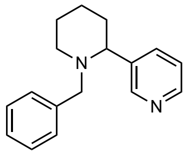

Prepared according to general procedure **6** using 1-benzylpiperidine *N*-oxide (38.2 mg, 0.20 mmol, 1.0 equiv), pivaloyl chloride (62 μL, 0.50 mmol, 2.5 equiv), TBSOTf (35 μL, 0.10 mmol, 75 mol%), copper iodide (19.0 mg, 0.10 mmol, 50 mol%), 3-iodopyridine (102.5 mg, 0.50 mmol, 2.5 equiv), zinc dust (19.6 mg, 0.30 mmol, 1.5 equiv), and indium powder (69.7 mg, 0.60 mmol, 3.0 equiv). Purification by automated flash column chromatography (RediSep Rf Gold Si 4g, 0–10% EtOAc in [3% Et_3N in PE]) delivered the title product as a thin colourless film (7.1 mg, 14%, 0.03 mmol).

^{1}H NMR (500 MHz, $CDCl_3$) δ 8.65 (d, *J* = 2.2 Hz, 1H), 8.49 (dd, *J* = 4.8, 1.7 Hz, 1H), 7.82 (d, *J* = 7.9 Hz, 1H), 7.28 – 7.19 (m, 6H), 3.70 (d, *J* = 13.6 Hz, 1H), 3.17 (dd, *J* = 11.2, 2.8 Hz, 1H), 2.99 (d, *J* = 11.9 Hz, 1H), 2.86 (d, *J* = 13.6 Hz, 1H), 1.96 (td, *J* = 11.6, 3.4 Hz, 1H), 1.83 – 1.75 (m, 2H), 1.65 – 1.53 (m, 3H), 1.43 – 1.36 (m, 1H).

^{13}C{H} NMR (126 MHz, $CDCl_3$) δ 149.6, 148.7, 141.1, 139.4, 135.0, 128.7 (2C), 128.3 (2C), 126.9, 123.8, 66.6, 59.9, 53.4, 37.2, 26.0, 25.1.

HRMS: *m/z* calculated for $C_{17}H_{21}N_2$ $[M+H]^+$ 253.1699; found 253.1700.

IR (film, cm^{-1}): 2931, 2791, 1494, 1451, 1423, 1319, 1129, 1101, 1025, 803, 738, 717, 698.

6-(1-benzylpiperidin-2-yl)nicotinonitrile (10h):

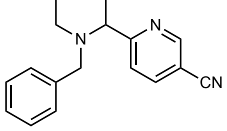

Prepared according to general procedure **6** using 1-benzylpiperidine *N*-oxide (38.2 mg, 0.20 mmol, 1.0 equiv), pivaloyl chloride (62 μL, 0.50 mmol, 2.5 equiv), TBSOTf (35 μL, 0.10 mmol, 75 mol%), copper iodide (19.0 mg, 0.10 mmol, 50 mol%), 6-iodonicotinitrile (115.0 mg, 0.50 mmol, 2.5 equiv), zinc dust (19.6 mg, 0.30 mmol, 1.5 equiv), and indium powder (69.7 mg, 0.60 mmol, 3.0 equiv). Purification by automated flash column

chromatography (RediSep Rf Gold Si 4g, 0–5% EtOAc in [3% Et_3N in PE]) delivered the title product as a pale yellow oil (18.3 mg, 33%, 0.07 mmol).

^{1}H NMR (500 MHz, $CDCl_3$) δ 8.81 (dd, *J* = 2.1, 0.9 Hz, 1H), 7.92 (dd, *J* = 8.2, 2.1 Hz, 1H), 7.77 (dd, *J* = 8.2, 0.9 Hz, 1H), 7.30 – 7.20 (m, 6H), 3.56 (d, *J* = 13.6 Hz, 1H), 3.46 (dd, *J* = 11.1, 3.1 Hz, 1H), 3.05 – 2.99 (m, 2H), 2.02 (td, *J* = 11.8, 3.1 Hz, 1H), 1.88 – 1.80 (m, 2H), 1.67 – 1.49 (m, 3H), 1.41 (tt, *J* = 12.8, 3.7 Hz, 1H).

^{13}C{H} NMR (126 MHz, $CDCl_3$) δ 169.9, 152.1, 139.9, 138.6, 128.7 (2C), 128.4 (2C), 127.1, 121.8, 117.0, 108.2, 70.4, 60.5, 52.9, 35.4, 25.7, 24.6.

HRMS: *m/z* calculated for $C_{18}H_{20}N_3$ $[M+H]^+$ 278.1652; found 278.1654.

IR (film, cm^{-1}): 2933, 2799, 2231, 1593, 1494, 1479, 1451, 1372, 1333, 1102, 1024, 841, 736, 698.

3-(1-benzylpiperidin-2-yl)-6-methylpyridazine (10i):

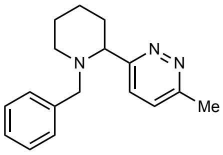

Prepared according to general procedure **6** using 1-benzylpiperidine *N*-oxide (38.2 mg, 0.20 mmol, 1.0 equiv), pivaloyl chloride (62 μL, 0.50 mmol, 2.5 equiv), TBSOTf (35 μL, 0.10 mmol, 75 mol%), copper iodide (19.0 mg, 0.10 mmol, 50 mol%), 3-iodo-6-methylpyridazine (110.0 mg, 0.50 mmol, 2.5 equiv), zinc dust (19.6 mg, 0.30 mmol, 1.5 equiv), and indium powder (69.7 mg, 0.60 mmol, 3.0 equiv). Purification by automated flash column chromatography (RediSep Rf Gold Si 4g, 0–5% EtOAc in [3% Et_3N in PE]) delivered the title product as a colourless oil (11.2 mg, 21%, 0.04 mmol).

^{1}H NMR (500 MHz, $CDCl_3$) δ 7.70 (d, *J* = 8.6 Hz, 1H), 7.29 – 7.18 (m, 6H), 3.74 (dd, *J* = 11.0, 3.1 Hz, 1H), 3.52 (d, *J* = 13.9 Hz, 1H), 3.07 – 2.96 (m, 2H), 2.67 (s, 3H), 2.06 (td, *J* = 11.8, 2.9 Hz, 1H), 1.94 – 1.88 (m, 1H), 1.85 – 1.78 (m, 1H), 1.68 – 1.39 (m, 4H).

^{13}C{H} NMR (126 MHz, $CDCl_3$) δ 164.6, 159.3, 139.1, 128.6 (2C), 128.3 (2C), 127.8, 126.9, 125.2, 67.9, 60.2, 53.1, 35.3, 25.9, 24.6, 22.2.

HRMS: *m/z* calculated for $C_{17}H_{22}N_3$ $[M+H]^+$ 268.1808; found 268.1809.

IR (film, cm^{-1}): 2932, 2854, 2801, 1442, 1323, 1104, 1046, 836, 738, 698.

1-benzyl-2-(perfluorophenyl)-piperidine (10j):

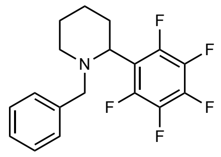

Prepared according to general procedure **6** using 1-benzylpiperidine *N*-oxide (38.2 mg, 0.20 mmol, 1.0 equiv), pivaloyl chloride (62 μL, 0.50 mmol, 2.5 equiv), TBSOTf (35 μL, 0.10 mmol, 75 mol%), copper iodide (19.0 mg, 0.10 mmol, 50 mol%), iodopentafluorobenzene (147.0 mg, 0.50 mmol, 2.5 equiv), zinc dust (19.6 mg, 0.30 mmol, 1.5 equiv), and indium powder (69.7 mg, 0.60 mmol, 3.0 equiv). Purification by automated flash column chromatography (RediSep Rf Gold Si 4g, 0–10% EtOAc in [3% Et_3N in PE]) delivered the title product as a colourless amorphous solid (43.0 mg, 63%, 0.13 mmol).

^{1}H NMR (500 MHz, $CDCl_3$) δ 7.29 – 7.18 (m, 5H), 3.67 (dd, *J* = 11.6, 3.0 Hz, 1H), 3.62 (d, *J* = 13.4 Hz, 1H), 3.05 – 2.94 (m, 2H), 2.07 (qd, *J* = 13.1, 4.0 Hz, 1H), 1.96 – 1.89 (m, 1H), 1.88 – 1.82 (m, 1H), 1.75 – 1.71 (m, 1H), 1.65 – 1.59 (m, 2H), 1.43 – 1.33 (m, 1H).

^{13}C{H} NMR (126 MHz, $CDCl_3$) δ 146.9 – 143.9 (m), 141.5 – 139.1 (m), 138.8 – 136.1 (m), 128.6 (2C), 128.3 (2C), 127.0, 118.0 – 117.0 (m), 60.8, 59.5, 54.2, 31.9, 25.7, 25.0.

^{19}F{H} NMR (376 MHz, $CDCl_3$) δ -140.5, -156.5 (t, *J* = 20.9 Hz), -162.4 (td, *J* = 21.8, 8.2 Hz).

HRMS: *m/z* calculated for $C_{18}H_{17}NF_5$ $[M+H]^+$ 342.1276; found 342.1283.

IR (film, cm^{-1}): 2934, 1520, 1498, 1382, 1126, 1007, 985, 933, 732, 698.

1-benzyl-5-(1-benzylpiperidine-2-yl)-1,2,3,4-tetrahydropyridine (24):

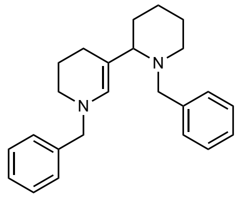

Prepared according to general procedure **2** using 1-benzylpiperidine *N*-oxide (38.2 mg, 0.20 mmol, 1.0 equiv), pivaloyl chloride (62 μL, 0.50 mmol, 2.5 equiv), TBSOTf (23 μL, 0.10 mmol, 50 mol%), copper iodide (19.0 mg, 0.10 mmol, 50 mol%), methyl iodide (38 μL, 0.60 mmol, 3.0 equiv) and zinc dust (39.2 mg, 0.60 mmol, 3.0 equiv). Purification by reverse phase automated flash column chromatography (RediSep Rf 5.5 g C18-column, 0–100% MeCN in H_2O) delivered the title compound as a colourless oil (27.8 mg, 40%, 0.08 mmol).

Spectra are in accordance with the literature data.[44]

***N*-((*Z*)-5-(1-phenyl-2-((1*S*,3*R*,8*S*)-tricyclo[4.3.1.1^{3,8}]undecan-1-yl)piperidin-3-ylidene)pentyl)aniline (25):**

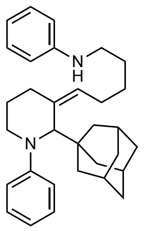

Prepared according to general procedure **3** using 1-phenylpiperidine *N*-oxide (35.5 mg, 0.20 mmol, 1.0 equiv), pivaloyl chloride (62 μL, 0.50 mmol, 2.5 equiv), copper perchlorate (78.2 mg, 0.30 mmol, 1.5 equiv), TBSOTf (23 μL, 0.10 mmol, 50 mol%), copper iodide (19.0 mg, 0.10 mmol, 50 mol%), 1-iodoadamantane (157.0 mg, 0.60 mmol, 3.0 equiv) and zinc dust (39.2 mg, 0.60 mmol, 3.0 equiv). Purification by reverse phase automated flash column chromatography (RediSep Rf 5.5 g C18-column, 0–100% MeCN in H_2O) followed by automated flash column chromatography (RediSep Rf Gold Si 4g, 0–10% EtOAc in PE) delivered the title compound as a colourless oil (15.2 mg, 33%, 0.03 mmol).

^{1}H NMR (700 MHz, $CDCl_3$) δ 7.17 (dt, *J* = 8.6, 7.3 Hz, 4H), 6.90 – 6.82 (m, 2H), 6.68 (tt, *J* = 7.3, 1.1 Hz, 1H), 6.64 (tt, *J* = 7.2, 1.1 Hz, 1H), 6.62 – 6.56 (m, 2H), 5.17 (td, *J* = 7.2, 2.0 Hz, 1H), 3.73 – 3.64 (m, 2H), 3.62 – 3.44 (m, 2H), 3.10 (t, *J* = 7.1 Hz, 2H), 2.64 (dt, *J* = 14.3, 3.9 Hz, 1H), 2.18 (td, *J* = 14.0, 5.3 Hz, 1H), 2.14 – 2.04 (m, 2H), 1.97 (p, *J* = 3.1 Hz, 3H), 1.79 – 1.73 (m, 3H), 1.70 – 1.60 (m, 13H), 1.47 (p, *J* = 7.5 Hz, 2H).

^{13}C{H} NMR (126 MHz, $CDCl_3$) δ 152.4, 148.6, 134.3, 129.4, 129.1, 127.3, 117.2, 116.5, 115.4, 112.8, 74.0, 46.0, 44.0, 42.2, 39.9, 37.2, 29.4, 29.2, 27.6, 26.9, 26.1, 25.0.

HRMS: *m/z* calculated for $C_{32}H_{42}N_2$ $[M+H]^+$ 455.3421; found 455.3414.

IR (film, cm^{-1}): 2900, 2846, 1596, 1501, 1321, 1256, 1178, 985, 746, 691.

1-(2-((3-methyl-4-oxo-2-phenyl-4H-chromene-8-carbonyl)oxy)ethyl)-2,3,4,5-tetrahydropyridin-1-ium chloride (26):

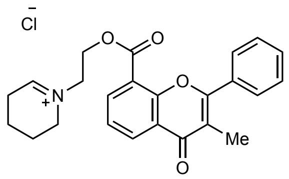

Prepared according to general procedure **1** using flavoxate *N*-oxide (81.4 mg, 0.20 mmol, 1.0 equiv) and pivaloyl chloride (163 μL, 1.32 mmol, 6.6 equiv). After warming to rt, 1,1,2,2-tetrachloroethane (21 μL, 0.20 mmol, 1.0 equiv) was added as an internal standard and the yield was determined *via* quantitative ^{1}H NMR spectroscopy (65%, >20:1 r.r.).

^{1}**H NMR** (500 MHz, CD_2Cl_2) δ 9.05 (s, 1H), 8.36 (dd, *J* = 7.9, 1.8 Hz, 1H), 8.30 (dd, *J* = 7.6, 1.8 Hz, 1H), 7.76 – 7.74 (m, 2H), 7.60 – 7.55 (m, 3H), 7.44 (t, *J* = 7.8 Hz, 1H), 4.82 (t, *J* = 5.0 Hz, 2H), 4.48 – 4.42 (m, 2H), 3.79 – 3.72 (m, 2H), 2.88 – 2.79 (m, 2H), 2.13 (s, 3H), 1.91 – 1.83 (m, 2H), 1.67 – 1.62 (m, 2H).

^{13}**C{H} NMR** (126 MHz, CD_2Cl_2) δ 182.1, 178.0, 163.8, 161.1, 154.9, 136.9, 133.3, 131.6, 131.2, 129.6 (2C), 129.0 (2C), 124.6, 123.5, 119.5, 118.1, 61.6, 61.3, 51.9, 29.5, 20.8, 15.4, 11.8.

HRMS & IR: *compound unstable.*

2-(2-methylpiperidin-1-yl)ethyl 3-methyl-4-oxo-2-phenyl-4H-chromene-8-carboxylate (27a):

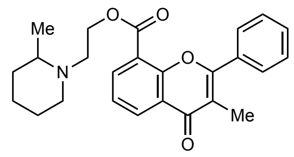

Prepared according to general procedure **4** using flavoxate *N*-oxide (81.4 mg, 0.20 mmol, 1.0 equiv), pivaloyl chloride (62 μL, 0.50 mmol, 2.5 equiv) and 3.4 M methyl magnesium bromide in 2-MeTHF (0.27 mL, 0.60 mmol, 3.0 equiv). Purification by reverse phase automated flash column chromatography (RediSep Rf 5.5 g C18-column, 0–100% MeCN in H_2O) followed by automated flash column chromatography (RediSep Rf Gold Si 4 g, 0–100% EtOAc in PE) delivered the title compound as a colourless oil (20.8 mg, 23%, 0.05 mmol).

^{1}**H NMR** (700 MHz, $CDCl_3$) δ 8.47 (dt, *J* = 7.9, 1.6 Hz, 1H), 8.25 (dt, *J* = 7.5, 1.6 Hz, 1H), 7.82 – 7.75 (m, 2H), 7.57 – 7.51 (m, 3H), 7.47 – 7.42 (m, 1H), 4.45 (t, *J* = 6.5 Hz, 2H), 3.01 (dt, *J* = 13.6, 6.6 Hz, 1H), 2.86 (dt, *J* = 11.6, 3.9 Hz, 1H), 2.73 (dt, *J* = 13.5, 6.4 Hz, 1H), 2.34 (t, *J* = 7.3 Hz, 1H), 2.31 – 2.22 (m, 4H), 1.67 – 1.63 (m, 1H), 1.61 – 1.54 (m, 2H), 1.53 – 1.45 (m, 1H), 1.29 – 1.22 (m, 2H), 1.05 (d, *J* = 6.2 Hz, 3H).

^{13}**C{H} NMR** (176 MHz, $CDCl_3$) δ 178.5, 164.6, 161.2, 154.6, 136.2, 133.2, 130.9, 130.6, 129.5 (2C), 128.6 (2C), 124.1, 123.4, 120.9, 117.8, 63.2, 56.2, 53.4, 52.1, 34.7, 26.3, 24.1, 19.6, 11.9.

HRMS: *m/z* calculated for $C_{25}H_{27}NO_4$ $[M+H]^+$ 406.2013; found 406.2016.

IR (film, cm^{-1}): 2919, 2849, 1725, 1637, 1478, 1439, 1390, 1373, 1262, 1177, 1126, 1023, 760, 735, 699.

2-(2-(methyl-d3)piperidin-1-yl)ethyl 3-methyl-4-oxo-2-phenyl-4H-chromene-8-carboxylate (d_3-27a):

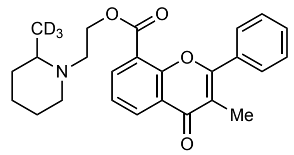

Prepared according to general procedure **4** using flavoxate *N*-oxide (81.4 mg, 0.20 mmol, 1.0 equiv), pivaloyl chloride (62 μL, 0.50 mmol, 2.5 equiv) and 1.0 M methyl-d_3 magnesium iodide in Et_2O (0.60 mL, 0.60 mmol, 3.0 equiv). Purification by reverse phase automated flash column chromatography (RediSep Rf 5.5 g C18-column, 0–100% MeCN in H_2O) delivered the title compound as a yellow oil (20.6 mg, 20%, 0.04 mmol).

^{1}H NMR (500 MHz, $CDCl_3$) δ 8.46 (dd, *J* = 7.9, 1.8 Hz, 1H), 8.25 (dd, *J* = 7.5, 1.8 Hz, 1H), 7.79 (dd, *J* = 7.8, 1.9 Hz, 2H), 7.53 (pd, *J* = 4.9, 1.8 Hz, 3H), 7.45 (t, *J* = 7.8 Hz, 1H), 4.46 (t, *J* = 6.5 Hz, 2H), 3.03 (dt, *J* = 13.5, 6.6 Hz, 1H), 2.91 – 2.85 (m, 1H), 2.75 (dt, *J* = 13.8, 6.4 Hz, 1H), 2.38 – 2.34 (m, 1H), 2.30 (td, *J* = 11.1, 3.4 Hz, 1H), 2.24 (s, 3H), 1.69 – 1.51 (m, 4H), 1.31 – 1.21 (m, 2H).

^{13}C{H} NMR (126 MHz, $CDCl_3$) δ 178.3, 164.4, 161.0, 154.4, 136.1, 133.1, 130.8, 130.5, 129.3 (2C), 128.5 (2C), 124.0, 123.3, 120.7, 117.7, 62.8, 55.9, 53.1, 51.9, 34.2, 25.9, 23.7, 18.4, 11.8.

HRMS: *m/z* calculated for $C_{25}H_{24}NO_4D_3$ $[M+H]^+$ 409.2201; found 409.2203.

IR (film, cm^{-1}): 2926, 2850, 1726, 1623, 1478, 1623, 1478, 1439, 1391, 1263, 1178, 1126, 1023, 759, 699.

2-(2-((benzoyloxy)methyl)piperidin-1-yl)ethyl 3-methyl-4-oxo-2phenyl-4H-chromene-8-carboxylate (27b):

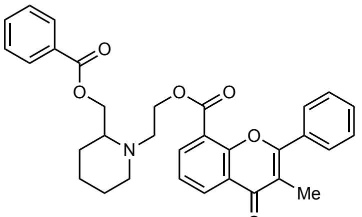

Prepared according to general procedure **2** using flavoxate *N*-oxide (81.4 mg, 0.20 mmol, 1.0 equiv), pivaloyl chloride (62 μL, 0.50 mmol, 2.5 equiv), TBSOTf (35 μL, 0.15 mmol, 75 mol%), copper iodide (15.2 mg, 0.08 mmol, 40 mol%), iodomethyl benzoate (131.0 mg, 0.50 mmol, 2.5 equiv) and zinc dust (33.2 mg, 0.50 mmol, 2.5 equiv). Purification by automated flash column chromatography (RediSep Rf Gold Si 4g, 0–50% EtOAc in PE) delivered the title compound as a colourless oil (25.2 mg, 24%, 0.04 mmol).

^{1}H NMR (500 MHz, $CDCl_3$) δ 8.43 (dd, *J* = 7.9, 1.8 Hz, 1H), 8.21 (dd, *J* = 7.5, 1.8 Hz, 1H), 7.97 (dd, *J* = 8.4, 1.4 Hz, 2H), 7.76 (dd, *J* = 8.1, 1.6 Hz, 2H), 7.54 – 7.47 (m, 4H), 7.41 – 7.33 (m, 3H), 4.48 (t, *J* = 6.3 Hz, 2H), 4.41 (dd, *J* = 11.5, 5.1 Hz, 1H), 4.31 (dd, *J* = 11.5, 5.4 Hz, 1H), 3.13 (dt, *J* = 13.4, 6.4 Hz, 1H), 2.89 (dt, *J* = 14.1, 6.2 Hz, 2H), 2.79 – 2.70 (m, 1H), 2.40 (ddd, *J* = 11.6, 9.4, 3.5 Hz, 1H), 2.22 (s, 3H), 1.75 (dt, *J* = 11.7, 3.7 Hz, 1H), 1.71 – 1.65 (m, 1H), 1.50 (dddd, *J* = 35.3, 22.2, 9.0, 3.9 Hz, 3H), 1.38 – 1.30 (m, 1H).

^{13}C{H} NMR (126 MHz, $CDCl_3$) δ 178.4, 166.5, 164.4, 161.1, 154.6, 136.2, 133.2, 133.1, 130.9, 130.6, 130.2, 129.6 (2C), 129.5 (2C), 128.6 (2C), 128.5 (2C), 124.1, 123.4, 120.8, 117.8, 66.1, 63.1, 59.2, 52.7, 52.5, 29.2, 25.6, 22.9, 11.9.

HRMS: *m/z* calculated for $C_{32}H_{31}NO_6$ $[M+H]^+$ 526.2224; found 526.2230.

IR (film, cm^{-1}): 2958, 2932, 1717, 1624,1478,1440, 1391, 1260, 1111, 1024, 758, 711, 664.

2-(2-fluoromethyl)piperidin-1-yl)ethyl 3-methyl-4-oxo-2-phenyl-4H-chromene-8-carboxylate (27c):

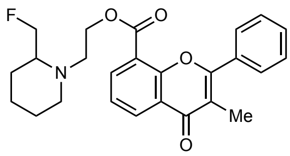

Prepared according to general procedure **2** using flavoxate *N*-oxide (81.4 mg, 0.20 mmol, 1.0 equiv), pivaloyl chloride (62 μL, 0.50 mmol, 2.5 equiv), TBSOTf (34 μL, 0.15 mmol, 75 mol%), copper iodide (15.2 mg, 0.08 mmol, 40 mol%), fluoroiodomethane (35 μL, 0.50 mmol, 2.5 equiv) and zinc dust (33.2 mg, 0.50 mmol, 2.5 equiv). Purification by SCX, followed by automated flash column chromatography (RediSep Rf Gold 4g, 0–100% EtOAc acetate in PE) and then reverse phase automated flash column chromatography (RediSep Rf 5.5 g C18-column, 0–100% MeCN in H_2O) delivered the title compound as a colourless oil (12.1 mg, 16%, 0.03 mmol).

^{1}H NMR (700 MHz, $CDCl_3$) δ 8.47 (d, *J* = 8.0 Hz, 1H), 8.25 (d, *J* = 7.4 Hz, 1H), 7.79 (d, *J* = 8.0 Hz, 2H), 7.54 (t, *J* = 7.6 Hz, 3H), 7.45 (t, *J* = 7.7 Hz, 1H), 4.52 – 4.33 (m, 4H), 3.17 – 3.05 (m, 1H), 2.93 – 2.75 (m, 2H), 2.67 – 2.53 (m, 1H), 2.36 (td, J = 11.1, 3.1 Hz, 1H), 2.24 (s, 3H), 1.70 – 1.52 (m, 4H), 1.50 – 1.43 (m, 1H), 1.35 – 1.26 (m, 2H).

^{13}C{H} NMR (176 MHz, $CDCl_3$) δ 178.3, 164.4, 161.1, 154.5, 136.1, 133.1, 130.8, 130.5, 129.4 (2C), 128.5 (2C), 124.0, 123.3, 120.8, 117.7, 86.1 (d, *J* = 170.6 Hz), 63.0, 60.3 (d, *J* = 17.6 Hz), 53.0, 52.7 (d, *J* = 2.9 Hz), 28.0 (d, *J* = 7.0 Hz), 25.5, 23.3, 11.8.

^{19}F{H} NMR (471 MHz,) δ -219.4.

HRMS: *m/z* calculated for $C_{25}H_{26}NO_4F$ $[M+H]^+$ 424.1919; found 424.1921.

IR (film, cm^{-1}): 3061, 2929, 2854, 1726, 1637, 1601, 1479, 1440, 1391, 1373, 1263, 1178, 1126, 1067, 1024, 760, 699.

2-(2-isopropylpiperidin-1-yl)ethyl 3-methyl-4-oxo-2-phenyl-4H-chromene-8-carboxylate (27d):

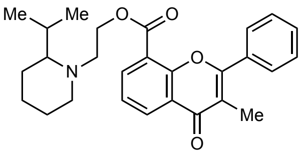

Prepared according to general procedure **2** using flavoxate *N*-oxide (81.4 mg, 0.20 mmol, 1.0 equiv), pivaloyl chloride (62 μL, 0.50 mmol, 2.5 equiv), TBSOTf (34 μL, 0.15 mmol, 75 mol%), copper iodide (9.5 mg, 0.05 mmol, 25 mol%), 2-iodopropane (50 μL, 0.50 mmol, 2.5 equiv) and zinc dust (19.6 mg, 0.30 mmol, 1.5 equiv). Purification by automated flash column chromatography (RediSep Rf Gold Si 4g, 0–100% EtOAc in PE) delivered the title compound as a yellow oil (9.0 mg, 10%, 0.01 mmol).

^{1}H NMR (700 MHz, $CDCl_3$) δ 8.46 (dd, *J* = 7.9, 1.8 Hz, 1H), 8.26 (dd, *J* = 7.5, 1.8 Hz, 1H), 7.83 – 7.78 (m, 2H), 7.56 – 7.52 (m, 3H), 7.44 (t, *J* = 7.7 Hz, 1H), 4.49 – 4.40 (m, 2H), 3.02 (dt, *J* = 13.7, 6.7 Hz, 1H), 2.99 – 2.94 (m, 1H), 2.76 (dt, *J* = 13.5, 6.4 Hz, 1H), 2.35 (ddd, *J* = 11.5, 9.0, 3.0 Hz, 1H), 2.25 (s, 3H), 2.05 – 1.98 (m, 2H), 1.71 (dt, *J* = 8.0, 3.8 Hz, 1H), 1.54 – 1.49 (m, 2H), 1.46 – 1.40 (m, 1H), 1.23 – 1.15 (m, 2H), 0.84 (d, *J* = 6.7 Hz, 3H), 0.80 (d, *J* = 6.6 Hz, 3H).

^{13}C{H} NMR (176 MHz, $CDCl_3$) δ 178.5, 164.6, 161.2, 154.6, 136.2, 133.2, 130.9, 130.6, 129.5 (2C), 128.6 (2C), 124.1, 123.4, 120.9, 117.8, 65.9, 63.0, 53.6, 50.5, 27.6, 25.0, 24.3, 23.7, 20.3, 16.3, 12.0.

HRMS: *m/z* calculated for $C_{27}H_{31}NO_4$ $[M+H]^+$ 434.2326; found 434.2325.

IR (film, cm^{-1}): 2924, 2852, 1727, 1639, 1574, 1478, 1440, 1391, 1262, 1127, 1023, 760, 699.

2-(2-ethylpiperidin-1-yl)ethyl 3-methyl-4-oxo-2-phenyl-4H-chromene-8-carboxylate (27e):

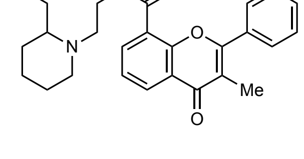

Prepared according to general procedure **4** using flavoxate *N*-oxide (81.4 mg, 0.20 mmol, 1.0 equiv), pivaloyl chloride (62 μL, 0.50 mmol, 2.5 equiv) and 3.0 M ethyl magnesium bromide in Et_2O (0.20 mL, 0.60 mmol, 3.0 equiv). Purification by reverse phase automated

flash column chromatography (RediSep Rf 5.5 g C18-column, 0–100% MeCN in H_2O) delivered the title compound as a yellow oil (6.9 mg, 8%, 0.01 mmol).

^{1}H NMR (700 MHz, $CDCl_3$) δ 8.46 (dd, *J* = 7.9, 1.8 Hz, 1H), 8.25 (dd, *J* = 7.5, 1.8 Hz, 1H), 7.82 – 7.76 (m, 2H), 7.56 – 7.51 (m, 3H), 7.45 (d, *J* = 7.6 Hz, 1H), 4.46 (t, *J* = 6.5 Hz, 2H), 3.01 (dt, *J* = 13.5, 6.6 Hz, 1H), 2.89 (d, *J* = 11.6 Hz, 1H), 2.80 – 2.74 (m, 1H), 2.36 (d, *J* = 11.4 Hz, 1H), 2.26 – 2.22 (m, 4H), 1.66 – 1.50 (m, 5H), 1.39 (dt, *J* = 14.4, 7.5 Hz, 1H), 1.28 (dt, *J* = 14.7, 8.8 Hz, 2H), 0.83 (t, *J* = 7.5 Hz, 3H).

^{13}C{H} NMR (176 MHz, $CDCl_3$) δ 178.3, 164.4, 161.0, 154.5, 136.1, 133.1, 130.8, 130.5, 129.4 (2C), 128.5 (2C), 124.0, 123.3, 120.7, 117.7, 63.0, 61.7, 52.8, 51.1, 29.5, 25.2, 24.1, 23.4, 11.8, 10.0.

HRMS: *m/z* calculated for $C_{26}H_{29}NO_4$ $[M+H]^+$ 420.2170; found 420.2170.

IR (film, cm^{-1}): 2926, 2850, 1726, 1625, 1478, 1440, 1392, 1264, 1178, 1127, 1023, 760, 700.

2-(2-(pyridin-2-yl)piperidin-1-yl)ethyl 3-methyl-4-oxo-2-phenyl-4H-chromene-8-carboxylate (27f):

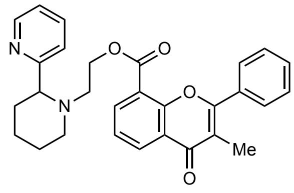

Prepared according to general procedure **6** using flavoxate *N*-oxide (81.4 mg, 0.20 mmol, 1.0 equiv), pivaloyl chloride (62 μL, 0.50 mmol, 2.5 equiv), TBSOTf (35 μL, 0.10 mmol, 75 mol%), copper iodide (19.0 mg, 0.10 mmol, 50 mol%), 2-iodopyridine (53 μL, 0.50 mmol, 2.5 equiv), zinc dust (19.6 mg, 0.30 mmol, 1.5 equiv), and indium powder (69.7 mg, 0.60 mmol, 3.0 equiv). Purification by reverse phase automated flash column chromatography (RediSep Rf 5.5 g C18-column, 0–100% MeCN in H_2O) delivered the title product as a colourless oil (12.9 mg, 14%, 0.02 mmol).

^{1}H NMR (700 MHz, $CDCl_3$) δ 8.49 – 8.43 (m, 2H), 8.20 (d, *J* = 7.4 Hz, 1H), 7.73 (d, *J* = 8.0 Hz, 2H), 7.50 (dd, *J* = 8.4, 4.1 Hz, 3H), 7.45 (dt, *J* = 15.3, 7.6 Hz, 2H), 7.36 (d, *J* = 6.8 Hz, 1H), 7.07 (dd, *J* = 6.8, 5.5 Hz, 1H), 4.42 – 4.35 (m, 1H), 4.32 (dt, *J* = 11.3, 5.8 Hz, 1H), 3.33 (dd, *J* = 11.2, 3.2 Hz, 1H), 3.22 (dt, *J* = 11.7, 3.7 Hz, 1H), 2.77 (dt, *J* = 13.8, 6.9 Hz, 1H), 2.39 (dt, *J* = 13.9, 5.4 Hz, 1H), 2.26 – 2.19 (m, 4H), 1.82 – 1.74 (m, 2H), 1.68 – 1.63 (m, 2H), 1.55 – 1.44 (m, 1H), 1.36 (qt, *J* = 12.5, 4.1 Hz, 1H).

^{13}C{H} NMR (176 MHz, $CDCl_3$) δ 178.4, 164.3, 164.3, 161.2, 154.6, 149.2, 136.6, 136.2, 133.1, 130.9, 130.6, 129.5 (2C), 128.6 (2C), 124.1, 123.4, 122.2, 121.8, 120.9, 117.7, 70.2, 63.2, 54.2, 54.0, 35.3, 26.0, 24.6, 11.9.

HRMS: *m/z* calculated for $C_{29}H_{28}N_2O_4$ $[M+H]^+$ 469.2122; found 469.2124.

IR (film, cm^{-1}): 2931, 2850, 1727, 1637, 1588, 1478, 1439, 1390, 1373, 1263, 1177, 1127, 1024, 759, 699.

*(2S,3S,4S)-4-(5-chloro-2-methoxy-4-pivalamidobenzamido)-1-(3-(4-fluorophenoxy) propyl)-3-methoxy-2-(trifluoromethyl)piperidin-1-ium 2,2,2-trifluoroacetate (**28a**), (2R,3S,4S)-4-(5-chloro-2-methoxy-4-pivalamidobenzamido)-1-(3-(4-fluorophenoxy) propyl)-3-methoxy-2-(trifluoromethyl)piperidin-1-ium 2,2,2-trifluoroacetate (**28b**) and (2R,3R,4S)-4-(5-chloro-2-methoxy-4-pivalamidobenzamido)-1-(3-(4-fluorophenoxy) propyl)-3-methoxy-2-(trifluoromethyl)piperidin-1-ium 2,2,2-trifluoroacetate (**28c**):*

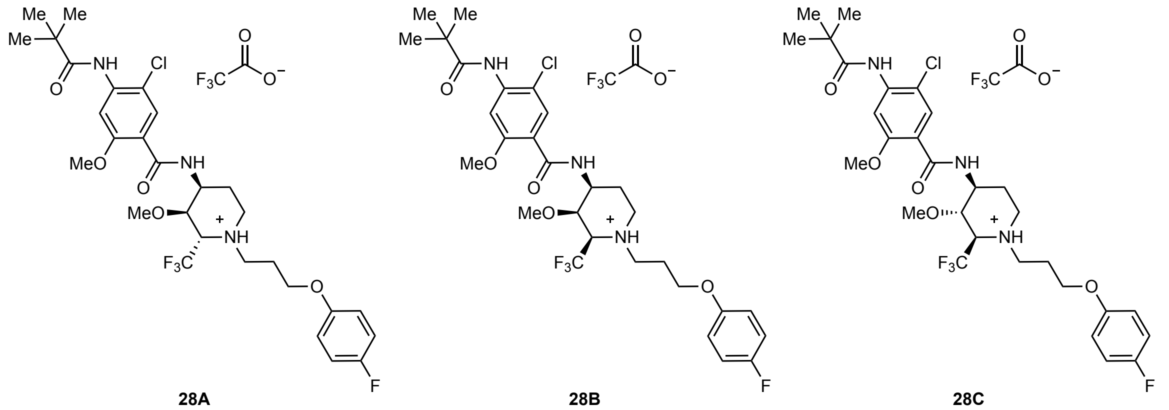

28A **28B** **28C**

Prepared according to general procedure **5** using cisapride *N*-oxide (96.2 mg, 0.20 mmol, 1.0 equiv), pivaloyl chloride (62 μL, 0.50 mmol, 2.5 equiv), MeCN (2 mL, 0.1 M), DMF (47 μL, 0.60 mmol, 3.0 equiv), trimethyl(trifluoromethyl)silane (118 μL, 0.80 mmol, 4.0 equiv), potassium bifluoride (62.5 mg, 0.80 mmol, 4.0 equiv) and trifluoroacetic acid (61 μL, 0.80 mmol, 4.0 equiv). Purification by SCX, followed by reverse phase automated flash column chromatography (RediSep Rf 5.5 g C18-column, 0–100% MeCN in H_2O) and preparative HPLC delivered the three diastereoisomers (total 18.8 mg, 15%, 0.03 mmol, d.r. 2.3:1:1.6).

Major diastereomer (9.2 mg, 7%, 0.01 mmol):

(2S,3S,4S)-4-(5-chloro-2-methoxy-4-pivalamidobenzamido)-1-(3-(4-fluorophenoxy) propyl)-3-methoxy-2-(trifluoromethyl)piperidin-1-ium 2,2,2-trifluoroacetate (28a):

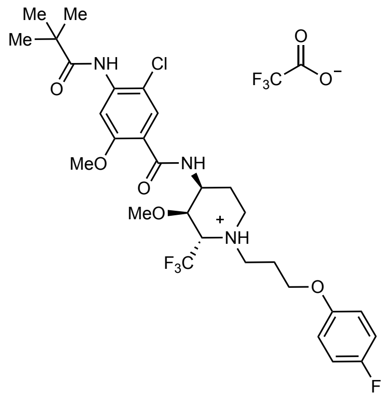

Obtained as a yellow oil.

^{1}H NMR (500 MHz, $CDCl_3$) δ 8.44 (d, *J* = 8.4 Hz, 1H), 8.40 (s, 1H), 8.24 (s, 1H), 8.21 (s, 1H), 7.02 – 6.92 (m, 2H), 6.89 – 6.79 (m, 2H), 4.41 (ddt, *J* = 12.2, 8.5, 4.4 Hz, 1H), 4.09 – 3.96 (m, 5H), 3.68 – 3.59 (m, 2H), 3.38 (s, 3H), 3.13 – 2.98 (m, 3H), 2.90 (d, *J* = 13.1 Hz, 1H), 1.94 (p, *J* = 6.6 Hz, 2H), 1.90 – 1.76 (m, 2H), 1.37 (s, 9H).

^{13}C{H} NMR (126 MHz, $CDCl_3$) δ 177.4, 163.1, 157.2 (d, *J* = 239.4 Hz), 157.0, 155.1 (d, *J* = 2.1 Hz), 138.3, 132.0, 126.2 (q, *J* = 291.3 Hz), 116.9, 115.8 (2C, d, *J* = 23.0 Hz), 115.4 (2C, d, *J* = 7.9 Hz), 114.6, 103.6, 75.2, 65.6, 60.8 (q, *J* = 25.4 Hz), 57.8, 56.3, 51.8, 46.5 (d, *J* = 1.9 Hz), 44.9, 40.5, 27.9, 27.5, 25.0.

^{19}F{H} NMR (376 MHz, $CDCl_3$) δ -66.3, -76.8, -125.2.

HRMS: *m/z* calculated for $C_{29}H_{36}N_3O_5F_4Cl$ $[M+H]^+$ 618.2352; found 618.2354.

IR (film, cm^{-1}): 2923, 2849, 1697, 1649, 1506, 1506,1401, 1250, 1205, 1171, 1133, 829.

The relative stereochemistry of the title compound was determined by 2D NMR experimentation. A HMBC correlation from the **C**H–OMe to the C**H**–CF_3 proton at 3.6 ppm can be observed, hence the CH–OMe and CH–CF_3 subunits must be adjacent to one another. In the heteronuclear NOESY spectrum, only signals arising from protons close to the ^{19}F are observed. A clear NOE signal from the fluorine to a C**H**–NHR proton at 4.42 ppm can be observed, which is only possible if the CF_3 and NHR group are trans to one another. On the basis that the NHR group is expected to be equatorial, axial-axial coupling (approx. 8 Hz) between the C**H**-NHR and the C**H**-OMe would be expected if the OMe and NHR groups were also trans. However, no such coupling is observed, thus the OMe group must be cis to the NHR group.

Minor diastereomer (3.6 mg, 3%, 0.01 mmol):

(2R,3S,4S)-4-(5-chloro-2-methoxy-4-pivalamidobenzamido)-1-(3-(4-fluorophenoxy) propyl)-3-methoxy-2-(trifluoromethyl)piperidin-1-ium 2,2,2-trifluoroacetate (28b):

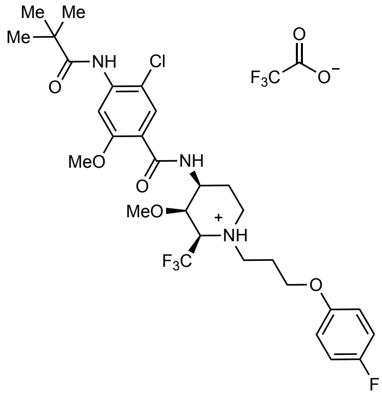

Obtained as a yellow oil.

^{1}H NMR (500 MHz, $CDCl_3$) δ 8.42 (s, 1H), 8.23 (s, 1H), 8.17 (s, 1H), 8.11 (d, *J* = 6.4 Hz, 1H), 7.02 – 6.94 (m, 2H), 6.86 – 6.81 (m, 2H), 4.51 – 4.42 (m, 1H), 4.11 – 3.96 (m, 6H), 3.82 – 3.75 (m, 1H), 3.51 (s, 3H), 3.36 – 3.08 (m, 4H), 2.55 – 2.45 (m, 1H), 2.13 – 2.04 (m, 2H), 1.37 (s, 9H).

^{13}C{H} NMR (126 MHz, $CDCl_3$) δ 177.5, 164.3, 157.4 (d, *J* = 238.7 Hz), 157.2, 154.9 (d, *J* = 2.1 Hz), 138.7, 132.0, 125.8 (q, *J* = 395.9 Hz), 116.6, 116.1 (2C, d, *J* = 23.0 Hz), 115.6 (2C, d, *J* = 8.0 Hz), 114.9, 103.9, 77.4, 65.9, 59.9, 58.7, 56.8, 51.0, 46.4, 45.3, 40.7, 27.6, 26.6.

^{19}F{H} NMR (376 MHz, $CDCl_3$) δ -63.6, -76.7, -124.5.

HRMS: *m/z* calculated for $C_{29}H_{36}N_3O_5F_4Cl$ $[M+H]^+$ 618.2352; found 618.2348.

IR (film, cm^{-1}): 2923, 2850, 1691, 1648, 1505, 1453, 1401, 1248, 1203, 1146, 828, 756.

The relative stereochemistry of the title compound was determined by 2D NMR experimentation. A correlation between the C**H**–OMe peak at 3.77 ppm to the C**H**–CF_3 proton at 4.00 ppm can be observed in the COSY spectrum, hence the C**H**–OMe and C**H**–CF_3 subunits must be adjacent to one another. A clear NOE correlation can be observed between the C**H**–CF_3 and C**H**–OMe, implying they are not axial-axial to each other. As the OMe and NHR groups are expected to cis, and taking into account the stereochemistry of **27a** (see page 127-128), this would imply all three substituents are on the same side of the ring.

Intermediate diastereomer (6.0 mg, 5%, 0.01 mmol):

(2R,3R,4S)-4-(5-chloro-2-methoxy-4-pivalamidobenzamido)-1-(3-(4-fluorophenoxy) propyl)-3-methoxy-2-(trifluoromethyl)piperidin-1-ium 2,2,2-trifluoroacetate (28c):

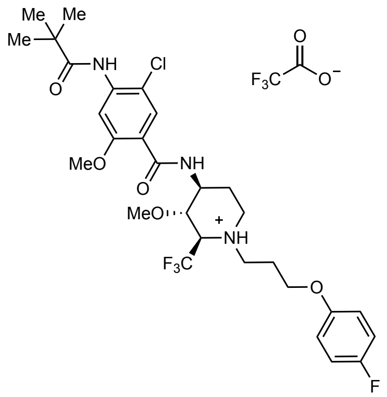

Obtained as a yellow oil.

^{1}H NMR (500 MHz, $CDCl_3$) δ 8.44 (s, 1H), 8.38 (d, *J* = 6.7 Hz, 1H), 8.27 (s, 1H), 8.17 (s, 1H), 7.02 – 6.93 (m, 2H), 6.89 – 6.77 (m, 2H), 4.22 – 4.06 (m, 2H), 4.04 (d, *J* = 12.8 Hz, 5H), 3.94 (dt, *J* = 12.3, 6.7 Hz, 1H), 3.48 (s, 3H), 3.48 – 3.26 (m, 4H), 2.36 – 2.02 (m, 4H), 1.37 (s, 9H).

^{13}C{H} NMR (126 MHz, $CDCl_3$) δ 177.8, 164.8, 157.5 (d, *J* = 238.7 Hz), 157.4, 154.7 (d, *J* = 2.2 Hz), 139.0, 132.0, 124.1 (q, *J* = 285.1 Hz), 116.0 (d, *J* = 23.2 Hz), 116.0, 115.7 (d, *J* = 8.1 Hz), 115.0, 103.9, 74.5, 65.4, 63.6 (q, *J* = 28.0 Hz), 59.3, 56.8, 56.7, 50.7, 49.1, 46.2, 40.7, 27.6, 25.8, 23.2.

^{19}F{H} NMR (376 MHz, $CDCl_3$) δ -66.5, -76.8, -124.4.

HRMS: *m/z* calculated for $C_{29}H_{36}N_3O_5F_4Cl$ $[M+H]^+$ 618.2352; found 618.2350.

IR (film, cm^{-1}): 2957, 2924, 1852, 1649, 1601, 1579, 1505, 1452, 1401, 1248, 1203, 1143, 829, 756.

The relative stereochemistry of the title compound was determined by 2D NMR experimentation and *J*-coupling constant analysis. A correlation between the C**H**–OMe peak at 4.17 ppm to the C**H**–CF_3 proton at 3.95 ppm can be observed in the COSY spectrum, hence the CH–OMe and CH–CF_3 subunits must be adjacent to one another. The C**H**–OMe proton exhibits two large *J*-coupling constants of approximately 6 Hz, which is the result of axial-axial coupling to both the C**H**–CF_3 and C**H**–NHR protons. Consequently, the OMe substituent must sit on the opposite face to the CF_3 and NHR substituents.

5.7 Problematic substrates

While the reaction developed and outlined in this report was found to tolerate a wide range of complex functionality, we were also able to identify certain functional groups that were not tolerated well.

With respect to the alkylamine component in this reaction (Figure S1), morpholine, piperazine and tetrahydroquinoline failed to produce the desired *endo*-cyclic iminium ions from the corresponding *N*-oxides. In the presence of adjacent electron-donating centres (such as a double bond, aromatic ring, or heteroatom), it is well precedented that C_α–C fragmentation may occur as the predominant pathway over the desired α-C–H elimination. [8c,11] Moreover, the morpholine *endo*-cyclic iminium ion is known to react further in a Pictet-Spengler mechanism to form a fused ring system.[8e] Changing the *N*-substituent had little impact on the outcome of iminium ion formation. Surprisingly, in the case of 1-benzyltetrahydroquinoline *N*-oxide and 2-(piperidin-1-ylmethyl)pyridine *N*-oxide, only starting material could be detected. Furthermore, *N*-benzyl pyrrolidine and azepane afforded no *endo*/*exo* selectivity during iminium ion formation and, as a result, delivered a mixture of the *endo* and *exo* α-alkylated products in good yield. It was initially reasoned that the presence of a reactive benzylic *N*-substituent was facilitating the formation of the undesired *exo*-iminium ion in these ring systems. However, changing the *N*-substitutent to -cyclohexyl or -3-phenylpropyl did not improve the observed regioselectivity of iminium ion formation in pyrrolidine systems.

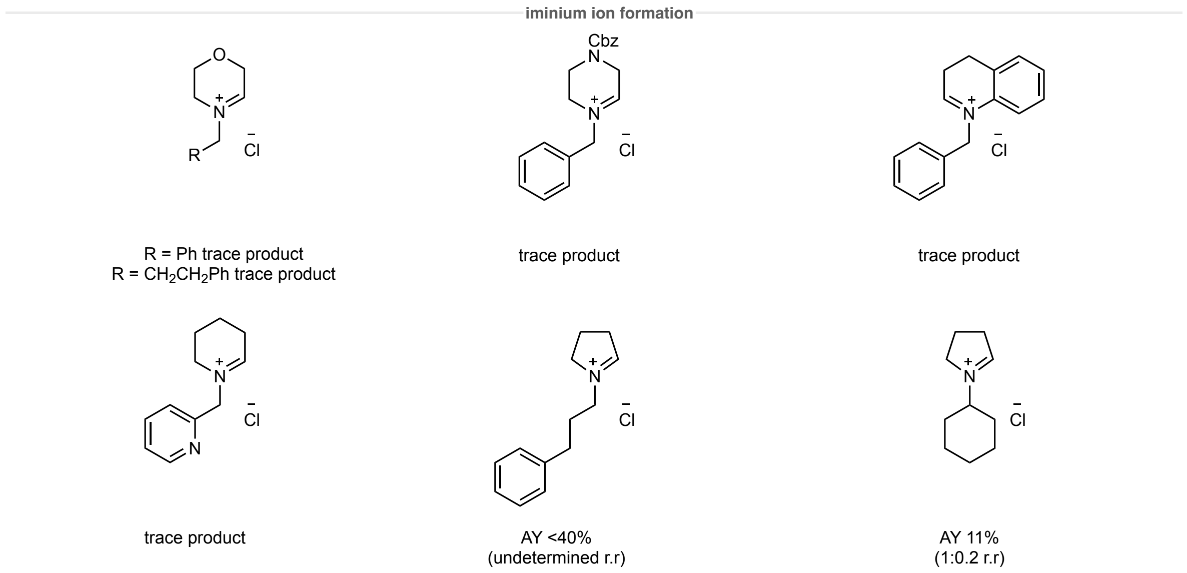

Figure S1: Unproductive or poorly performing cyclic tertiary amine *N*-oxides

For the synthesis of α-alkylated cyclic alkylamines (Figure S2A), the corresponding *endo*-cyclic iminium ion of 1-benzylpiperidine was found to couple effectively with most alkyl iodide partners. In the case of methyl iodide, the corresponding α-alkylated product **6w** was not observed. Instead, competitive dimerization of the intermediate iminium ion, forming **24**, was a major competing side reaction, and despite significant efforts to optimise the desired α-methylation reaction for this substrate (Table **S19**), we were

unable to achieve this transformation using the Zn mediated α-alkylation platform. Increasing the equivalents of each reagent, in addition to varying other reaction parameters, had no effect on the yield of **6w**. Attempted reactions using redox active ester **42,** a known alkyl iodide alternative, and methylzinc iodide (**43**), the active methylating reagent suspected of forming during this reaction, as the 'methyl' source also did not produce any of the desired product (**6w**).[9e] Other alkyl iodides, such as difluoroiodomethane, chloroiodomethane and 1-iodo-2,2,2,-trifluoroethane, were also found not to participate effectively in the α-alkylation reaction. In the case of the former, the polarity mismatch between the electrophilic iminium ion and alkyl radical may explain the poor performance of this substrate.[9a] Trifluoroacetate protected 1-iodo-2-aminoethane also did not produce any of the desired α-alkylated product.

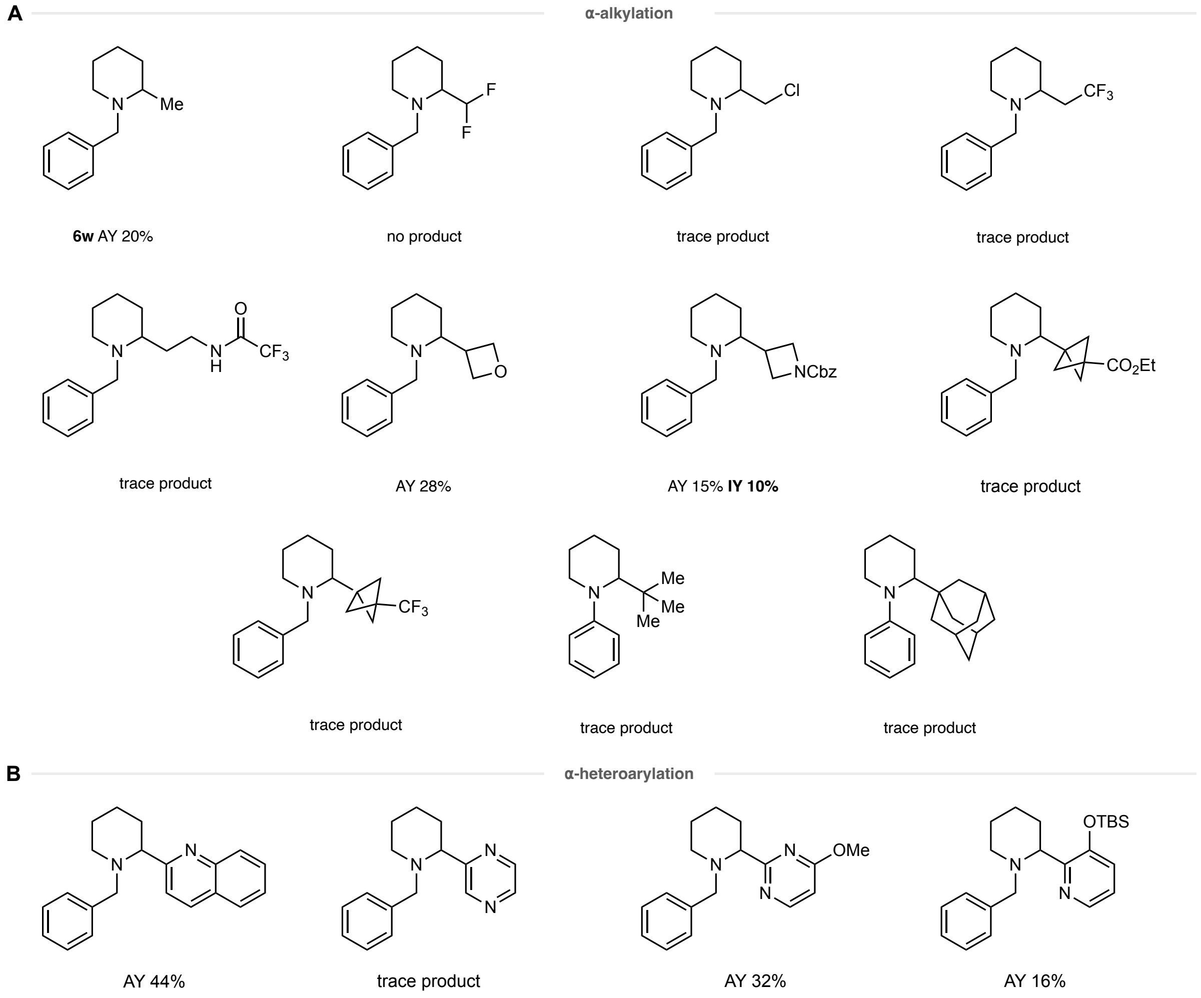

Figure S2: Unproductive or poorly performing A) alkyl iodides and B) heteroaryliodides

Employing 4-membered heterocycles, such as oxetane and azetadine, as the alkyl iodide component did not produce any of the desired α-alkylated products. Instead, visual decomposition of the crude reaction mixture and competitive protodeiodination were observed respectively. Using bicyclo[1.1.1]pentane derivatives as the alkyl iodide component also failed to deliver the desired α-alkylated products; formation of **24** was observed as the major byproduct from these reactions.

Although 1-phenylpiperidine *N*-oxide (**2e**) formed the corresponding *endo*-cyclic iminium ion (**3e**) in 65% assay yield, only trace product could be detected when coupling with *tert*-butyl iodide or 1-iodoadamantane under the Zn mediated α-alkylation reaction conditions, and substantial amounts of a dimeric by-product was isolated instead (**25**).

Iodoazines other than pyridine also performed poorly in the α-heteroarylation reaction (Figure S2B), with competitive protodeiodination of the idodoazine being the major by-product detected. In the case of iodopyrazine, visual decomposition was observed upon concentration of the crude reaction mixture, limiting the assay yield and preventing all attempts at isolation.

Table S19: optimisation efforts for α-methylation using the Zn mediated alkylation reaction conditions

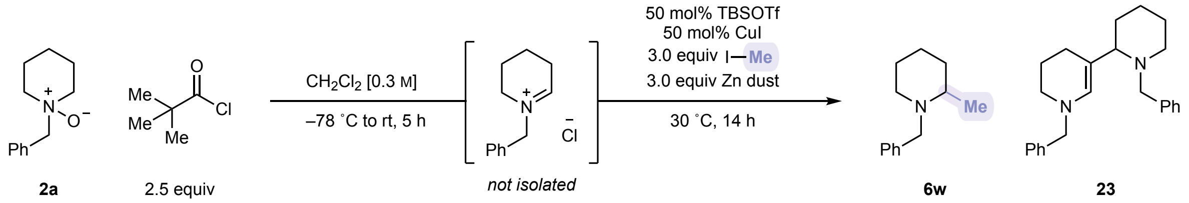

Entry	Deviation from standard conditions	'Methyl' source	6w / %[a]	23 / %[a]
**1**	*none*	Methyl iodide	20	40
**2**	5 equiv MeI	Methyl iodide	17	34
**3**	5 equiv Zn	Methyl iodide	20	28
**4**	100 mol% CuI	Methyl iodide	12	38
**5**	24 h	Methyl iodide	21	26
**6[b]**	1-(4-phenyl-benzyl)-piperidine *N*-oxide	Methyl iodide	16	32
**7[c]**	-	Me redox active ester (**42**)	15	18
**8**	-	Methyl zinc iodide (**43**)	0	28

Reactions carried out using 1.0 equiv of 1-benzylpiperidine *N*-oxide **2a** and 2.5 equiv of PivCl under the standard conditions for tertiary alkyl iodides outlined in general procedure 2. [a]Assay yields of **6w** and **24** were determined by ^{1}H NMR using 1,1,2,2-tetrachloroethane as an internal standard. [b]1-(4-phenyl-benzyl)-piperidine *N*-oxide was used as the amine component instead of **2a**. [c]75 mol% TBSOTf, 40 mol% CuI and 2.5 equiv of **42** and 1.5 equiv Zn were used.

6. Starting material synthesis

6.1 Tertiary amines

N-(3-phenylpropy)piperidine (**1b**),[45] *N*-cyclohexylpiperidine (**1d**),[46] *N*-benzyl-2-methylpiperidine (**1f**),[40] *N*-benzyl-4-methylpiperidine (**1h**),[47] *N*-benzyl morpholine (**1i**),[48] Benzyl 4-benzylpiperazine-1-carboxylate (**1j**),[49] N-benzylpyrrolidine (**1k**),[50] *N*-benzylazepan (**1l**),[51] *N*-benzyl-1,2,3,4-tetrahydroisoquinoline (**1m**),[39] *N*-benzyl-1,2,3,4-tetrahydroquinoline (**1n**),[52] 2-(piperidin-1-ylmethyl)pyridine (**1o**),[53,54] *N*-(3-phenylpropyl)pyrrolidine (**1p**),[55] *N*-cyclohexylpyrrolidine (**1q**),[46] *N*-(4-phenylbenzyl) -2-methylpiperidine (**1s**),[40] and flavoxate (**32**)[56] were synthesized according to literature procedures.

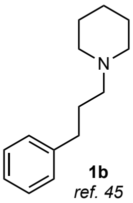

1b
ref. 45

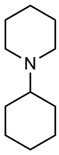

1d
ref. 46

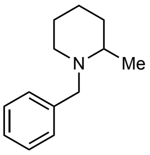

1f
ref. 40

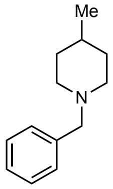

1h
ref. 47

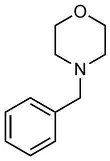

1i
ref. 48

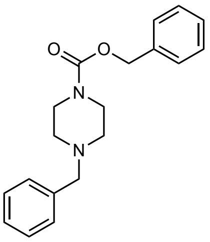

1j
ref. 49

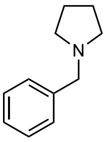

1k
ref. 50

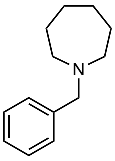

1l
ref. 51

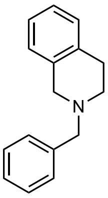

1m
ref. 39

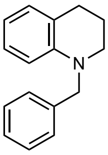

1n
ref. 52

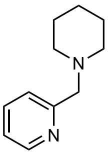

1o
ref. 53/54

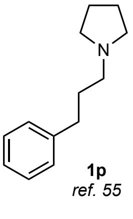

1p
ref. 55

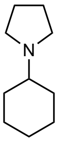

1q
ref. 46

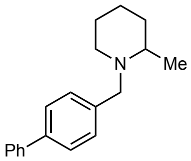

1s
ref. 40

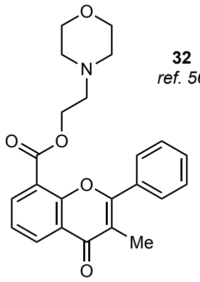

32
ref. 56

1-benzyl-3-methylpiperidine (1g):

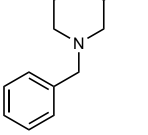

To a mixture of 3-methylpiperidine (3.5 mL, 30.0 mmol, 1.0 equiv) and benzyl bromide (3.6 mL, 30.0 mmol, 1.0 equiv) in ethanol (60 mL, 0.5 M) was added KOH (1.85 g, 33.0 mmol, 1.1 equiv). The mixture was stirred at rt for 24 h, then the solvent was removed

in vacuo. The residue was redissolved in water (40 mL) and extracted with EtOAc (40 mL then 3 x 20 mL). The combined organic layers were washed with brine (3 x 30 mL), dried over Na_2SO_4, filtered, and concentrated *in vacuo*. This delivered the title compound as a yellow oil (5.21 g, 92%, 27.6 mmol).

Spectra are in accordance with the literature data. [57]

1-(4-phenylbenzyl)piperidine (1r):

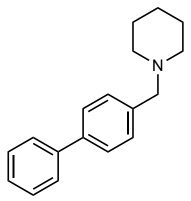

To a mixture of piperidine (2.0 mL, 20.0 mmol, 1.0 equiv) and 4-bromomethylbiphenyl (4.90 g, 20.0 mmol, 1.0 equiv) in MeCN (60 mL, 0.3 M) was added KOH (1.20 g, 22.0 mmol, 1.1 equiv). The mixture was stirred at rt for 24 h, then the solvent was removed *in vacuo*. The residue was redissolved in H_2O (100 mL) and extracted with EtOAc (100 mL then 3 x 75 mL). The combined organic layers were washed with brine (3 x 75 mL), dried over Na_2SO_4, filtered, and concentrated *in vacuo*. Purification by automated flash column chromatography (RediSep Rf Silver Si 40 g, 0–50% EtOAc in PE) delivered the title compound as a pale yellow oil (2.00 g, 40%, 8.0 mmol).

Spectra in accordance with the literature data.[58]

Dextromethorphan (29):

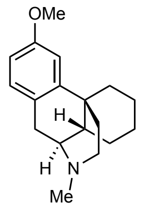

Prepared according to the literature procedure by Matosiuk and co-workers.[59]

^{1}H NMR (700 MHz, $CDCl_3$) δ 7.02 (d, *J* = 8.4 Hz, 1H), 6.80 (d, *J* = 2.7 Hz, 1H), 6.69 (dd, *J* = 8.4, 2.7 Hz, 1H), 3.78 (s, 3H), 2.98 (d, *J* = 18.0 Hz, 1H), 2.79 (dd, *J* = 5.8, 3.1 Hz, 1H), 2.57 (ddd, *J* = 18.0, 5.8, 1.2 Hz, 1H), 2.45 – 2.36 (m, 4H), 2.37 – 2.31 (m, 1H), 2.07 (td, *J* = 12.3, 3.3 Hz, 1H), 1.81 (dt, *J* = 12.8, 3.2 Hz, 1H), 1.73 (td, *J* = 12.7, 4.8 Hz, 1H), 1.64 (dt, *J* = 13.8, 2.6 Hz, 1H), 1.55 – 1.48 (m, 1H), 1.44 – 1.25 (m, 5H), 1.13 (qd, *J* = 12.5, 3.8 Hz, 1H).

^{13}C{H} NMR (176 MHz, $CDCl_3$) δ 158.3, 141.9, 130.1, 128.6 (2C), 111.2 (2C), 110.8, 58.1, 55.3, 47.4, 45.6, 43.0, 42.3, 37.4, 36.8, 27.0, 26.7, 23.5, 22.4.

HRMS: *m/z* calculated for $C_{18}H_{25}NO$ $[M+H]^+$ 272.2009; found 272.2006.

IR (film, cm^{-1}): 2924, 2852, 2796, 1608, 1575, 1494, 1445, 1280, 1237, 1156, 1044, 850, 800, 759.

Cloperastine (30):

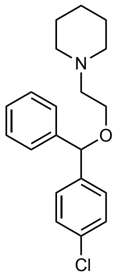

To a solution of cloperastine hydrochloride (2.00 g, 5.46 mmol, 1.0 equiv) in Et_2O (36 mL, 0.15 M) was added 10% NaOH solution (22 mL, 54.60 mmol, 10.0 equiv) and water (8 mL, 0.16 M) under air at rt. The reaction mixture was stirred at rt for 1.5 h. The reaction mixture was diluted with EtOAc (60 mL), extracted with H_2O (3 x 100 mL), and then washed with brine (100 mL). The organic layer was dried over $MgSO_4$, filtered and concentrated *in vacuo* to afford cloperastine as a pale yellow oil (1.45 g, 81%, 4.40 mmol).

^{1}H NMR (500 MHz, $CDCl_3$) δ 7.35 – 7.23 (m, 9H), 5.35 (s, 1H), 3.58 (td, *J* = 6.3, 1.1 Hz, 2H), 2.64 (t, *J* = 6.3 Hz, 2H), 2.43 (br s, 4H), 1.61 – 1.52 (m, 4H), 1.49 – 1.36 (m, 2H).

^{13}C{H} NMR (126 MHz, $CDCl_3$) δ 142.0, 141.1, 133.2, 128.6 (2C), 128.6 (2C), 128.5 (2C), 127.8, 127.1 (2C), 83.3, 67.3, 58.7, 55.2 (2C), 26.2 (2C), 24.4.

HRMS: *m/z* calculated for $C_{20}H_{24}NOCl$ $[M+H]^+$ 330.1619; found 330.1626.

IR (film, cm^{-1}): 2931, 2852, 2783, 1488, 1452, 1302, 1087, 1014, 850, 796, 756, 699.

Ticlopidine (31):

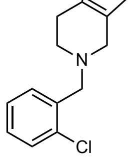

To a solution of ticlopidine hydrochloride hydrate (1.00 g, 3.30 mmol, 1.0 equiv) in $CHCl_3$ (5 mL, 0.7 M) was added 10% NaOH solution (3 mL). The reaction was stirred at rt for 2 h. The organic layer was separated, dried over Na_2SO_4, and concentrated *in vacuo* to afford dextromethorphan as a colourless oil (0.88 g, 100%, 3.30 mmol).

^{1}**H NMR** (700 MHz, $CDCl_3$) δ 7.55 (dd, *J* = 7.6, 1.8 Hz, 1H), 7.37 (dd, *J* = 7.9, 1.4 Hz, 1H), 7.25 (dd, *J* = 7.5, 1.4 Hz, 1H), 7.20 (td, *J* = 7.6, 1.8 Hz, 1H), 7.08 (d, *J* = 5.1 Hz, 1H), 6.72 (d, *J* = 5.1 Hz, 1H), 3.83 (s, 2H), 3.65 (t, *J* = 1.7 Hz, 2H), 2.95 – 2.83 (m, 4H).

^{13}**C{H} NMR** (176 MHz, $CDCl_3$) δ 136.3, 134.4, 134.1, 133.6, 130.8, 129.6, 128.3, 126.9, 125.4, 122.8, 58.6, 53.3, 50.9, 25.7.

HRMS: *m/z* calculated for $C_{14}H_{14}NSCl$ $[M+H]^+$ 264.0608; found 264.0609.

IR (film, cm^{-1}): 2920, 2797, 1441, 1356, 1320, 1167, 1048, 1036, 1015, 839, 752, 701.

6.2 Amine *N*-oxides

1-benzylpiperidine N-oxide (2a):

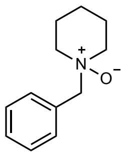

Prepared according to general procedure **7** using 1-benzylpiperidine (3 mL, 16.0 mmol, 1.0 equiv), *m*-CPBA (4.72 g, 19.1 mmol, 1.2 equiv) and anhydrous K_2CO_3 (9.30 g, 67.1 mmol, 4.5 equiv). Purification by automated flash column chromatography (RediSep Rf Silver Si 40 g, 0–20% MeOH in $CHCl_3$) delivered the title compound as a white powder (3.06 g, 100%, 16.0 mmol).

Spectra in accordance with the literature data. [28]

1-(3-phenylpropyl)piperidine N-oxide (2b):

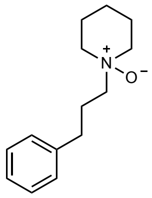

Prepared according to general procedure **7** using crude *1*-(3-phenylpropyl)piperidine (1.36 g, 6.8 mmol, 1.0 equiv), *m*-CPBA (1.40 g, 8.1 mmol, 1.2 equiv) and anhydrous K_2CO_3 (3.71 g, 26.9 mmol, 4.5 equiv). Purification by automated flash column chromatography (RediSep Rf Silver Si 40 g, 0–20% MeOH in $CHCl_3$) delivered the title compound as an off-white powder (0.86 g, 58%, 3.9 mmol).

Spectra are in accordance with the literature data.[60]

1-methylpiperidine N-oxide (2c):

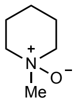

Prepared according to general procedure **7** using 1-methylpiperidine (5.0 mL, 41.1 mmol, 1.0 equiv), *m*-CPBA (12.18 g, 49.4 mmol, 1.2 equiv) and anhydrous K_2CO_3 (25.60 g, 185 mmol, 4.5 equiv). Purification by automated flash column chromatography (RediSep Rf Silver Si 40 g, 0–20% MeOH in $CHCl_3$) delivered the title compound as a brown oil (2.67 g, 56%, 23.0 mmol).

Spectra are in accordance with the literature data. [61]

1-cylcohexylpiperidine N-oxide (2d):

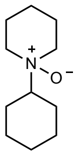

1-cyclohexylpiperidine *N*-oxide was prepared according to general procedure **7** using crude *1*-cyclohexylpiperidine (0.97 g, 5.8 mmol, 1.0 equiv), *m*-CPBA (1.40 g, 8.1 mmol, 1.2 equiv) and anhydrous K_2CO_3 (3.71 g, 26.9 mmol, 4.5 equiv). Purification by automated flash column chromatography (RediSep Rf Silver Si 12 g, 0–20% MeOH in $CHCl_3$) delivered the title compound as an amorphous pale pink solid (0.77 g, 73%, 4.2 mmol).

^{1}H NMR (500 MHz, $CDCl_3$) δ 3.03 (m, 4H), 2.91 (tt, *J* = 11.9, 3.4 Hz, 1H), 2.52 – 2.28 (m, 4H), 1.96 – 1.81 (m, 2H), 1.80 – 1.67 (m, 1H), 1.63 (m, 1H), 1.58 – 1.37 (m, 4H), 1.32 – 1.18 (m, 3H), 1.09 (qt, *J* = 13.1, 3.8 Hz, 1H).

^{13}C{H} NMR (126 MHz, $CDCl_3$) δ 78.5, 61.4 (2C), 26.6 (2C), 25.9 (2C), 25.6, 22.9, 20.8 (2C).

HRMS: *m/z* calculated for $C_{11}H_{22}NO$ $[M+H]^+$ 184.1696; found 184.1697.

IR (solid, cm^{-1}): 3014, 2935, 2918, 2852, 1442, 1355.

1-phenylpiperidine N-oxide (2e):

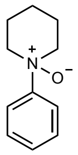

Prepared according to general procedure **7** using 1-phenylpiperidine (2.00 g, 12.4 mmol, 1.0 equiv), *m*-CPBA (3.67 g, 14.9 mmol, 1.2 equiv) and anhydrous K_2CO_3 (7.71 g, 55.8 mmol, 4.5 equiv). Purification by automated flash column chromatography (RediSep Rf Silver Si 12 g, 0–20% MeOH in $CHCl_3$) delivered the title compound as an amorphous white solid (2.02 g, 92%, 11.4 mmol).

^{1}H NMR (500 MHz, $CDCl_3$) δ 8.04 – 7.98 (m, 2H), 7.48 – 7.40 (m, 2H), 7.39 – 7.32 (m, 1H), 3.64 (ddd, *J* = 12.6, 11.4, 3.0 Hz, 2H), 3.19 (m, 2H), 2.76 – 2.63 (m, 2H), 1.93 – 1.83 (m, 1H), 1.65 (m, 2H), 1.40 (qt, *J* = 13.3, 4.0 Hz, 1H).

^{13}C{H} NMR (101 MHz, $CDCl_3$) δ 155.6, 129.1 (2C), 128.8 (2C), 120.5, 68.8 (2C), 22.0 (2C), 21.3.

HRMS: *m/z* calculated for $C_{11}H_{16}NO$ $[M+H]^+$ 178.1226; found 178.1229.

IR (solid, cm^{-1}): 3047, 2926, 2861, 1590, 1488, 1433, 1016, 934, 756.

1-benzyl-2-methylpiperidine N-oxide (2f):

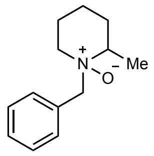

Prepared according to general procedure **7** using 1-benzyl-2-methylpiperidine (1.42 g, 7.5 mmol, 1.0 equiv), *m*-CPBA (2.22 g, 9.0 mmol, 1.2 equiv) and anhydrous K_2CO_3 (4.70 g, 33.8 mmol, 4.5 equiv). Purification by automated flash column chromatography (RediSep Rf Silver Si 40 g, 0–20% MeOH in $CHCl_3$) delivered the title compound as an amorphous brown solid (0.89 g, 58%, 4.4 mmol).

^{1}H NMR (500 MHz, $CDCl_3$) δ 7.56 – 7.43 (m, 2H), 7.44 – 7.36 (m, 3H), 4.50 (d, *J* = 13.1 Hz, 1H), 4.35 (d, *J* = 13.1 Hz, 1H), 3.21 – 3.12 (m, 2H), 3.03 – 2.92 (m, 1H), 2.34 (q, *J* = 13.5 Hz, 1H), 2.21 – 2.08 (m, 1H), 1.67 (dt, *J* = 13.1, 4.1 Hz, 1H), 1.59 – 1.41 (m, 5H), 1.19 (qt, *J* = 13.2, 4.0 Hz, 1H).

^{13}C{H} NMR (126 MHz, $CDCl_3$): δ 132.2 (2C), 130.7, 129.6, 128.9 (2C), 72.7, 66.3, 64.3, 29.0, 22.9, 20.5, 15.9.

HRMS: *m/z* calculated for $C_{13}H_{19}NO$ $[M+H]^+$ 206.1467; found 206.1539.

IR (solid, cm^{-1}): 3045, 2974, 2940, 1496, 1450, 1355, 1073, 1032, 964, 895.

1-benzyl-3-methylpiperidine N-oxide (2g):

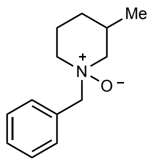

Prepared according to general procedure **7** using 1-benzyl-3-methylpiperidine (5.21 g, 27.6 mmol, 1.0 equiv), *m*-CPBA (8.14 g, 33.0 mmol, 1.2 equiv) and anhydrous K_2CO_3 (17.1 g, 124.0 mmol, 4.5 equiv). Purification by automated flash column chromatography (RediSep Rf Silver Si 40 g, 0–20% MeOH in $CHCl_3$) delivered the title compound as an amorphous brown solid (4.03 g, 71%, 19.6 mmol).

^{1}H NMR (500 MHz, $CDCl_3$) δ 7.52 – 7.45 (m, 2H), 7.39 – 7.32 (m, 3H), 4.32 (s, 2H), 3.09 – 2.89 (m, 3H), 2.74 – 2.26 (m, 3H), 1.72 (m, 1H), 1.53 (m, 1H), 0.90 – 0.73 (m, 4H).

^{13}C{H} NMR (126 MHz, $CDCl_3$) δ 132.6 (2C), 130.1, 129.4, 128.5 (2C), 77.4, 70.4, 63.8, 31.1, 26.2, 20.4, 18.7.

HRMS: *m/z* calculated for $C_{13}H_{19}NO$ $[M+H]^+$ 206.1539; found 206.1543.

IR (solid, cm^{-1}): 2950, 2839, 2495, 1454, 949, 894, 730.

1-benzyl-4-methylpiperidine N-oxide (2h):

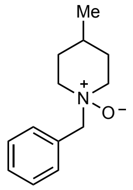

Prepared according to general procedure **7** using 1-benzyl-4-methylpiperidine (1.77 g, 9.4 mmol, 1.0 equiv), *m*-CPBA (2.77 g, 11.2 mmol, 1.2 equiv) and anhydrous K_2CO_3 (5.81 g, 42.1 mmol, 4.5 equiv). Purification by automated flash column chromatography (RediSep Rf Silver Si 24 g, 0–20% MeOH in $CHCl_3$) delivered the title compound as an amorphous white solid (1.28 g, 67%, 6.2 mmol).

^{1}H NMR (500 MHz, $CDCl_3$) δ 7.54 – 7.44 (m, 2H), 7.44 – 7.32 (m, 3H), 4.34 (s, 2H), 3.27 – 2.85 (m, 4H), 2.15 (dtd, *J* = 13.9, 12.4, 4.1 Hz, 2H), 1.49 (ddt, *J* = 13.9, 3.6, 1.5 Hz, 2H), 1.43 – 1.28 (m, 1H), 0.96 (d, *J* = 6.5 Hz, 3H).

^{13}C{H} NMR (126 MHz, $CDCl_3$) δ 132.6 (2C), 130.3, 129.5, 128.6 (2C), 76.8, 64.0, 29.4, 28.6, 21.3.

HRMS: *m/z* calculated for $C_{13}H_{19}NO$ $[M+H]^+$ 206.1539; found 206.1544.

IR (solid, cm^{-1}): 2952, 2924, 1456, 1347, 993, 926, 767, 713, 641, 505.

1-benzyl morpholine N-oxide (2i):

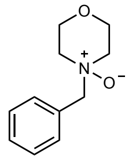

Prepared according to general procedure **7** using 1-benzyl morpholine (4.10 g, 23.1 mmol, 1.0 equiv), *m*-CPBA (4.78 g, 27.7 mmol, 1.2 equiv) and anhydrous K_2CO_3 (12.77 g, 92.4 mmol, 4.5 equiv). Purification by automated flash column chromatography (RediSep Rf Silver Si 40 g, 0–20% MeOH in $CHCl_3$) delivered the title compound as an amorphous pale yellow solid (3.60 g, 81%, 18.6 mmol).

Spectra in accordance with literature data. [62]

Benzyl 4-benzylpiperazine-1-carboxylate N-oxide (2j):

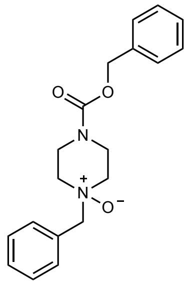

Prepared according to general procedure **7** using crude benzyl 4-benzylpiperazine-1-carboxylate (1.01 g, 3.3 mmol, 1.0 equiv), *m*-CPBA (0.67 g, 3.9 mmol, 1.2 equiv) and anhydrous K_2CO_3 (1.80 g, 13.0 mmol, 4.5 equiv). Purification by automated flash column chromatography (RediSep Rf Silver Si 12 g, 0–20% MeOH in $CHCl_3$) delivered the title compound as an amorphous white solid (0.73 g, 69%, 2.2 mmol).

^{1}H NMR (500 MHz, $CDCl_3$) δ 7.53 – 7.26 (m, 10H), 5.08 (s, 2H), 4.34 (s, 2H), 3.95 (m, 4H), 3.14 (br s, 2H), 2.97 (br s, 2H).

^{13}C{H} NMR (126 MHz, $CDCl_3$) δ 154.7, 136.2, 132.5 (2C), 129.8, 129.2, 128.8 (2C), 128.6 (2C), 128.3, 128.1 (2C), 77.4, 67.7, 63.2 (2C), 38.5 (2C).

HRMS: m/z calculated for $C_{19}H_{23}N_2O_3$ $[M+H]^+$ 327.1703; found 327.1705.

IR (solid, cm^{-1}): 2942, 1693, 1466, 1426 , 1260, 1244, 1149, 1072, 914, 755.

1-benzylpyrrolidine (2k):

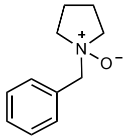

1-benzylpyrrolidine *N*-oxide was prepared according to general procedure **7** using crude 1-benzylpyrrolidine (3.15 g, 19.6 mmol, 1.0 equiv), *m*-CPBA (4.04 g, 23.4 mmol, 1.2 equiv) and anhydrous K_2CO_3 (10.78 g, 78.0 mmol, 4.5 equiv). Purification by automated flash column chromatography (RediSep Rf Silver Si 40 g, 0–20% MeOH in $CHCl_3$) delivered the title compound as an amorphous pale brown solid (2.23 g, 65%, 12.7 mmol).

Spectra are in accordance with the literature data.[63]

1-benzylazepan N-oxide (2l):

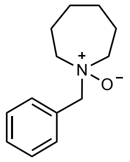

Prepared according to general procedure **7** using crude 1-benzylazepan (2.49 g, 13.1 mmol, 1.0 equiv), *m*-CPBA (2.73 g, 15.8 mmol, 1.2 equiv) and anhydrous K_2CO_3 (7.30 g, 52.8 mmol, 4.5 equiv). Purification by automated flash column chromatography (RediSep Rf Silver Si 24 g, 0–20% MeOH in $CHCl_3$) delivered the title compound as an amorphous pale orange solid (2.42 g, 90%, 11.8 mmol).

^{1}H NMR (500 MHz, $CDCl_3$) δ 7.60 – 7.52 (m, 2H), 7.45 – 7.31 (m, 3H), 4.36 (s, 2H), 3.39 – 3.21 (m, 4H), 2.38 – 2.19 (m, 2H), 1.81 – 1.65 (m, 2H), 1.59 – 1.47 (m, 4H).

^{13}C{H} NMR (126 MHz, $CDCl_3$) δ 132.6 (2C), 130.8, 129.4, 128.5 (2C), 76.6, 69.6, 26.8, 21.4.

HRMS: *m/z* calculated for $C_{13}H_{20}NO$ $[M+H]^+$ 206.1539; found 206.1538.

IR (solid, cm^{-1}): 3168, 2930, 2863, 1496, 1452, 1352, 1215, 1021.

1-benzyl-1,2,3,4-tetrahydroisoquinoline N-oxide (2m):

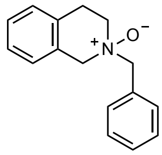

Prepared according to general procedure **7** using 1-benzyl-1,2,3,4-tetrahydroisoquinoline (3.50 g, 15.8 mmol, 1.0 equiv), *m*-CPBA (4.70 g, 18.9 mmol, 1.2 equiv) and anhydrous K_2CO_3 (9.80 g, 70.9 mmol, 4.5 equiv). Purification by automated flash column chromatography (RediSep Rf Silver Si 40 g, 0–20% MeOH in $CHCl_3$) delivered the title compound as an amorphous white solid (2.90 g, 76%, 12.1 mmol).

^{1}H NMR (400 MHz, $CDCl_3$): δ 7.59 (dd, *J* = 7.3, 2.3 Hz, 2H), 7.50 – 7.36 (m, 3H), 7.26 – 7.18 (m, 3H), 6.98 (d, *J* = 7.4 Hz, 1H), 4.53 – 4.39 (m, 3H), 4.22 (d, *J* = 14.9 Hz, 1H), 3.69 – 3.38 (m, 3H), 2.97 (dt, *J* = 16.4, 5.2 Hz, 1H).

^{13}C{H} NMR (101 MHz, $CDCl_3$): δ 132.6 (2C), 131.1, 130.2, 129.8, 129.5, 128.7 (2C), 128.7, 127.8, 127.2, 126.9, 73.6, 66.7, 62.3, 25.9.

HRMS: *m/z* calculated for $C_{16}H_{18}NO$ $[M+H]^+$ 240.1383; found 240.1387.

IR (solid, cm^{-1}): 3032, 2976, 2905, 1495, 1455, 1029, 985, 930, 845.

1-benzyl-1,2,3,4-tetrahydroquinoline N-oxide (2n):

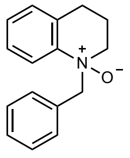

Prepared according to general procedure **7** using 1-benzyl-1,2,3,4-tetrahydroquinoline (3.40 g, 15.2 mmol, 1.0 equiv), *m*-CPBA (4.50 g, 18.3 mmol, 1.2 equiv) and anhydrous K_2CO_3 (9.50 g, 68.5 mmol, 4.5 equiv). Reverse phase automated flash column chromatography (RediSep Rf 130 g C18-column, 0–100% CH_3CN in H_2O) delivered the title compound as a yellow oil (0.98 g, 27%, 4.1 mmol).

^{1}H NMR (500 MHz, $CDCl_3$): δ 7.50 – 7.43 (m, 2H), 7.43 – 7.31 (m, 3H), 7.18 – 7.07 (m, 2H), 7.03 – 6.95 (m, 1H), 6.84 (td, *J* = 7.2, 1.6 Hz, 1H), 4.96 (s, 2H), 3.37 – 3.22 (m, 2H), 2.75 (t, *J* = 6.7 Hz, 2H), 2.10 – 1.95 (m, 2H).

^{13}C{H} NMR (126 MHz, $CDCl_3$): δ 148.2, 137.0, 129.1 (2C), 128.9, 128.4 (2C), 128.1, 126.6, 124.7, 121.3, 115.9, 75.9, 51.3, 26.4, 22.0.

HRMS*:* *m/z* calculated for $C_{16}H_{18}NO$ $[M+H]^+$ 240.1383; found 240.1388.

IR (film, cm^{-1}): 3062, 3030, 2930, 2843, 1602, 1486, 1455, 1364, 1293, 1004, 748.

1-(pyridin-2-ylmethyl)piperidine N-oxide (2o):

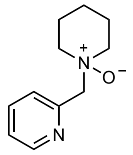

m-CPBA (1.20 g, 5.0 mmol, 1.5 equiv) was added portion wise to a suspension of anhydrous K_2CO_3 (1.40 g, 10.0 mmol, 4.5 equiv) and 1-(pyridine-2-ylmethyl)piperidine (0.58 g, 3.30 mmol, 1.0 equiv) in CH_2Cl_2 (18 mL, 0.18 M) at –78 ˚C. The resulting mixture was warmed slowly to rt over 5 h, after which it was stirred for a further 15 h at rt. The solids were removed by vacuum filtration and washed with CH_2Cl_2 (3 x 20 mL). The combined filtrates were concentrated *in vacuo* to yield the crude product. Purification by automated flash column chromatography (RediSep Rf Silver Si 24 g, 0–20% MeOH in $CHCl_3$) delivered the title compound as an amorphous off-white solid (0.64 g, 79%, 2.6 mmol).

^{1}H NMR (700 MHz, $CDCl_3$) δ 8.55 (ddd, *J* = 4.9, 1.9, 0.9 Hz, 1H), 7.82 (d, *J* = 7.7 Hz, 1H), 7.72 (td, *J* = 7.7, 1.8 Hz, 1H), 7.29 (ddd, *J* = 7.6, 4.9, 1.2 Hz, 1H), 4.43 (s, 2H), 3.20 (td, *J* = 11.5, 3.3 Hz, 2H), 3.15 (dt, *J* = 11.3, 4.3 Hz, 2H), 2.26 (dtt, *J* = 15.1, 11.3, 4.0 Hz, 2H), 1.68 (dp, *J* = 13.2, 4.3 Hz, 1H), 1.61 (dt, *J* = 14.3, 4.1 Hz, 2H), 1.31 (ddt, *J* = 24.6, 11.5, 3.9 Hz, 1H).

^{13}C{H} NMR (176 MHz, $CDCl_3$) δ 150.8, 148.9, 136.7, 128.6, 124.0, 75.5, 65.6, 22.1, 20.9.

HRMS: m/z calculated for $C_{11}H_{16}N_2O$ $[M+H]^+$ 193.1335; found 193.1336.

IR (film, cm^{-1}): 2922, 2846, 1587, 1473, 1431, 1300, 1143, 941, 775, 750, 713, 639.

1-(3-phenylpropyl)pyrrolidine N-oxide (2p):

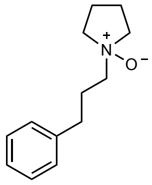

Prepared according to general procedure **7** using crude *1*-(3-phenylpropyl)pyrrolidine (3.30 g, 17.4 mmol, 1.0 equiv), *m*-CPBA (3.90 g, 20.9 mmol, 1.2 equiv) and anhydrous K_2CO_3 (8.90 g, 64.8 mmol, 4.5 equiv). Purification by automated flash column chromatography (RediSep Rf Silver Si 40 g, 0–20% MeOH in $CHCl_3$) delivered the title compound as an amorphous orange solid (2.08 g, 58%, 10.1 mmol).

^{1}H NMR (500 MHz, $CDCl_3$) δ 7.25 – 7.19 (m, 2H), 7.17 – 7.09 (m, 3H), 3.44 – 3.25 (m, 6H), 2.67 (t, *J* = 7.6 Hz, 2H), 2.38 – 2.22 (m, 4H), 1.99 – 1.89 (m, 2H).

^{13}C{H} NMR (126 MHz, $CDCl_3$) δ 140.6, 128.6 (2C), 128.3 (2C), 126.3, 67.8 (2C), 67.0, 32.9, 25.5, 21.6 (2C).

HRMS: *m/z* calculated for $C_{13}H_{20}NO$ $[M+H]^+$ 206.1539; found 206.1539.

IR (solid, cm^{-1}): 3351, 3203, 2958, 2874, 1653, 1604, 1454, 1396.

1-cyclohexylpyrrolidine N-oxide (2q):

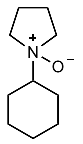

Prepared according to general procedure **7** using crude 1-cyclohexylpyrrolidine (4.74 g, 30.0 mmol, 1.0 equiv), *m*-CPBA (6.21 g, 36.0 mmol, 1.2 equiv) and anhydrous K_2CO_3 (18.65 g, 135 mmol, 4.5 equiv). Purification by automated flash column chromatography (RediSep Rf Silver Si 40 g, 0–20% MeOH in $CHCl_3$) delivered the title compound as an amorphous pink solid (3.15 g, 62%, 18.6 mmol).

^{1}H NMR (500 MHz, $CDCl_3$) δ 3.55 – 3.39 (m, 2H), 3.17 – 3.09 (m, 2H), 2.98 (m, 1H), 2.59 – 2.39 (m, 2H), 2.16 – 2.02 (m, 2H), 2.04 – 1.71 (m, 6H), 1.75 – 1.55 (m, 1H), 1.43 – 1.10 (m, 3H).

^{13}C{H} NMR (126 MHz, $CDCl_3$) δ 75.1, 66.0 (2C), 27.1 (2C), 25.1 (2C), 25.1, 21.4 (2C).

HRMS: *m/z* calculated for $C_{10}H_{20}NO$ $[M+H]^+$ 170.1539; found 170.1545.

IR (solid, cm^{-1}): 2946, 2916, 2851, 1453, 1392, 1369.

1-(3-phenylpropyl)morpholine N-oxide (2r):

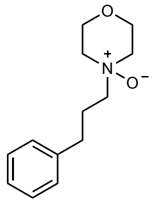

A solution of morpholine (0.7 mL, 8.0 mmol, 1.0 equiv), 1-bromo-3-phenylpropane (1.22 mL, 8.0 mmol, 1.0 equiv) and K_2CO_3 (2.21 g, 16.0 mmol, 2 equiv) in MeCN (12 mL, 0.67 M) was stirred at rt for 16 h. After which, the mixture was filtered through celite®, dried over Na_2SO_4 and concentrated under reduced pressure to yield crude 1-(3-phenylpropyl)morpholine as a pale yellow oil (1.39 g, 84%, 6.7 mmol).

1-(3-phenylpropyl)morpholine *N*-oxide was prepared according to general procedure **7** using crude 1-(3-phenylpropyl)morpholine (1.39 g, 6.7 mmol, 1.0 equiv), *m*-CPBA (2.00 g, 8.1 mmol, 1.2 equiv) and anhydrous K_2CO_3 (4.00 g, 30.4 mmol, 4.5 equiv). Purification by automated flash column chromatography (RediSep Rf Silver 24 g, 0–20% MeOH in CH_2Cl_2) delivered the title compounds as a white solid (1.47 g, 98%, 6.6 mmol).

^{1}H NMR (500 MHz, $CDCl_3$) δ 7.3 – 7.3 (m, 2H), 7.2 – 7.1 (m, 3H), 4.5 – 4.4 (m, 2H), 3.7 (ddt, *J* = 12.6, 3.6, 1.1 Hz, 2H), 3.2 – 3.2 (m, 4H), 3.1 – 3.0 (m, 2H), 2.7 (t, *J* = 7.5 Hz, 2H), 2.4 – 2.3 (m, 2H).

^{13}C{H} NMR (126 MHz, $CDCl_3$) δ 140.5, 128.7, 128.4, 126.5, 71.8, 64.6, 61.8, 32.9, 23.2.

HRMS: *m/z* calculated for $C_{13}H_{19}NO_2$ $[M+H]^+$ 222.1494; found 222.1491.

IR (solid, cm^{-1}): 3083, 3021, 2967, 2854, 1494, 1452, 1265, 1122, 1113, 862, 750, 696.

1-(4-phenyl-benzyl)piperidine N-oxide (7a):

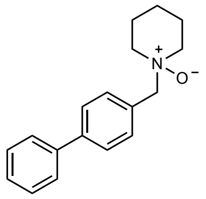

Prepared according to general procedure **7** using 1-(4-phenylbenzyl)piperidine (2.00 g, 8.0 mmol, 1.0 equiv), *m*-CPBA (2.35 g, 9.6 mmol, 1.2 equiv) and anhydrous K_2CO_3 (4.95 g, 35.8 mmol, 4.5 equiv). The title compound was delivered as an amorphous white solid (1.90 g, 89%, 7.1 mmol).

^{1}H NMR (500 MHz, $CDCl_3$) δ 7.60 – 7.54 (m, 6H), 7.42 (dd, *J* = 8.4, 6.9 Hz, 2H), 7.36 – 7.30 (m, 1H), 4.34 (s, 2H), 3.19 – 3.02 (m, 4H), 2.45 – 2.34 (m, 2H), 1.77 – 1.67 (m, 1H), 1.62 – 1.52(m, 2H), 1.22 (qt, *J* = 12.4, 3.6 Hz, 1H).

^{13}C{H} NMR (126 MHz, $CDCl_3$) δ 142.6, 140.2, 133.3 (2C), 129.0 (2C), 128.2 (2C), 127.8, 127.3 (2C), 127.2 (2C), 74.3, 63.9 (2C), 21.8, 20.7 (2C).

HRMS: *m/z* calculated for $C_{18}H_{21}NO$ $[M+H]^+$ 268.1696; found 268.1696.

IR (solid, cm^{-1}): 2921, 1793, 1739, 1378, 1361, 1299, 1199, 1142, 1034, 807, 735.

1-(4-phenyl-benzyl)-2-methyl-piperidine N-oxide (7b):

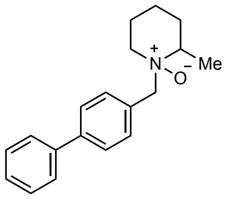

Prepared according to general procedure **7** using 1-(4-phenylbenzyl)-2-methyl-piperidine (0.47 g, 1.78 mmol, 1.0 equiv), *m*-CPBA (0.53 g, 2.14 mmol, 1.2 equiv) and anhydrous K_2CO_3 (1.10 g, 8.0 mmol, 4.5 equiv). Purification by automated flash column chromatography (RediSep Rf Silver Si 12 g, 0–20% MeOH in $CHCl_3$) delivered the title compound as an amorphous off-white solid (0.50 g, 100%, 1.8 mmol).

^{1}H NMR (700 MHz, $CDCl_3$) δ 7.61 – 7.56 (m, 4H), 7.52 (d, *J* = 8.3 Hz, 2H), 7.43 (t, *J* = 7.7 Hz, 2H), 7.37 – 7.33 (m, 1H), 4.46 (d, *J* = 13.1 Hz, 1H), 4.31 (d, *J* = 13.1 Hz, 1H), 3.20 – 3.09 (m, 2H), 2.97 (ddd, *J* = 12.7, 11.6, 3.0 Hz, 1H), 2.35 (qt, *J* = 12.9, 3.9 Hz, 1H), 2.15 (dddd, *J* = 14.3, 13.2, 11.4, 4.1 Hz, 1H), 1.71 – 1.62 (m, 1H), 1.53 – 1.40 (m, 5H), 1.19 (qt, *J* = 13.2, 4.0 Hz, 1H).

^{13}C{H} NMR (176 MHz, $CDCl_3$) δ 142.3, 140.2, 132.5 (2C), 129.6, 129.0 (2C), 127.9, 127.5 (2C), 127.2 (2C), 72.5, 66.3, 64.4, 29.0, 22.9, 20.5, 15.8.

HRMS: *m/z* calculated for $C_{19}H_{23}NO$ $[M+H]^+$ 282.1852; found 282.1855.

IR (solid, cm^{-1}): 2936, 2855, 1488, 1442, 1410, 1367, 1322, 961,934, 835, 765, 733, 692.

Dextromethorphan N-oxide (33):

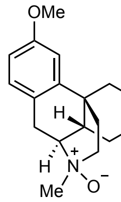

Prepared according to general procedure **7** using dextromethorphan (2.30 g, 8.5 mmol, 1.0 equiv), *m*-CPBA (2.52 g, 10.2 mmol, 1.2 equiv) and anhydrous K_2CO_3 (5.29 g, 38.3 mmol, 4.5 equiv). Purification by automated flash column chromatography (RediSep Rf Silver Si 24 g, 0–20% MeOH in $CHCl_3$) delivered the title compound as a pale yellow oil (2.08 g, 85%, 7.2 mmol).

^{1}H NMR (500 MHz, MeOD) δ 7.10 (d, *J* = 8.5 Hz, 1H), 6.85 (d, *J* = 2.6 Hz, 1H), 6.80 (dd, *J* = 8.4, 2.6 Hz, 1H), 3.76 (s, 4H), 3.50 (s, 3H), 3.44 – 3.36 (m, 1H), 3.20 – 3.13 (m, 2H), 3.05 – 2.97 (m, 1H), 2.97 – 2.84 (m, 1H), 2.42 (dq, *J* = 14.0, 2.6 Hz, 1H), 2.32 (td, *J* = 13.3, 4.5 Hz, 1H), 1.70 – 1.62 (m, 1H), 1.55 (dt, *J* = 16.1, 3.2 Hz, 1H), 1.48 – 1.35 (m, 4H), 1.33 – 1.21 (m, 1H), 1.02 (qd, *J* = 13.0, 4.1 Hz, 1H).

^{13}**C{H} NMR** (126 MHz, MeOD) δ 160.6, 140.5, 130.4, 126.2, 113.4, 111.8, 75.0, 61.0, 58.1, 55.7, 38.6, 38.0, 37.1, 36.3, 28.5, 27.3, 27.3, 23.1.

HRMS: *m/z* calculated for $C_{18}H_{25}NO_2$ $[M+H]^+$ 288.1958; found 288.1958.

IR (film, cm^{-1}): 2930, 2855, 2495, 1608, 1495, 1436, 1238, 1163, 1040, 938, 921, 874, 849, 806, 739, 653

Reserpine N-oxide (34):

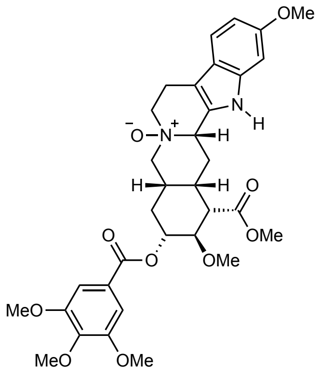

Prepared according to general procedure **7** using reserpine (900 mg, 1.5 mmol, 1.0 equiv), *m*-CPBA (401 mg, 1.6 mmol, 1.2 equiv) and anhydrous K_2CO_3 (920 mg, 6.7 mmol, 4.5 equiv). Purification by automated flash column chromatography (RediSep Rf Gold Si 12 g, 0–20% MeOH in $CHCl_3$) delivered the title compound as an amorphous brown solid (385 mg, 41%, 0.6 mmol).

^{1}**H NMR** (700 MHz, $CDCl_3$) δ 7.36 (s, 2H), 7.19 (d, *J* = 8.6 Hz, 1H), 6.93 (d, *J* = 2.3 Hz, 1H), 6.74 (dd, *J* = 8.6, 2.2 Hz, 1H), 4.92 (ddd, *J* = 12.2, 9.4, 4.6 Hz, 1H), 4.53 (brs, 1H), 4.14 (t, *J* = 10.3 Hz, 1H), 3.90 (s, 3H), 3.88 (s, 9H), 3.82 (s, 3H), 3.69 – 3.62 (m, 3H), 3.55 (s, 3H), 3.36 (br s, 2H), 3.27 – 3.21 (m, 1H), 3.02 (d, *J* = 12.3 Hz, 1H), 2.94 – 2.79 (m, 1H), 2.68 (dd, *J* = 11.3, 5.0 Hz, 1H), 2.65 – 2.56 (m, 1H), 2.26 (d, *J* = 14.3 Hz, 1H), 2.15 (dq, *J* = 8.2, 4.3 Hz, 2H), 2.03 – 1.96 (m, 1H).

^{13}**C{H} NMR** (176 MHz, $CDCl_3$) δ 172.1, 165.7, 156.9, 153.0 (2C), 142.3, 138.5, 127.7, 125.3, 120.7, 118.7, 109.6, 107.0 (2C), 106.1, 95.7, 77.7, 77.6, 71.4, 68.4, 60.9, 60.8, 60.4, 56.4 (2C), 55.8, 52.4, 51.5, 33.4, 31.5, 31.3, 20.9, 20.1.

HRMS: *m/z* calculated for $C_{33}H_{40}N_2O_{10}$ $[M+H]^+$ 625.2756; found 625.2752.

IR (solid, cm^{-1}): 2940, 2837, 2252, 1737, 1706, 1629, 1588, 1503, 1462, 1415, 1331, 1221, 1125, 981, 905, 722, 646.

Cloperastine N-oxide (35):

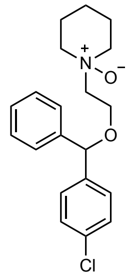

Prepared according to general procedure **7** using cloperastine (1.45 g, 4.4 mmol, 1.0 equiv), *m*-CPBA (1.30 g, 5.3 mmol, 1.2 equiv) and anhydrous K_2CO_3 (2.74 g, 19.8 mmol, 4.5 equiv). The title compound was delivered as an amorphous white solid (1.47 g, 97%, 4.3 mmol).

^{1}H NMR (500 MHz, $CDCl_3$) δ 7.31 – 7.19 (m, 9H), 5.36 (s, 1H), 4.08 – 3.97 (m, 2H), 3.45 – 3.35 (m, 2H), 3.28 – 3.15 (m, 4H), 2.31 – 2.19 (m, 2H), 1.72 – 1.57 (m, 3H), 1.48 – 1.28 (m, 1H).

^{13}C{H} NMR (126 MHz, $CDCl_3$) δ 141.2, 140.4, 133.4, 128.7 (2C), 128.7 (2C), 128.2 (2C), 127.9, 126.9 (2C), 83.5, 69.6, 66.9, 66.8, 62.9, 22.3, 21.4 (2C).

HRMS: *m/z* calculated for $C_{20}H_{24}NO_2Cl$ $[M+H]^+$ 346.1568; found 346.1574.

IR (solid, cm^{-1}): 2939, 2866, 1651, 1488, 1450, 1328, 1185, 1088, 1013, 860, 797, 756, 718, 700.

Ticlopidine N-oxide (36):

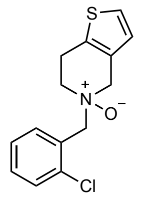

Prepared according to general procedure **7** using ticlopidine (878 mg, 3.3 mmol, 1.0 equiv), *m*-CPBA (986 mg, 4.0 mmol, 1.2 equiv) and anhydrous K_2CO_3 (2.07 g, 15.0 mmol, 4.5 equiv). Purification by automated flash column chromatography (RediSep Rf Silver Si 12 g, 0–20% MeOH in $CHCl_3$) delivered the title compound as an amorphous brown solid (541 mg, 58%, 1.9 mmol).

^{1}H NMR (400 MHz, MeOD) δ 7.92 – 7.89 (m, 1H), 7.53 (dd, *J* = 7.9, 1.5 Hz, 1H), 7.47 (td, *J* = 7.6, 1.8 Hz, 1H), 7.41 (td, *J* = 7.5, 1.6 Hz, 1H), 7.29 (d, *J* = 5.2 Hz, 1H), 6.74 (d, *J* = 5.2 Hz, 1H), 4.85 (d, *J* = 12.8 Hz, 1H), 4.75 – 4.61 (m, 2H), 4.16 (d, *J* = 14.7 Hz, 1H), 3.77 (ddd, *J* = 11.8, 10.1, 4.9 Hz, 1H), 3.70 – 3.59 (m, 1H), 3.39 (ddd, *J* = 15.9, 9.8, 5.7 Hz, 1H), 3.05 (dt, *J* = 16.8, 4.1 Hz, 1H).

^{13}C{H} NMR (101 MHz, MeOD) δ 137.3, 137.2, 132.7, 132.2, 130.9, 129.3, 128.6, 128.2, 126.4, 125.7, 70.2, 65.3, 64.4, 22.6.

HRMS: *m/z* calculated for $C_{14}H_{14}NOSCl$ $[M+H]^+$ 280.0557; found 280.0561.

IR (solid, cm^{-1}): 3023, 2853, 1570, 1478, 1444, 1355, 1215, 1161, 1055, 1010, 959, 910, 830, 747, 699, 683, 655.

Matrine N-oxide (37):

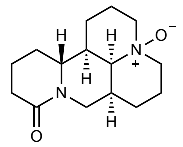

Prepared according to general procedure **7** using Matrine (0.20 g, 0.8 mmol, 1.0 equiv), *m*-CPBA (0.27 g, 1.0 mmol, 1.2 equiv) and anhydrous K_2CO_3 (0.50 g, 3.6 mmol, 4.5 equiv). The title product was delivered as a pale yellow oil (0.35 g, 100%, 0.8 mmol).

Spectra are in accordance with literature data.[64]

Flavoxate N-oxide (38):

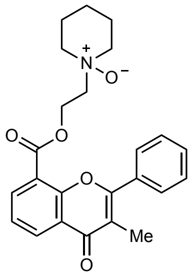

Prepared according to general procedure **7** using flavoxate (1.40 g, 3.6 mmol, 1.0 equiv), *m*-CPBA (1.06 g, 4.3 mmol, 1.2 equiv) and anhydrous K_2CO_3 (2.23 g, 16.1 mmol, 4.5 equiv). The title compound was delivered as an amorphous off-white solid (0.95 g, 65%, 2.3 mmol).

^{1}H NMR (400 MHz, $CDCl_3$) δ 8.49 (dd, *J* = 8.0, 1.8 Hz, 1H), 8.25 (dd, *J* = 7.6, 1.8 Hz, 1H), 7.81 – 7.73 (m, 2H), 7.66 – 7.51 (m, 3H), 7.46 (t, *J* = 7.7 Hz, 1H), 5.10 – 4.94 (m, 2H), 3.56 – 3.40 (m, 2H), 3.23 – 3.11 (m, 2H), 2.87 (td, *J* = 11.3, 3.2 Hz, 2H), 2.33 – 2.15 (m, 5H), 1.64 (dt, *J* = 13.6, 4.6 Hz, 1H), 1.52 – 1.39 (m, 2H), 1.24 – 1.10 (m, 1H).

^{13}C{H} NMR (101 MHz, $CDCl_3$) δ 178.2, 164.1, 161.0, 154.7, 136.4, 133.3, 131.5, 130.8, 129.4 (2C), 128.8 (2C), 124.3, 123.5, 120.0, 118.1, 68.6, 66.7 (2C), 59.4, 22.1, 21.1 (2C), 11.8.

HRMS: *m/z* calculated for $C_{24}H_{26}NO_5$ $[M+H]^+$ 408.1806; found 408.1798.

IR (solid, cm^{-1}): 3062, 2931, 2854, 2800, 1727, 1639, 1602, 1479, 1440, 1391, 1376, 1263, 1178, 1127, 761, 699.

Risperidone N-oxide (39):

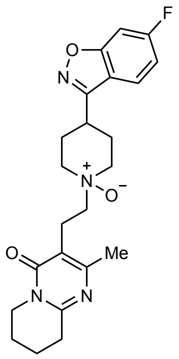

Prepared according to general procedure **7** using risperidone (400 mg, 1.0 mmol, 1.0 equiv), *m*-CPBA (264 mg, 1.2 mmol, 1.2 equiv) and anhydrous K_2CO_3 (604 mg, 4.5 mmol, 4.5 equiv). The title compound was delivered as an amorphous white solid (220 mg, 53%, 0.5 mmol).

^{1}H NMR (500 MHz, $CDCl_3$) δ 7.97 (dd, *J* = 8.8, 5.1 Hz, 1H), 7.22 (dd, *J* = 8.4, 2.1 Hz, 1H), 7.04 (td, *J* = 8.9, 2.2 Hz, 1H), 3.90 (t, *J* = 6.2 Hz, 2H), 3.59 – 3.44 (m, 2H), 3.41 – 3.34 (m, 2H), 3.34 – 3.19 (m, 5H), 3.11 (qd, *J* = 13.0, 3.8 Hz, 2H), 2.86 (t, *J* = 6.6 Hz, 2H), 2.38 (s, 3H), 2.01 – 1.90 (m, 4H), 1.90 – 1.79 (m, 2H).

^{13}C{H} NMR (126 MHz, $CDCl_3$) δ 164.3 (d, *J* = 251.3 Hz), 164.3 (d, *J* = 13.6 Hz), 162.9, 160.2, 160.1, 156.8, 123.5 (d, *J* = 11.1 Hz), 116.8, 116.6 (d, *J* = 1.3 Hz), 112.85 (d, *J* = 25.2 Hz), 97.58 (d, *J* = 26.8 Hz), 69.2, 64.1 (2C), 42.9, 32.9, 31.6, 25.1 (2C), 22.0, 21.5, 20.2, 19.3.

^{19}F{H} NMR (471 MHz, $CDCl_3$) δ -108.96.

HRMS: *m/z* calculated for $C_{23}H_{27}N_4O_3F$ $[M+H]^+$ 427.2140; found 427.2141.

IR (solid, cm^{-1}): 2951, 1643, 1531, 1414, 1347, 1268, 1191, 1113, 957, 826, 739, 620.

Cisapride N-oxide (40):

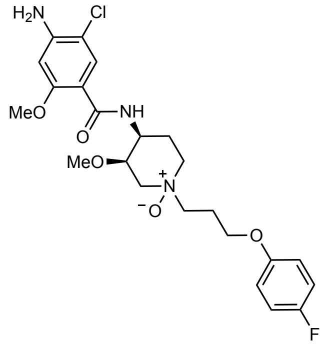

Prepared according to general procedure **7** using cisapride (967 mg, 2.1 mmol, 1.0 equiv), *m*-CPBA (616 mg, 2.3 mmol, 1.2 equiv) and anhydrous K_2CO_3 (1.29 g, 9.4 mmol, 4.5 equiv). Purification by automated flash column chromatography (RediSep Rf Gold Si 12 g, 0–20% MeOH in $CHCl_3$) delivered the title compound as an amorphous red solid (944 mg, 94%, 2.0 mmol).

^{1}H NMR (700 MHz, MeOD) δ 7.85 (s, 1H), 7.02 – 6.98 (m, 2H), 6.93 – 6.89 (m, 2H), 6.52 (s, 1H), 4.38 – 4.21 (m, 1H), 4.09 (t, *J* = 5.9 Hz, 2H), 3.94 (s, 3H), 3.72 (q, *J* = 3.8 Hz, 1H), 3.64 (s, 1H), 3.55 – 3.48 (m, 9H), 3.42 – 3.36 (m, 1H), 2.56 (dtd, *J* = 13.7, 10.0, 3.5 Hz, 1H), 2.43 – 2.32 (m, 2H), 1.91 (dq, *J* = 10.3, 3.2 Hz, 1H).

^{13}C{H} NMR (176 MHz, MeOD) δ 166.7, 159.7, 158.8 (d, *J* = 237.1 Hz), 156.3 (d, *J* = 2.0 Hz), 150.6, 133.2, 115.3 (d, *J* = 8.0 Hz), 115.3 (d, *J* = 23.4 Hz), 111.7, 111.0, 98.6, 75.7, 67.1, 64.0, 63.8, 57.8, 56.4, 47.8, 46.4, 24.1, 23.7.

^{19}F{H} NMR (471 MHz, MeOD) δ -125.95.

HRMS: *m/z* calculated for $C_{23}H_{29}N_3O_5FCl$ $[M+H]^+$ 482.1853; found 482.1857.

IR (solid, cm^{-1}): 3345, 3208, 2943, 2512, 1626, 1595, 1506, 1441, 1400, 1249, 1209, 1082, 829

6.3 Other

*Anhydrous copper perchlorate (**41**):*

Adapted from the literature procedure by Tanaka and co-workers. [65]

Crystals of hydrated copper perchlorate (1.50 g, 4.1 mmol, 1.0 equiv) were dissolved in DMF (3 mL, 1.4 M) in the presence of 4 Å MS and allowed to stand for 1 day under reduced pressure. The 4 Å MS were removed and the resulting mixture was concentrated *in vacuo* at 40 ˚C to deliver the title compound as an amorphous blue solid (1.05 g, 100%, 4.1 mmol).

2-acetoxy-4,5,6,7-tetrachloro-isoindoline-1,3-dione (**42**) and zinc methyl iodide (**43**) were prepared according to the literature procedures by Gaunt and co-workers.[9e]

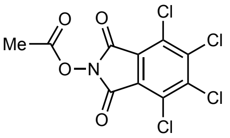

42
ref. 62

43
ref. 62

Diethyl magnesium (**44**):

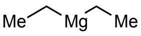

Adapted from the literature procedure by Brückner and co-workers.[67]

An oven-dried 2-neck round bottom flask was charged with a magnetic stirrer bar and magnesium (1. 65 g, 68.0 mmol, 1.0 equiv). The flask was sealed and evacuate-refilled with N_2 (3 cycles). Et_2O was added (30 mL) and then ethyl bromide (5.0 mL, 68.0 mmol, 1.0 equiv) was added dropwise over 90 mins, after which, the dark grey suspension was heated under reflux for 4 h. After cooling to 0 ˚C, a solution of 1-methoxy-2-(2-methoxy)ethane (3.60 mL, 25.2 mmol, 0.4 equiv) in Et_2O (4.5 mL) followed by 1,4-dioxane (3.3 mL, 38.6 mmol, 0.6 equiv) in Et_2O (3 mL) were added dropwise over 75 and 90 mins respectively. The suspension was stirred at –10 ˚C for 16 h and then filtered with suction under an N_2 atmosphere. The clear and colourless filtrate was concentrated to approximately half its original volume by a stream of nitrogen. Its concentration was determined by titration against iodine in THF (see ref 157).

7. Computational calculations

7.1 General information

All calculations were performed using Gaussian 16 A.03.[16a] Geometry optimisation and frequency calculations were run at the wB97XD/6-31'+g(d,p) level of theory in CH_2Cl_2 (IEPCM).[16b-c] Single point energies of the optimised structures were calculated at the wB97XD/6-311++g(d,p) level of theory in CH_2Cl_2 (IEPCM).[16d] wB97XD is a long-range corrected (LC) hybrid functional incorporating additional empirical dispersion corrections which have been shown to be effective in predicting noncovalent interactions.[16b] Gibbs free energies were obtained by adding the thermal correction to Gibbs Free Energy term from geometry optimisation to the single point energy. To account for the change in the standard state (1 atm → 1 M) an entropic correction term of 1.89 kcal/mol (–RTlnQ) was added to all considered species.[16e]

Gibbs Free Energy (6-311++g(d,p)) = Single Point Energy (6-311++g(d,p)) + thermal correction to Gibbs Free Energy (6-31'+g(d,p)) + x(–RTlnQ)

T = 298K, x = number of species

All reported structures were confirmed as either true minima (no imaginary frequencies), or true first-order saddle points (one imaginary frequency for transition states) on the potential energy surface *via* vibrational analysis. Transition states were confirmed by relaxation towards both the reactant and the products.

7.2 Predicting regioselectivity using DFT

The low temperatures associated with iminium ion formation suggest that this reaction is under kinetic control. As a result, regioselectivity can be directly linked to the difference in the relative Gibbs Free Energies of activation of the two transitions states for the competing reaction paths (i.e *endo* vs *exo* α-C–H elimination).[68]

$$\frac{exo}{endo} = e^{-\frac{\Delta\Delta G^{\neq}}{RT}} \quad (\textbf{Equation 1})$$

The temperature at which iminium ion formation occurs was determined *via* low temperature ^1H NMR studies (Figure **S3**). A solution of pivaloyl chloride (163 μL, 1.32 mmol, 6.6 equiv) in anhydrous CD_2Cl_2 (0.25 mL, 0.8 M) was cooled to –78 °C. To this was added dropwise a solution of 1-benzylpiperidine *N*-oxide **2a** (38.2 mg, 0.20 mmol, 1.0 equiv) in anhydrous CD_2Cl_2 (0.25 mL, 0.8 M), which was also cooled to –78 °C. The vial containing the *N*-oxide was washed with CD_2Cl_2 (0.2 mL), cooled to –78 °C and added dropwise to the pivaloyl chloride solution. After complete addition, the mixture was stirred for a further 15 mins at –78 °C. Next, 1,1,2,2-tetrachloroethane was added as an internal standard and the reaction mixture was transferred to an NMR spectrometer equipped with a cryoprobe set to an internal temperature of –50 °C. Formation of iminium ion **3a** was monitored by ^1H NMR spectroscopic experiments

recorded at 5 ˚C intervals between –50 ˚C and 25 ˚C. This investigation revealed that formation of iminium ion **3a** starts at approximately –20 ˚C and this value was used as the temperature parameter for subsequent calculations.

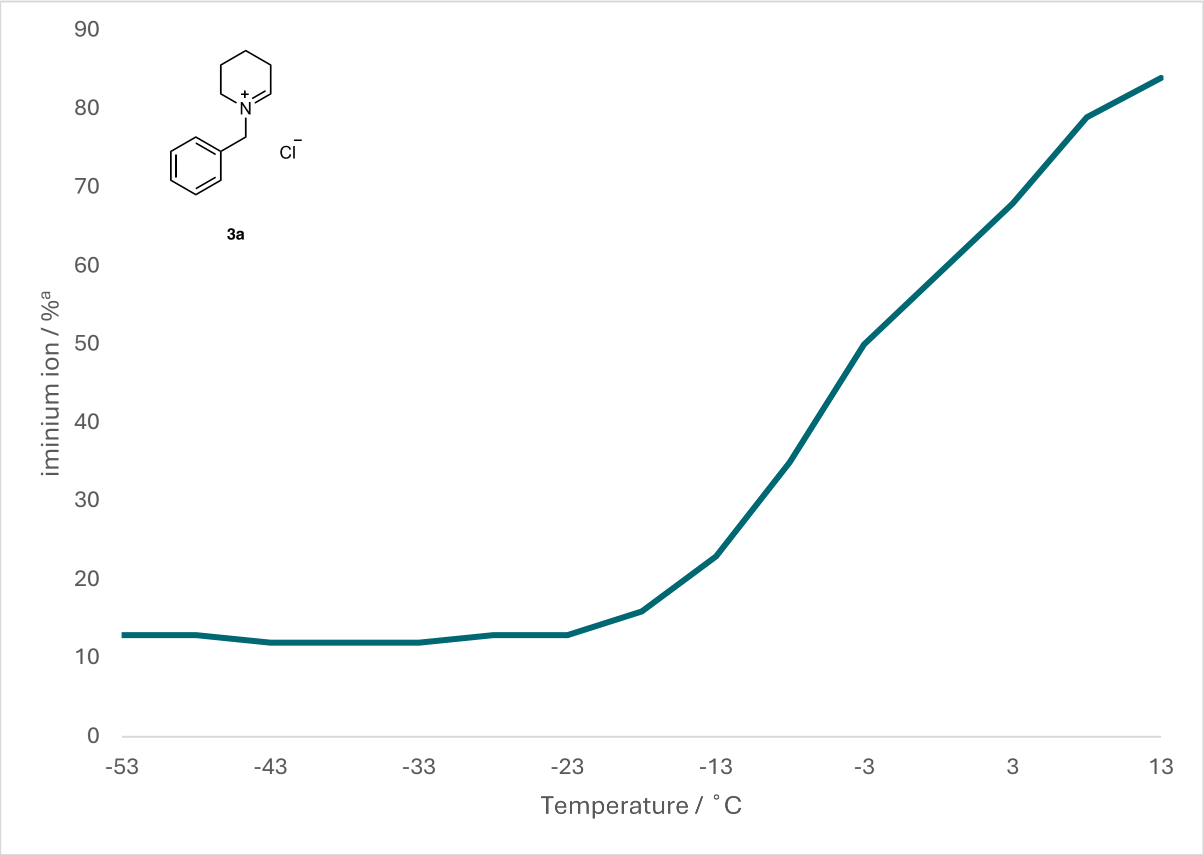

Figure S3: Formation of iminium ion **3a** from 1-benzylpiperidine *N*-oxide **2a** and PivCl was monitored by ^{1}H NMR at 5 ˚C intervals between –50 ˚C and 25 ˚C. [a]Assay yields were determined by ^{1}H NMR using 1,1,2,2-tetrachloroethane as an internal standard

For 1-benzylpiperidine, DFT calculations revealed a difference in the relative Gibbs Free Energies between the ***endo*-TS1** and ***exo*-TS1** transition states of 1.6 kcal/mol (Figure **S4**). This difference translates with equation 1 to a predicted regioisomeric ratio (r.r.) of >20 : 1 for *endo*:*exo* iminium ion formation at 253 K, which is in perfect agreement with our experimental values for **3a**.

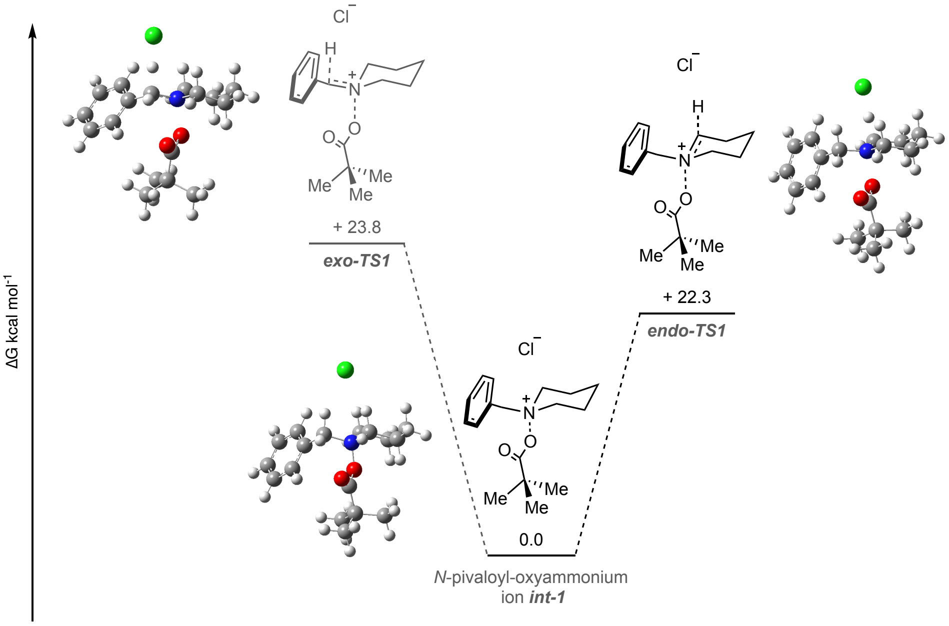

Figure S4: Single point energies of the *exo*- and *endo*-cyclic α-C–H elimination transition states in 1-benzylpiperidine. Calculations were performed at the wB97XD/6-311++g(d,p) level of theory.

For 1-benzylpyrroldine, ^{1}H NMR spectroscopic experiments revealed that formation of iminium ions **3k/4k** also starts at approximately –20 ˚C (Figure **S5**). DFT calculations revealed a difference in the relative Gibbs Free Energies between the ***endo*-TS2** and ***exo*-TS2** transition states of 0.2 kcal/mol (Figure **S6**). This difference translates with equation 1 to a predicted regioisomeric ratio (r.r.) of approximately 1 : 1 for *endo*:*exo* iminium ion formation at 253 K, which is in agreement with our experimental values for **3k/4k**.

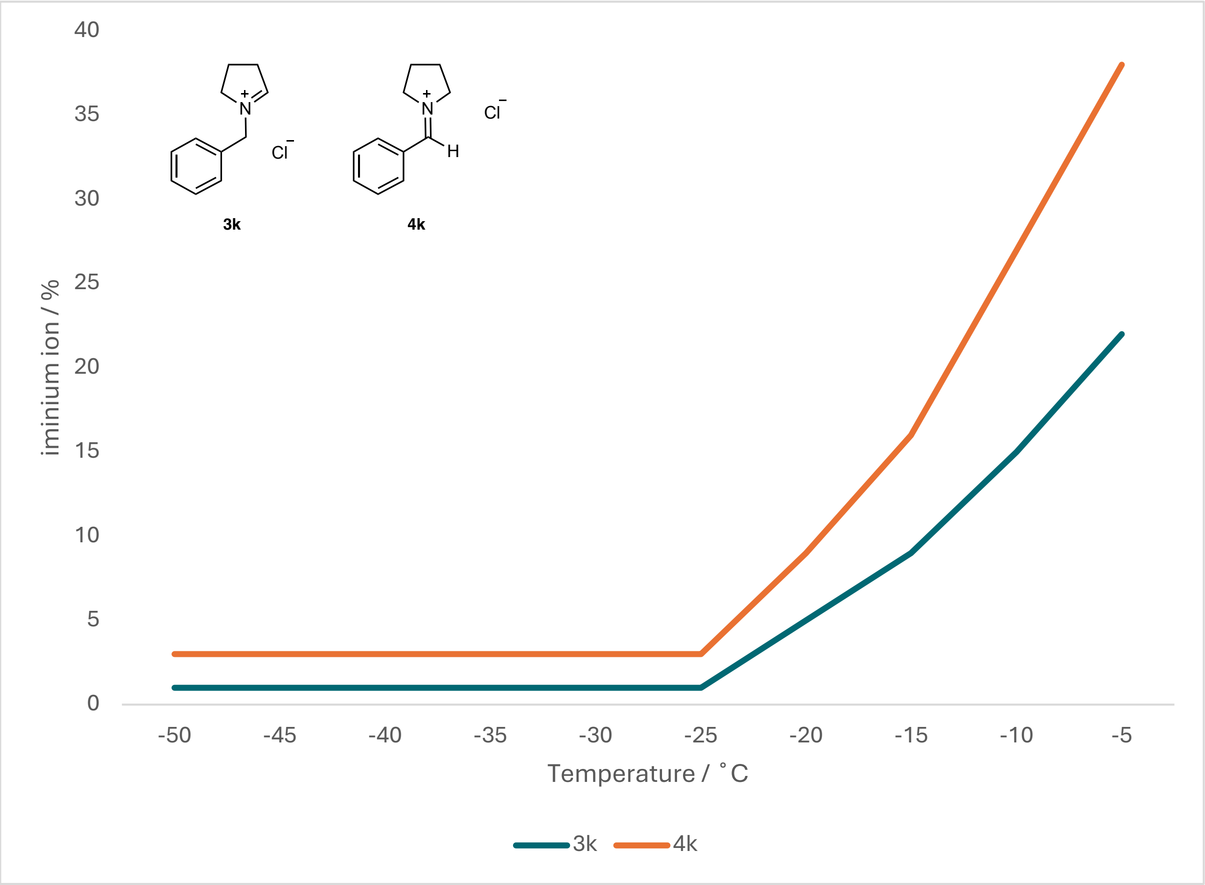

Figure S5: Formation of iminium ions **3k/4k** from 1-benzylpyrrolidine *N*-oxide **2k** and PivCl was monitored by ^{1}H NMR at 5 ˚C intervals between –50 ˚C and 5 ˚C. [a]Assay yields determined by ^{1}H NMR using 1,1,2,2-tetrachloroethane as an internal standard

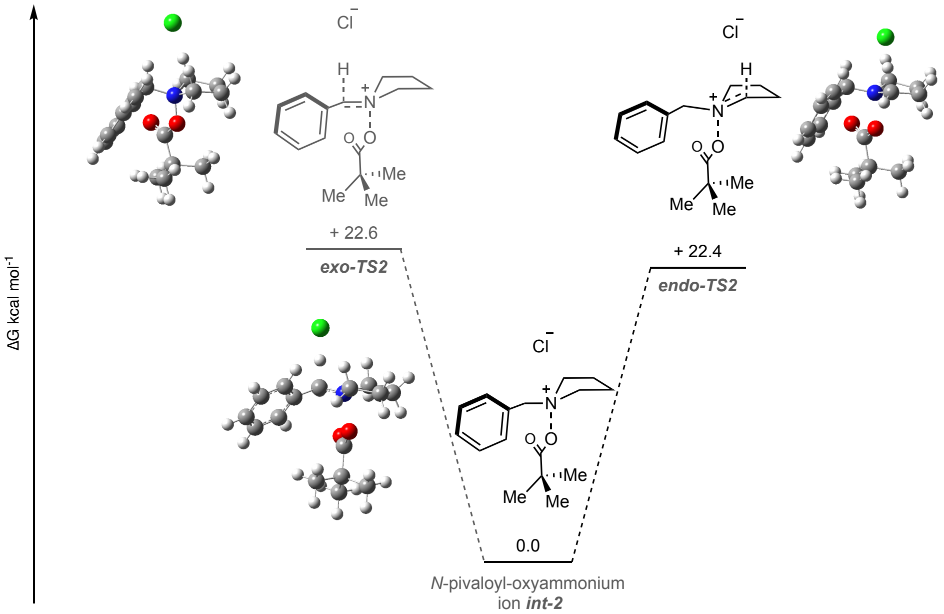

Figure S6: Single point energies of the *exo*- and *endo*-cyclic α-C–H elimination transition states in 1-benzylpyrrolidine. Calculations were performed at the wB97XD/6-311++g(d,p) level of theory.

7.3 DFT computed transition states for the regiodetermining step

1-benzyl-1-(pivaloyloxy)piperidin-1-ium chloride int-1

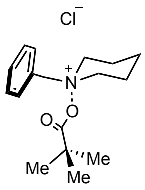

Geometry optimisation and frequency:

Sum of electronic and thermal Free Energies = -1328.645387 Hatrees
Thermal correction to Gibbs Free Energy = 0.360966 Hatrees

Single Point Energy:

Energy = -1328.846475 Hatrees

C	-0.72916670	-0.17361111	0.00000000
C	-1.03321370	1.05974089	-0.83685300
C	-0.62544770	-0.23179711	-2.91265400
C	-0.29154470	-1.47017311	-2.09913700
C	-1.02916970	-1.46462811	-0.76052200
H	-2.09175670	1.11594789	-1.11925900
H	-0.72998970	1.97905189	-0.33448300
H	0.31624130	-0.15326311	0.32824800
H	-1.34854770	-0.09790311	0.89956400
H	-1.70487170	-0.14566511	-3.08975400
H	-0.09473970	-0.18884311	-3.86250000
H	-0.58404470	-2.33261111	-2.70639100
H	0.79202530	-1.54568511	-1.94465800
H	-2.10896570	-1.54055711	-0.94289100
H	-0.73568170	-2.33213611	-0.16161400
C	-0.67889770	2.25153789	-3.01577400
H	-1.76333970	2.13497289	-3.13411900
C	2.08540730	1.03493789	-2.67474400
C	3.41837030	1.00620689	-1.93835500
N	-0.29790370	1.04385889	-2.16015100
O	1.06676030	1.08839789	-1.74763000
O	1.89751430	0.99723289	-3.85805600
C	4.53715930	1.17871689	-2.97111500
H	4.45769130	2.14238389	-3.48516600
H	5.50355130	1.14161589	-2.45781900
H	4.51477630	0.38459889	-3.72336600
C	3.53426430	-0.36833211	-1.24681200
H	4.50566830	-0.43054311	-0.74516300
H	2.75175630	-0.50514511	-0.49348100
H	3.47469930	-1.18719611	-1.97254100
C	3.47881530	2.13133989	-0.88959900
H	4.45945730	2.10397289	-0.40302400
H	3.35667930	3.11620789	-1.35295100
H	2.70978830	2.01203389	-0.12138400
C	-0.31771670	3.57415089	-2.40326200
C	-1.23501970	4.24695189	-1.58845400
C	0.91832530	4.16877089	-2.67565000
C	-0.90739570	5.47915989	-1.02739400

H	-2.21238070	3.80668689	-1.40283600
C	1.24849830	5.39998589	-2.11363700
H	1.62045730	3.67813889	-3.34598500
C	0.33820730	6.05313889	-1.28307800
H	-1.62720970	5.99379189	-0.39728900
H	2.21210930	5.85226089	-2.33120000
H	0.59358330	7.01499189	-0.84690600
H	-0.18779470	2.09928089	-3.97647200
Cl	-4.05778670	1.09939989	-2.74907300

1-benzylpiperidine *exo*-TS1

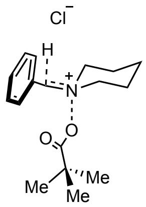

Geometry optimisation and frequency:

Sum of electronic and thermal Free Energies = -1328.599181 Hatrees
Thermal correction to Gibbs Free Energy = 0.352199 Hatrees

Single Point Energy:

Energy = -1328.799744 Hatrees

C	-1.38888896	-0.24305555	0.00000000
C	-1.81802096	1.02165245	-0.73080600
C	-1.48818696	-0.14639155	-2.92105500
C	-1.01463896	-1.38858855	-2.18752000
C	-1.67223696	-1.50290755	-0.81422200
H	-2.91279296	1.06280945	-0.82646400
H	-1.47664296	1.92094345	-0.21774800
H	-0.32360096	-0.17838255	0.23765700
H	-1.93954996	-0.25705555	0.94650800
H	-2.56967096	-0.20395355	-3.11579100
H	-0.95835396	0.01207745	-3.85983700
H	-1.26992496	-2.24574755	-2.81915800
H	0.07580804	-1.36160155	-2.09075700
H	-2.75638096	-1.63511855	-0.93467300
H	-1.29575496	-2.38374855	-0.28482300
C	-1.61830996	2.25289045	-2.83817600
H	-2.94367096	2.09895145	-2.97177200
C	1.33307404	1.40001245	-2.59251000
C	2.72668104	1.82116945	-2.07558100
N	-1.33379296	1.10871145	-2.13231900
O	0.50111104	1.10976145	-1.63650000
O	1.06947004	1.32286445	-3.78977700
C	3.45101304	2.61919845	-3.16526800
H	2.91641804	3.54765745	-3.39775700
H	4.45617104	2.88368345	-2.81578600
H	3.54467204	2.04039245	-4.08852400
C	3.49914904	0.52210445	-1.77546900
H	4.50846004	0.76613545	-1.42298400

H	2.99758004	-0.06485855	-0.99790300
H	3.59347504	-0.09825255	-2.67437100
C	2.60565104	2.66512145	-0.79859000
H	3.60481904	2.97537045	-0.47001500
H	2.00918804	3.56799745	-0.97571000
H	2.13547904	2.09997245	0.01136200
C	-1.38333496	3.59828445	-2.23178800
C	-2.21348896	4.15231045	-1.25088000
C	-0.29691396	4.34324745	-2.70801900
C	-1.93493796	5.41206145	-0.72466100
H	-3.09083796	3.60974645	-0.90926300
C	-0.01773096	5.60083845	-2.17906900
H	0.33594004	3.92770345	-3.48875800
C	-0.83310596	6.13490945	-1.18130200
H	-2.58582496	5.83194545	0.03706200
H	0.83543104	6.16329345	-2.54839500
H	-0.61790396	7.11706945	-0.76940900
H	-1.27080796	2.16356945	-3.86711100
Cl	-4.52194296	1.93877345	-3.13729400

1-benzylpiperidine *endo*-TS1

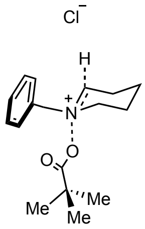

Geometry optimisation and frequency:

Sum of electronic and thermal Free Energies = -1328.601363 Hatrees
Thermal correction to Gibbs Free Energy = 0.351872 Hatrees

Single Point Energy:

Energy = -1328.801991 Hatrees

C	-2.11805566	0.59027777	0.00000000
C	-2.39010666	1.76911977	-0.91260100
C	-1.48739966	0.59255077	-2.83731800
C	-1.05783266	-0.56223423	-1.94901800
C	-2.01154766	-0.72545323	-0.76773500
H	-3.57709966	1.58551777	-1.41258200
H	-2.49992666	2.73784477	-0.42389200
H	-1.18520666	0.79590077	0.54080900
H	-2.92341166	0.55284477	0.73990900
H	-2.47271366	0.39842377	-3.28609000
H	-0.76958866	0.80836177	-3.62819500
H	-1.04096466	-1.46262823	-2.57113400
H	-0.03523166	-0.38558023	-1.59723300
H	-3.00395566	-1.01818823	-1.13361000
H	-1.66221566	-1.51987723	-0.10126900
C	-1.96453366	3.02045577	-2.97531500
H	-2.92063966	2.75675577	-3.44825300

C	1.09677834	2.31888877	-2.25049800
C	2.41934534	2.53466277	-1.48070800
N	-1.68185166	1.85687977	-2.07817100
O	0.07188234	2.22326177	-1.45305800
O	1.03792034	2.25885777	-3.47531300
C	3.59346834	2.52557277	-2.46336500
H	3.49514134	3.31949577	-3.21036100
H	4.52821734	2.68376277	-1.91218200
H	3.66346534	1.56937977	-2.99232700
C	2.58329734	1.40397677	-0.45055200
H	3.53091134	1.52997177	0.08636800
H	1.76764734	1.41101777	0.27904800
H	2.60121234	0.42269377	-0.94032000
C	2.34761634	3.89317677	-0.76096500
H	3.27114234	4.05554777	-0.19270300
H	2.24673434	4.71722077	-1.47773800
H	1.50175434	3.93225877	-0.06734200
C	-2.05713566	4.35237577	-2.28573900
C	-3.28060966	4.81347377	-1.78903700
C	-0.92172666	5.15905977	-2.16887100
C	-3.36091866	6.05363677	-1.15777100
H	-4.17144566	4.19678577	-1.89446100
C	-1.00038366	6.39810977	-1.53851000
H	0.02416634	4.81789477	-2.58027200
C	-2.21969966	6.84467977	-1.02743400
H	-4.31488766	6.40240977	-0.77240200
H	-0.11138866	7.01661477	-1.45062600
H	-2.28183166	7.81213877	-0.53647900
H	-1.17694366	3.00495577	-3.72935100
Cl	-5.10257166	1.31822577	-1.96913600

1-benzyl-1-(pivaloyloxy)-pyrrolidin-1-ium chloride int-2

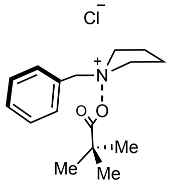

Geometry optimisation and frequency:

Sum of electronic and thermal Free Energies = -1289.332847 Hatrees
Thermal correction to Gibbs Free Energy = 0.331639 Hatrees

Single Point Energy:

Energy = -1289.526712 Hatrees

C	-1.21527784	0.27777777	0.00000000
C	-1.54239784	-1.11599123	0.53691200
C	-1.89459884	-1.96613523	-0.70963000
C	-1.68797884	-1.03410623	-1.89898800
H	-1.55961084	1.11124777	0.61158200
H	-0.15388284	0.38479477	-0.24810900
H	-0.67595984	-1.50995623	1.07192000
H	-2.38288384	-1.07860423	1.23373500

H	-1.24978584	-2.84088123	-0.81379900
H	-2.92928284	-2.31609023	-0.66425800
H	-0.63994284	-1.00262623	-2.21860100
H	-2.33984984	-1.19060523	-2.75593700
C	-1.43970884	1.46225877	-2.23465600
H	-0.36384384	1.26340277	-2.31784300
H	-1.91708984	1.29758677	-3.20006300
N	-1.92617484	0.34443377	-1.32923500
C	-1.72620484	2.83032777	-1.68414400
C	-2.88136984	3.51814577	-2.06709900
C	-0.82599984	3.44042777	-0.80394500
C	-3.15041484	4.78556677	-1.55469500
H	-3.56833784	3.06828077	-2.78073200
C	-1.09378684	4.70733977	-0.29060600
H	0.09279016	2.92677577	-0.52958500
C	-2.25979784	5.37852277	-0.66044300
H	-4.05142584	5.31121077	-1.85849800
H	-0.38797684	5.17368777	0.39084000
H	-2.46717984	6.36788777	-0.26201500
O	-3.28733384	0.50960077	-0.93432900
C	-4.29154784	0.46629777	-1.88011900
O	-4.07947284	0.34603077	-3.05363200
C	-5.63587284	0.57790377	-1.17404400
C	-6.73509984	0.60566677	-2.24099900
H	-7.70863784	0.68097377	-1.74573300
H	-6.62122584	1.46581177	-2.90849800
H	-6.72679884	-0.30445223	-2.84862800
C	-5.67570984	1.86630277	-0.33070900
H	-6.65516484	1.93727877	0.15385500
H	-4.90557884	1.86685877	0.44584100
H	-5.54004084	2.75618377	-0.95450900
C	-5.80229584	-0.65706523	-0.26498300
H	-6.78822984	-0.61547123	0.20973000
H	-5.74328084	-1.58720723	-0.84144600
H	-5.04328684	-0.68116023	0.52311100
Cl	1.81249516	0.03969877	-1.87227900

1-benzylpyrrolidine *exo*-TS2

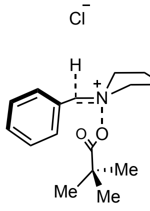

Geometry optimisation and frequency:

Sum of electronic and thermal Free Energies = -1289.288596 Hatrees
Thermal correction to Gibbs Free Energy = 0.322784 Hatrees

Single Point Energy:

Energy = -1289.481830 Hatrees

C	0.34722224	0.38194444	0.00000000

C	-0.26992676	-0.95434656	0.42006700
C	-0.66693476	-1.67311956	-0.89331500
C	-0.16929876	-0.77112856	-2.01806200
H	0.03314724	1.24541944	0.58629800
H	1.44334524	0.33711244	-0.02658000
H	0.45756924	-1.52966556	0.99697400
H	-1.14709976	-0.78667156	1.04710800
H	-0.21254076	-2.66262356	-0.97711800
H	-1.75079576	-1.78808956	-0.95558300
H	0.85605124	-1.02163756	-2.32290000
H	-0.80863476	-0.70607556	-2.89748500
C	0.45579624	1.58946244	-2.17219000
H	1.72146724	1.16636544	-2.25028700
H	0.10841724	1.50965444	-3.20185500
N	-0.06141976	0.57679744	-1.40891800
C	0.48170424	2.98023444	-1.63111800
C	-0.36138876	3.92614644	-2.22607600
C	1.32299724	3.37463544	-0.58468500
C	-0.39069176	5.23724944	-1.75721300
H	-1.00611476	3.62530144	-3.04867600
C	1.29274324	4.68645644	-0.11703700
H	2.01997424	2.66586644	-0.14637900
C	0.43243224	5.61827944	-0.69787500
H	-1.05741676	5.96014844	-2.21925900
H	1.95002924	4.98266544	0.69565500
H	0.41176024	6.64139844	-0.33254800
O	-1.86044976	0.91867144	-0.94014800
C	-2.64417376	1.26453344	-1.92226100
O	-2.35809476	1.14788444	-3.10970200
C	-3.98225876	1.85738444	-1.43042300
C	-4.91101476	2.08702944	-2.62597600
H	-5.85783676	2.51296344	-2.27287500
H	-4.46787676	2.78104744	-3.34709500
H	-5.12975676	1.14952244	-3.14829300
C	-3.68493776	3.19655944	-0.73182000
H	-4.62080076	3.63150344	-0.36096400
H	-3.00620076	3.06042544	0.11633600
H	-3.22896376	3.91293044	-1.42480100
C	-4.63384076	0.88217344	-0.43522100
H	-5.59514676	1.28824444	-0.09878100
H	-4.82288176	-0.09206556	-0.90194200
H	-3.99840376	0.72652044	0.44207300
Cl	3.24559924	0.68439144	-2.34391600

1-benzylpyrrolidine *endo*-TS2

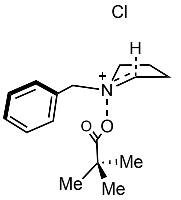

Geometry optimisation and frequency:

Sum of electronic and thermal Free Energies = -1289.288970 Hatrees

Thermal correction to Gibbs Free Energy = 0.322986 Hatrees

Single Point Energy:
Energy = -1289.482341 Hatrees

C	-0.55555558	-0.27777777	0.00000000
C	-0.17559958	-1.61112777	-0.61551600
C	0.52729142	-2.36645777	0.54055100
C	0.35108042	-1.46385677	1.76110700
H	-0.66827958	0.62338023	-0.60178400
H	-1.76092058	-0.43585477	0.48847700
H	-1.06927758	-2.13082577	-0.97321600
H	0.49260242	-1.46316877	-1.46842100
H	0.08812842	-3.34884377	0.72338900
H	1.58974842	-2.49905277	0.32449600
H	-0.56719858	-1.68447677	2.32226700
H	1.19313742	-1.41369277	2.45005300
C	-0.20238358	0.98350823	2.10765200
H	-1.17119858	0.68289023	2.53152900
H	0.55793542	0.94701823	2.88834800
N	0.12710842	-0.11928377	1.16990000
C	-0.29092458	2.33742723	1.45939900
C	0.81604642	3.19011223	1.47394500
C	-1.48003758	2.76448623	0.86033200
C	0.74565642	4.44431423	0.87282000
H	1.73307442	2.87191523	1.96331600
C	-1.55120558	4.01920123	0.25727300
H	-2.35242158	2.11348623	0.86769700
C	-0.43701458	4.85817223	0.25878200
H	1.61256942	5.09918723	0.88661000
H	-2.47821258	4.34253923	-0.20792400
H	-0.49277558	5.83704223	-0.20980500
O	1.85886042	0.31703223	0.56673700
C	2.87079542	0.41269823	1.38246200
O	2.79222342	0.30179123	2.60239500
C	4.19810542	0.68925023	0.64343500
C	5.34395342	0.75909923	1.65661400
H	6.28371942	0.95599523	1.12695600
H	5.18462742	1.56104823	2.38457900
H	5.44899742	-0.18179377	2.20644200
C	4.07660942	2.02754923	-0.10614100
H	5.01169042	2.23385123	-0.64013400
H	3.25934142	2.00366123	-0.83352700
H	3.89647042	2.85685323	0.58848500
C	4.44668742	-0.45245477	-0.35785700
H	5.40122042	-0.28909077	-0.87196400
H	4.49957542	-1.42161677	0.15289400
H	3.65245542	-0.50037477	-1.10931600
Cl	-3.27230458	-0.72732377	1.03020300

7.4 Predicted pK$_a$H data

pK$_a$H (Figures **S7**&**S8**) were predicted using the methods described in the literature[69] *via* the open access software developed by the Rowan Scientific Corporation.[70] These

calculations employed the xTB family of semiempirical methods as well as the AIMNet2 neural network potential.[27,71-75] At the time of running these calculations, the software could not take into account isotopes during pK_aH prediction.

Predicted pK_aH data for dextromethorphan and its derivatives

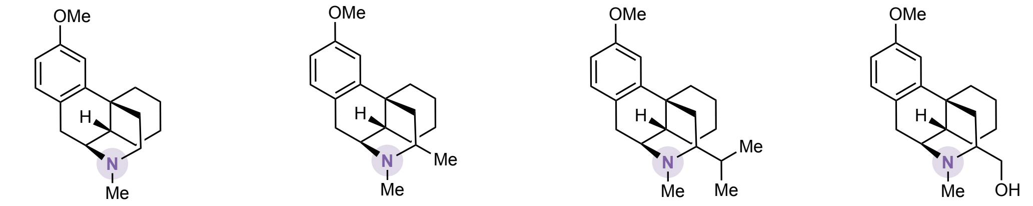

29 predicted pK_aH: 10.90 **17a** predicted pK_aH: 10.92 **17b** predicted pK_aH: 11.47 **17c** predicted pK_aH: 10.86

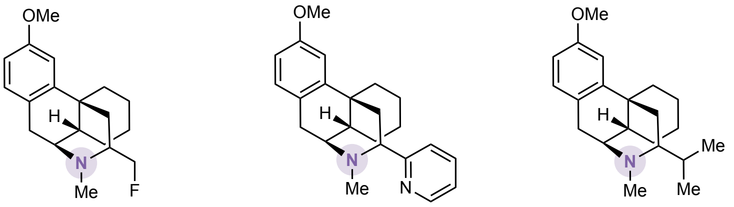

17d predicted pK_aH: 9.58 **17e** predicted pK_aH: 11.39 **17e** predicted pK_aH: 11.47

Figure S7: Predicted pK_aH values of the aliphatic nitrogen in dextromethorphan and its α-functionalised derivatives.

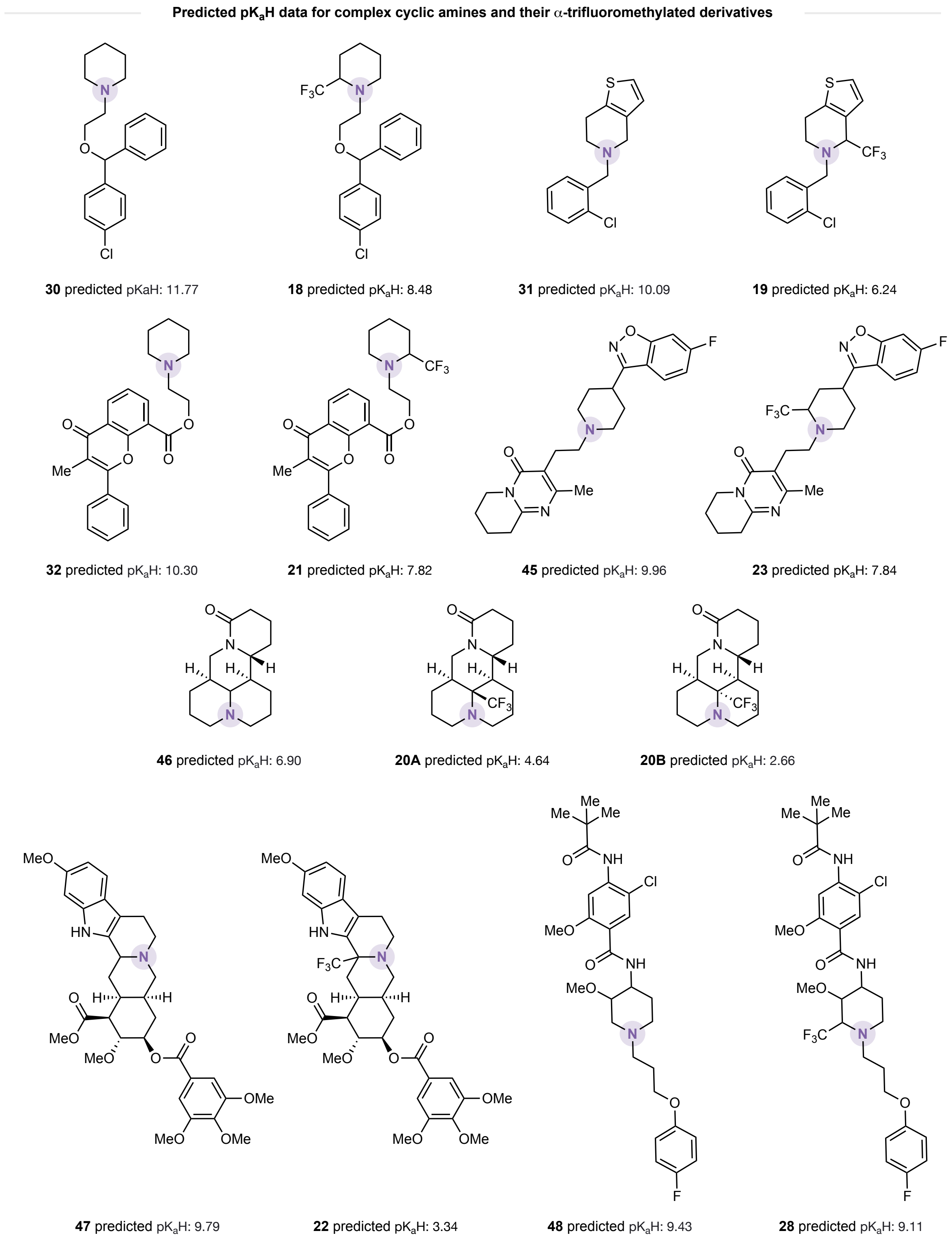

Figure S8: Predicted pK_aH values of the aliphatic nitrogen in complex cyclic alkylamines and their α-trifluoromethylated derivatives.

8. References

[28] H. P. Kokatla, P. F. Thomson, S. Bae, V. R. Doddi, M. K. Lakshman. Reduction of Amine N-Oxides by Diboron Reagents. *J. Org. Chem.,* **2011**, *76*, 7842-7848.

[29] *CRC Handbook of Chemistry and Physics*, 84th Ed. (Eds.: D. R. Lide), CRC Press LLC, **2003**, pp 2616.

[30] F. P. W. Agterberg, W. L. Driessen, J. Reedijk, H. Oeveringb, W. Buijs. Copper-catalyzed oxidative decarboxylation of aliphatic carboxylic acids. In *Studies in Surface Science and Catalysis*, Vol. 82 (Eds.: S. V. Bellón, V. C Corberán,) Elsevier, **1994**, pp 639-646.

[31] A. Fanizza, R. Berg, J. Hoffman, T. M. Gilbert, V. Ryzhov. Decarboxylation of fatty acids by ternary zinc cationic complexes studied by mass spectrometry and theoretical calculations. *Int. J. Mass Spectrom.* **2024**, *499*, 117216.

[32] J. M. Vohs, M. A. Barteau. Conversion of methanol, formaldehyde and formic acid on the polar faces of zinc oxide. *Surf. Sci.* **1986**, *176*, 91-114.

[33] D. Marcoux, P. Bindschädler, A. W. H. Speed, A. Chiu, J. E. Pero, G. A. Borg, D. A. Evans. Effect of Counterion Structure on Rates and Diastereoselectivities in α,β-Unsaturated Iminium-Ion Diels–Alder Reactions. *Org. Let.* **2011**, *13*, 3758-3761.

[34] D. Y. Ong, D. Fan, D. J. Dixon, S. Chiba. Transition-Metal-Free Reductive Functionalization of Tertiary Carboxamides and Lactams for α-Branched Amine Synthesis. *Angew. Chem. Int. Ed.,* **2020**, *59*, 11903-11907.

[35] K. J. Xiao, Y. Wang, K. Y. Ye, P. Q. Huang. Versatile One-Pot Reductive Alkylation of Lactams/Amides via Amide Activation: Application to the Concise Syntheses of Bioactive Alkaloids (±)-Bgugaine, (±)-Coniine, (+)-Preussin, and (−)-Cassine. *Chem. Eur. J.* **2010**, *16*, 12792-12796.

[36] P. Mateo, J. E. Cinqualbre, M. Meyer Mojzes, K. Schenk, P. Renaud. Reductive Alkylation of Tertiary Lactams via Addition of Organocopper (RCu) Reagents to Thioiminium Ions. *J. Org. Chem.* **2017**, *82*, 12318-12327.

[37] G. Stamatiou, G. B. Foscolos, G. Fytas, A. Kolocouris, N. Kolocouris, C. Pannecouque, M. Witvrouw, E. Padalko, J. Neyts, E. D. Clercq. Heterocyclic rimantadine analogues with antiviral activity. *Bioorg. Med. Chem.,* **2003**, *11*, 5485-5492.

[38] H. Deng, S. Kooijman, A. M. C.H. Van den Nieuwendijk, D. Ogasawara, T. Van der Wel, F. van Dalen, M. P. Baggelaar, F. J. Janssen, R. J. B. H. MN. Van Den Berg, H. Den Dulk, B. F. Cravatt, H. S. Overkleeft, P. C. N. Rensen, M. Van der Stelt. Triazole Ureas Act as Diacylglycerol Lipase Inhibitors and Prevent Fasting-Induced Refeeding. *J. Med. Chem.* **2017**, *60*, 428-440.

[39] G. Oss, S. D. de Vos, K. N. H. Luc, J. B. Harper, T. V. Nguyen. Tropylium-Promoted Oxidative Functionalization of Tetrahydroisoquinolines. *J. Org. Chem.* **2018**, *83*, 1000-1010.

[40] J. Zhang, S. Chang. cine-Silylative Ring-Opening of α-Methyl Azacycles Enabled by the Silylium-Induced C–N Bond Cleavage. *J. Am. Chem. Soc.* **2020**, *142*, 12585-12590.

[41] A. Massaro, A. Mordini, A. Mingardi, J. Klein, D. A. Andreotti. New Sequential Intramolecular Cyclization Based on the Boekelheide Rearrangement. *Eur. J. Org. Chem.* **2011**, *2011*, 271-279.

[42] A. Howarth, K. Ermanis, J. M. Goodman. DP4-AI automated NMR data analysis: straight from spectrometer to structure. *Chem. Sci.* **2020**, *11*, 4351-4359.
[43] H. T. Huang, T. C. Lacy, B. Błachut, G. X. Ortiz, Q. Wang. An Efficient Synthesis of Fluorinated Azaheterocycles by Aminocyclization of Alkenes. *Org. Lett.* **2013**, *15*, 1818-1821.
[44] Y. Sasano, S. Nagasawa, M. Yamazaki, M. Shibuya, J. Park, Y. Iwabuchi. Highly Chemoselective Aerobic Oxidation of Amino Alcohols into Amino Carbonyl Compounds. *Angew. Chem. Int. Ed.* **2014**, *53*, 3236-3240.
[45] W. Wichitnithad, J. P. O'Callaghan, D. B. Miller, B. C. Train, P. S. Callery. Time-dependent slowly-reversible inhibition of monoamine oxidase A by N-substituted 1,2,3,6-tetrahydropyridines. *Bioorg. Med. Chem.*, **2011**, *19*, 7482-7492.
[46] M. Tajbakhsh, R. Hosseinzadeh, H. Alinezhad, S. Ghahari, A. Heydari, S. Khaksar. Catalyst-Free One-Pot Reductive Alkylation of Primary and Secondary Amines and N,N-Dimethylation of Amino Acids Using Sodium Borohydride in 2,2,2-Trifluoroethanol. *Synthesis,* **2011**, *2011*, 490-496.
[47] A. Liu, C. Ni, Q. Xie, J. Hu. TMSCF2Br-Enabled Fluorination–Aminocarbonylation of Aldehydes: Modular Access to α-Fluoroamides. *Angew. Chem. Int. Ed.,* **2022**, *61*, e202115467.
[48] J. A. R. Tilden, A. T. Lubben, S. B. Reeksting, G. Kociok-Köhn, C. G. Frost. Pd(II)-Mediated C−H Activation for Cysteine Bioconjugation. *Chem. Eur. J.* **2022**, *28*, e202104385.
[49] B. P. Reddy, K. R. Reddy, A. P. Reddy, G. L. D. Krupadanam, M. Venkati, N. Sudhakar, L. V. Subrahmanyam. Novel betulinic substituted amide derivatives as hiv inhibitors. WO 2017 017630, **2017**.
[50] Z. Li, H. J. Feiten, J. B. van Beilen, W. Duetz, B. Witholt. Preparation of Optically Active N-Benzyl-3-hydroxypyrrolidine by Enzymatic Hydroxylation. *Tetrahedron: Asymmetry,* **1999**, *10*, 1323-1333.
[51] A. Tota, M. Colella, C. Carlucci, A. Aramini, G. Clarkson, L. Degennaro, J. A. Bull, J. R. Luisi. N−N Bond Formation Using an Iodonitrene as an Umpolung of Ammonia: Straightforward and Chemoselective Synthesis of Hydrazinium Salts. *Adv. Synth. Cat.* **2021**, *363*, 194-199.
[52] A. Noble, D. W. C. MacMillan. Photoredox α-Vinylation of α-Amino Acids and N-Aryl Amines. *J. Am. Chem. Soc.* **2014**, *136*, 11602-11605.
[53] E. Tayama, G. Shimizu, R. Nakao. Base-induced Sommelet–Hauser rearrangement of N-(pyridinylmethyl) tetraalkylammonium salts. *Tetrahedron,* **2022**, *111*, 132721.
[54] T. Thierry, E. Pfund, T. Lequeux. Metal-Free Aminomethylation of Aromatic Sulfones Promoted by Eosin Y. *Chem. Eur. J.* **2021**, *27*, 14826-14830.
[55] A. Singh, A. Arora, J. D. Weaver. Photoredox-Mediated C–H Functionalization and Coupling of Tertiary Aliphatic Amines with 2-Chloroazoles. *Org. Lett.* **2013**, *15*, 5390-5393.
[56] Y. Byun, K. Moon, J. Park, P. Ghosh, N. K. Mishra, I. S. Kim. Methylene Thiazolidinediones as Alkylation Reagents in Catalytic C–H Functionalization: Rapid Access to Glitazones. *Org. Lett.,* **2022**, *24*, 8578-8583.
[57] V. Harawa, T. W. Thorpe, J. R. Marshall, J. J. Sangster, A. K. Gilio, L. Pirvu, R. S. Heath, A. Angelastro, J. D. Finnigan, S. J. Charnock, J. W. Nafie, G. Grogan, R. C. Whitehead, N. J. Turner**.** Synthesis of Stereoenriched Piperidines via Chemo-

Enzymatic Dearomatization of Activated Pyridines. *J. Am. Chem. Soc.* **2022**, *144*, 21088-21095.
[58] C. Houle, P. R. Savoie, C. Davies, D. Jardel, P. A. Champagne, B. Bibal, J. F. Paquin. Thiourea-Catalyzed C−F Bond Activation: Amination of Benzylic Fluorides. *Chem. Eur. J.* **2020**, *26*, 10620-10625.
[59] K. Jozwiak, K. M. Targowska-Duda, A. A. Kaczor, J. Kozak, A. Ligeza, E. Szacon, T. M. Wrobel, B. Budzynska, G. Biala, E. Fornal, A. Poso, I. W. Wainer amd D. Matosiuk. Synthesis, in vitro and in vivo studies, and molecular modeling of N-alkylated dextromethorphan derivatives as non-competitive inhibitors of α3β4 nicotinic acetylcholine receptor. *Bioorg. Med. Chem.,* **2014**, *22*, 6846-6856.
[60] J. Jeong, D. Lee, S. Chang. Copper-catalyzed oxygen atom transfer of N-oxides leading to a facile deoxygenation procedure applicable to both heterocyclic and amine N-oxides. *Chem. Comm.,* **2015**, *51*, 7035-7038.
[61] A. K. Seitz, P. J. Kohlpaintner, T. van Lingen, M. Dyga, F. Sprang, M. Zirbes, S. R. Waldvogel, L. J. Gooßen. Concentrated Aqueous Peroxodicarbonate: Efficient Electrosyn- thesis and Use as Oxidizer in Epoxidations, S-, and N-Oxidations. *Angew. Chem. Int. Ed.* **2022**, *61*, e202117563.
[62] T. Rosenau, P. Schmid, P. Kosma. On the non-classical course of Polonowski reactions of N-benzylmorpholine-N-oxide (NBnMO). *Tetrahedron,* **2005**, *61*, 3483-3487.
[63] H. Petride, A. Corbu, O. Costan, C. Florea, V. Marin, A. Petride, E. Şerban. N-oxides of some N-benzyl azacycloalkanes. Preferred conformation by NMR spectroscopy. *Rev. Roum. Chim.* **2005**, *50*, 633-640.
[64] N. L. Magann, E. Westley, M. J. Sowden, M. G. Gardiner, M. S. Sherburn**.** Total synthesis of matrine alkaloids. *J. Am. Chem. Soc*. **2022**, *144*, 43, 19695–19699
[65] S. Funahashi, Y. S. Yamaguchi, M. Tanaka. Kinetics and Mechanism of Copper(II), Zinc(II), and Cadmium(II) Incorporation into 5,10,15,20-Tetraphenylporphine and N-Methyl-5,10,15,20-tetraphenylporphine in N,N-Dimethylformamide. *Bull. Chem. Soc. Jpn.* **1986**, *57*, 204-208.
[66] A. Krasovskiy, P. Knochel. Convenient Titration Method for Organometallic Zinc, Magnesium, and Lanthanide- Reagents. *Synthesis,* **2006**, *2006*, 0890-0891.
[67] S. Ruppenthal, R. Brückner. Symmetric Diarylsulfoxides as Asymmetric Sulfinylating Reagents for Dialkylmagnesium Compounds. *J. Org. Chem.* **2015**, 80, 897-910.
[68] A. M. Krieger, E. A. Pidko. The Impact of Computational Uncertainties on the Enantioselectivity Predictions: A Microkinetic Modeling of Ketone Transfer Hydrogenation with a Noyori-type Mn-diamine Catalyst. *Chem. Cat. Chem.* **2021**, *13*, 3517-3524.
[69] C. Wagen, A. Wagen. Efficient and Accurate pKa Prediction Enabled by Pre-Trained Machine-Learned Interatomic Potentials. *ChemRxiv,* **2024**. DOI: 10.26434/chemrxiv-2024-8489b (accessed 18.07.2024).
[70] D. Anstine, R. Zubatyuk, O. Isayev. AIMNet2: A Neural Network Potential to Meet your Neutral, Charged, Organic, and Elemental-Organic Needs. *ChemRxiv,* **2024**. DOI: 10.26434/chemrxiv-2023-296ch-v2 (accessed 18 July 2024).
[71] S. Grimme, C. Bannwarth, P. Shushkov. A Robust and Accurate Tight-Binding Quantum Chemical Method for Structures, Vibrational Frequencies, and

Noncovalent Interactions of Large Molecular Systems Parameterized for All spd-Block Elements (Z = 1-86). *J. Chem. Theory Comput.* **2017**, *13*, 1989-2009.
[72] C. Bannwarth, S. Ehlert, S. Grimme. GFN2-xTB—An Accurate and Broadly Parametrized Self-Consistent Tight-Binding Quantum Chemical Method with Multipole Electrostatics and Density-Dependent Dispersion Contributions; *J. Chem. Theory Comput.* **2019**, *15*, 1652–1671.
[73] S. Grimme, C. Bannwarth. Ultra-fast computation of electronic spectra for large systems by tight-binding based simplified Tamm-Dancoff approximation (sTDA-xTB). *J. Chem. Phys.* **2016**, *145*, 054103.
[74] S. Spicher, S. Grimme. Robust atomistic modeling of materials, organometallic and biochemical systems; *Angew. Chem. Int. Ed.* **2020**, *59,* 15665.
[75] E. Caldeweyher, S. Ehlert, A. Hansen, H. Neugebauer, S. Spicher, C. Bannwarth, S. Grimme. A generally applicable atomic-charge dependent London dispersion correction*; J. Chem. Phys.* **2019**, *150*, 154122.

9. NMR spectral data

^{1}H NMR (400 MHz, CD_2Cl_2) of 1-benzyl-2,3,4,5-tetrahydropyridin-1-ium chloride (**3a**):

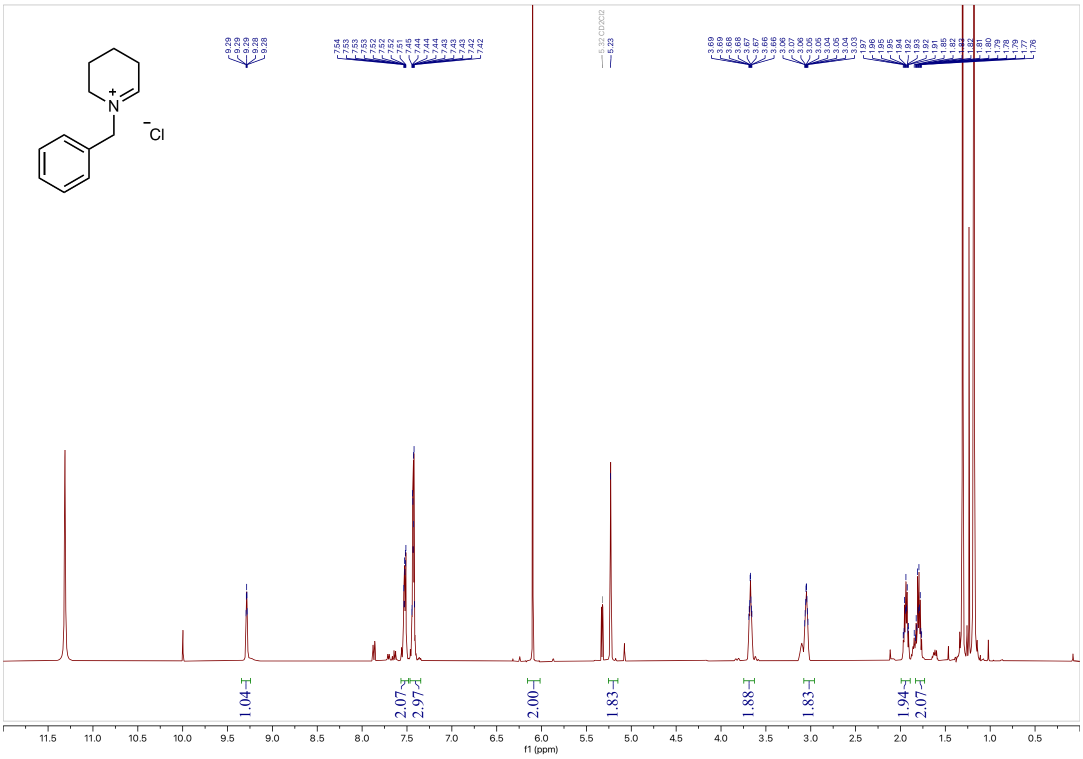

^{13}C{H} NMR (101 MHz, CD_2Cl_2) of *N*-benzyl-2,3,4,5-tetrahydropyridin-1-ium chloride (**3a**):

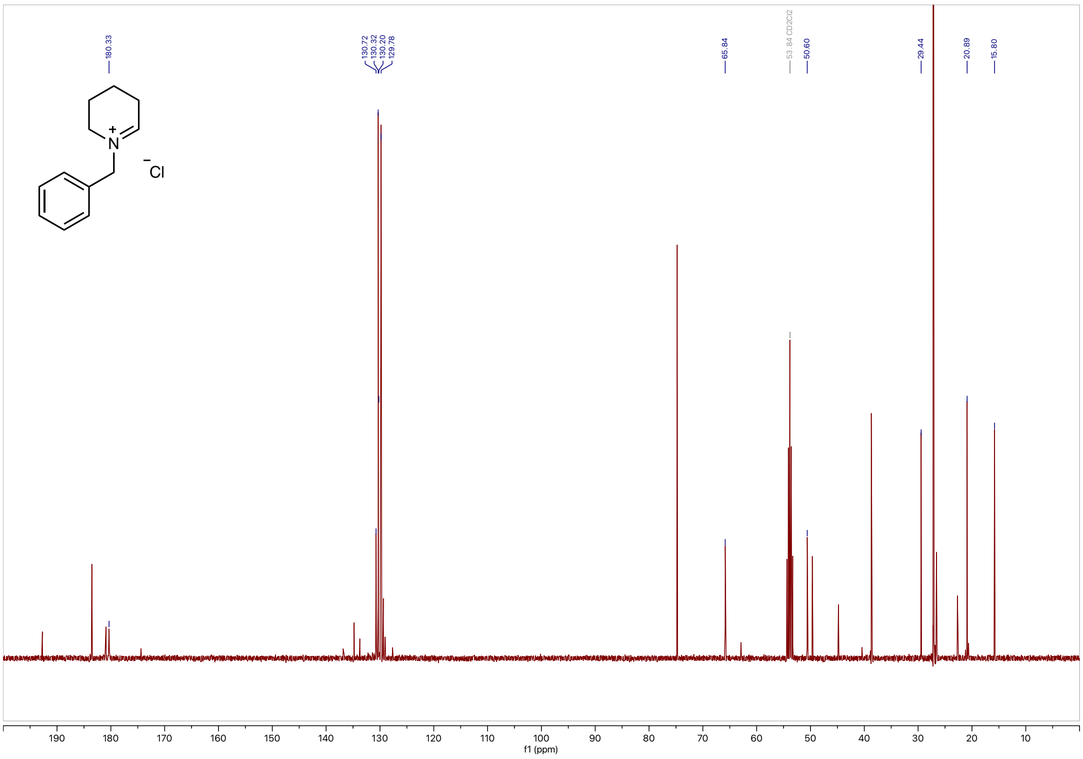

^{1}H NMR (400 MHz, CD_2Cl_2) of 1-benzyl-2,3,4,5-tetrahydropyridin-1-ium chloride (**3a**, 1 mmol):

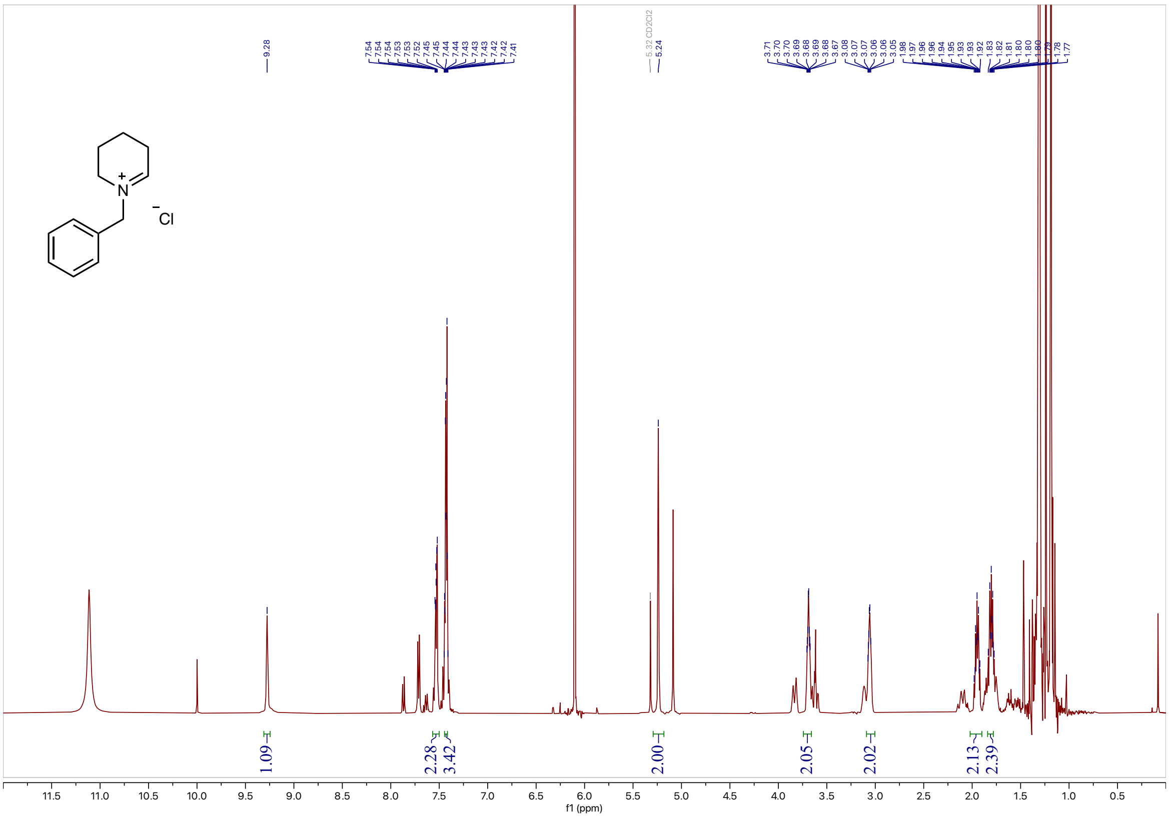

^{13}C{H} NMR (101 MHz, CD_2Cl_2) of *N*-benzyl-2,3,4,5-tetrahydropyridin-1-ium chloride (**3a,** 1 mmol):

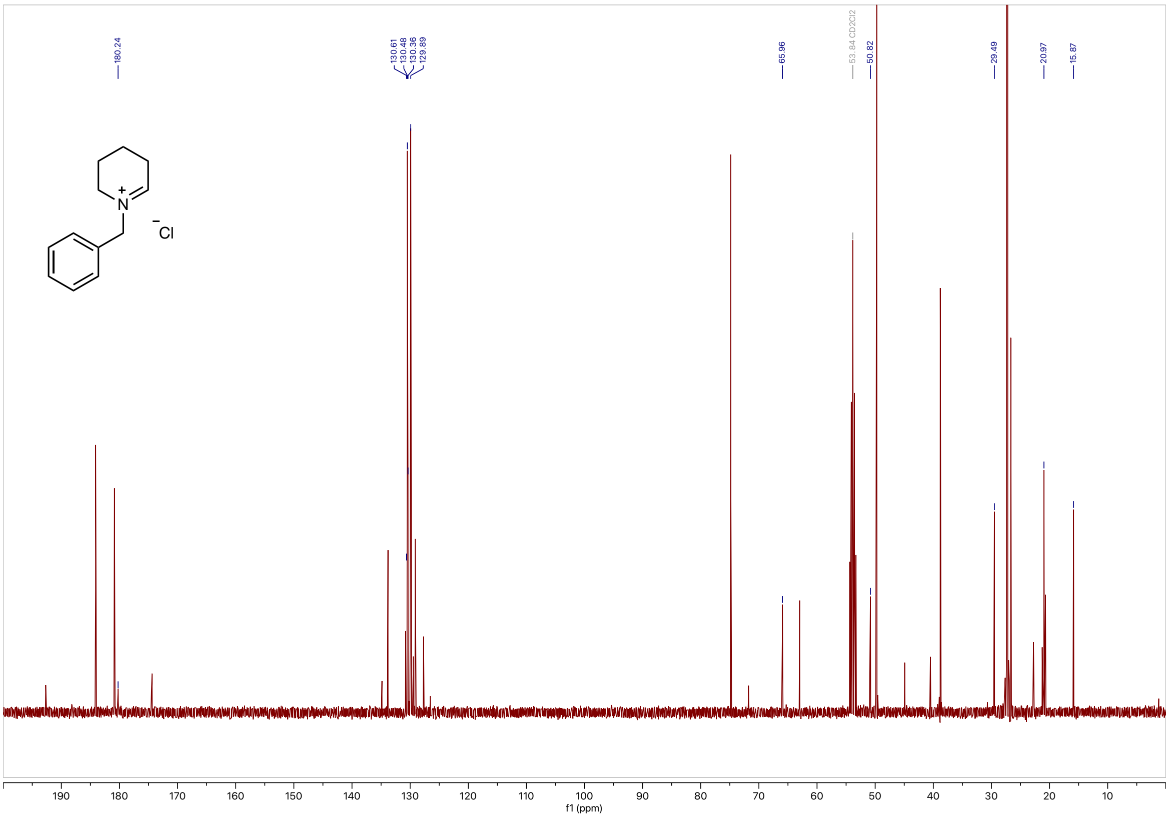

^1H NMR (400 MHz, CD_2Cl_2) of 1-(3-phenylpropyl)-2,3,4,5-tetrahydropyridin-1-ium chloride (**3b**):

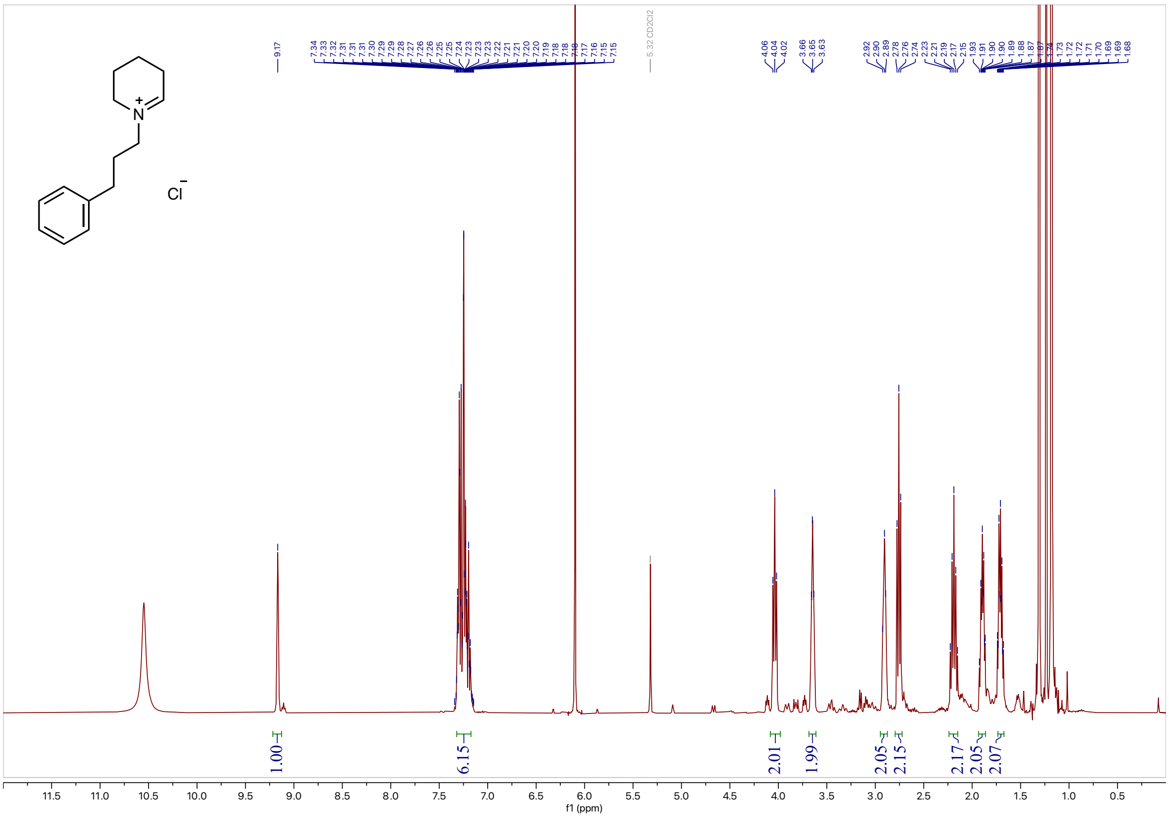

$^{13}C\{H\}$ NMR (101 MHz, CD_2Cl_2) of 1-(3-phenylpropyl)-2,3,4,5-tetrahydropyridin-1-ium chloride (**3b**):

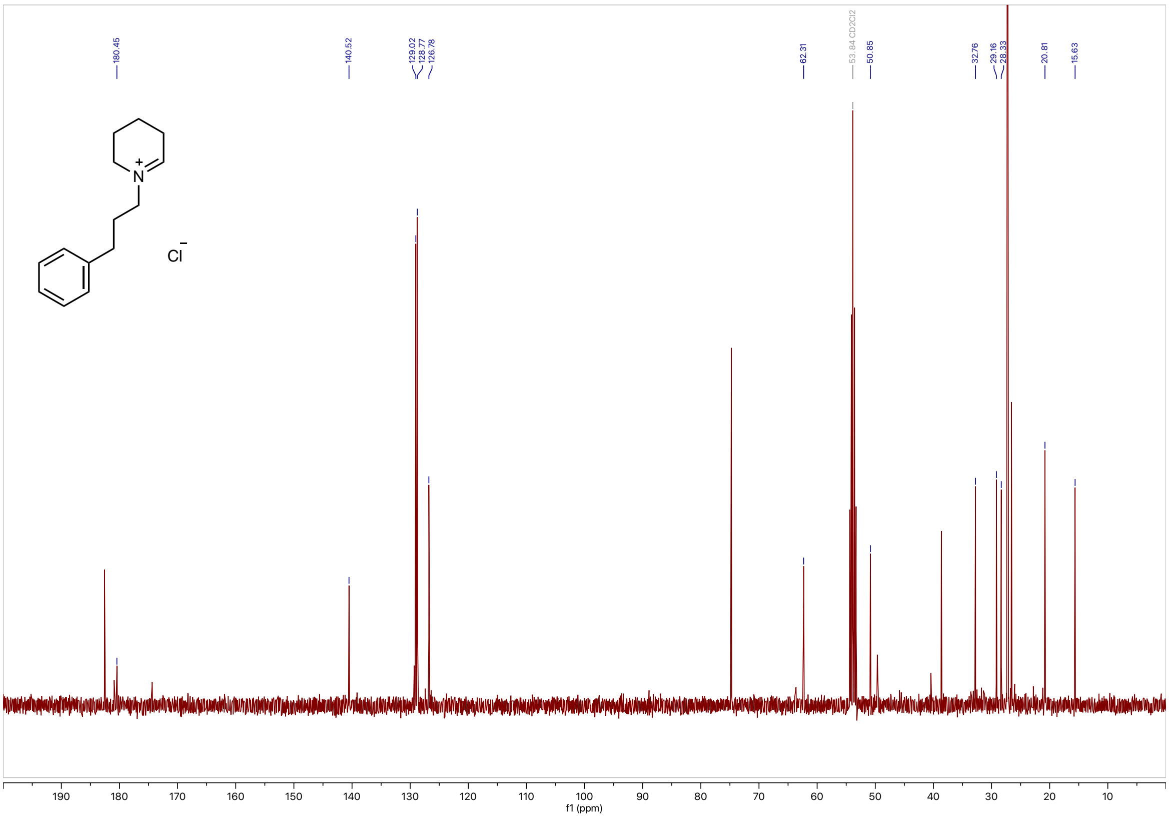

^{1}H NMR (400 MHz, CD_2Cl_2) of 1-methyl-2,3,4,5-tetrahydropyridin-1-ium chloride (**3c**):

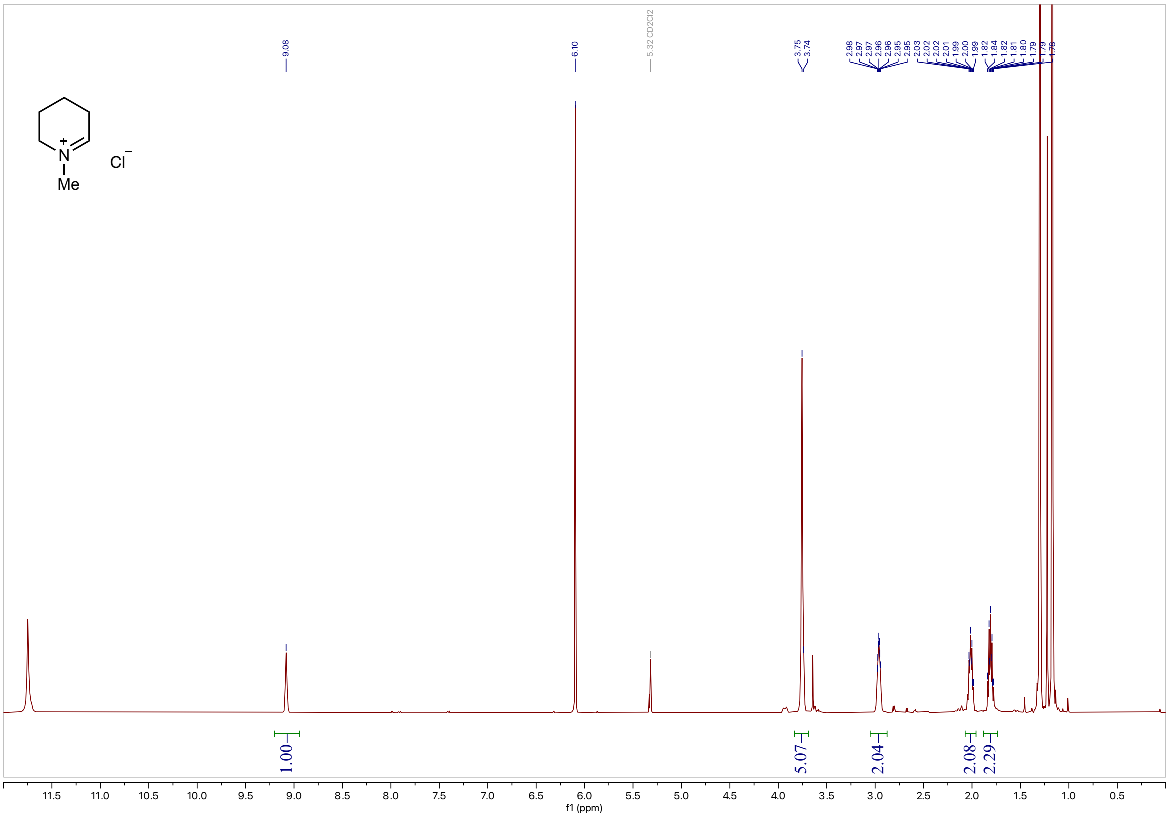

$^{13}C\{H\}$ NMR (101 MHz, CD_2Cl_2) of 1-methyl-2,3,4,5-tetrahydropyridin-1-ium chloride (**3c**):

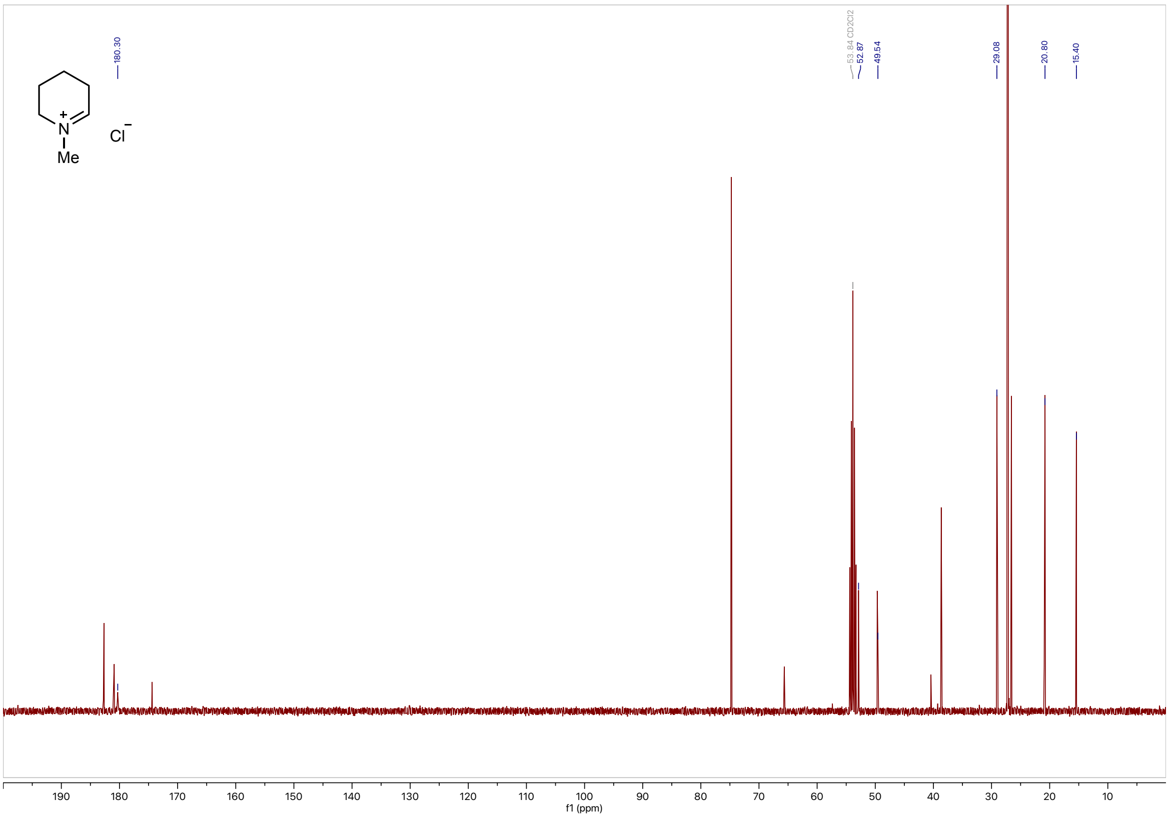

^{1}H NMR (700 MHz, CD_2Cl_2) of 1-cyclohexyl-2,3,4,5-tetrahydropyridin-1-ium chloride (**3d**):

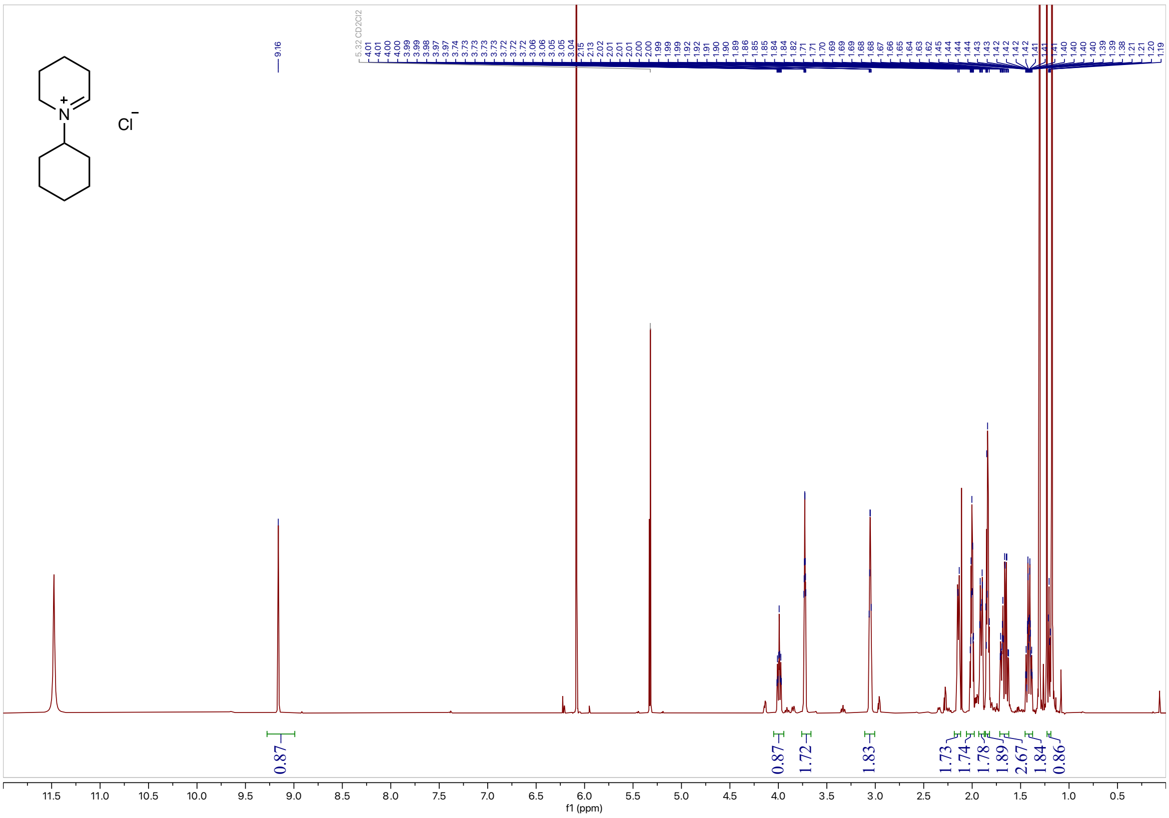

$^{13}C\{H\}$ NMR (176 MHz, CD_2Cl_2) of 1-cyclohexyl-2,3,4,5-tetrahydropyridin-1-ium chloride (**3d**):

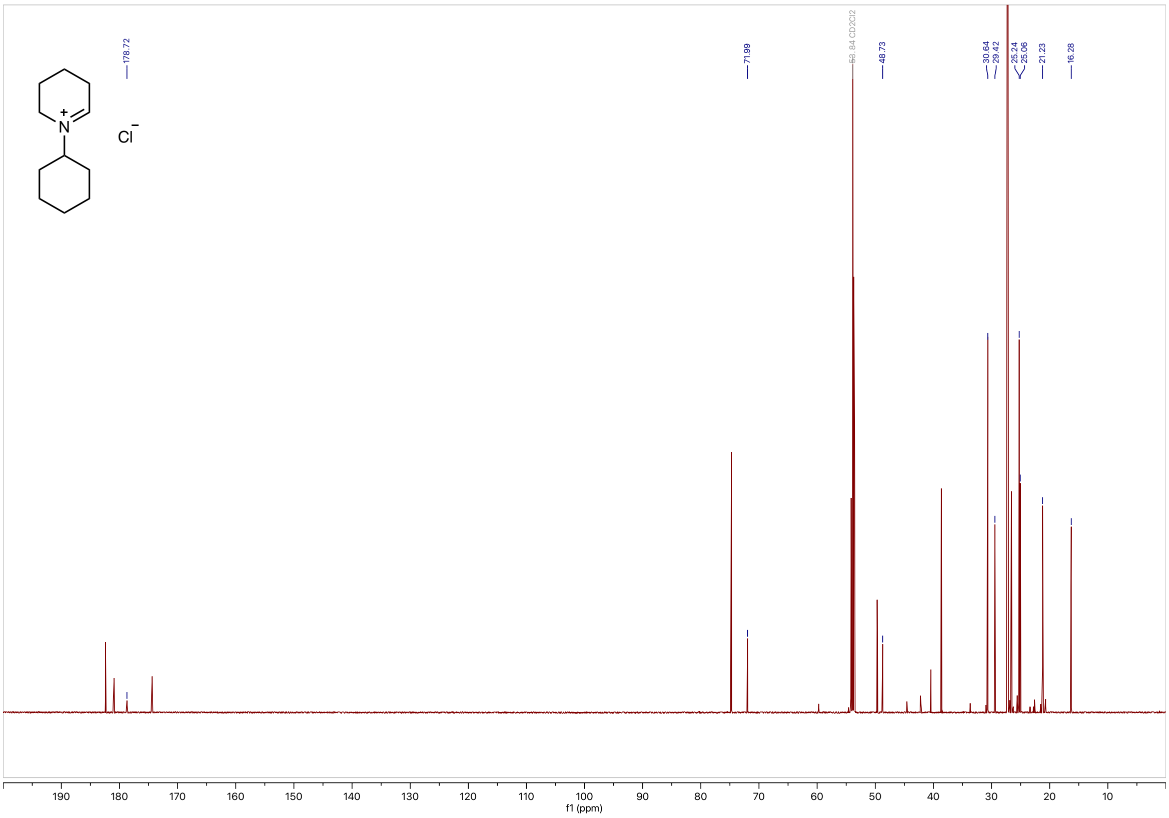

^{1}H NMR (700 MHz, CD_2Cl_2) of 1-phenyl-2,3,4,5-tetrahydropyridin-1-ium chloride (**3e**):

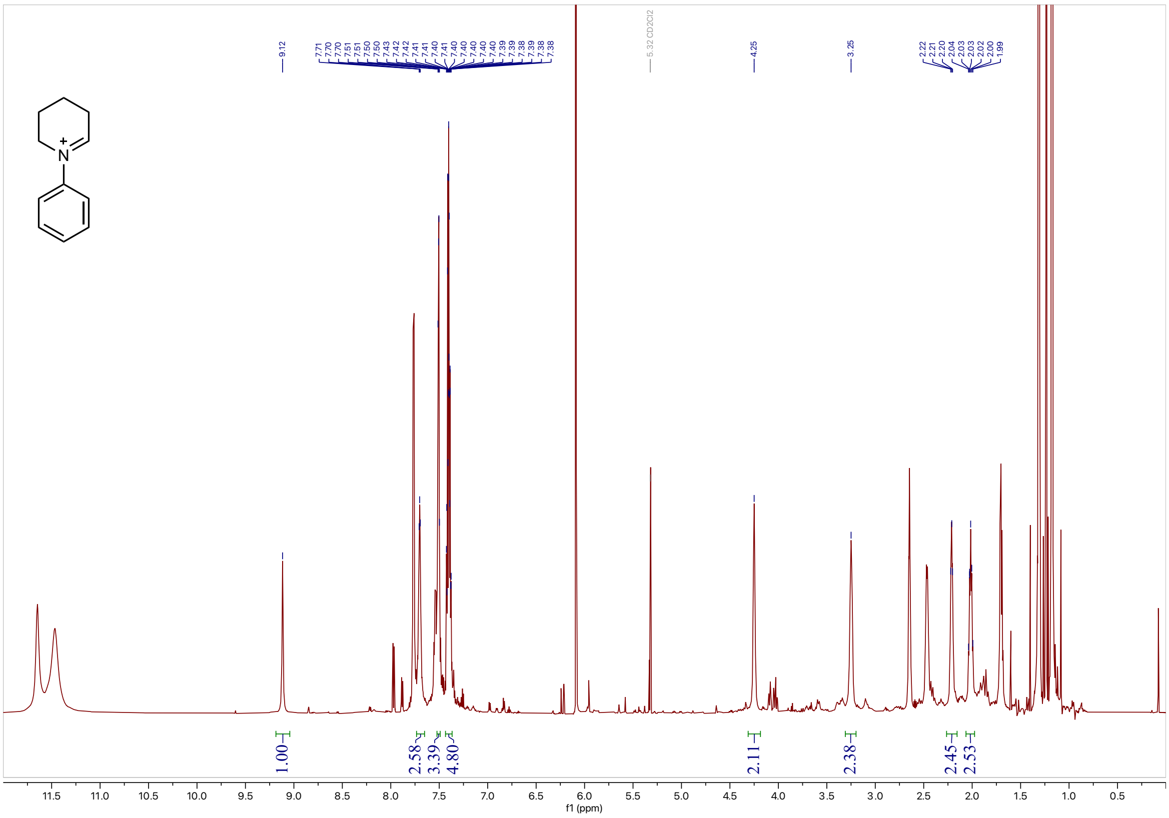

$^{13}C\{H\}$ NMR (176 MHz, CD_2Cl_2) of 1-phenyl-2,3,4,5-tetrahydropyridin-1-ium chloride (**3e**):

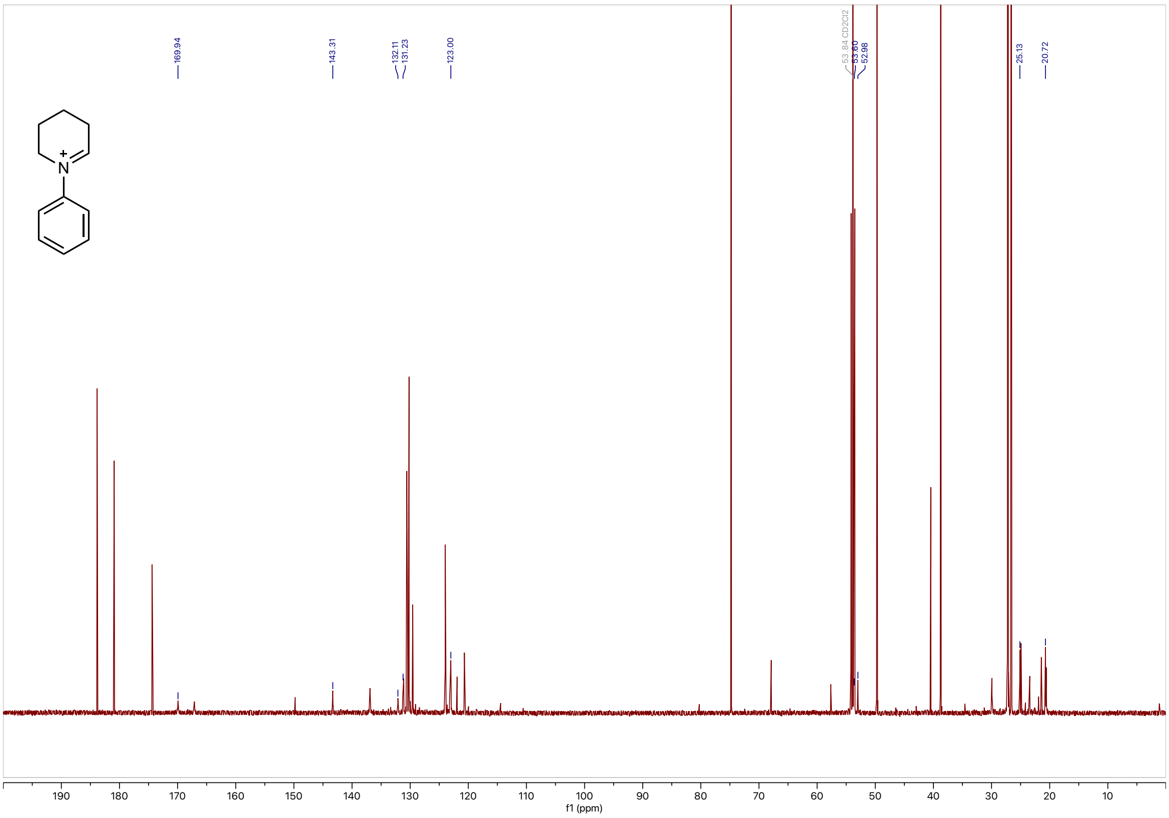

^{1}H NMR (400 MHz, CD_2Cl_2) of 1-benzyl-2-methyl-2,3,4,5-tetrahydropyridin-1-ium chloride (**3f**):

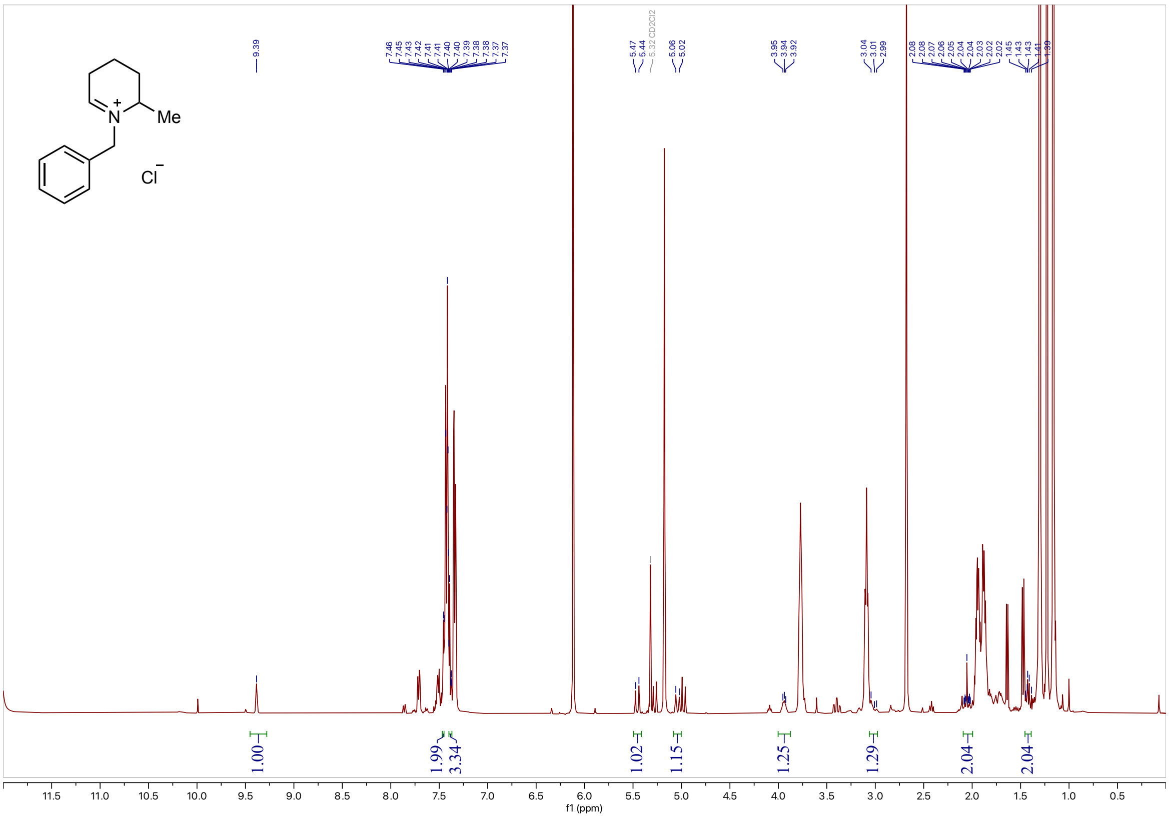

$^{13}C\{H\}$ NMR (101 MHz, CD_2Cl_2) of 1-benzyl-2-methyl-2,3,4,5-tetrahydropyridin-1-ium chloride (**3f**):

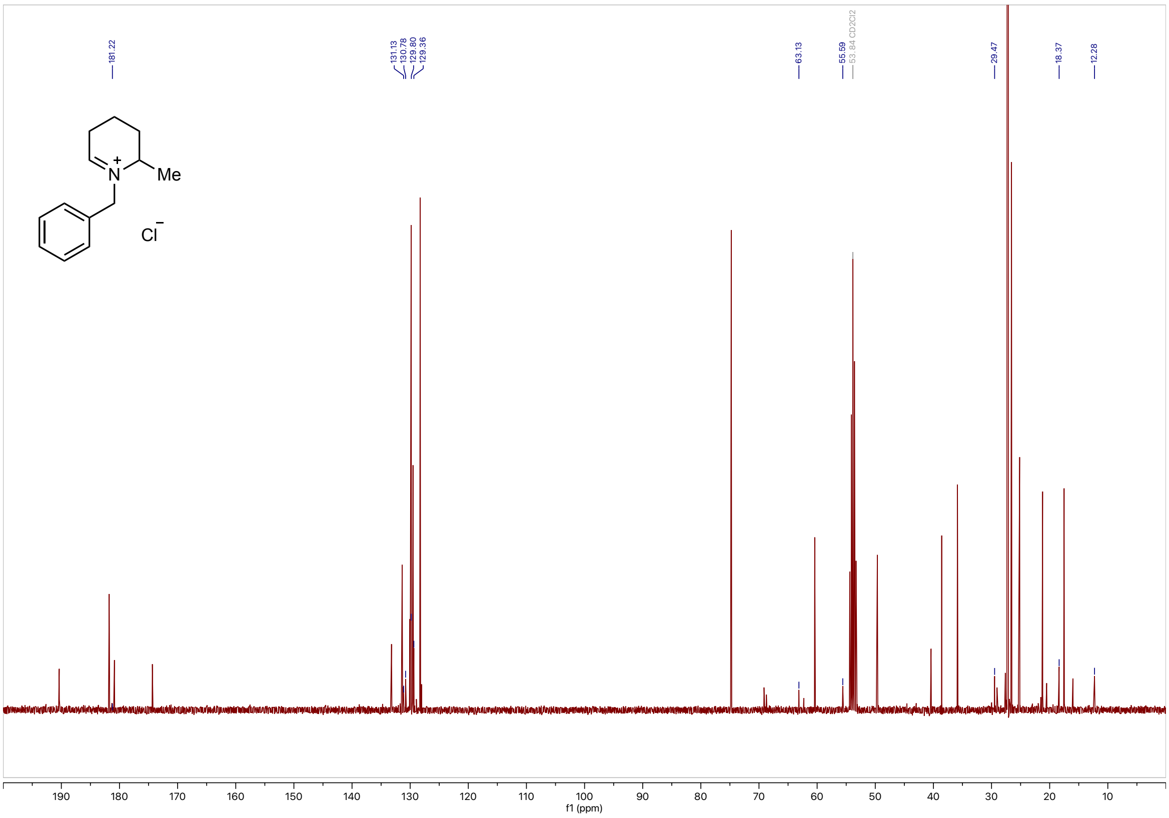

^{1}H NMR (400 MHz, CD_2Cl_2) of 1-benzyl-6-methyl-2,3,4,5-tetrahydropyridin-1-ium chloride (**3f'**):

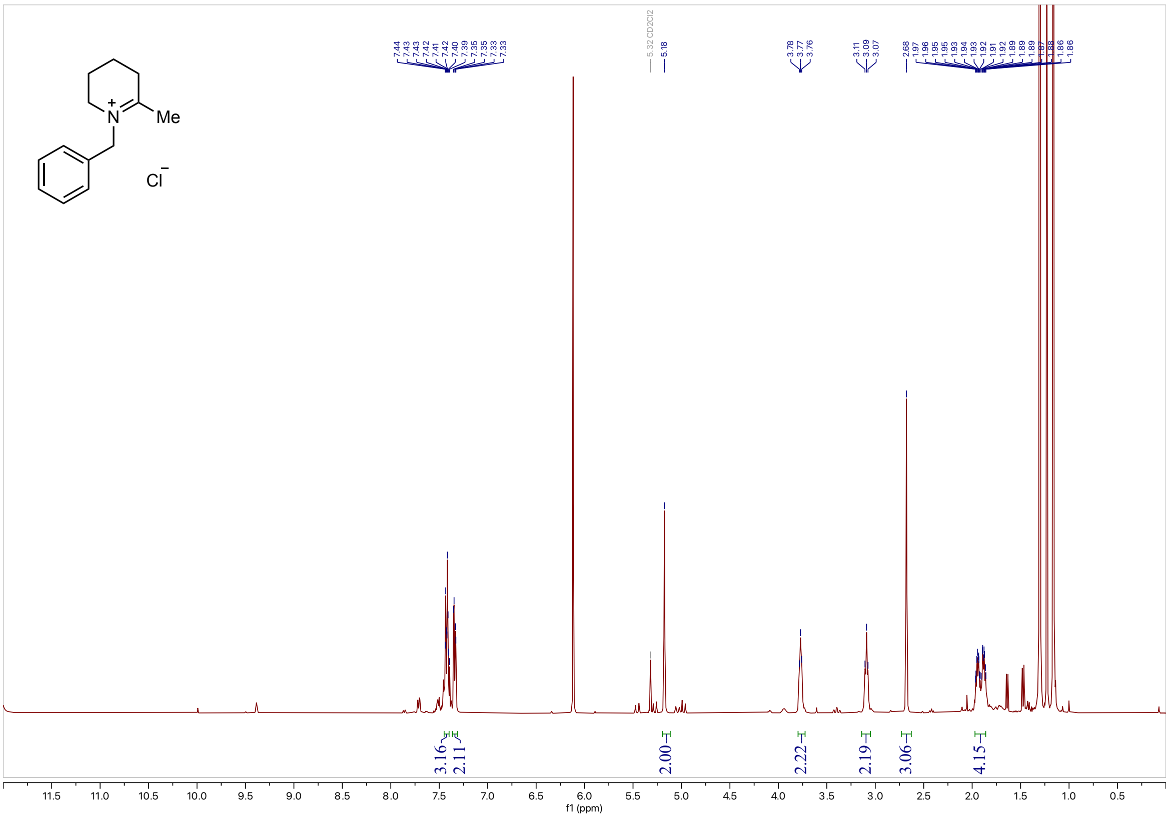

$^{13}C\{H\}$ NMR (101 MHz, CD_2Cl_2) of 1-benzyl-6-methyl-2,3,4,5-tetrahydropyridin-1-ium chloride (**3f'**):

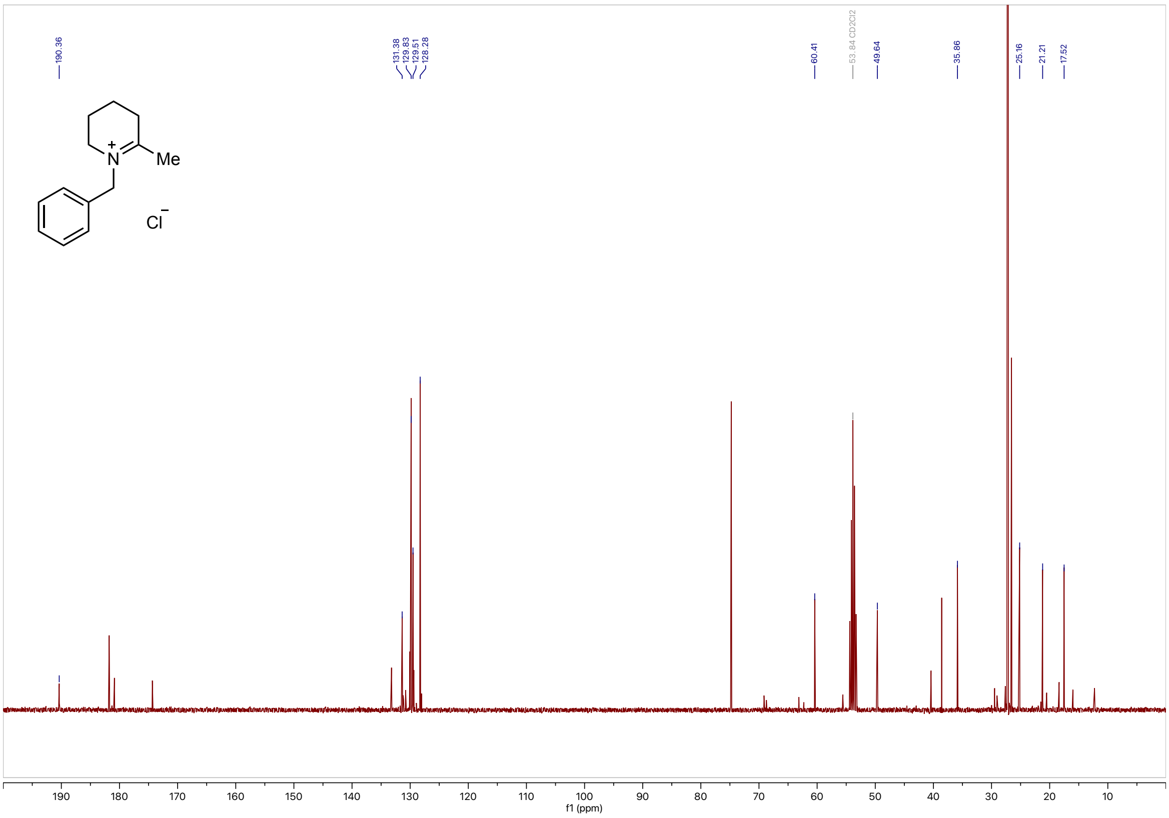

^{1}H NMR (700 MHz, CD_2Cl_2) of 1-benzyl-5-methyl-2,3,4,5-tetrahydropyridin-1-ium chloride (**3g**):

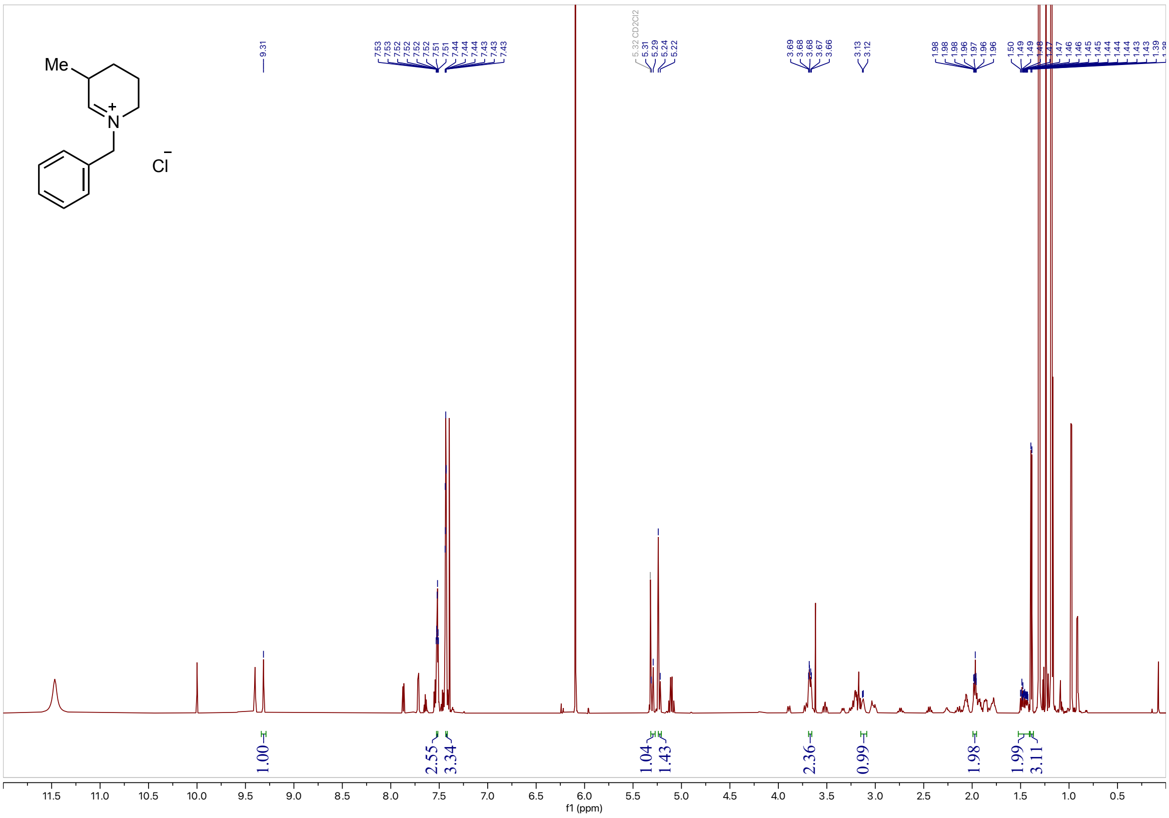

$^{13}C\{H\}$ NMR (176 MHz, CD_2Cl_2) of 1-benzyl-5-methyl-2,3,4,5-tetrahydropyridin-1-ium chloride (**3g**):

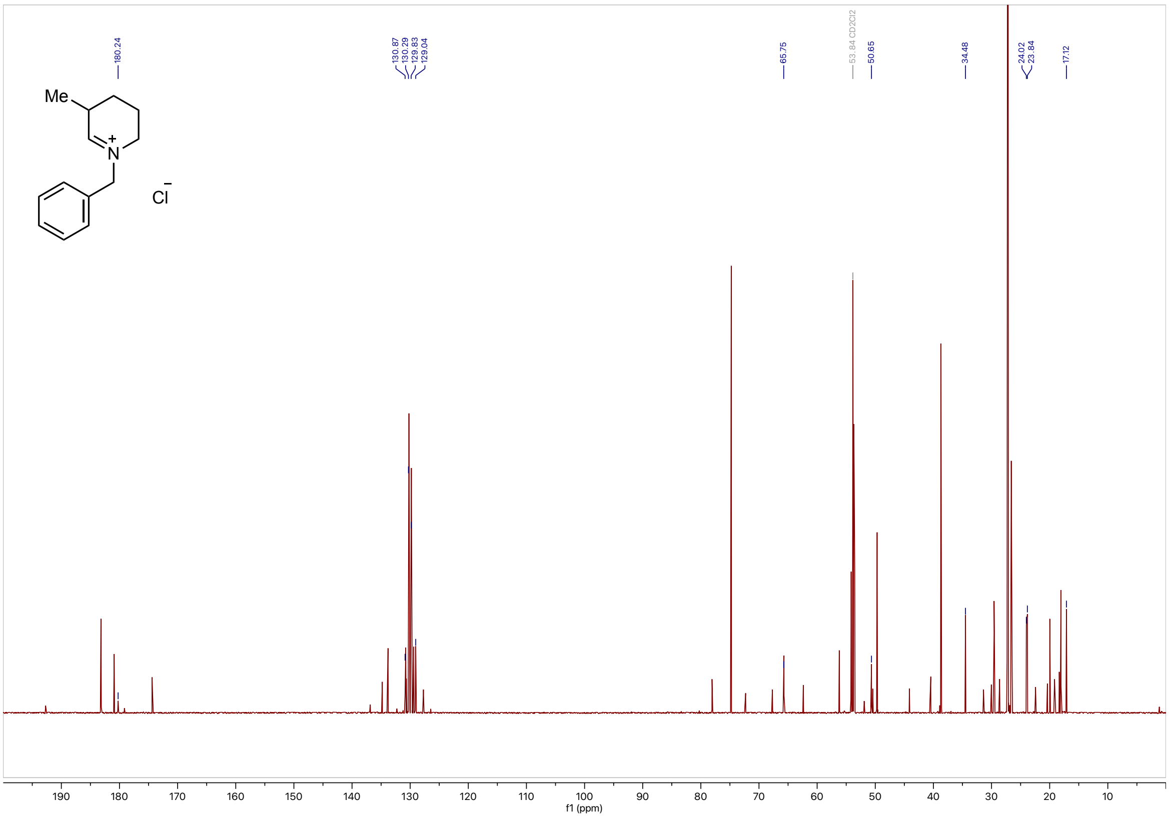

^{1}H NMR (700 MHz, CD_2Cl_2) of 1-benzyl-3-methyl-2,3,4,5-tetrahydropyridin-1-ium chloride (**3g’**):

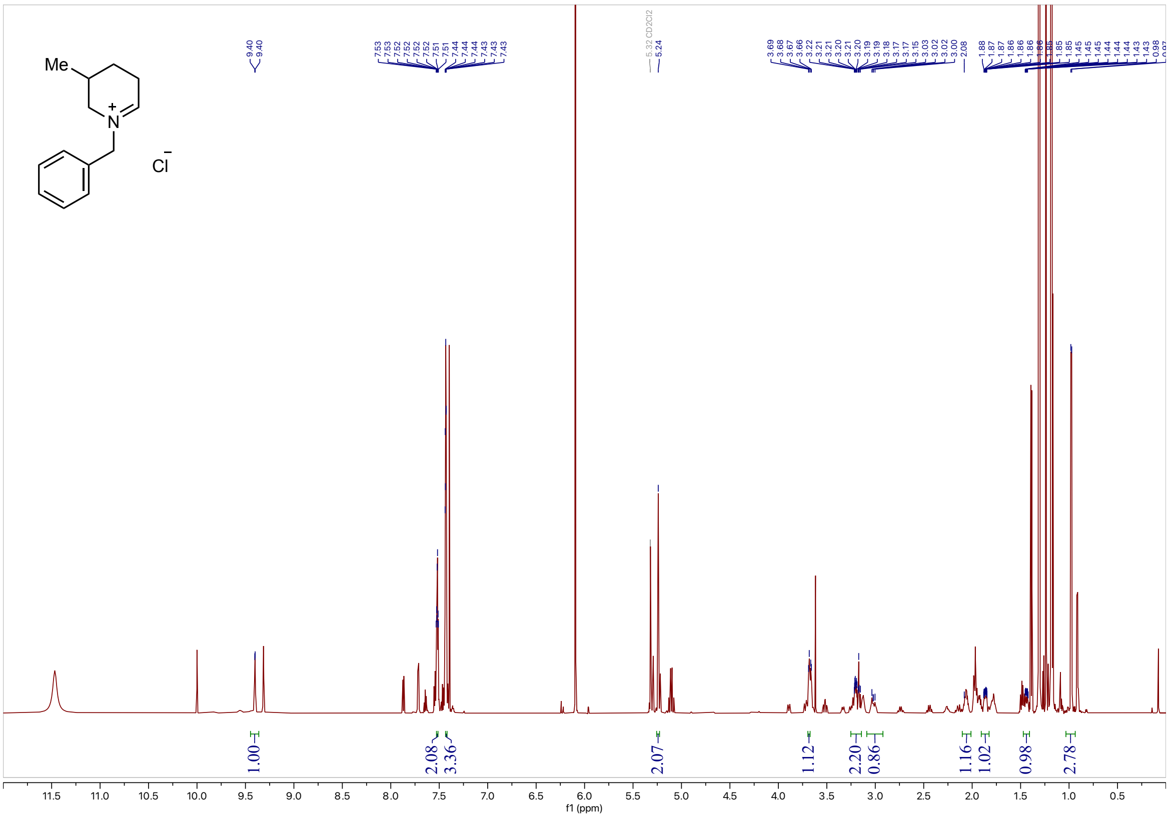

$^{13}C\{H\}$ NMR (176 MHz, CD_2Cl_2) of 1-benzyl-3-methyl-2,3,4,5-tetrahydropyridin-1-ium chloride (**3g’**):

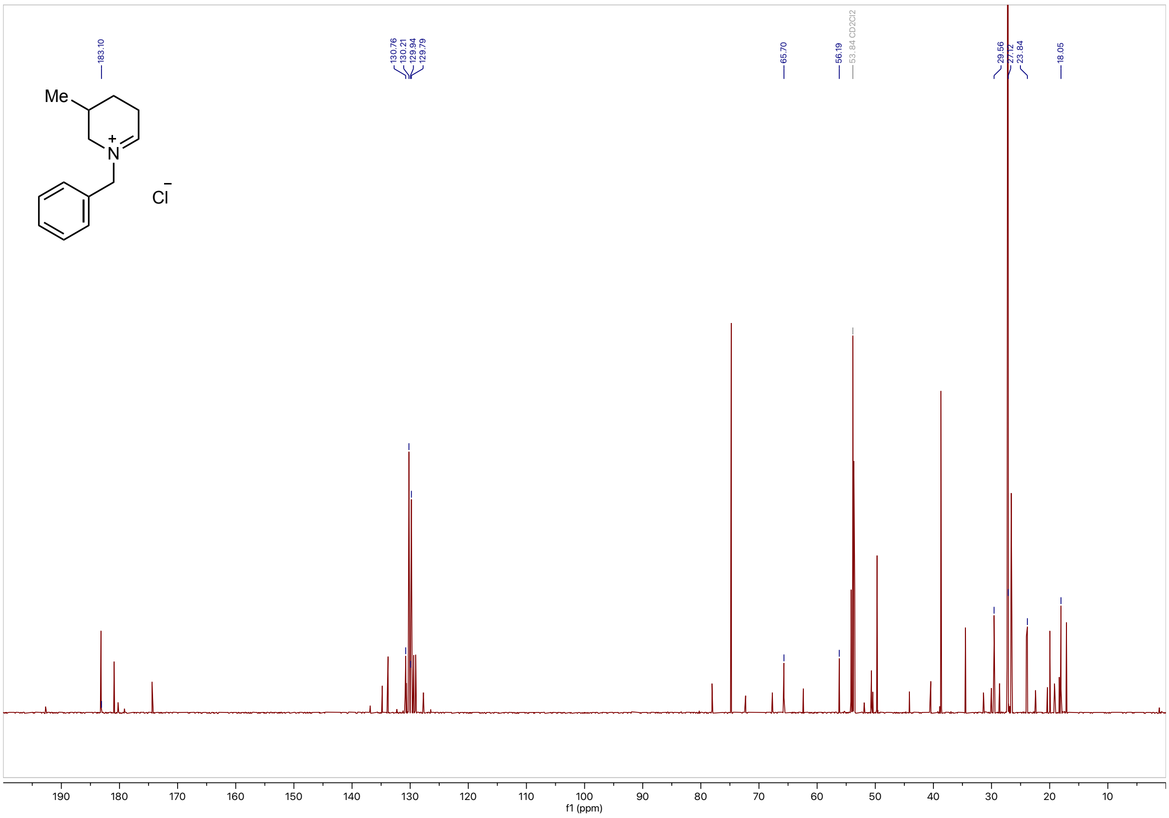

^{1}H NMR (700 MHz, CD_2Cl_2) of 1-benzyl-4-methyl-2,3,4,5-tetrahydropyridine-1-ium chloride (**3h**):

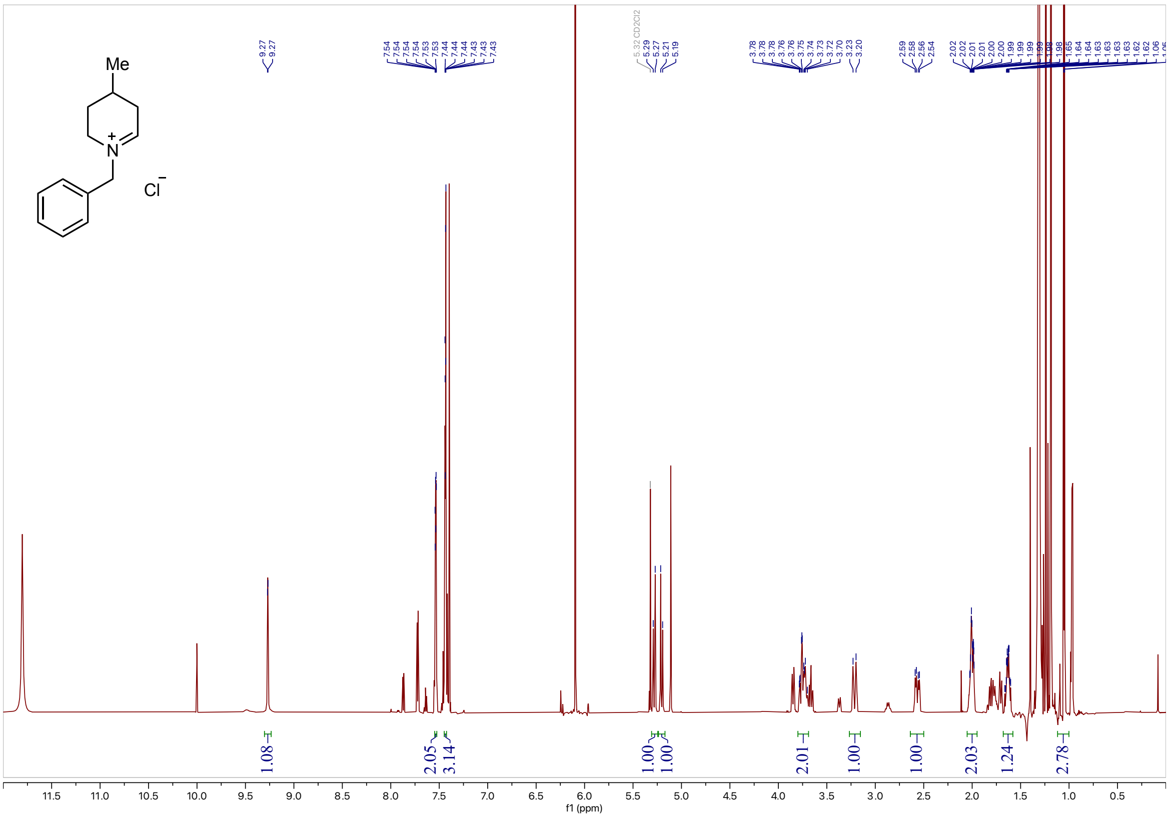

^{13}C{H} NMR (176 MHz, CD_2Cl_2) of 1-benzyl-4-methyl-2,3,4,5-tetrahydropyridine-1-ium chloride (**3h**):

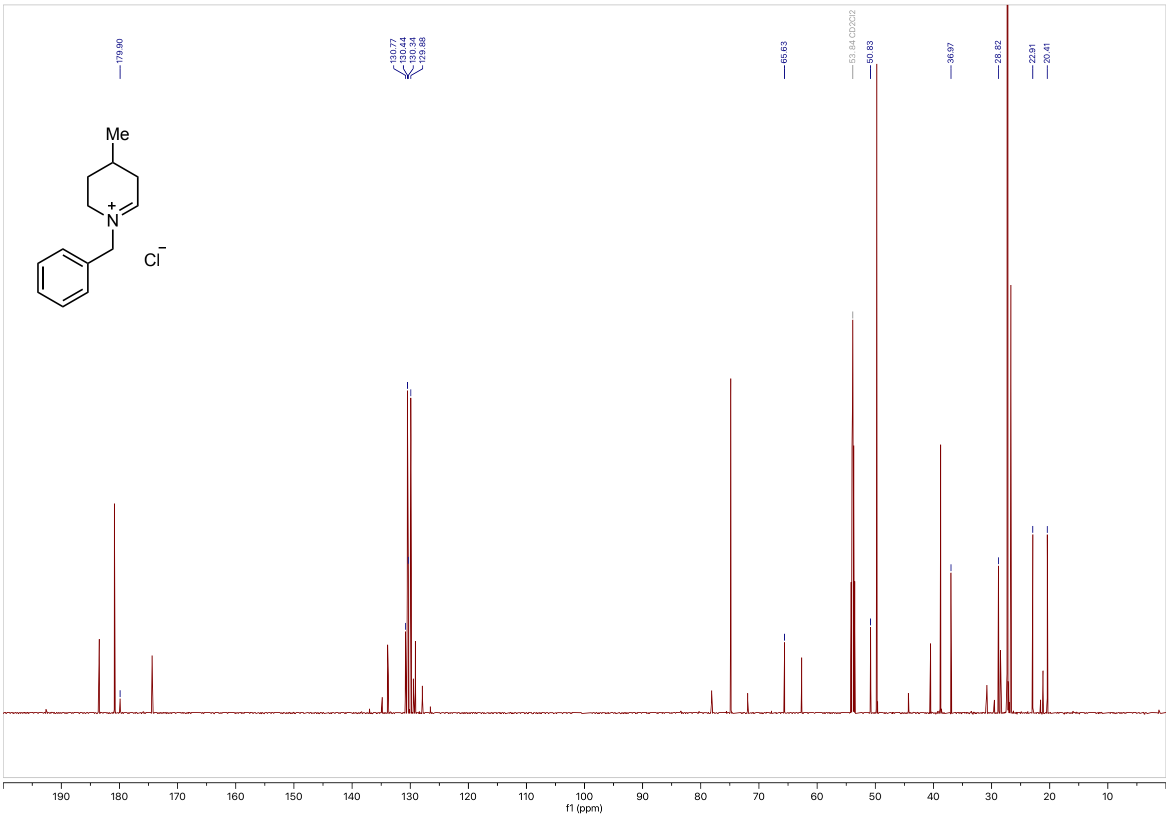

^{1}H NMR (700 MHz, CD_2Cl_2) of 1-benzyl-3-4-dihydro-2H-pyrrol-1-ium chloride (**3k**):

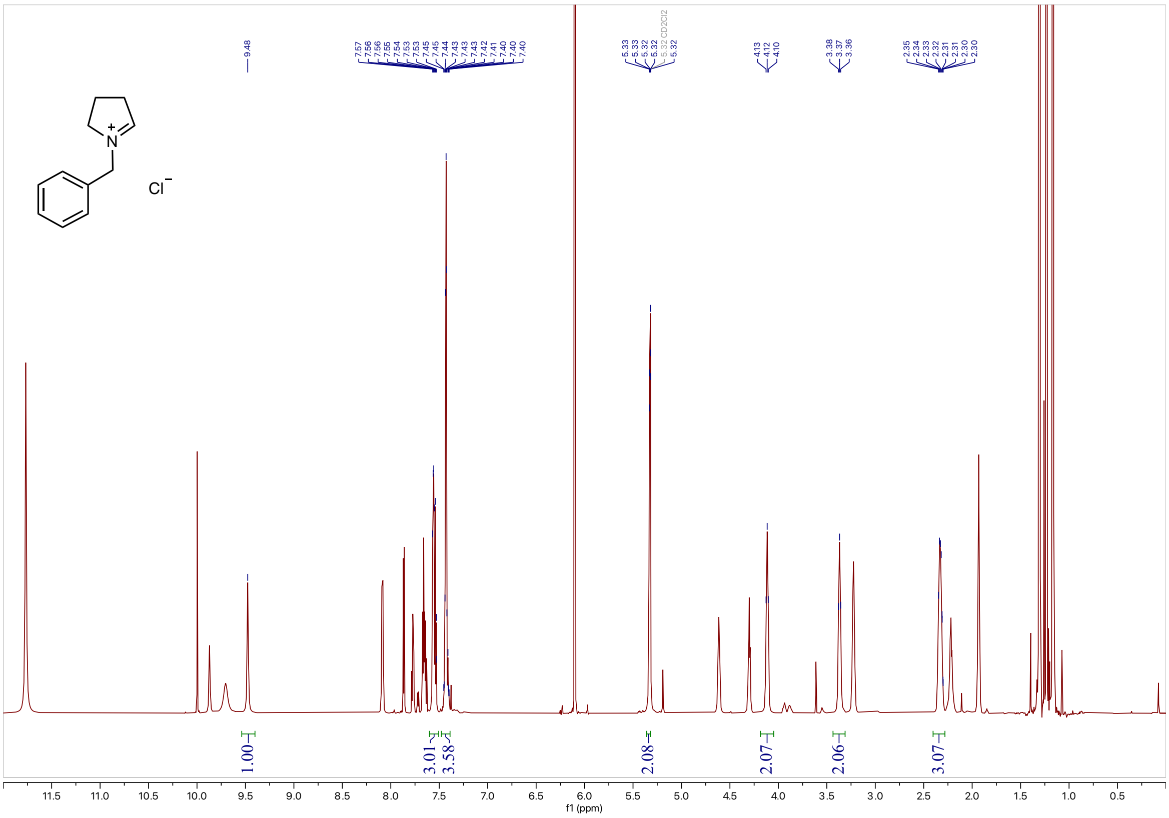

^{13}C{H} NMR (176 MHz, CD_2Cl_2) of 1-benzyl-3-4-dihydro-2H-pyrrol-1-ium chloride (**3k**):

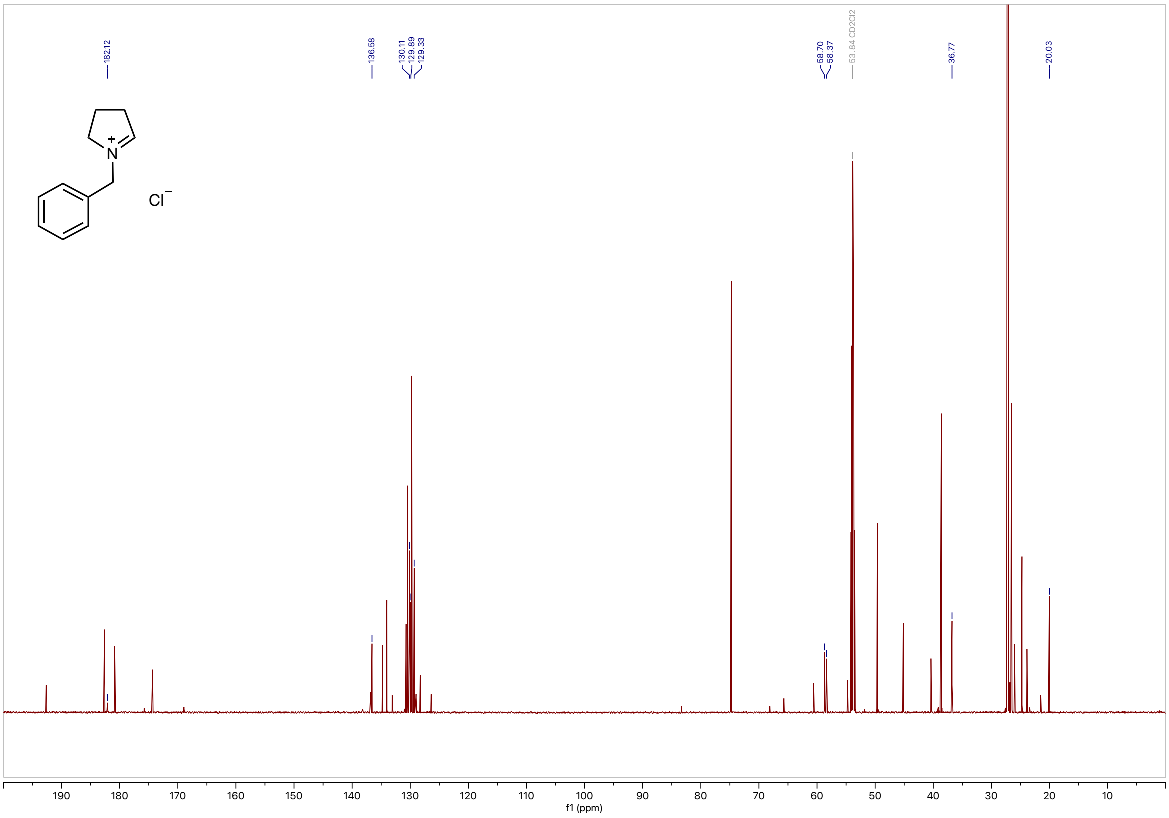

^{1}H NMR (700 MHz, CD_2Cl_2) of 1-benzylidenepyrrolidin-1-ium chloride (**4k**):

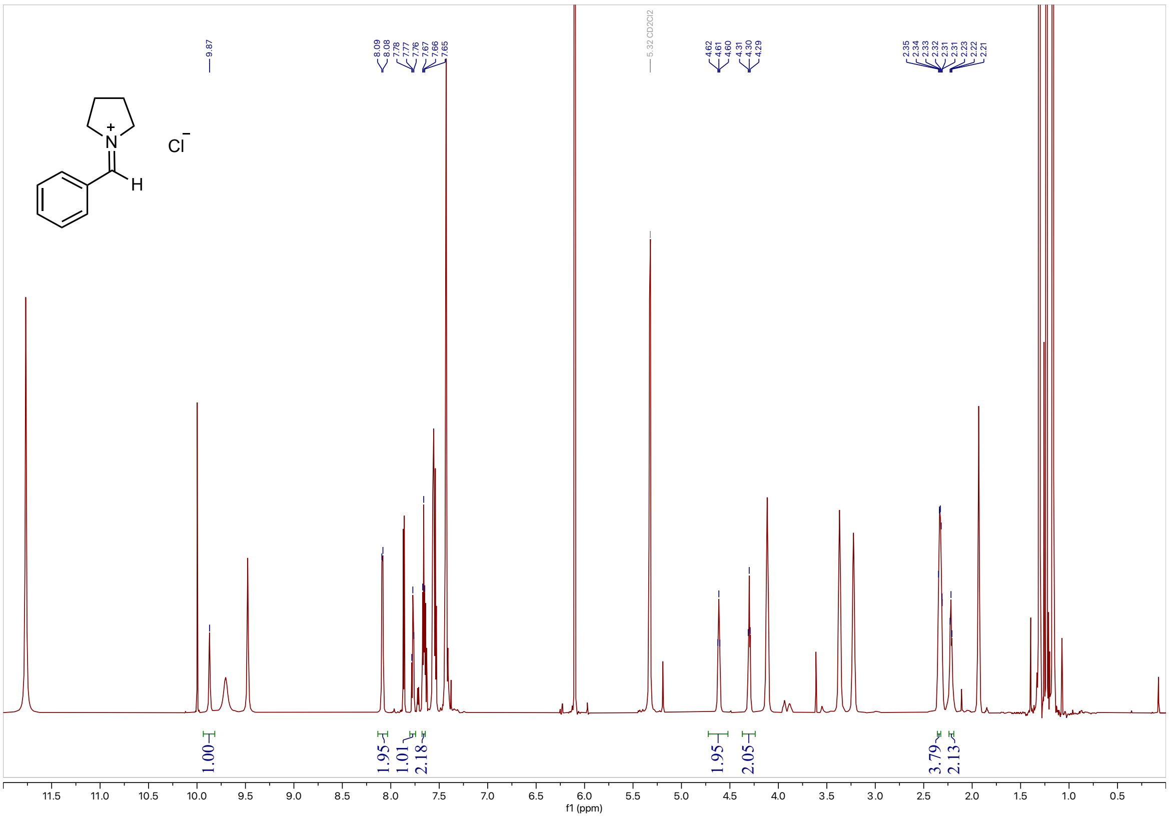

$^{13}C\{H\}$ NMR (176 MHz, CD_2Cl_2) of 1-benzylidenepyrrolidin-1-ium chloride (**4k**):

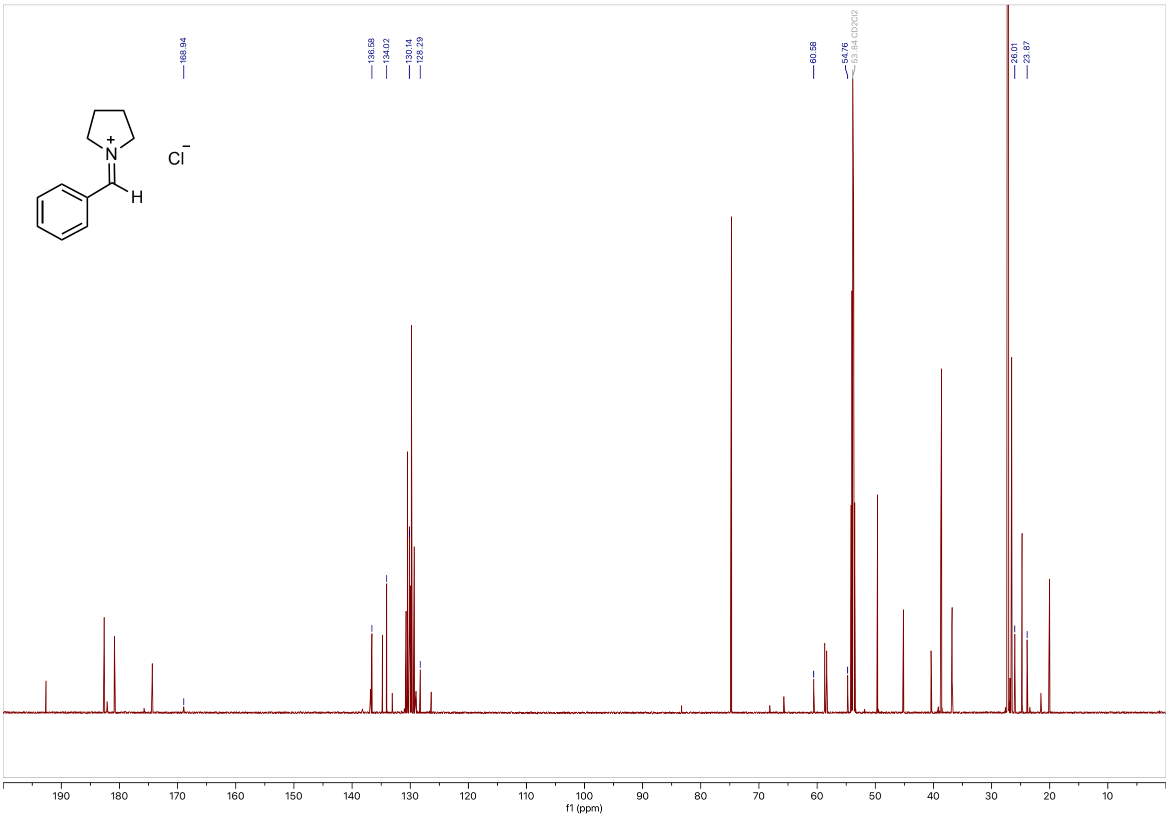

^{1}H NMR (400 MHz, CD_2Cl_2) of 1-benzyl-3,4,5,6-tetrahydrp-*2H*-azepin-1-ium chloride (**3l**):

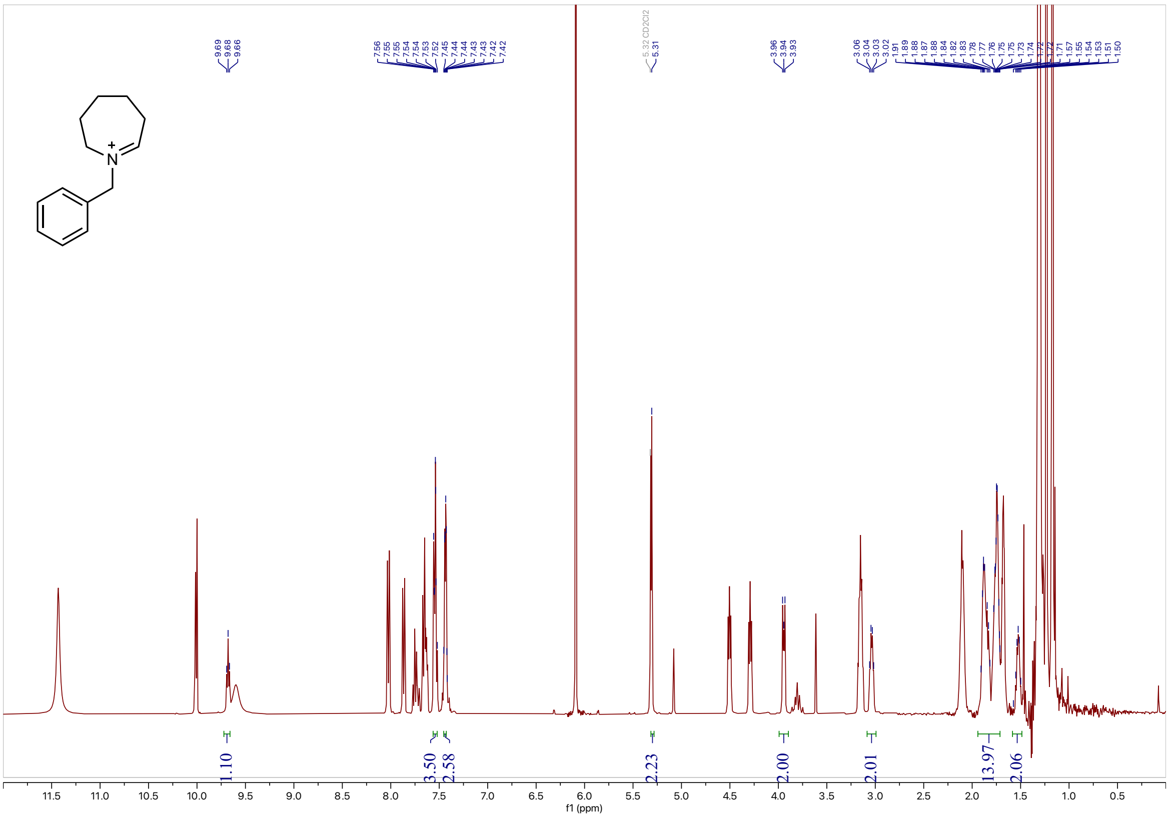

^{13}C{H} NMR (101 MHz, CD_2Cl_2) of 1-benzyl-3,4,5,6-tetrahydrp-*2H*-azepin-1-ium chloride (**3l**):

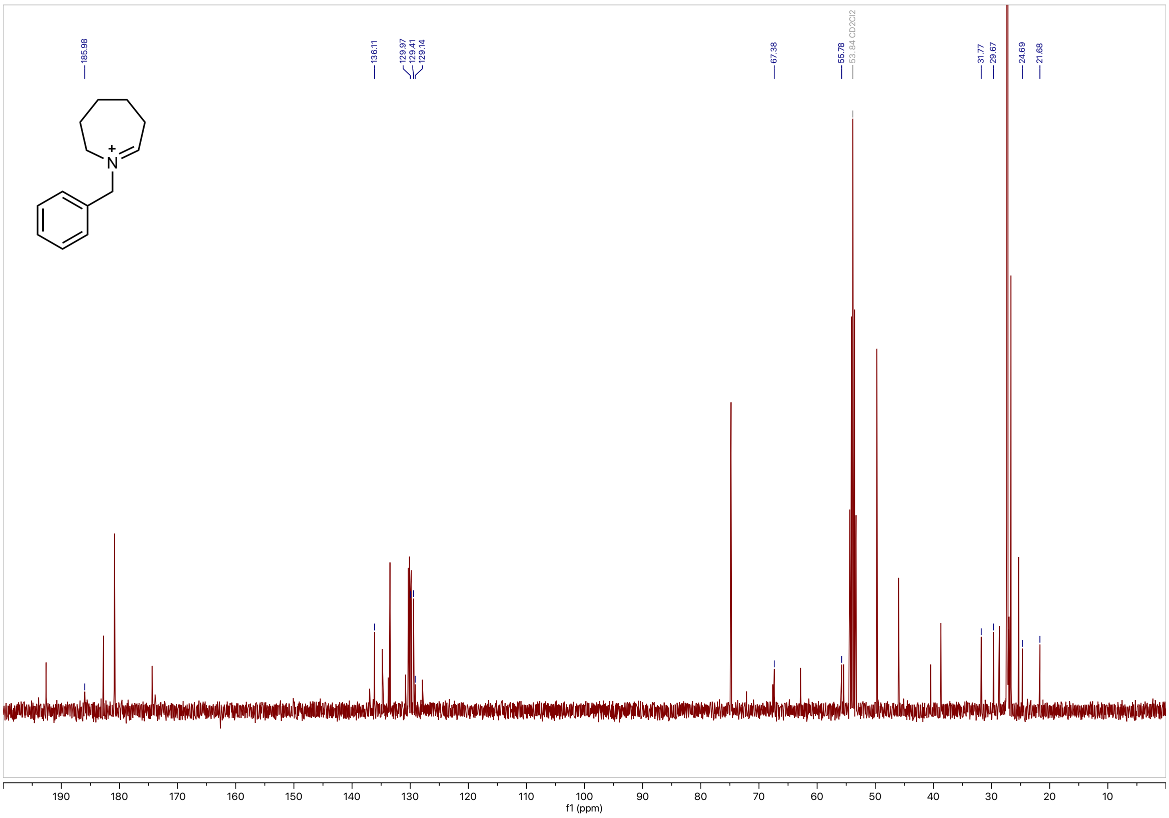

^{1}H NMR (400 MHz, CD_2Cl_2) of 1-benzylideneazepan-1-ium chloride (**4l**):

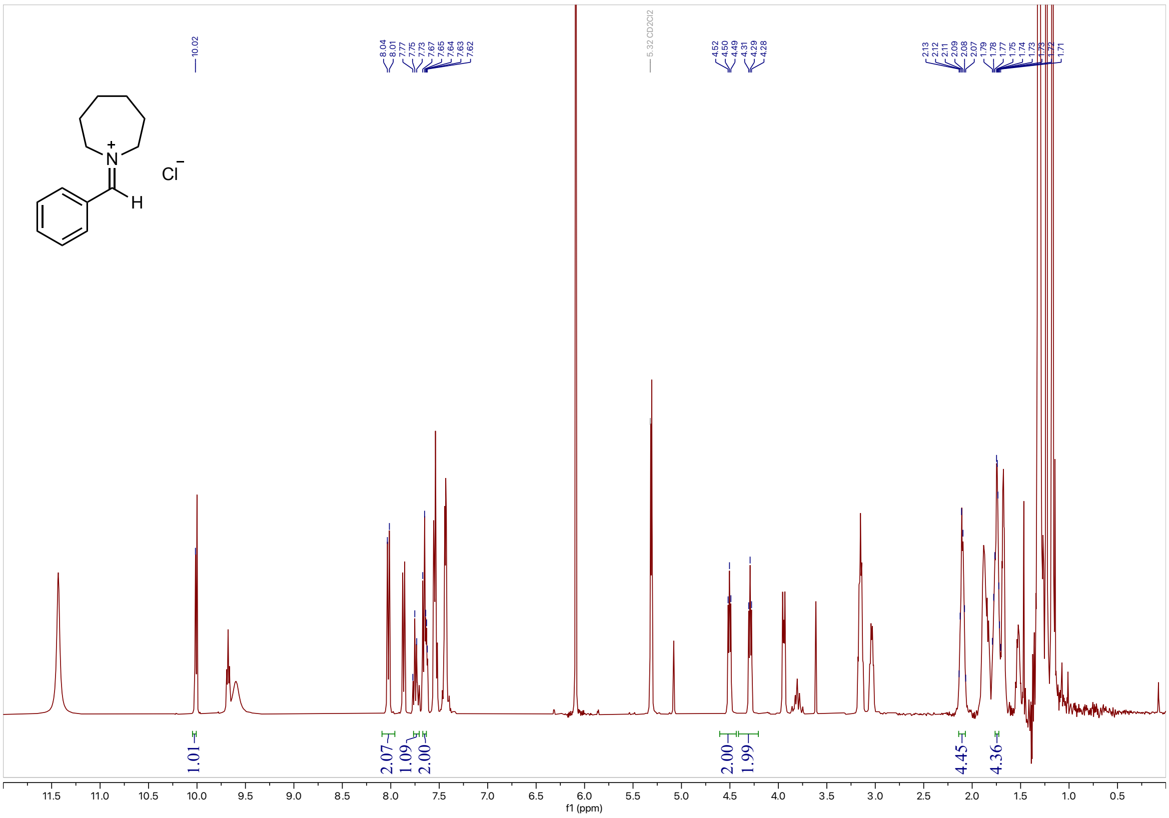

$^{13}C\{H\}$ NMR (101 MHz, CD_2Cl_2) of 1-benzylideneazepan-1-ium chloride (**4l**):

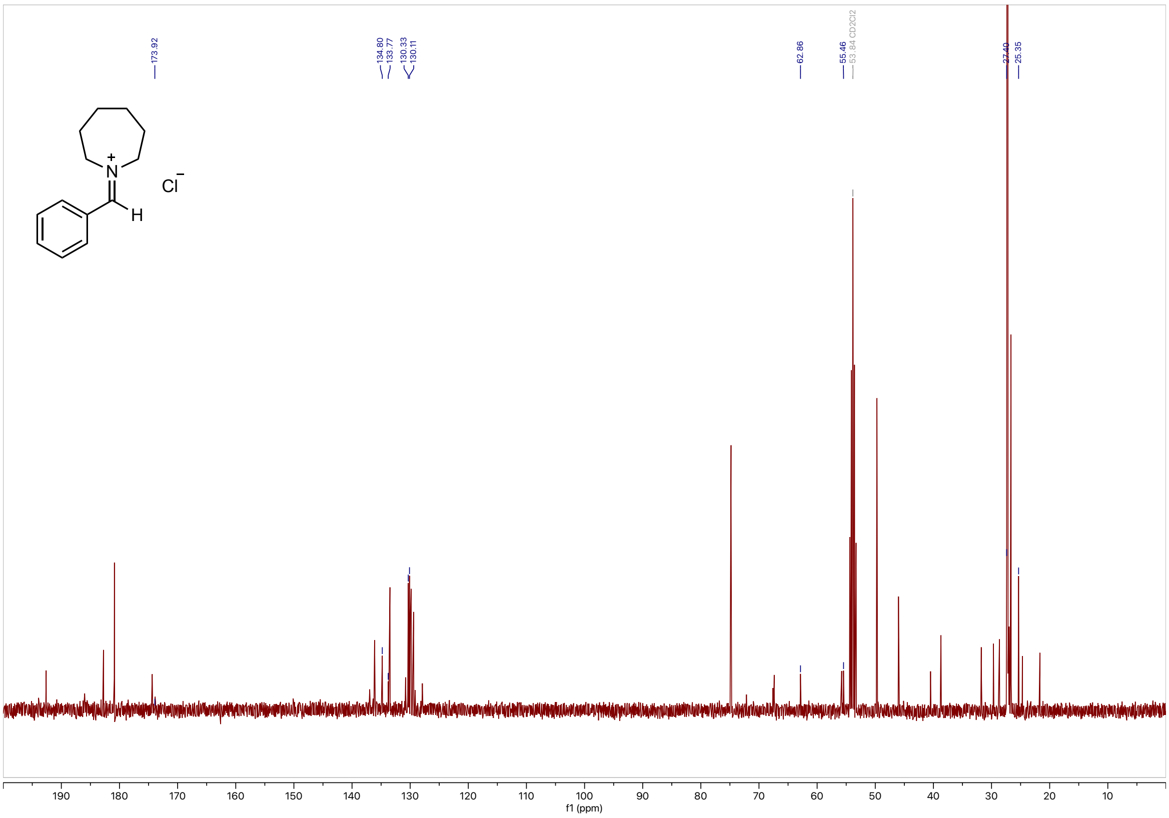

^{1}H NMR (500 MHz, CD_2Cl_2) of 2-benzyl-3,4-dihydroisoqinolin-2-ium chloride (**3m**):

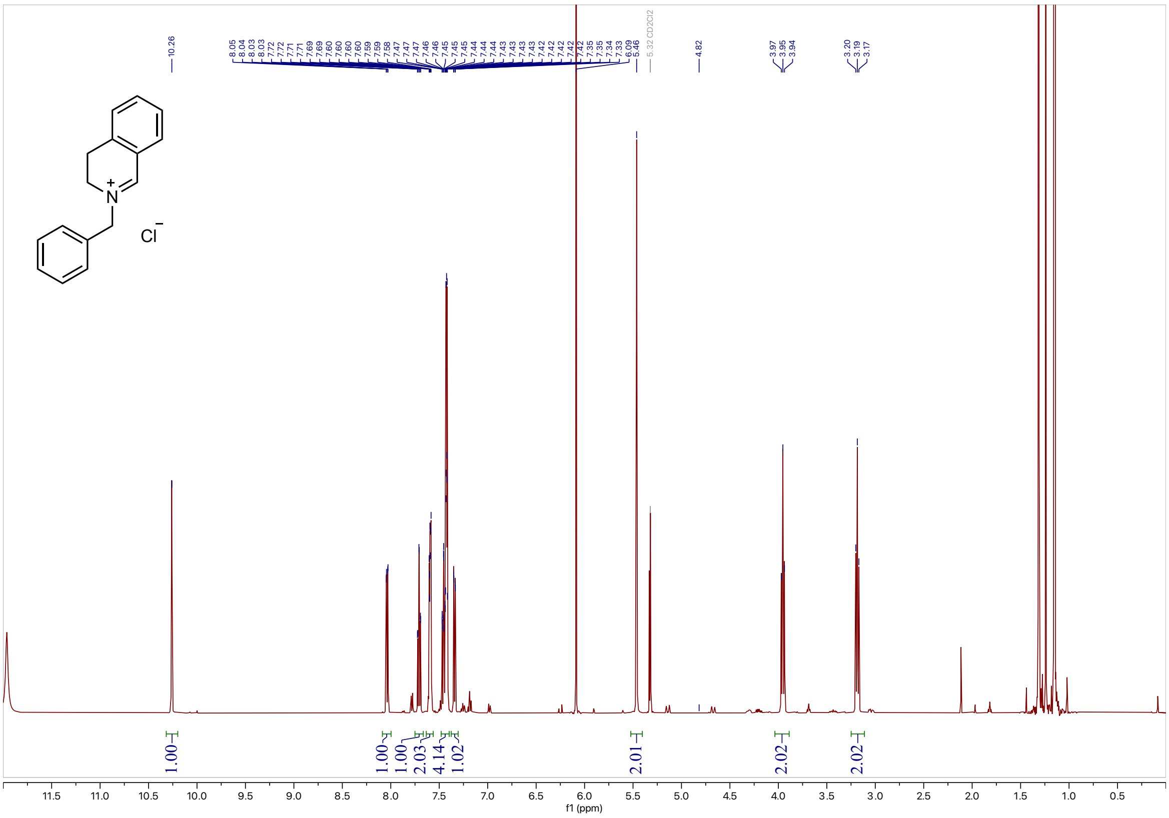

^{13}C{H} NMR (126 MHz, CD_2Cl_2) of 2-benzyl-3,4-dihydroisoqinolin-2-ium chloride (**3m**):

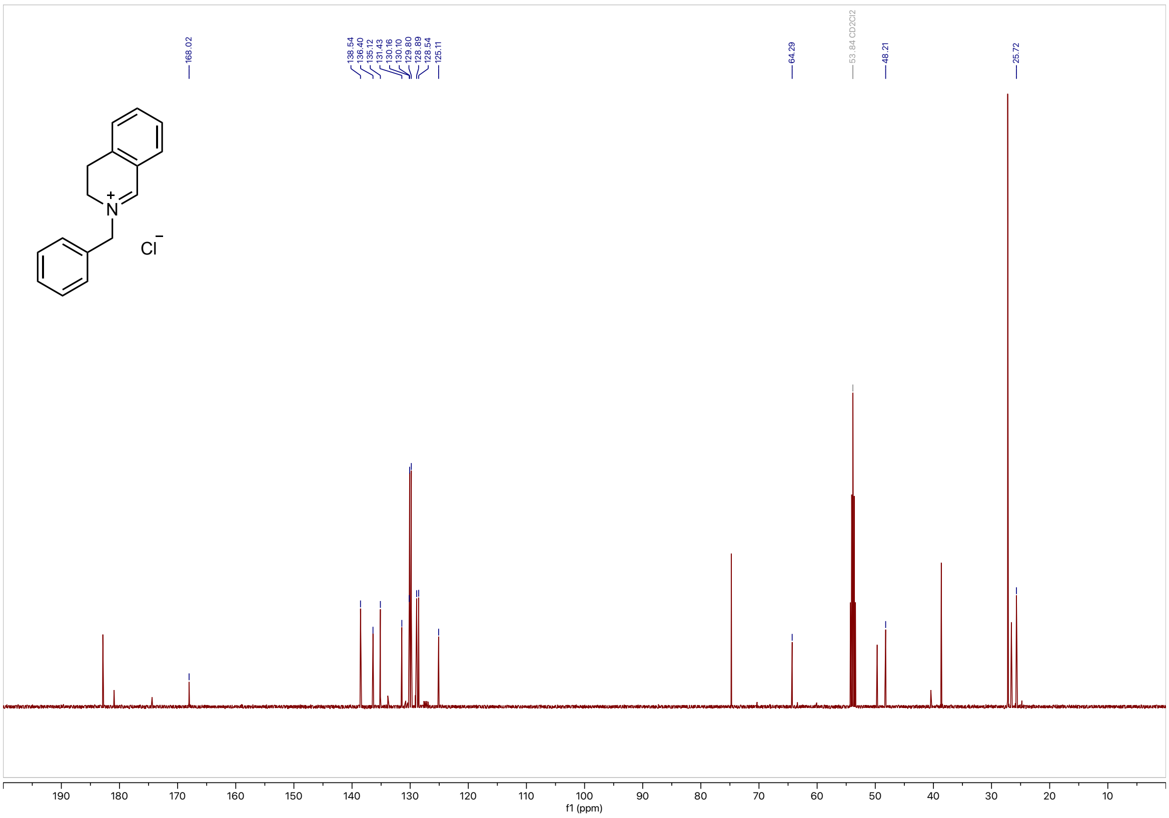

^{1}H NMR (500 MHz, CD_2Cl_2) of 1-(4-phenylbenzyl)-2,3,4,5-tetrahydropyridine-1-ium chloride (**3n**):

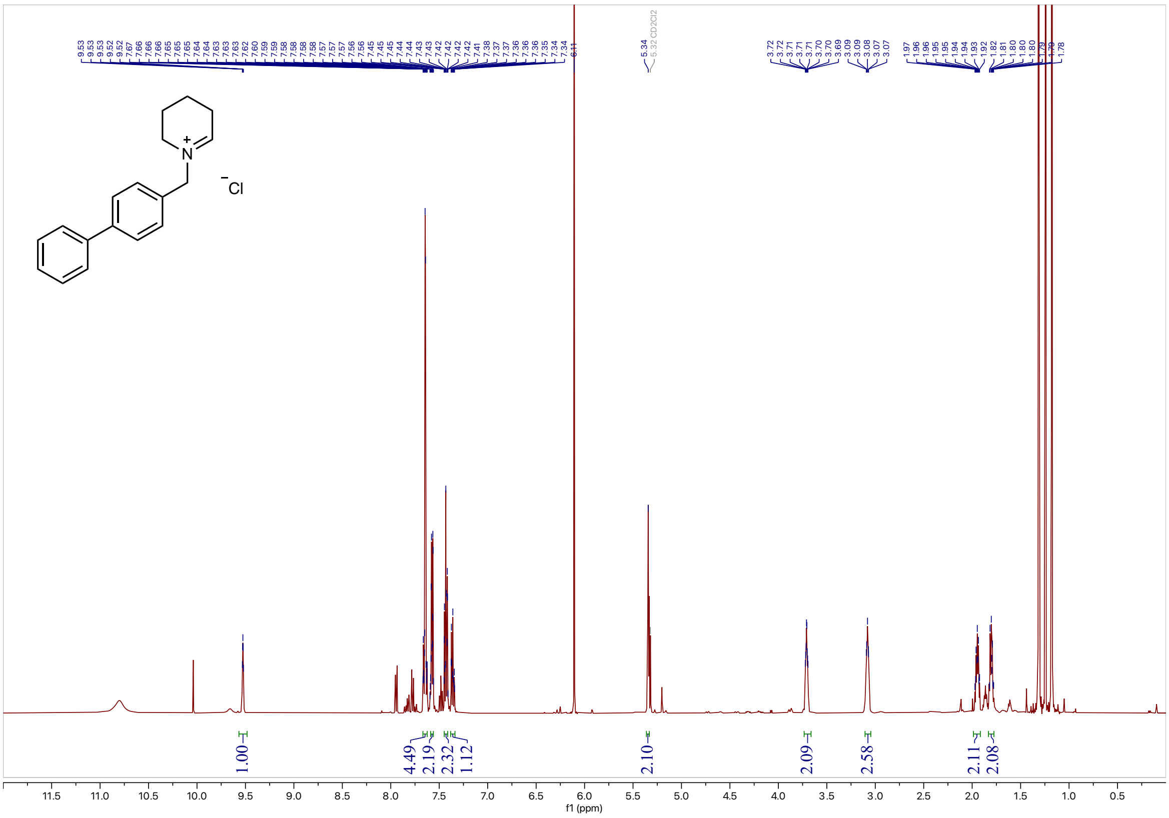

$^{13}C\{H\}$ NMR (126 MHz, CD_2Cl_2) of 1-(4-phenylbenzyl)-2,3,4,5-tetrahydropyridine-1-ium chloride (**3n**):

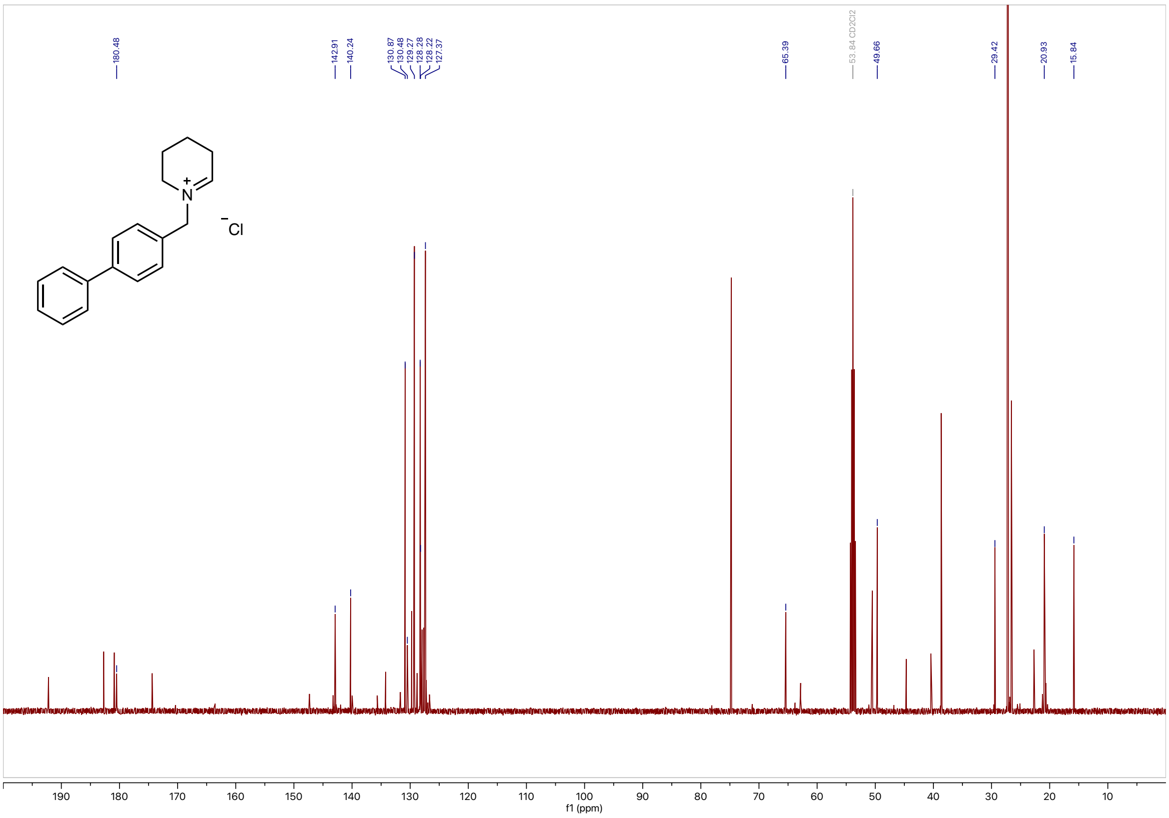

^{1}H NMR (700 MHz, CD_2Cl_2) of 1-(4-phenylbenzyl)-2-methyl-2,3,4,5-tetrahydropyridin-1-ium chloride (**3o**):

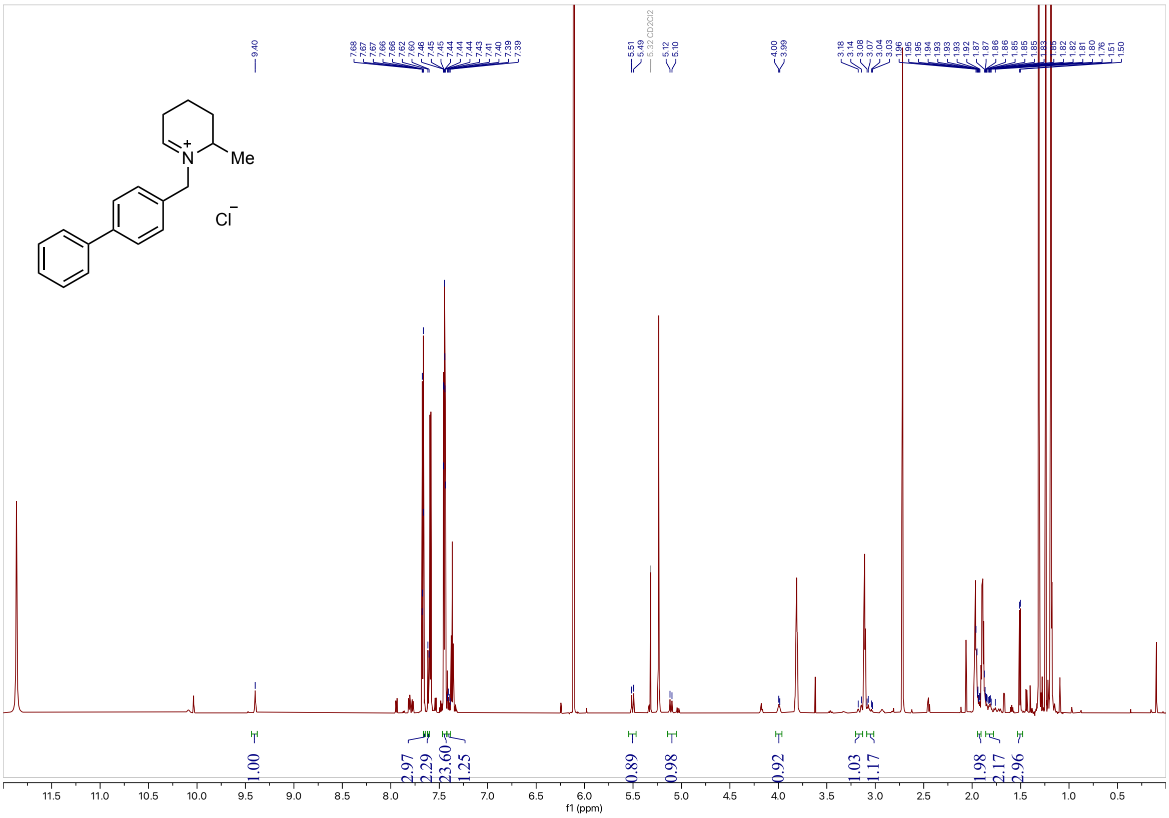

^{13}C{H} NMR (176 MHz, CD_2Cl_2) of 1-(4-phenylbenzyl)-2-methyl-2,3,4,5-tetrahydropyridin-1-ium chloride (**3o**):

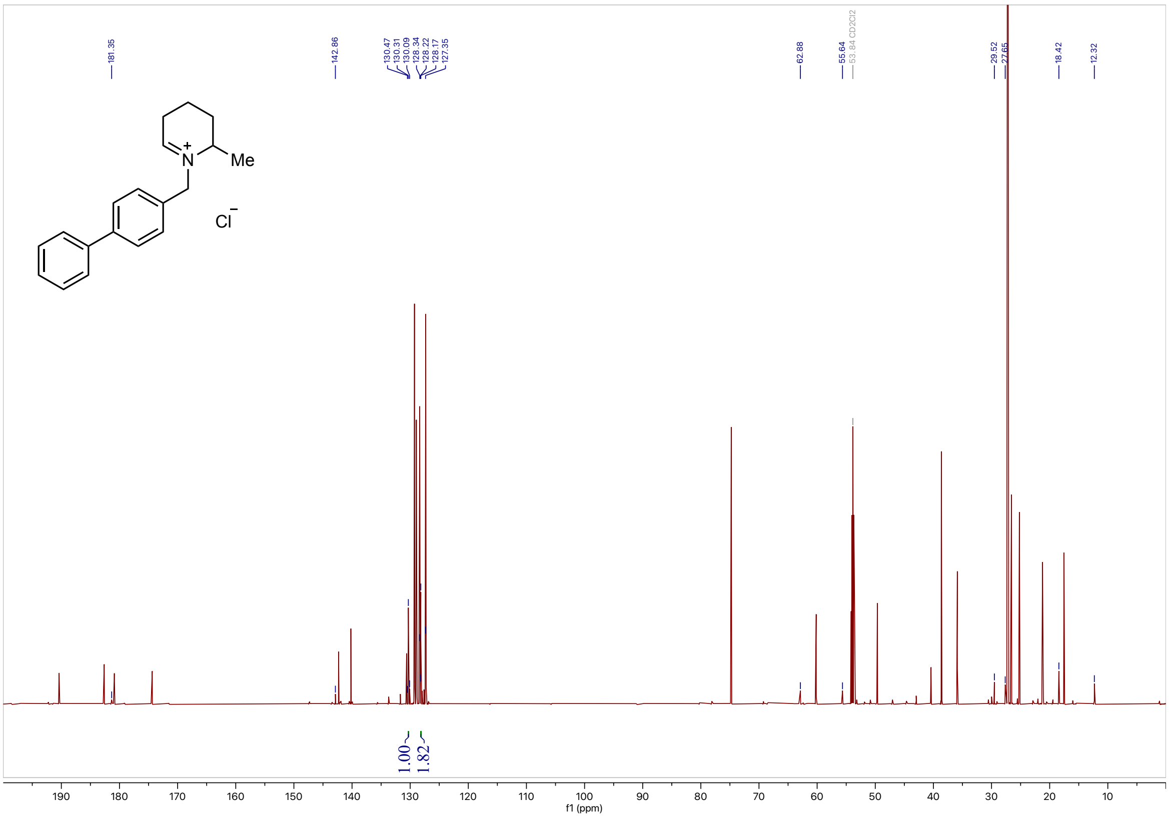

^{1}H NMR (700 MHz, CD_2Cl_2) of 1-(4-phenylbenzyl)-6-methyl-2,3,4,5-tetrahydropyridin-1-ium chloride (**3o'**):

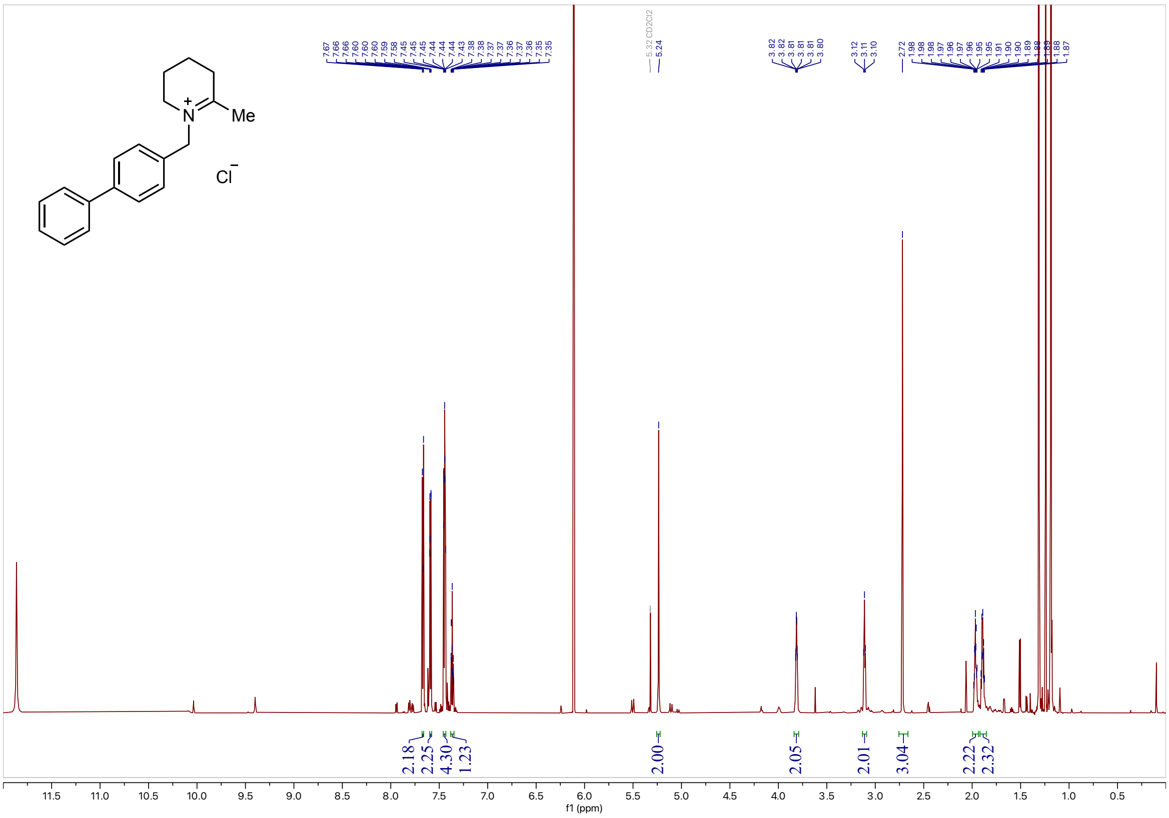

$^{13}C\{H\}$ NMR (176 MHz, CD_2Cl_2) of 1-(4-phenylbenzyl)-6-methyl-2,3,4,5-tetrahydropyridin-1-ium chloride (**3o'**):

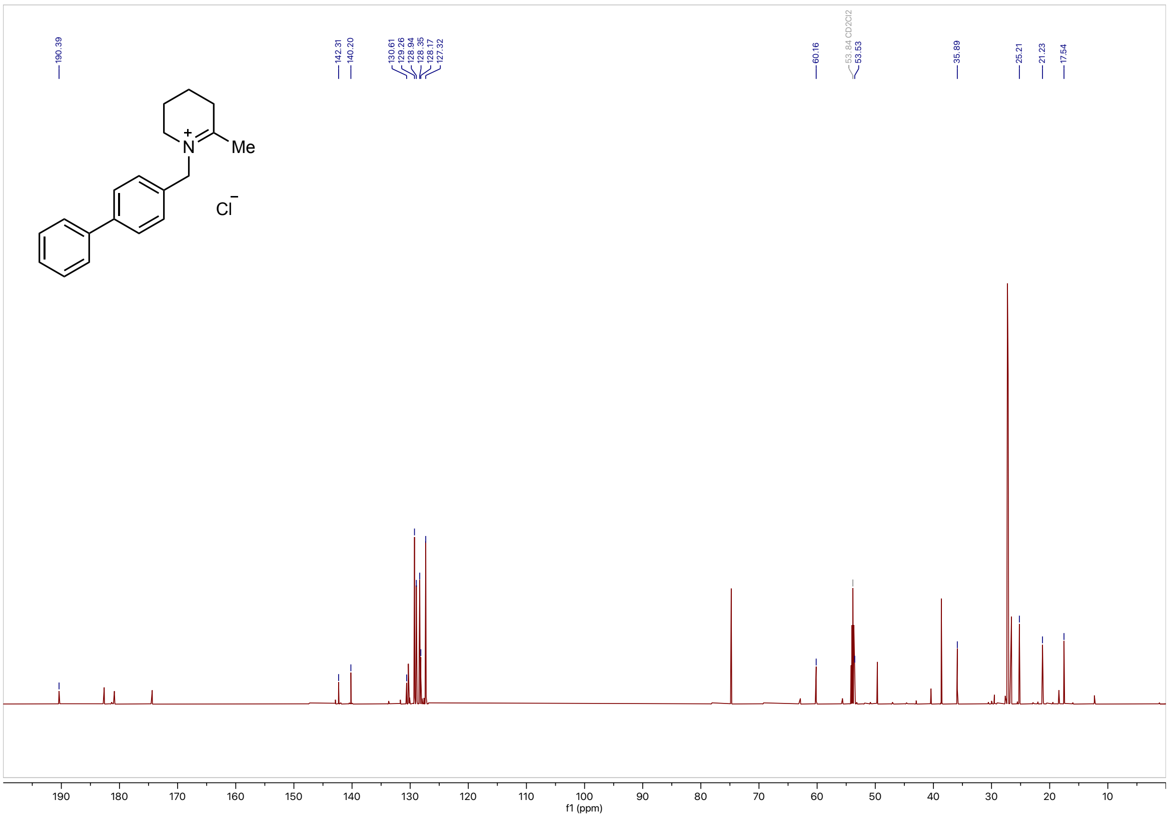

^{1}H NMR (400 MHz, $CDCl_3$) of 1-benzyl-2-isopropyl-piperidine (**6a**):

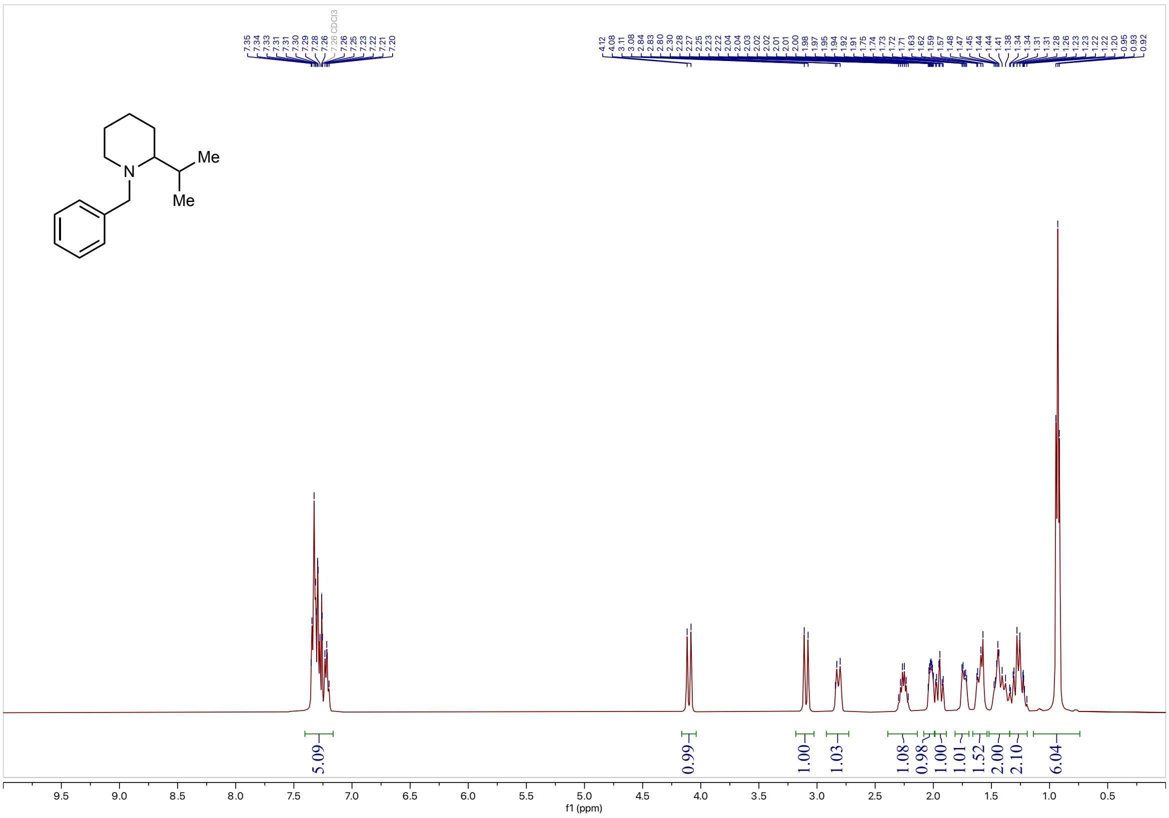

$^{13}C\{H\}$ NMR (126 MHz, $CDCl_3$) of 1-benzyl-2-isopropyl-piperidine (**6a**):

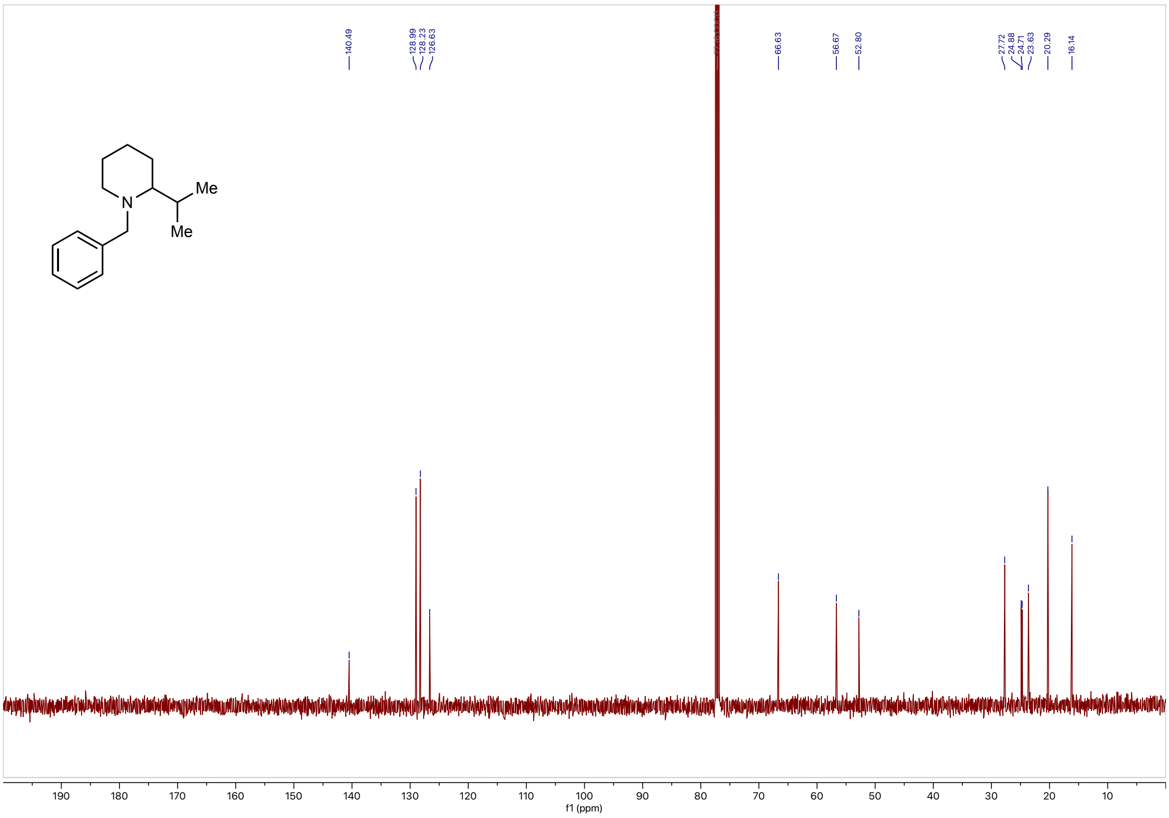

^{1}H NMR (400 MHz, $CDCl_3$) of 1-benzyl-2-isopropyl-piperidine (**6a**, 1 mmol):

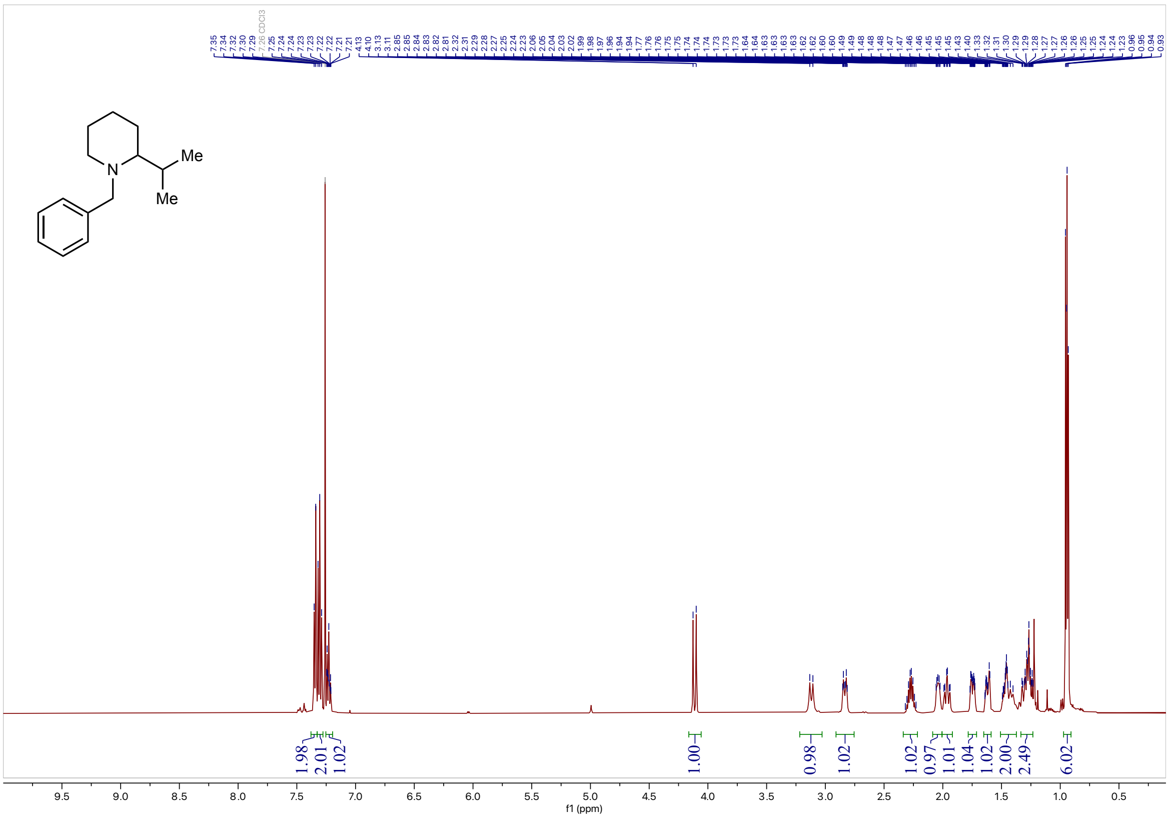

$^{13}C\{H\}$ NMR (126 MHz, $CDCl_3$) of 1-benzyl-2-isopropyl-piperidine (**6a**, 1 mmol):

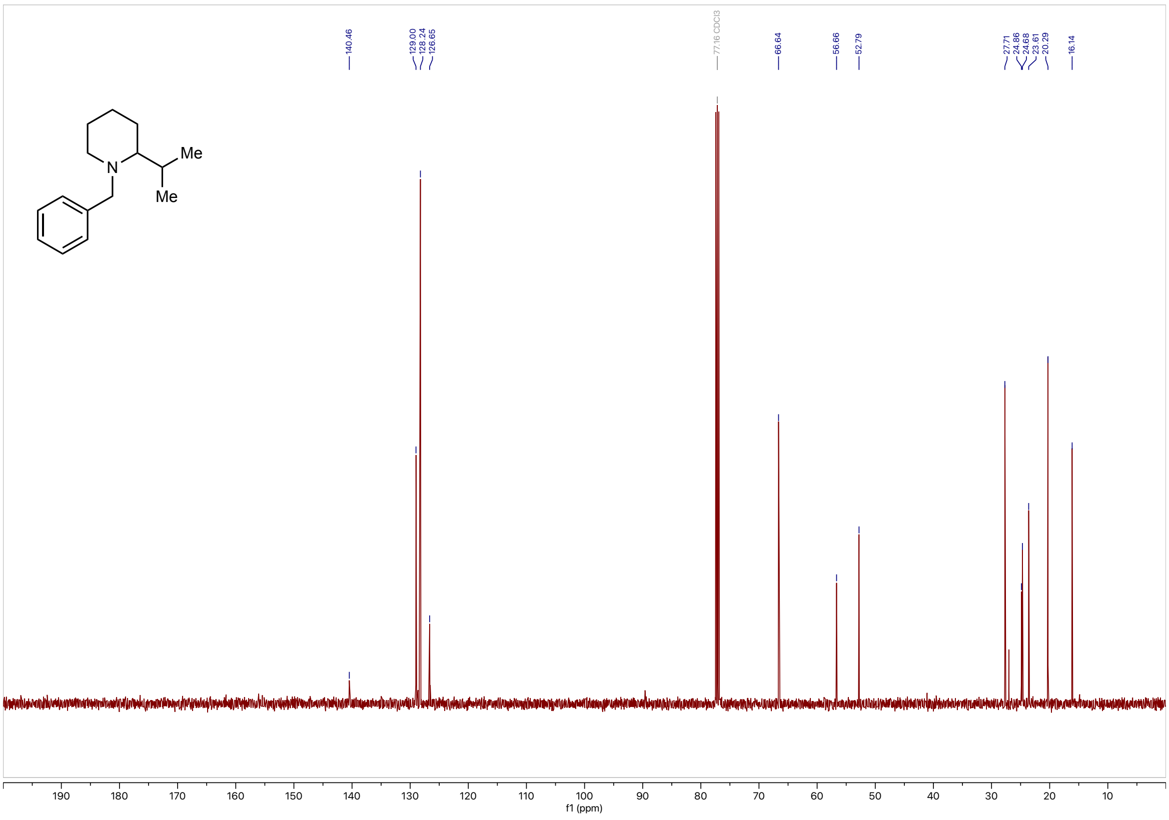

^{1}H NMR (500 MHz, $CDCl_3$) of 1-benzyl-2-ethyl-piperidine (**6b**):

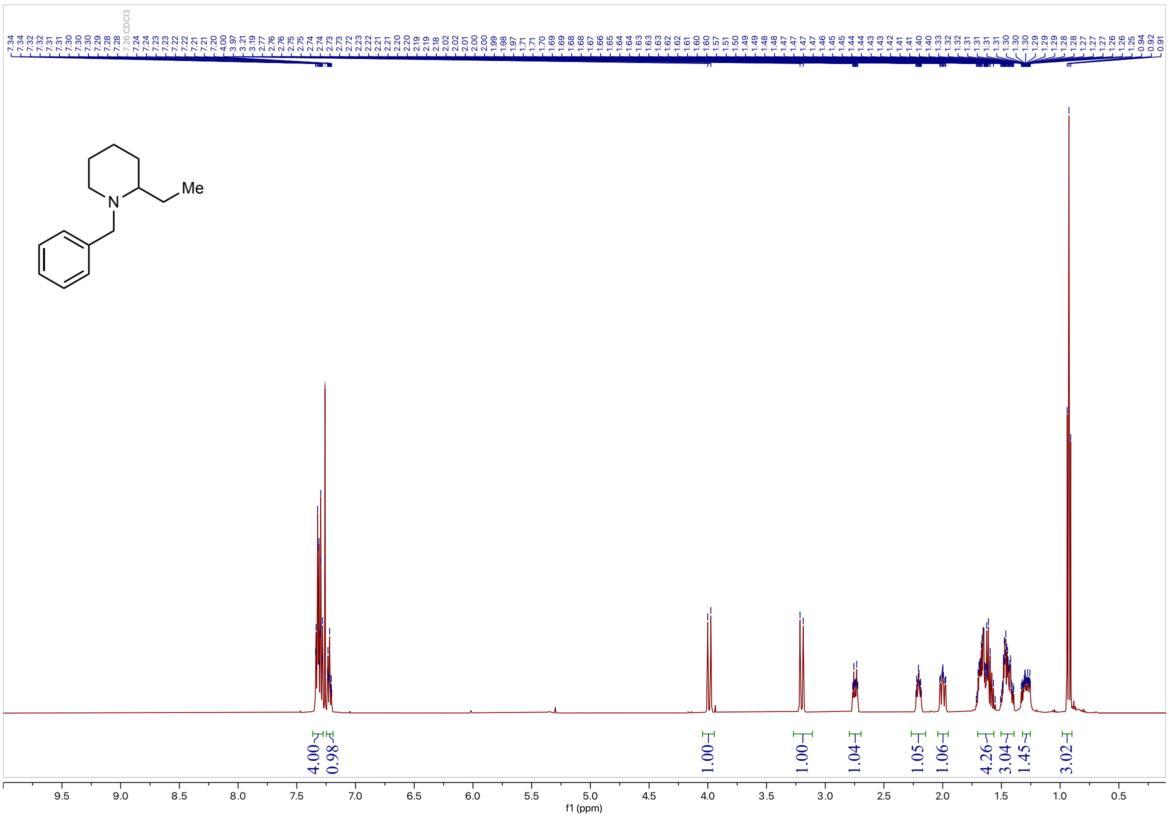

^{13}C{H} NMR (126 MHz, $CDCl_3$) of 1-benzyl-2-ethyl-piperidine (**6b**):

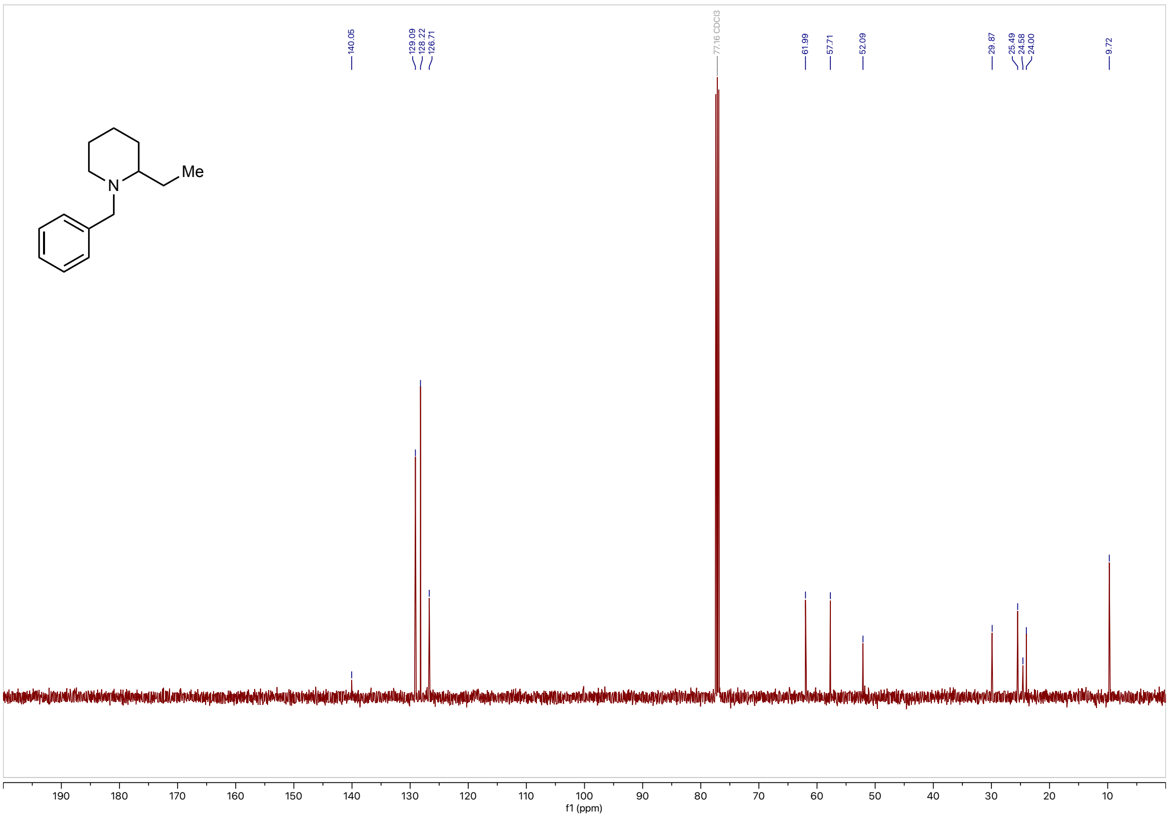

^{1}H NMR (400 MHz, $CDCl_3$) of 1-benzyl-2-(*tert*-butyl)-piperidine (**6c**):

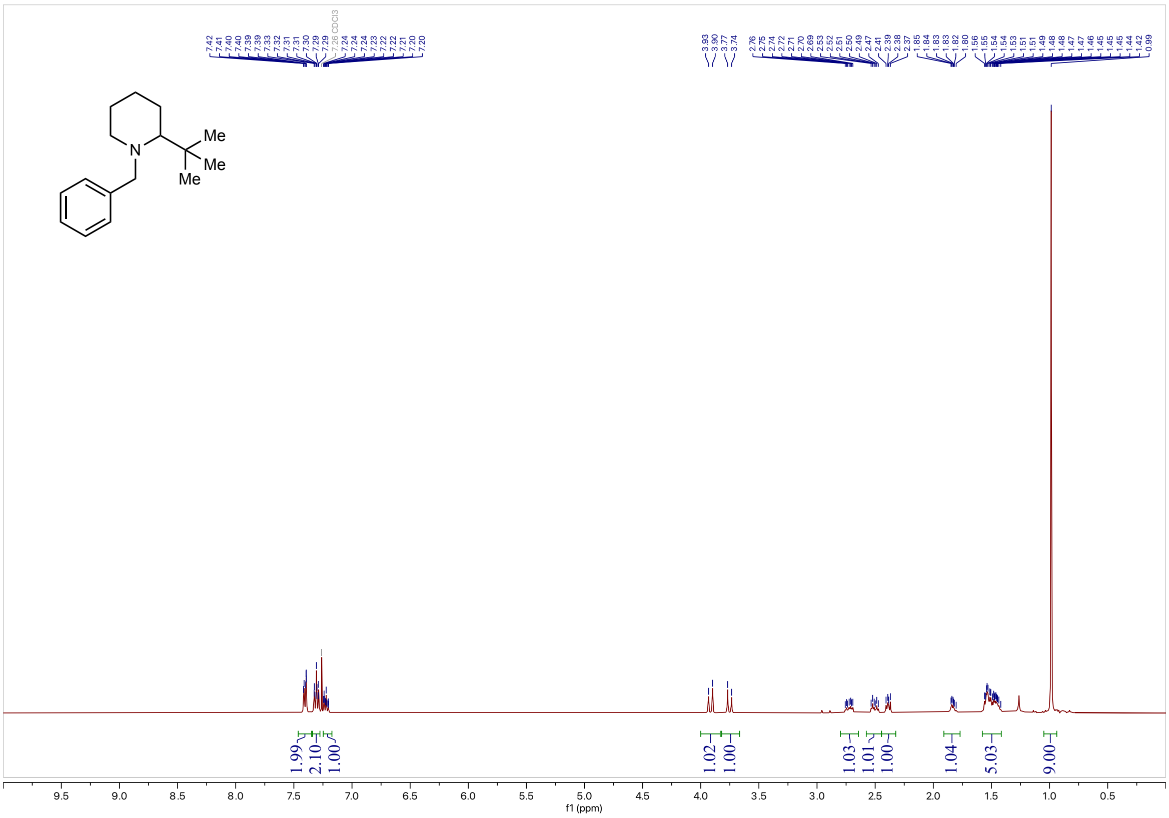

$^{13}C\{H\}$ NMR (101 MHz, $CDCl_3$) of 1-benzyl-2-(*tert*-butyl)-piperidine (**6c**):

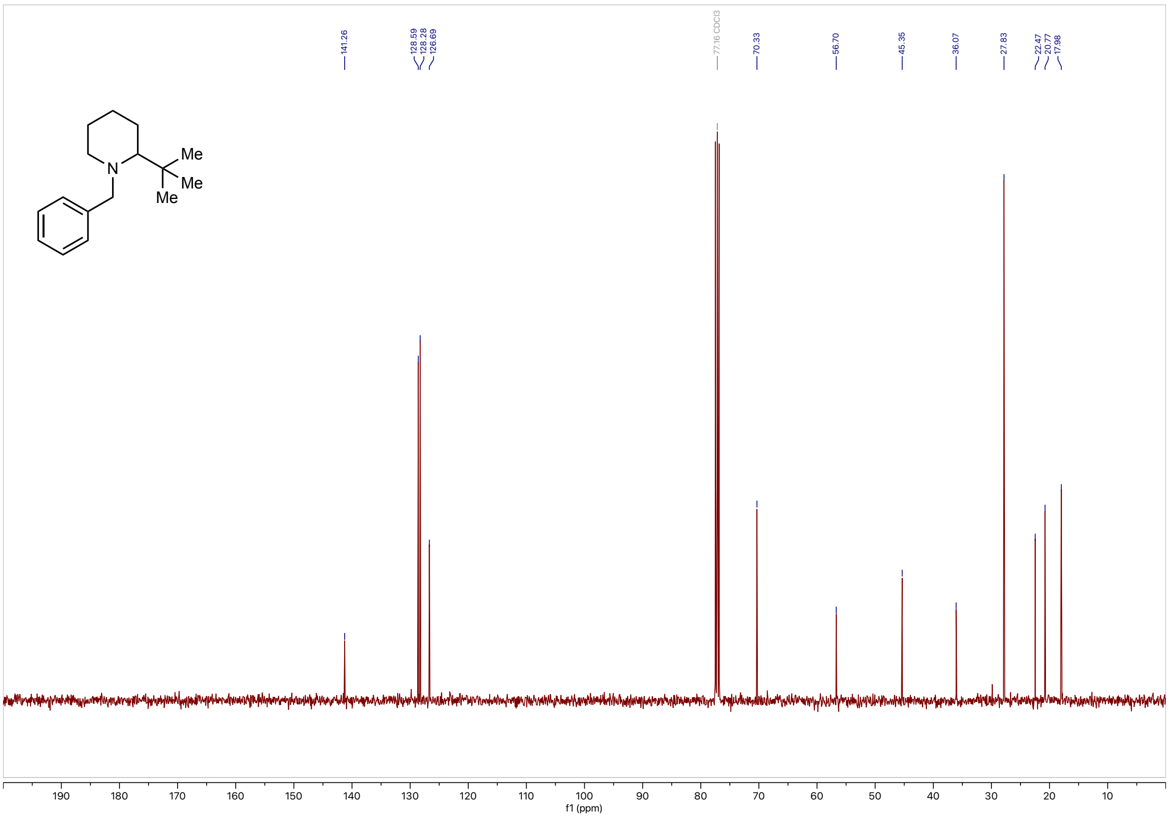

^{1}H NMR (400 MHz, $CDCl_3$) of 2-adamantan-1-yl-1-benzyl-piperidine (**6d**):

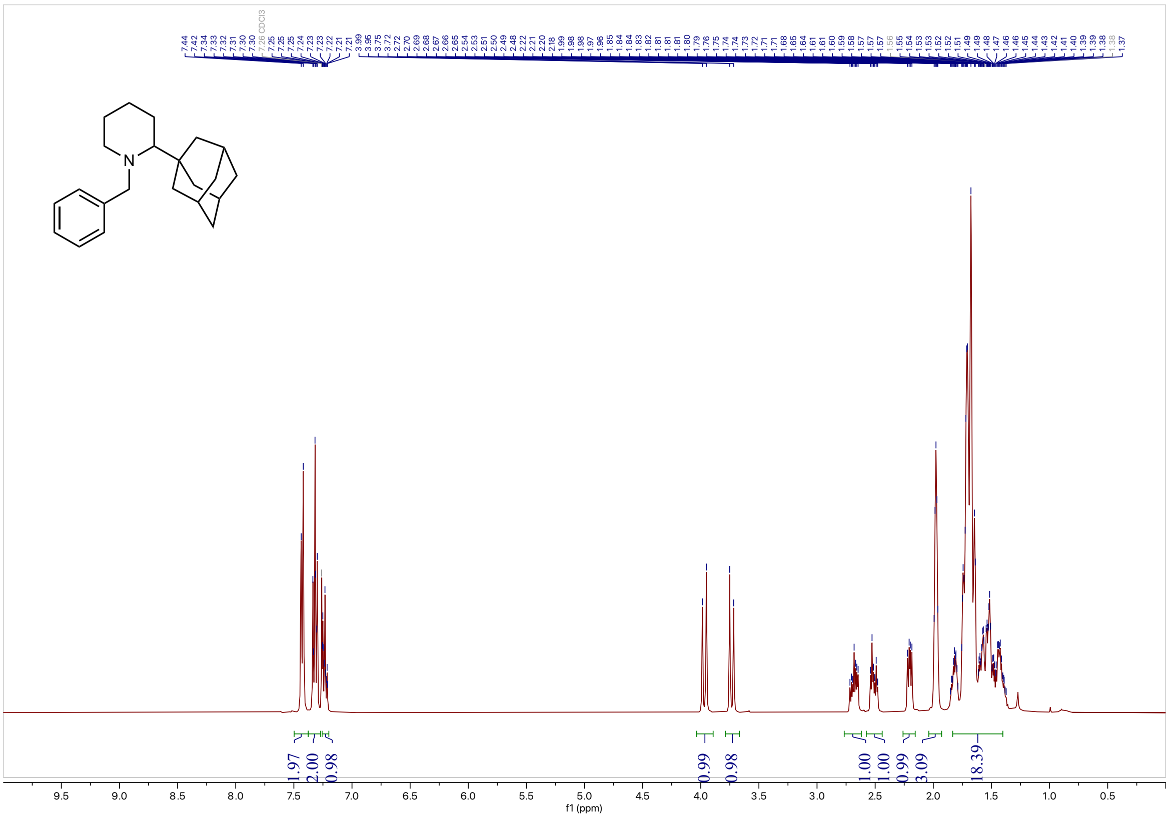

$^{13}C\{H\}$ NMR (126 MHz, $CDCl_3$) of 2-adamantan-1-yl-1-benzyl-piperidine (**6d**):

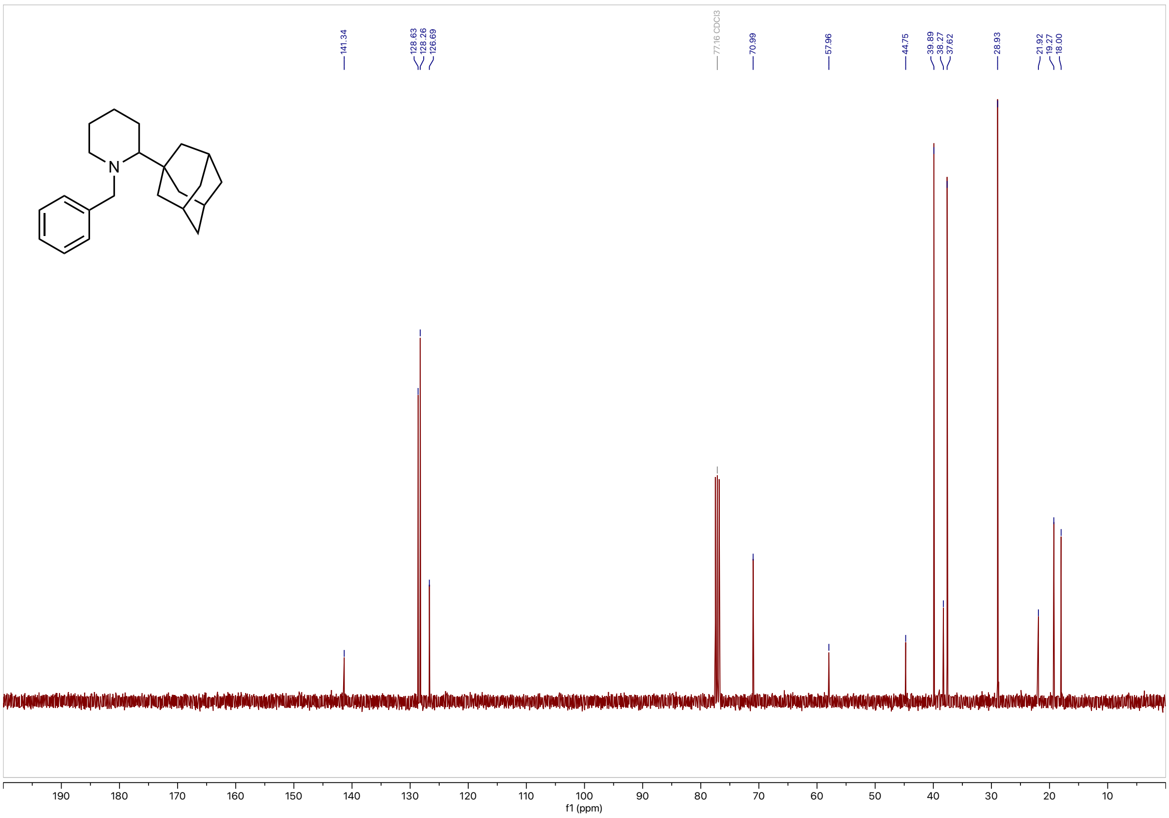

^{1}H NMR (500 MHz, $CDCl_3$) of *1-benzyl-2-cyclohexyl-piperidine* (**6e**):

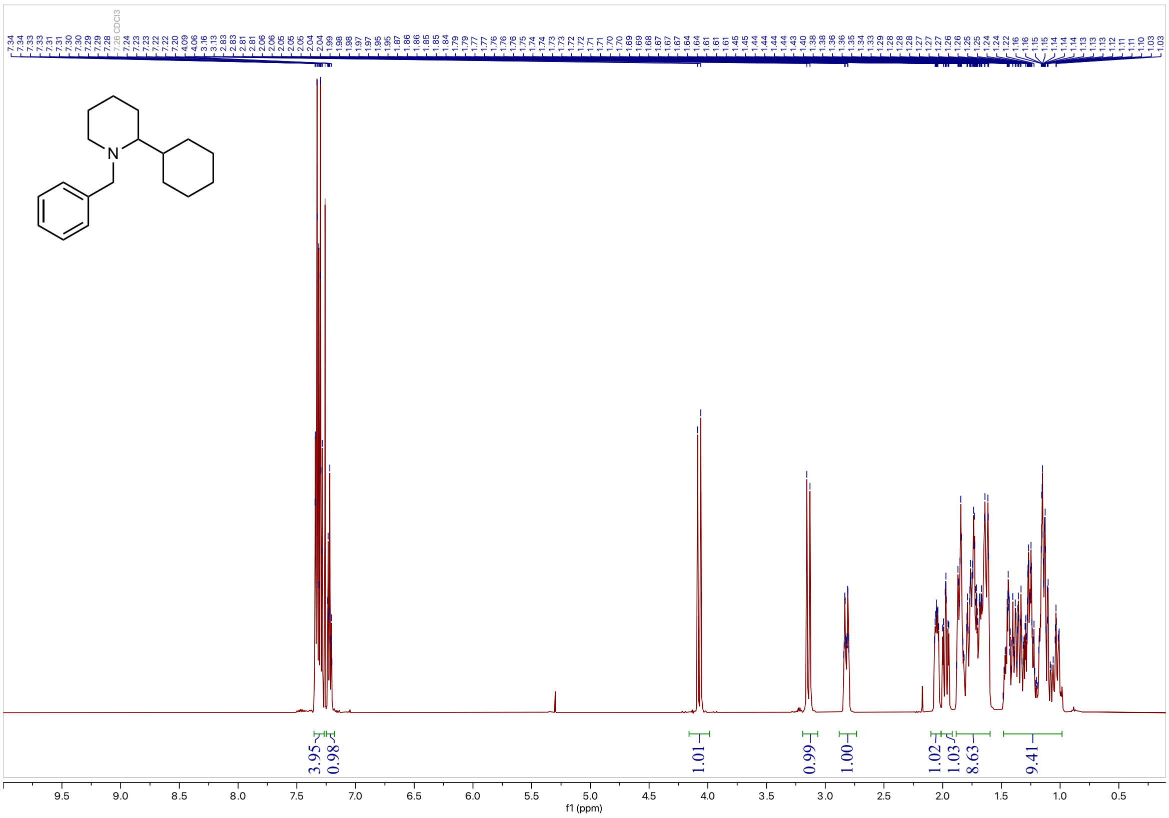

$^{13}C\{H\}$ NMR (126 MHz, $CDCl_3$) of *1-benzyl-2-cyclohexyl-piperidine* (**6e**):

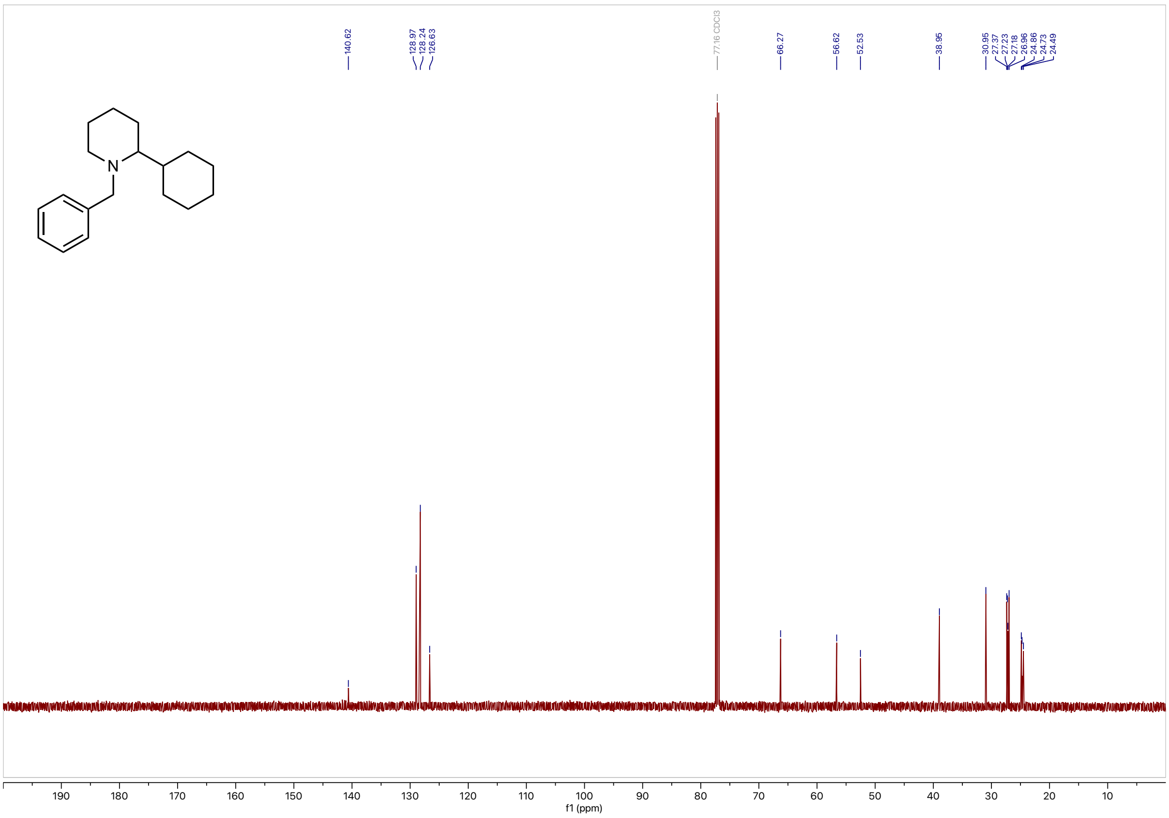

^{1}H NMR (500 MHz, $CDCl_3$) of (1-benzylpiperidin-2-yl) methyl pivalate (**6f**):

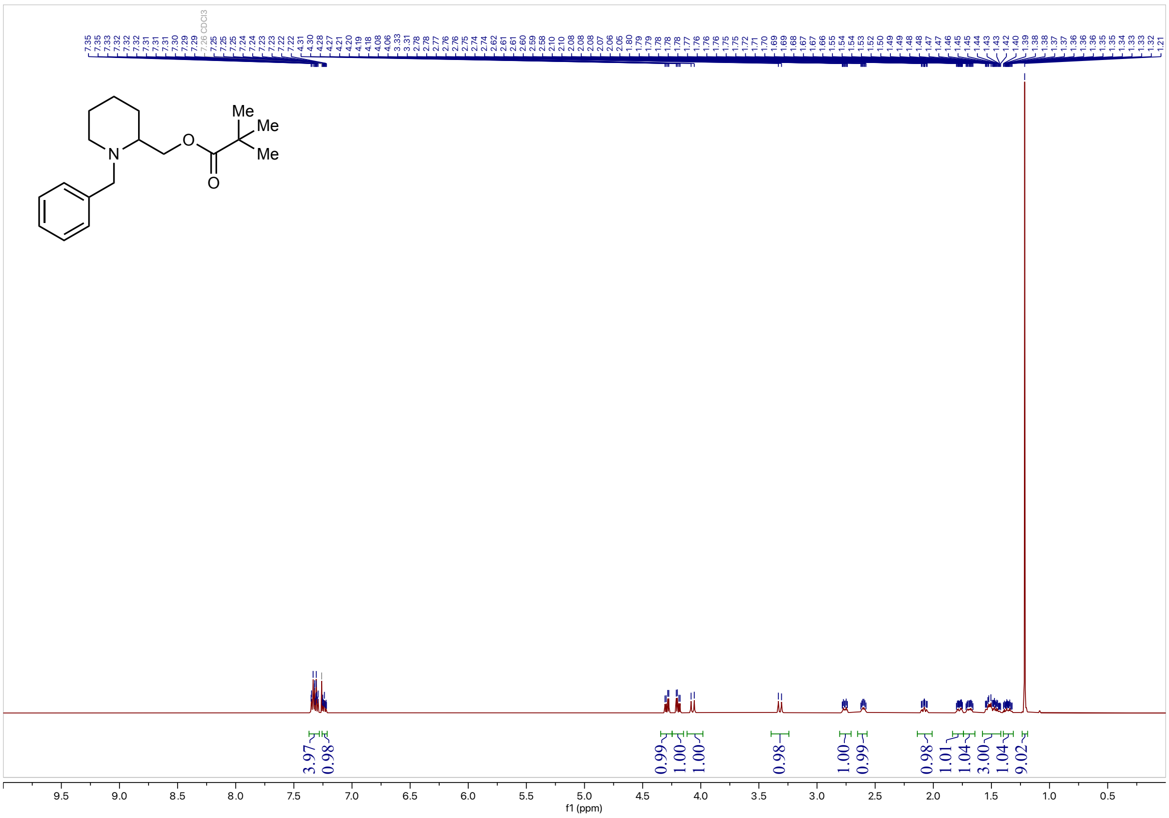

^{13}C{H} NMR (126 MHz, $CDCl_3$) of (1-benzylpiperidin-2-yl) methyl pivalate (**6f**):

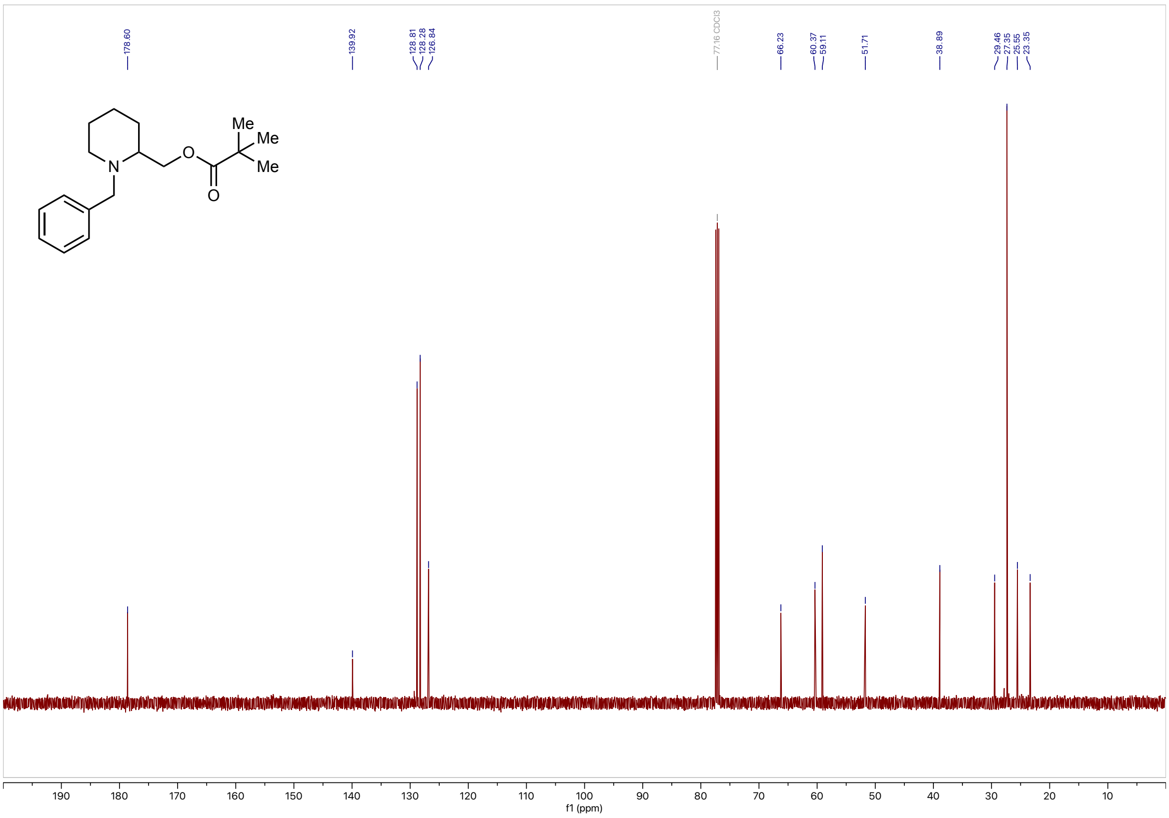

^{1}H NMR (500 MHz, $CDCl_3$) of (1-benzylpiperidin-2-yl) methyl benzoate (**6g**):

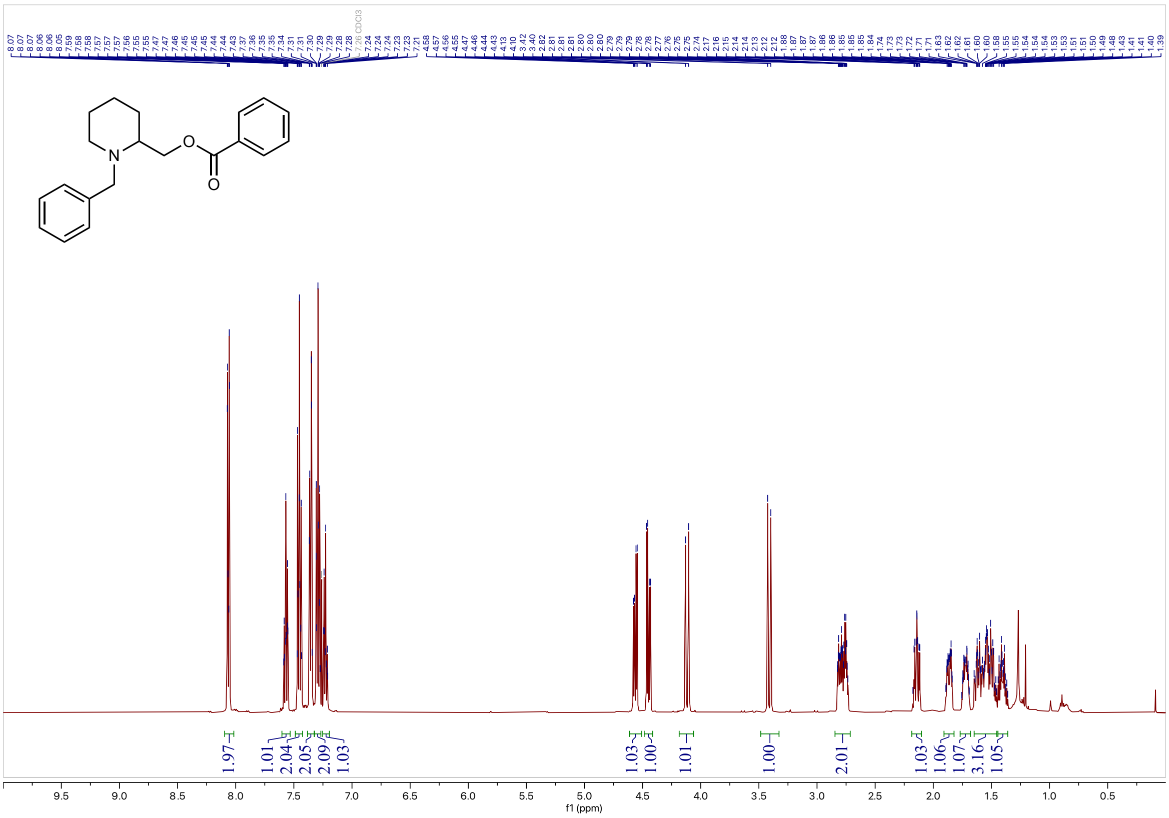

^{13}C{H} NMR (126 MHz, $CDCl_3$) of (1-benzylpiperidin-2-yl) methyl benzoate (**6g**):

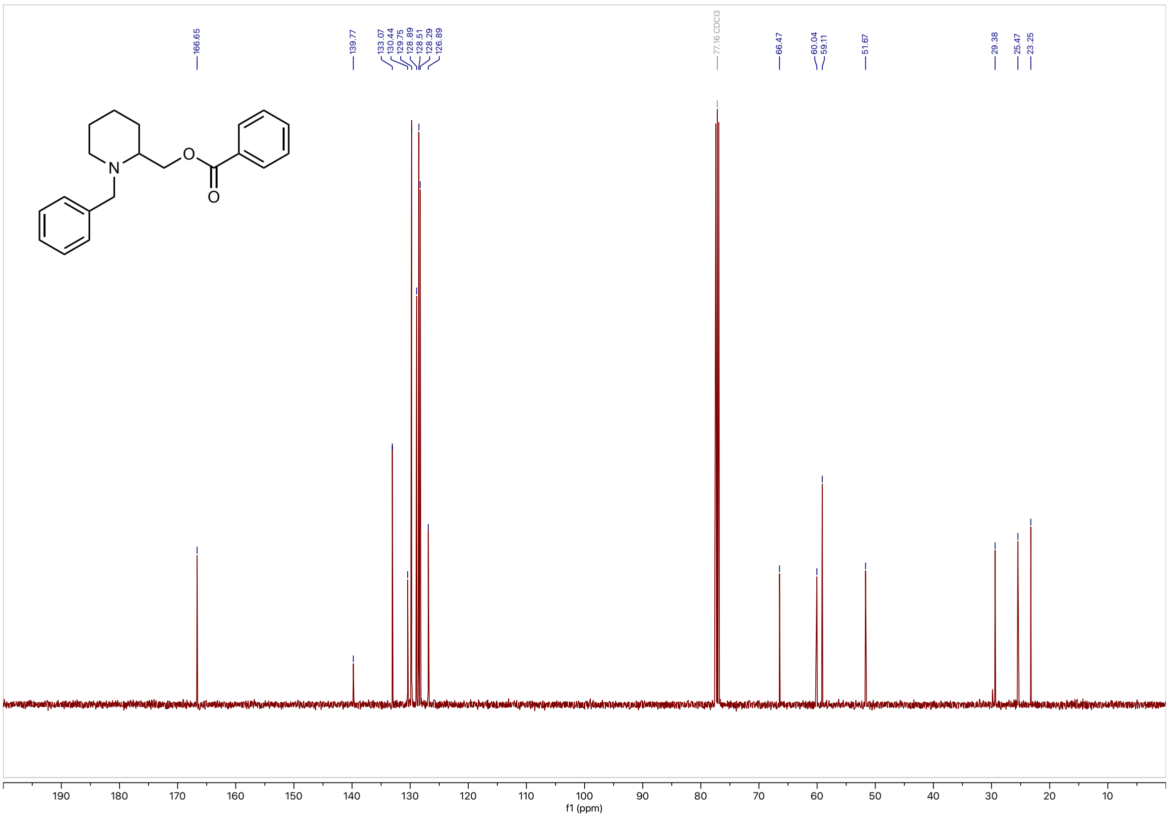

^{1}H NMR (400 MHz, $CDCl_3$) of (1-benzylpiperidin-2-yl) methanol (**6h**):

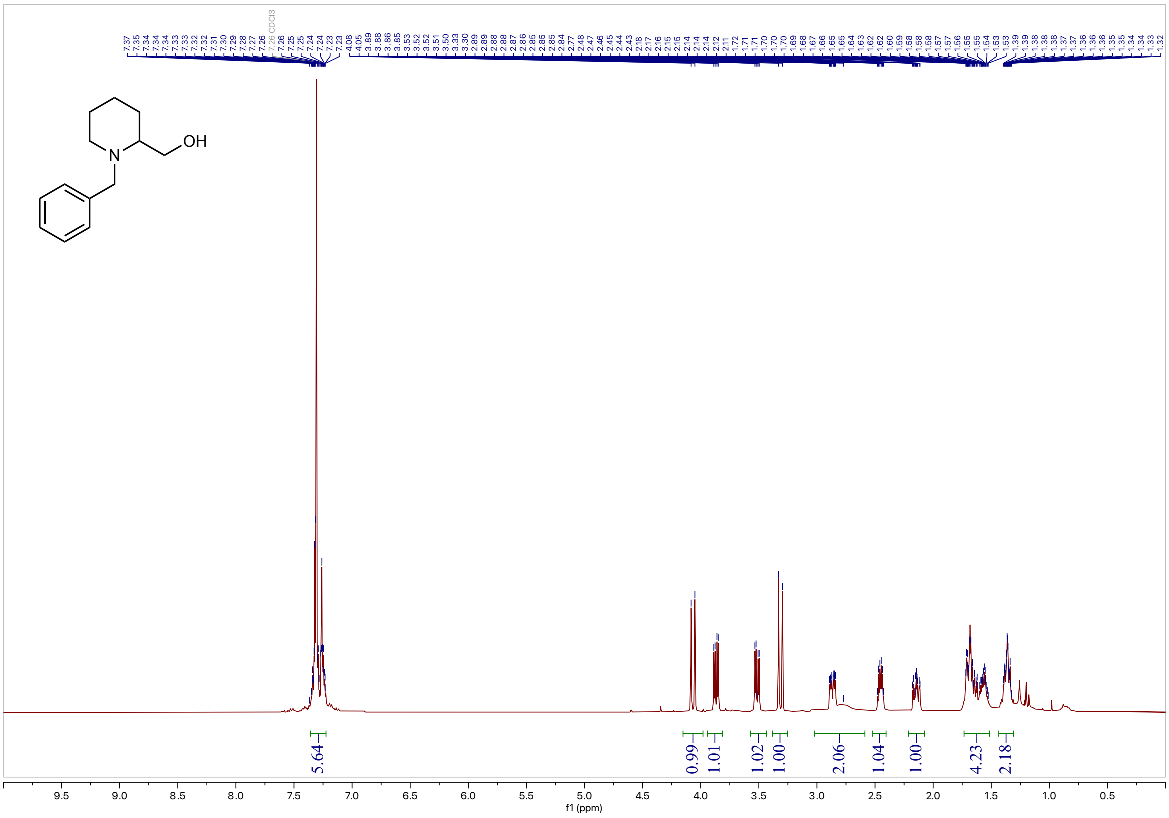

$^{13}C\{H\}$ NMR (126 MHz, $CDCl_3$) of (1-benzylpiperidin-2-yl) methanol (**6h**):

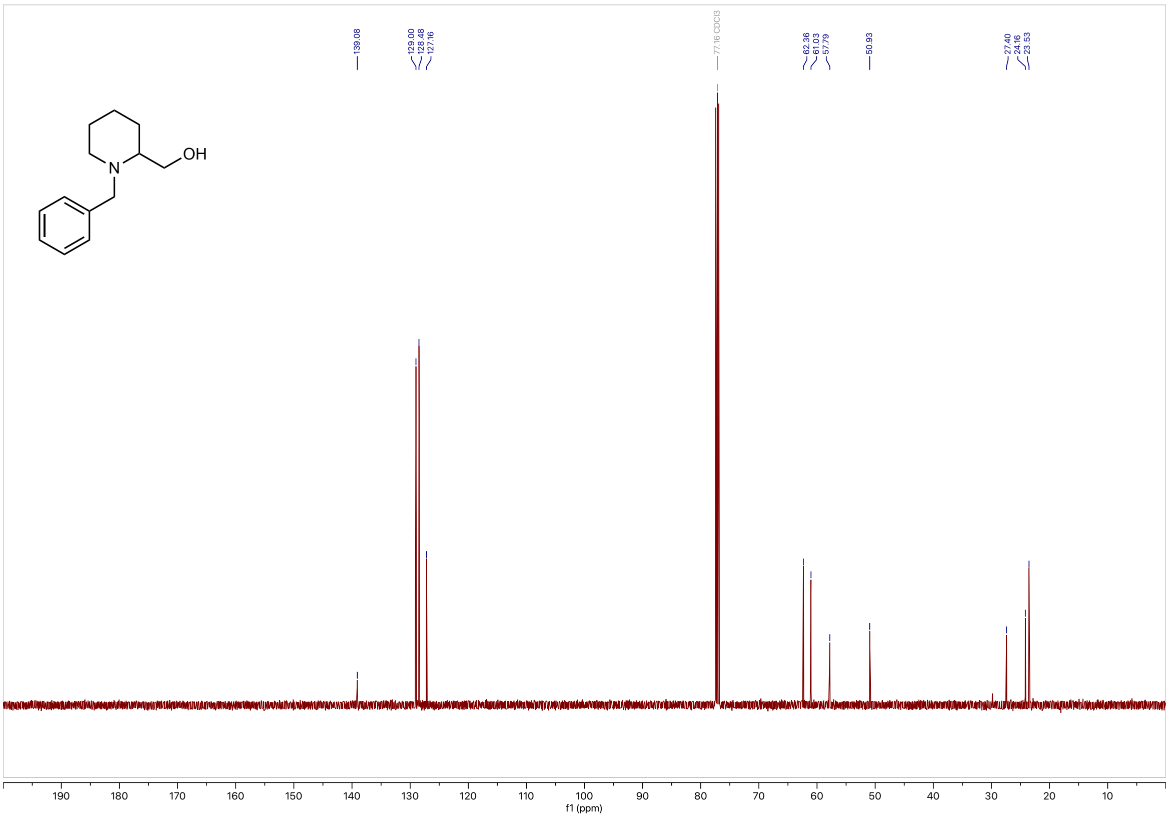

^{1}H NMR (500 MHz, $CDCl_3$) of 1-(4-phenyl-benzyl)-2-(fluoromethyl)piperidine (**6i**):

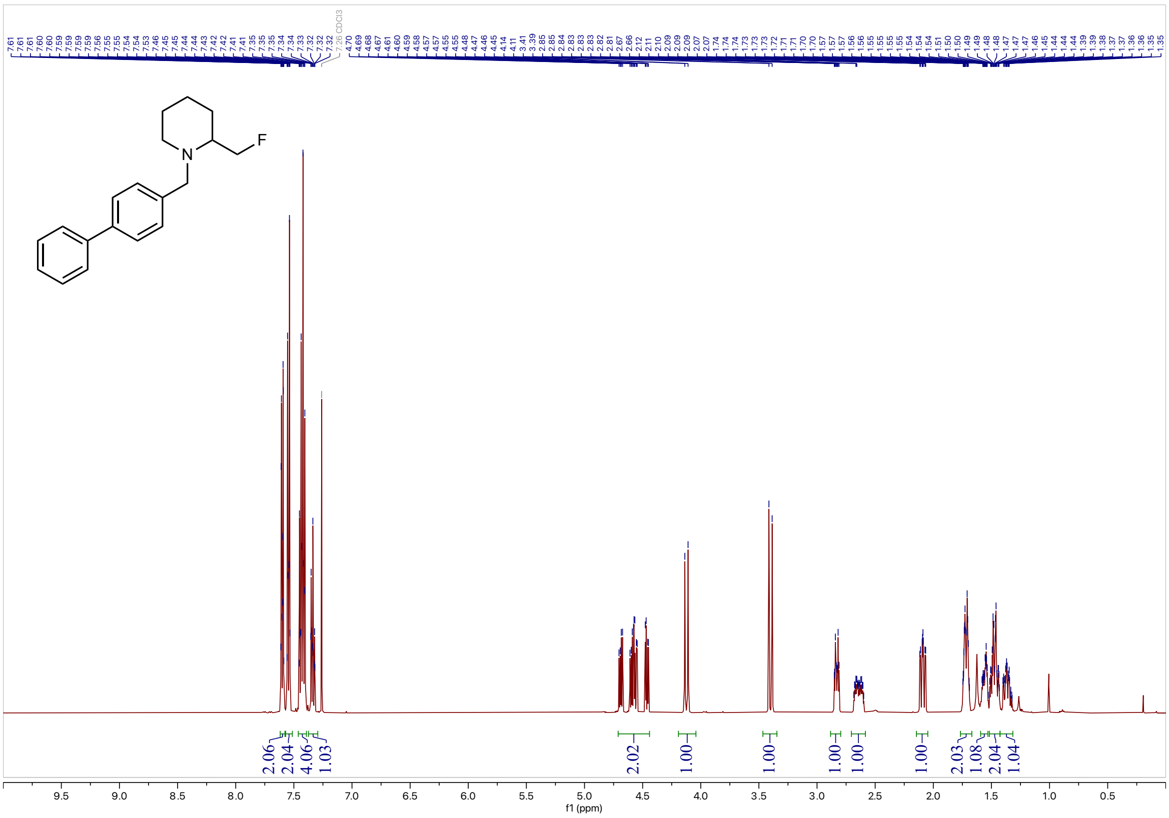

^{13}C{H} NMR (126 MHz, $CDCl_3$) of 1-(4-phenyl-benzyl)-2-(fluoromethyl)piperidine (**6i**):

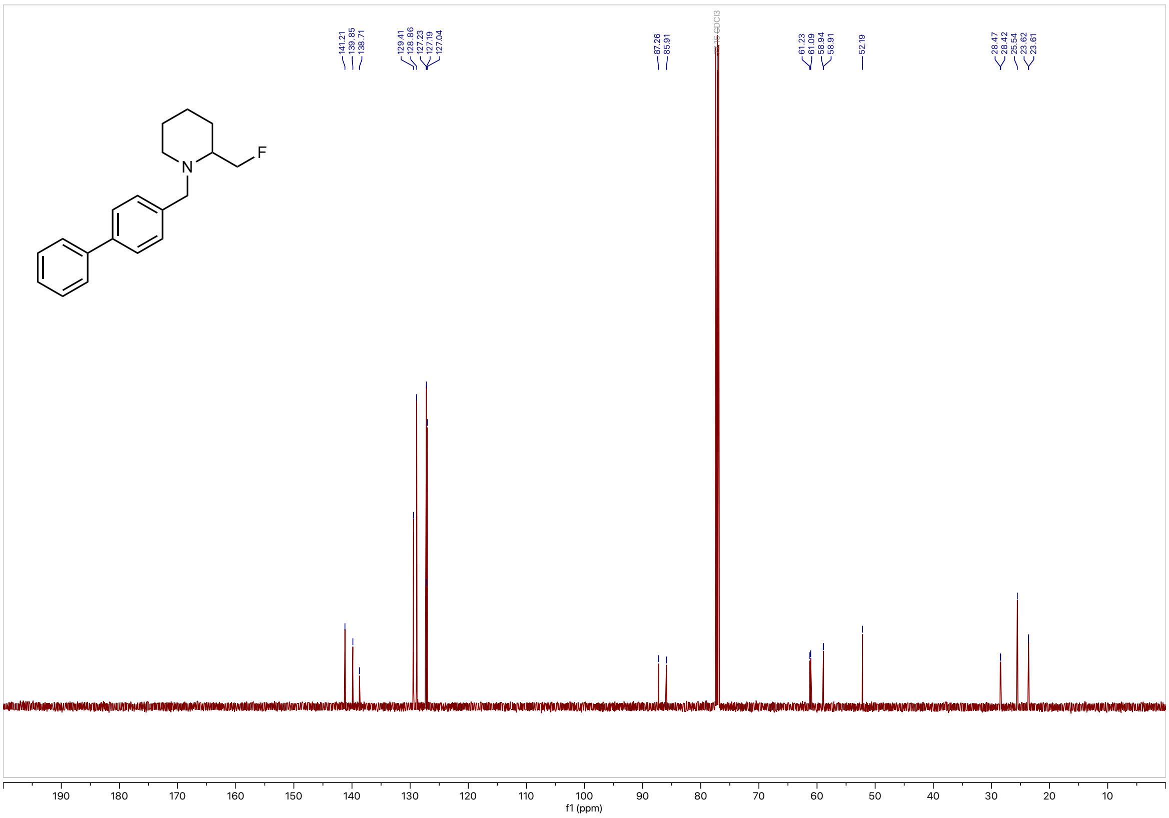

$^{19}F\{H\}$ NMR (471 MHz, $CDCl_3$) of 1-(4-phenyl-benzyl)-2-(fluoromethyl)piperidine (**6i**):

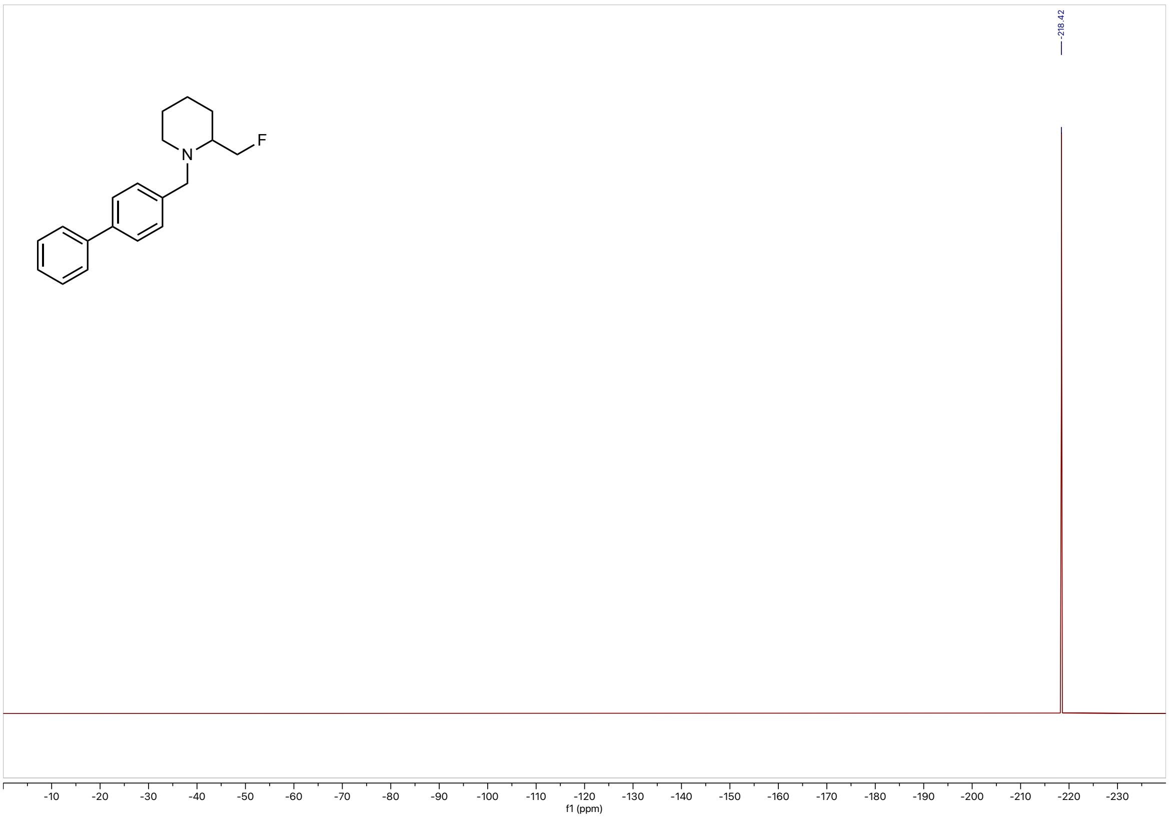

^{1}H NMR (400 MHz, $CDCl_3$) of 1-benzyl-2-(methoxymethyl) piperidine (**6j**):

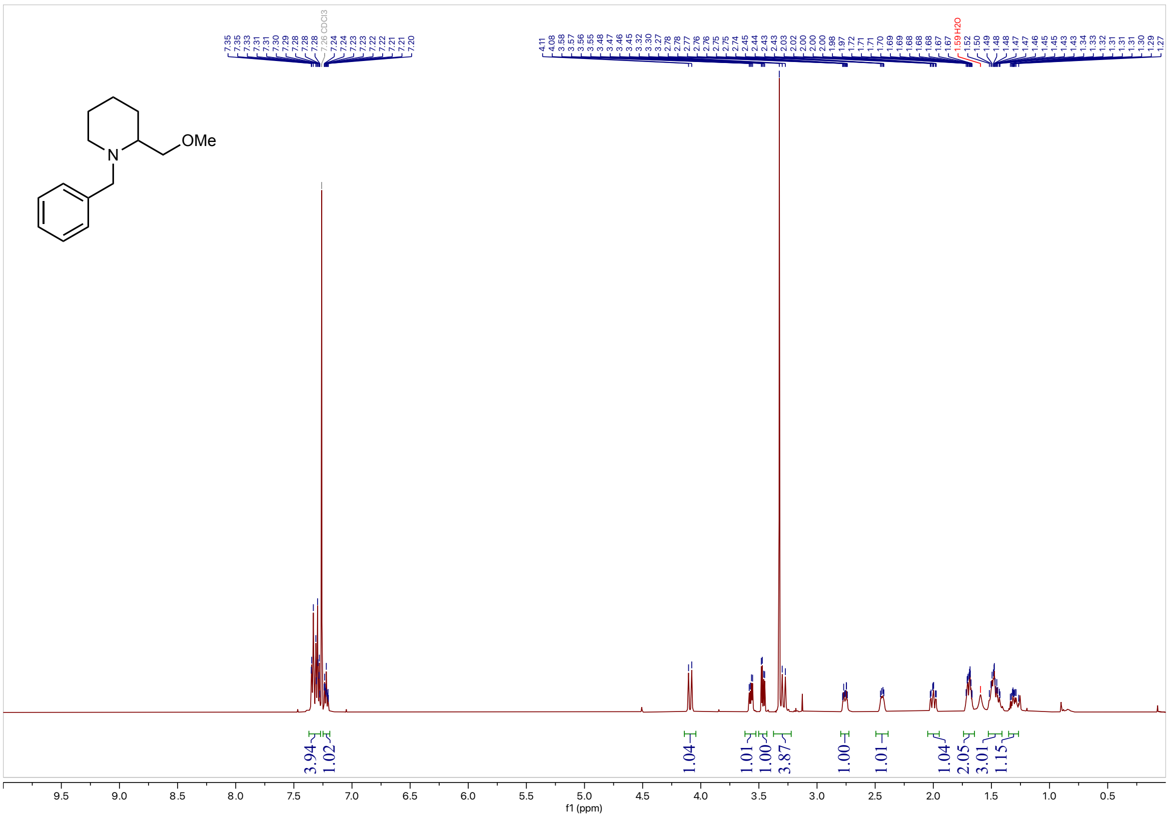

$^{13}C\{H\}$ NMR (126 MHz, $CDCl_3$) of 1-benzyl-2-(methoxymethyl) piperidine (**6j**):

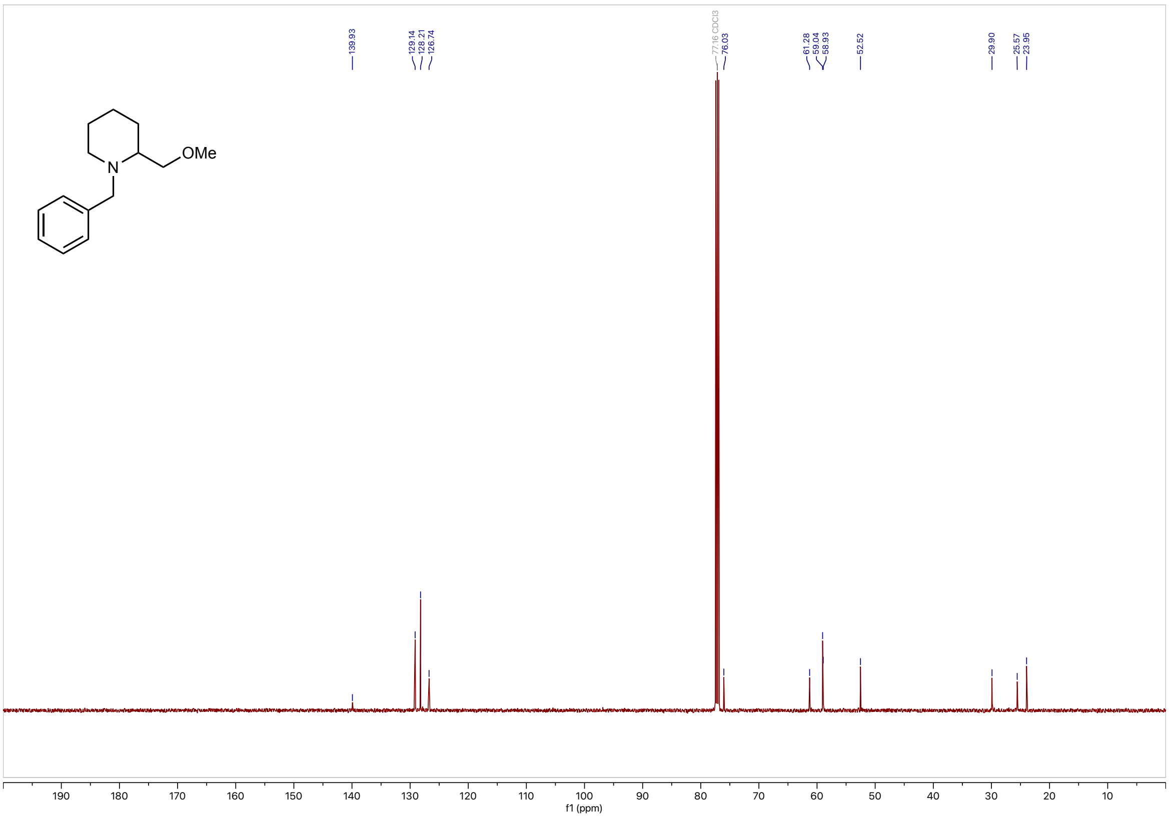

^{1}H NMR (500 MHz, $CDCl_3$) of 1-benzyl-2-(tetrahydro-2*H*-pyran-4-yl)piperidine (**6k**):

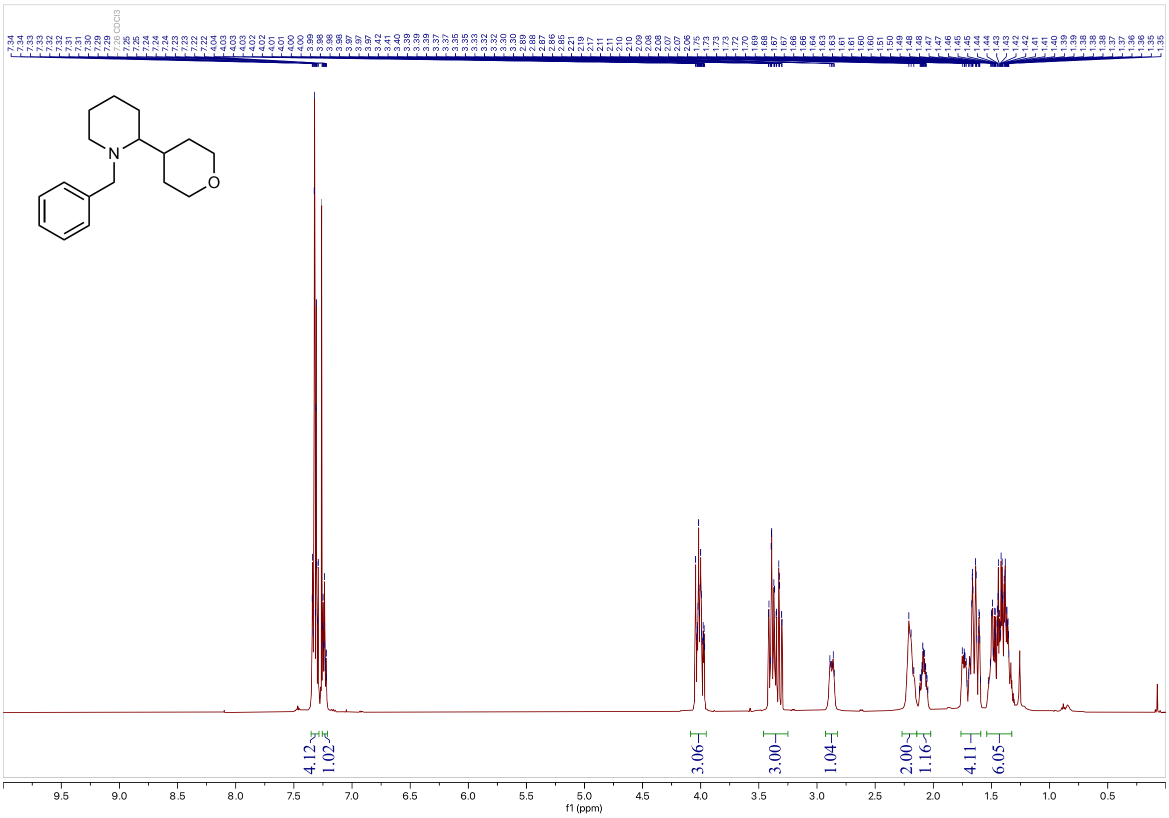

^{13}C{H} NMR (126 MHz, $CDCl_3$) of 1-benzyl-2-(tetrahydro-2*H*-pyran-4-yl)piperidine (**6k**):

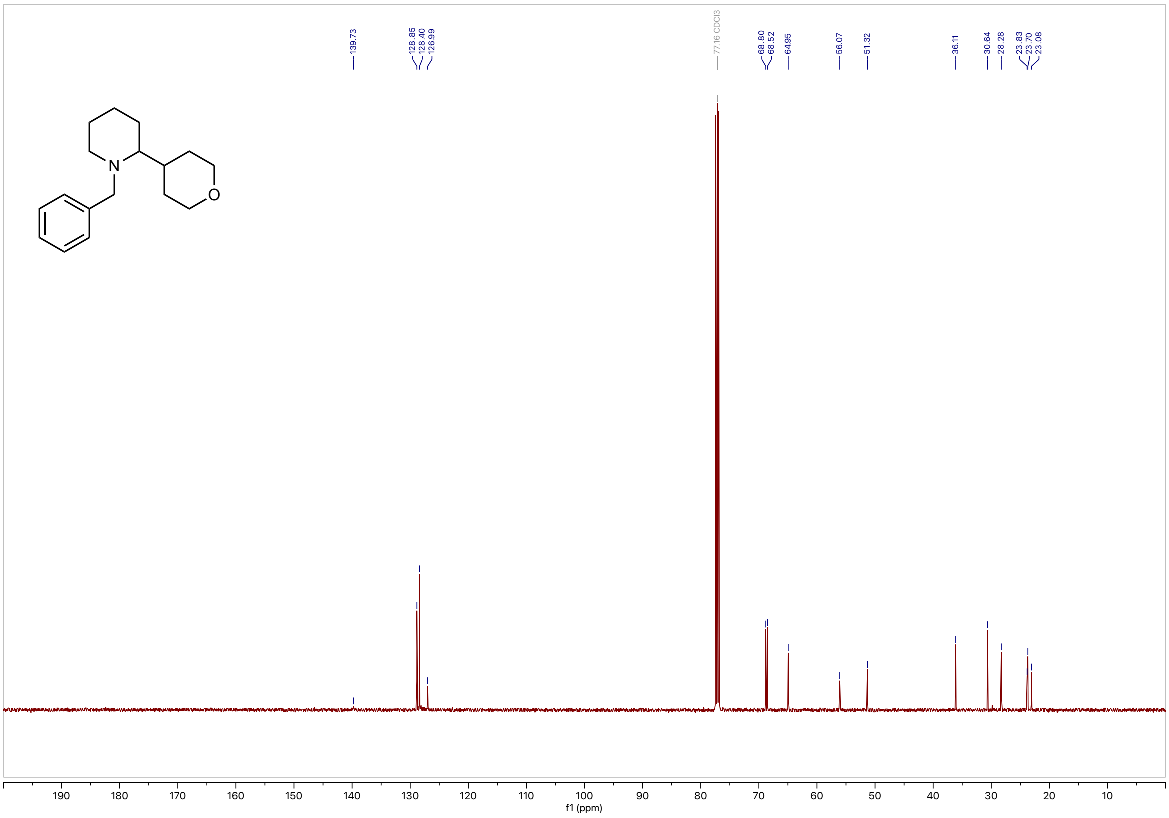

^{1}H NMR (700 MHz, $CDCl_3$) of Benzyl 1-benzyl-[2,4'-bipiperidine]-1'-carboxylate (**6l**):

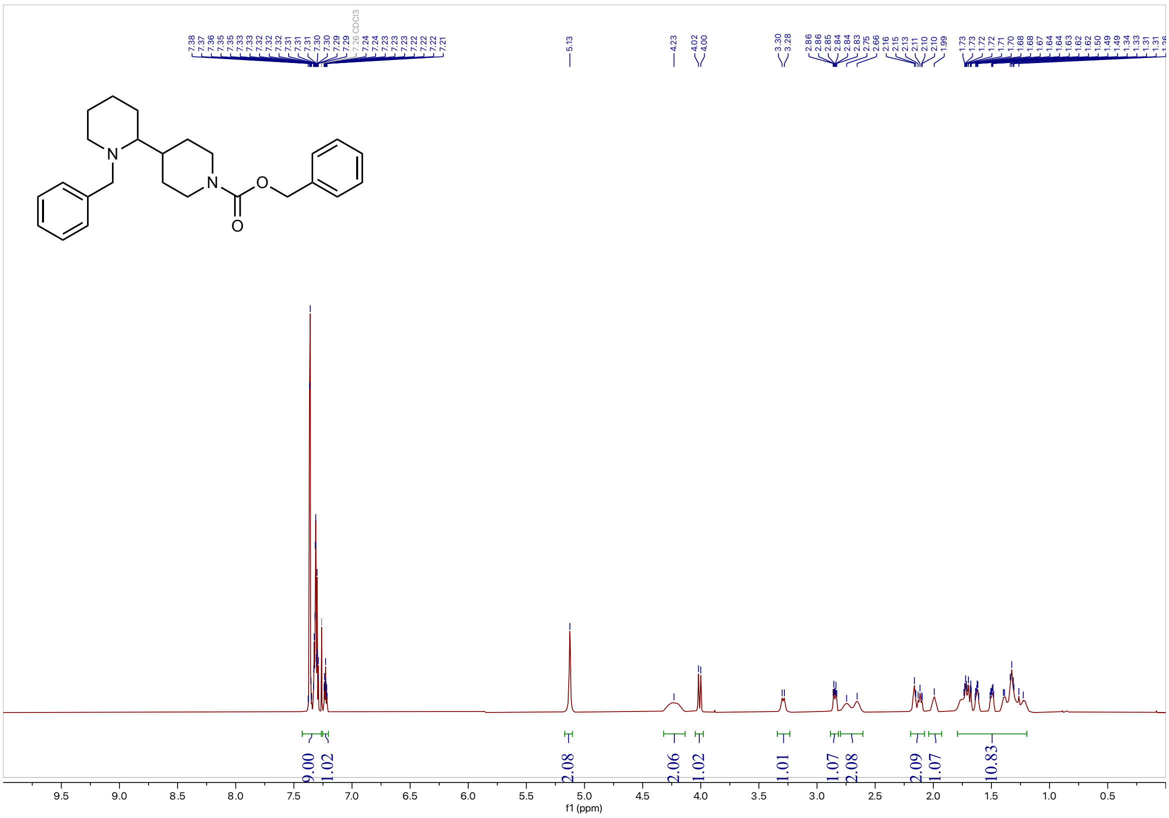

^{13}C{H} NMR (176 MHz, $CDCl_3$) of Benzyl 1-benzyl-[2,4'-bipiperidine]-1'-carboxylate (**6l**):

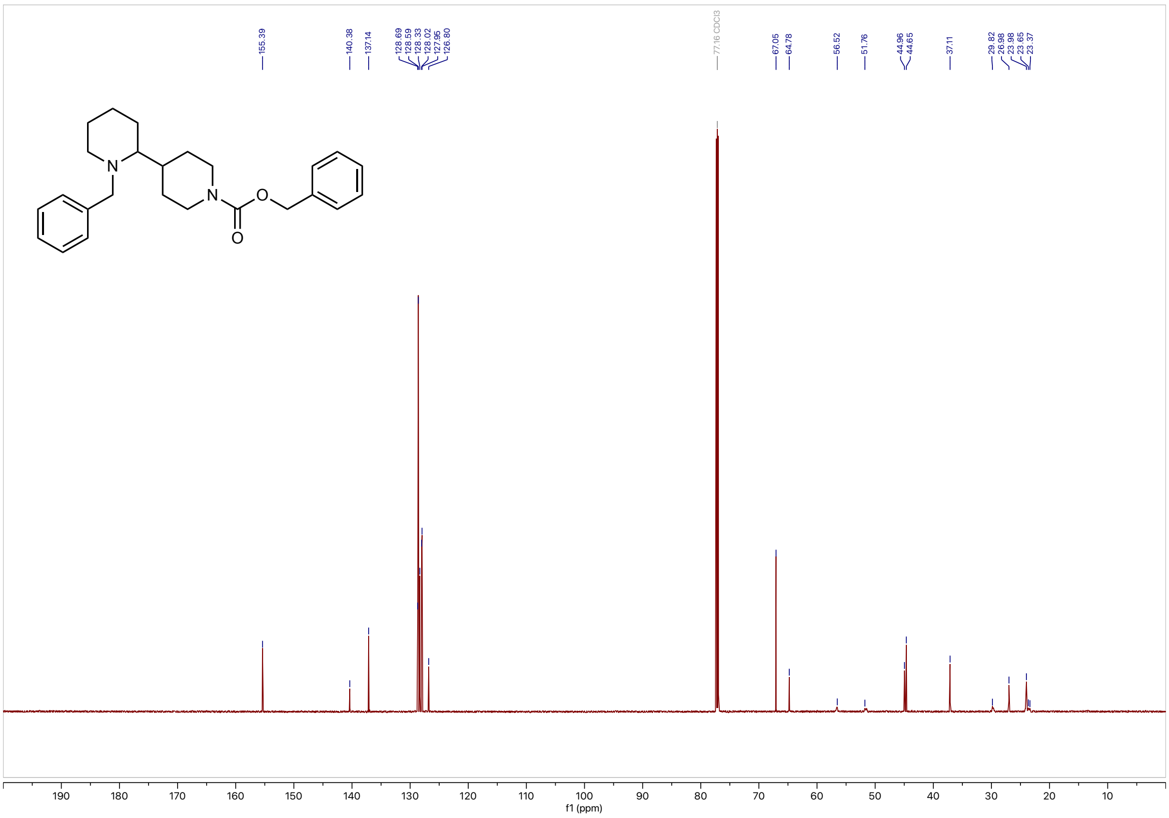

^{1}H NMR (500 MHz, $CDCl_3$) of (1-benzyl-2-piperidine)acetonitrile (**6m**):

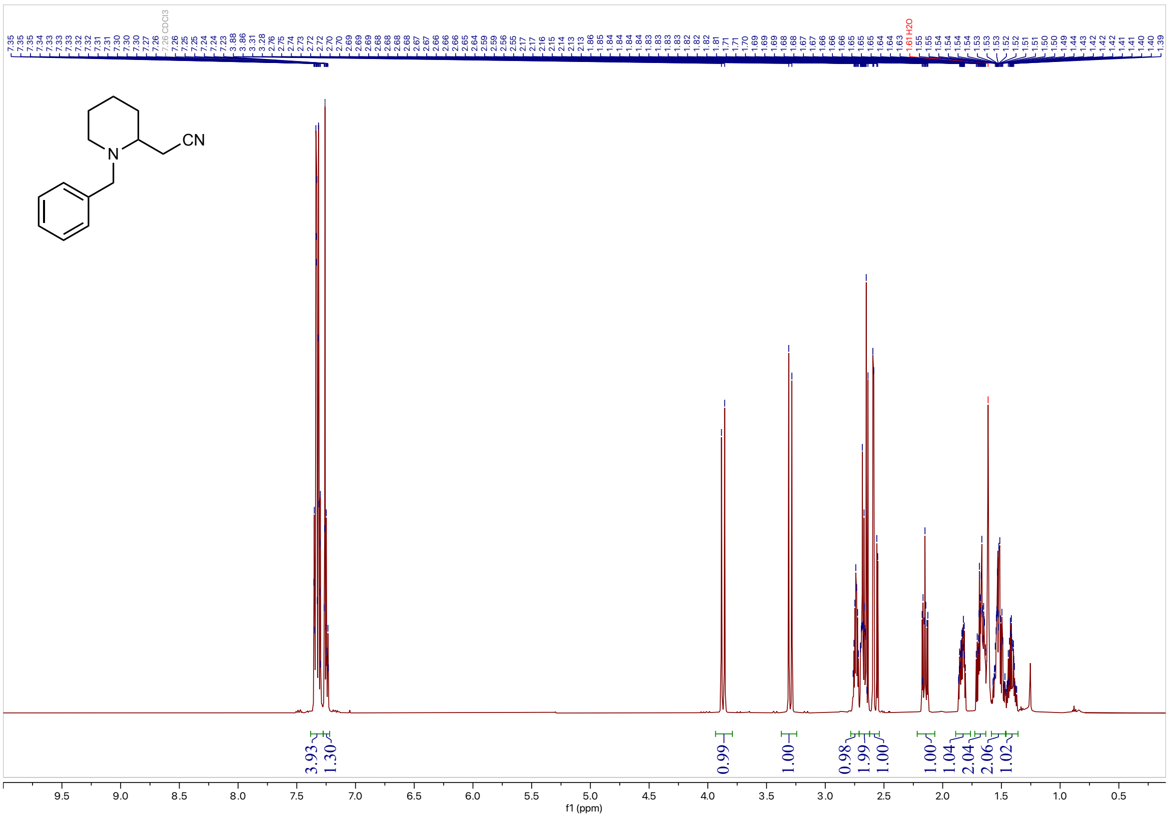

^{13}C{H} NMR (126 MHz, $CDCl_3$) of (1-benzyl-2-piperidine)acetonitrile (**6m**):

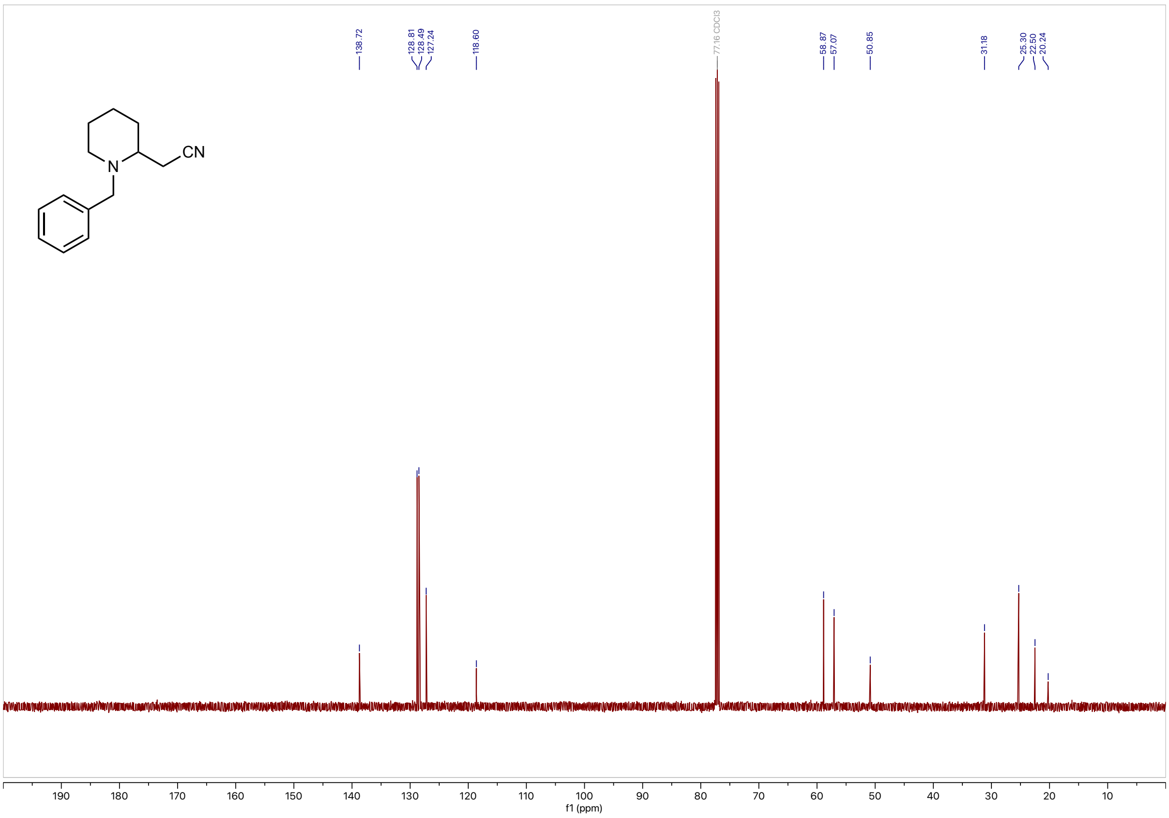

^{1}H NMR (400 MHz, $CDCl_3$) of 2-(1-benzylpiperidin-2-yl)acetamide (**6n**):

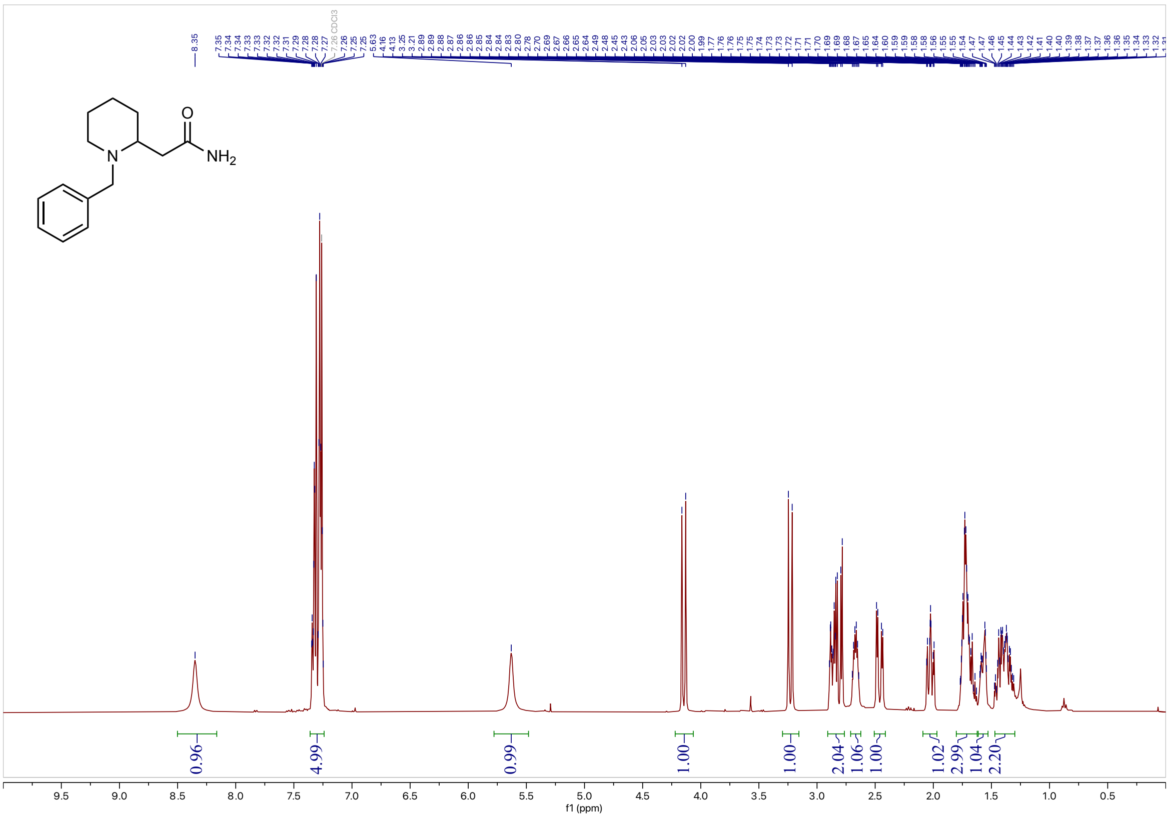

^{13}C{H} NMR (101 MHz, $CDCl_3$) of 2-(1-benzylpiperidin-2-yl)acetamide (**6n**):

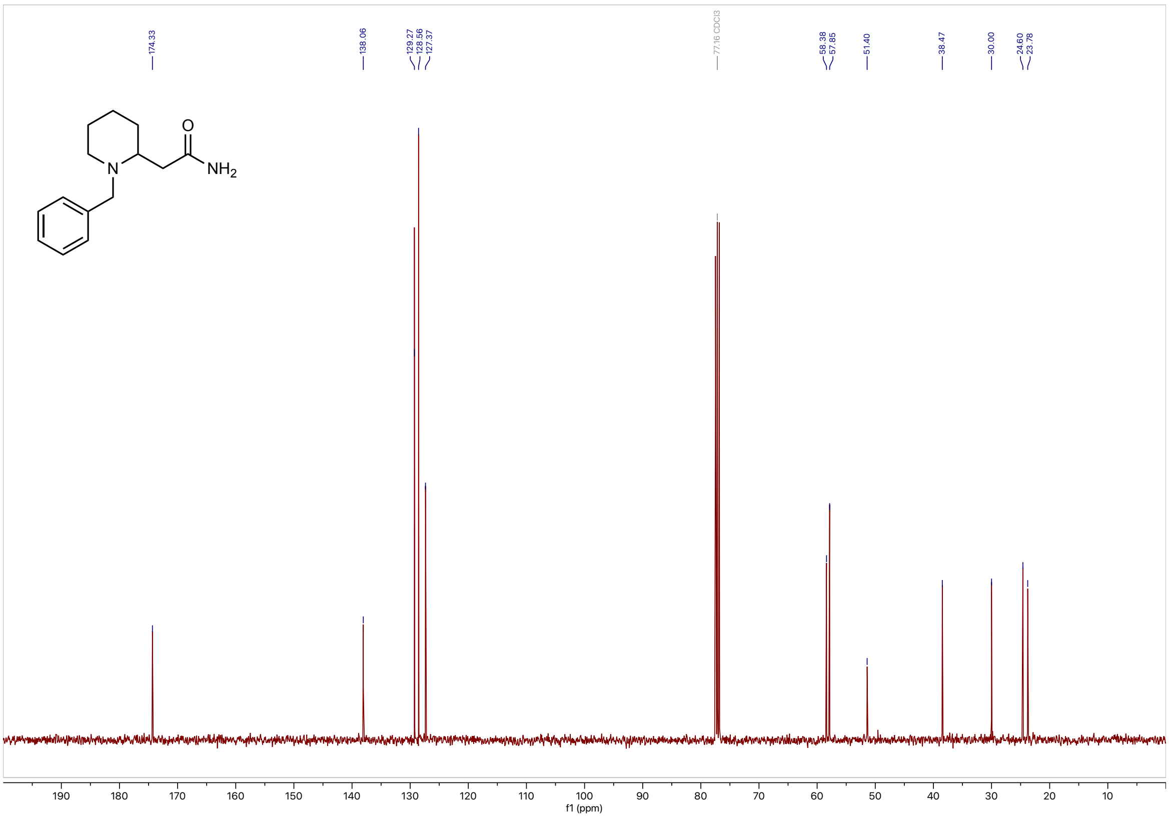

^{1}H NMR (700 MHz, $CDCl_3$) 2-(1-benzylpiperidin-2-yl)-*N,N*-dimethylacetamide (**6o**):

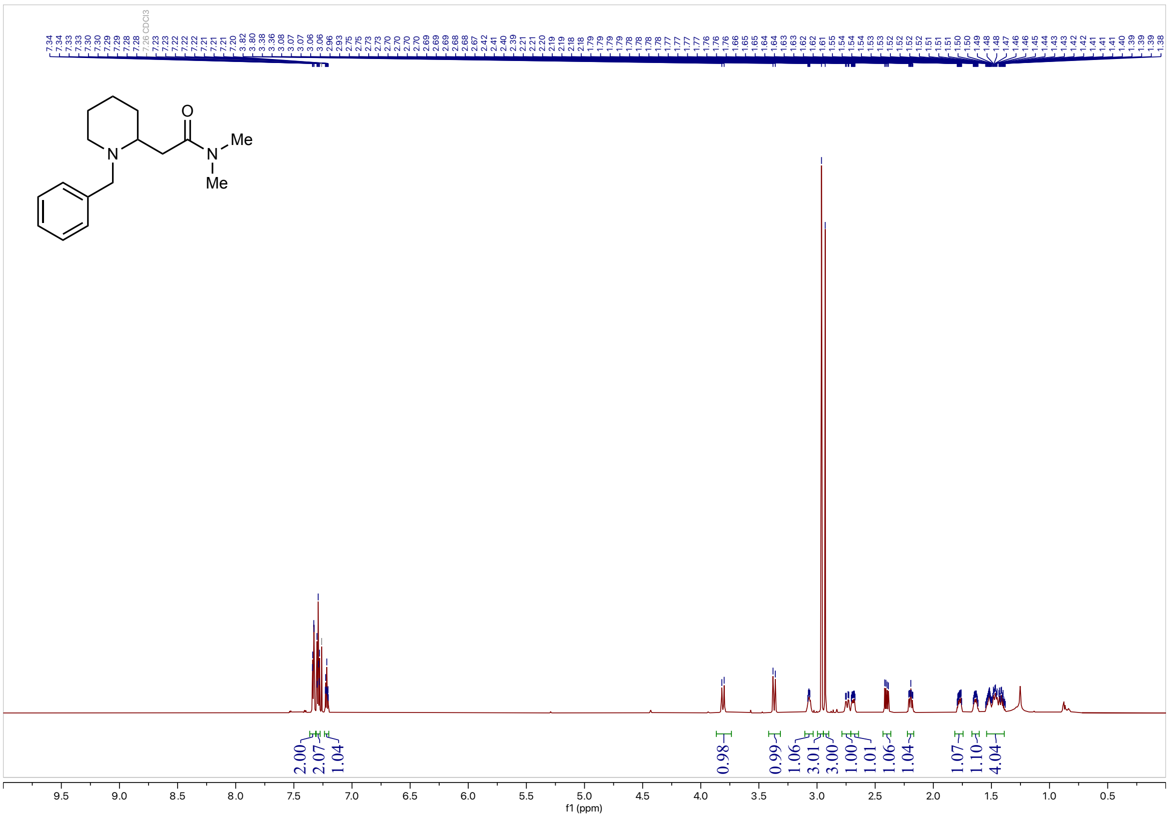

$^{13}C\{H\}$ NMR (101 MHz, $CDCl_3$) 2-(1-benzylpiperidin-2-yl)-*N,N*-dimethylacetamide (**6o**):

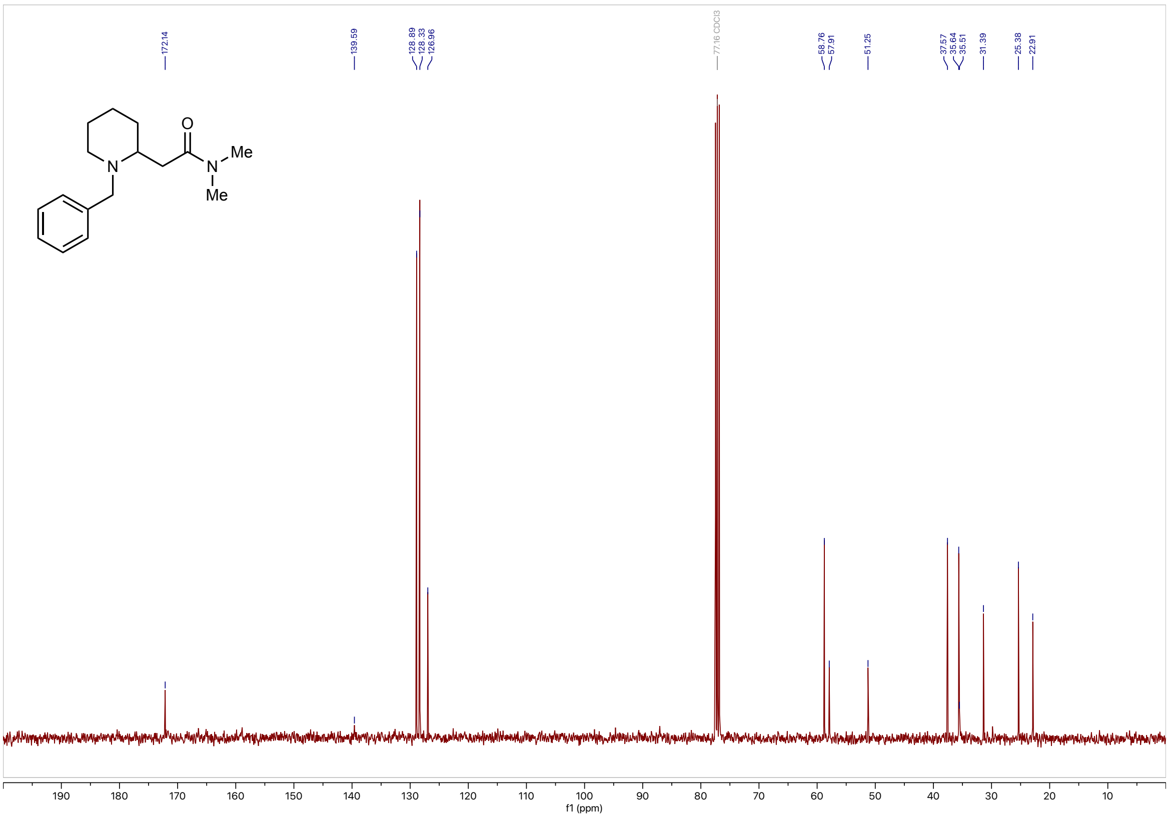

^{1}H NMR (700 MHz, $CDCl_3$) of ethyl 2-(N-benzylpiperidin-2-yl)-2-methylpropanoate (**6p**):

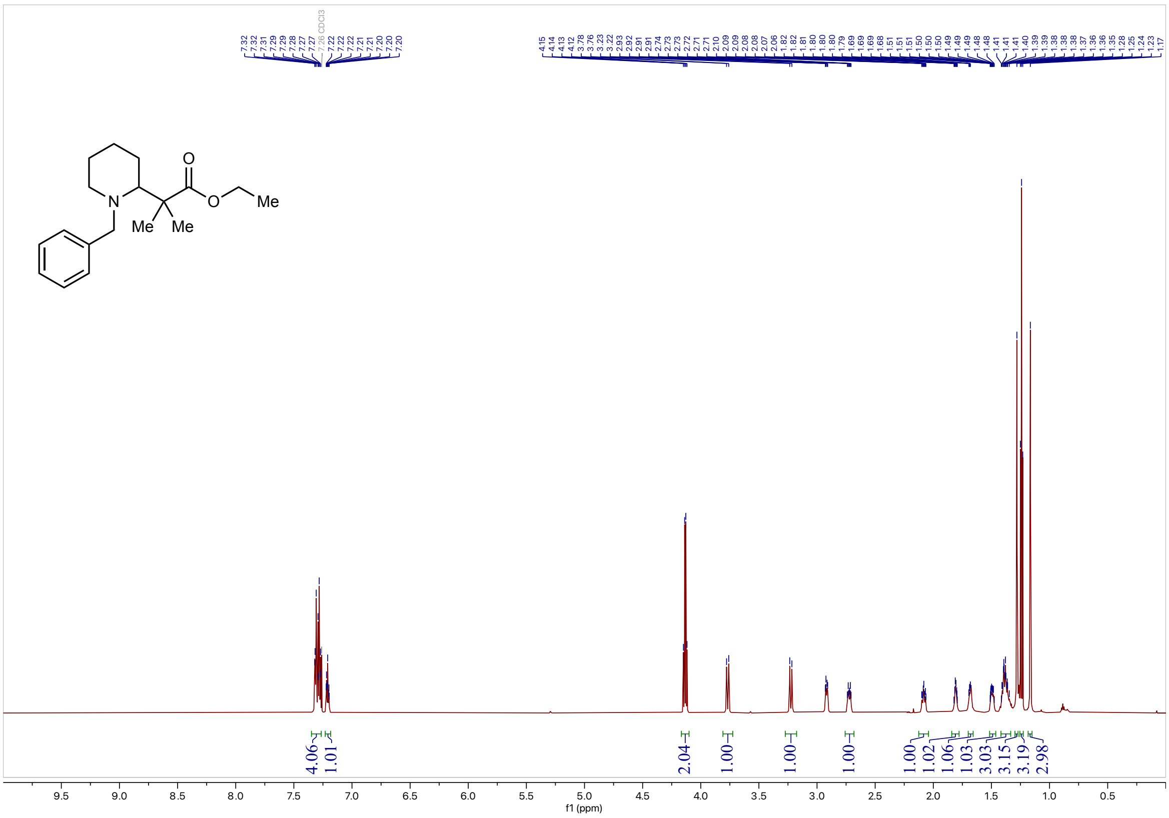

^{13}C{H} NMR (101 MHz, $CDCl_3$) of ethyl 2-(N-benzylpiperidin-2-yl)-2-methylpropanoate (**6p**):

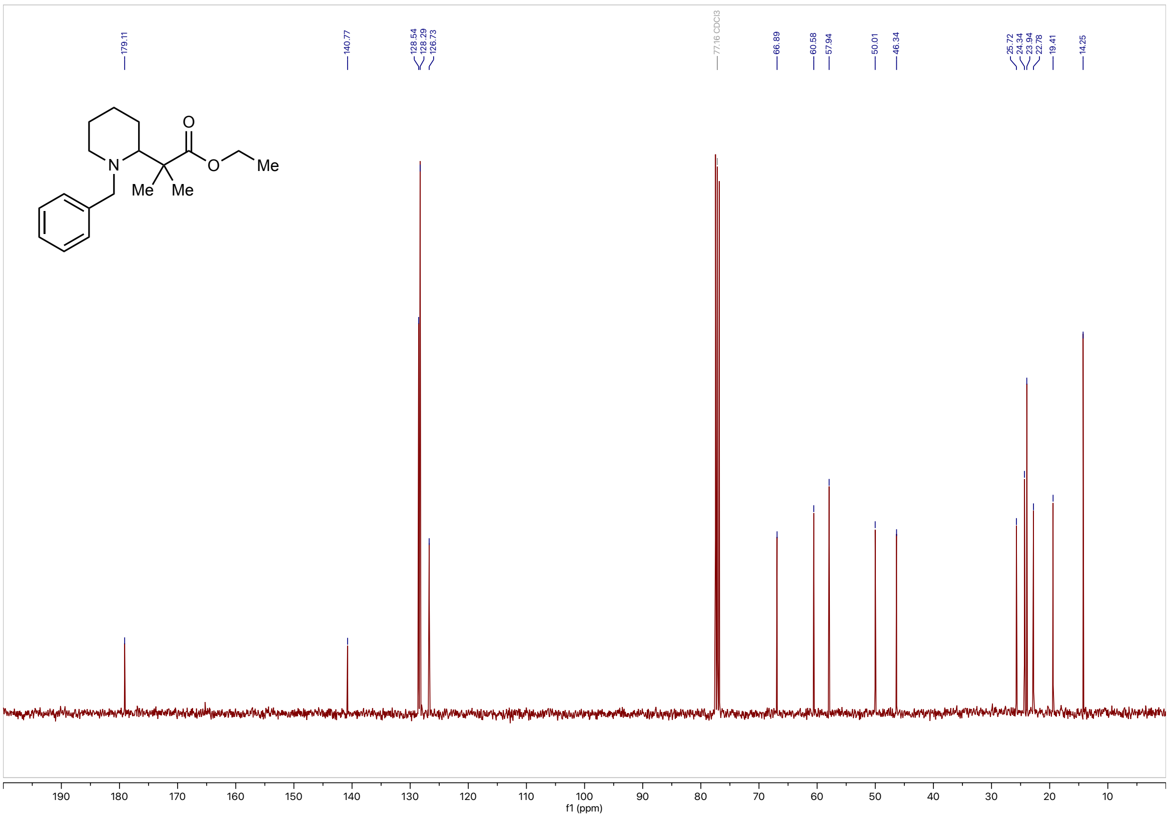

^{1}H NMR (700 MHz, $CDCl_3$) of 2-(adamantan-1-yl)-1-methylpiperidine (**6q**):

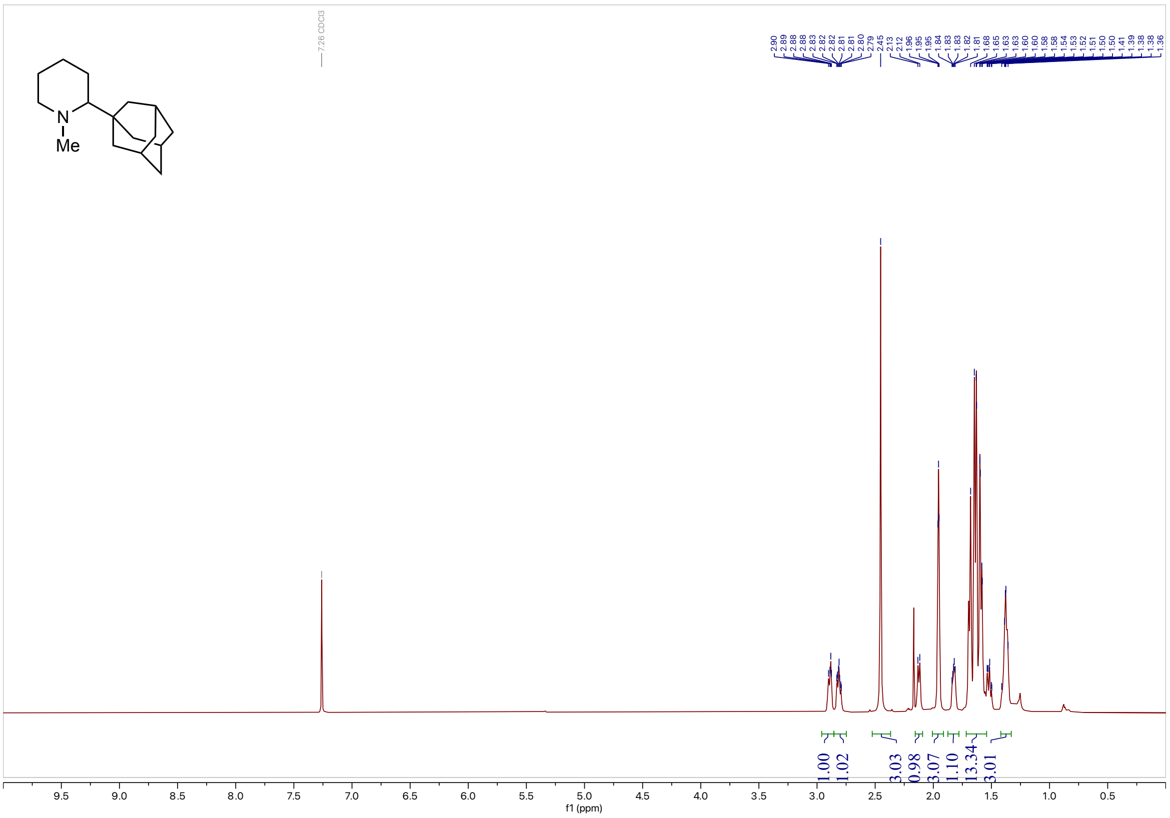

^{13}C{H} NMR (176 MHz, $CDCl_3$) of 2-(adamantan-1-yl)-1-methylpiperidine (**6q**):

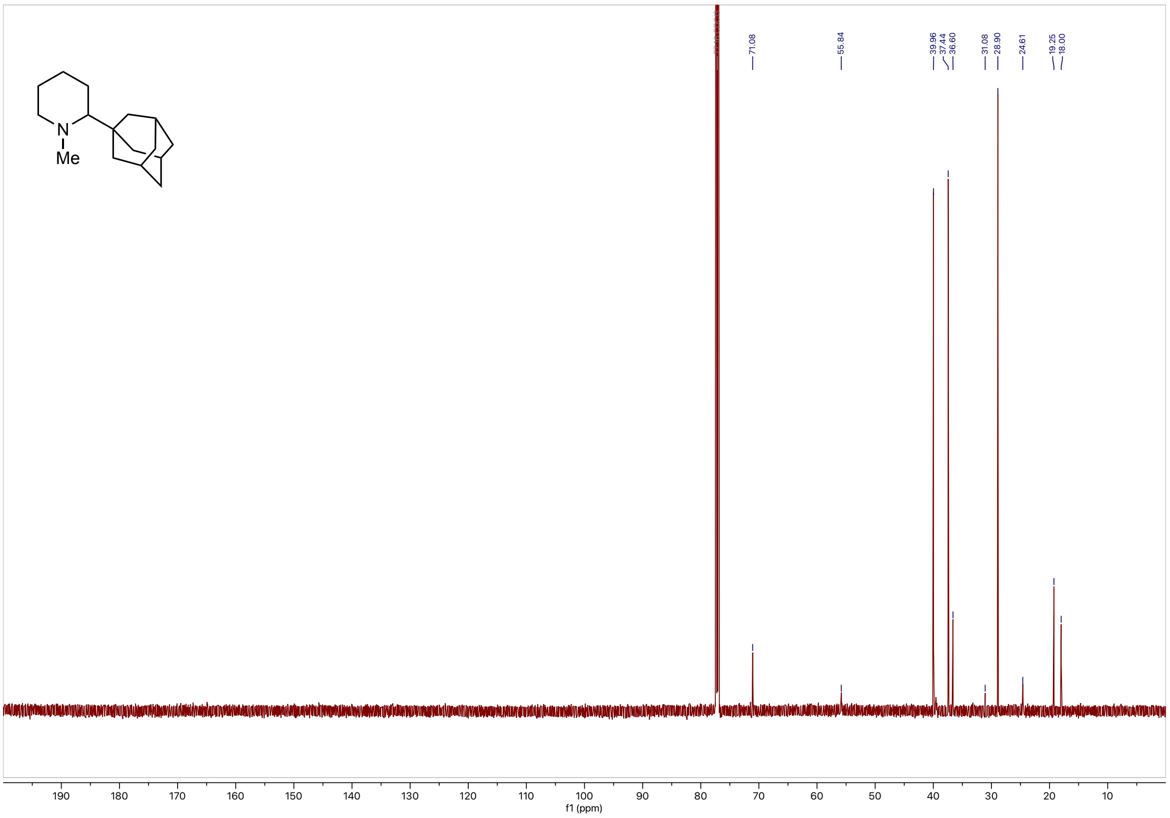

^{1}H NMR (700 MHz, $CDCl_3$) of 2-(tert-butyl)-1-(3-phenylpropyl)piperidine (**6r**):

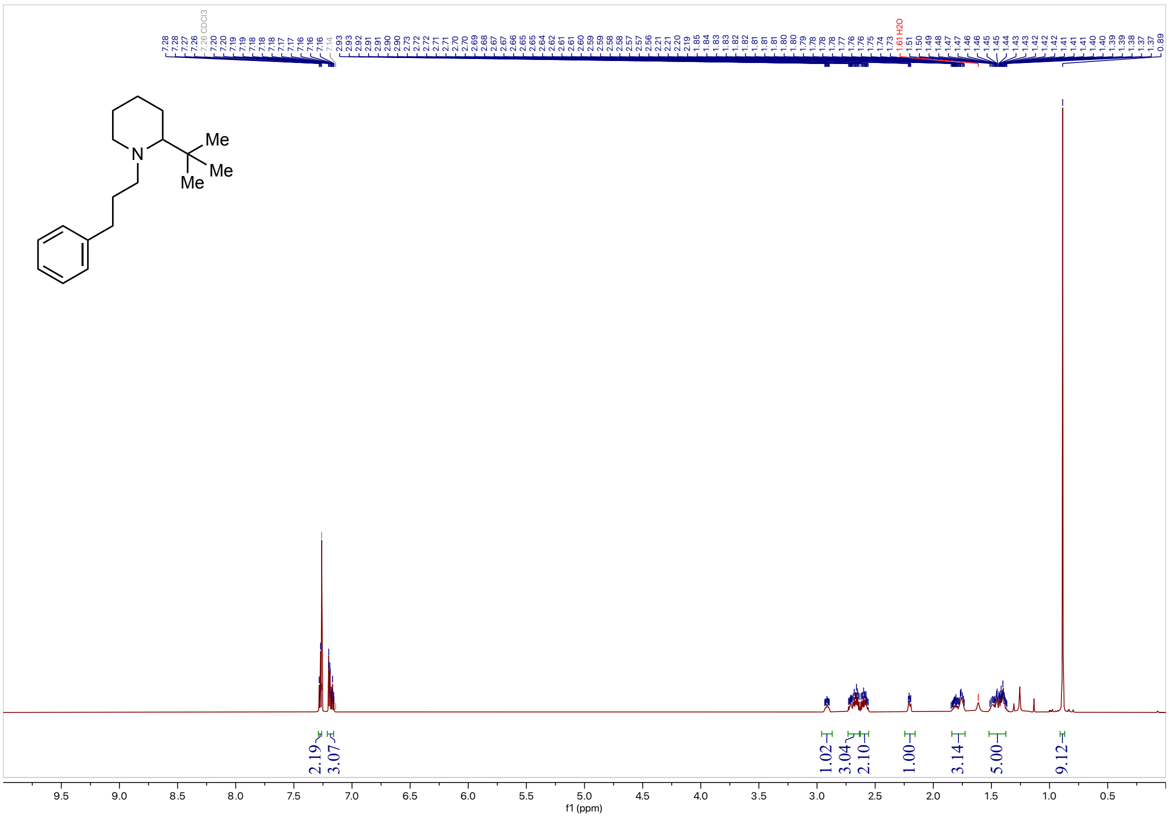

$^{13}C\{H\}$ NMR (176 MHz, $CDCl_3$) of 2-(tert-butyl)-1-(3-phenylpropyl)piperidine (**6r**):

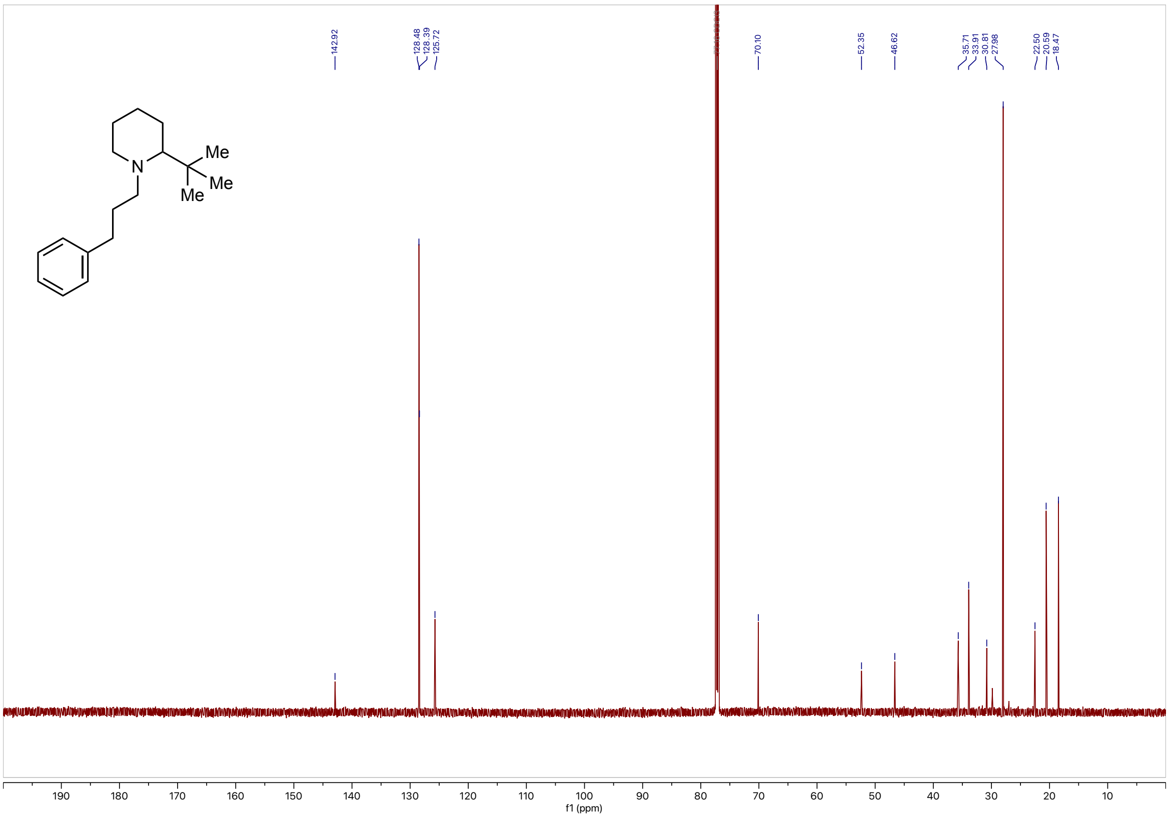

^1H NMR (700 MHz, $CDCl_3$) of 2-(adamantan-1-yl)-1-cyclohexylpiperidine (**6s**):

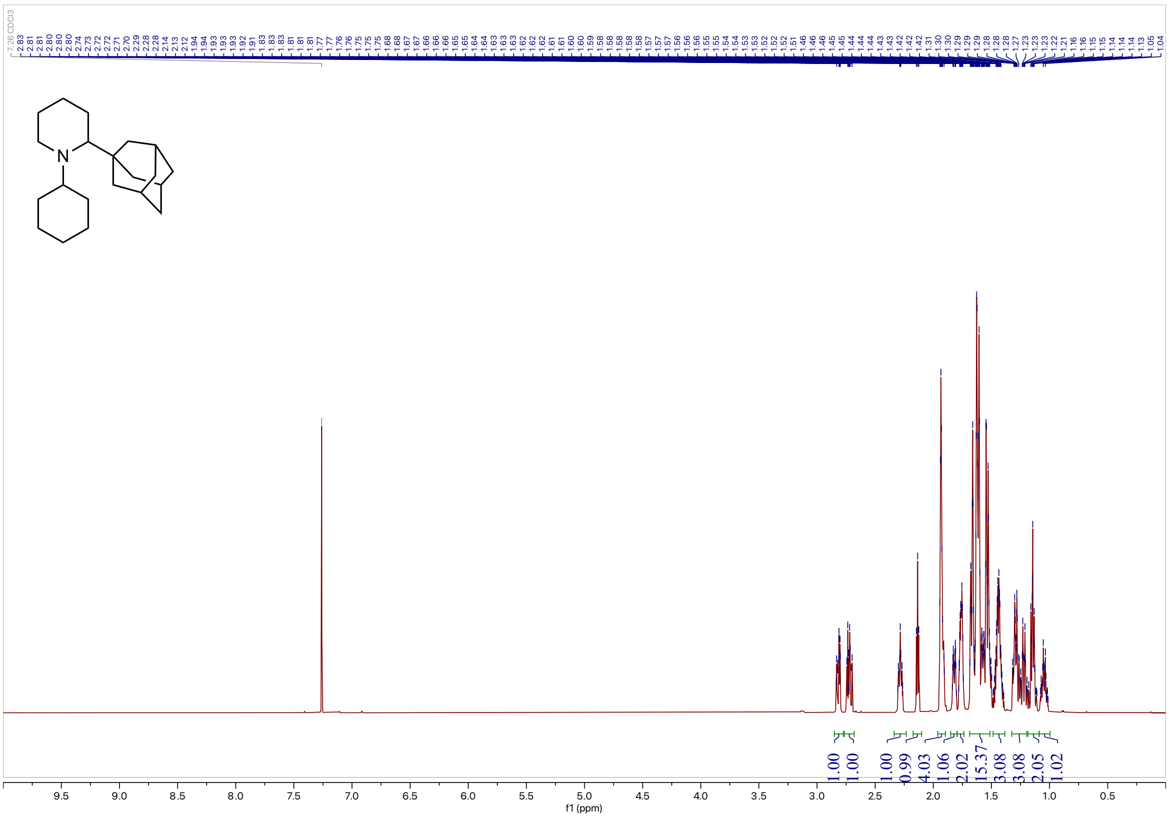

$^{13}C\{H\}$ NMR (176 MHz, $CDCl_3$) of 2-(adamantan-1-yl)-1-cyclohexylpiperidine (**6s**):

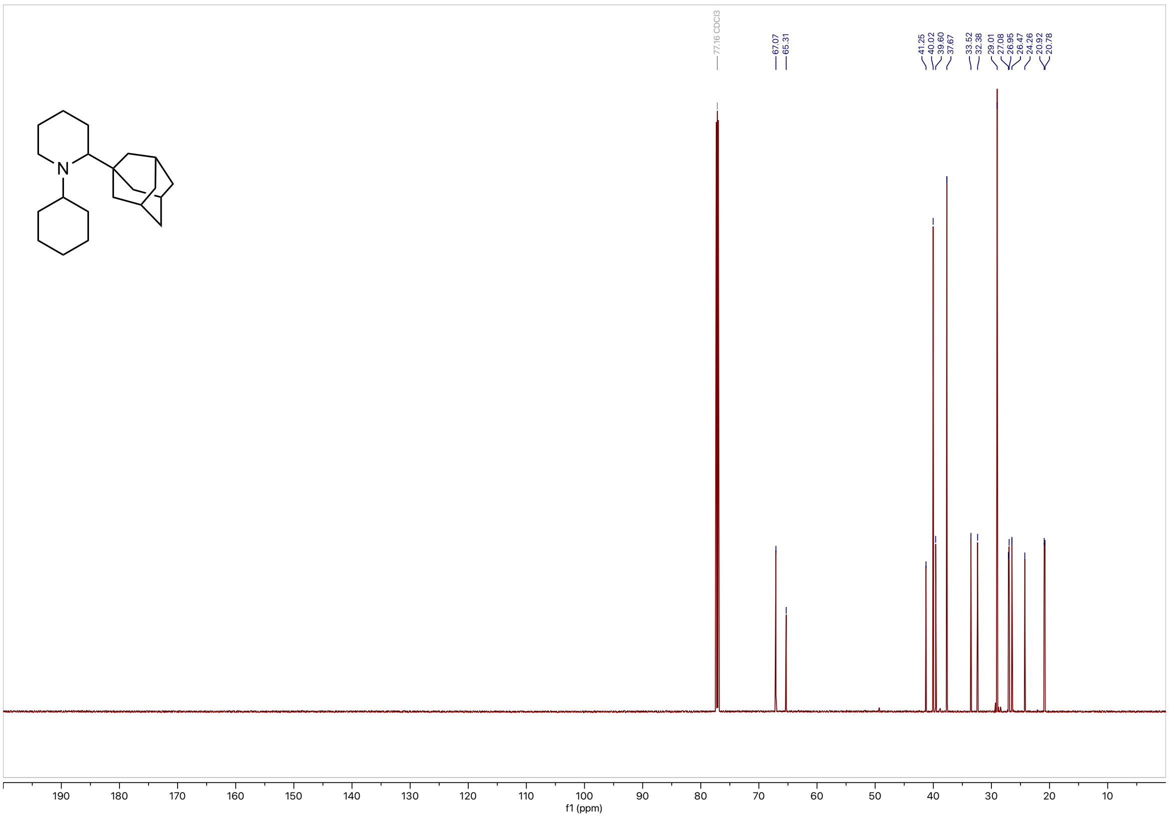

^{1}H NMR (700 MHz, $CDCl_3$) of 2-(adamantan-1-yl)-1-benzylpyrrolidine (**6u**):

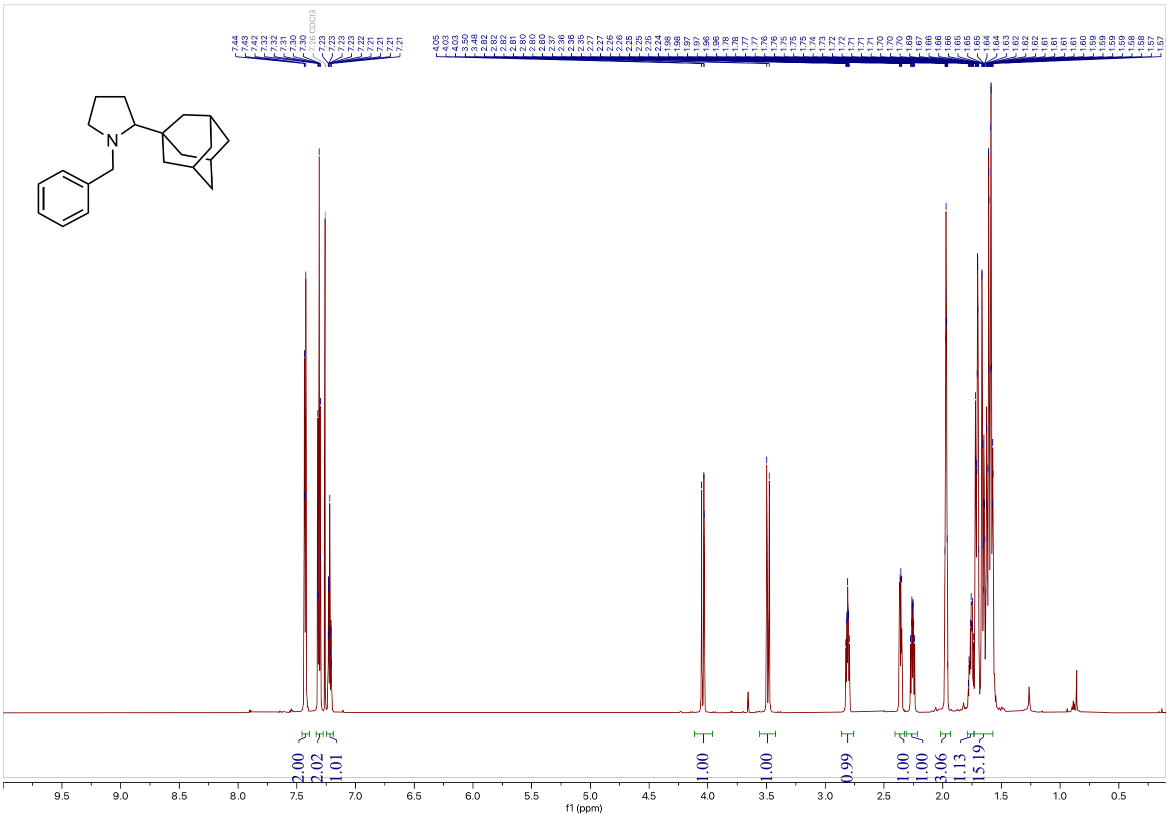

^{13}C{H} NMR (176 MHz, $CDCl_3$) of 2-(adamantan-1-yl)-1-benzylpyrrolidine (**6u**):

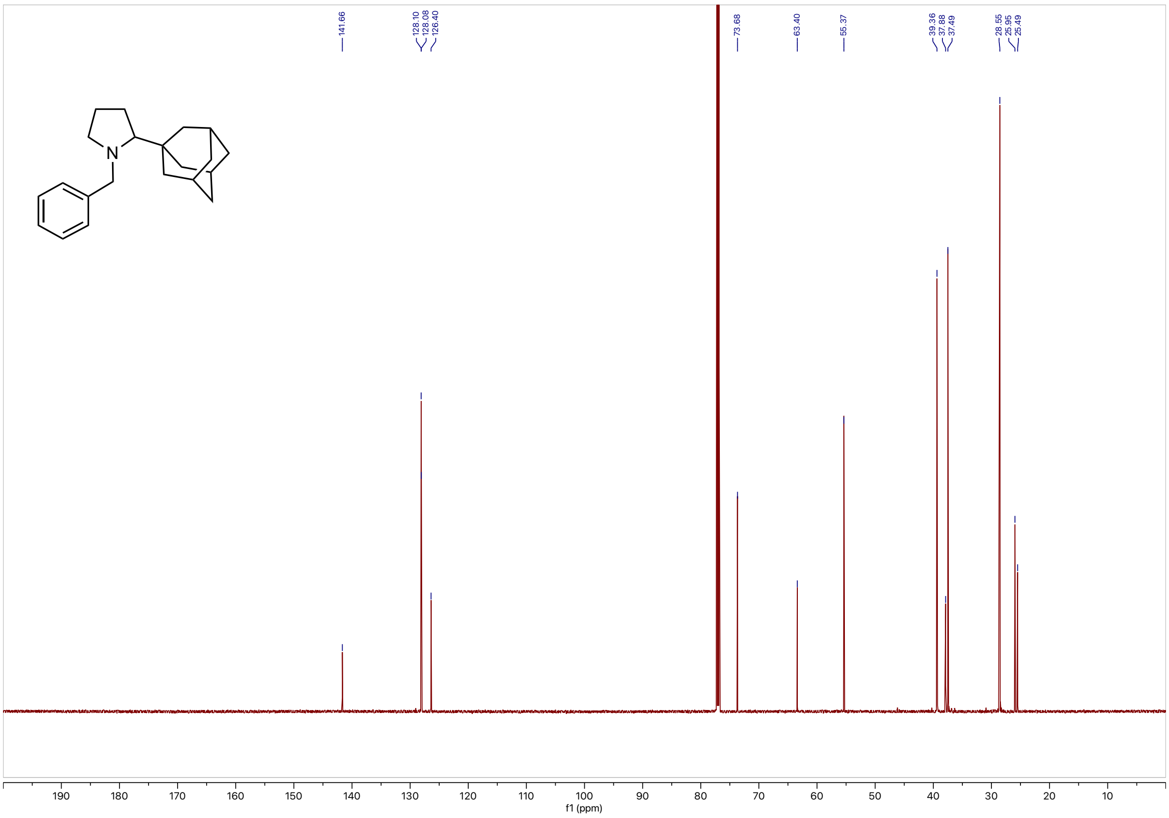

^{1}H NMR (700 MHz, $CDCl_3$) of 1-((adamantan-1-yl)(phenyl)methyl)pyrrolidine (**6u’**):

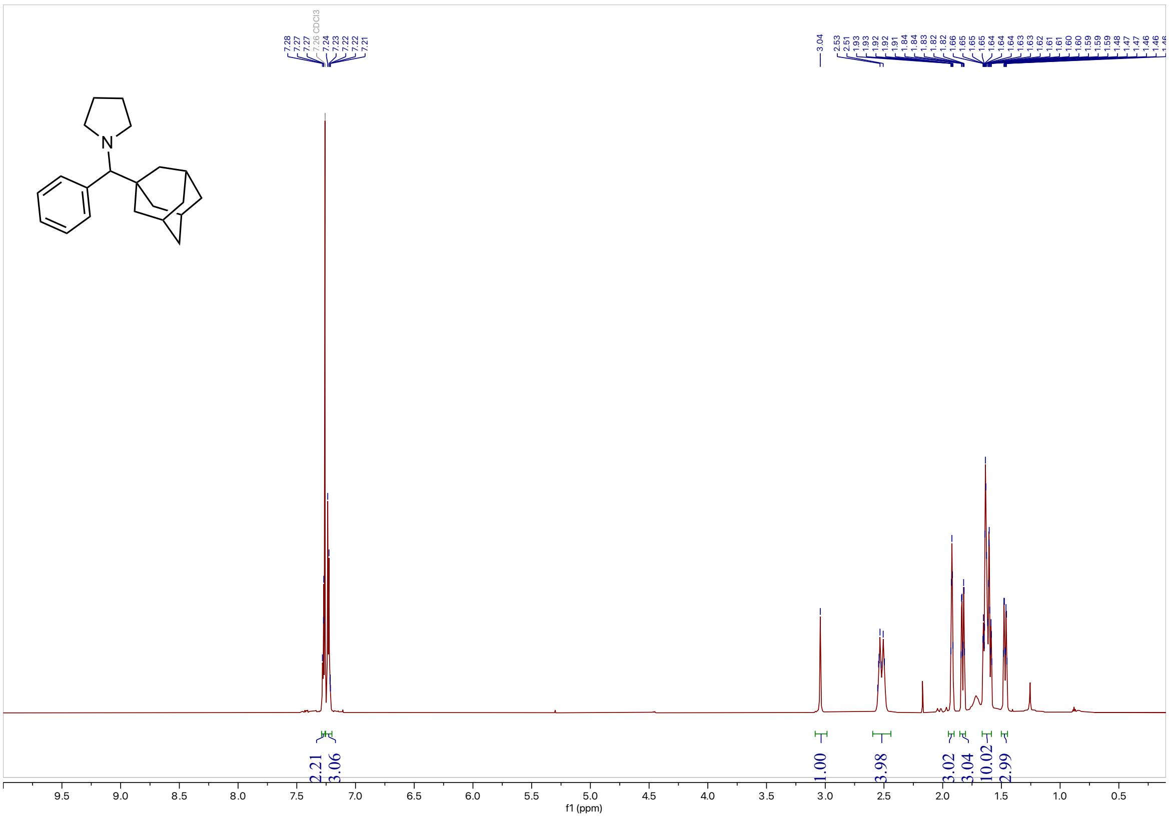

$^{13}C\{H\}$ NMR (176 MHz, $CDCl_3$) of 1-((adamantan-1-yl)(phenyl)methyl)pyrrolidine (**6u’**):

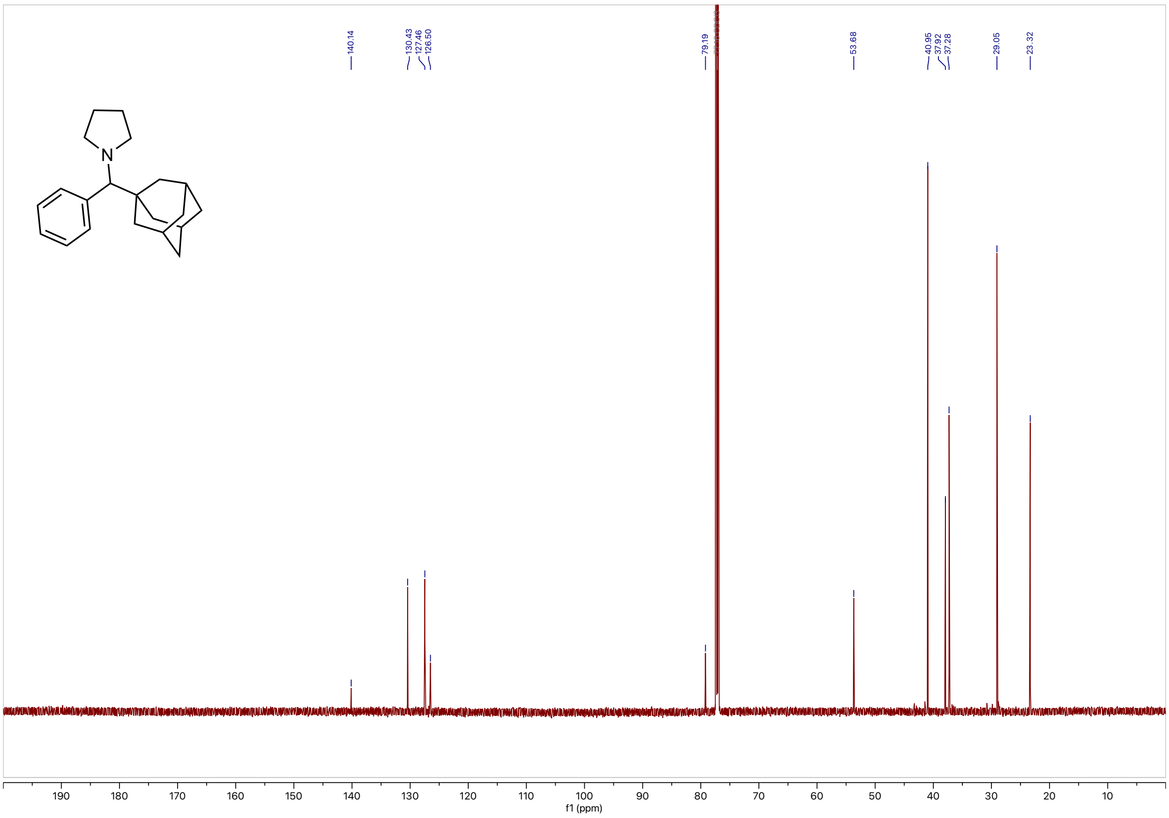

^{1}H NMR (700 MHz, $CDCl_3$) of 1-(4-phenylbenzyl)-2-ethyl-6-methylpiperidine (**11**):

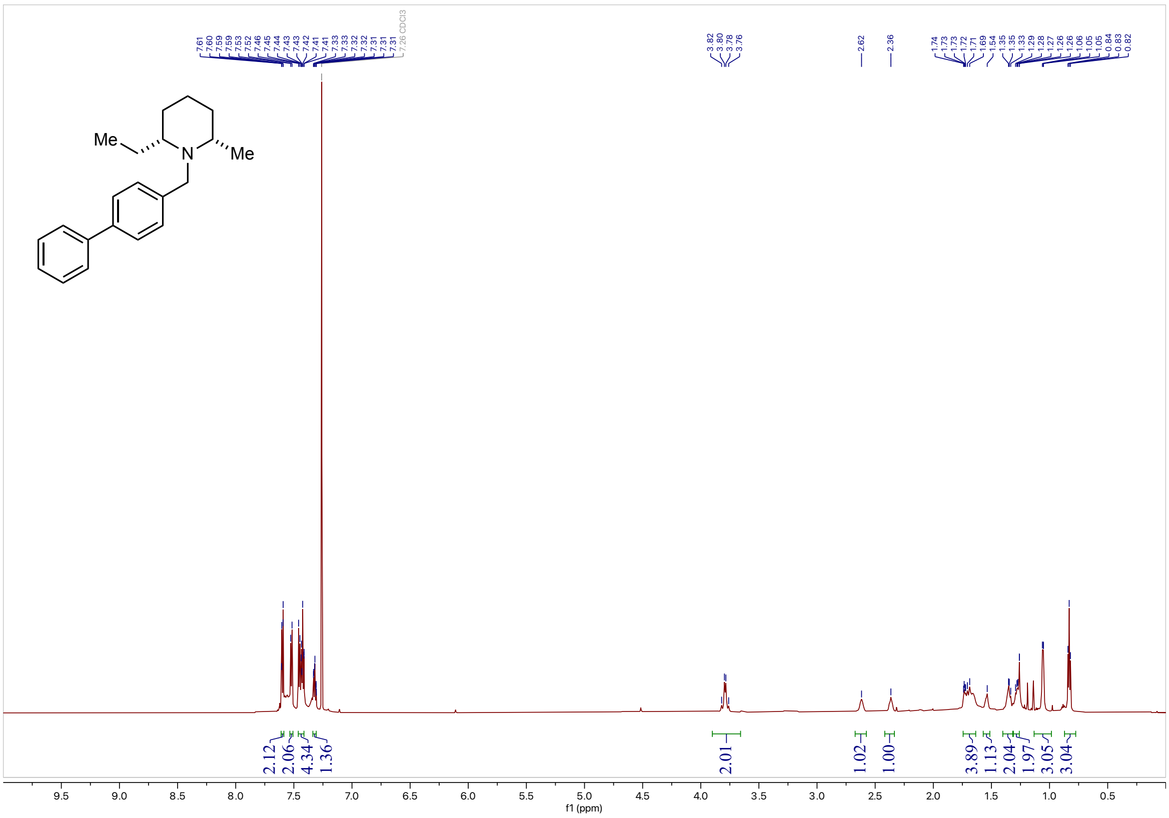

$^{13}C\{H\}$ NMR (176 MHz, $CDCl_3$) of 1-(4-phenylbenzyl)-2-ethyl-6-methylpiperidine (**11**):

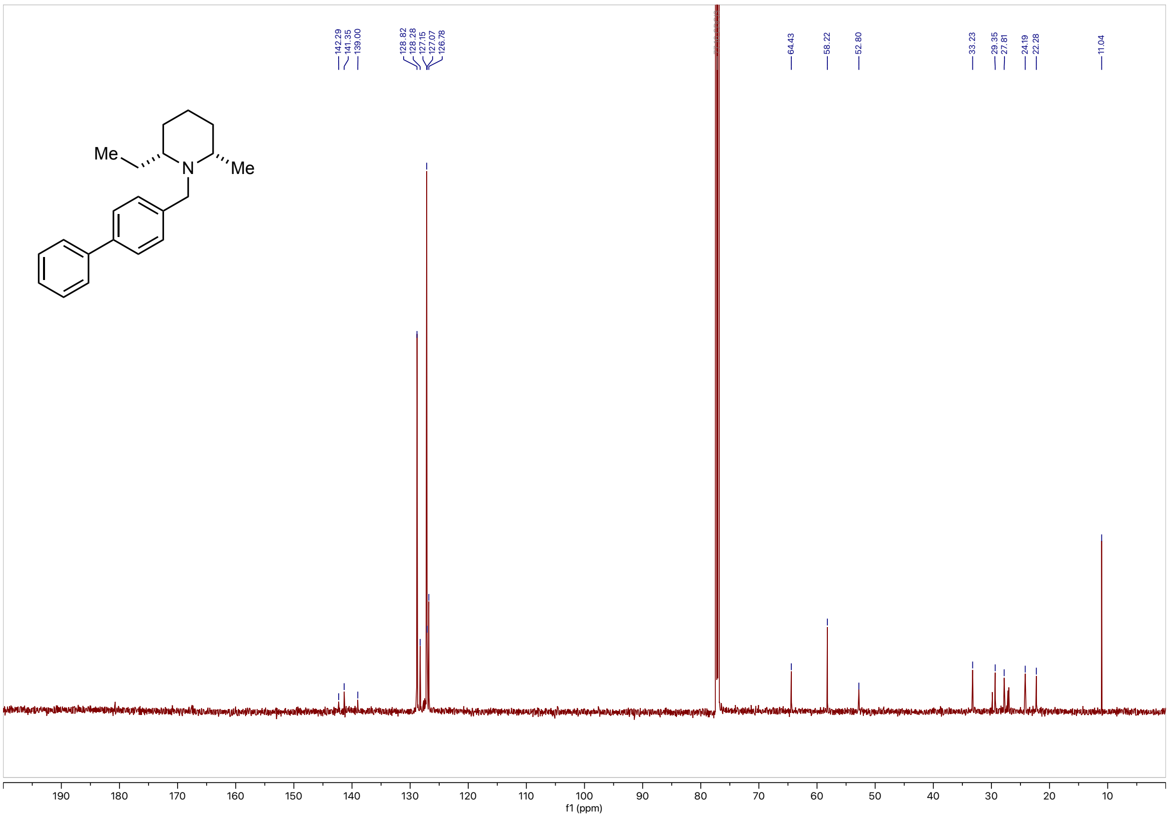

^{1}H NMR (500 MHz, $CDCl_3$) of (*2R,4S*)-1-benzyl-2-ethyl-4-methylpiperidine (**13**):

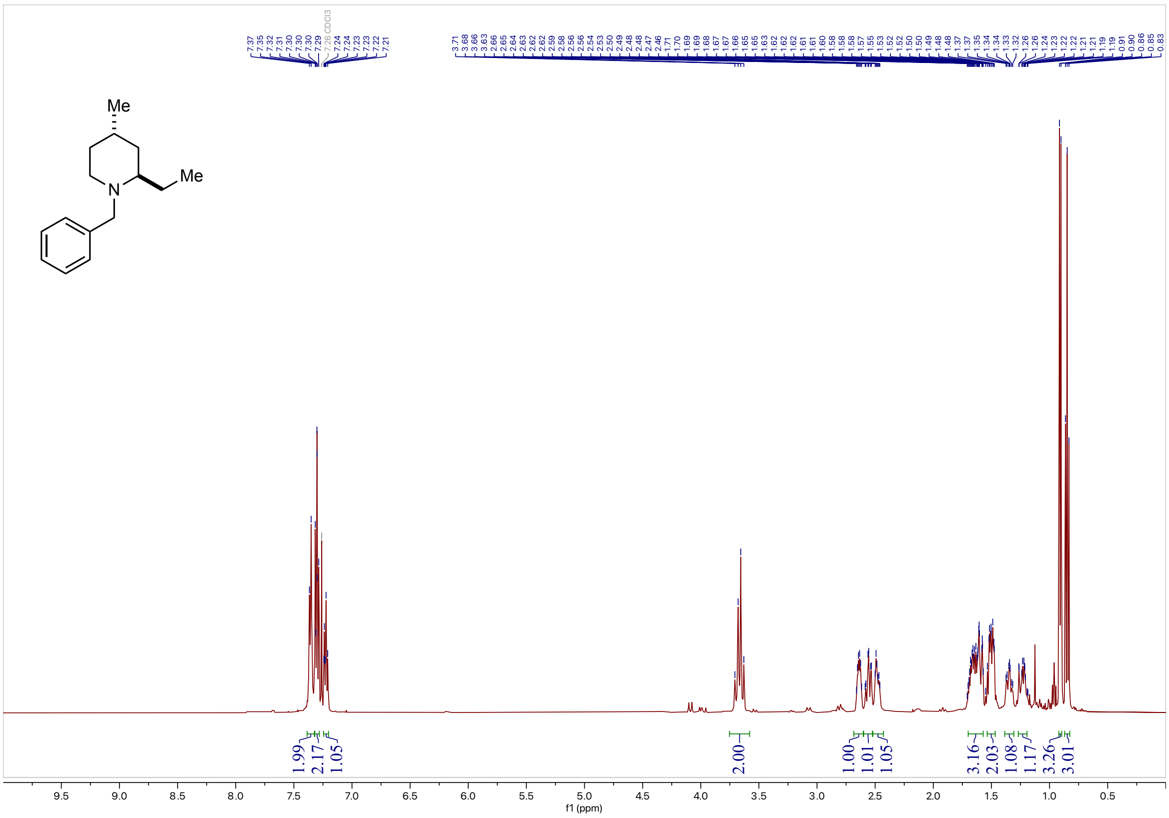

$^{13}C\{H\}$ NMR (126 MHz, $CDCl_3$) of (*2R,4S*)-1-benzyl-2-ethyl-4-methylpiperidine (**13**):

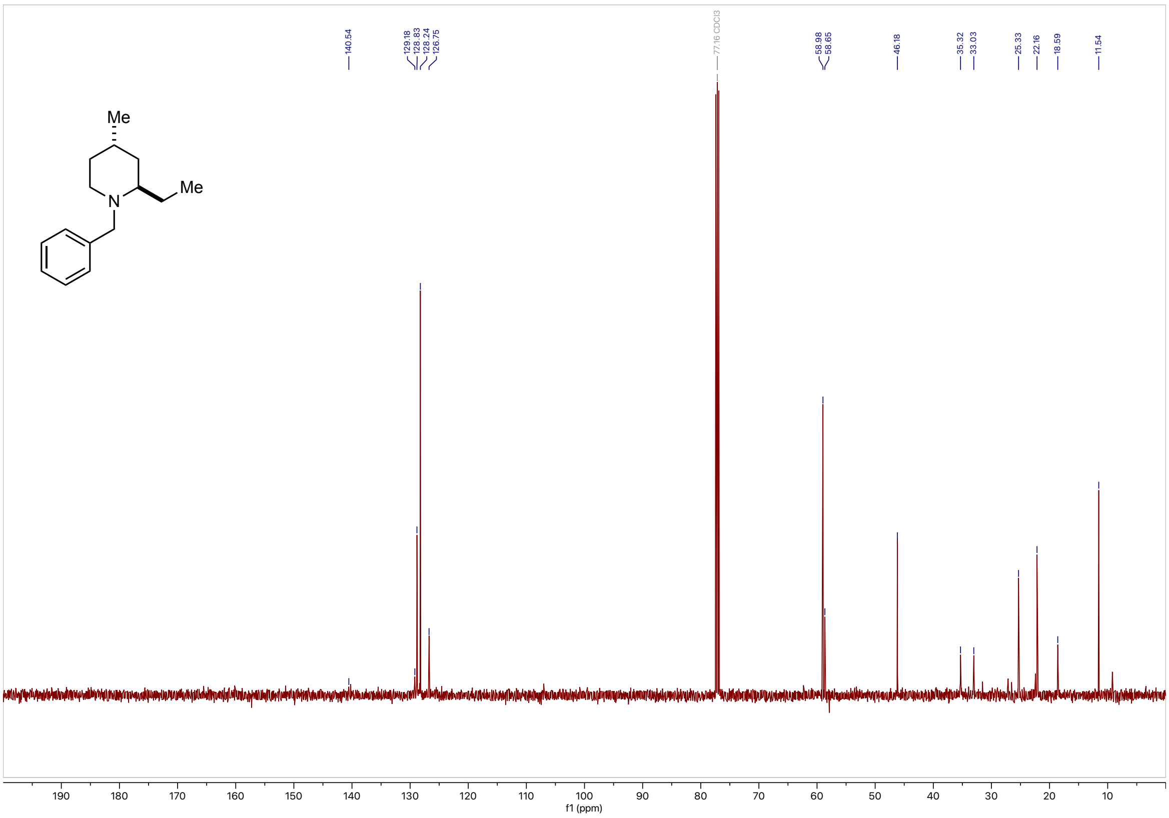

Selective NOSEY (500 MHz, $CDCl_3$) of (*2R,4S*)-1-benzyl-2-ethyl-4-methylpiperidine (**13**) Me-C**H** proton signal at 1.70 ppm:

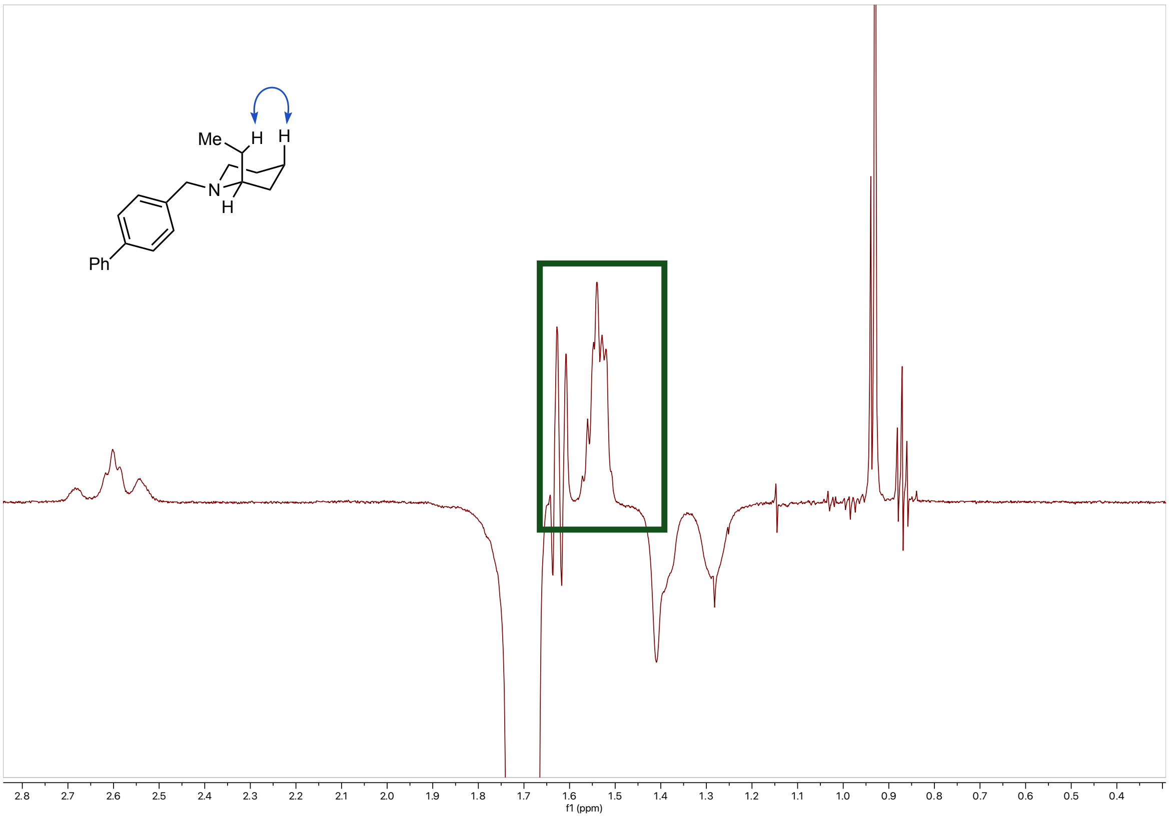

HSQC (500 MHz, CDCl3) of (*2R,4S*)-1-benzyl-2-ethyl-4-methylpiperidine (blue) overlapped with one of ethyl C**H**$_2$ proton signal (red) and a ring C**H**$_2$ proton at 1.54 ppm (green)

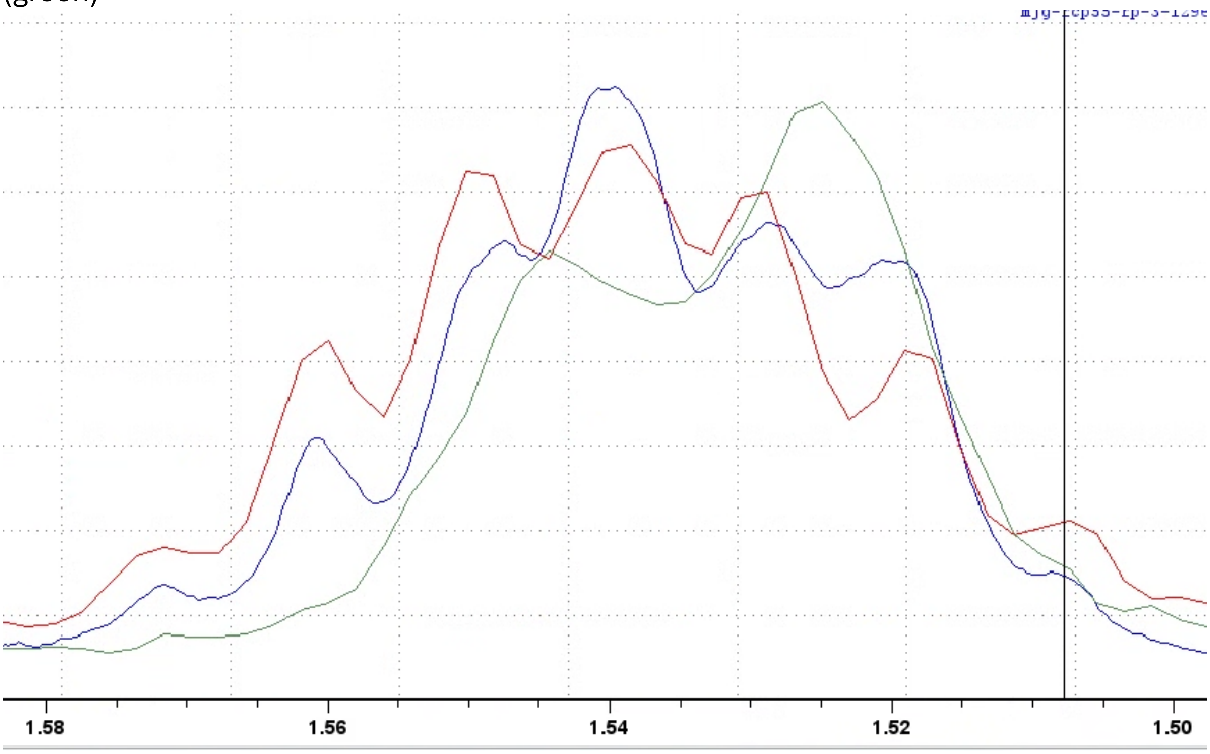

^{1}H NMR (400 MHz, $CDCl_3$) of 2-benzyl-1-(*tert*-butyl)-1,2,3,4-tetrahydroisoquinoline (**14a**):

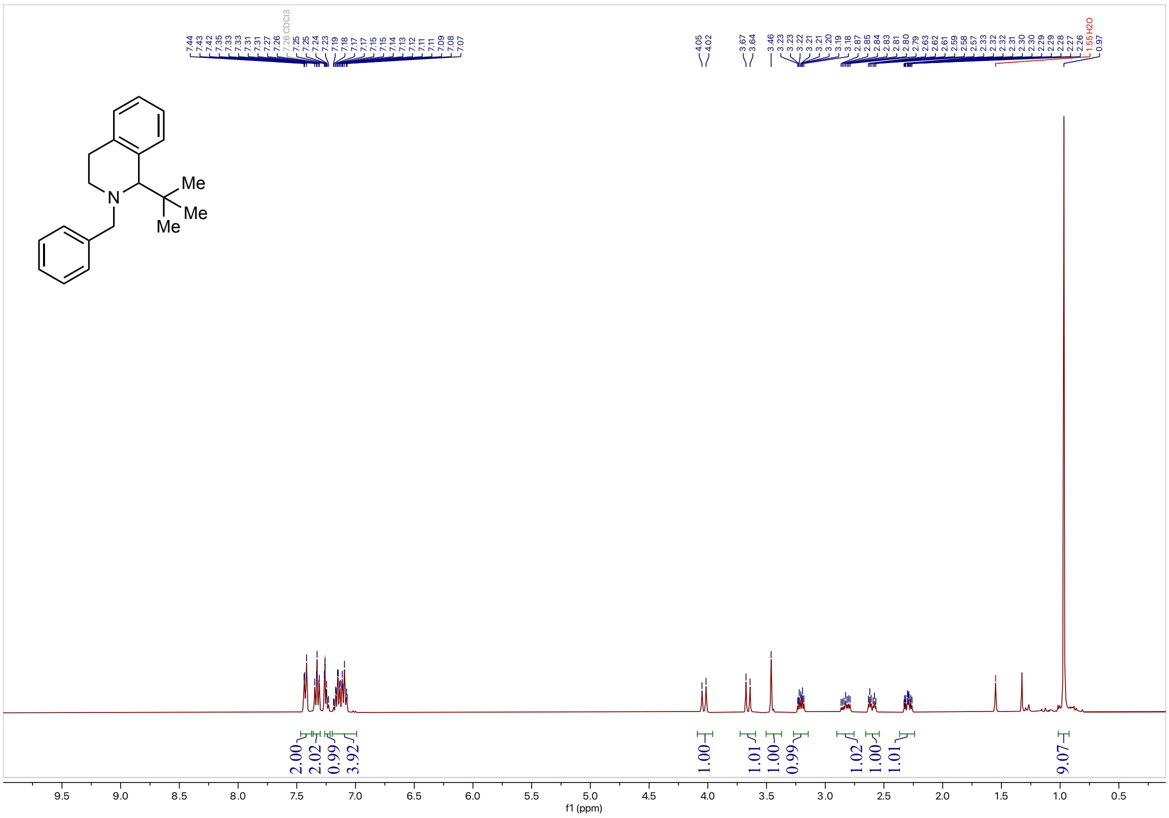

$^{13}C\{H\}$ NMR (176 MHz, $CDCl_3$) of 2-benzyl-1-(*tert*-butyl)-1,2,3,4-tetrahydroisoquinoline (**14a**):

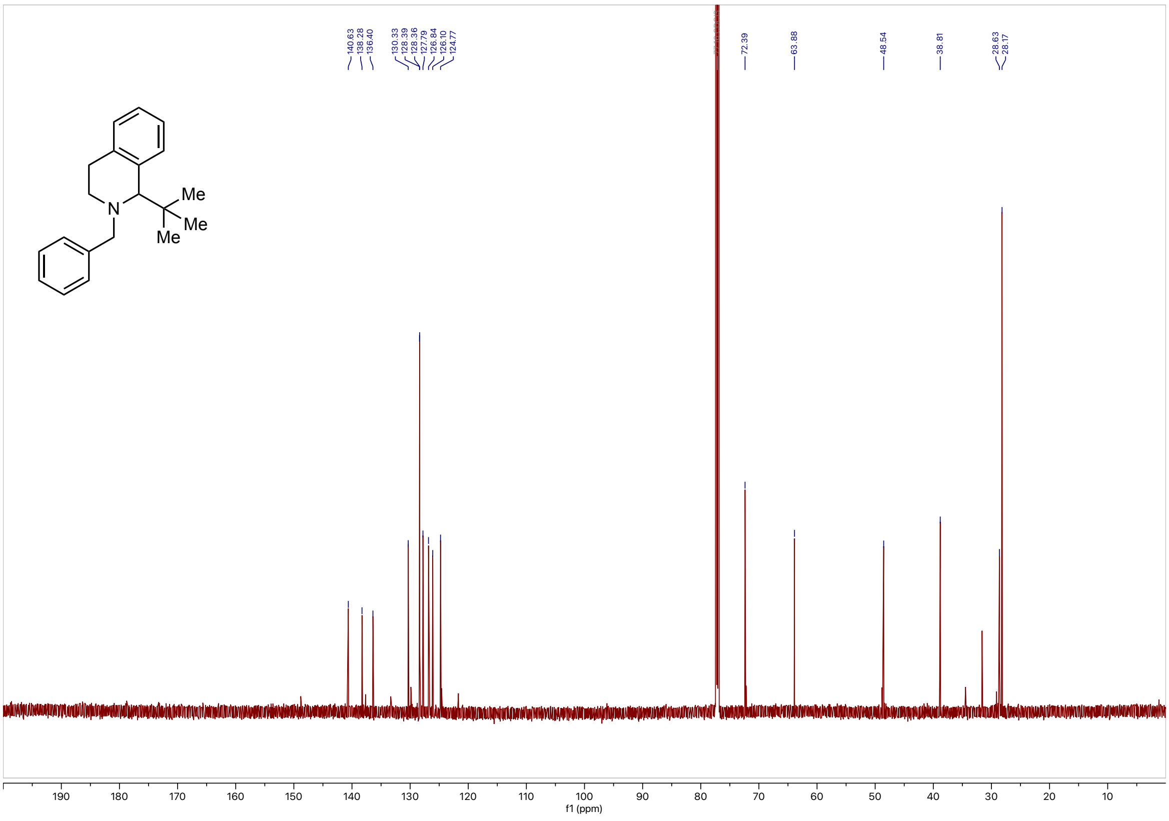

^{1}H NMR (700 MHz, $CDCl_3$) of 2-benzyl-1-ethyl-1,2,3,4-tetrahydroisoquinoline (**14b**):

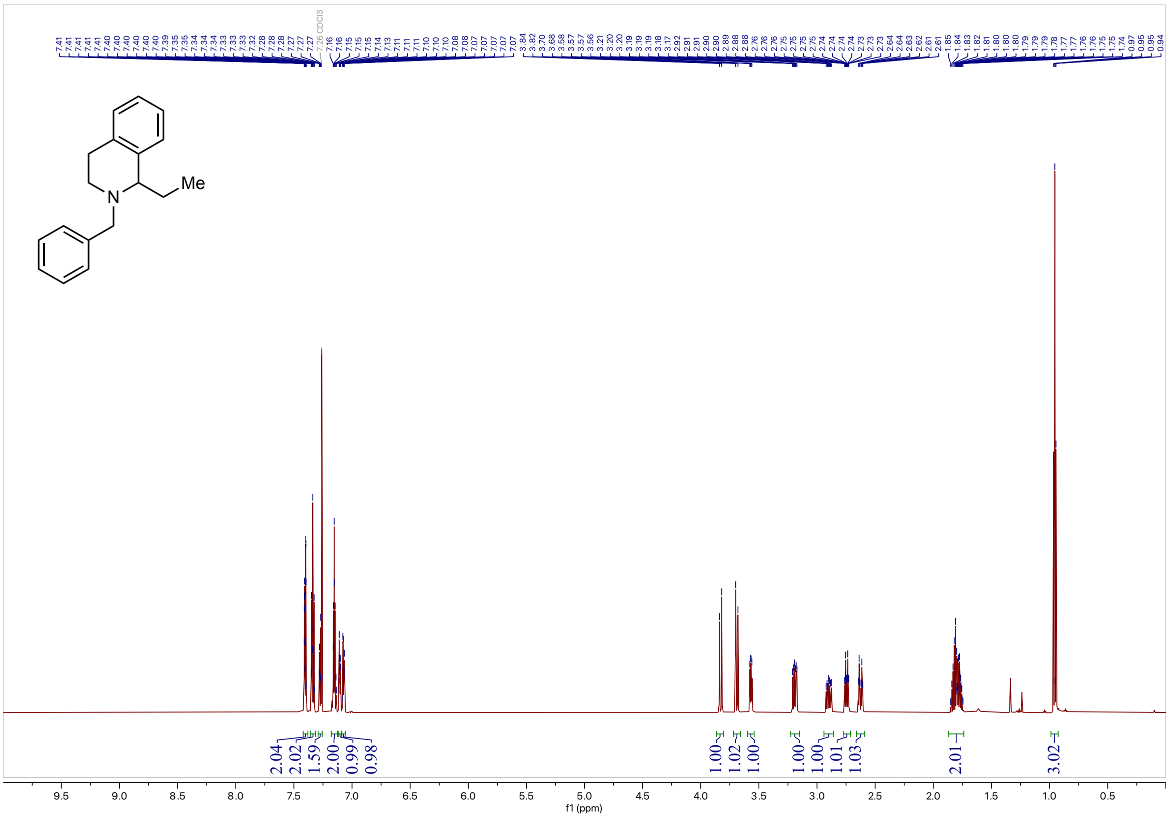

^{13}C{H} NMR (176 MHz, $CDCl_3$) of 2-benzyl-1-ethyl-1,2,3,4-tetrahydroisoquinoline (**14b**):

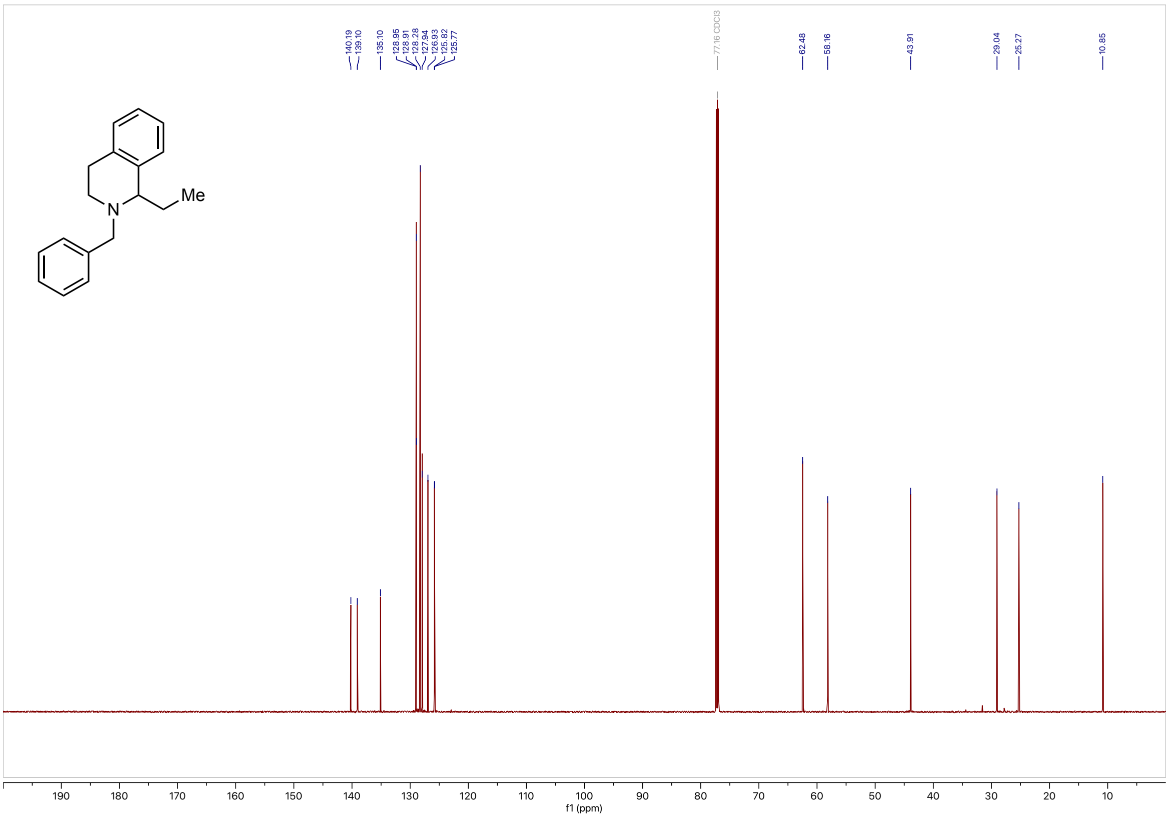

^1H NMR (700 MHz, $CDCl_3$) of 2-benzyl-6-(*tert*-butyl)-1,2,3,4-tetrahydroisoquinoline (**15a**):

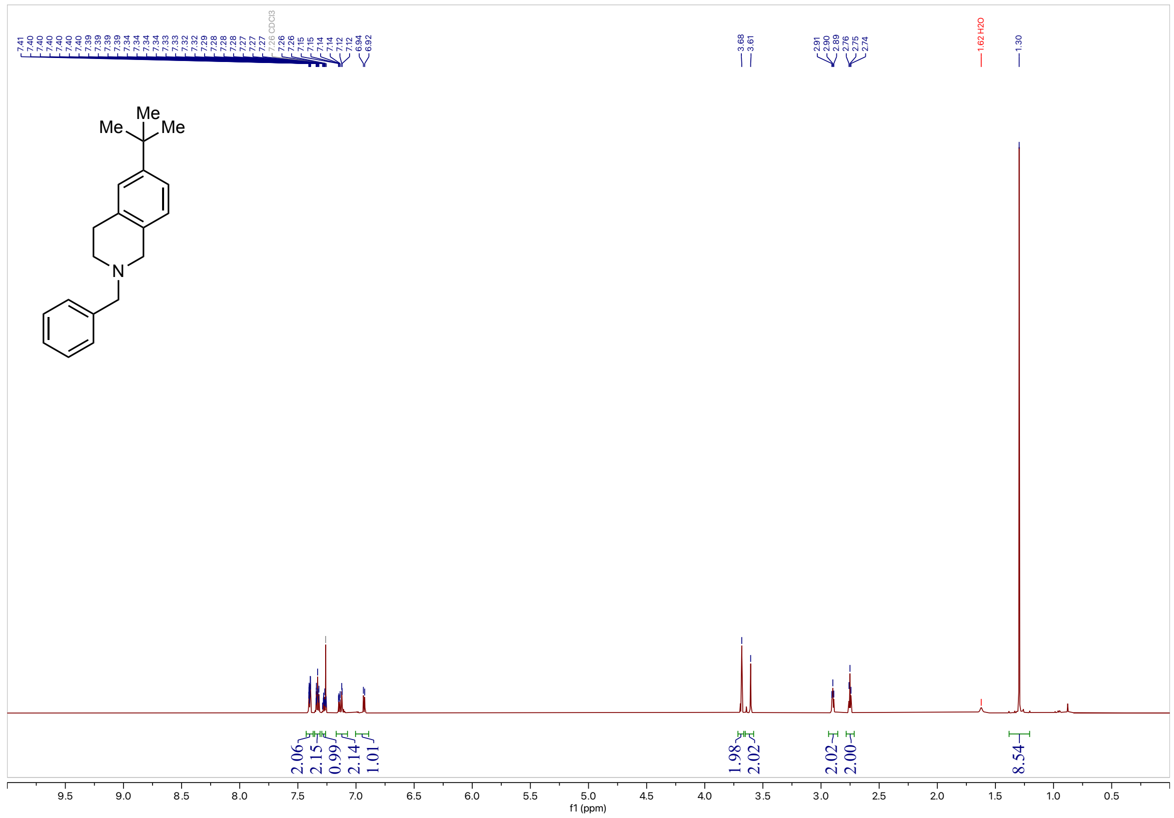

$^{13}C\{H\}$ NMR (126 MHz, $CDCl_3$) of 2-benzyl-6-(*tert*-butyl)-1,2,3,4-tetrahydroisoquinoline (**15a**):

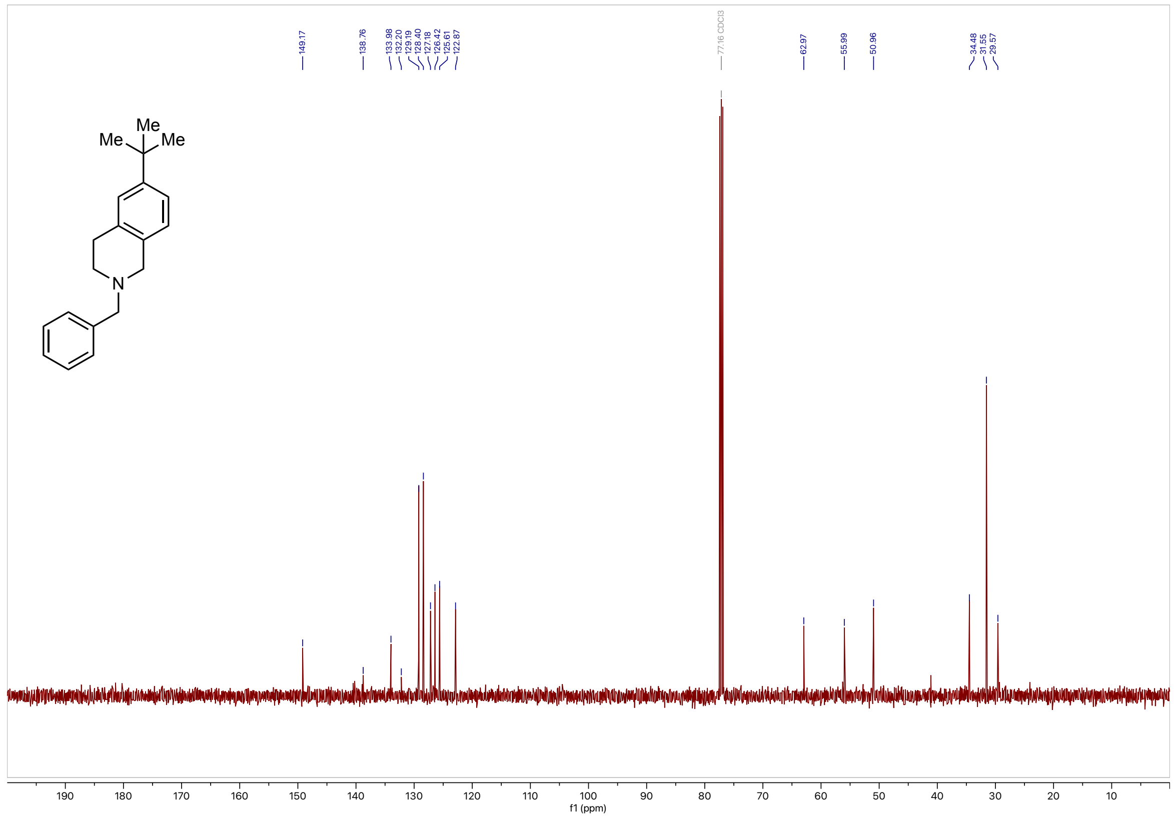

^{1}H NMR (500 MHz, $CDCl_3$) of 1-(4-phenylbenzyl)-2-methylpiperidine (**8**):

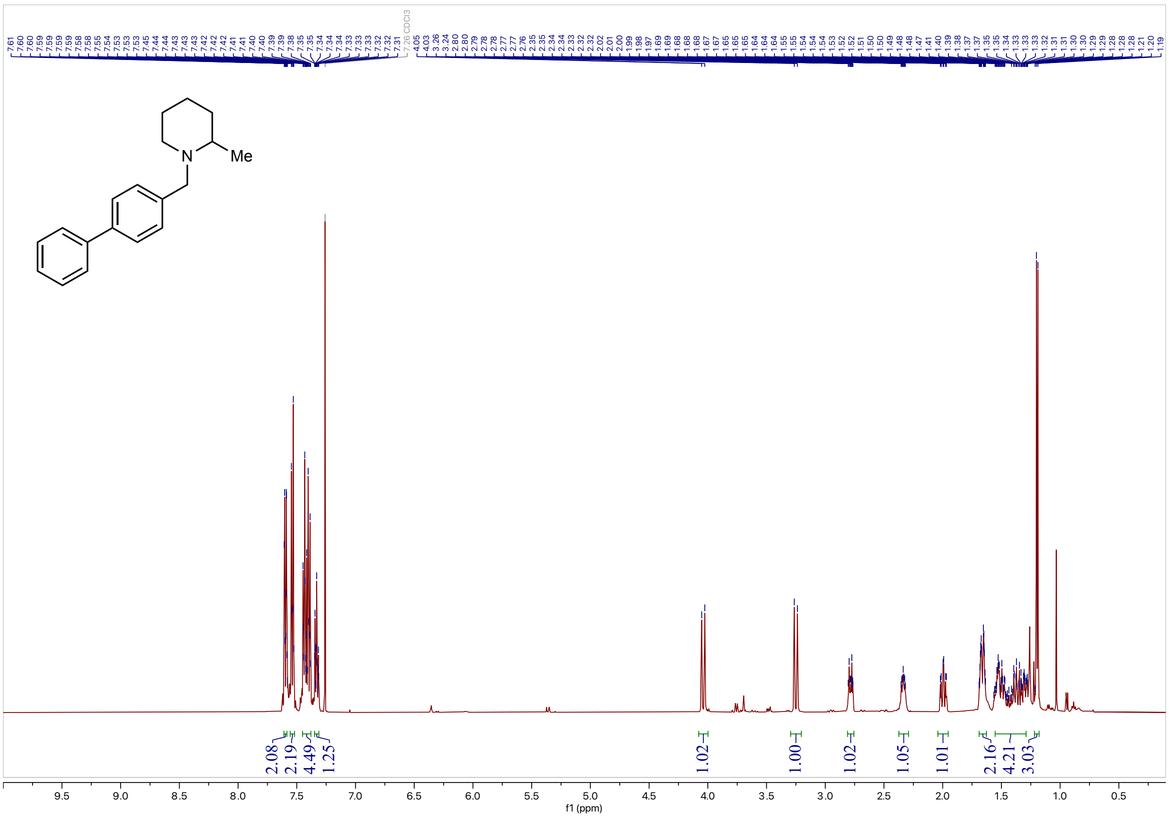

$^{13}C\{H\}$ NMR (176 MHz, $CDCl_3$) of 1-(4-phenylbenzyl)-2-methylpiperidine (**8**):

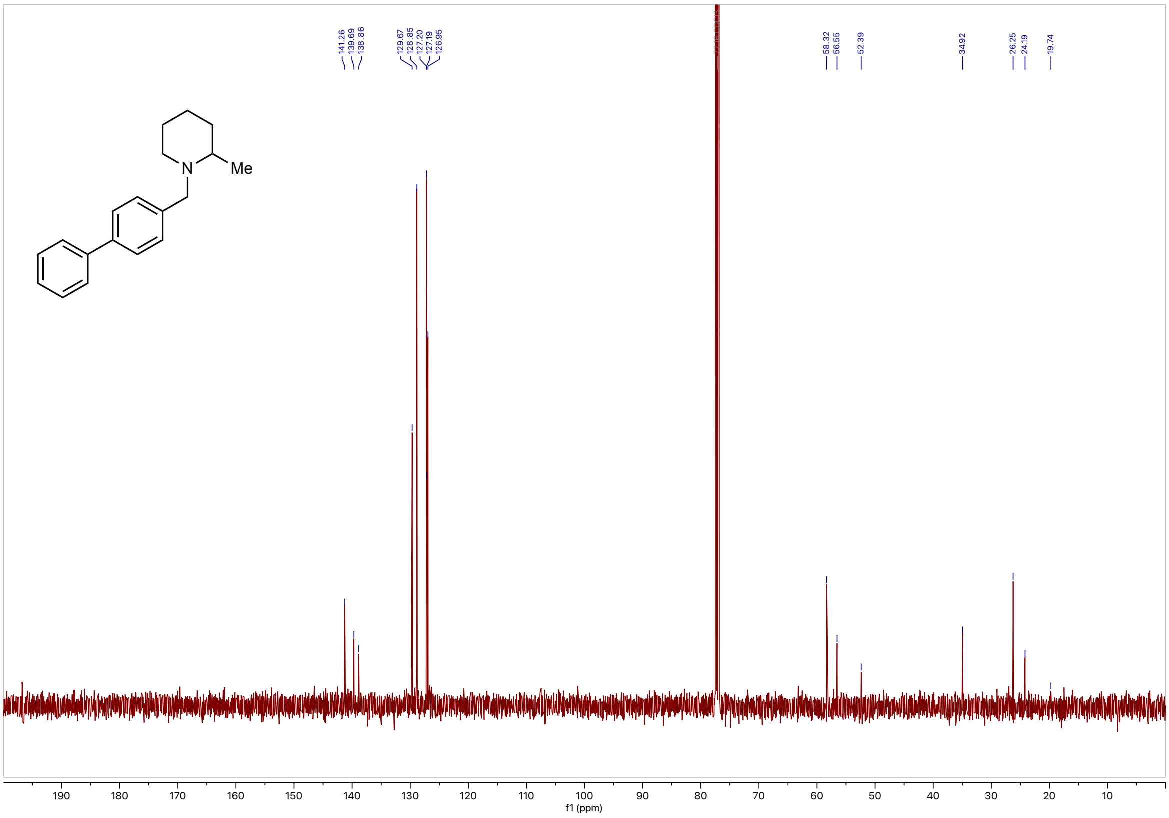

^{1}H NMR (500 MHz, $CDCl_3$) of 1-(4-phenylbenzyl)-2-(methyl-d_3)piperidine (***d*₃-8**):

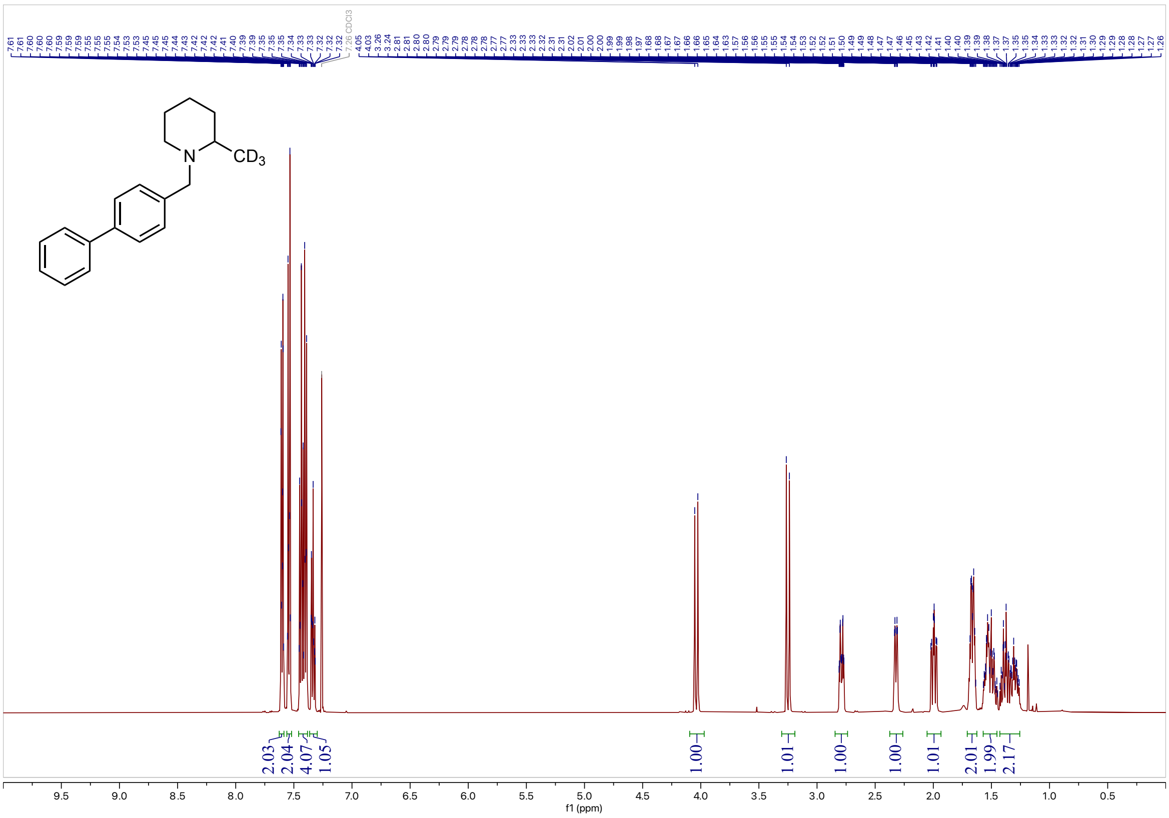

^{13}C{H} NMR (126 MHz, $CDCl_3$) of 1-(4-phenylbenzyl)-2-(methyl-d_3)piperidine (***d*₃-8**):

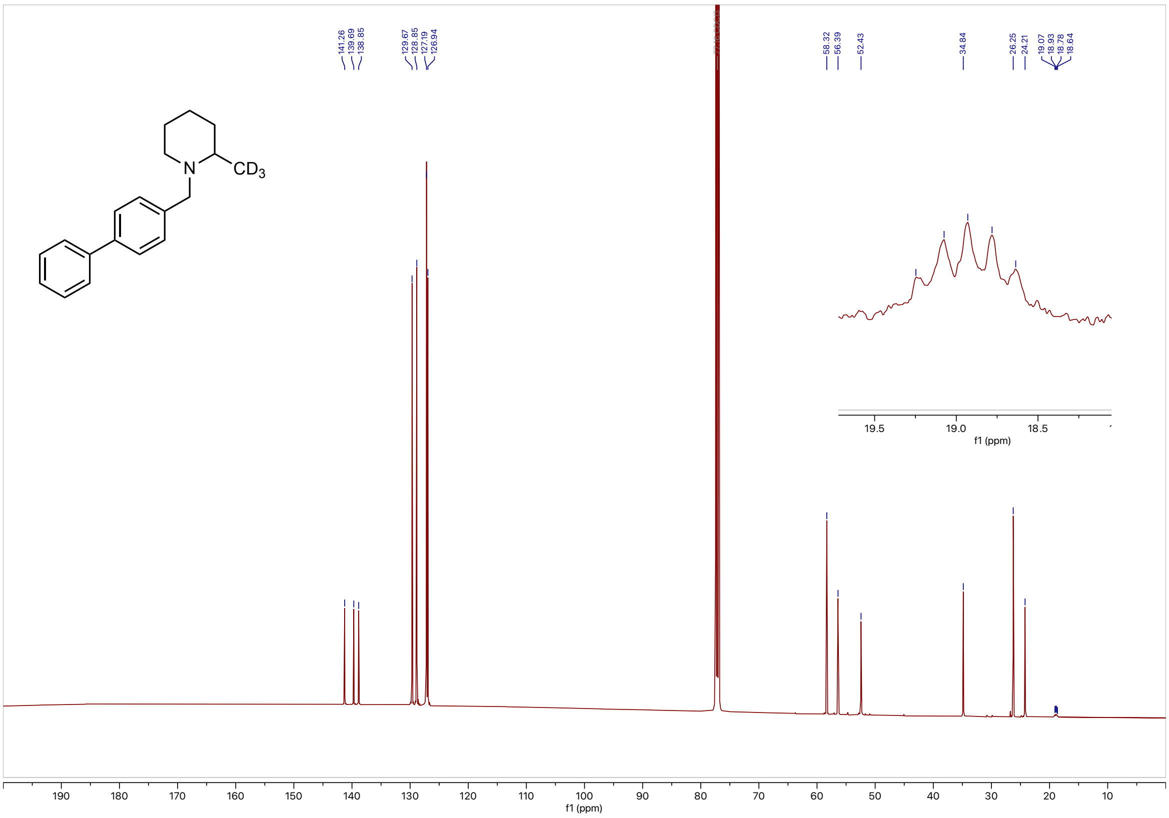

^{1}H NMR (500 MHz, $CDCl_3$) of 1-(4-phenylbenzyl)-2-methyl-2-ethylpiperidine (**12**):

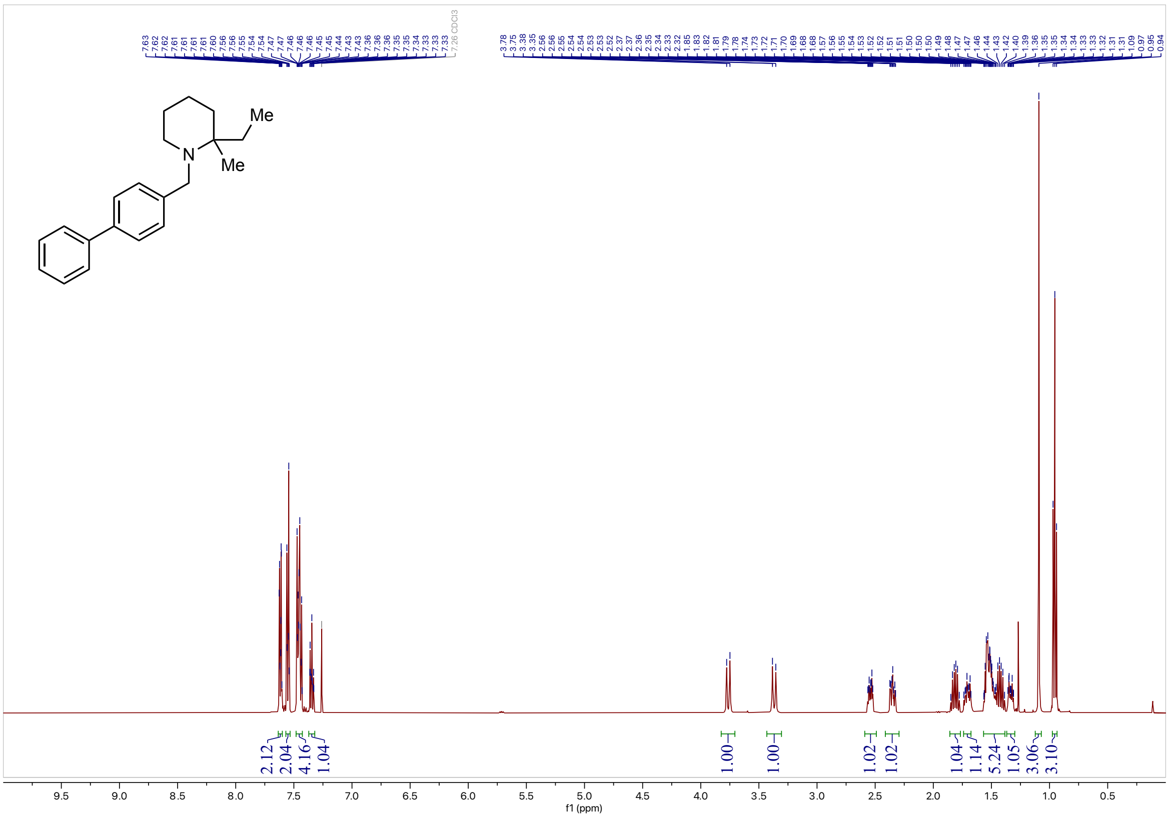

^{13}C{H} NMR (126 MHz, $CDCl_3$) of 1-(4-phenylbenzyl)-2-methyl-2-ethylpiperidine (**12**):

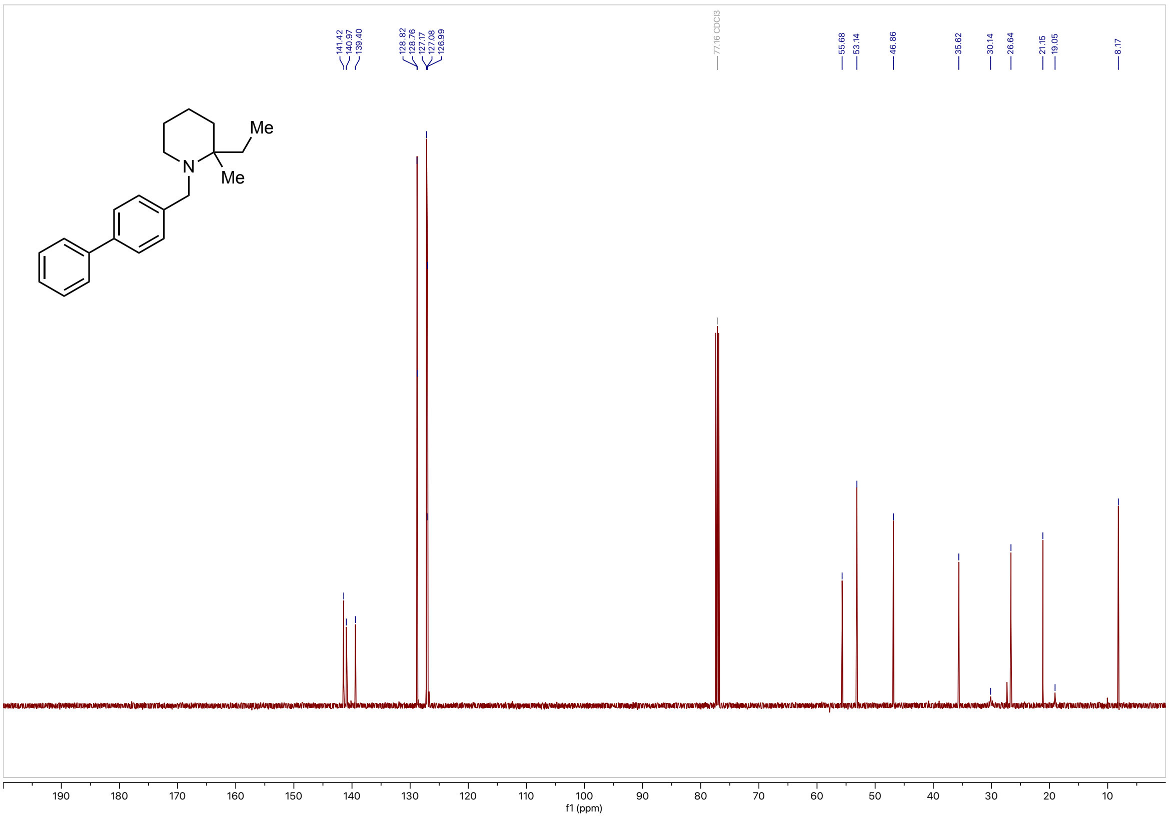

^{1}H NMR (700 MHz, $CDCl_3$) of (2*R*,6*S*)-1-(4-phenylbenzyl)-2-ethyl-6-methylpiperidine (**12’**):

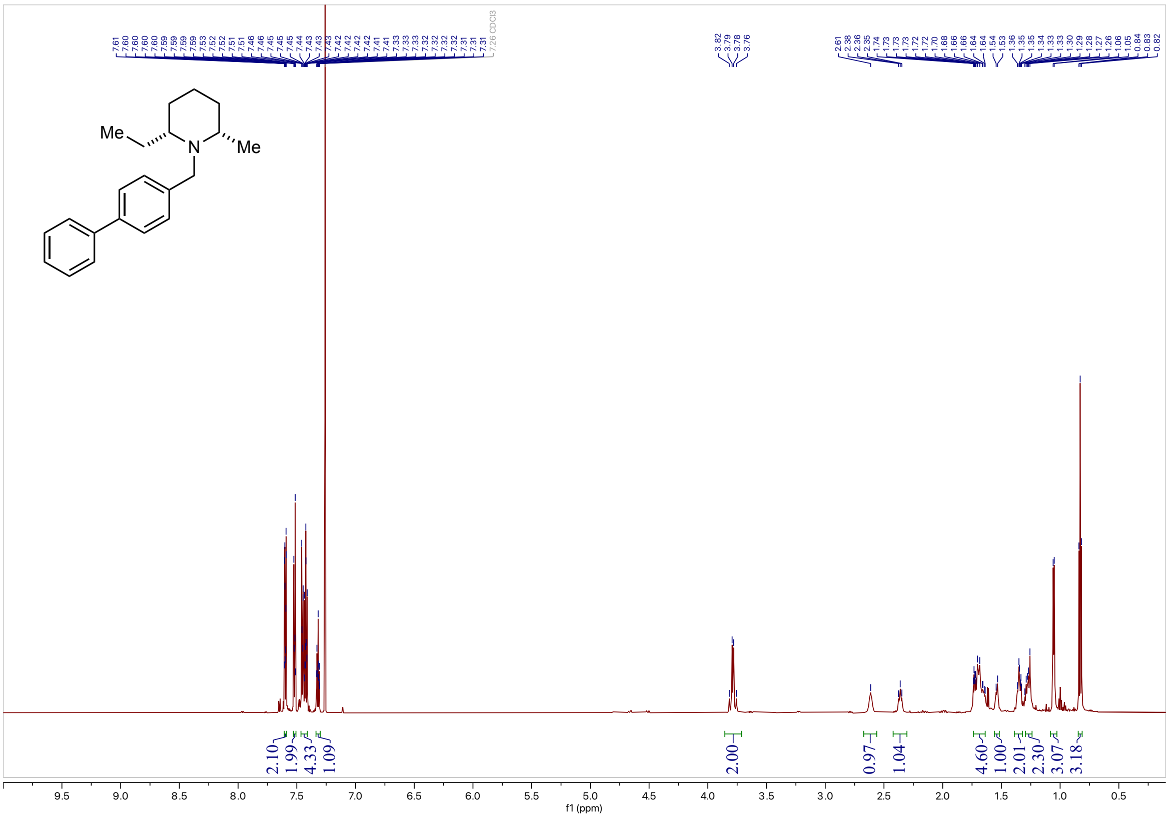

^{13}C{H} NMR (176 MHz, $CDCl_3$) of (2*R*,6*S*)-1-(4-phenylbenzyl)-2-ethyl-6-methylpiperidine (**12’**):

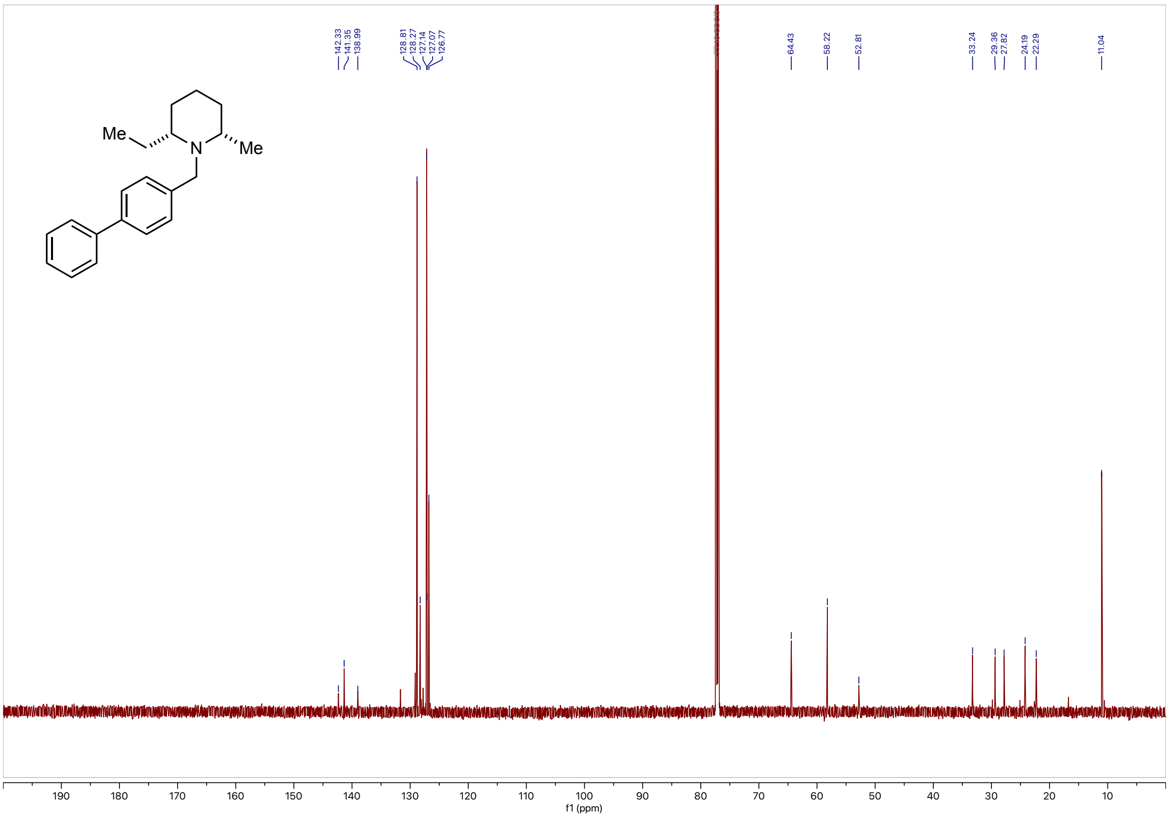

NOSEY (500 MHz, $CDCl_3$) of (*2R,6S*)-1-(4-phenylbenzyl)-2-ethyl-6-ethylpiperidine (**12'**):

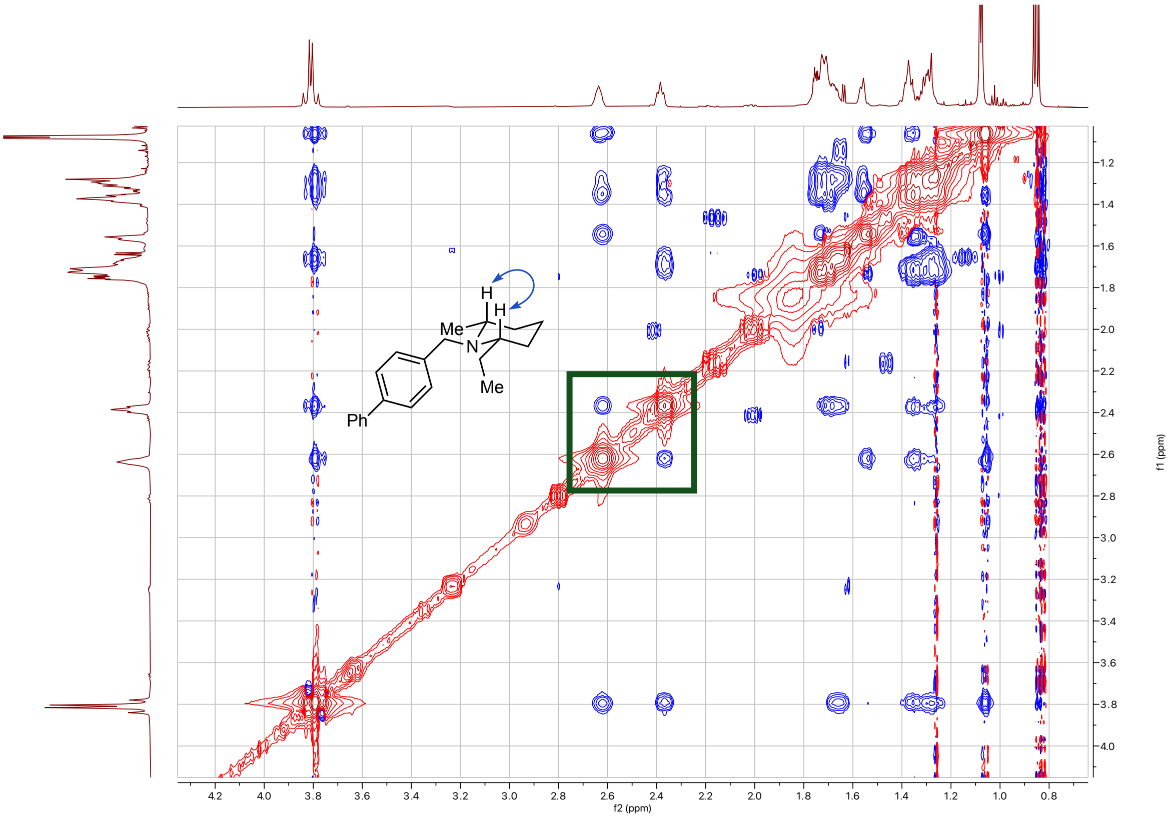

^{1}H NMR (500 MHz, $CDCl_3$) of (*2S,4S*)-1-benzyl-2-ethyl-4-methylpiperidine (**13'**):

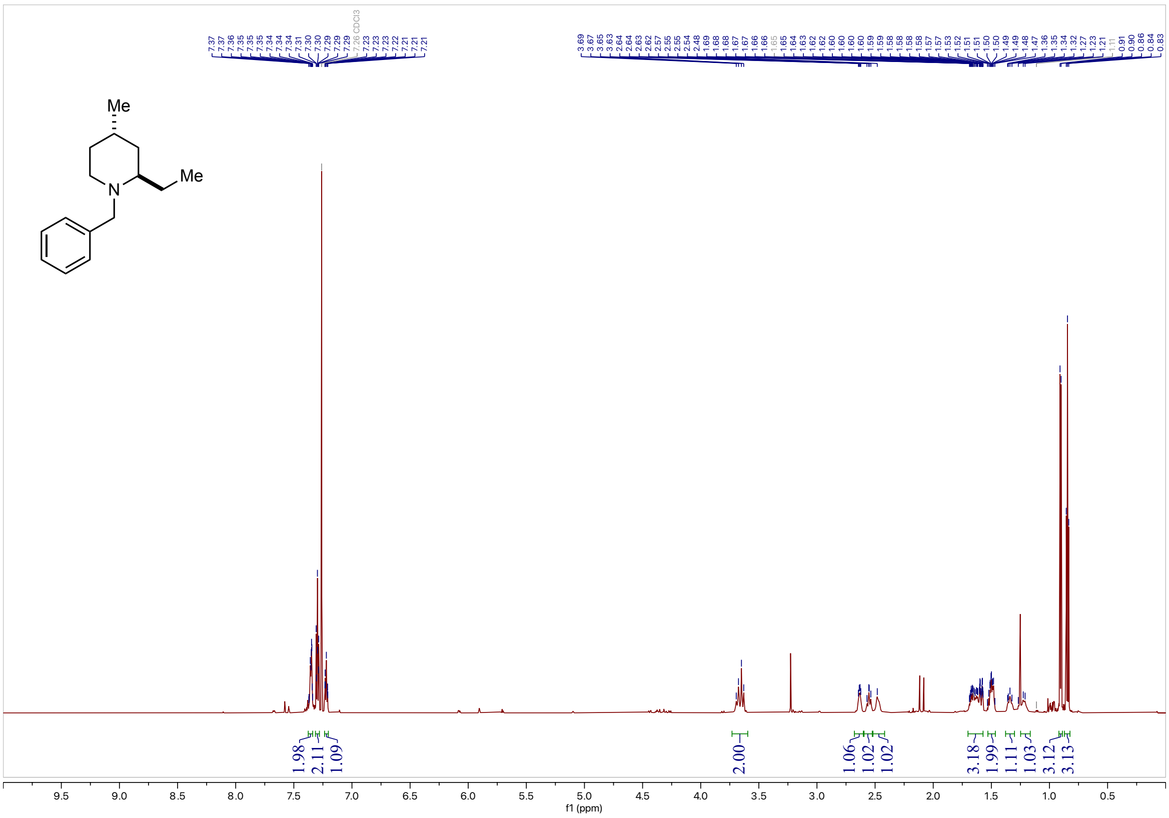

^{13}C{H} NMR (126 MHz, $CDCl_3$) of (*2S,4S*)-1-benzyl-2-ethyl-4-methylpiperidine (**13'**):

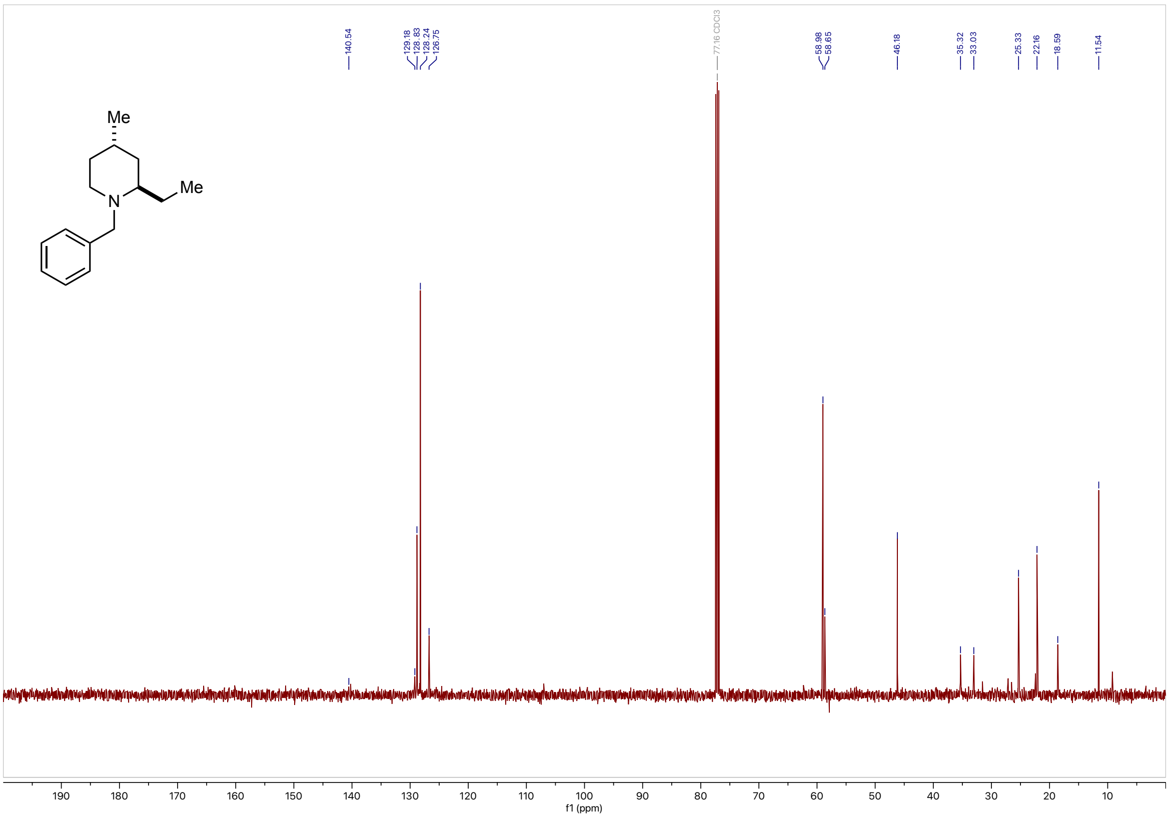

^{1}H NMR (700 MHz, $CDCl_3$) of 2-benzyl-1-ethyl-1,2,3,4-tetrahydroisoquinoline (**14c**):

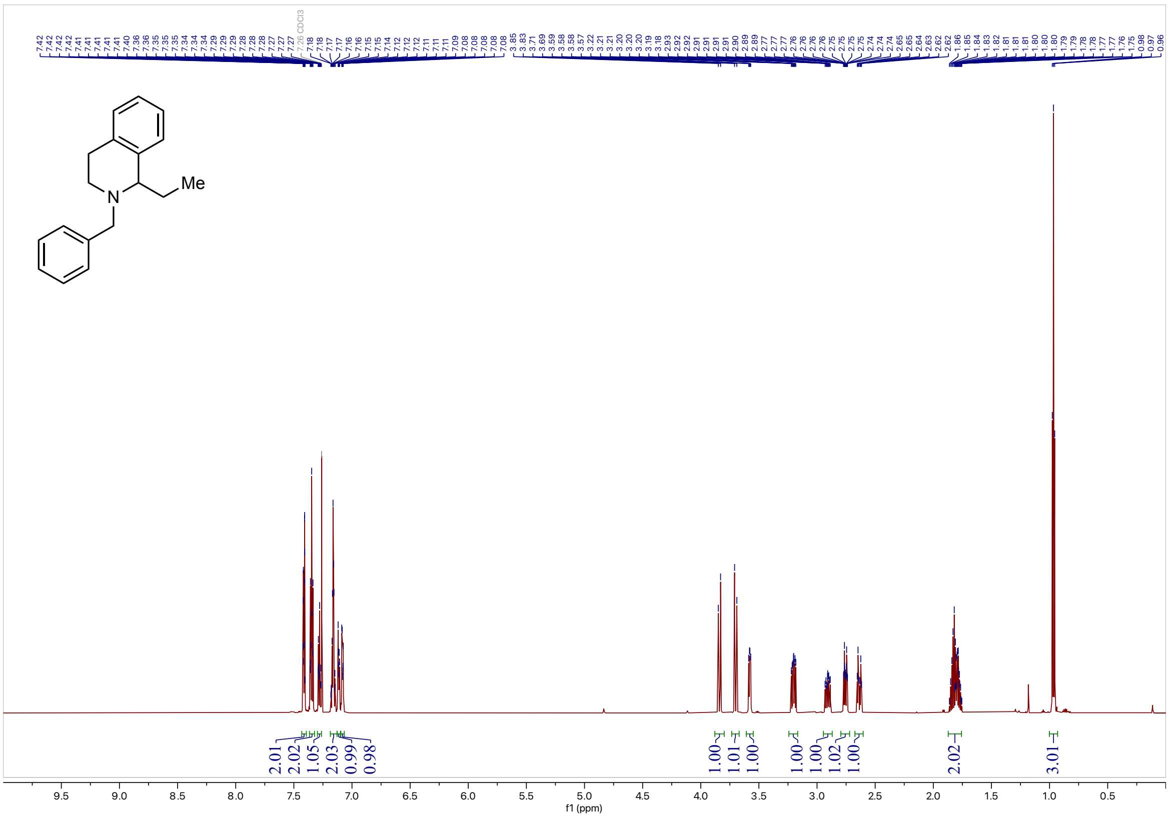

^{13}C{H} NMR (176 MHz, $CDCl_3$) of 2-benzyl-1-ethyl-1,2,3,4-tetrahydroisoquinoline (**14c**):

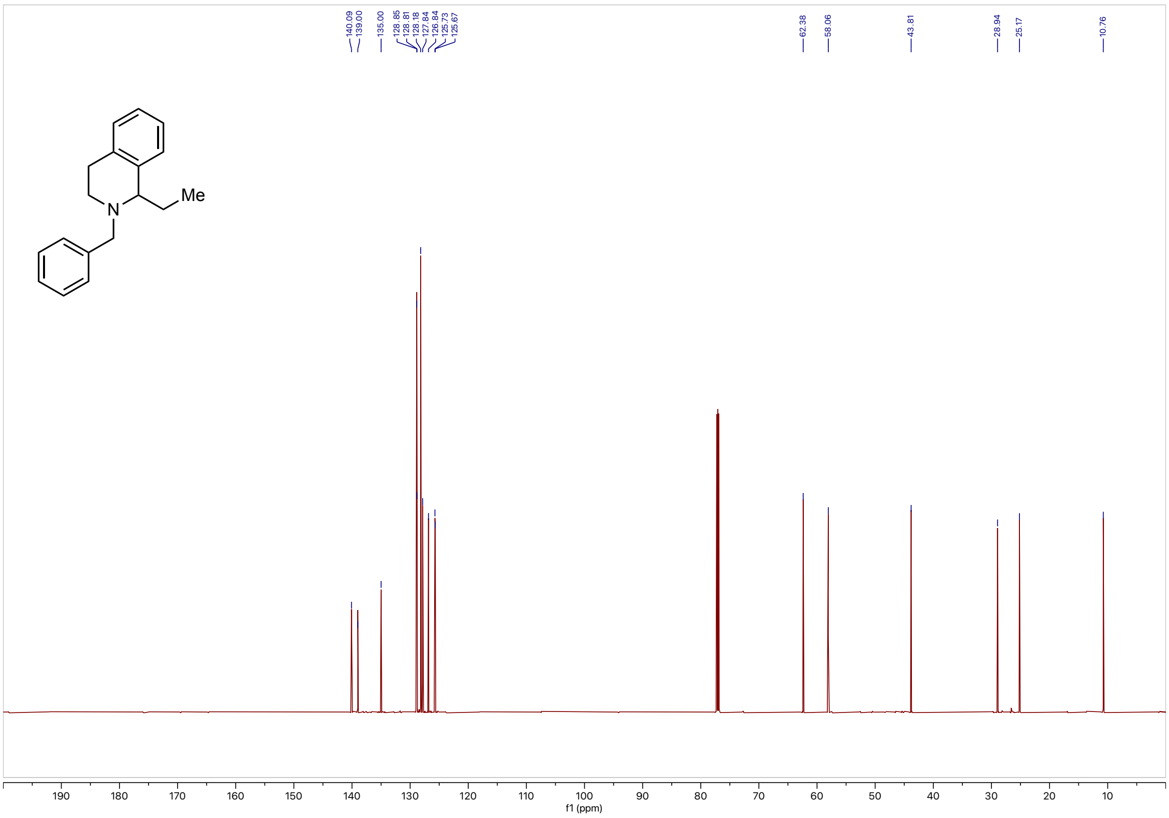

^{1}H NMR (500 MHz, $CDCl_3$) of 1-(4-phenylbenzyl)-2-(trifluoromethyl)piperidine (**9a**):

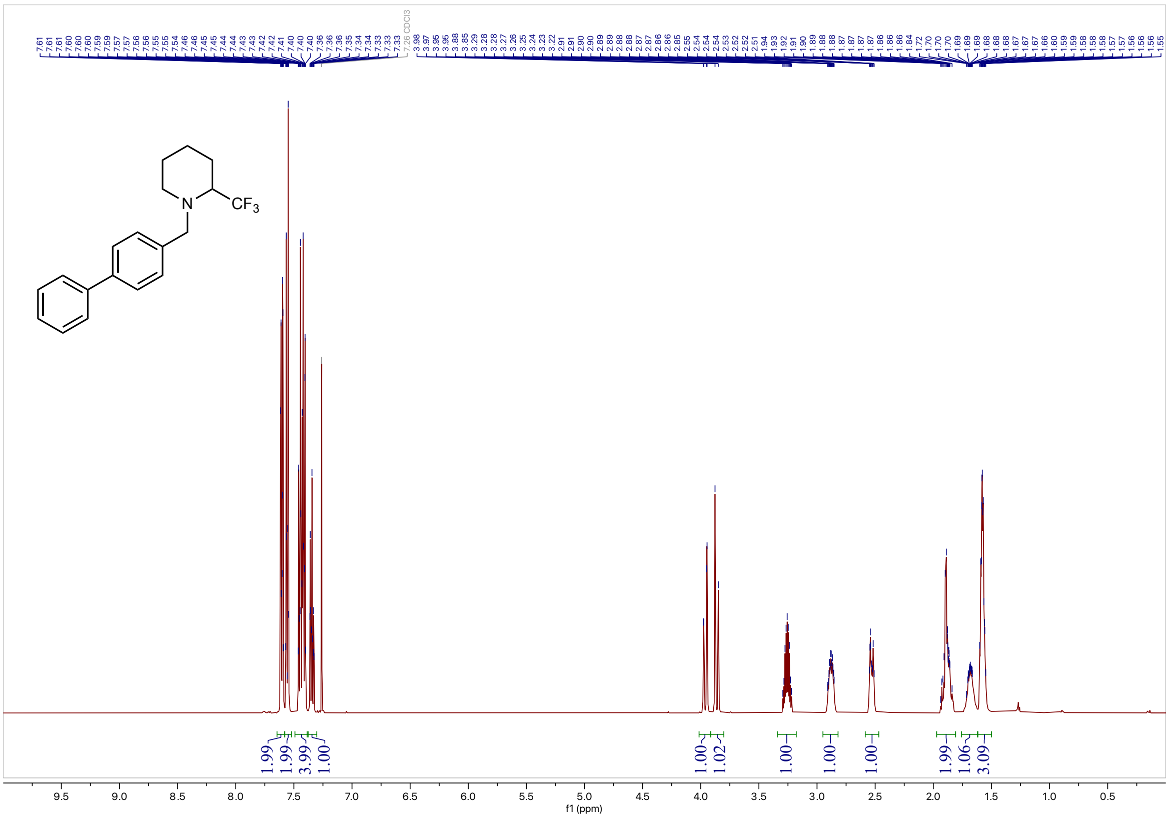

$^{13}C\{H\}$ NMR (176 MHz, $CDCl_3$) of 1-(4-phenylbenzyl)-2-(trifluoromethyl)piperidine (**9a**):

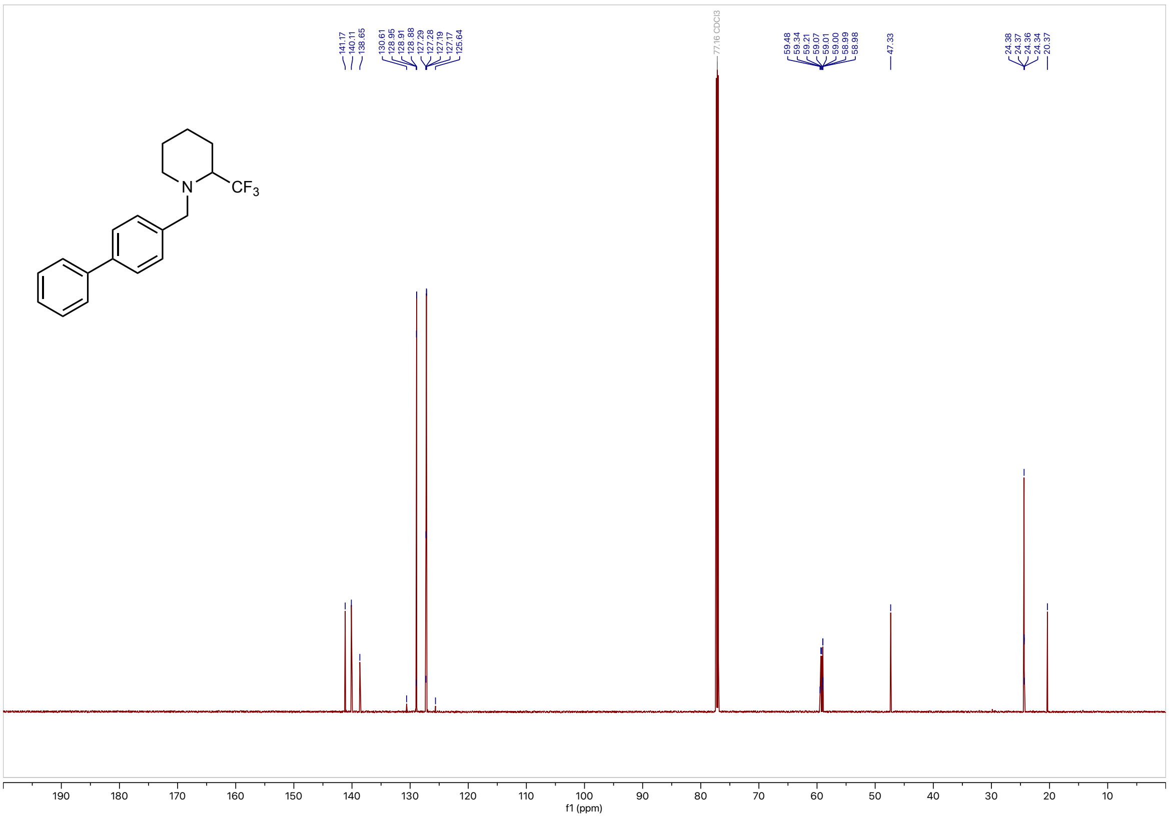

$^{19}F\{H\}$ NMR (471 MHz, $CDCl_3$) of 1-(4-phenylbenzyl)-2-(trifluoromethyl)piperidine (**9a**):

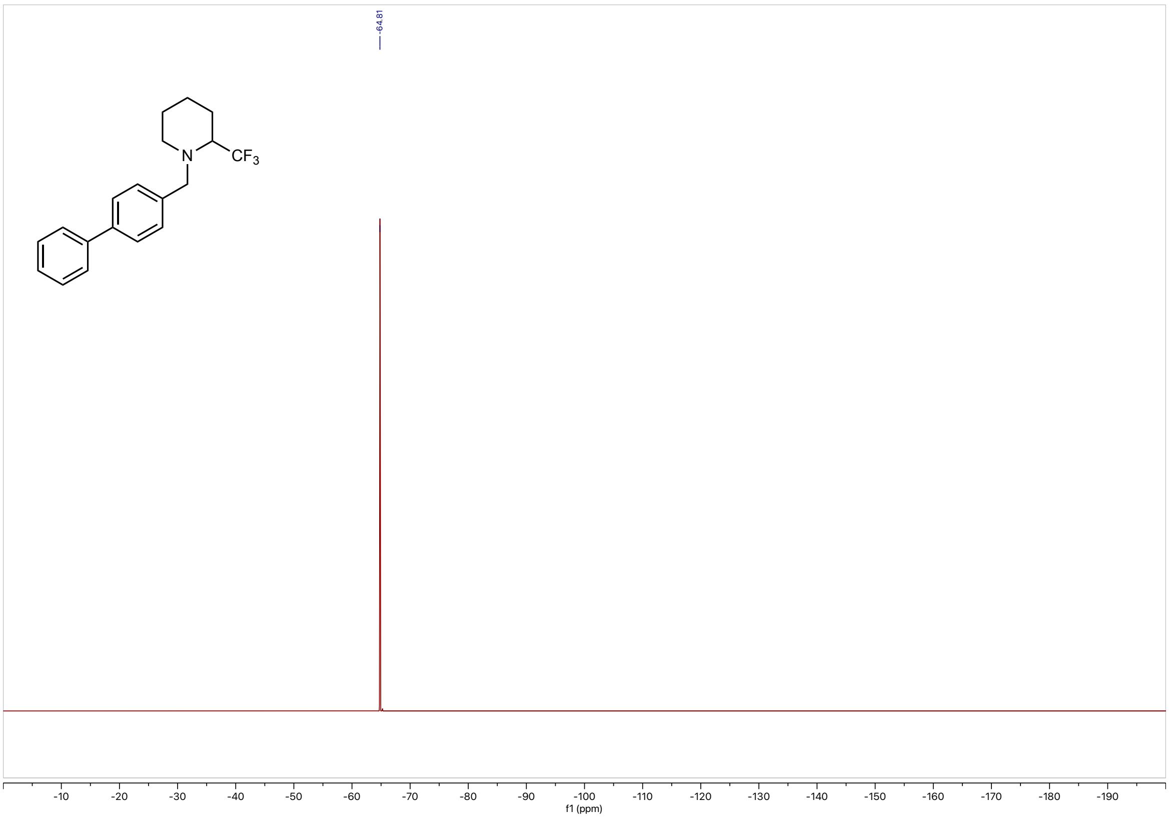

^{1}H NMR (500 MHz, $CDCl_3$) of 1-(4-phenylbenzyl)-2-methyl-2-(trifluoromethyl)piperidine (**9b**):

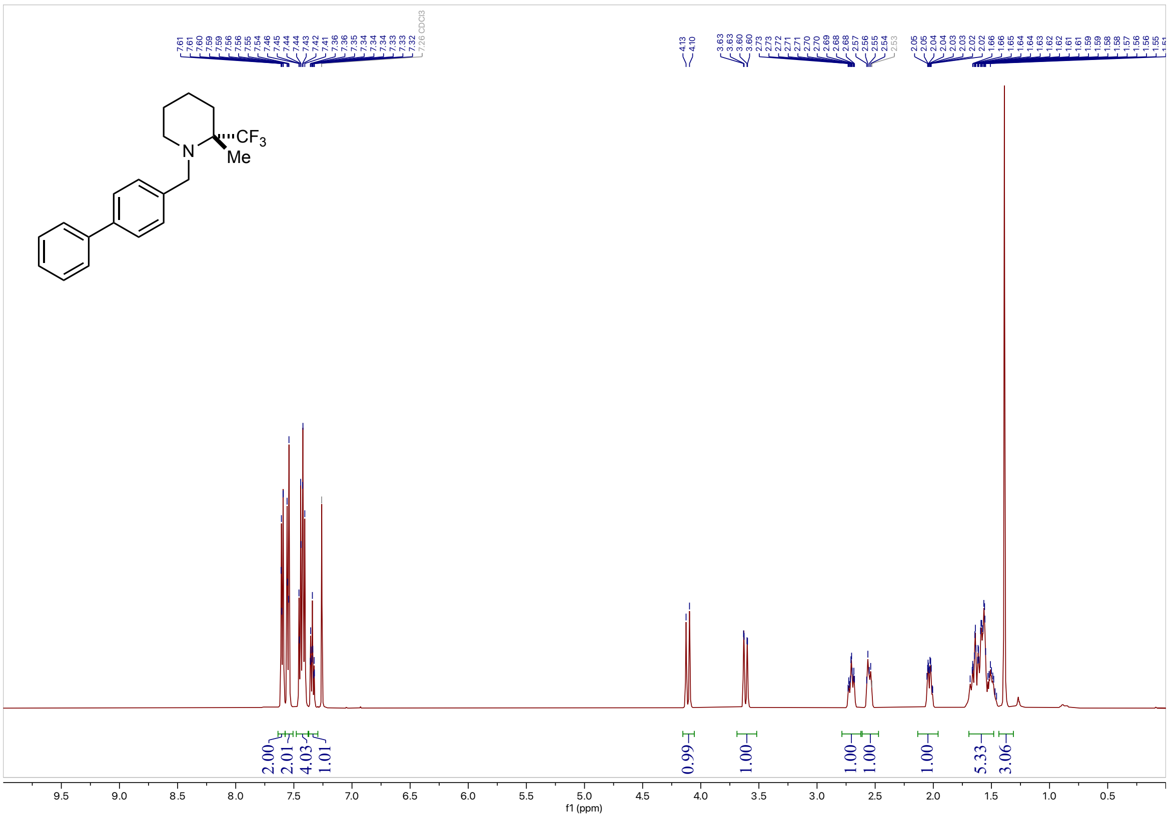

$^{13}C\{H\}$ NMR (126 MHz, $CDCl_3$) of 1-(4-phenylbenzyl)-2-methyl-2-(trifluoromethyl) piperidine (**9b**):

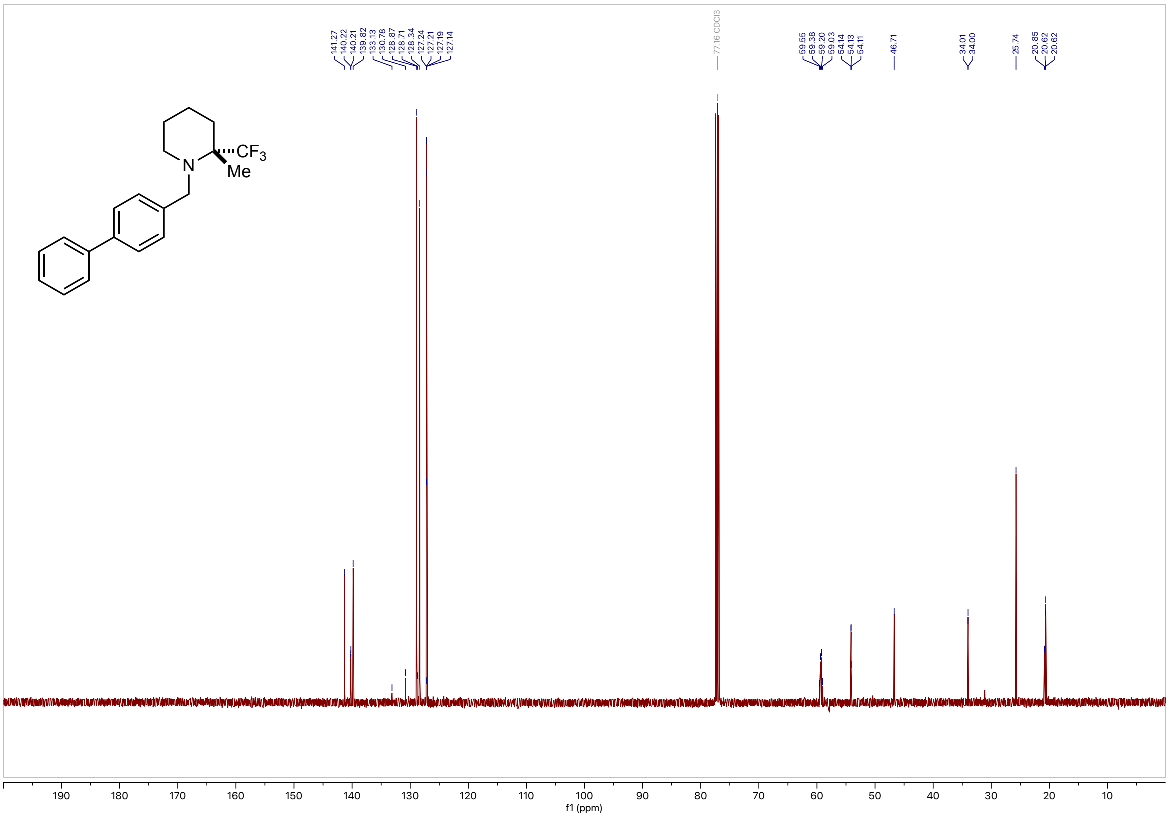

$^{19}F\{H\}$ NMR (471 MHz, $CDCl_3$) of 1-(4-phenylbenzyl)-2-methyl-2-(trifluoromethyl) piperidine (**9b**):

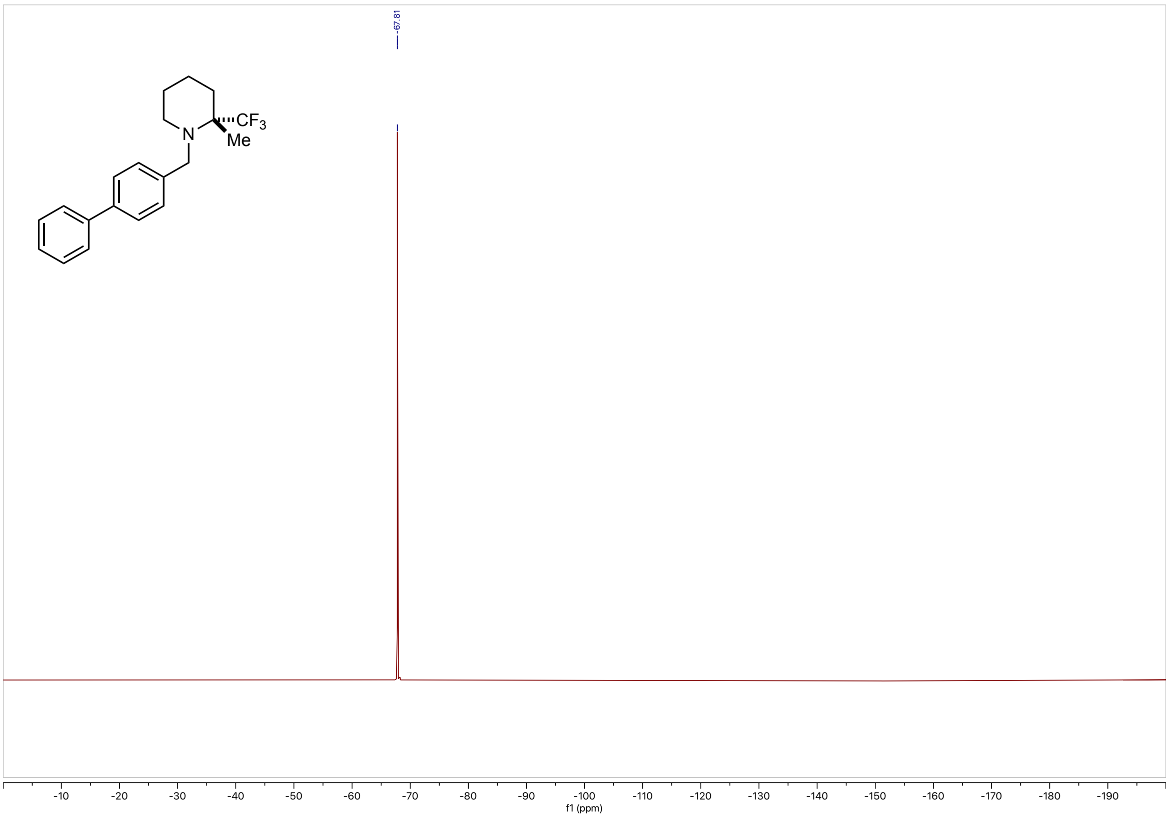

^{1}H NMR (700 MHz, $CDCl_3$) of 2-benzyl-1-(trifluoromethyl)-1,2,3,4-tetrahydroisoquinoline (**14d**):

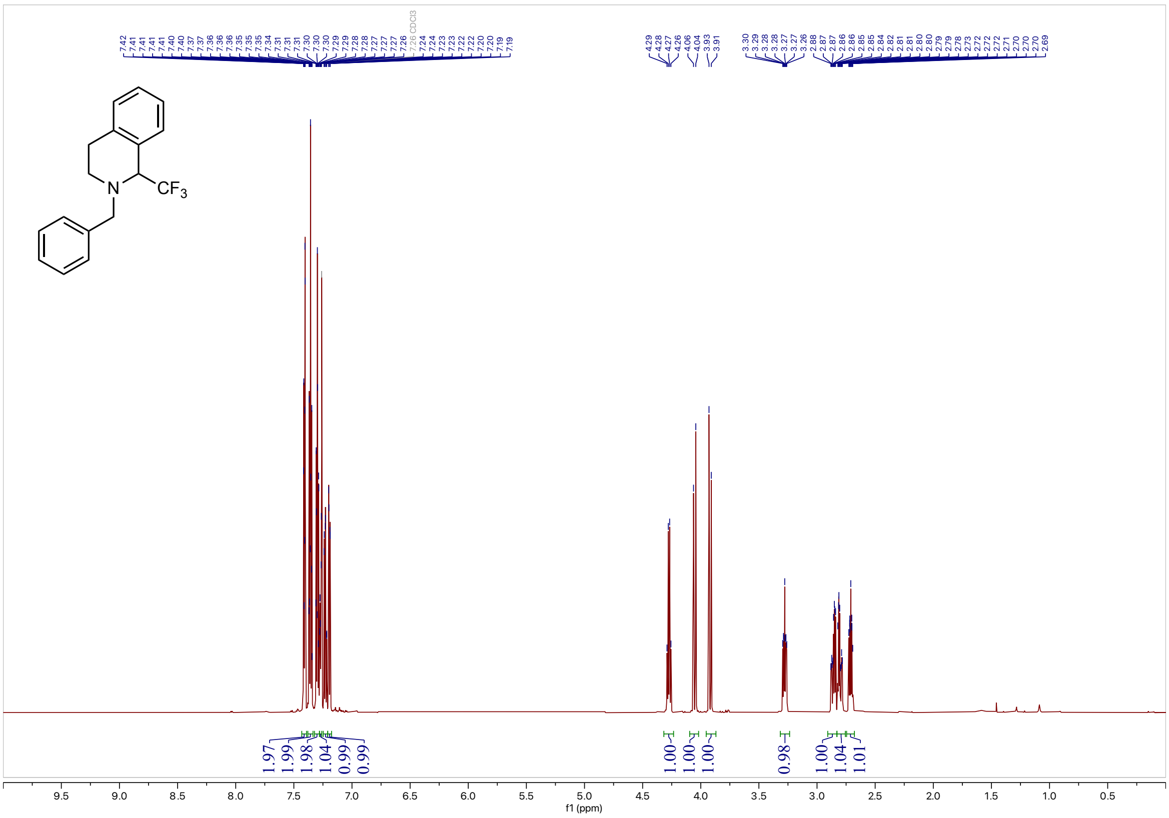

^{13}C{H} NMR (176 MHz, $CDCl_3$) of 2-benzyl-1-(trifluoromethyl)-1,2,3,4-tetrahydroisoquinoline (**14d**):

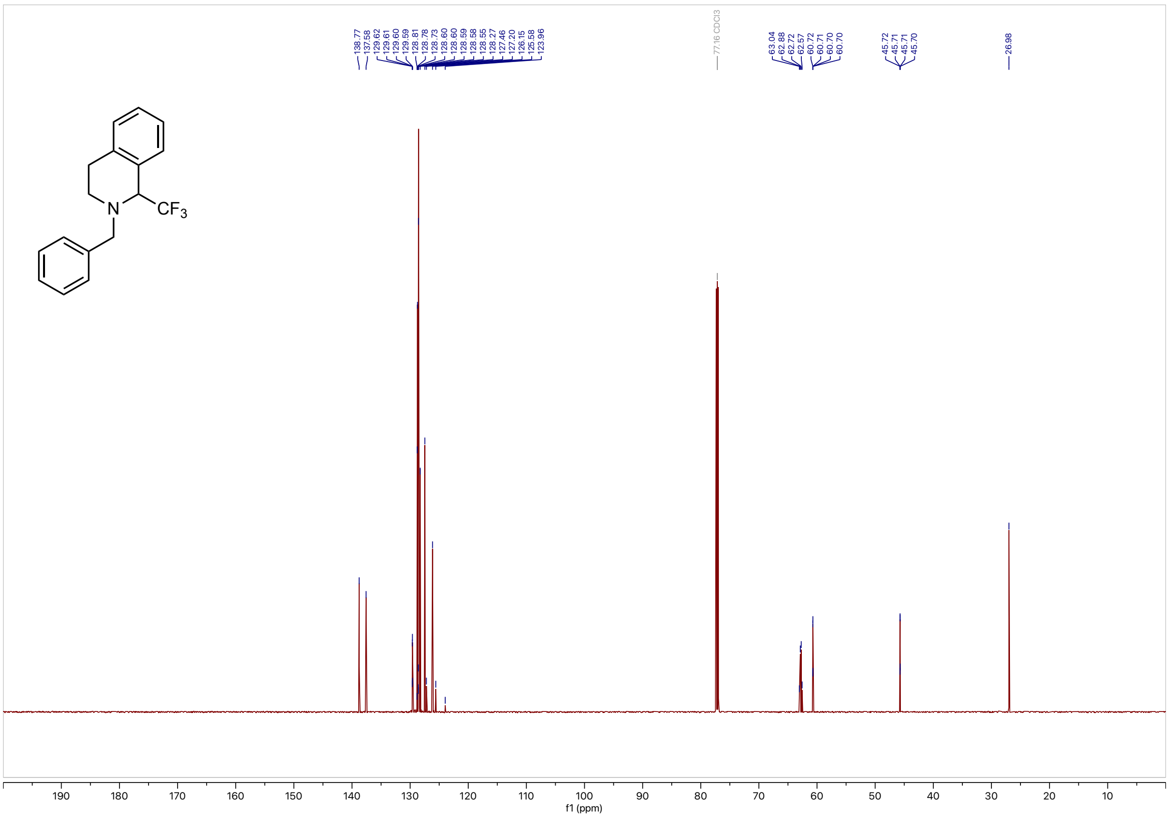

$^{19}F\{H\}$ NMR (176 MHz, $CDCl_3$) of 2-benzyl-1-(trifluoromethyl)-1,2,3,4-tetrahydroisoquinoline (**14d**):

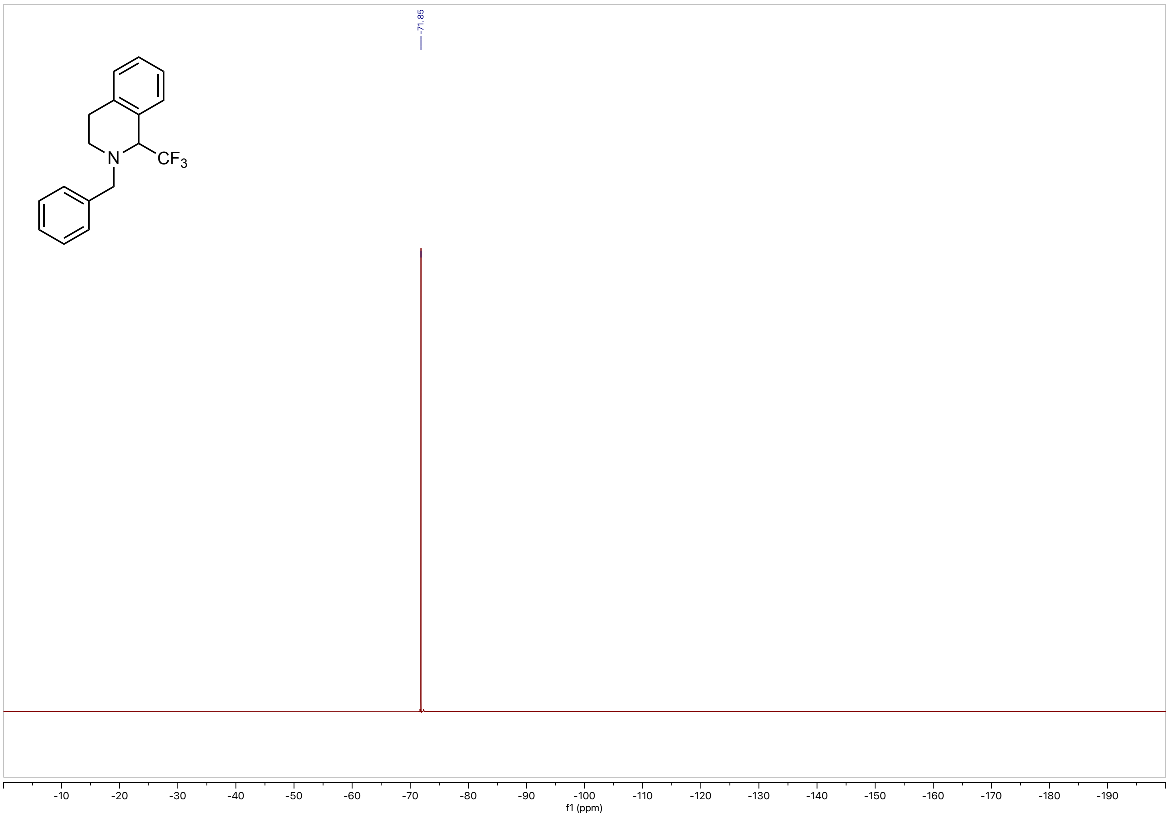

^{1}H NMR (400 MHz, $CDCl_3$) of 2-(1-benzylpiperidin-2-yl)pyridine (**10a**):

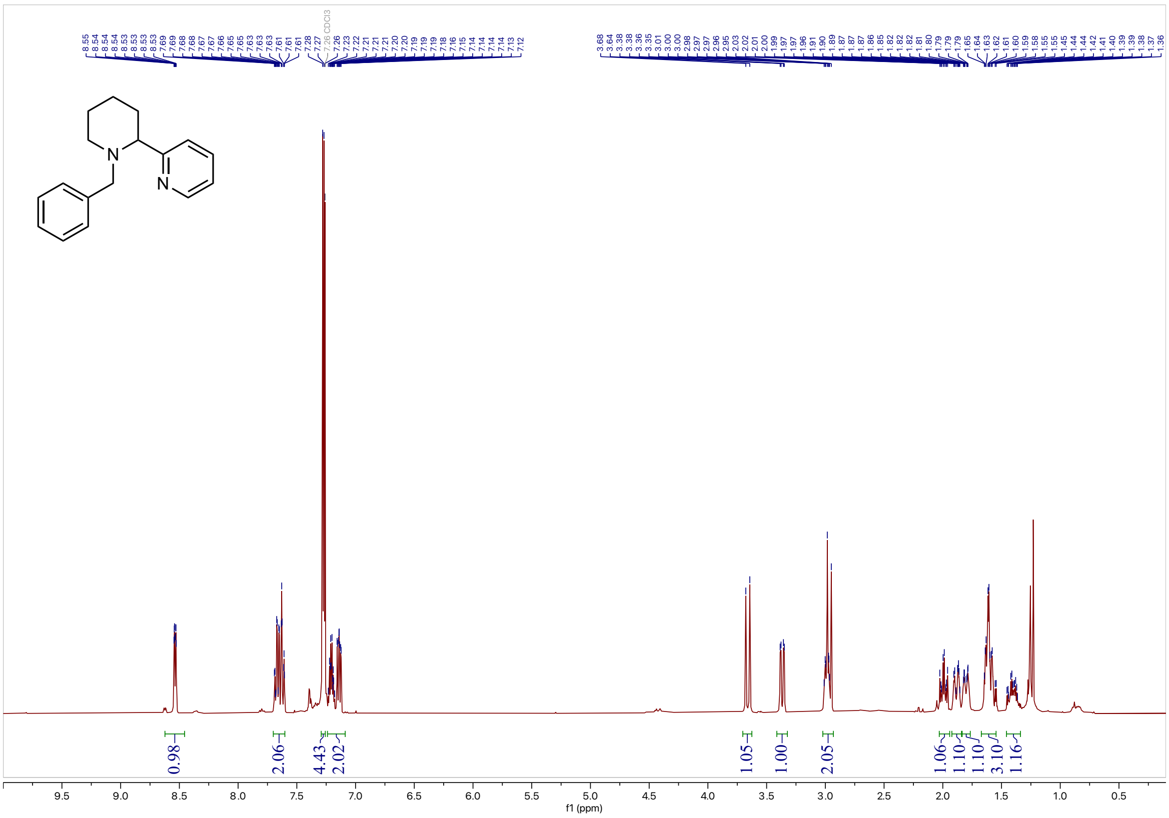

^{13}C{H} NMR (101 MHz, $CDCl_3$) of 2-(1-benzylpiperidin-2-yl)pyridine (**10a**):

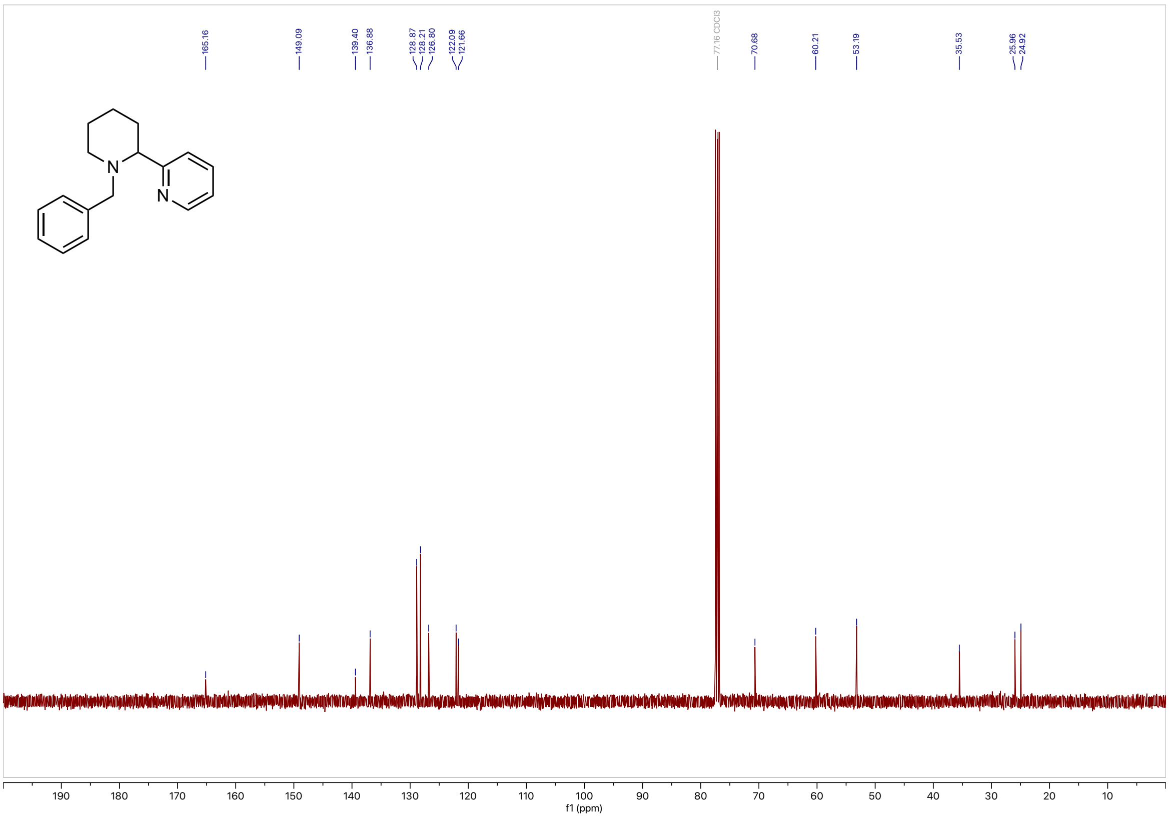

^{1}H NMR (500 MHz, $CDCl_3$) of 2-(1-benzylpiperidin-2-yl)-3-bromopyridine (**10b**):

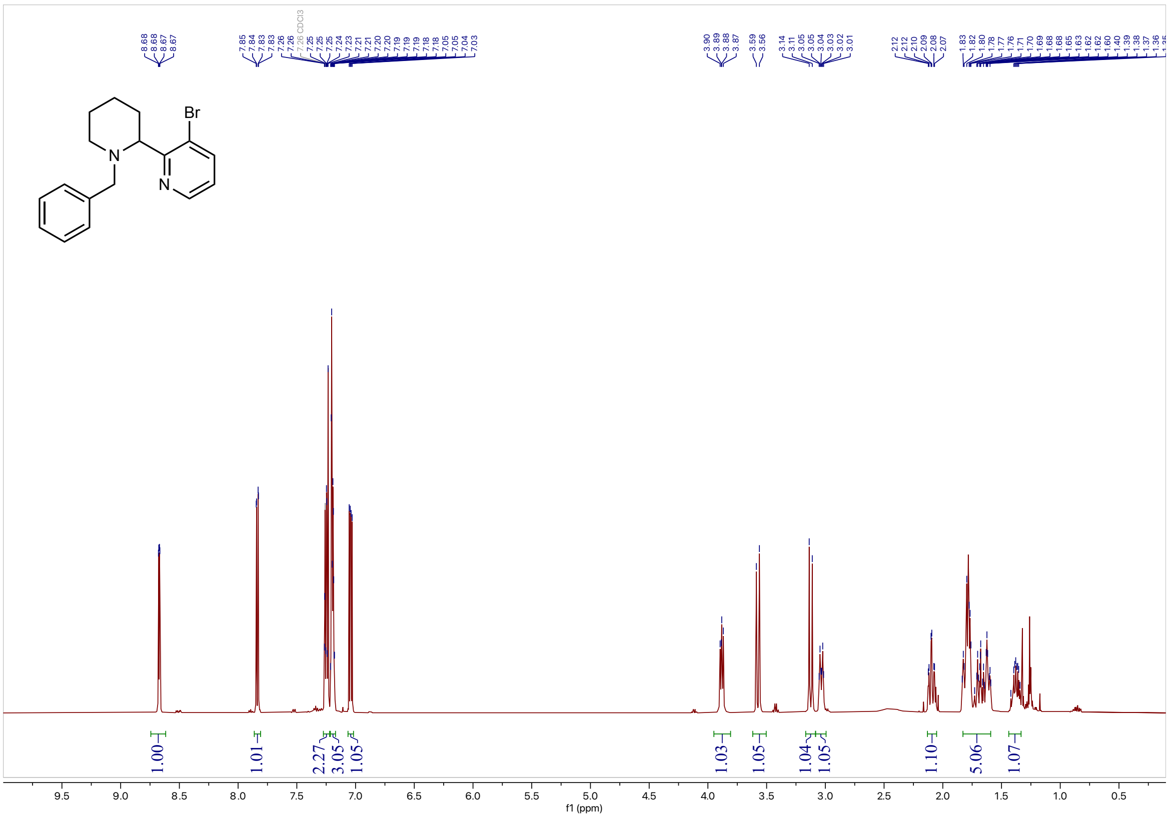

$^{13}C\{H\}$ NMR (126 MHz, $CDCl_3$) of 2-(1-benzylpiperidin-2-yl)-3-bromopyridine (**10b**):

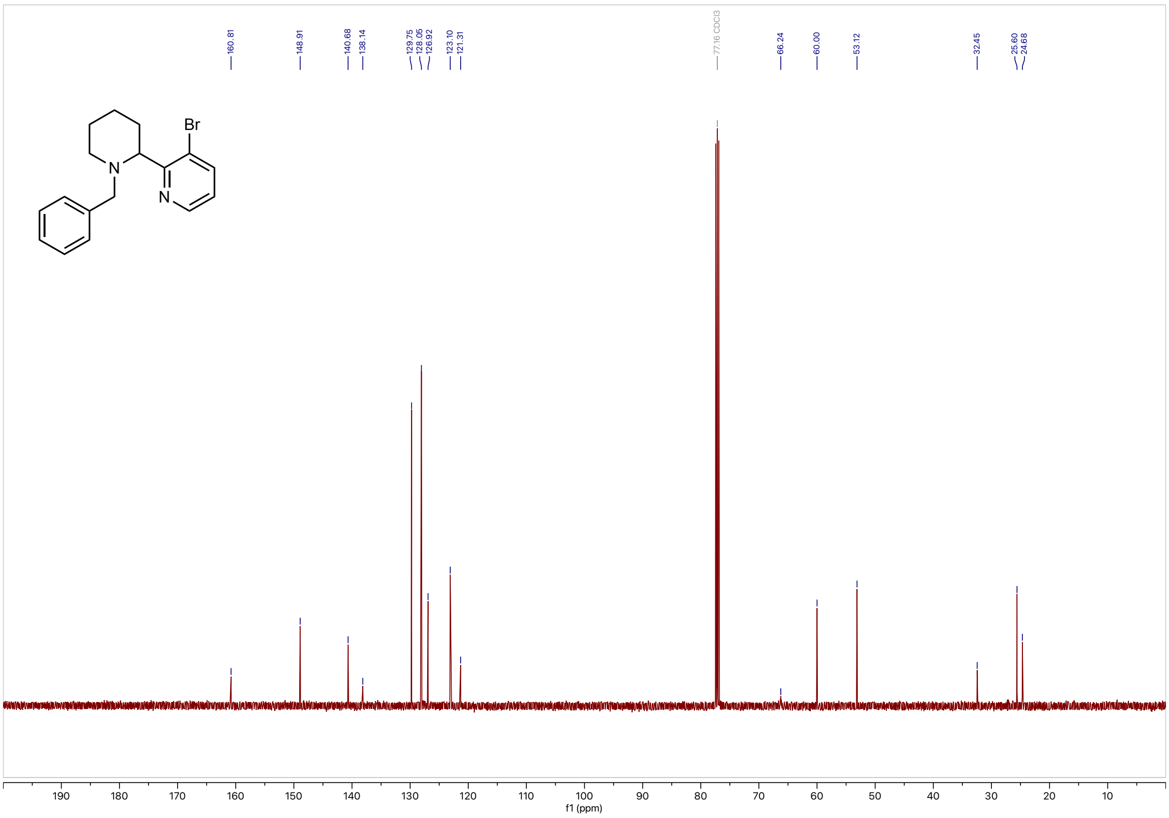

^{1}H NMR (500 MHz, $CDCl_3$) of 2-(1-benzylpiperidin-2-yl)-3-methoxypyridine (**10c**):

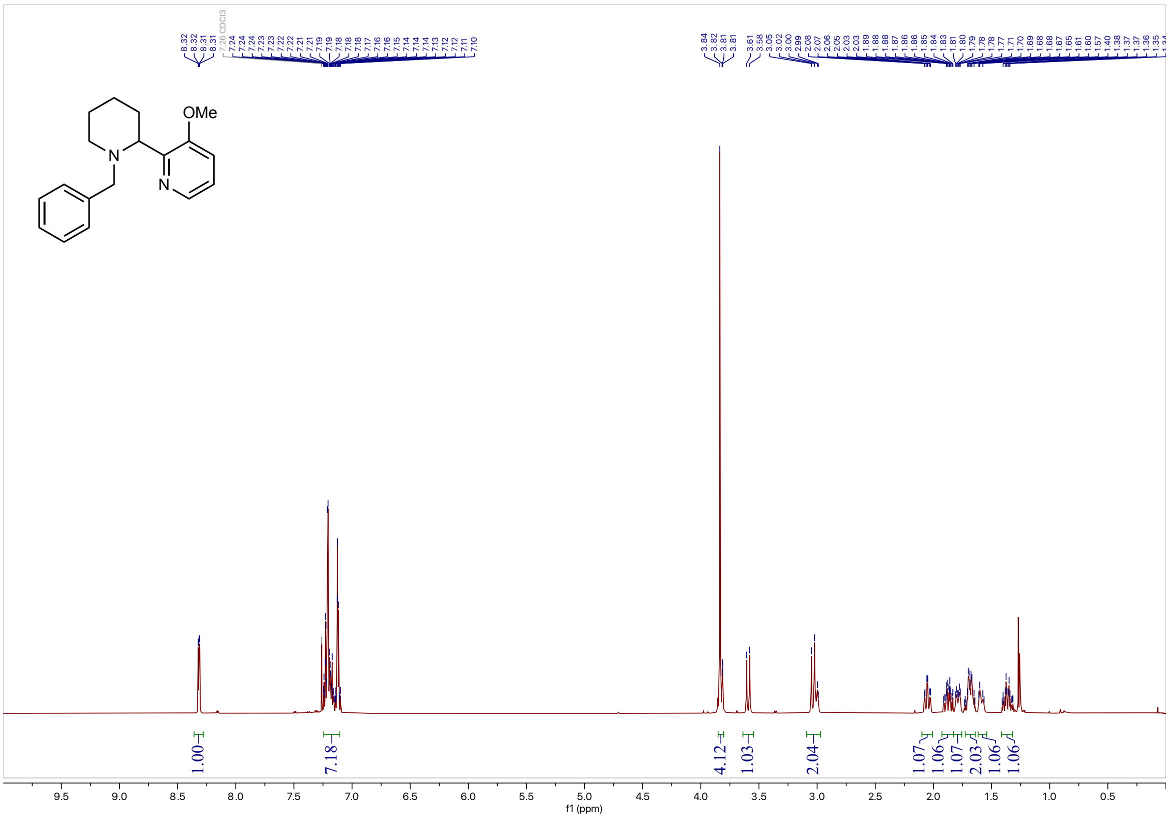

^{13}C{H} NMR (126 MHz, $CDCl_3$) of 2-(1-benzylpiperidin-2-yl)-3-methoxypyridine (**10c**):

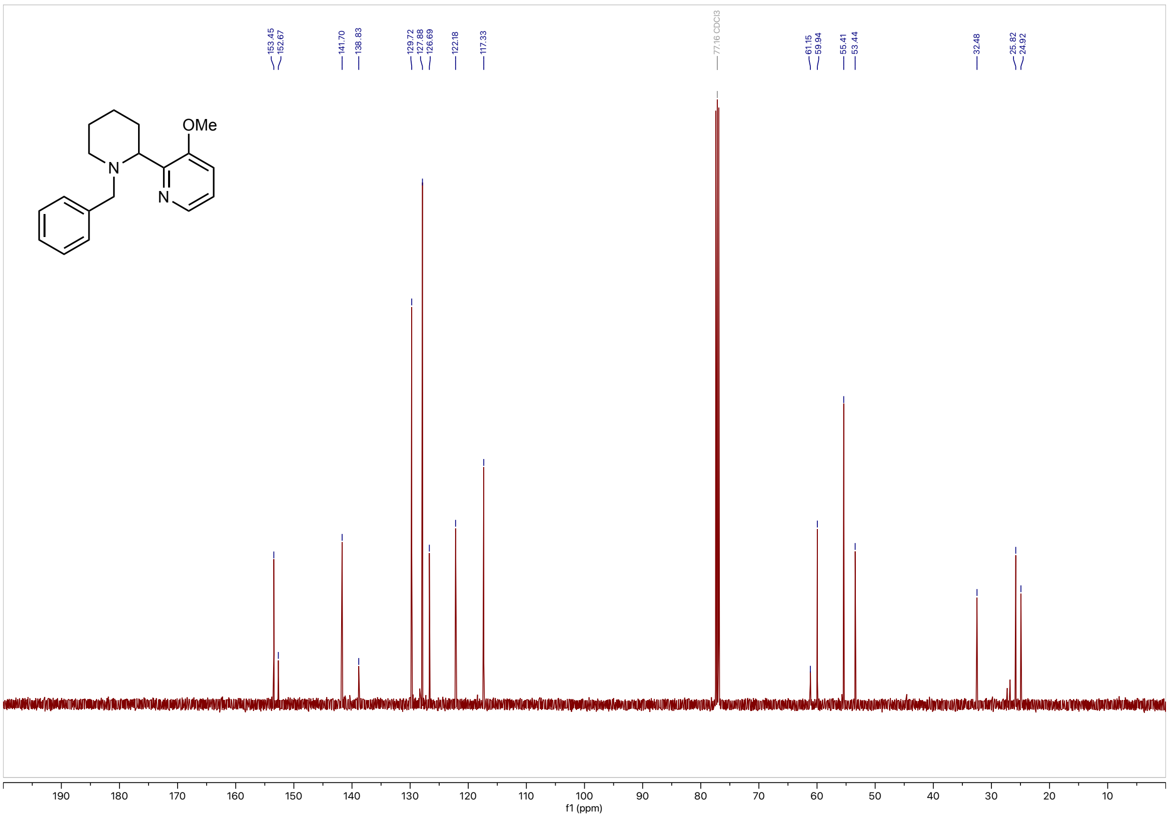

^{1}H NMR (400 MHz, $CDCl_3$) of 2-(1-benzylpiperidin-2-yl)-3-bromo-5-chloropyridine (**10d**):

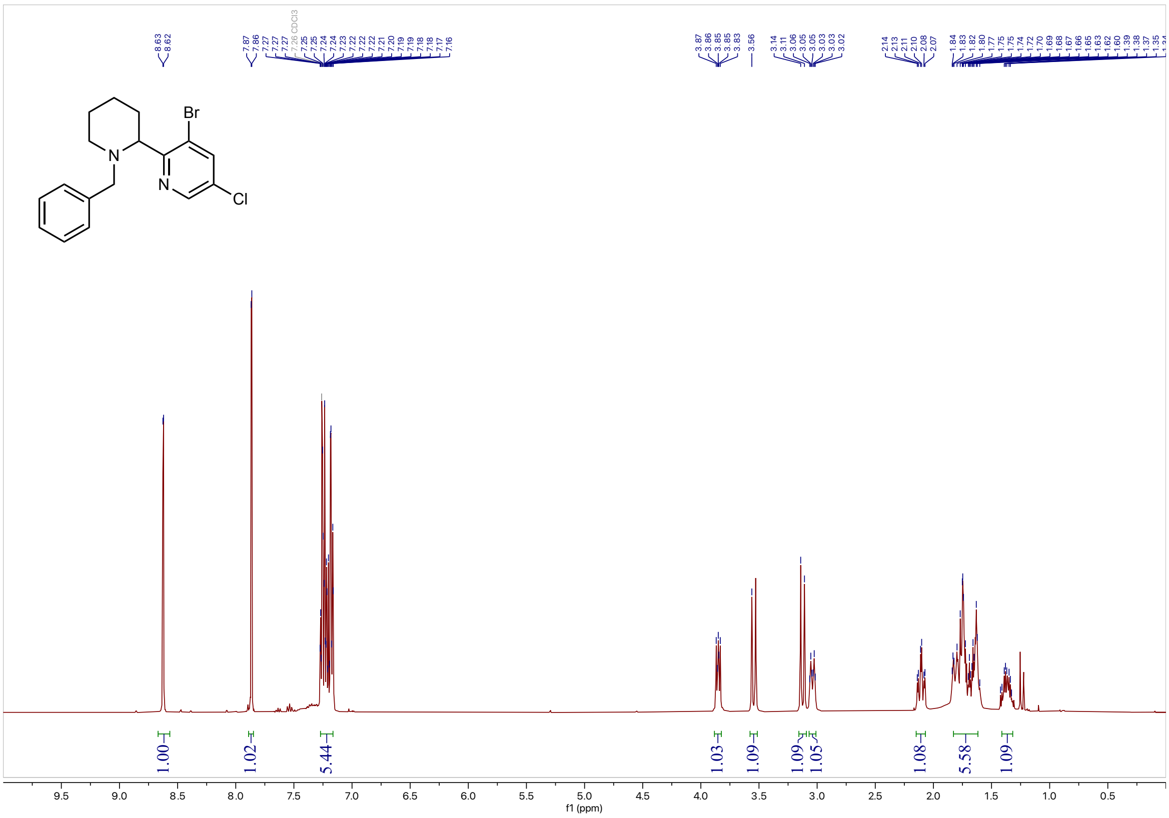

^{13}C{H} NMR (101 MHz, $CDCl_3$) of 2-(1-benzylpiperidin-2-yl)-3-bromo-5-chloropyridine (**10d**):

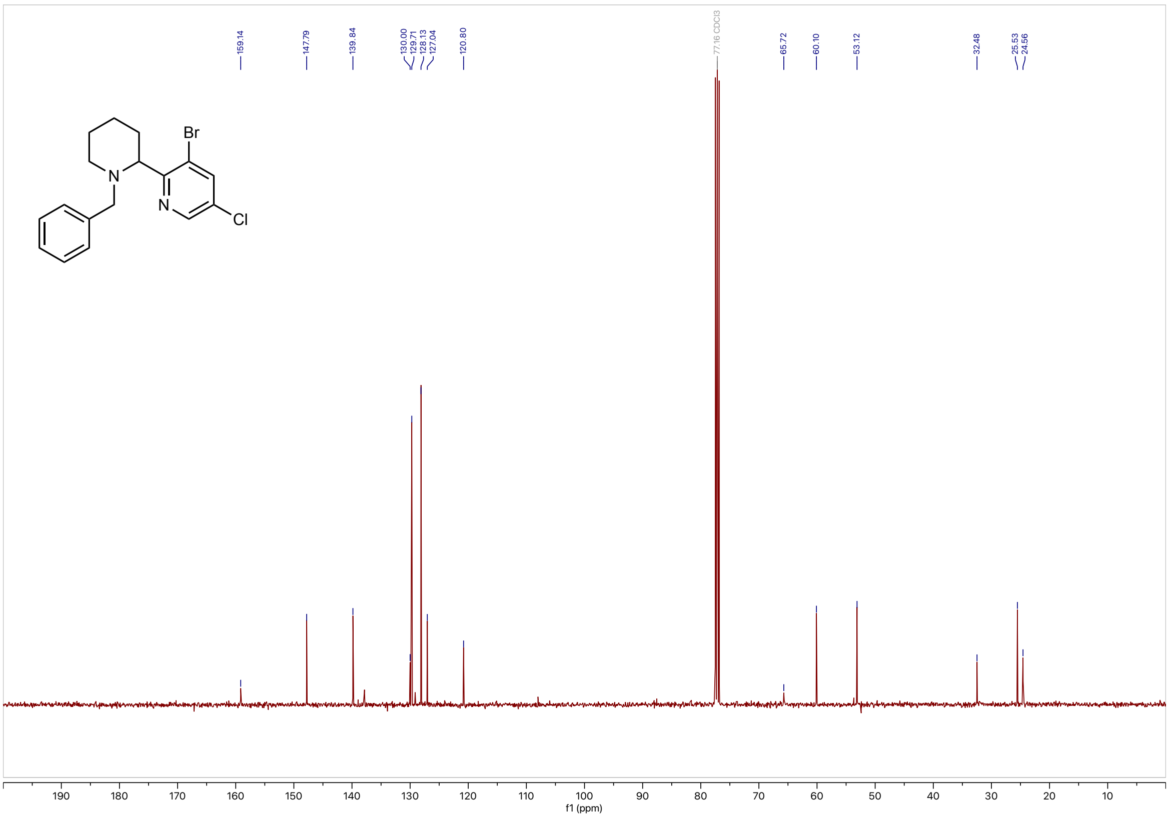

^{1}H NMR (400 MHz, $CDCl_3$) of 4-(1-benzylpiperidin-2-yl) furo[3,2-*c*]pyridine (**10e**):

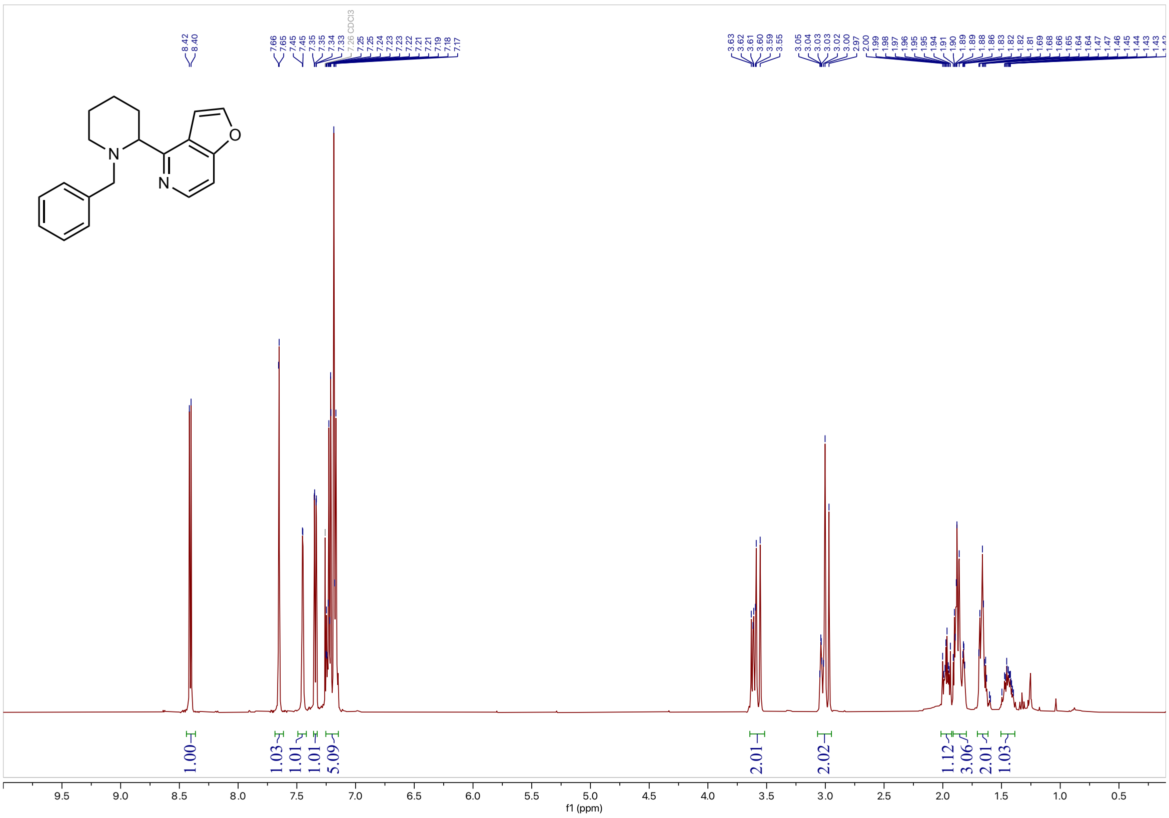

^{13}C{H} NMR (101 MHz, $CDCl_3$) of 4-(1-benzylpiperidin-2-yl) furo[3,2-*c*]pyridine (**10e**):

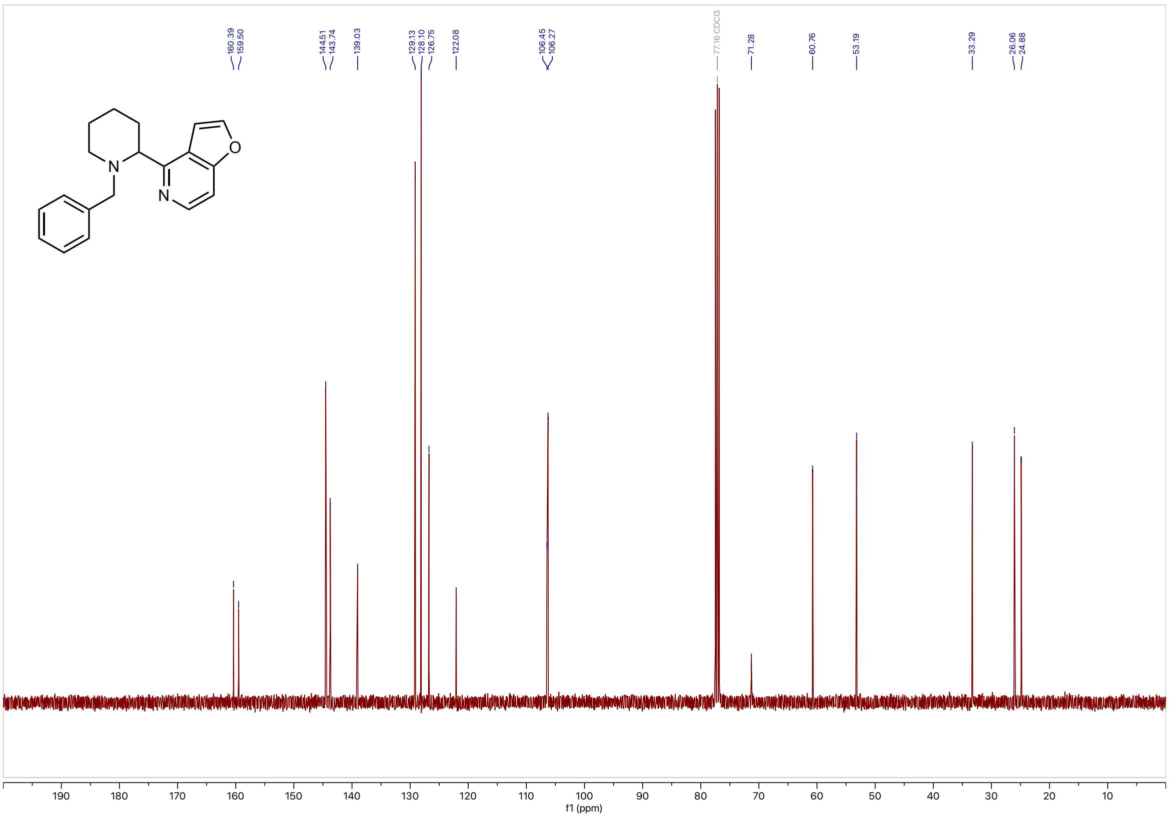

^{1}H NMR (400 MHz, $CDCl_3$) of 1-(1-benzylpiperidin-2-yl) isoquinoline (**10f**):

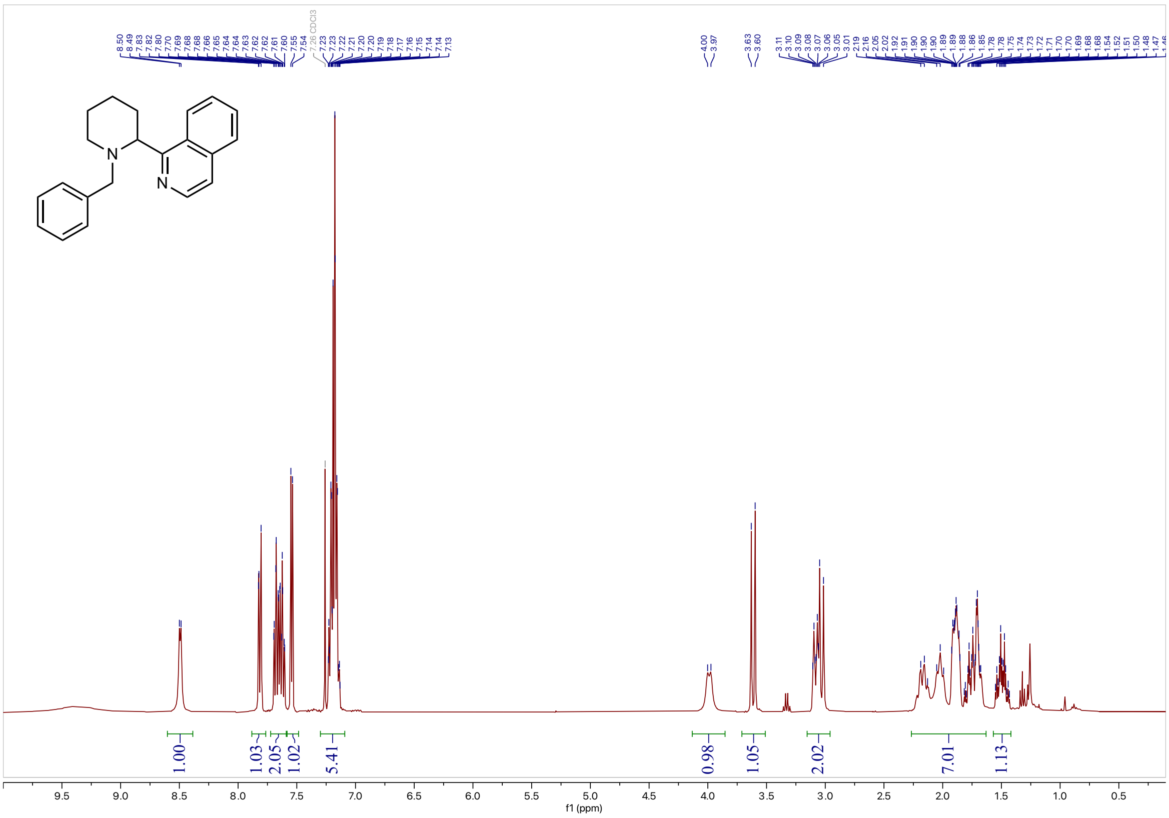

^{13}C{H} NMR (126 MHz, $CDCl_3$) of 1-(1-benzylpiperidin-2-yl) isoquinoline (**10f**):

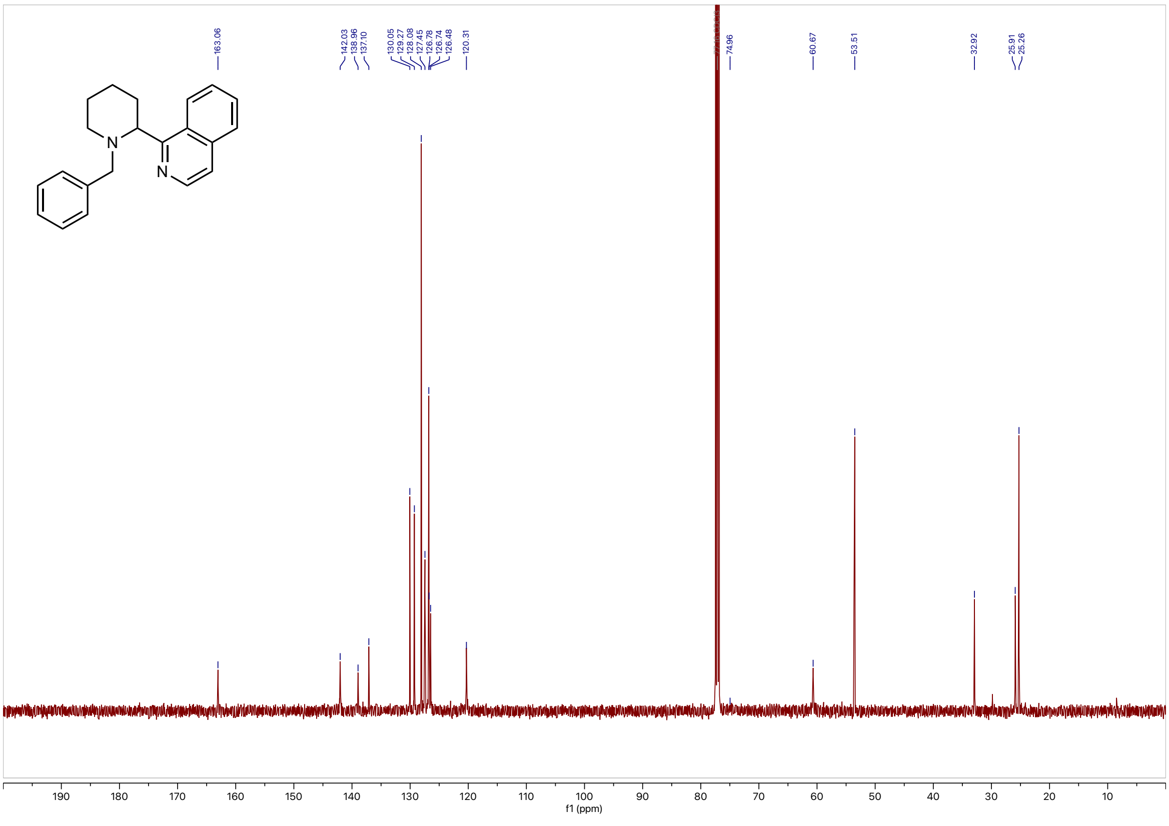

^{1}H NMR (700 MHz, CD_2Cl_2) of (4bS,8aS,9S)-3-methoxy-11-methyl-6,7,8,8a,9,10-hexahydro-5H-11λ[4]-9,4b-(aminediylethan[2]yl[1]ylidene)phenanthrene chloride (**16**):

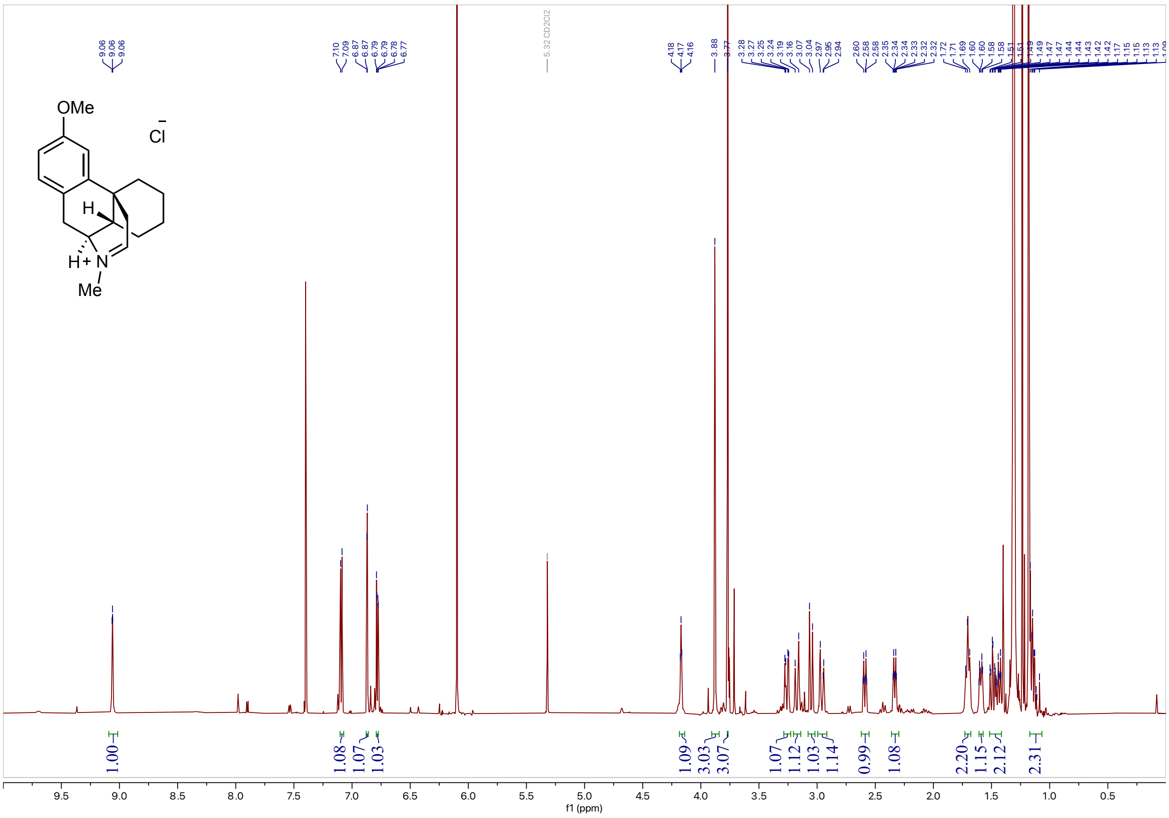

^{13}C{H} NMR (176 MHz, CD_2Cl_2) of (4bS,8aS,9S)-3-methoxy-11-methyl-6,7,8,8a,9,10-hexahydro-5H-11λ[4]-9,4b-(aminediylethan[2]yl[1]ylidene)phenanthrene chloride (**16**):

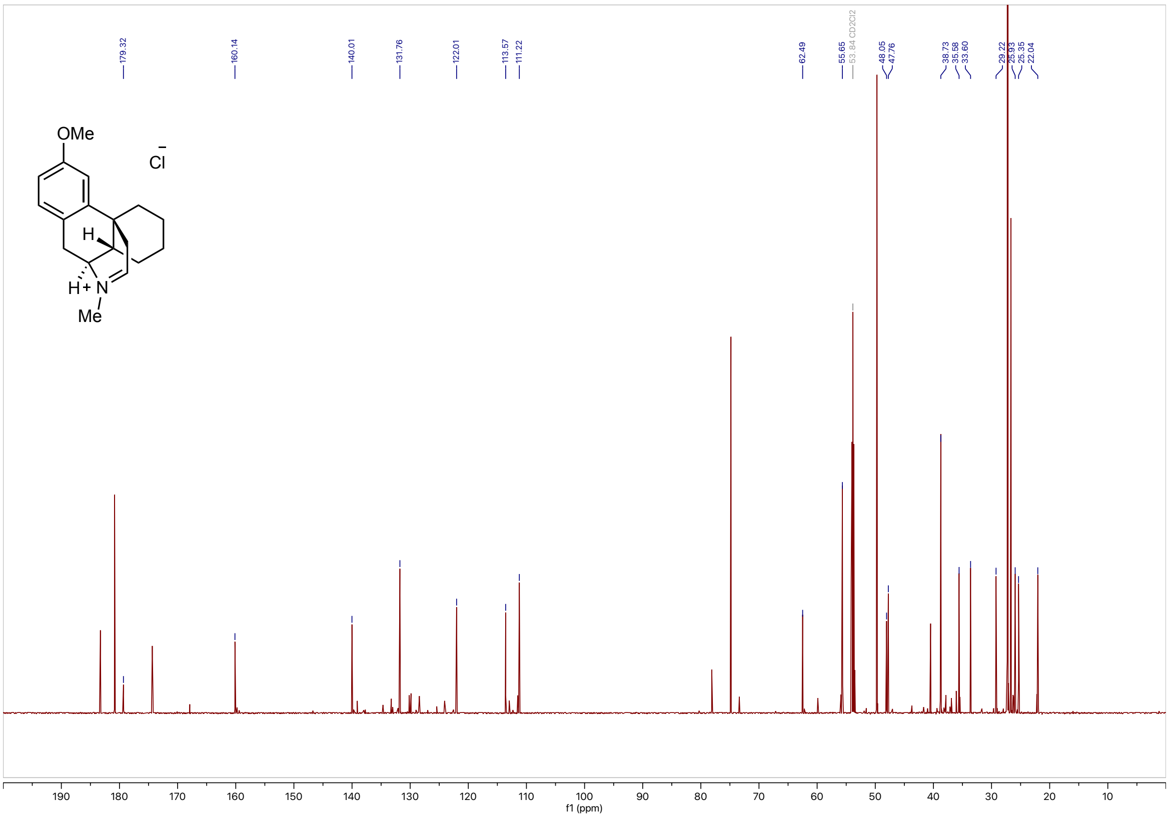

^{1}H NMR (500 MHz, $CDCl_3$) of (4bS,8aS,9S,12R)-3-methoxy-11,12-dimethyl-6,7,8,8a,9,10-hexahydro-5H-9,4b-(epiminoethano)phenanthrene (**17a**):

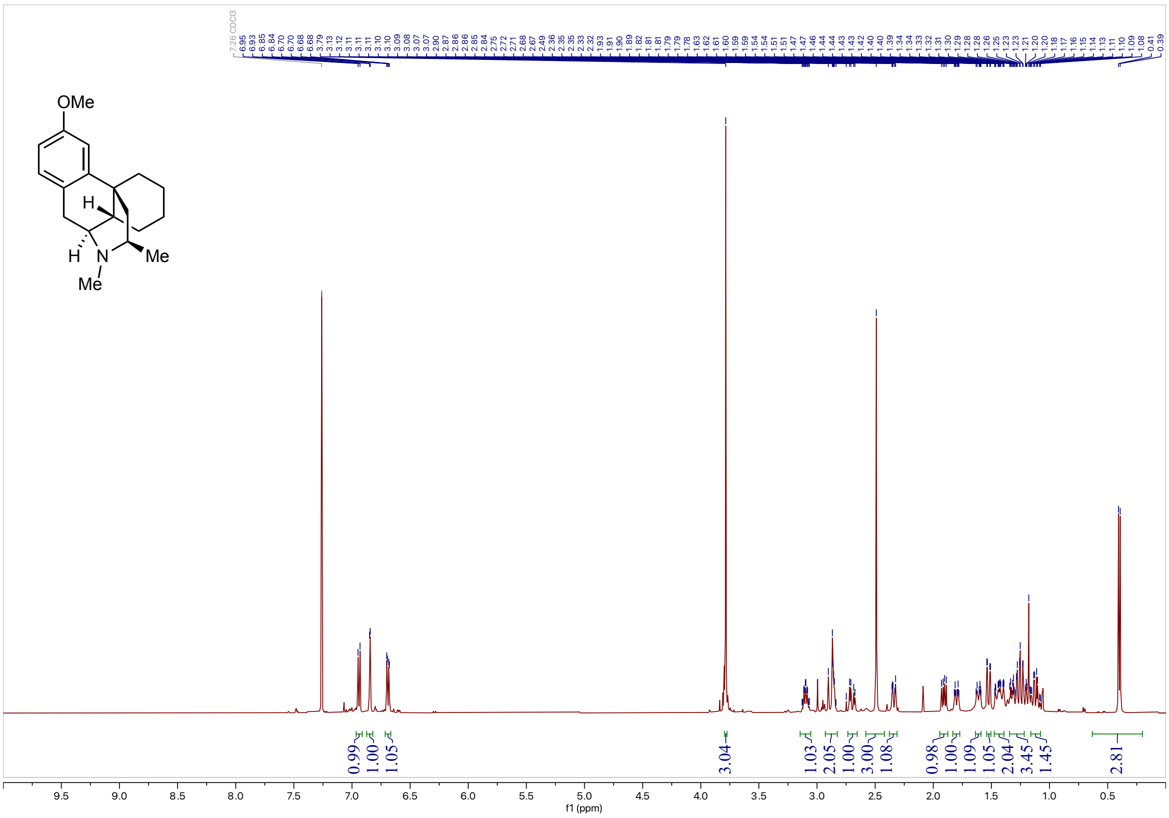

^{13}C{H} NMR (126 MHz, $CDCl_3$) of (4bS,8aS,9S,12R)-3-methoxy-11,12-dimethyl-6,7,8,8a,9,10-hexahydro-5H-9,4b-(epiminoethano)phenanthrene (**17a**):

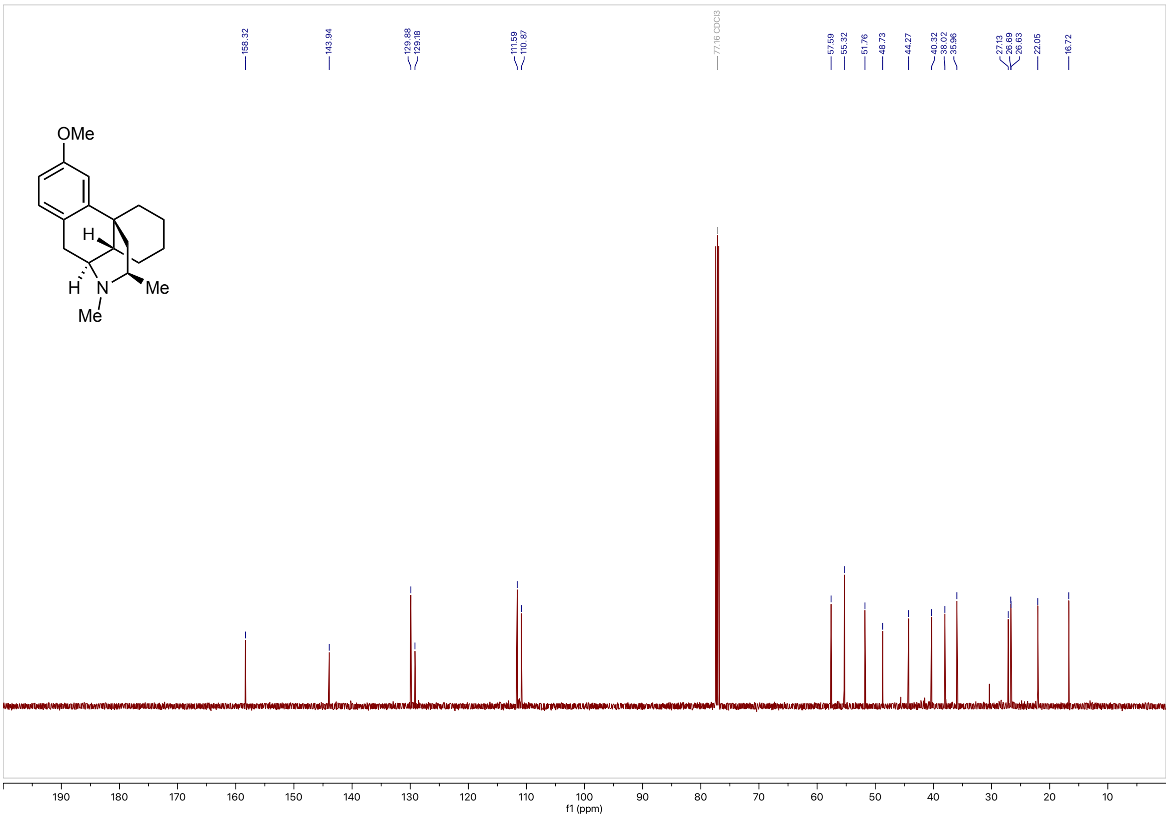

NOSEY (500 MHz, $CDCl_3$) of (*4bS,8aS,9S,12R*)-3-methoxy-11,12-dimethyl-6,7,8,8a,9,10-hexahydro-5H-9,4b- (epiminoethano)phenanthrene (**17a**):

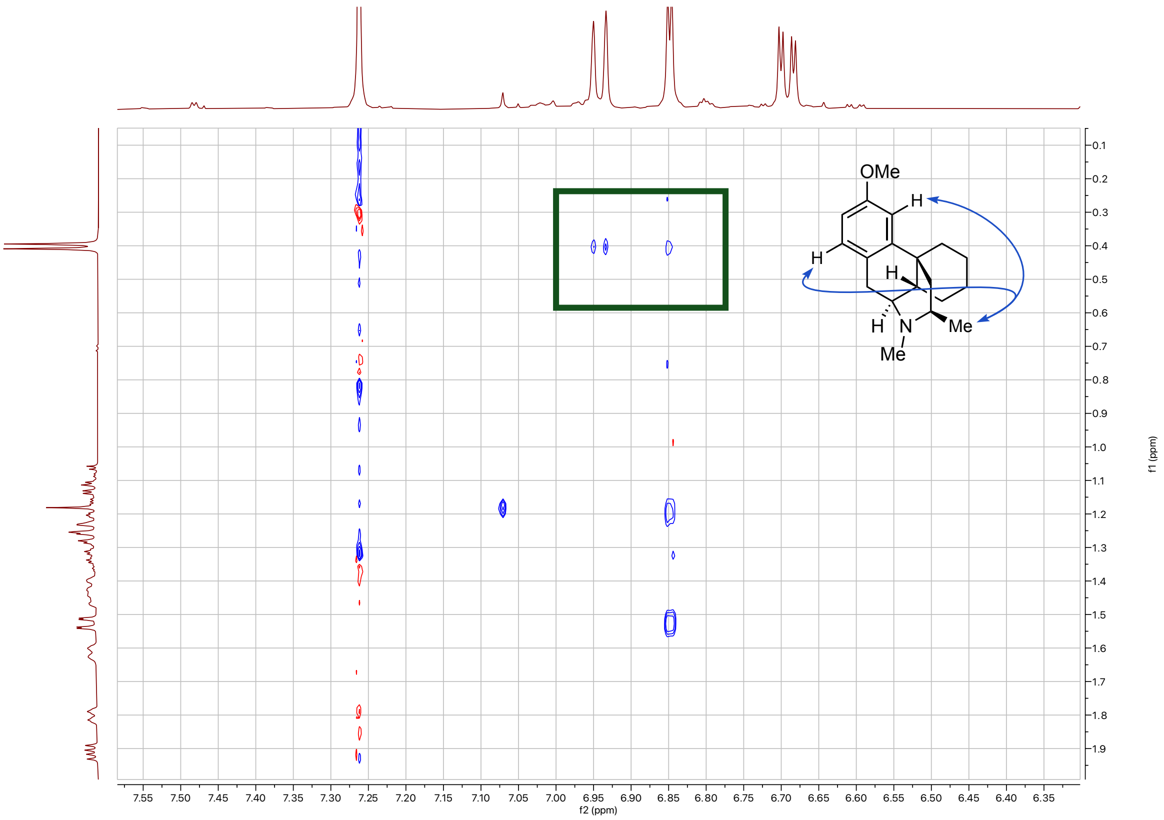

^{1}H NMR (700 MHz, $CDCl_3$) of (4bS,8aS,9S)-12-isopropyl-3-methoxy-11-methyl-6,7,8,8a,9,10-hexahydro-5H-9,4b-(epiminoethano)phenanthrene (**17b**):

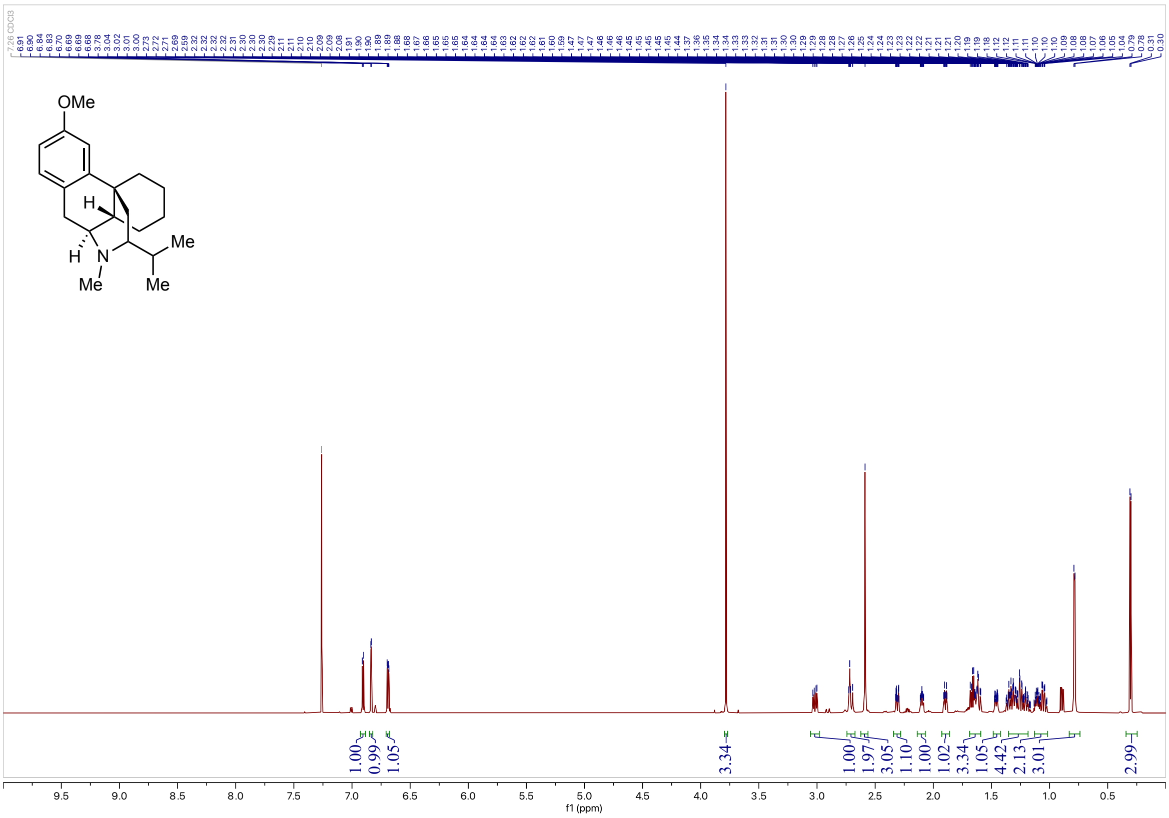

^{13}C{H} NMR (176 MHz, $CDCl_3$) of (4bS,8aS,9S)-12-isopropyl-3-methoxy-11-methyl-6,7,8,8a,9,10-hexahydro-5H-9,4b-(epiminoethano)phenanthrene (**17b**):

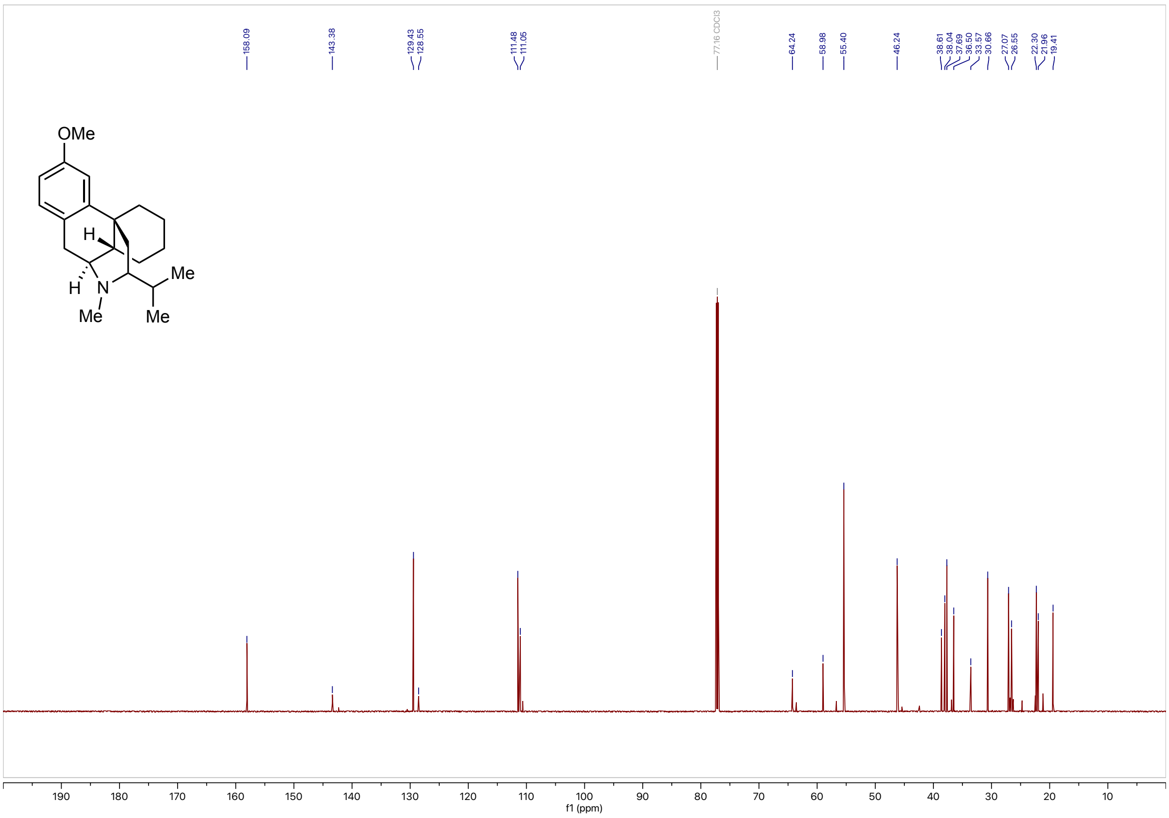

^{1}H NMR (700 MHz, $CDCl_3$) of (4bS,8aS,9S,12R)-3-methoxy-11-methyl-6,7,8,8a,9,10-hexahydro-5H-9,4b-(epiminoethano)phenanthren-12-yl)methanol (**17c**):

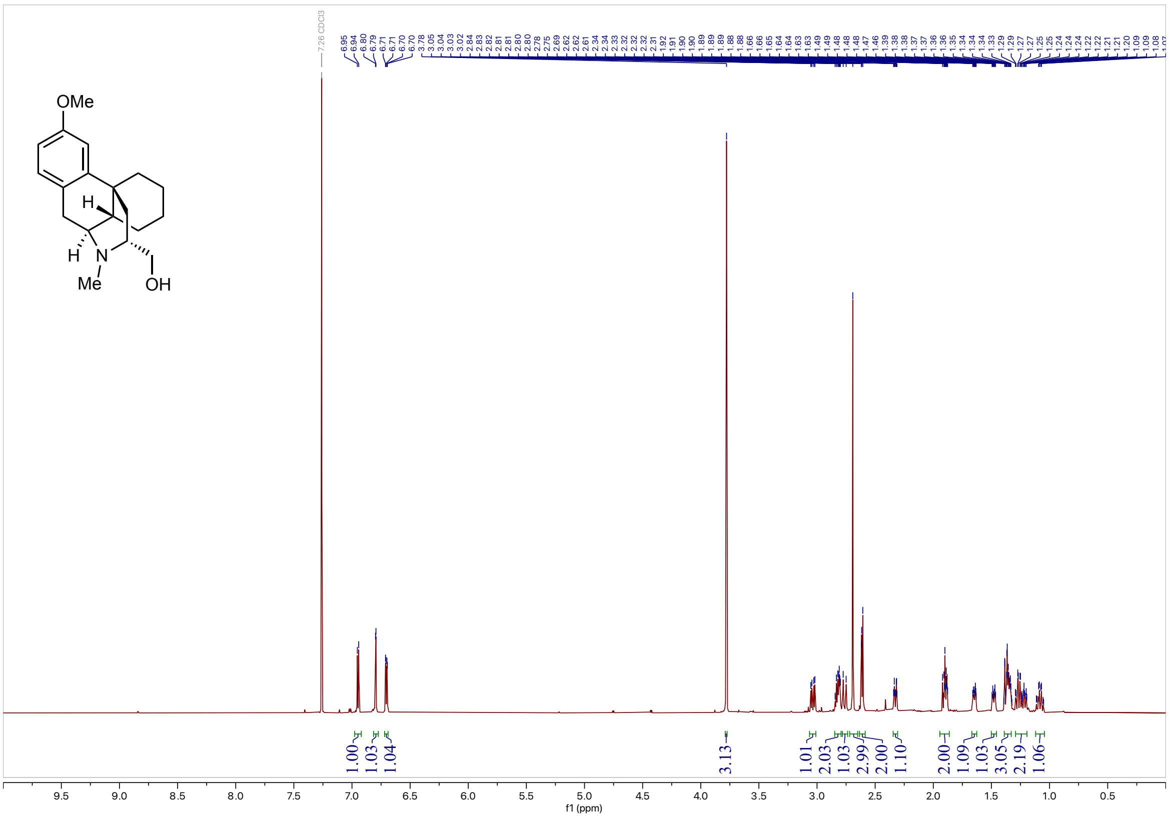

^{13}C{H} NMR (176 MHz, $CDCl_3$) of (4bS,8aS,9S,12R)-3-methoxy-11-methyl-6,7,8,8a,9,10-hexahydro-5H-9,4b-(epiminoethano)phenanthren-12-yl)methanol (**17c**):

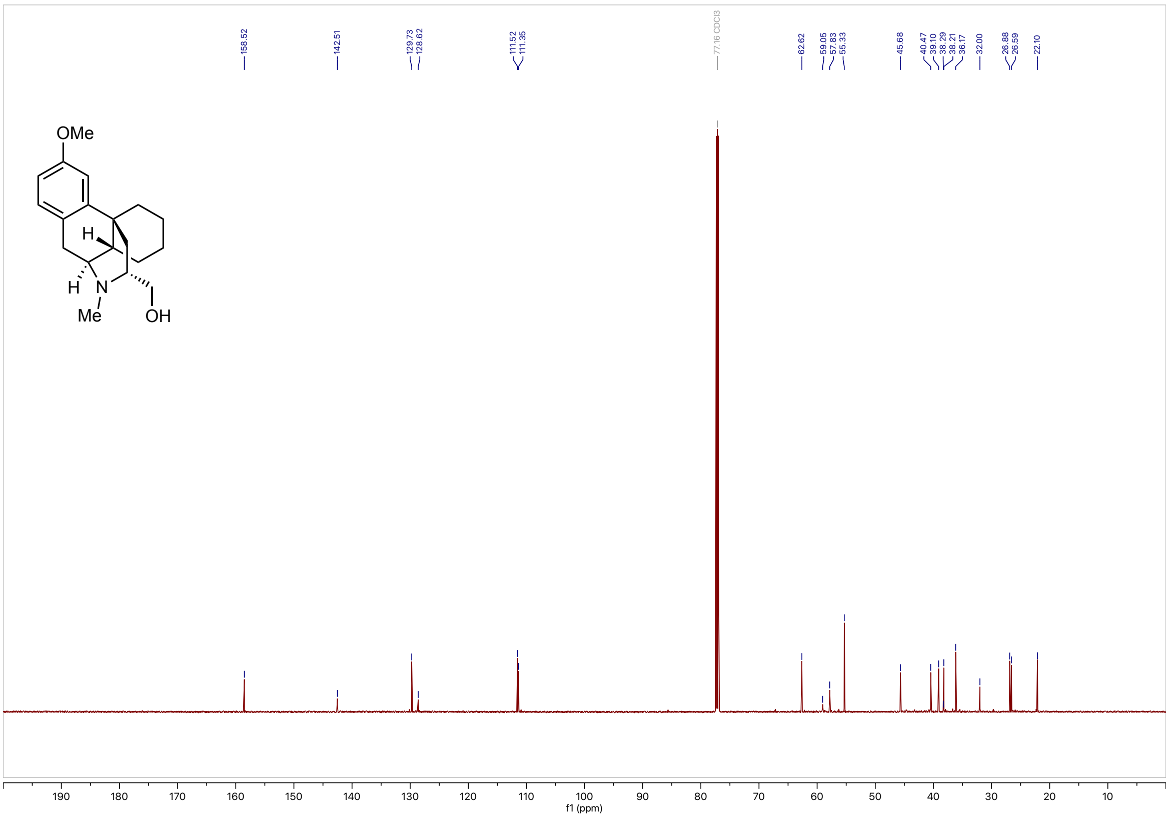

^{1}H NMR (700 MHz, $CDCl_3$) of (4bS,8aS,9S,12S)-3-methoxy-11-methyl-6,7,8,8a,9,10-hexahydro-5H-9,4b-(epiminoethano)phenanthren-12-yl)methanol (**17c’**):

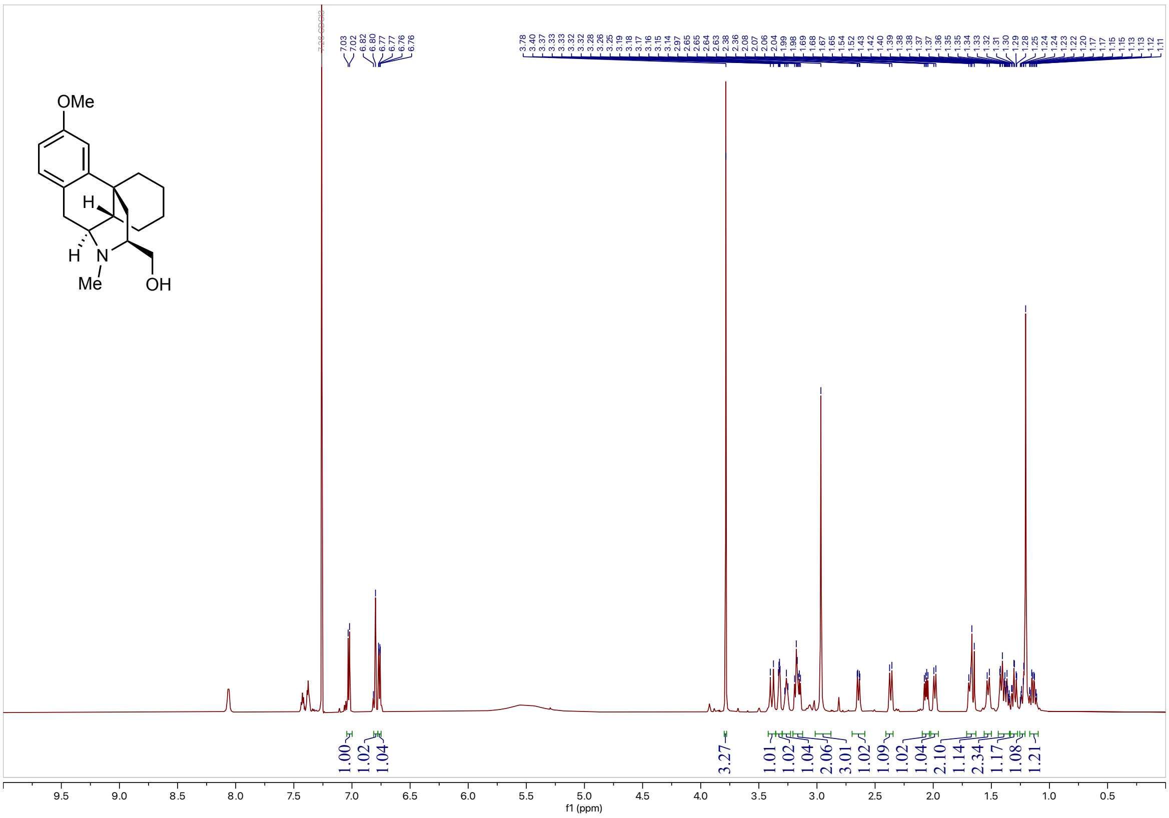

$^{13}C\{H\}$ NMR (126 MHz, $CDCl_3$) of (4bS,8aS,9S,12S)-3-methoxy-11-methyl-6,7,8,8a,9,10-hexahydro-5H-9,4b-(epiminoethano)phenanthren-12-yl)methanol (**17c’**):

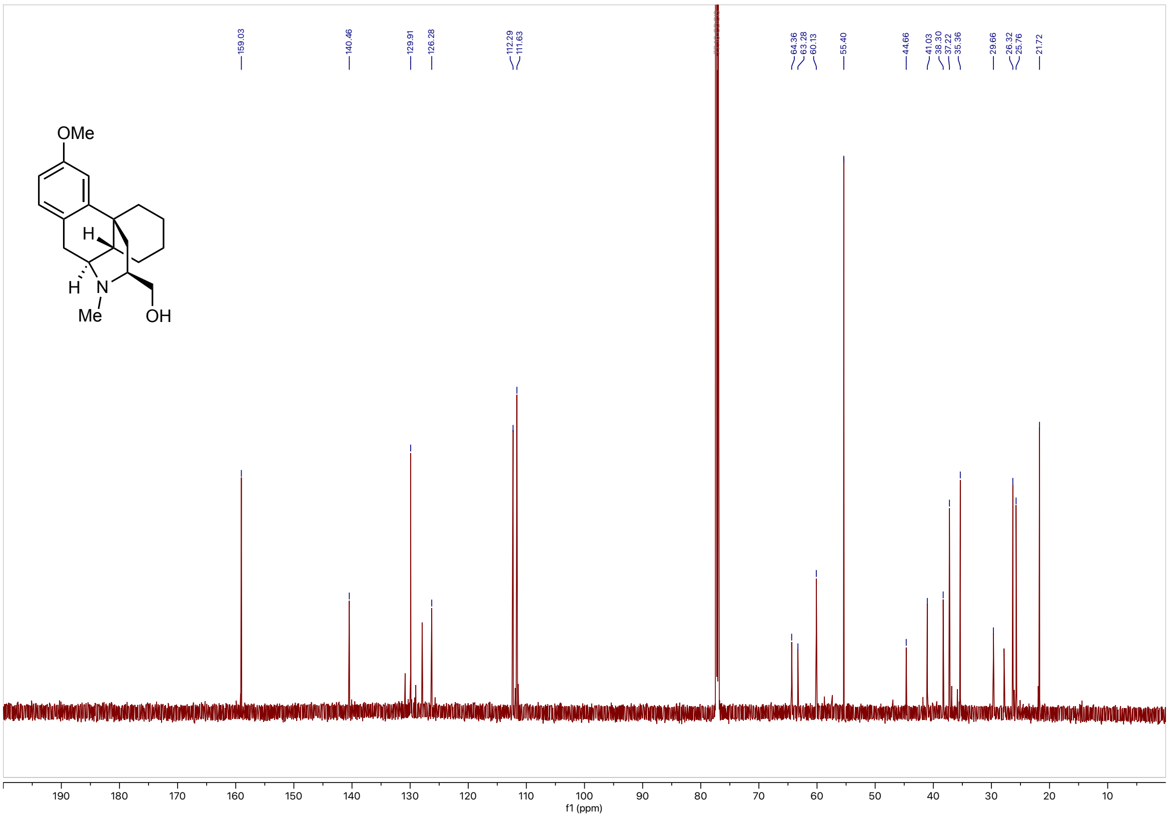

^{1}H NMR (700 MHz, $CDCl_3$) of (4bS,8aS,9S)-12-(fluoromethyl)-3-methoxy-11-methyl-6,7,8,8a,9,10-hexahydro-5H-9,4b-(epiminoethano)phenanthrene (**17d**):

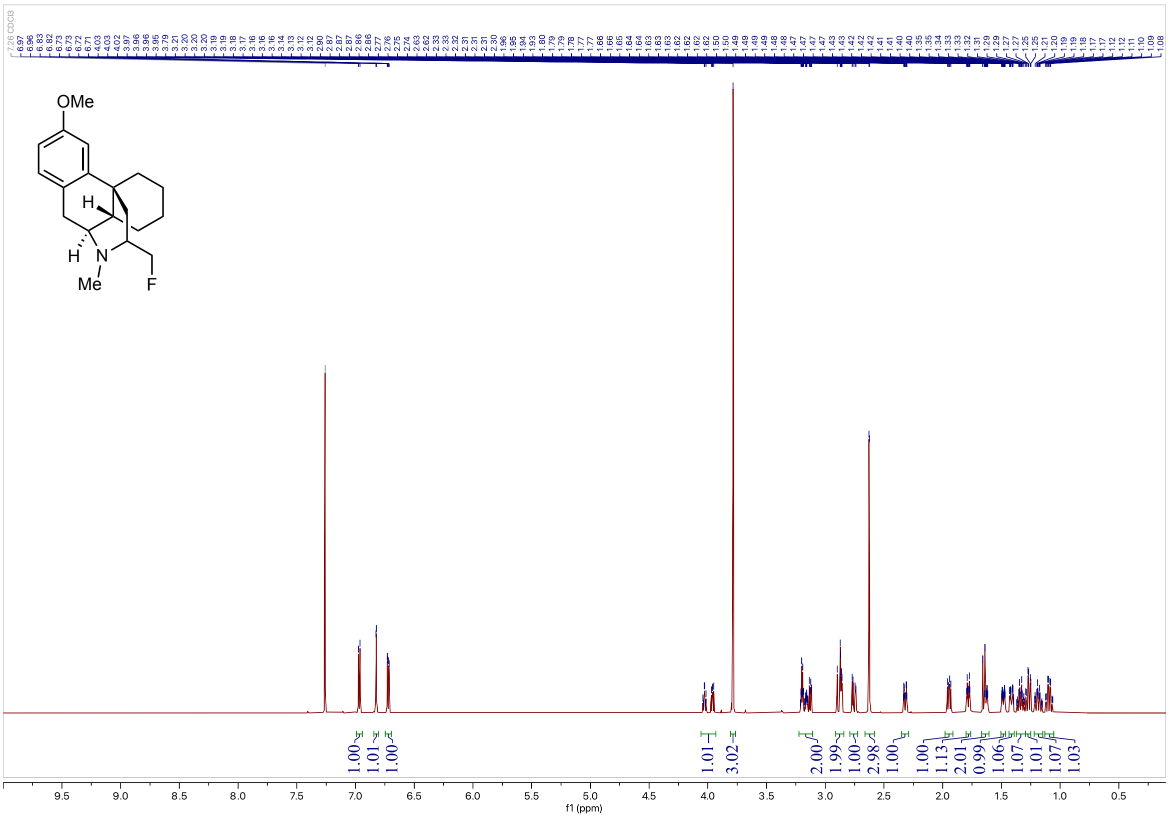

$^{13}C\{H\}$ NMR (176 MHz, $CDCl_3$) of (4bS,8aS,9S)-12-(fluoromethyl)-3-methoxy-11-methyl-6,7,8,8a,9,10-hexahydro-5H-9,4b-(epiminoethano)phenanthrene (**17d**):

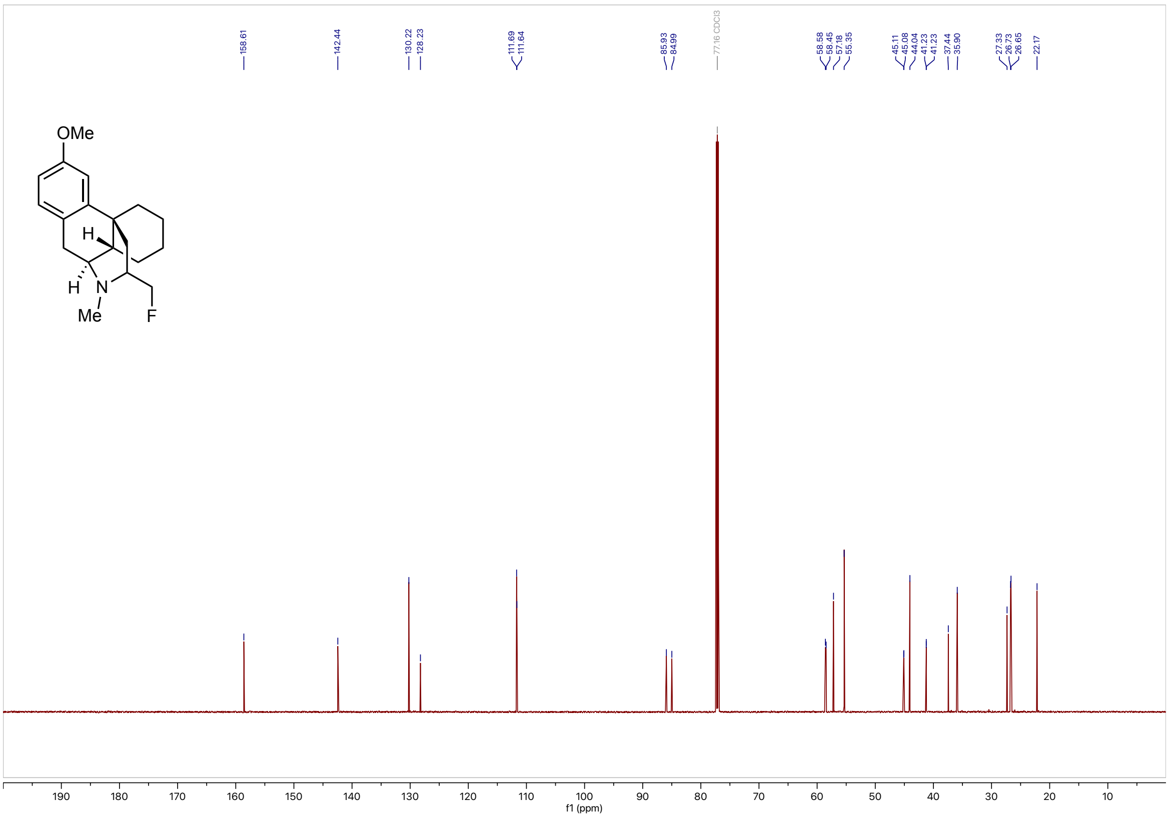

$^{19}F\{H\}$ NMR (376 MHz, $CDCl_3$) of (4bS,8aS,9S)-12-(fluoromethyl)-3-methoxy-11-methyl-6,7,8,8a,9,10-hexahydro-5H-9,4b-(epiminoethano)phenanthrene (**17d**):

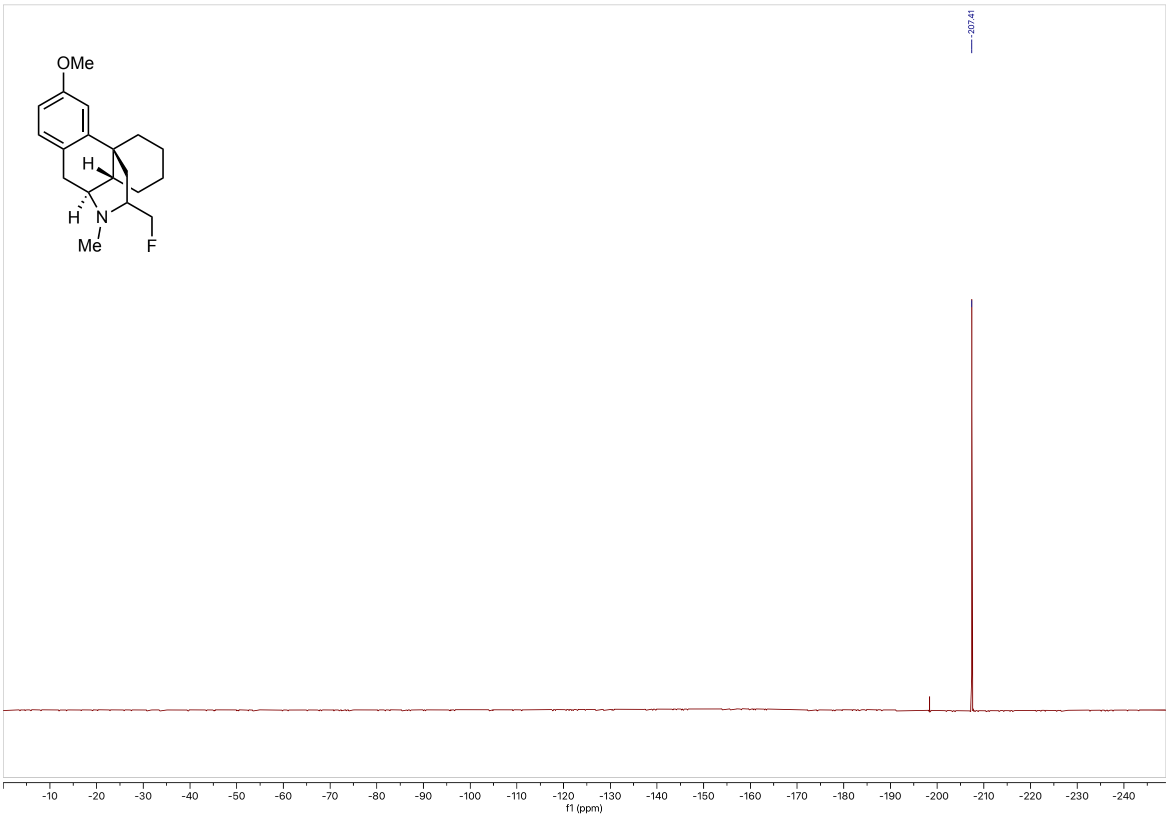

^{1}H NMR (500 MHz, $CDCl_3$) of (4bS,8aS,9S,12R)-3-methoxy-11-methyl-12-(pyridin-2-yl)-6,7,8,8a,9,10-hexahydro-5H-9,4b-(epiminoethano)phenanthrene (**17e**):

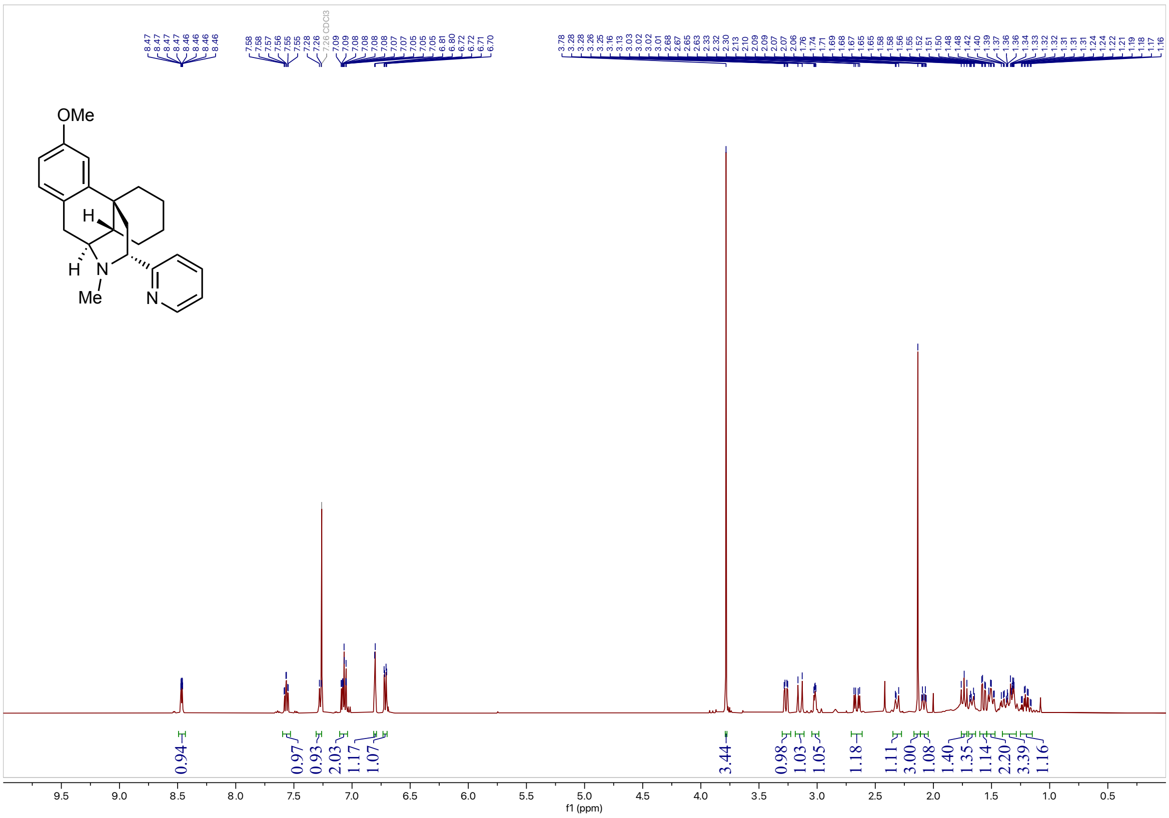

^{13}C{H} NMR (126 MHz, $CDCl_3$) of (4bS,8aS,9S,12R)-3-methoxy-11-methyl-12-(pyridin-2-yl)-6,7,8,8a,9,10-hexahydro-5H-9,4b-(epiminoethano)phenanthrene (**17e**):

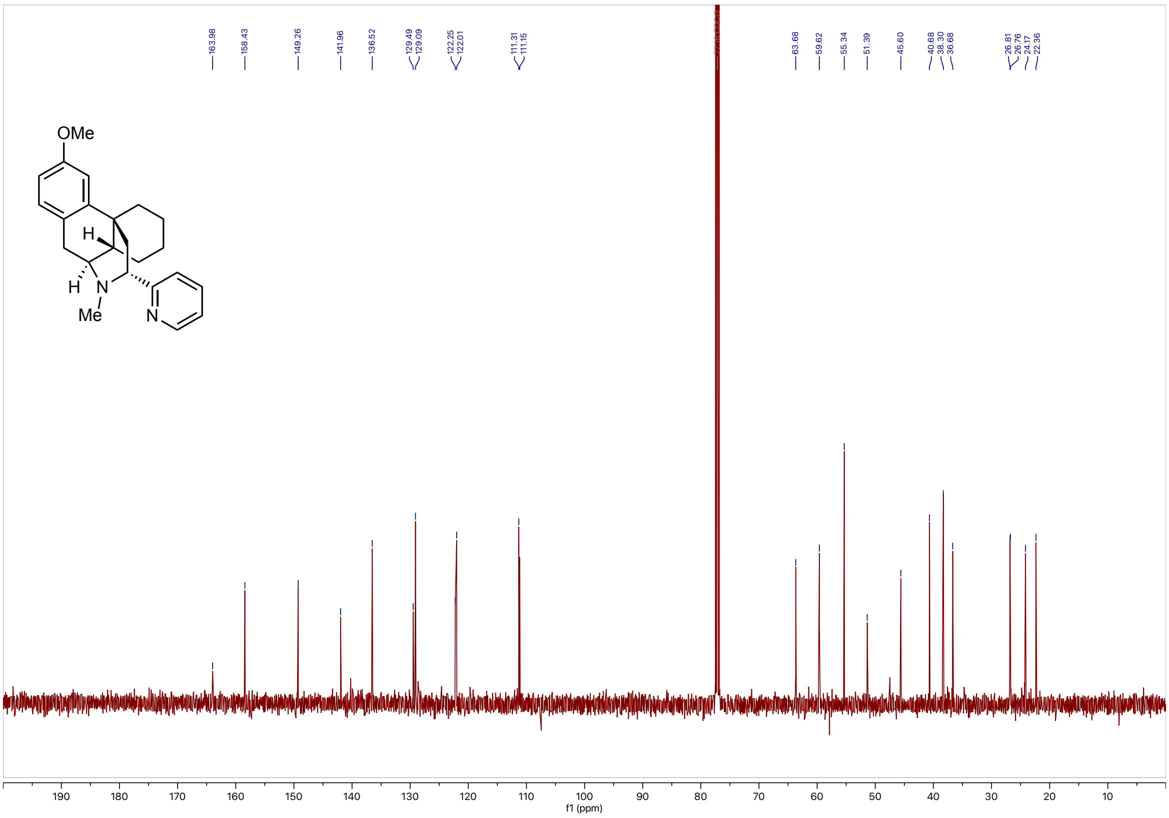

NOSEY (500 MHz, CDCl$_3$) of (*4bS,8aS,9S, 12R*)-3-methoxy-11-methyl-12-(pyridin-2-yl)-6,7,8,8a,9,10-hexahydro-5H- 9,4b-(epiminoethano)phenanthrene (**17e**):

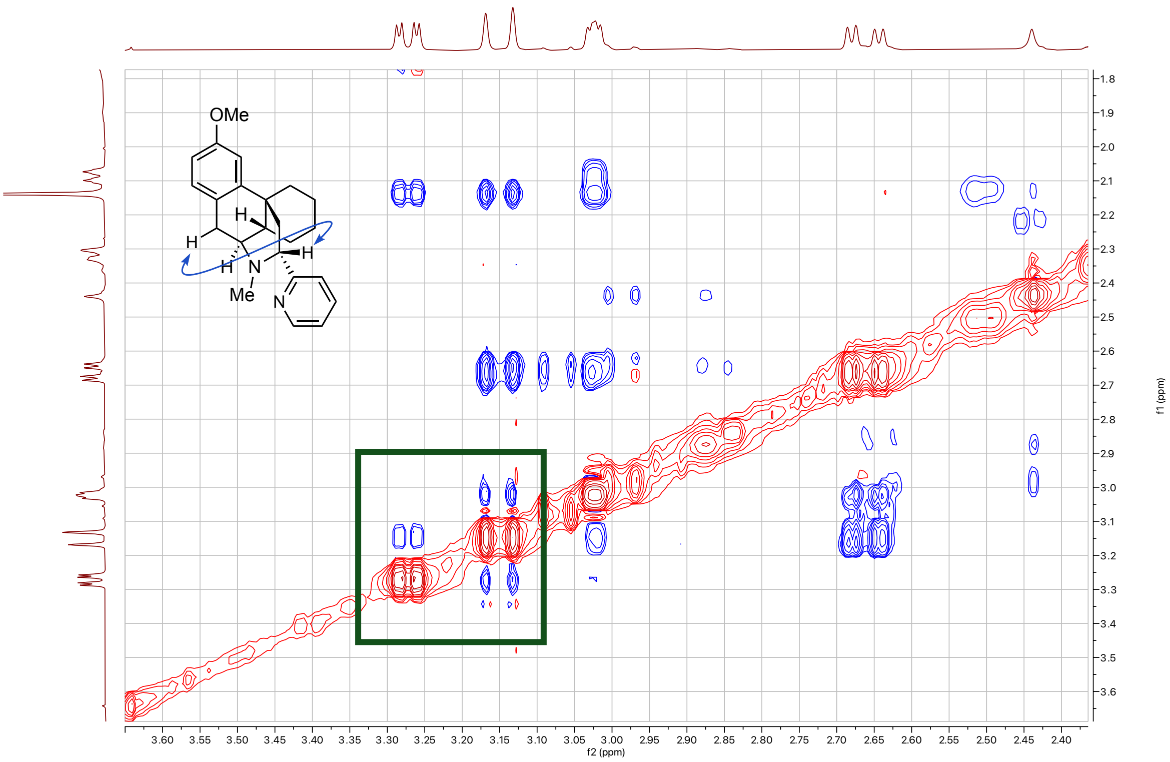

^{1}H NMR (700 MHz, $CDCl_3$) of (4bS,8aS,9S,12S)-3-methoxy-11-methyl-12-(pyridin-2-yl)-6,7,8,8a,9,10-hexahydro-5H-9,4b-(epiminoethano)phenanthrene (**17e'**):

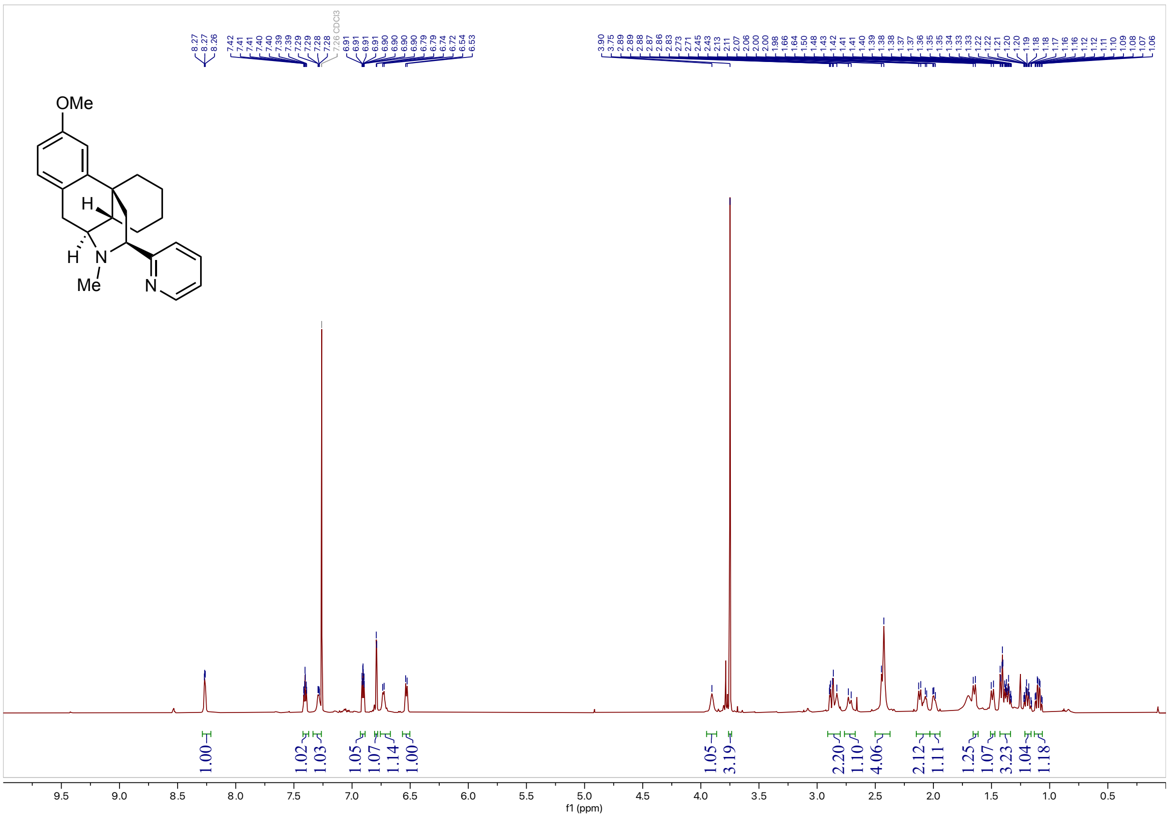

^{13}C{H} NMR (176 MHz, $CDCl_3$) of (4bS,8aS,9S,12S)-3-methoxy-11-methyl-12-(pyridin-2-yl)-6,7,8,8a,9,10-hexahydro-5H-9,4b-(epiminoethano)phenanthrene (**17e'**):

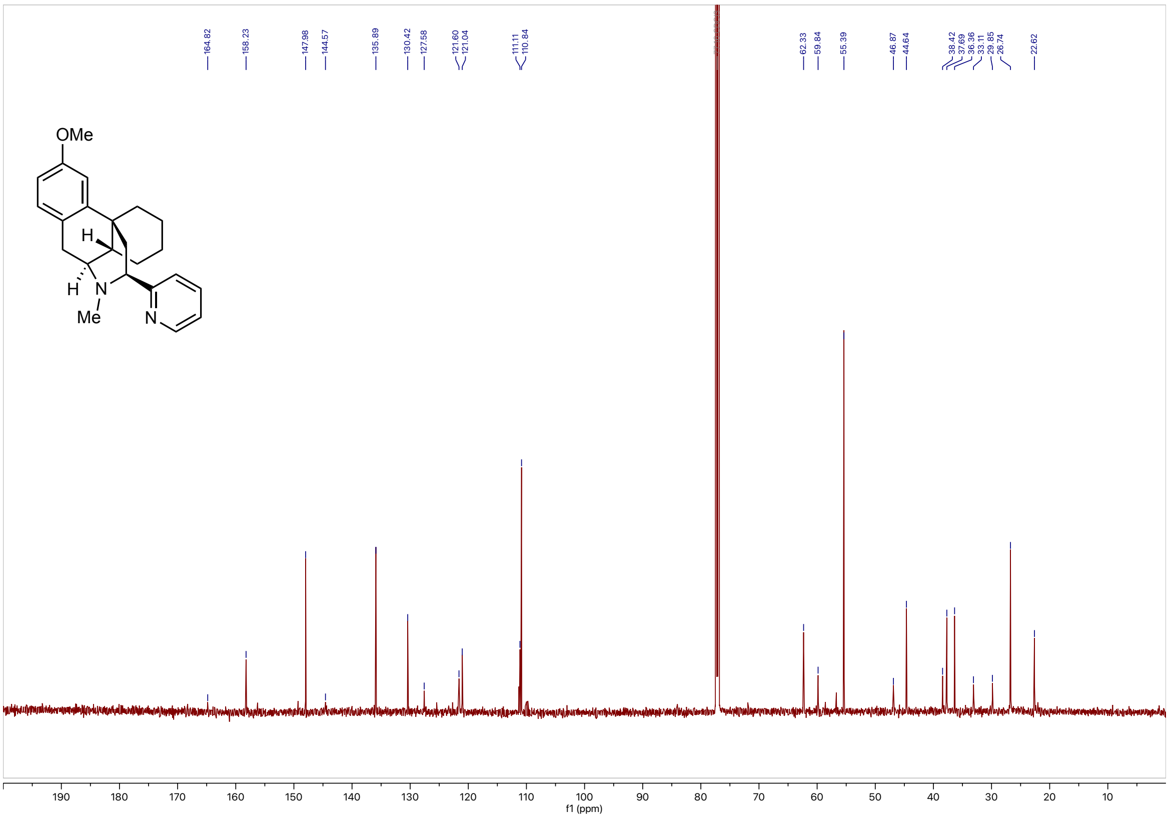

NOSEY (500 MHz, $CDCl_3$) of (*4bS,8aS,9S,12S*)-3-methoxy-11-methyl-12-(pyridin-2-yl)-6,7,8,8a,9,10-hexahydro-5H- 9,4b-(epiminoethano)phenanthrene (**17e'**):

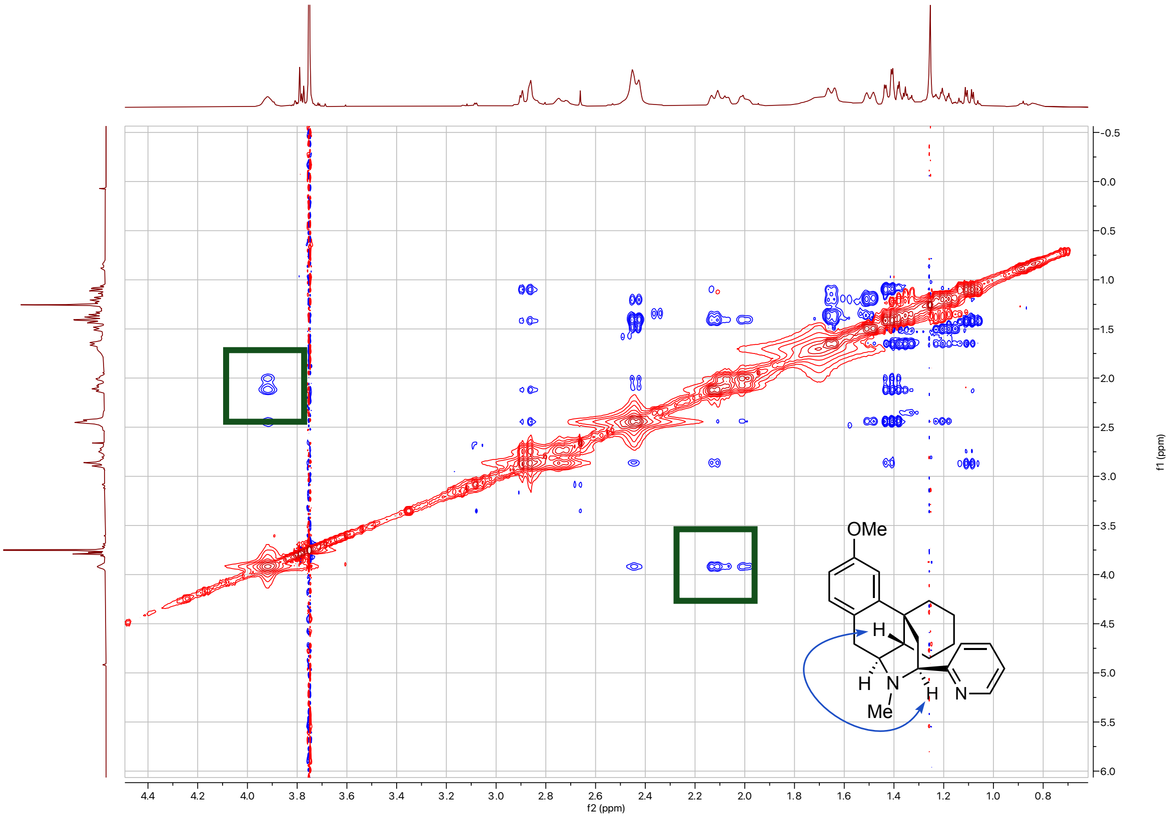

^{1}H NMR (700 MHz, $CDCl_3$) of (4bS,8aS,9S,12R)-3-methoxy-11-methyl-12-(trifluorometh-yl)-6,7,8,8a,9,10-hexahydro-5*H*-9,4b-(epiminoethano)phenanthrene (**17f**):

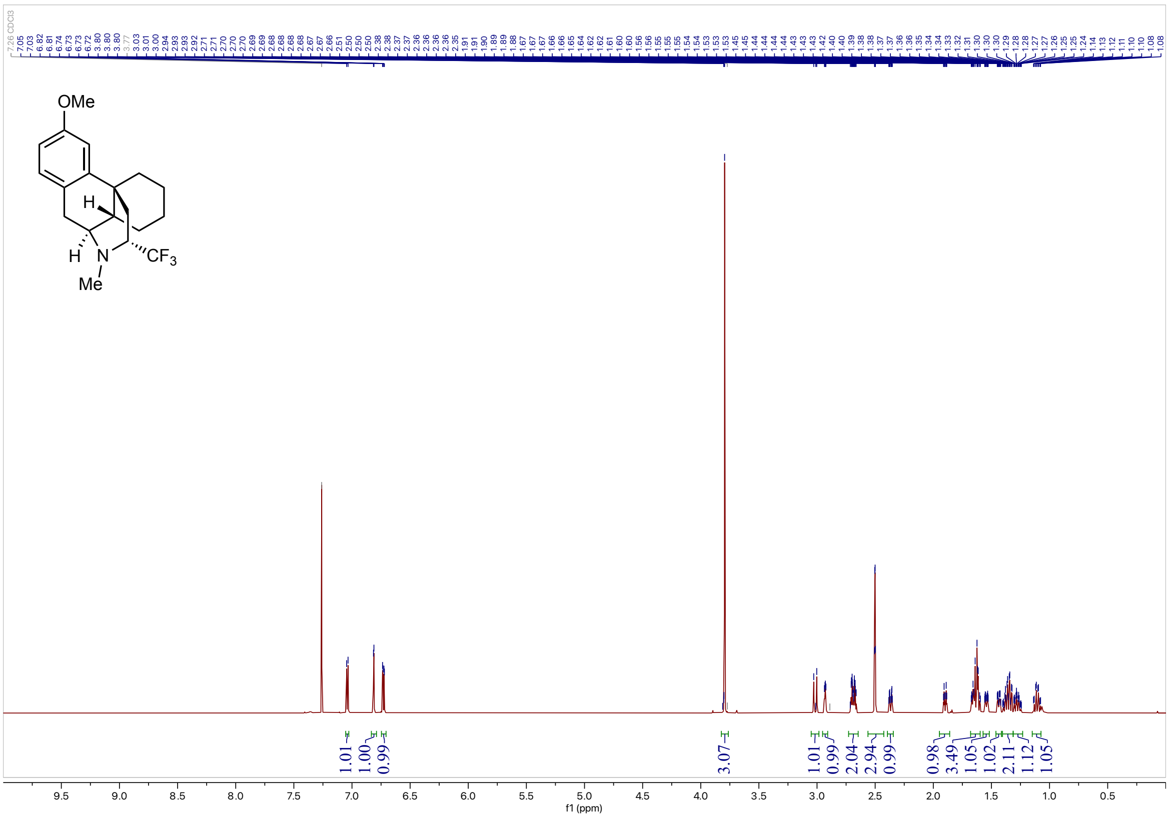

$^{13}C\{H\}$ NMR (176 MHz, $CDCl_3$) of *(4bS,8aS,9S,12R)-3-methoxy-11-methyl-12-(trifluoro meth-yl)-6,7,8,8a,9,10-hexahydro-5H-9,4b-(epiminoethano)phenanthrene* (**17f**):

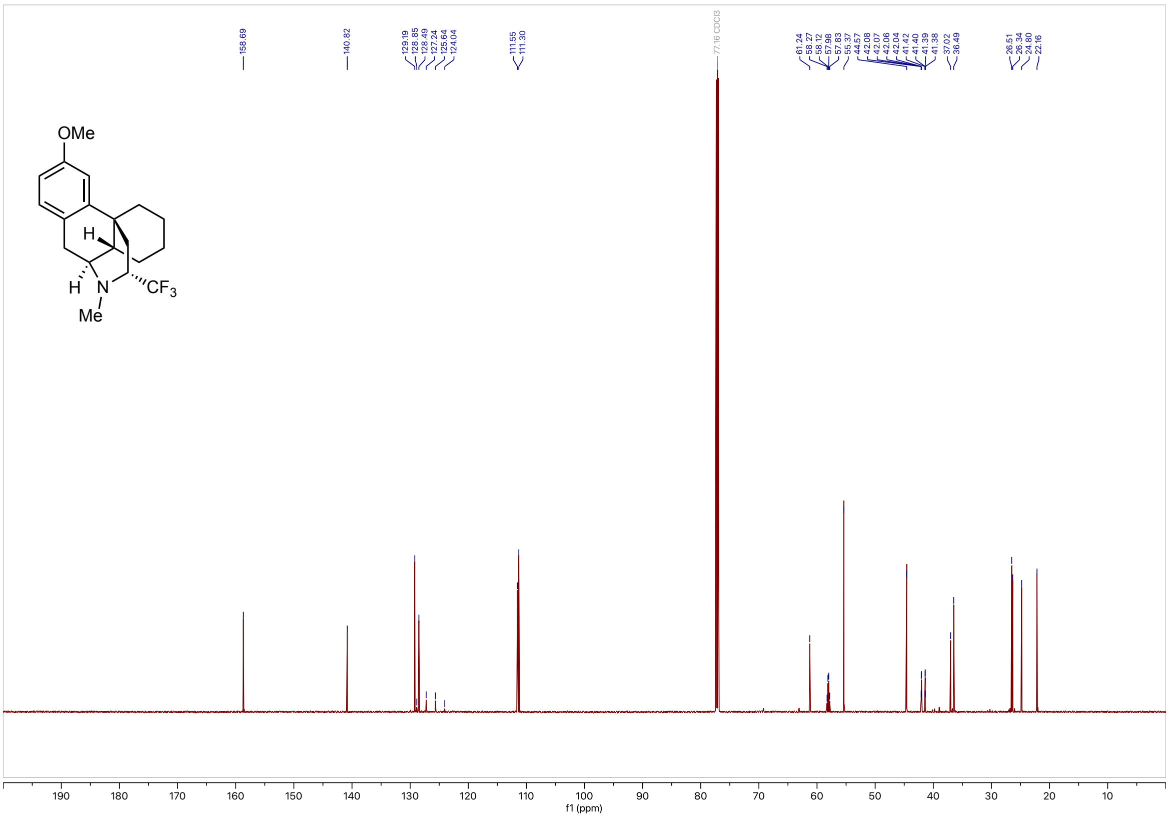

^{19}F{H} NMR (471 MHz, $CDCl_3$) of *(4bS,8aS,9S,12R)-3-methoxy-11-methyl-12-(trifluoro meth-yl)-6,7,8,8a,9,10-hexahydro-5H-9,4b-(epiminoethano)phenanthrene* (**17f**):

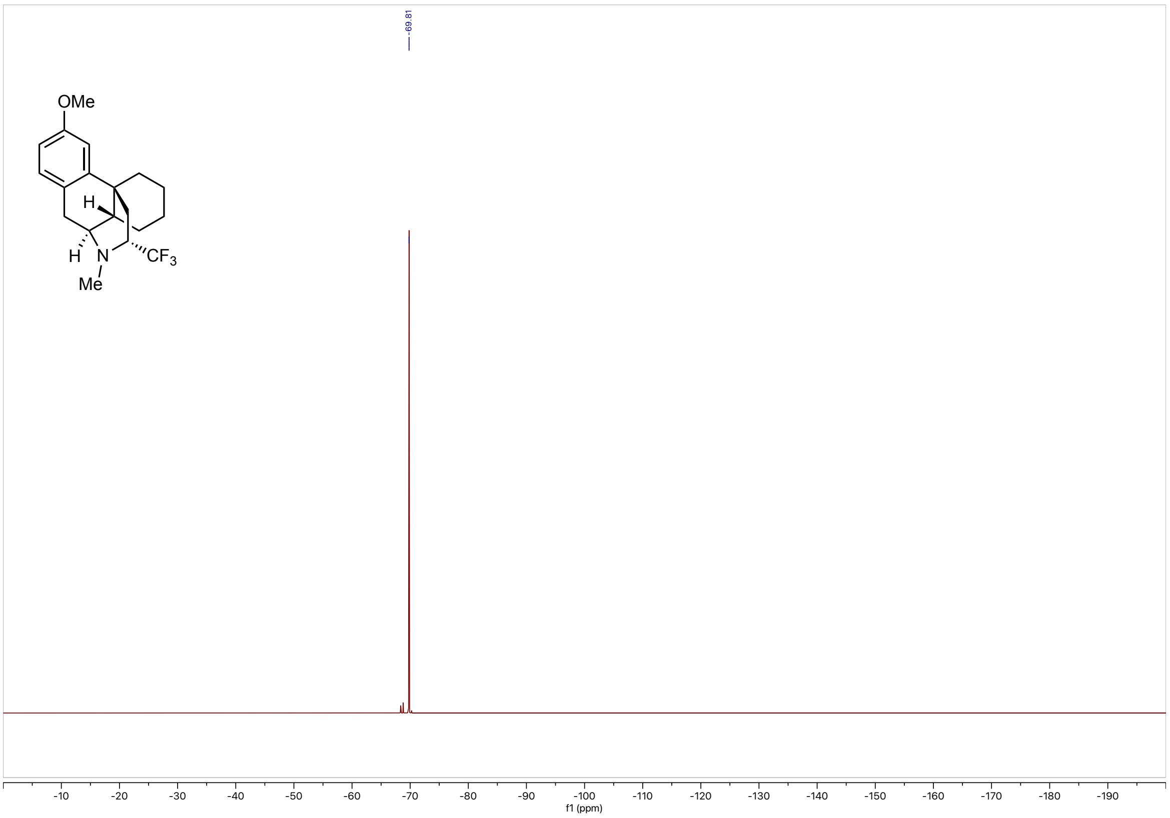

NOSEY (500 MHz, $CDCl_3$) of (*4bS,8aS,9S,12R*)-3-methoxy-11-methyl-12-(trifluoro methyl)-6,7,8,8a,9,10-hexahydro-5H-9,4b-(epiminoethano)phenanthrene (**17f**):

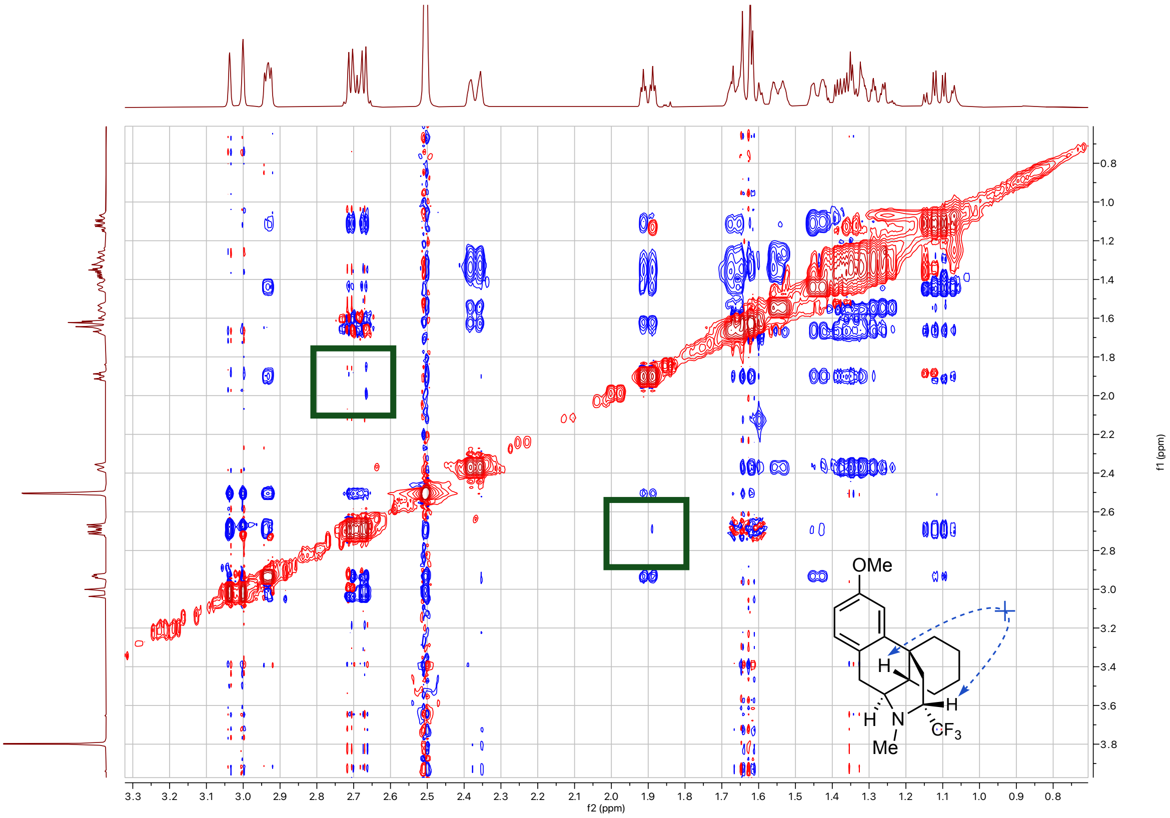

^{1}H NMR (700 MHz, $CDCl_3$) of *(4bS,8aS,9S,12S)-3-methoxy-11-methyl-12-(trifluorometh-yl)-6,7,8,8a,9,10-hexahydro-5H-9,4b-(epiminoethano)phenanthrene* (**17f'**):

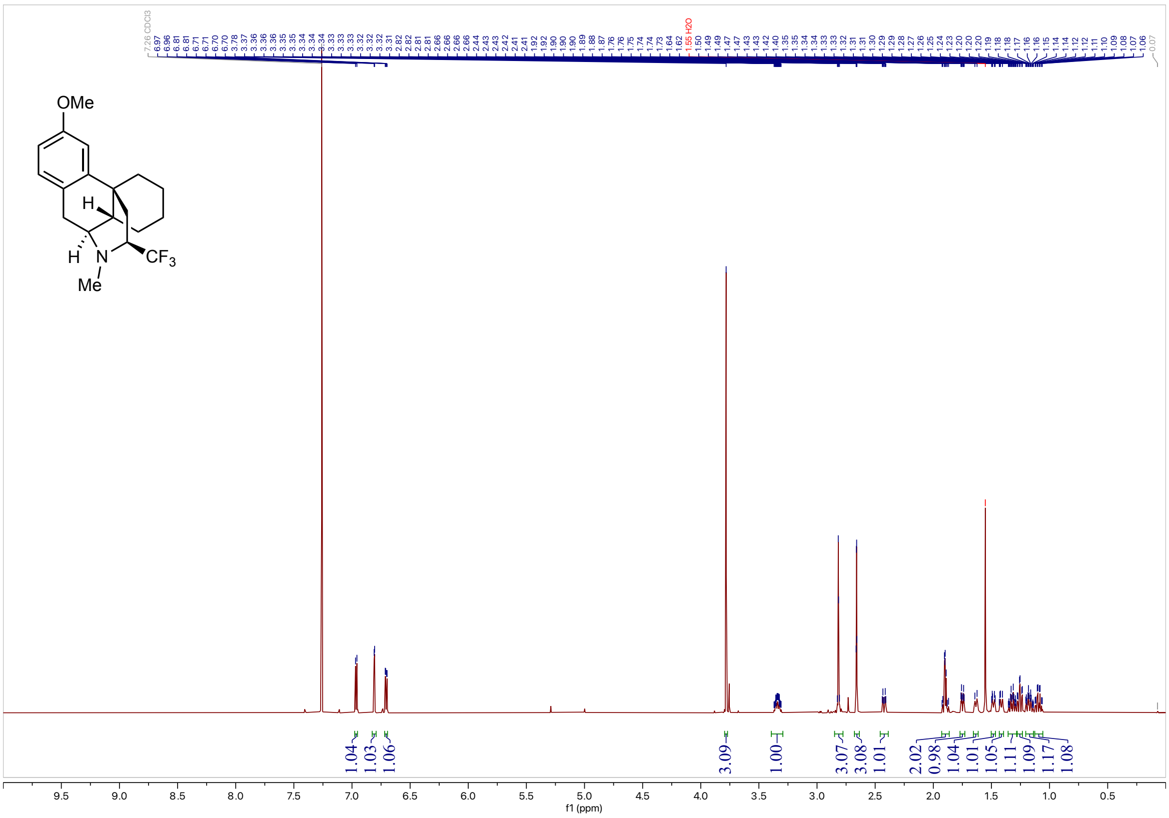

^{13}C{H} NMR (700 MHz, $CDCl_3$) of *(4bS,8aS,9S,12S)-3-methoxy-11-methyl-12-(trifluoro meth-yl)-6,7,8,8a,9,10-hexahydro-5H-9,4b-(epiminoethano)phenanthrene* (**17f'**):

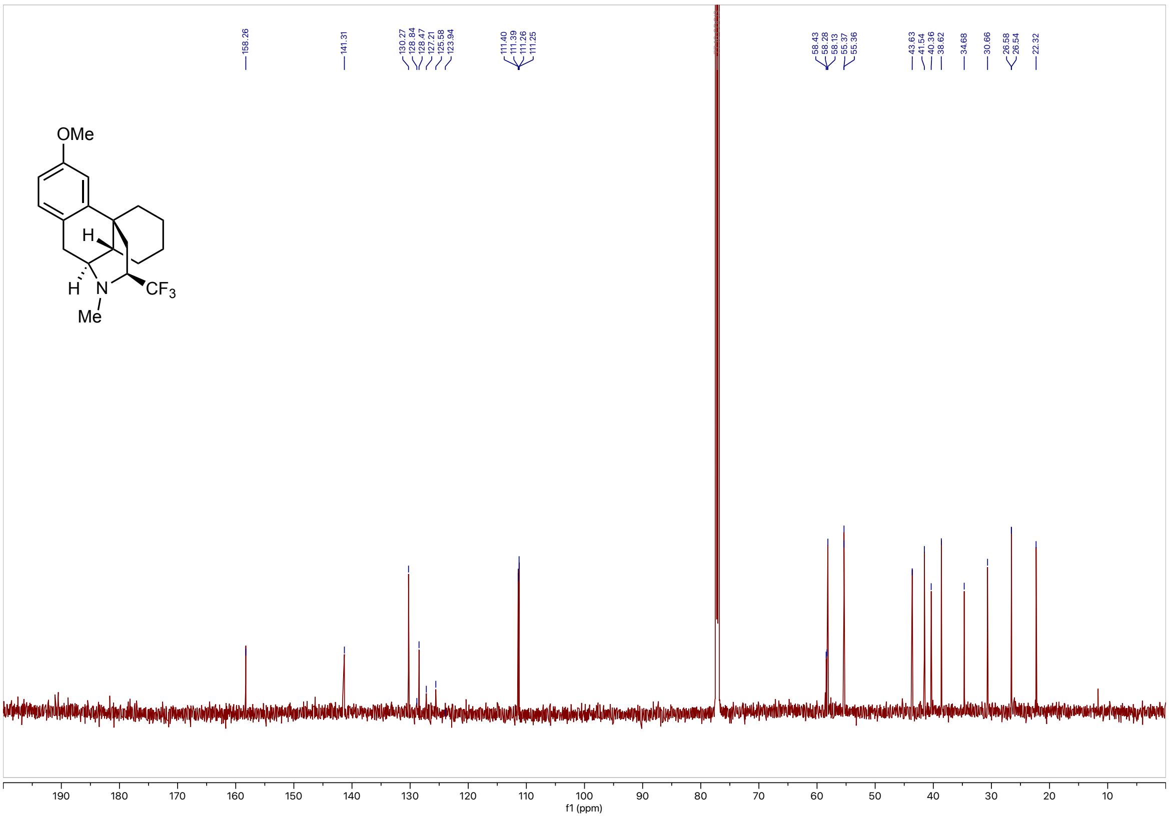

^{19}F{H} NMR (471 MHz, $CDCl_3$) of *(4bS,8aS,9S,12S)-3-methoxy-11-methyl-12-(trifluoro meth-yl)-6,7,8,8a,9,10-hexahydro-5H-9,4b-(epiminoethano)phenanthrene* (**17f'**):

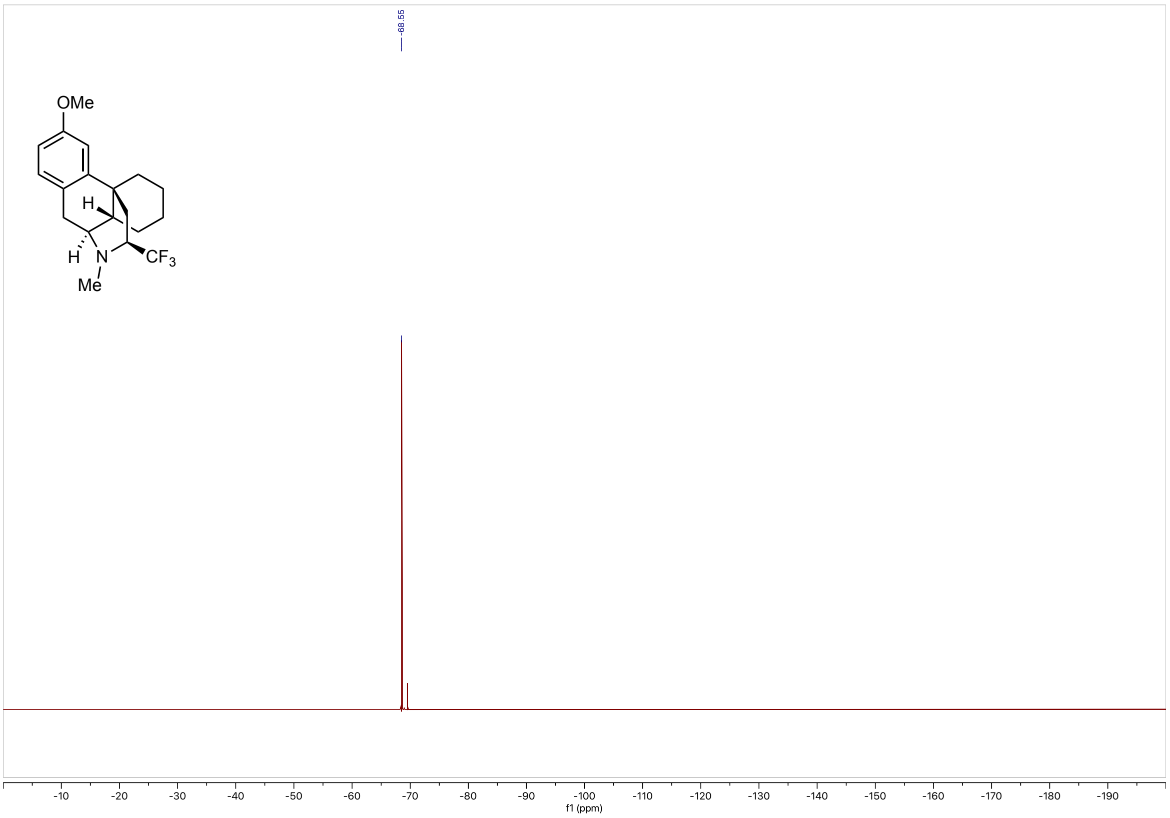

NOSEY (500 MHz, $CDCl_3$) of (*4bS,8aS,9S,12S*)-3-methoxy-11-methyl-12-(trifluoro-methyl)-6,7,8,8a,9,10-hexahydro-5*H*-9,4b-(epiminoethano)phenanthrene (**17f'**):

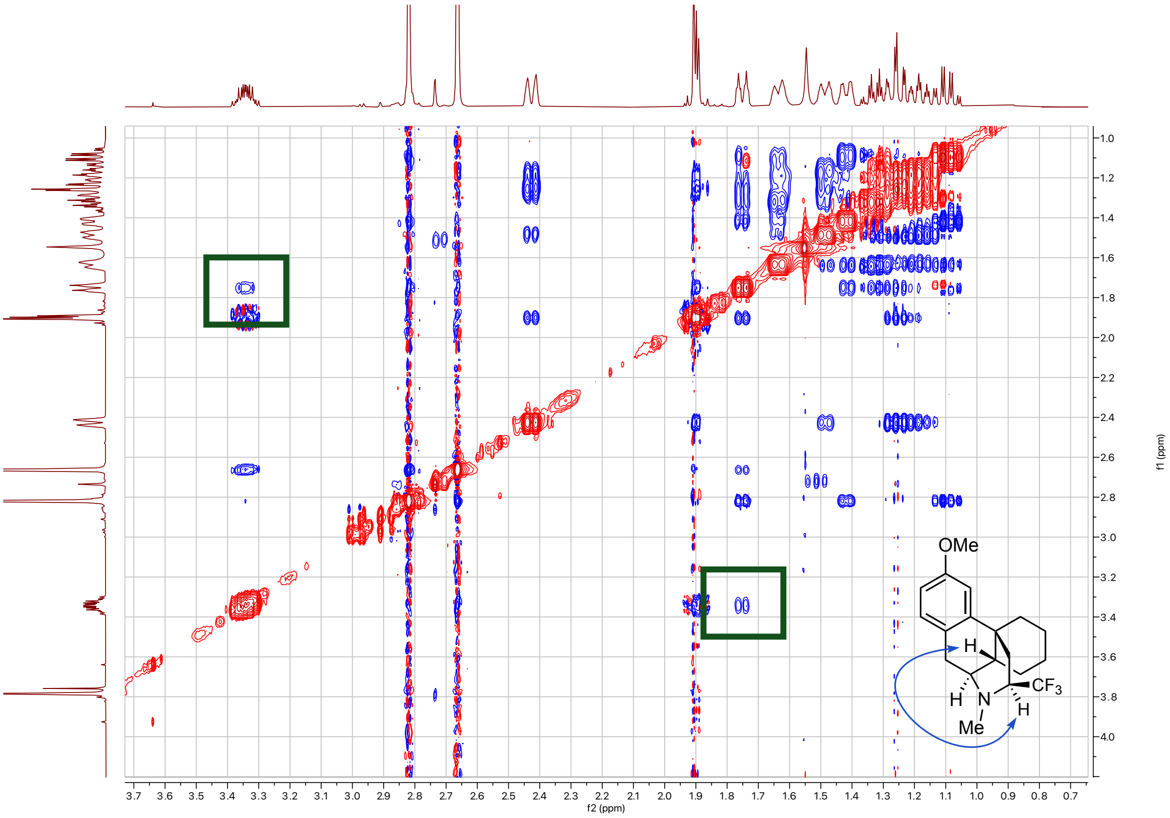

^{1}H NMR (700 MHz, $CDCl_3$) of 1-(2-((4-chlorophenyl)(phenyl)methoxy)ethyl)-2-(trifluoromethyl)piperidine (**18**):

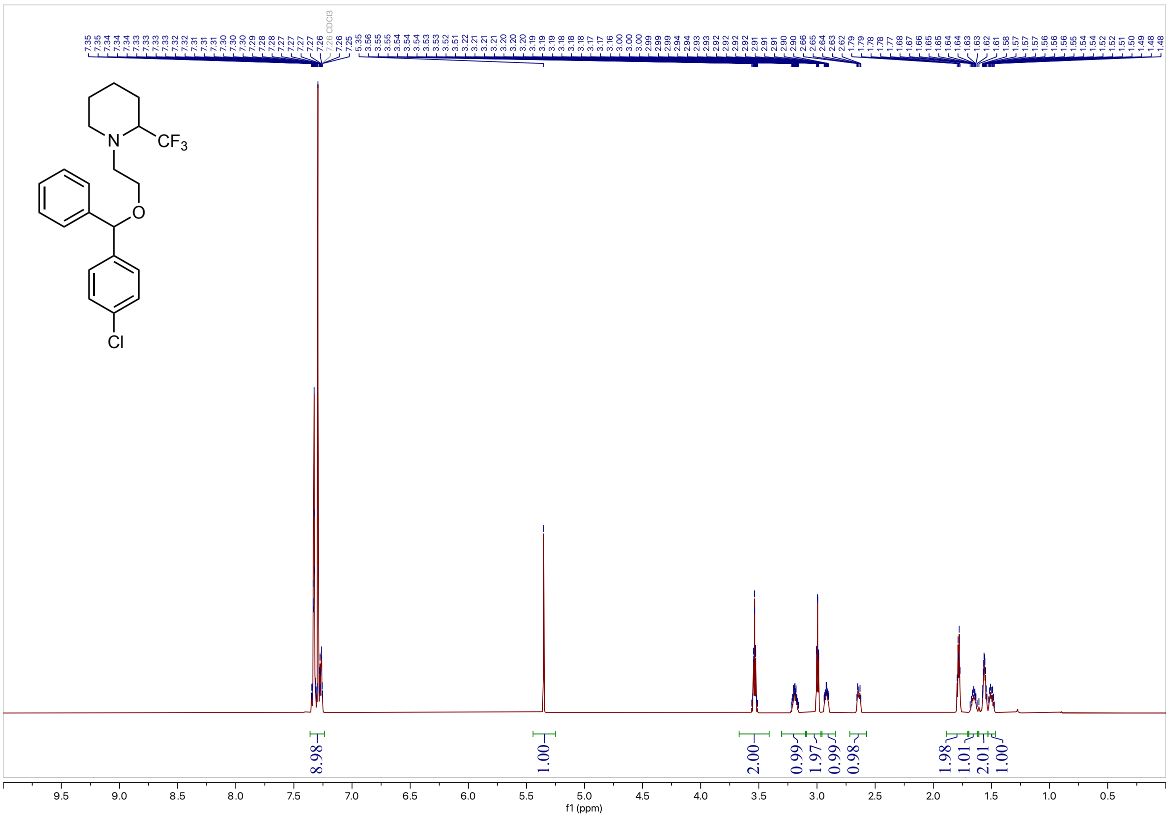

^{13}C{H} NMR (176 MHz, $CDCl_3$) of 1-(2-((4-chlorophenyl)(phenyl)methoxy)ethyl)-2-(trifluoromethyl)piperidine (**18**):

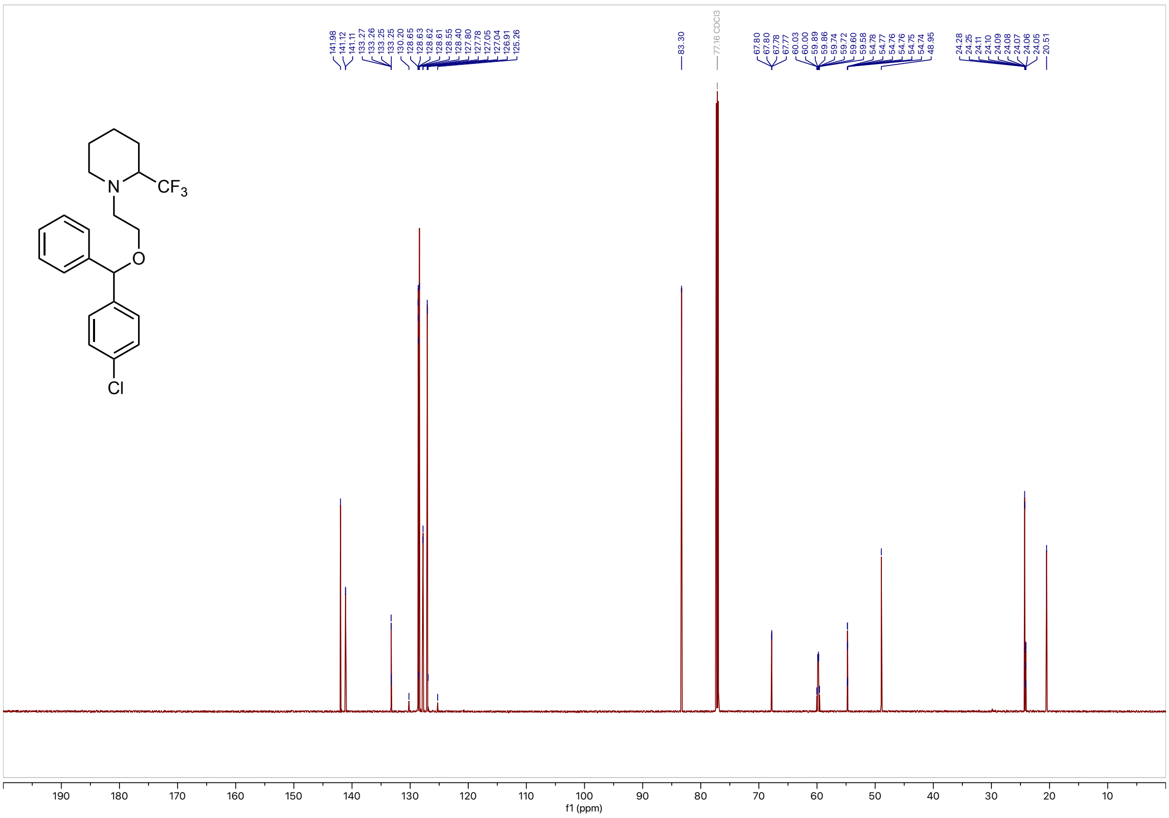

$^{19}F\{H\}$ NMR (471 MHz, $CDCl_3$) of 1-(2-((4-chlorophenyl)(phenyl)methoxy)ethyl)-2-(trifluoromethyl)piperidine (**18**):

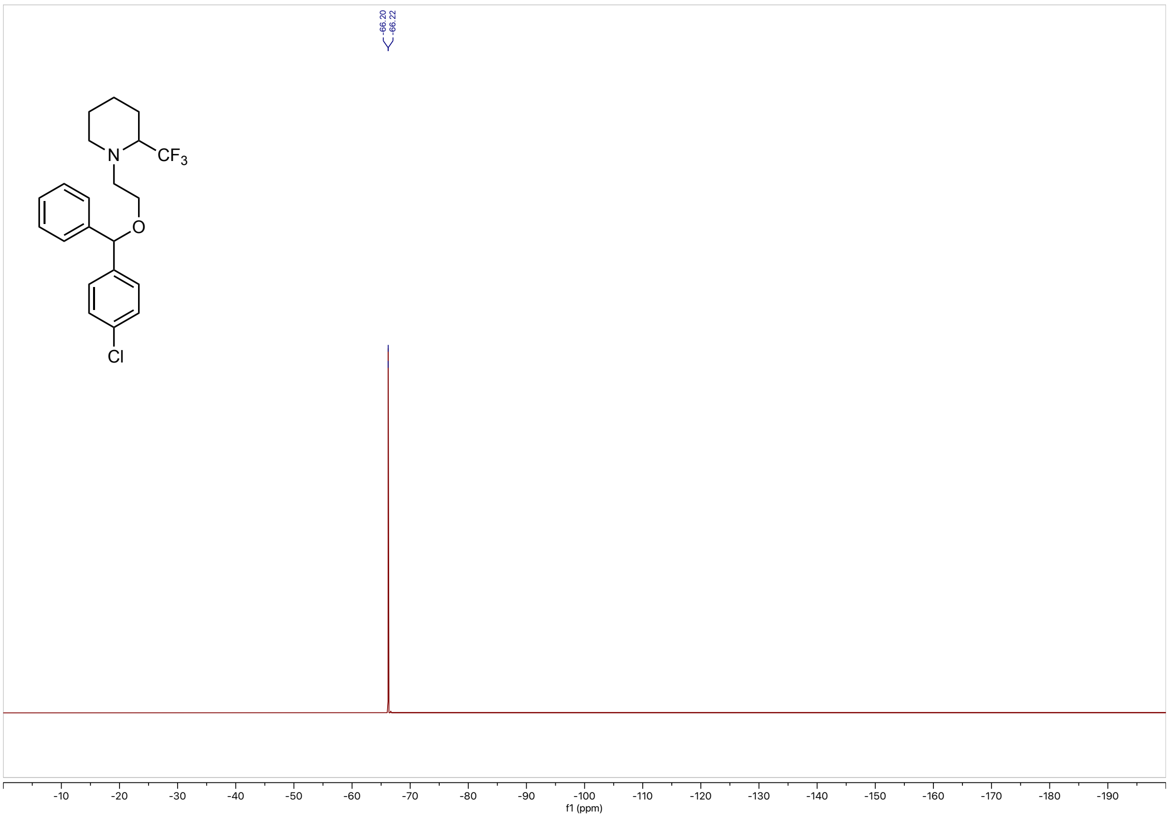

^{1}H NMR (700 MHz, $CDCl_3$) of 5-(2-chlorobenzyl)-4-(trifluoromethyl)-4,5,6,7-tetrahydrothieno[3,2-c]pyridine (**19**):

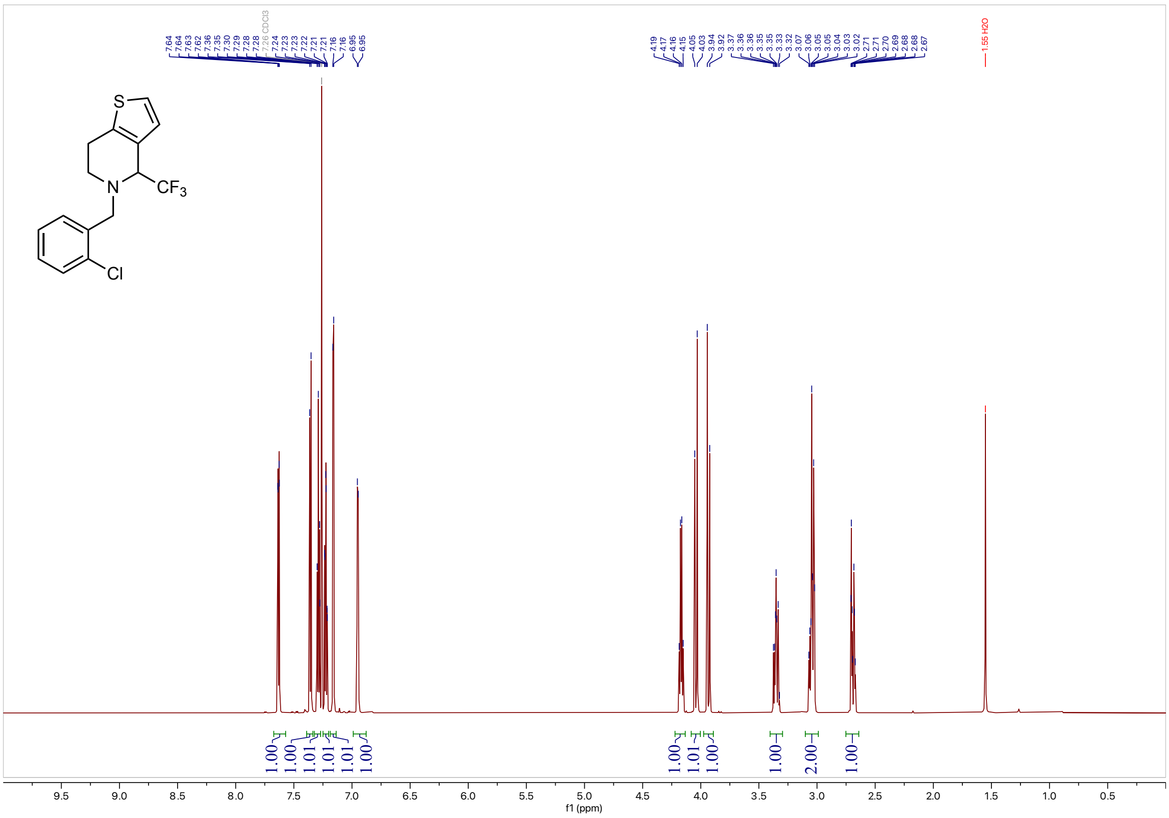

$^{13}C\{H\}$ NMR (176 MHz, $CDCl_3$) of 5-(2-chlorobenzyl)-4-(trifluoromethyl)-4,5,6,7-tetrahydrothieno[3,2-c]pyridine (**19**):

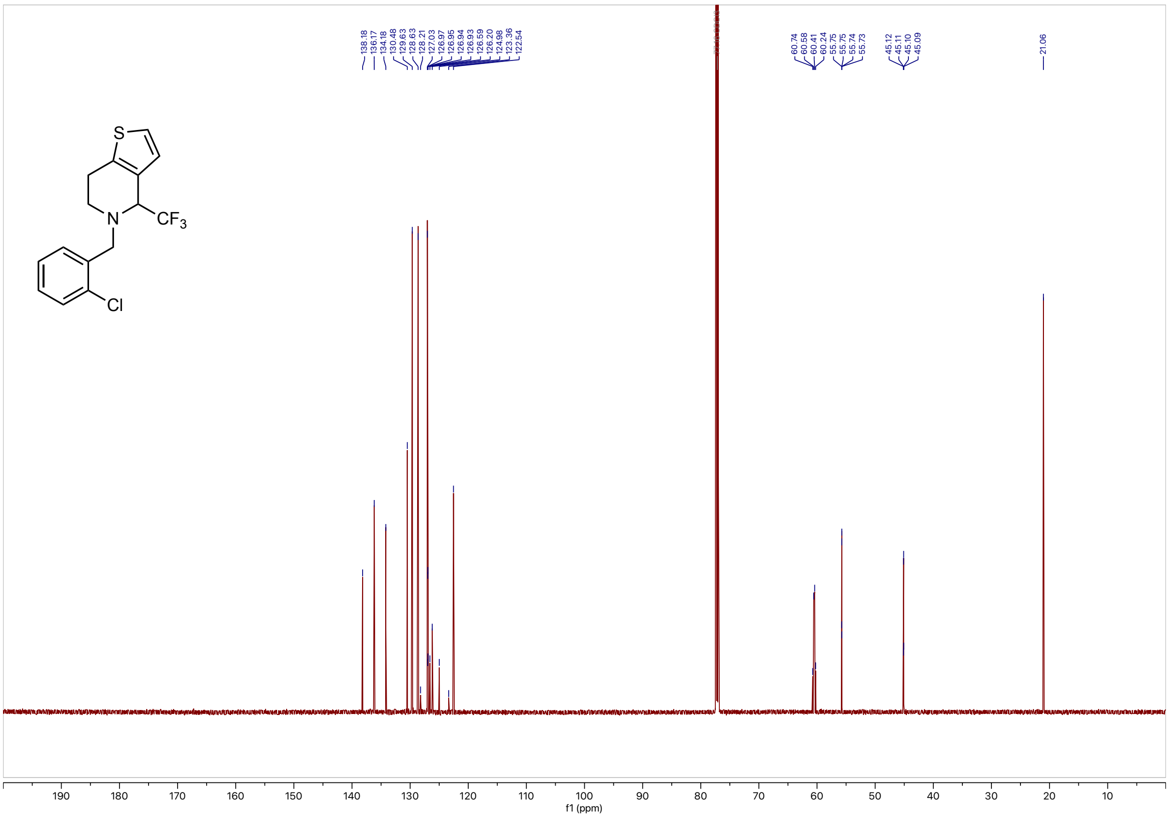

$^{19}F\{H\}$ NMR (471 MHz, $CDCl_3$) of 5-(2-chlorobenzyl)-4-(trifluoromethyl)-4,5,6,7-tetrahydrothieno[3,2-c]pyridine (**19**):

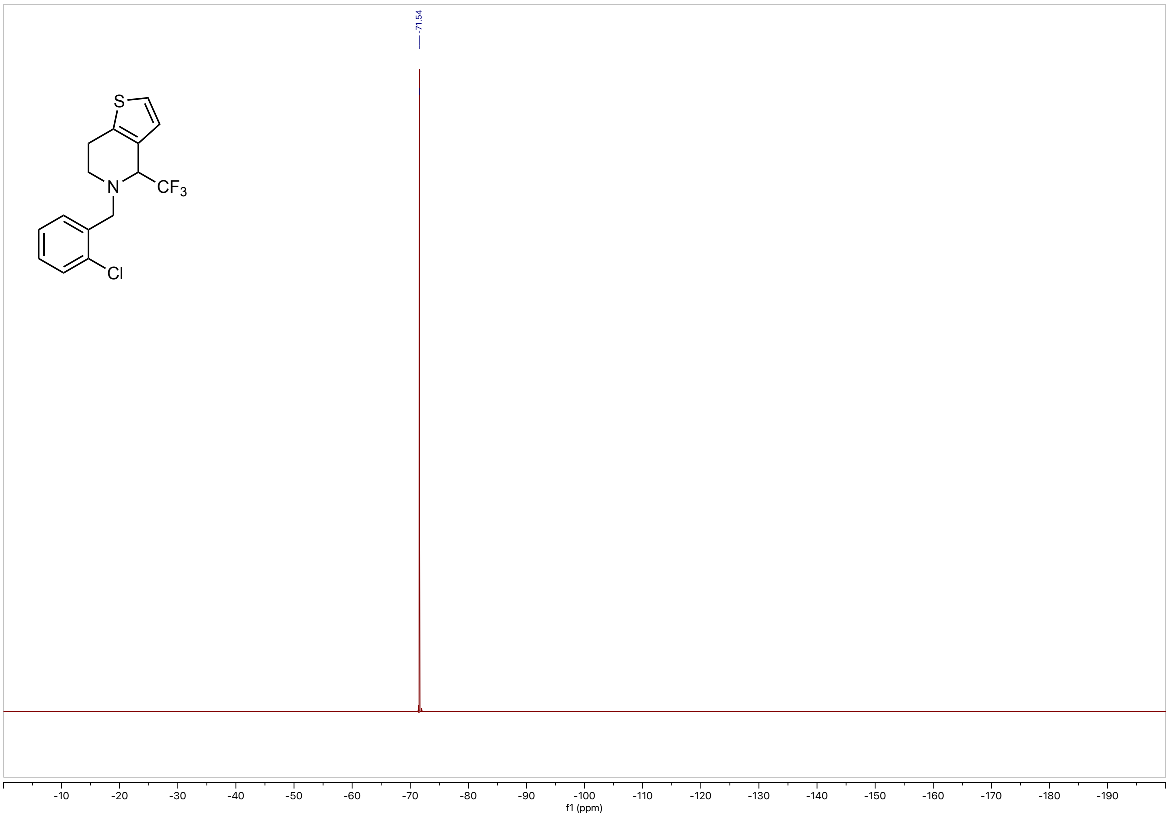

^{1}H NMR (700 MHz, $CDCl_3$) of *(4^{1}R,7aS,13aR,13bR)-4^{1}*-(trifluoromethyl)dodecahydro-1H,5*H*,10*H*-dipyrido[2,1-f:3',2',1'-ij][1,6]naphthyridin-10-one (**20A**)

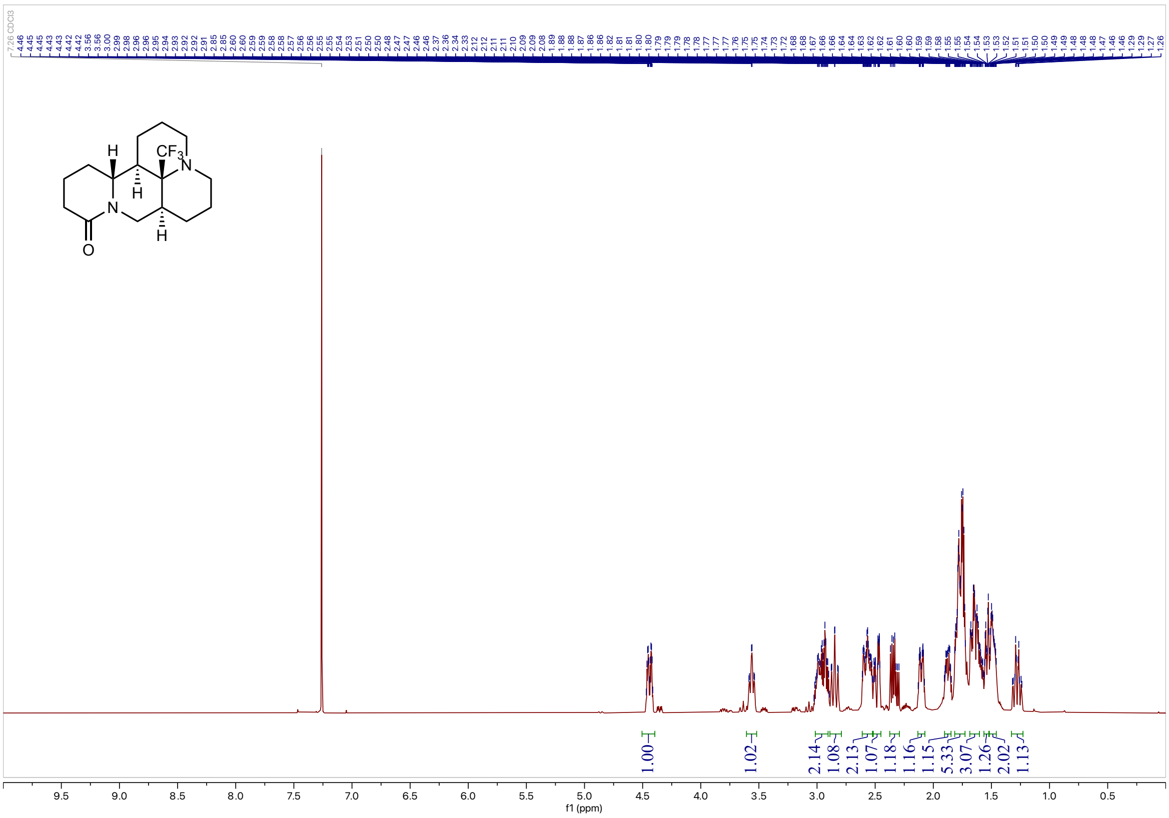

^{13}C{H} NMR (176 MHz, $CDCl_3$) of *(4^{1}R,7aS,13aR,13bR)-4^{1}*-(trifluoromethyl)dodecahydro-1H,5*H*,10*H*-dipyrido[2,1-f:3',2',1'-ij][1,6]naphthyridin-10-one (**20A**):

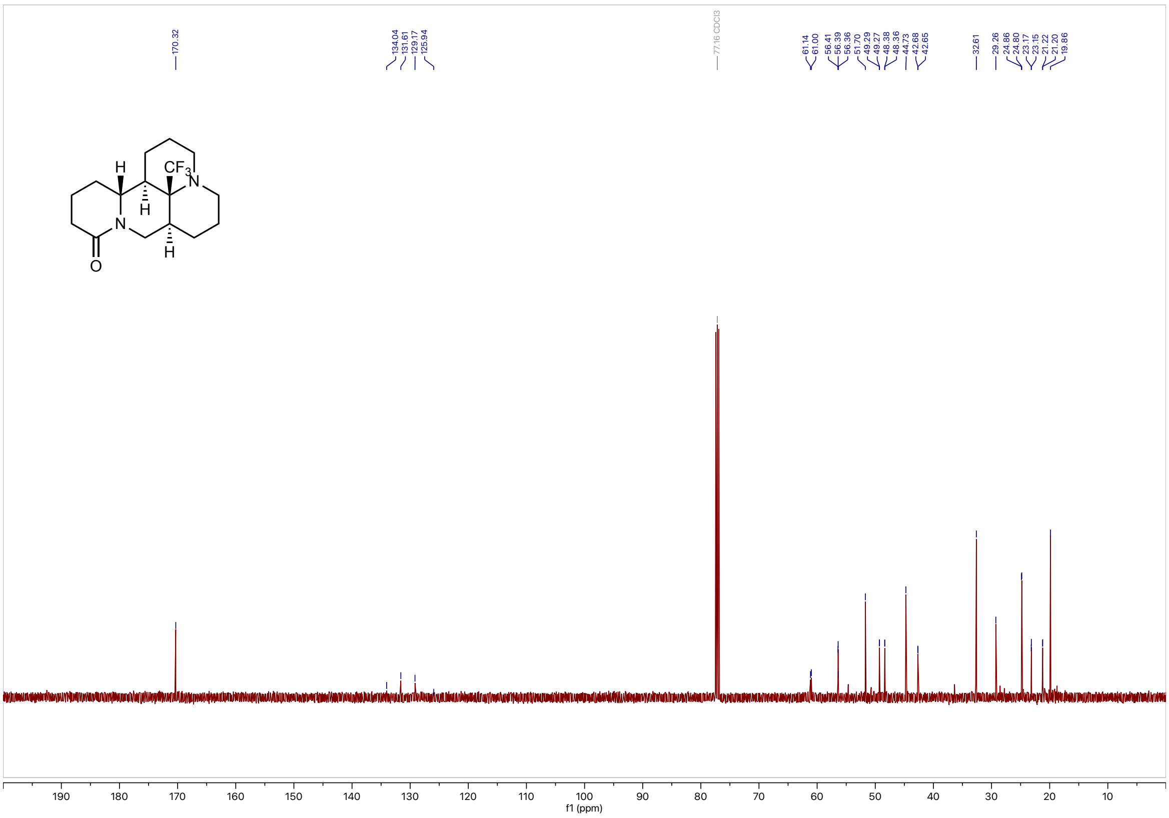

$^{19}F\{H\}$ NMR (471 MHz, $CDCl_3$) of *(4[1]R,7aS,13aR,13bR)-4[1]*-(trifluoromethyl)dodecahydro-1H,5*H*,10*H*-dipyrido[2,1-f:3',2',1'-ij][1,6]naphthyridin-10-one (**20A**)

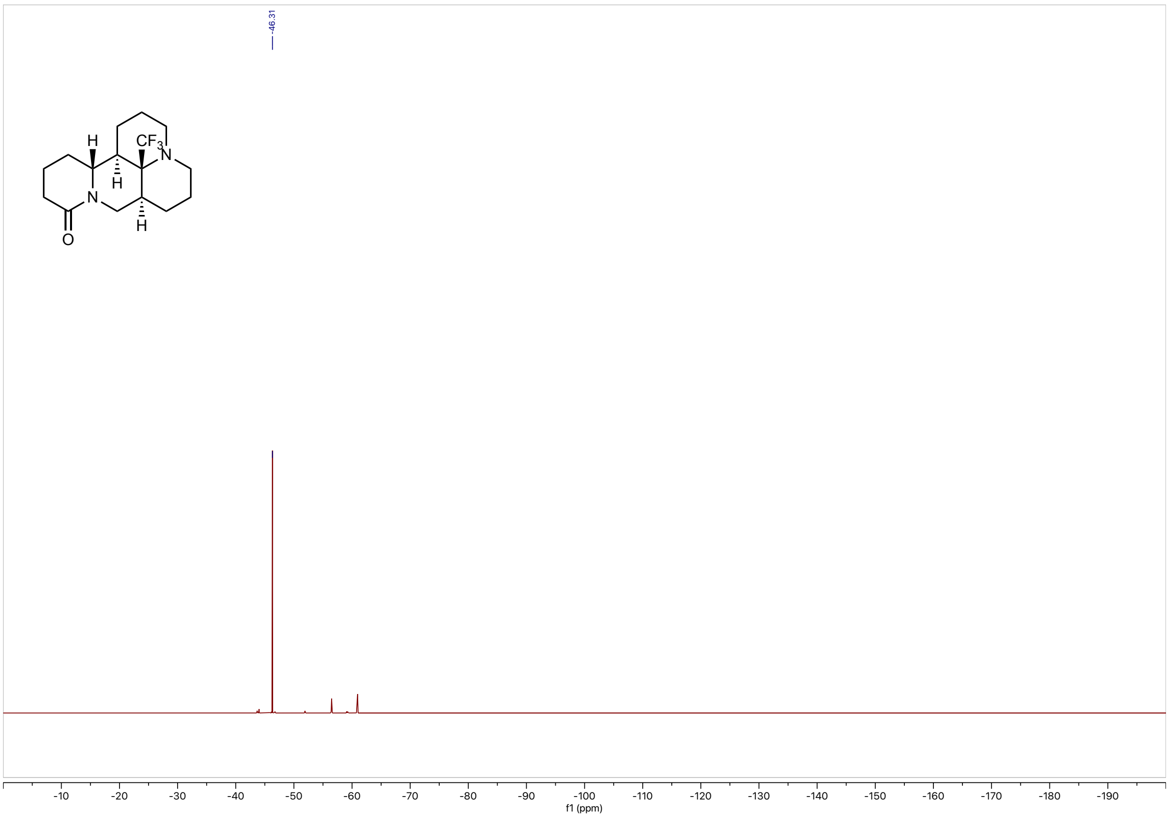

Heteronuclear ^{19}F-^{1}H NOSEY (500 MHz, $CDCl_3$) of *(4[1]R,7aS,13aR,13bR)-4[1]*-(trifluoro methyl)dodecahydro-1H,5*H*,10*H*-dipyrido[2,1-f:3',2',1'-ij][1,6]naphthyridin-10-one (**20A**)

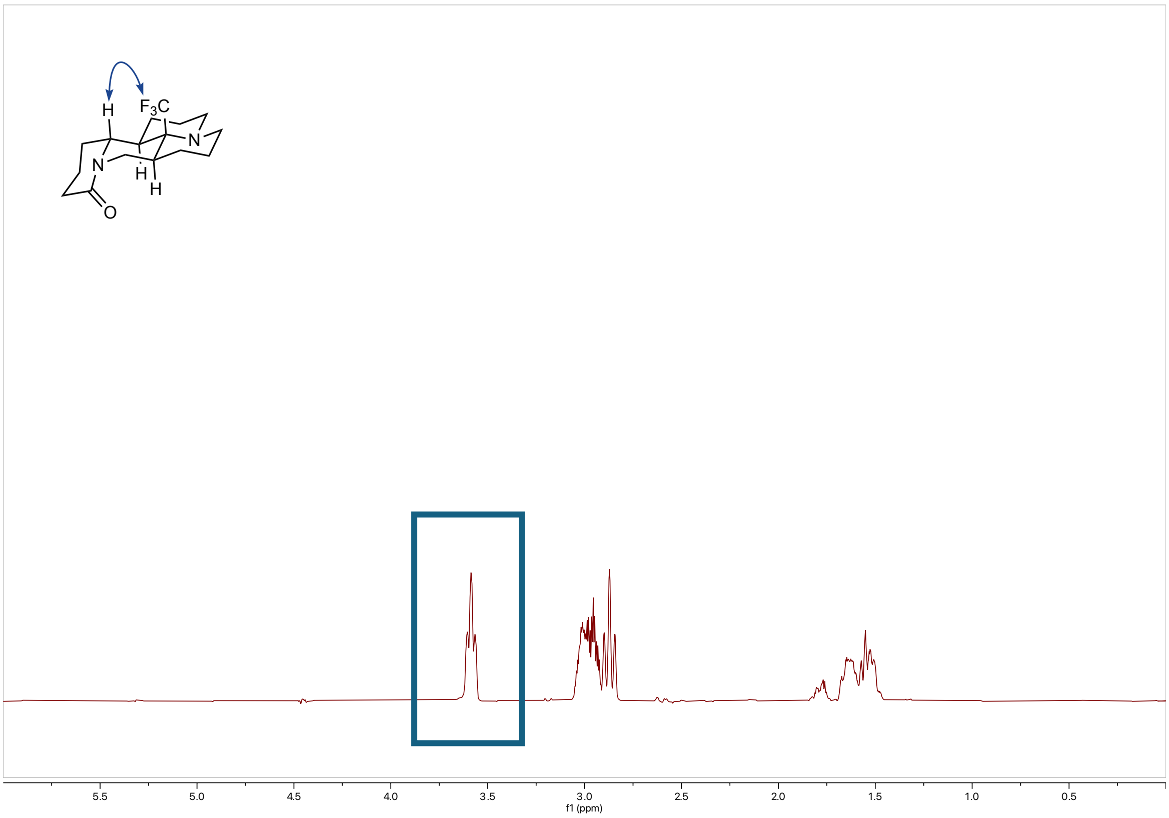

^{1}H NMR (500 MHz, $CDCl_3$) of (4[1]*S*,7a*S*,13a*R*,13b*R*)-4[1]-(trifluoromethyl)dodecahydro-1H,5*H*,10*H*-dipyrido[2,1-f:3',2',1'-ij][1,6]naphthyridin-10-one (**20B**)

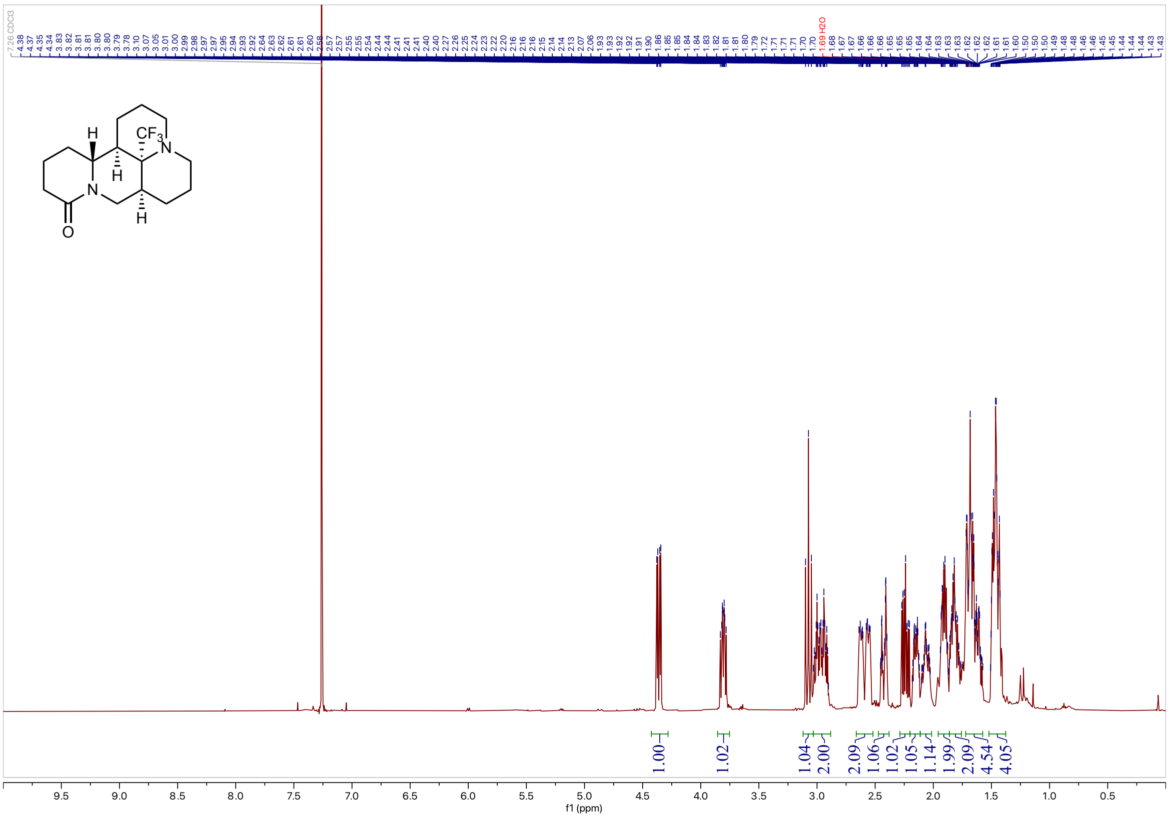

^{13}C{H} NMR (126 MHz, $CDCl_3$) of (4[1]*S*,7a*S*,13a*R*,13b*R*)-4[1]-(trifluoromethyl)dodecahydro-1H,5*H*,10*H*-dipyrido[2,1-f:3',2',1'-ij][1,6]naphthyridin-10-one (**20B**)

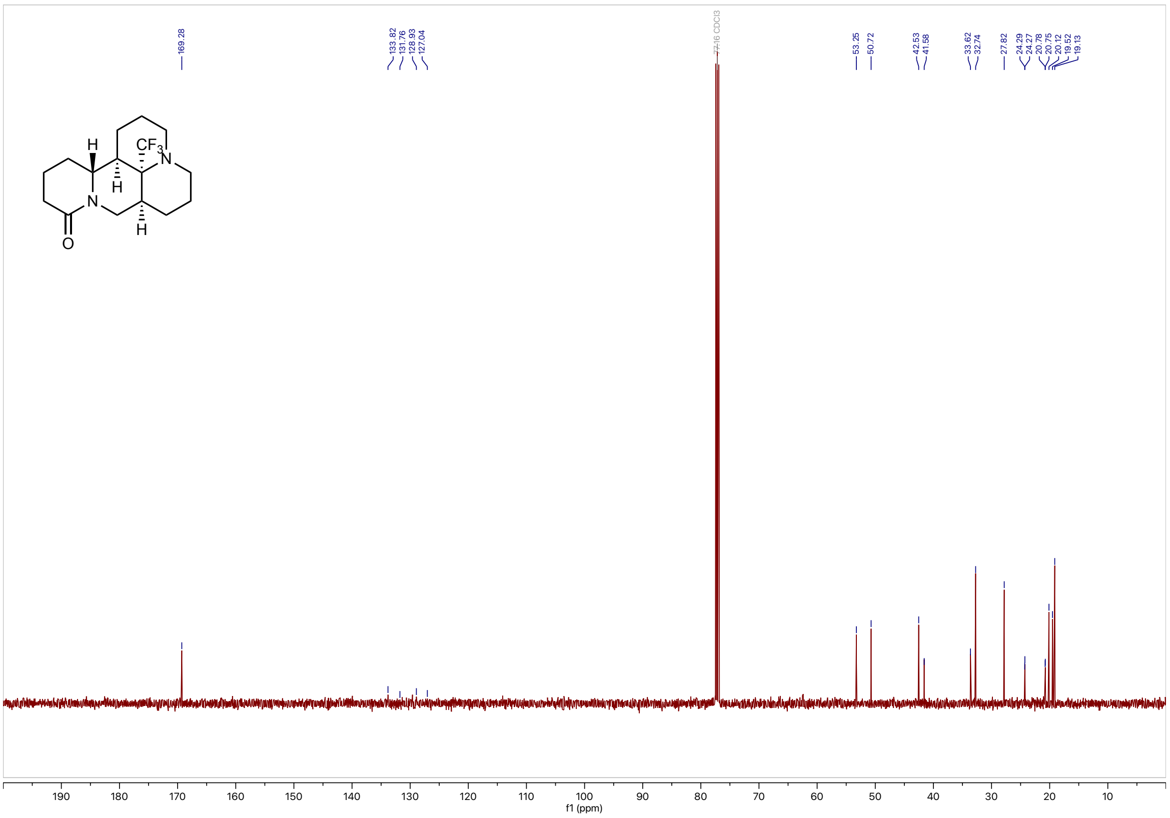

$^{19}F\{H\}$ NMR (471 MHz, $CDCl_3$) of (4[1]*S*,7a*S*,13a*R*,13b*R*)-4[1]-(trifluoromethyl)dodecahydro-1H,5*H*,10*H*-dipyrido[2,1-f:3',2',1'-ij][1,6]naphthyridin-10-one (**20B**)

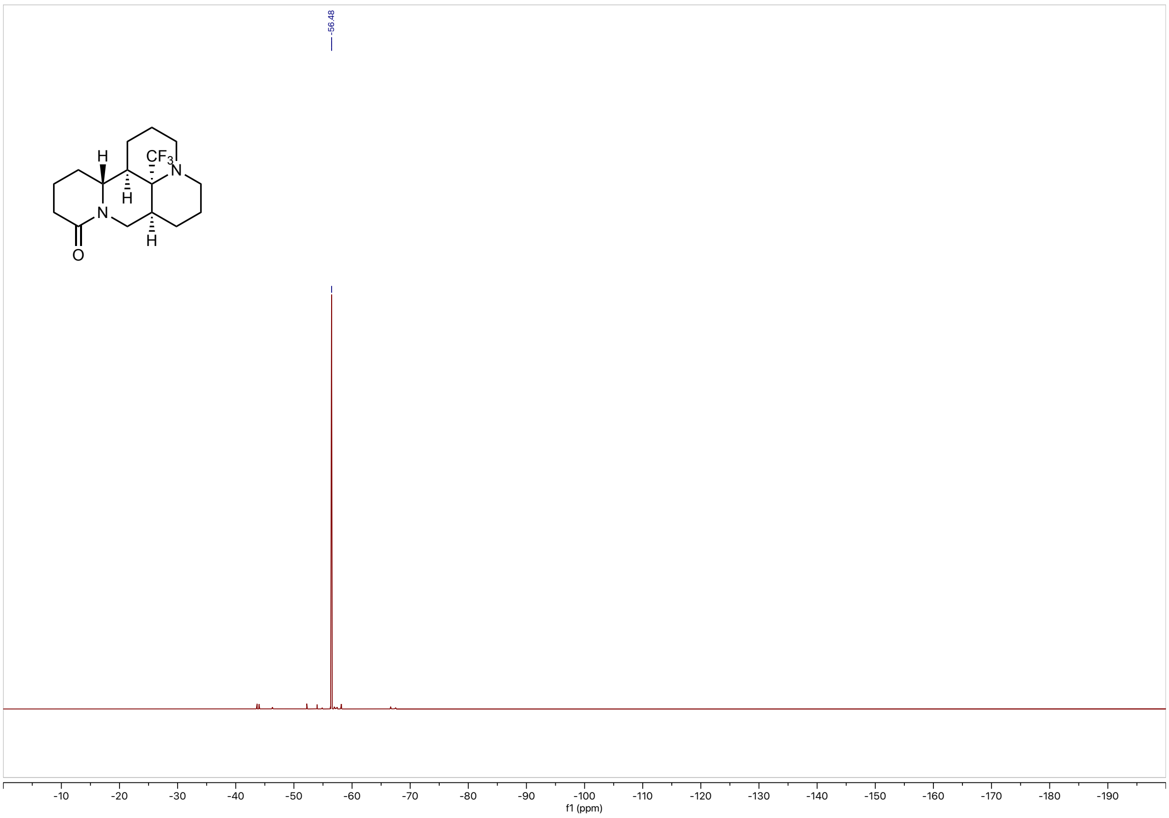

Heteronuclear ^{19}F-^{1}H NOSEY (500 MHz, $CDCl_3$) of (4[1]*S*,7a*S*,13a*R*,13b*R*)-4[1]-(trifluoro methyl)dodecahydro-1*H*,5*H*,10*H*-dipyrido[2,1-f:3',2',1'-ij][1,6]naphthyridin-10-one (**20B**)

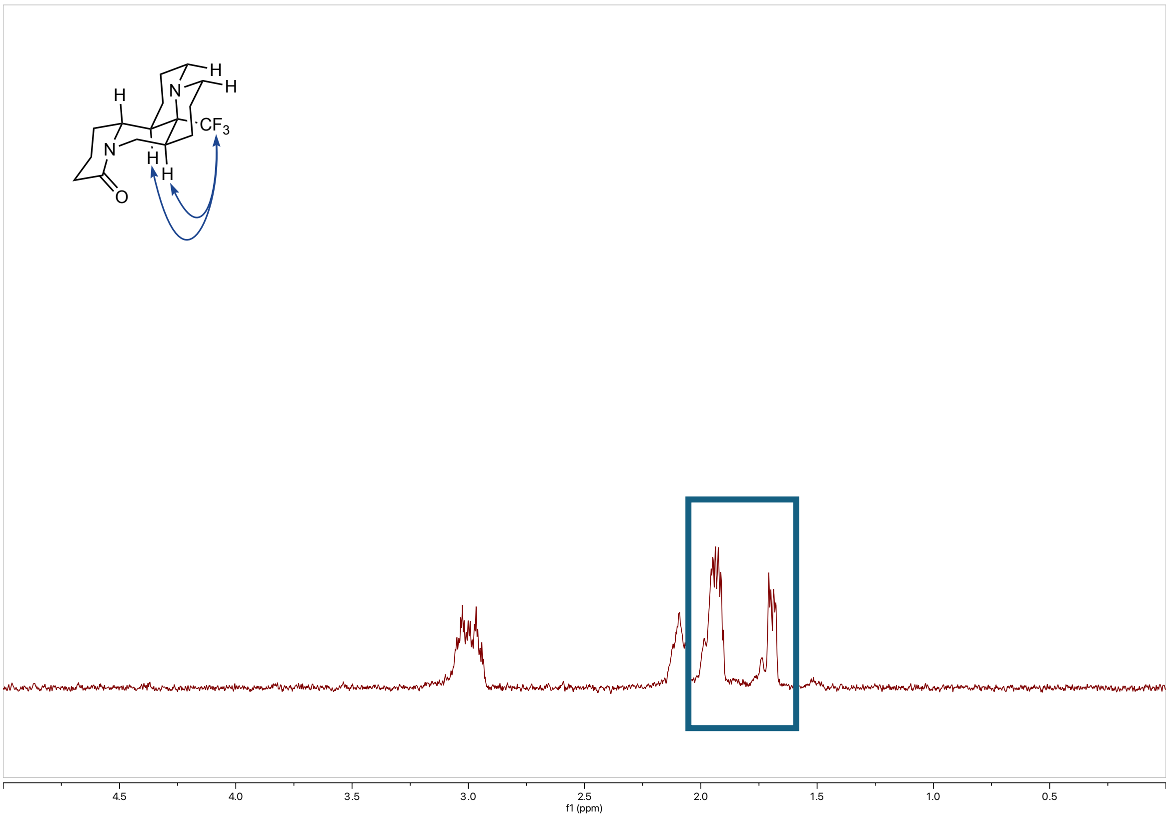

^{1}H NMR (700 MHz, $CDCl_3$) of 2-(2-(trifluoromethyl)piperidin-1-yl)ethyl 3-methyl-4-oxo-2-phenyl-4*H*-chromene-8-carboxylate (**21**):

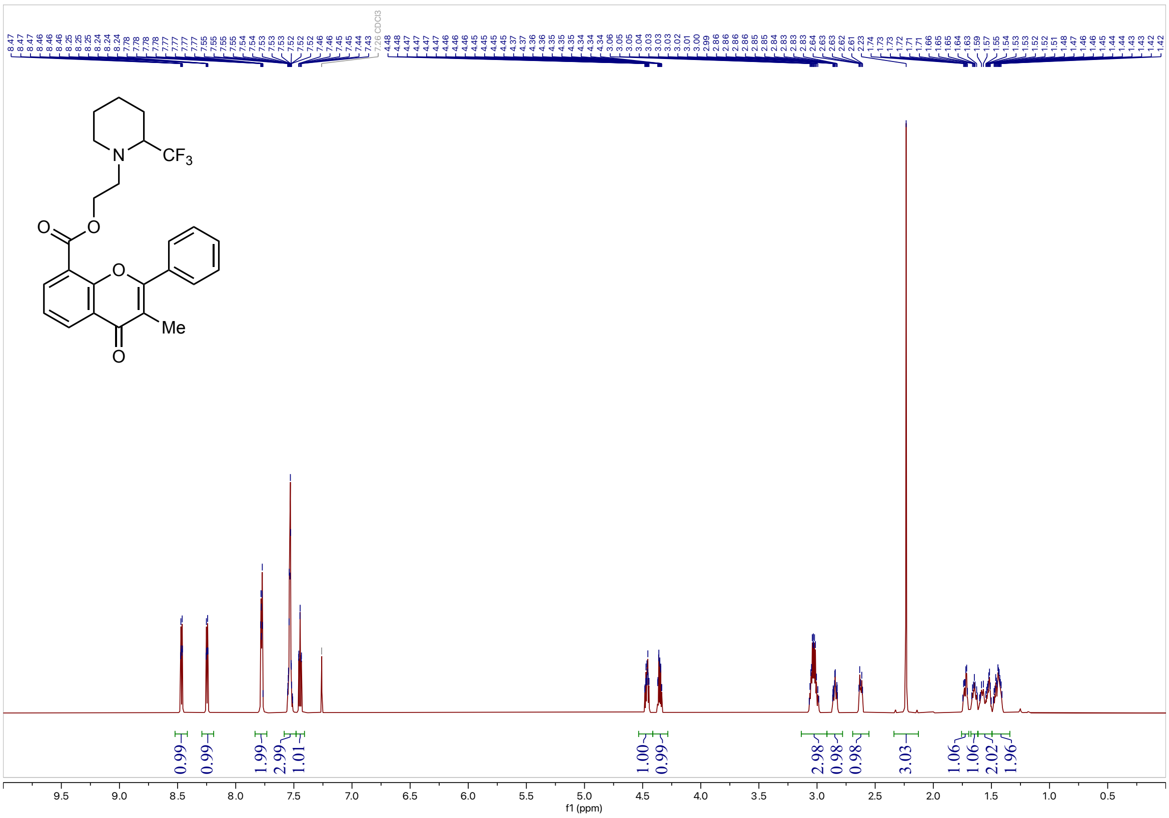

^{13}C{H} NMR (126 MHz, $CDCl_3$) of 2-(2-(trifluoromethyl)piperidin-1-yl)ethyl 3-methyl-4-oxo-2-phenyl-4*H*-chromene-8-carboxylate (**21**):

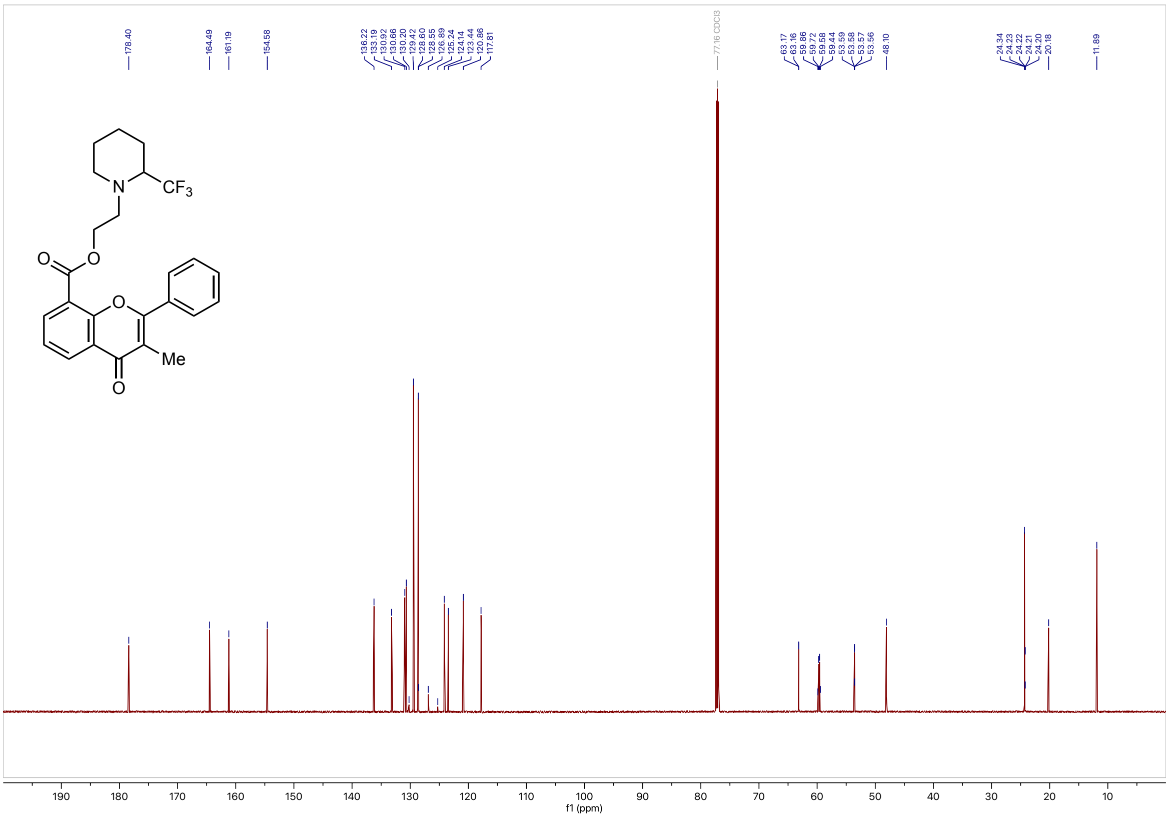

$^{19}F\{H\}$ NMR (471 MHz, $CDCl_3$) of 2-(2-(trifluoromethyl)piperidin-1-yl)ethyl 3-methyl-4-oxo-2-phenyl-4*H*-chromene-8-carboxylate (**21**):

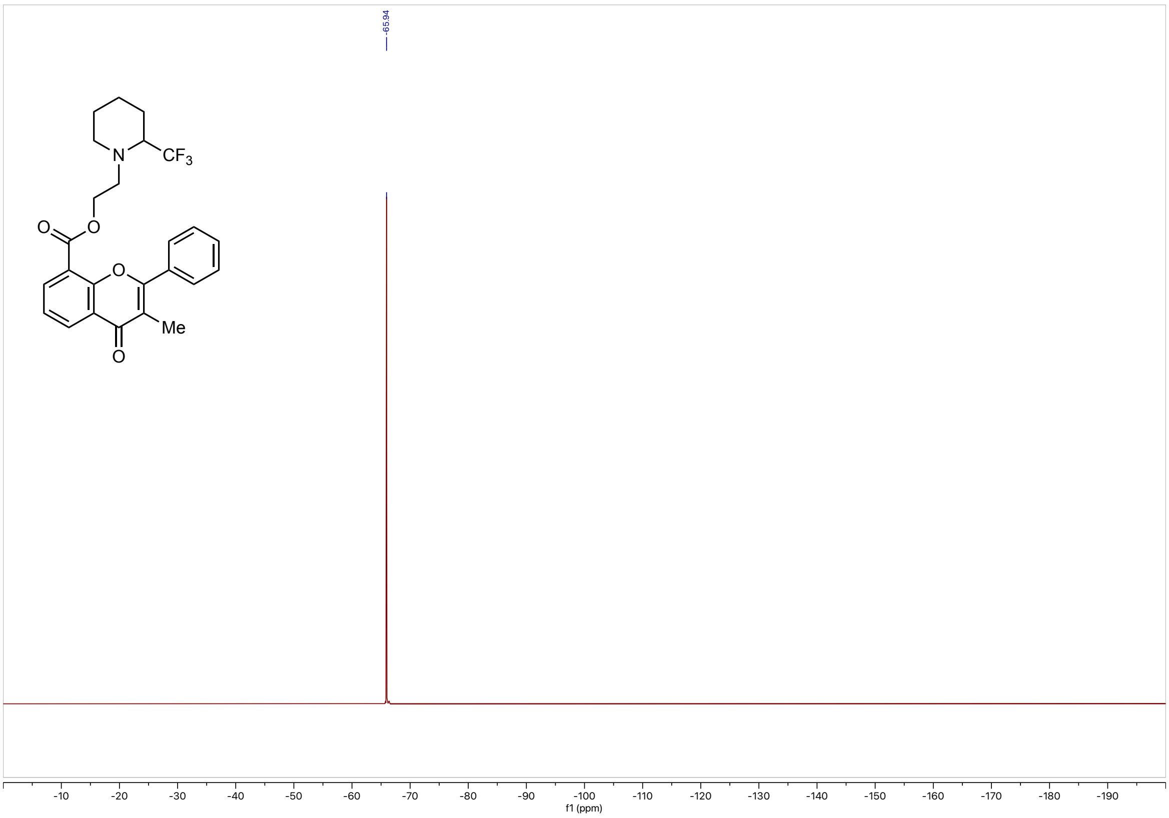

^{1}H NMR (500 MHz, $CDCl_3$) of (*1S,2R,3R,4aS,13bS,14aS*)-2,11-dimethoxy-1-(methoxycarbonyl)-13b-(trifluoromethyl)-3-((3,4,5-trimethoxybenzoyl)oxy)-2,3,4,4a,5,6,7,8,13,13b,14,14a-dodecahydro-1*H*-indolo[2',3':3,4]pyrido[1,2-b]isoquinolin-6-ium trifluoroacetate (**22**):

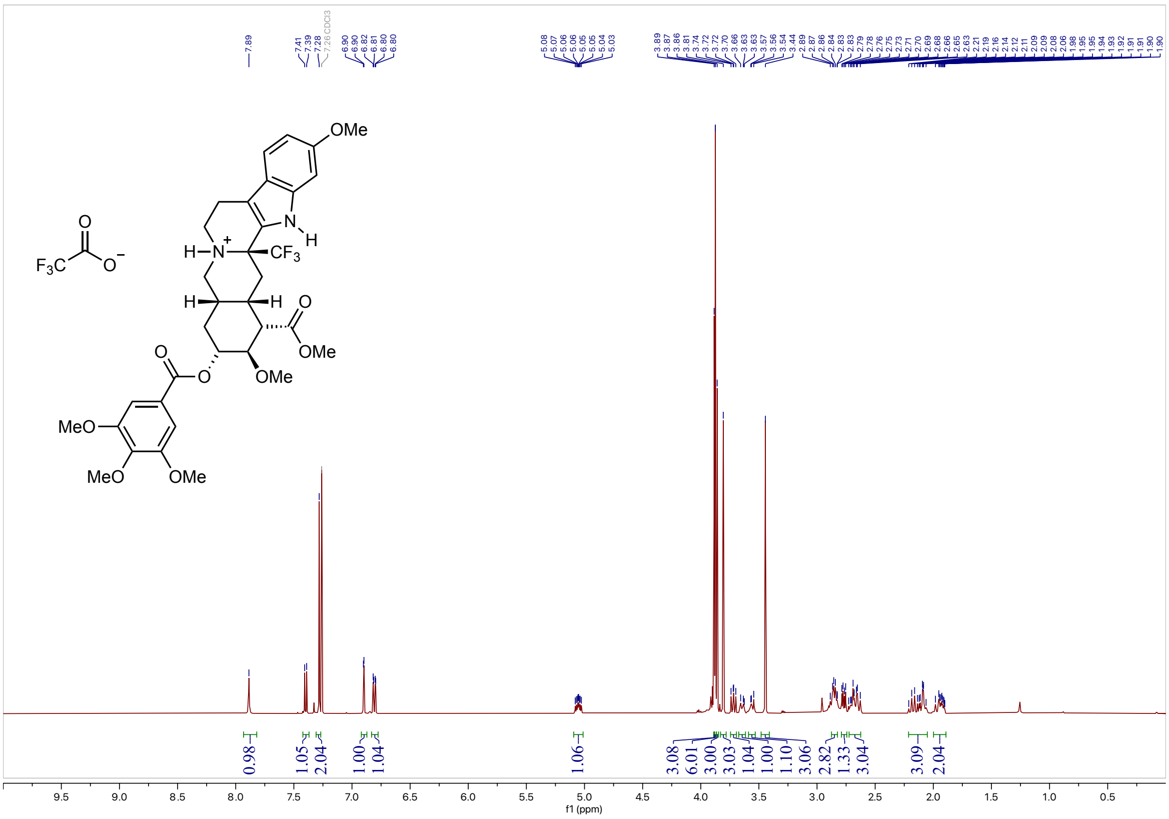

$^{13}C\{H\}$ NMR (126 MHz, $CDCl_3$) of (*1S,2R,3R,4aS,13bS,14aS*)-2,11-dimethoxy-1-(methoxycarbonyl)-13b-(trifluoromethyl)-3-((3,4,5-trimethoxybenzoyl)oxy)-2,3,4,4a,5,6,7,8,13,13b,14,14a-dodecahydro-1*H*-indolo[2',3':3,4]pyrido[1,2-b]isoquinolin-6-ium trifluoroacetate (**22**):

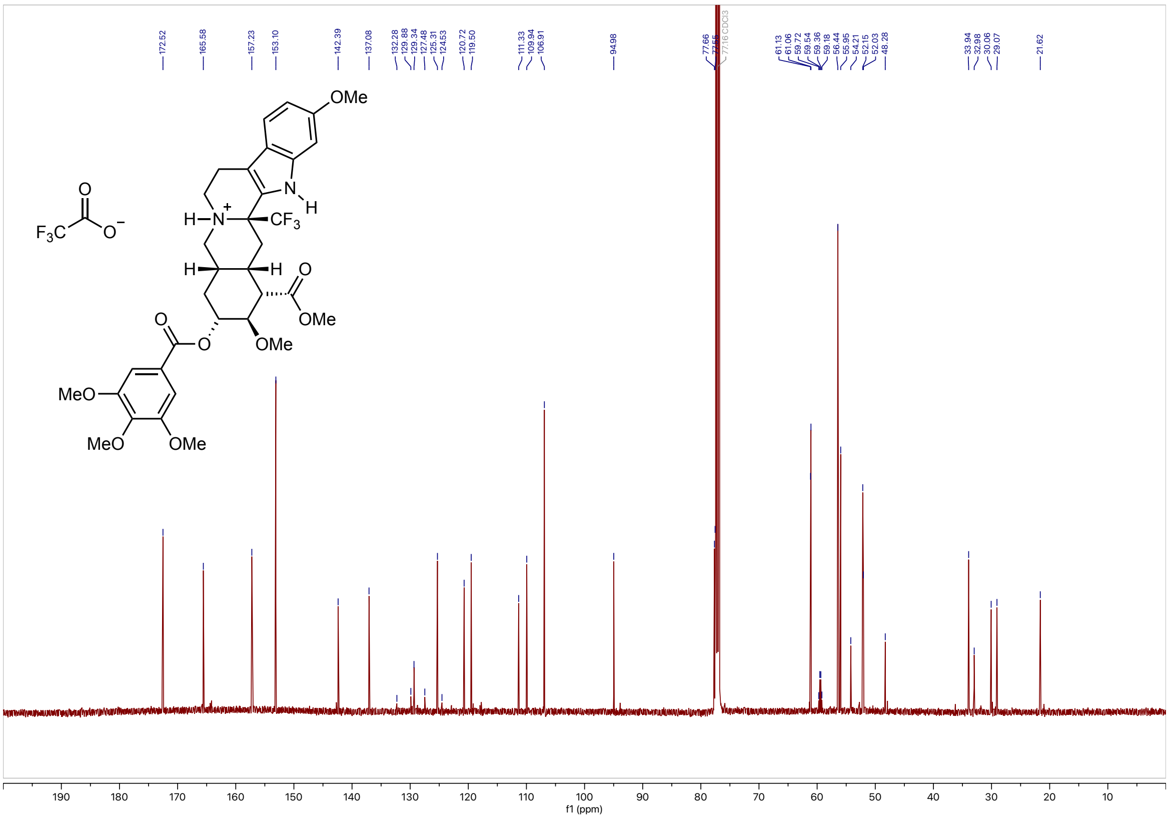

$^{19}F\{H\}$ NMR (471 MHz, $CDCl_3$) of (*1S,2R,3R,4aS,13bS,14aS*)-2,11-dimethoxy-1-(methoxycarbonyl)-13b-(trifluoromethyl)-3-((3, 4,5-trimethoxybenzoyl)oxy)-2,3,4,4a,5,6,7,8,13,13b,14,14a-dodecahydro-1*H*-indolo[2', 3':3,4]pyrido[1,2-b]isoquinolin-6-ium trifluoroacetate (**22**):

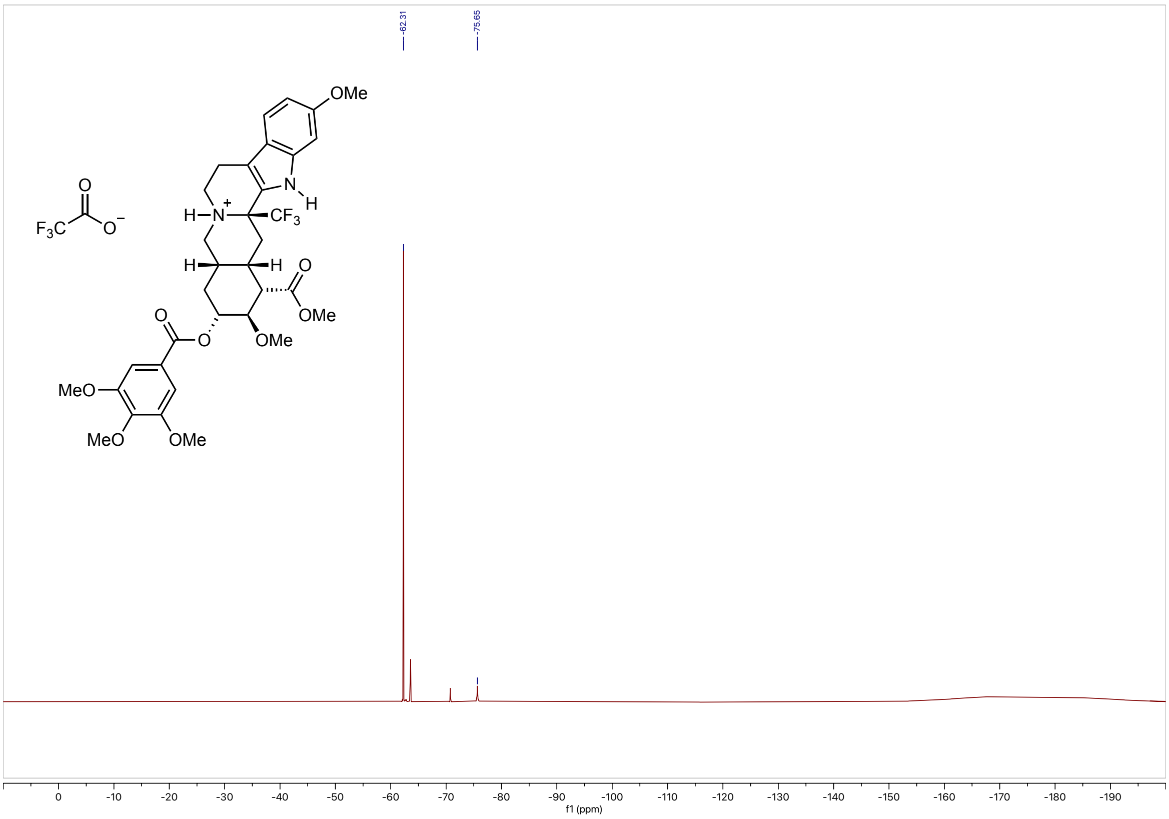

Heteronuclear ^{19}F–^{1}H NOSEY (500 MHz, $CDCl_3$) of (*1S,2R,3R,4aS,13bS,14aS*)-2,11-dimethoxy-1-(methoxycarbonyl)-13b-(trifluoromethyl)- 3-((3,4,5-trimethoxybenzoyl) oxy)-2,3,4,4a,5,6,7,8,13,13b,14,14a-dodecahydro-1*H*- indolo[2', 3':3,4]pyrido[1,2-b]isoquinolin-6-ium trifluoroacetate (**22**):

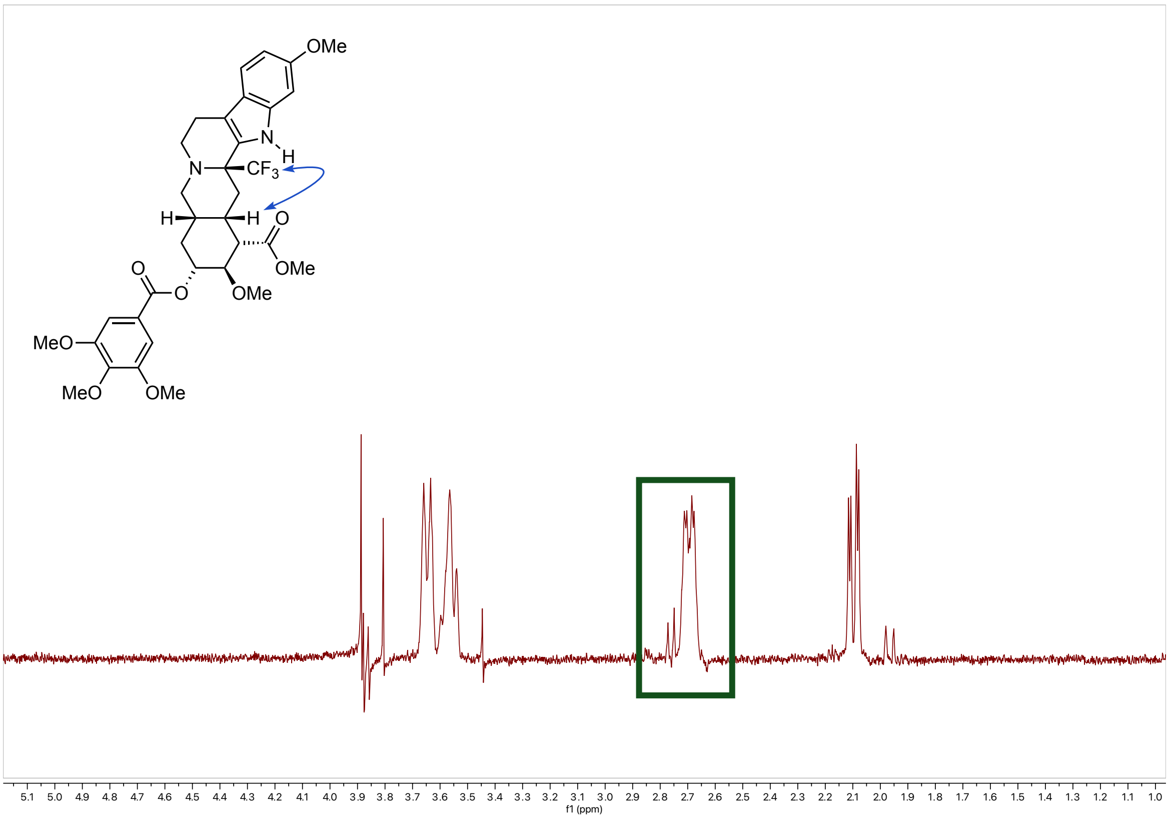

HSQC (500 MHz, $CDCl_3$) of (*1S,2R,3R,4aS,13bS,14aS*)-2,11-dimethoxy-1-(methoxycarbonyl)-13b-(trifluoromethyl)- 3-((3,4,5-trimethoxybenzoyl)oxy)-2,3,4,4a,5,6,7,8,13,13b,14,14a-dodecahydro-1*H*- indolo[2', 3':3,4]pyrido[1,2-b]isoquinolin-6-ium trifluoroacetate (**22**):

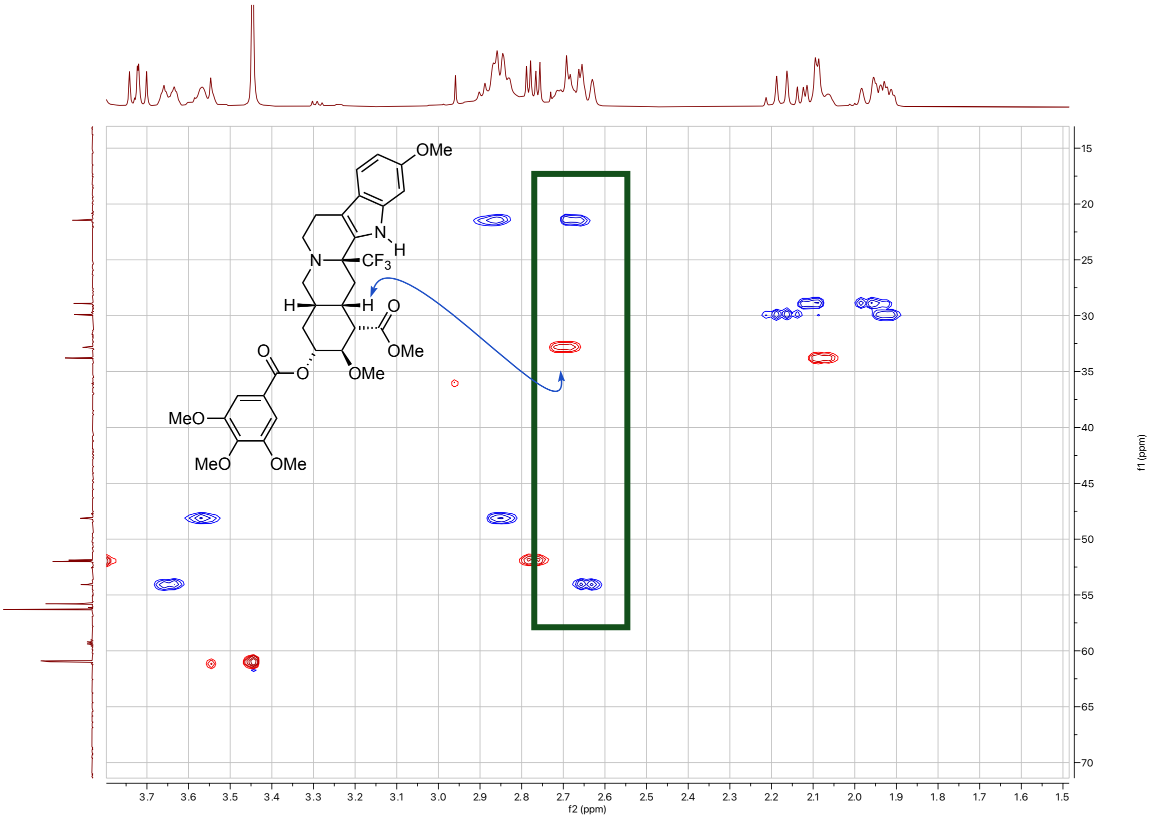

^{1}H NMR (700 MHz, $CDCl_3$) of 4-(6-fluorobenzo[d]isoxazol-3-yl)-1-(2-(2-methyl-4-oxo-6,7,8,9-tetrahydro-4*H*-pyrido[1,2-a]pyrimidin-3-yl)ethyl)-2-(trifluoromethyl)piperidin-1-ium trifluoroacetate (**23**):

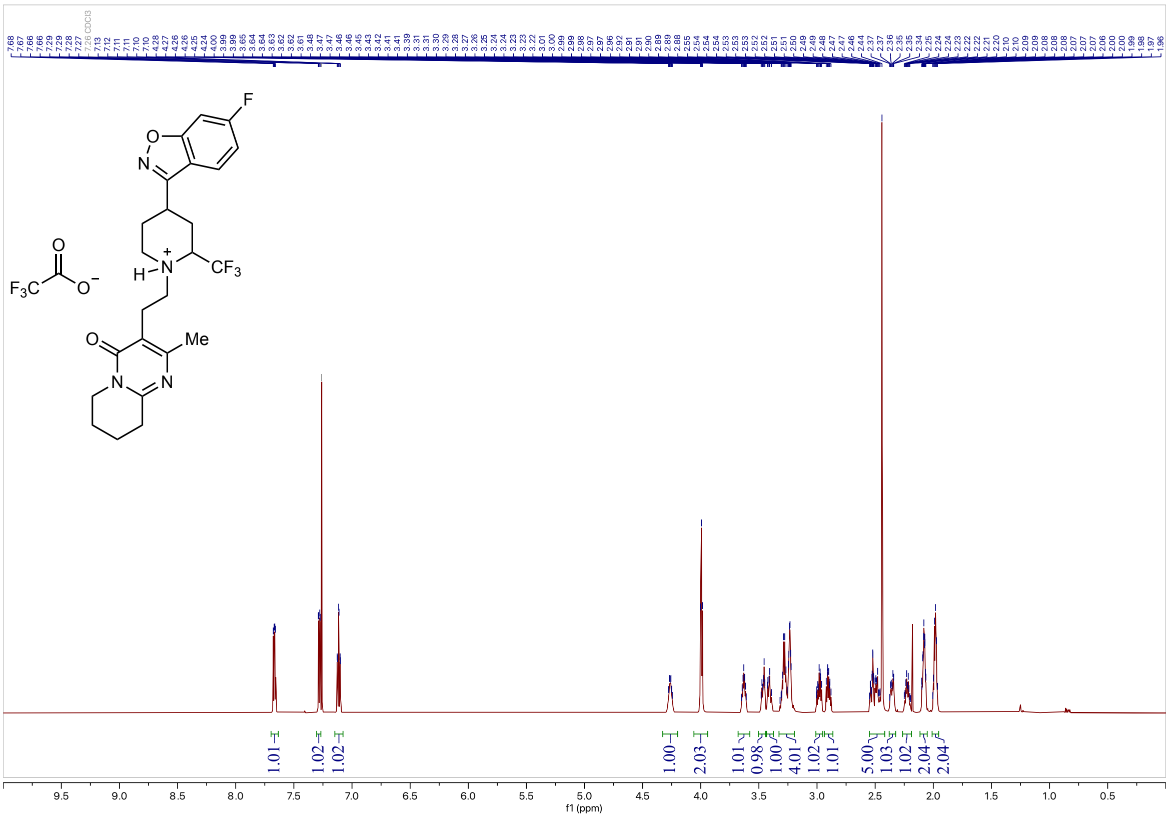

$^{13}C\{H\}$ NMR (176 MHz, $CDCl_3$) of 4-(6-fluorobenzo[d]isoxazol-3-yl)-1-(2-(2-methyl-4-oxo-6,7,8,9-tetrahydro-4*H*-pyrido[1,2-a]pyrimidin-3-yl)ethyl)-2-(trifluoromethyl)piperidin-1-ium trifluoroacetate (**23**):

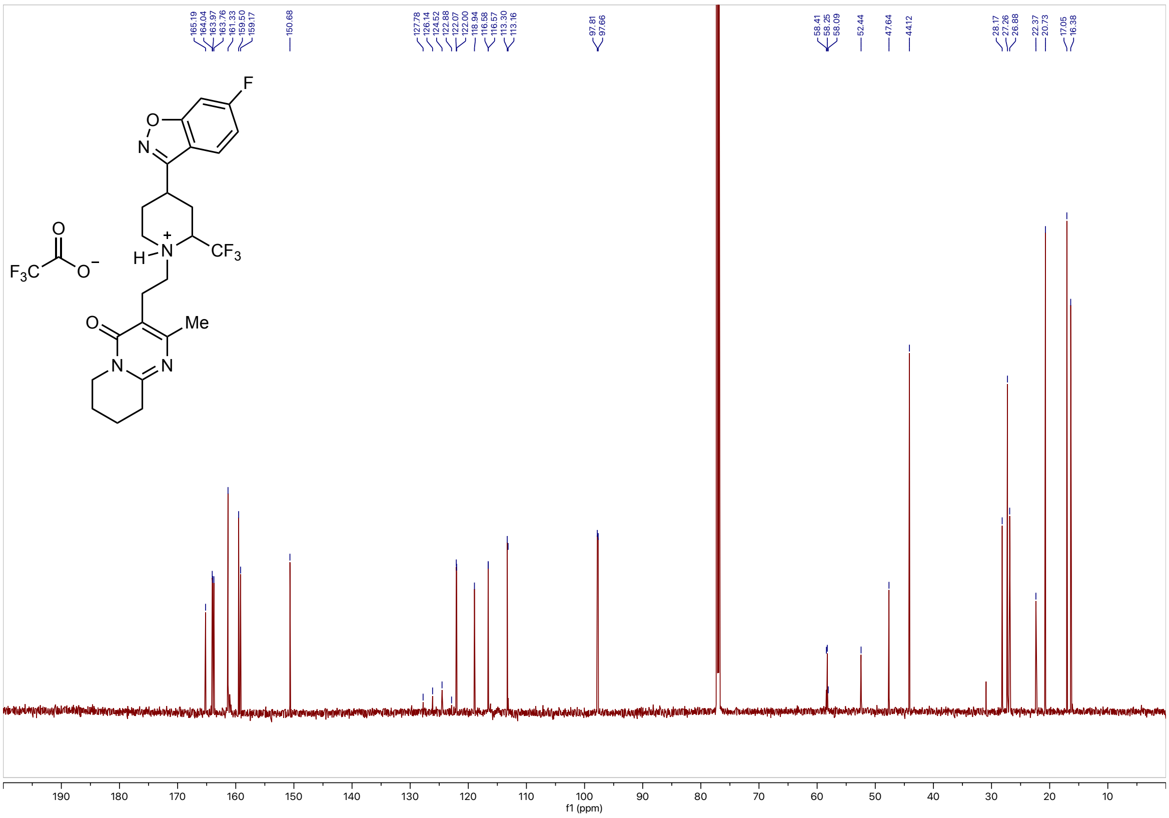

$^{19}F\{H\}$ NMR (471 MHz, $CDCl_3$) of 4-(6-fluorobenzo[d]isoxazol-3-yl)-1-(2-(2-methyl-4-oxo-6,7,8,9-tetrahydro-4*H*-pyrido[1,2-a]pyrimidin-3-yl)ethyl)-2-(trifluoromethyl)piperidin-1-ium trifluoroacetate (**23**):

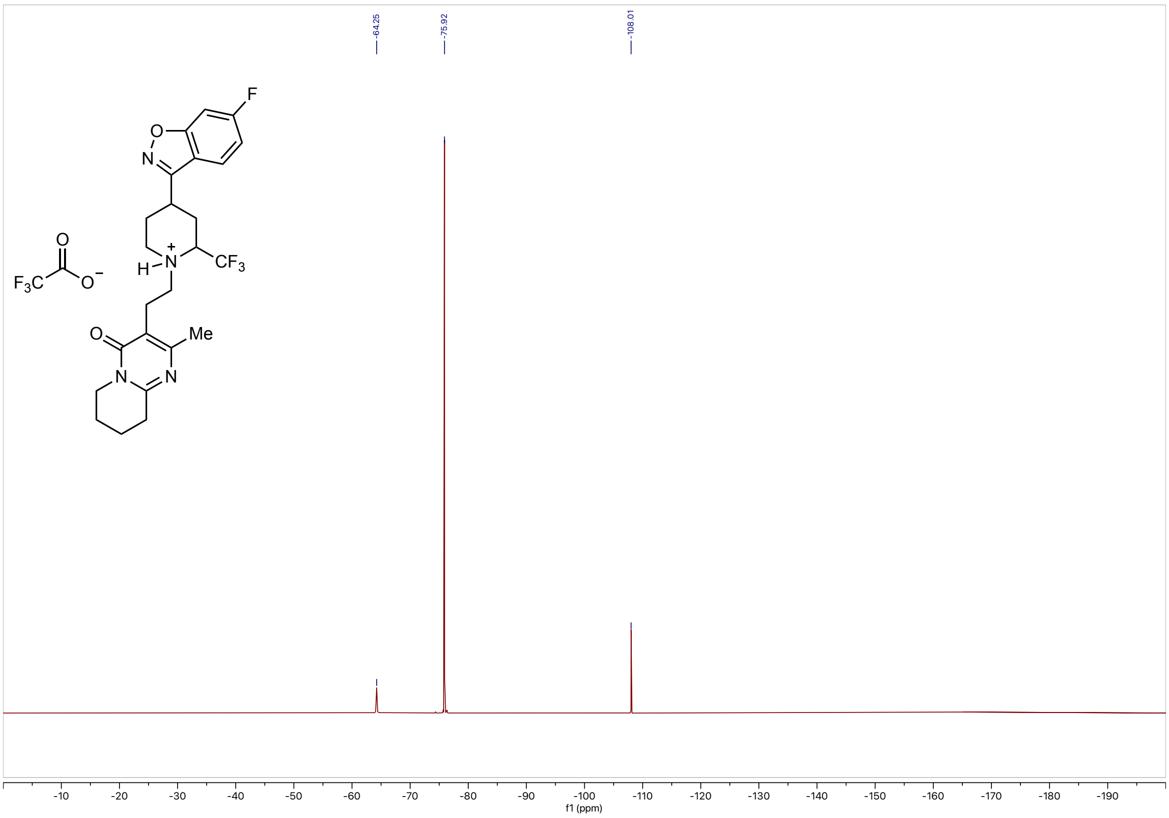

^{1}H NMR (500 MHz, $CDCl_3$) of 1-benzyl-1-hydroxypiperidin-1-ium chloride (**5**):

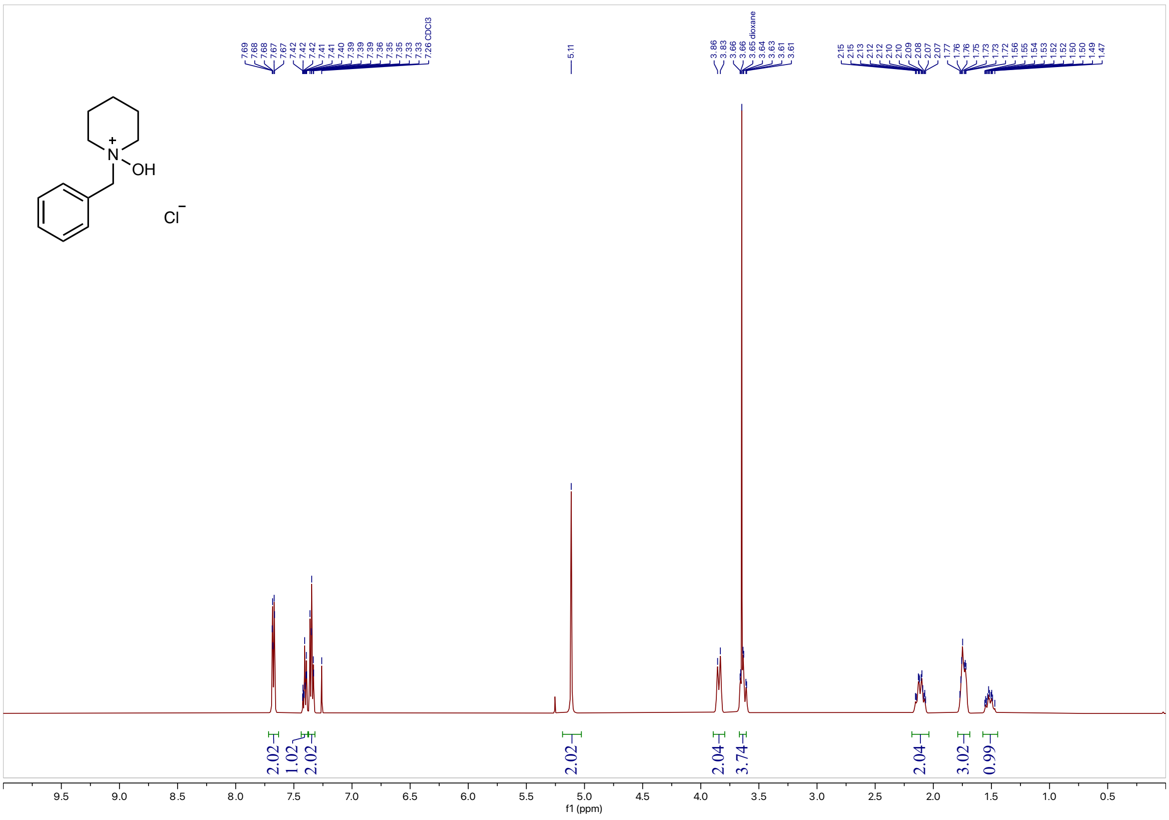

^{13}C{H} NMR (126 MHz, $CDCl_3$) of 1-benzyl-1-hydroxypiperidin-1-ium chloride (**5**):

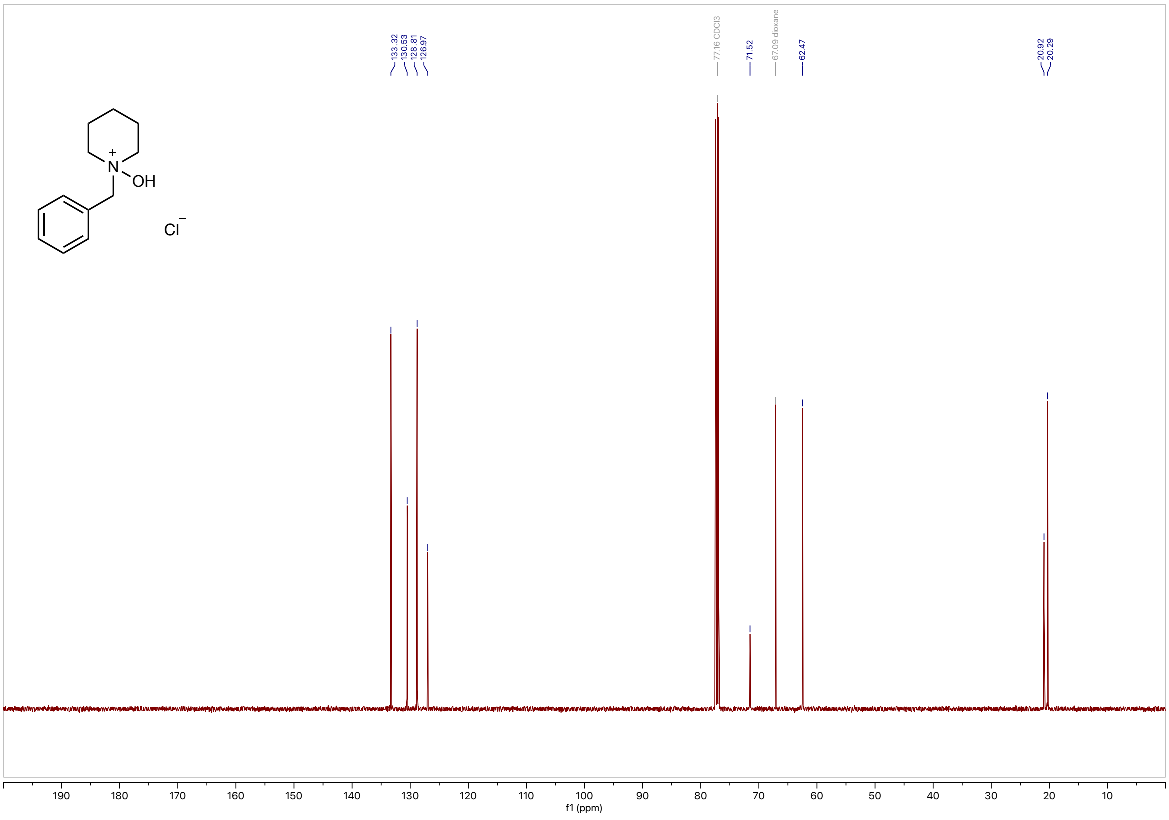

^{1}H NMR (500 MHz, $CDCl_3$) of *1-benzyl-2-(fluoromethyl)piperidine* (**6y**):

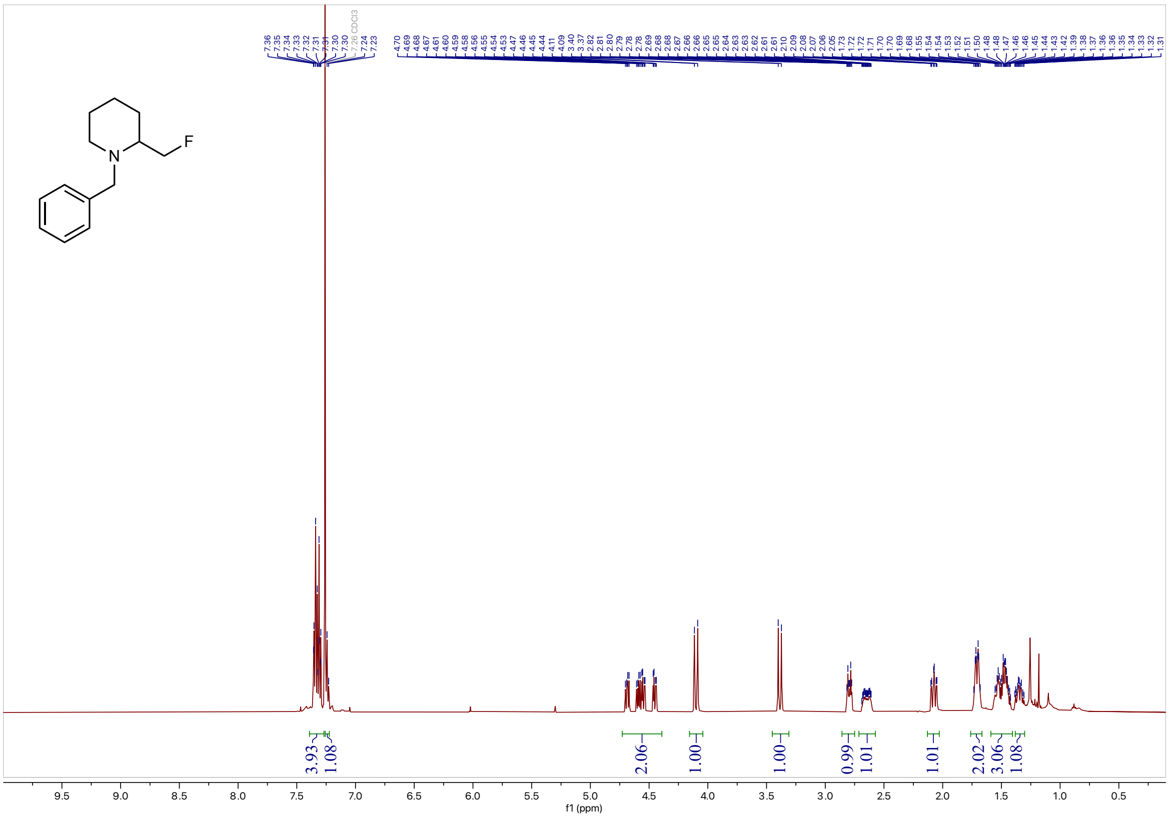

^{13}C{H} NMR (101 MHz, CD_2Cl_2) of *1-benzyl-2-(fluoromethyl)piperidine* (**6y**):

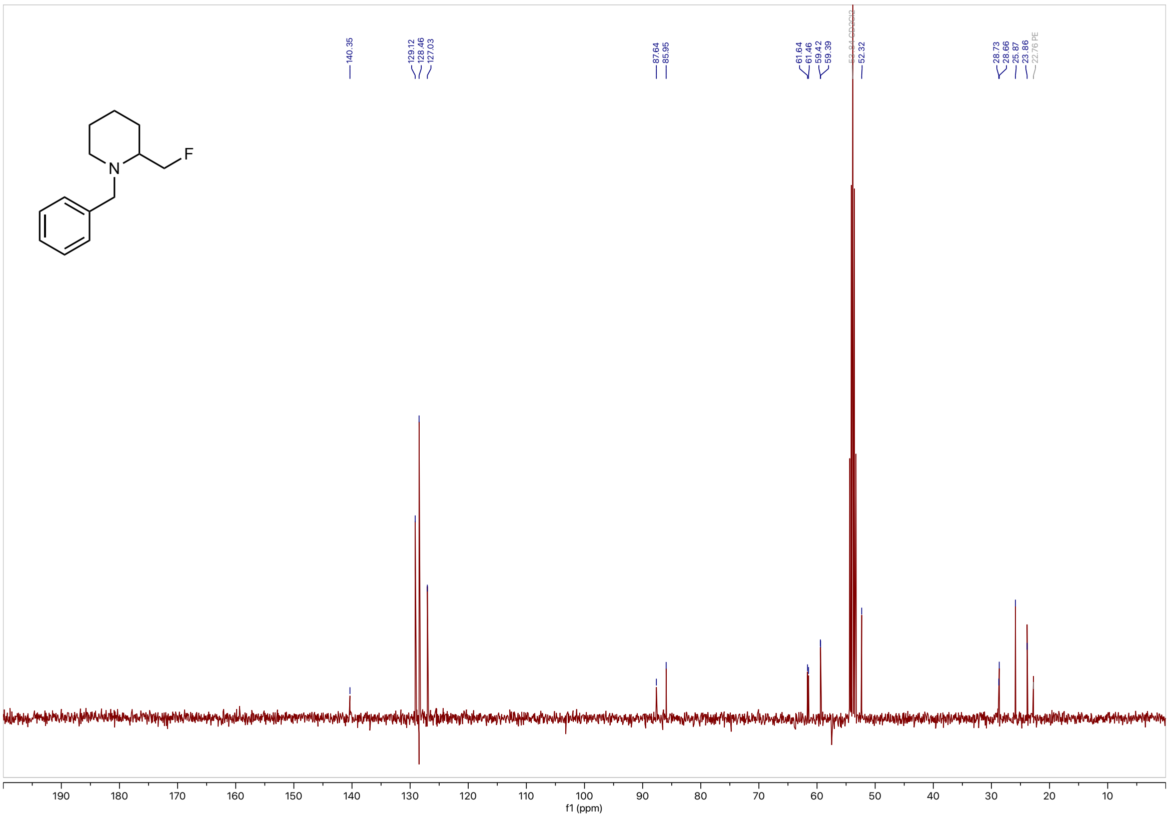

$^{19}F\{H\}$ NMR (376 MHz, CD_2Cl_2) of *1-benzyl-2-(fluoromethyl)piperidine* (**6y**):

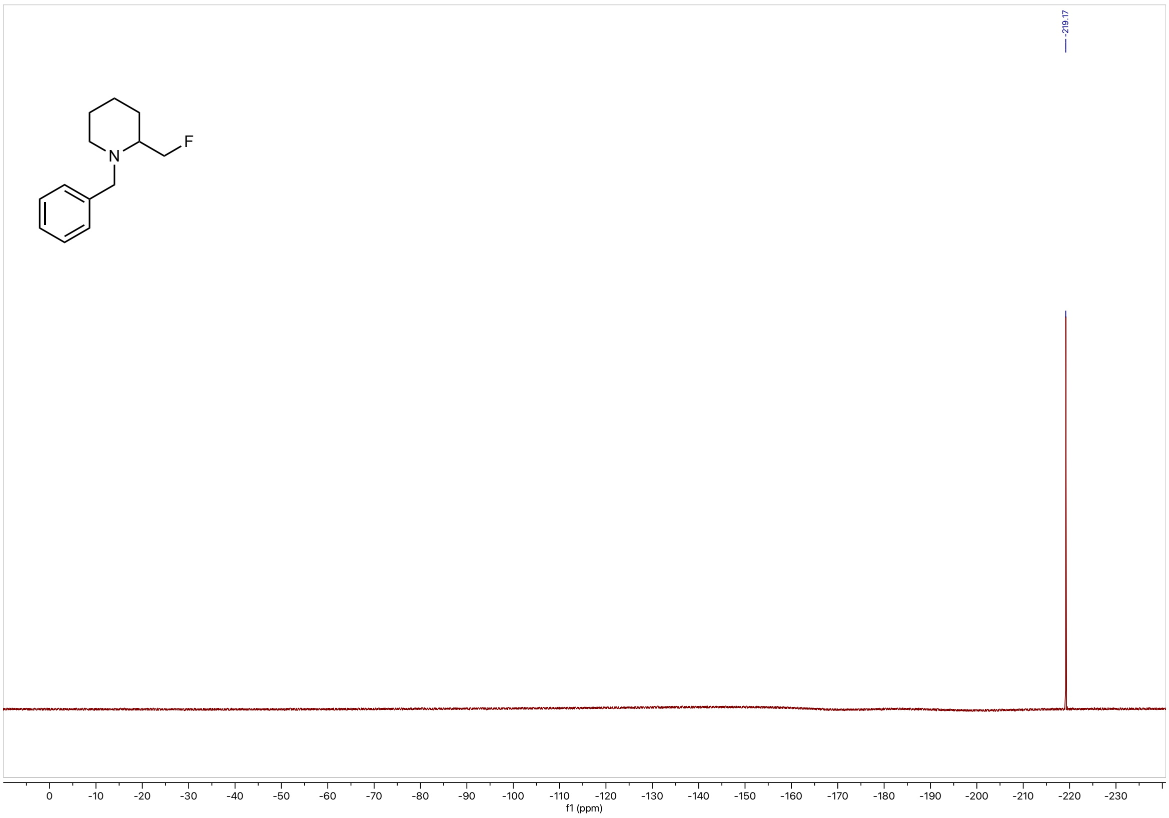

^{1}H NMR (700 MHz, $CDCl_3$) of 2-(adamantan-1-yl)-1-benzyl-5-methyl-piperidine (**6z**):

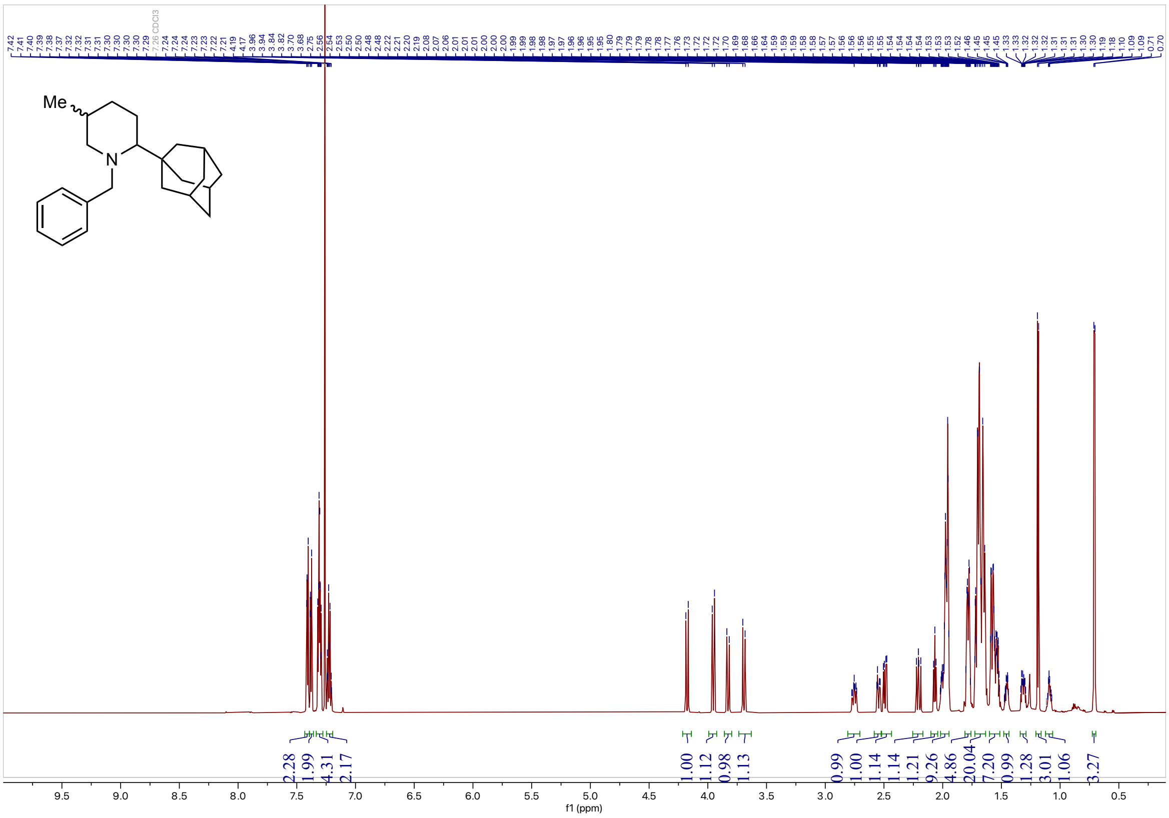

$^{13}C\{H\}$ NMR (176 MHz, $CDCl_3$) of 2-(adamantan-1-yl)-1-benzyl-5-methyl-piperidine (**6z**):

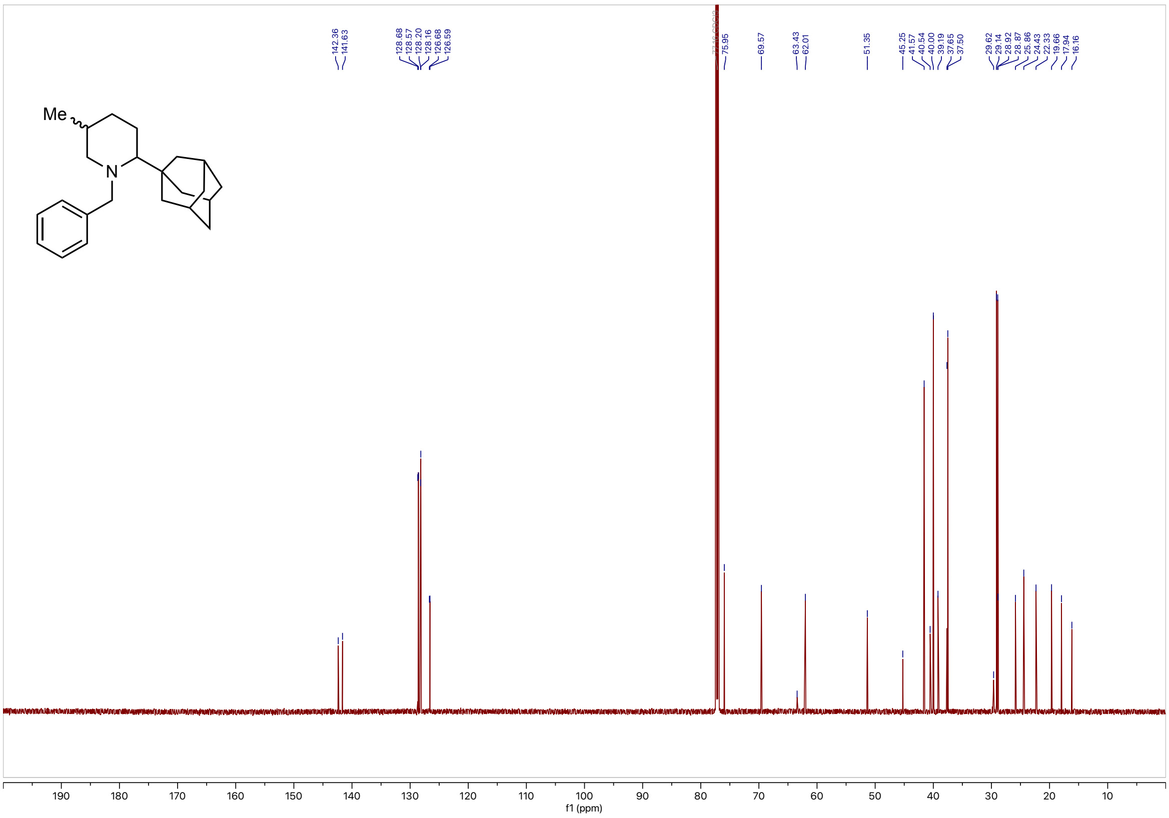

^{1}H NMR (700 MHz, $CDCl_3$) of (*2R,3R*)-2-((*1s,3S*)-adamantan-1-yl)-1-benzyl-3-methyl-piperidine (**6z’**):

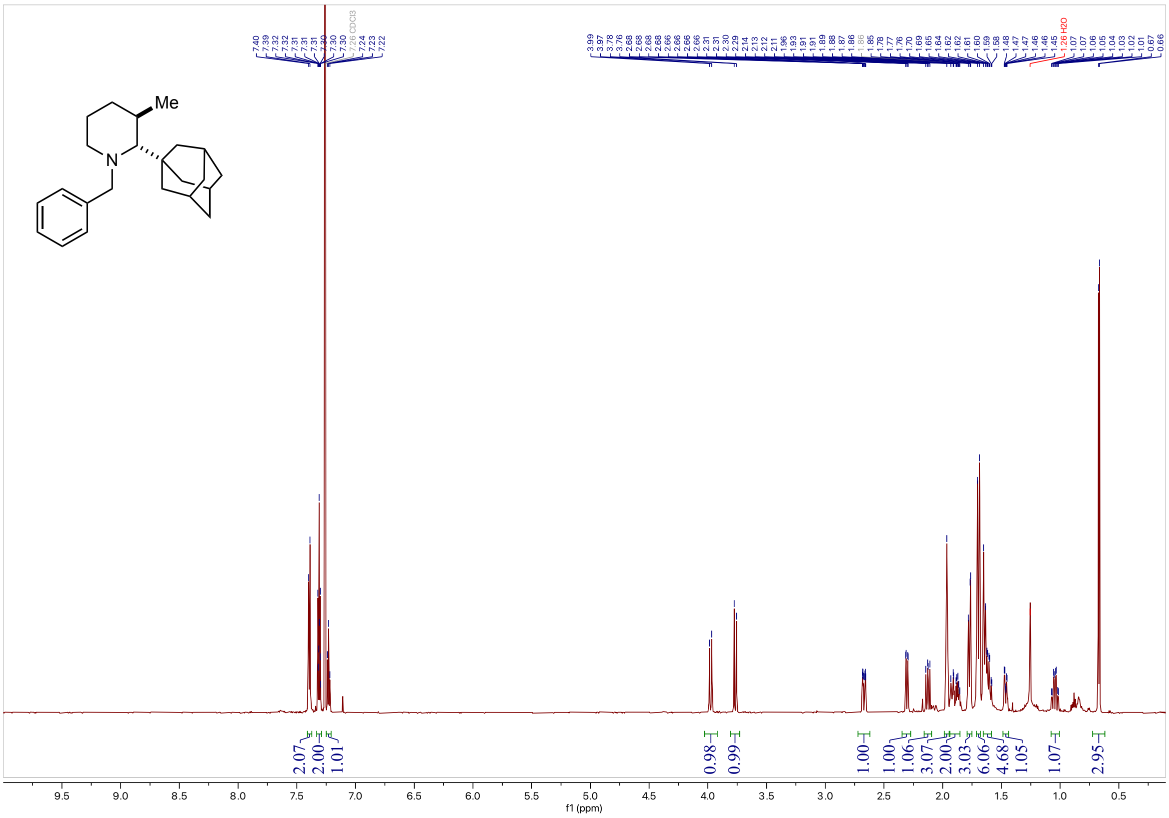

^{13}C{H} NMR (176 MHz, $CDCl_3$) of (*2R,3R*)-2-((*1s,3S*)-adamantan-1-yl)-1-benzyl-3-methyl-piperidine (**6z’**):

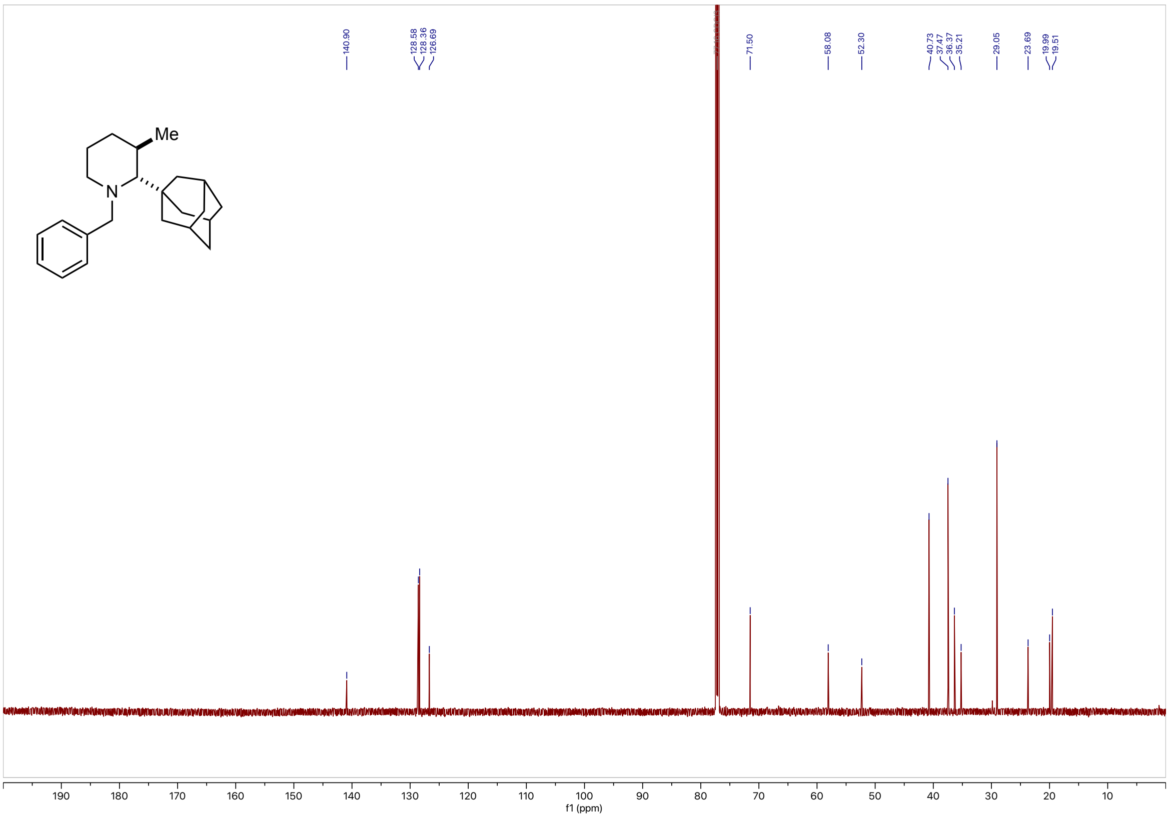

^{1}H NMR (700 MHz, $CDCl_3$) of 2-((1*r*,3*S*)-adamantan-1-yl)-1-benzyl-4-methylpiperidine (**6aa**):

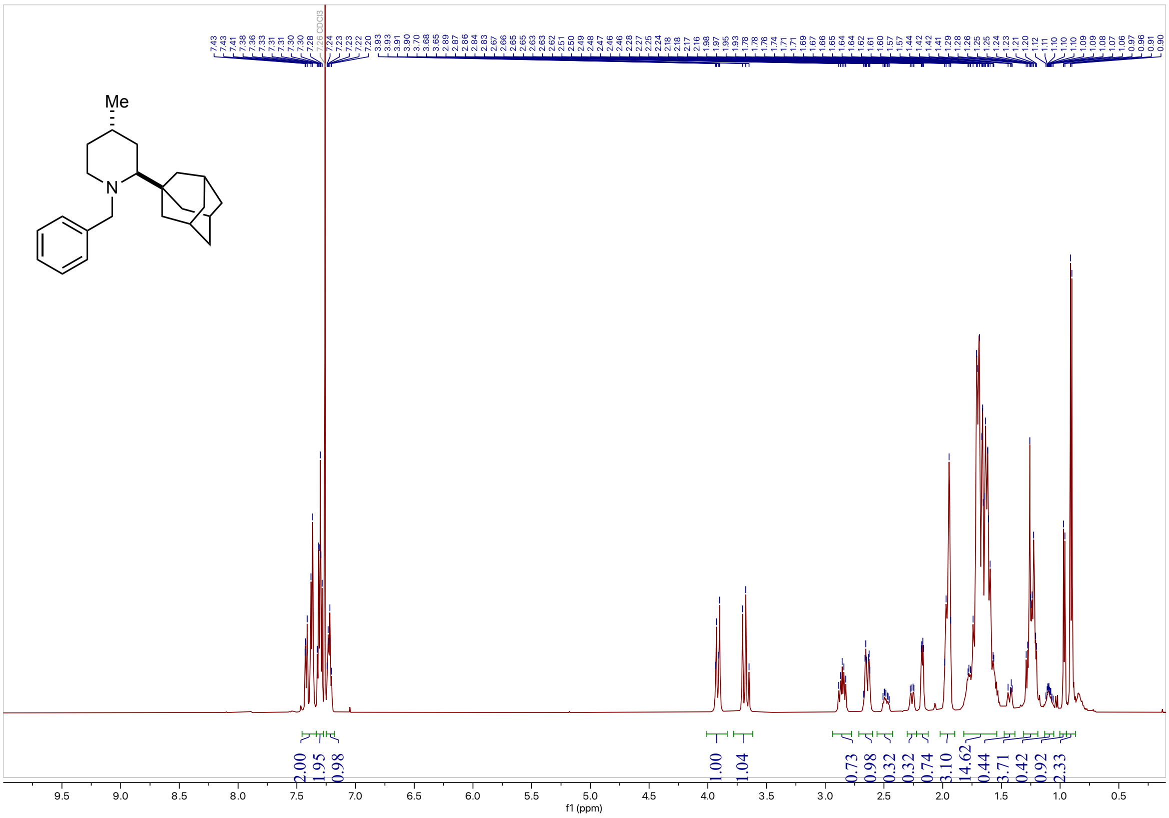

$^{13}C\{H\}$ NMR (176 MHz, $CDCl_3$) of 2-((1*r*,3*S*)-adamantan-1-yl)-1-benzyl-4-methyl piperidine (**6aa**):

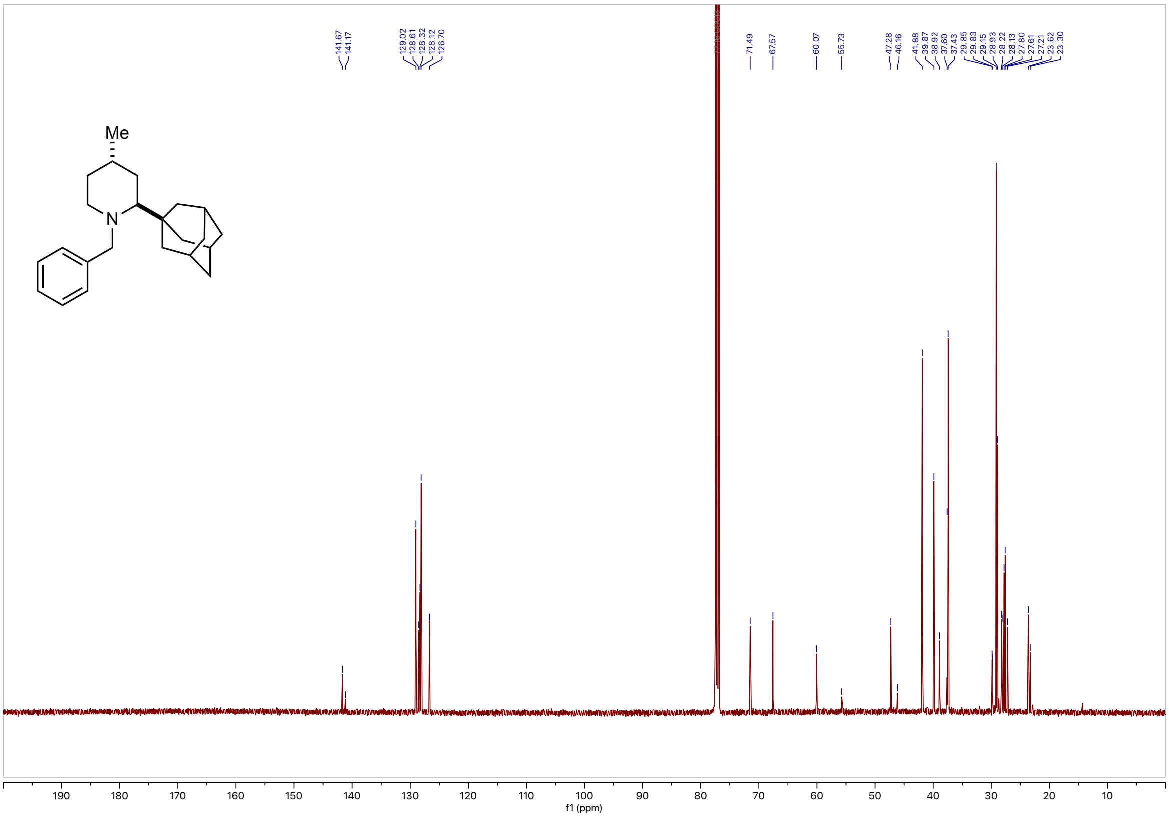

NOSEY (500 MHz, CDCl$_3$) of 2-((*1s,3s*)-adamantan-1-yl)-1-benzyl-4-methylpiperidine (**6aa**)*:*

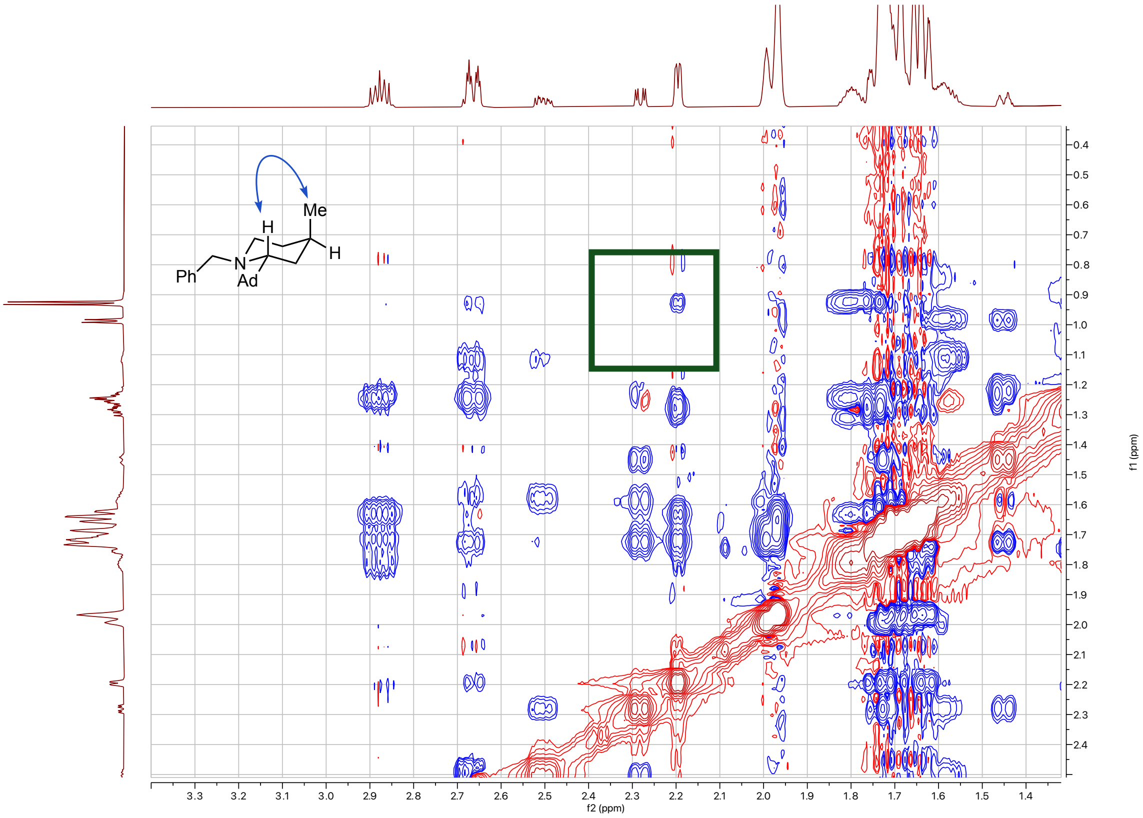

^{1}H NMR (700 MHz, $CDCl_3$) of 2-(adamantan-1-yl)-1-benzylazepane (**6ab**):

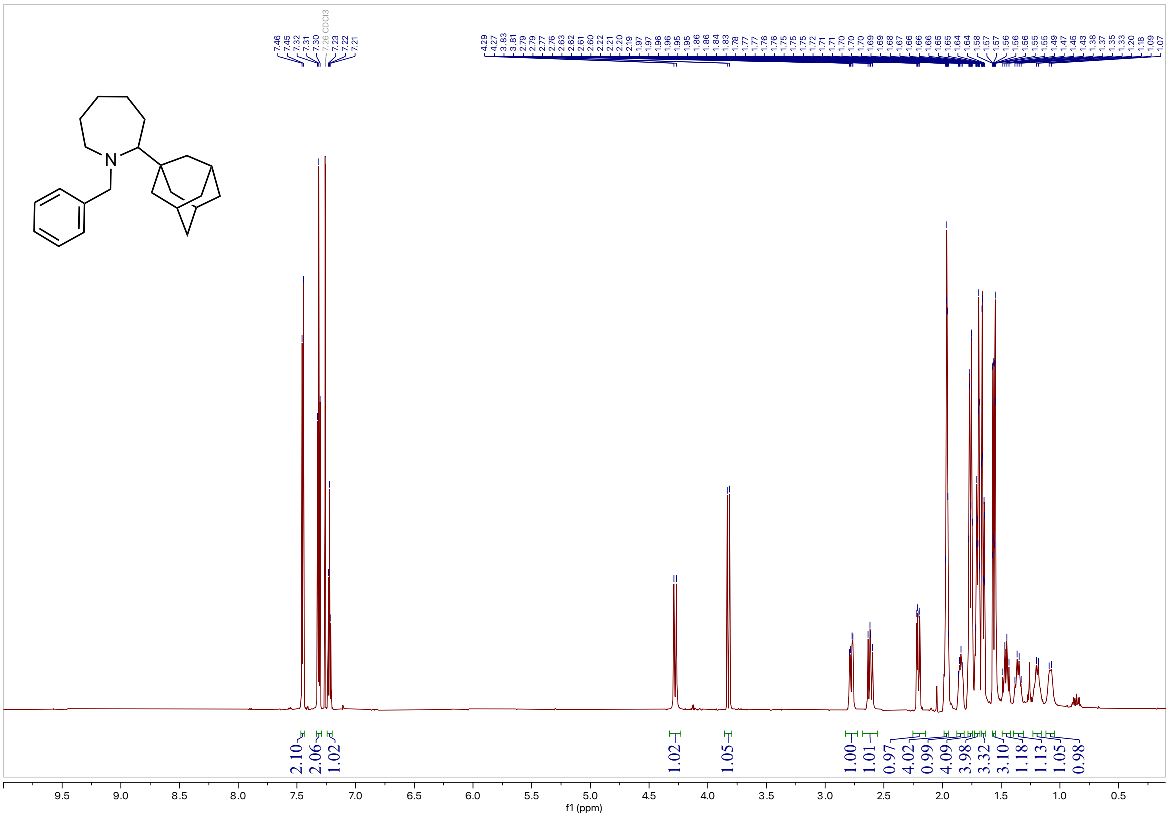

$^{13}C\{H\}$ NMR (176 MHz, $CDCl_3$) of 2-(adamantan-1-yl)-1-benzylazepane (**6ab**):

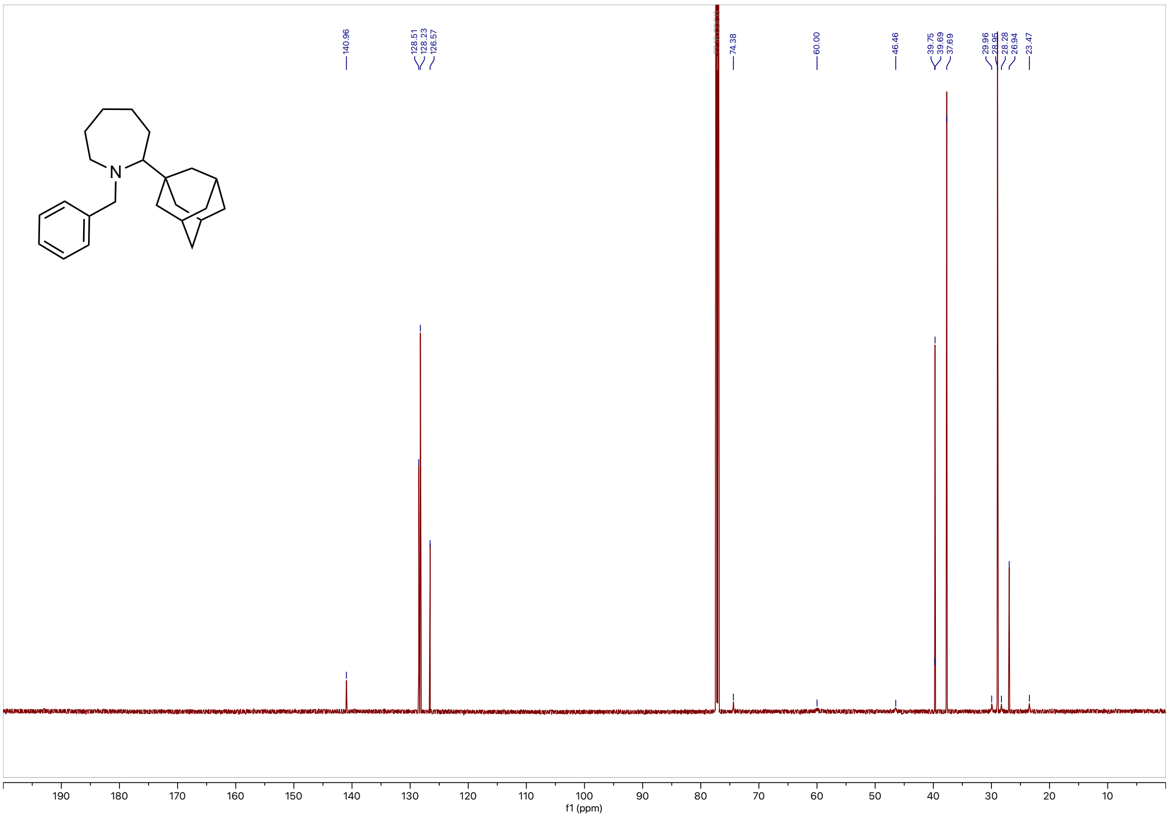

^{1}H NMR (700 MHz, $CDCl_3$) of 1-((adamantan-1-yl)(phenyl)methyl)azepane (**6ab’**):

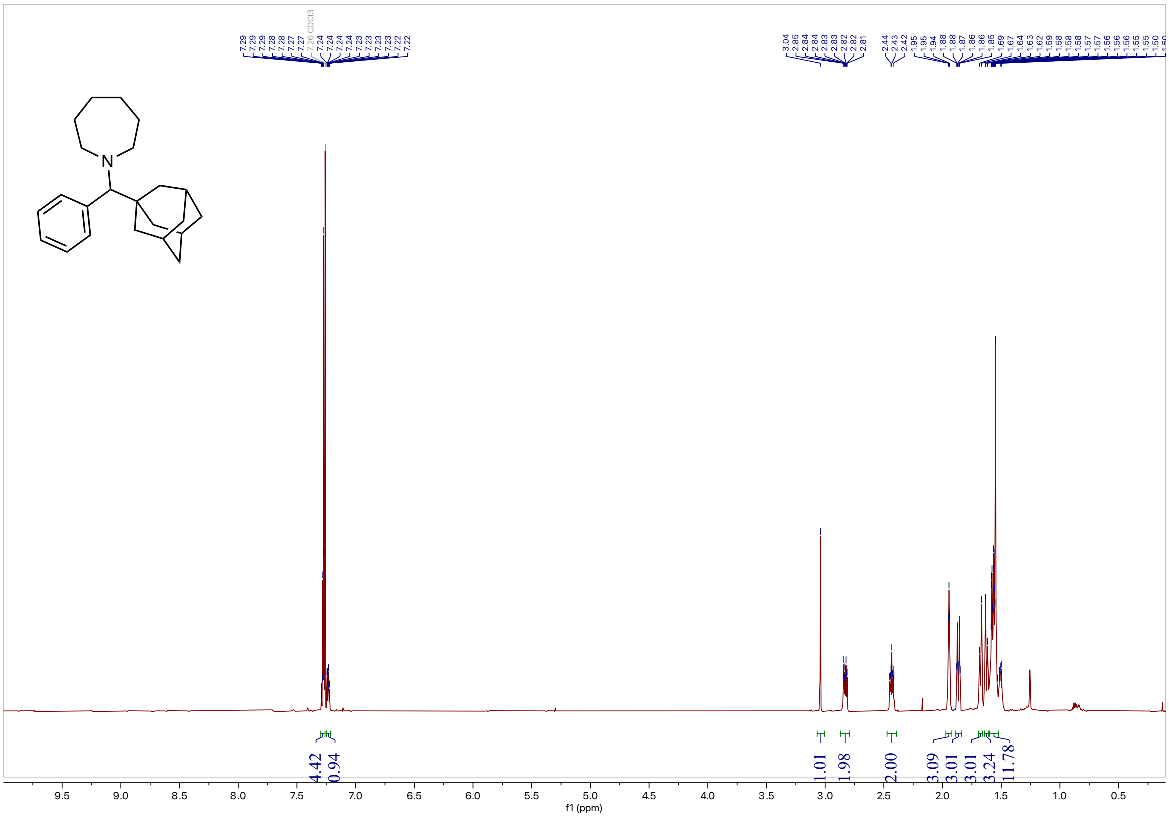

^{13}C{H} NMR (176 MHz, $CDCl_3$) of 1-((adamantan-1-yl)(phenyl)methyl)azepane (**6ab’**):

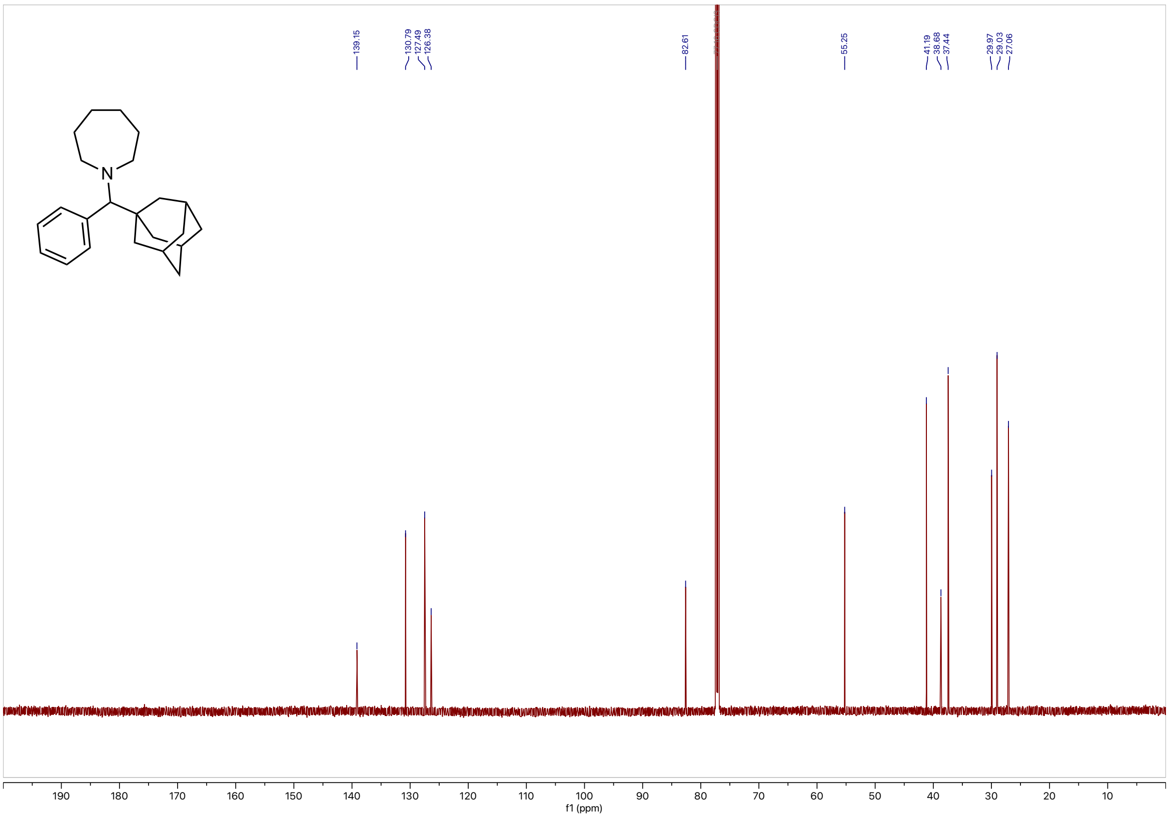

^{1}H NMR (700 MHz, $CDCl_3$) of (2*R*,4*S*)-1-benzyl-2,4-dimethylpiperidine (**8b**):

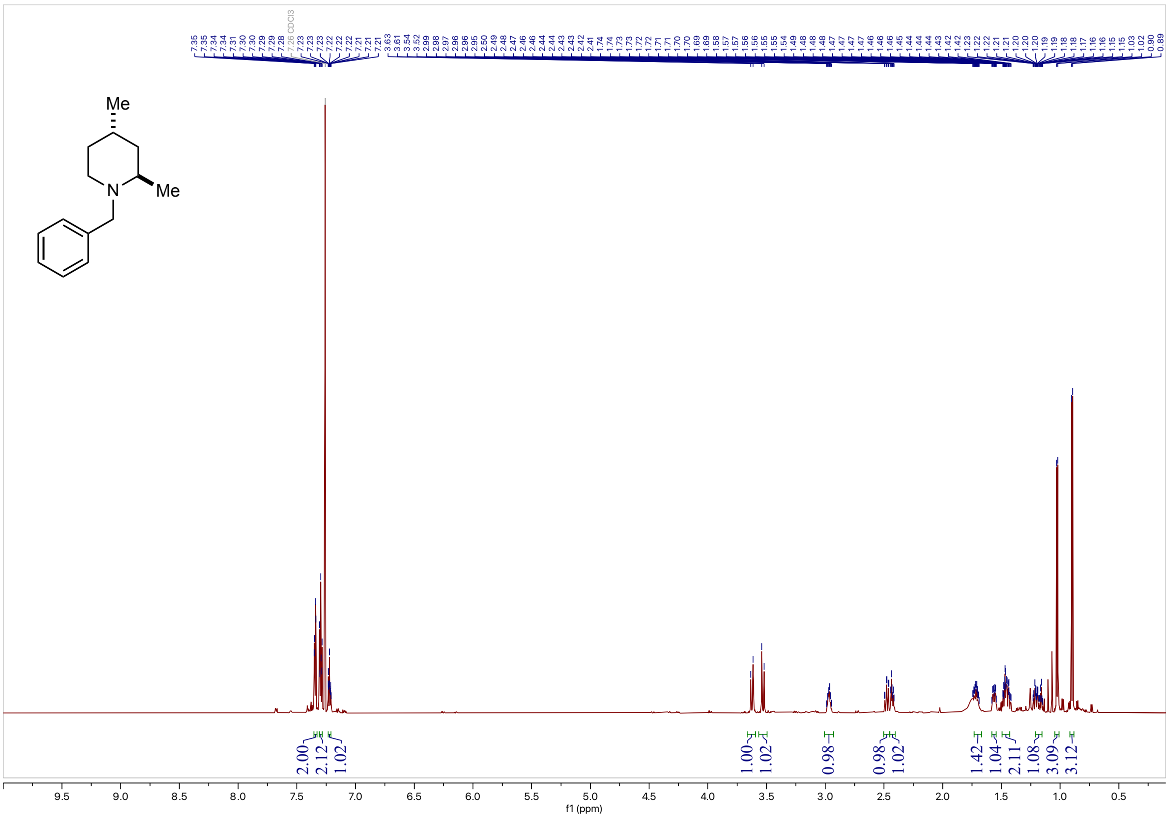

^{13}C{H} NMR (176 MHz, $CDCl_3$) of (2*R*,4*S*)-1-benzyl-2,4-dimethylpiperidine (**8b**):

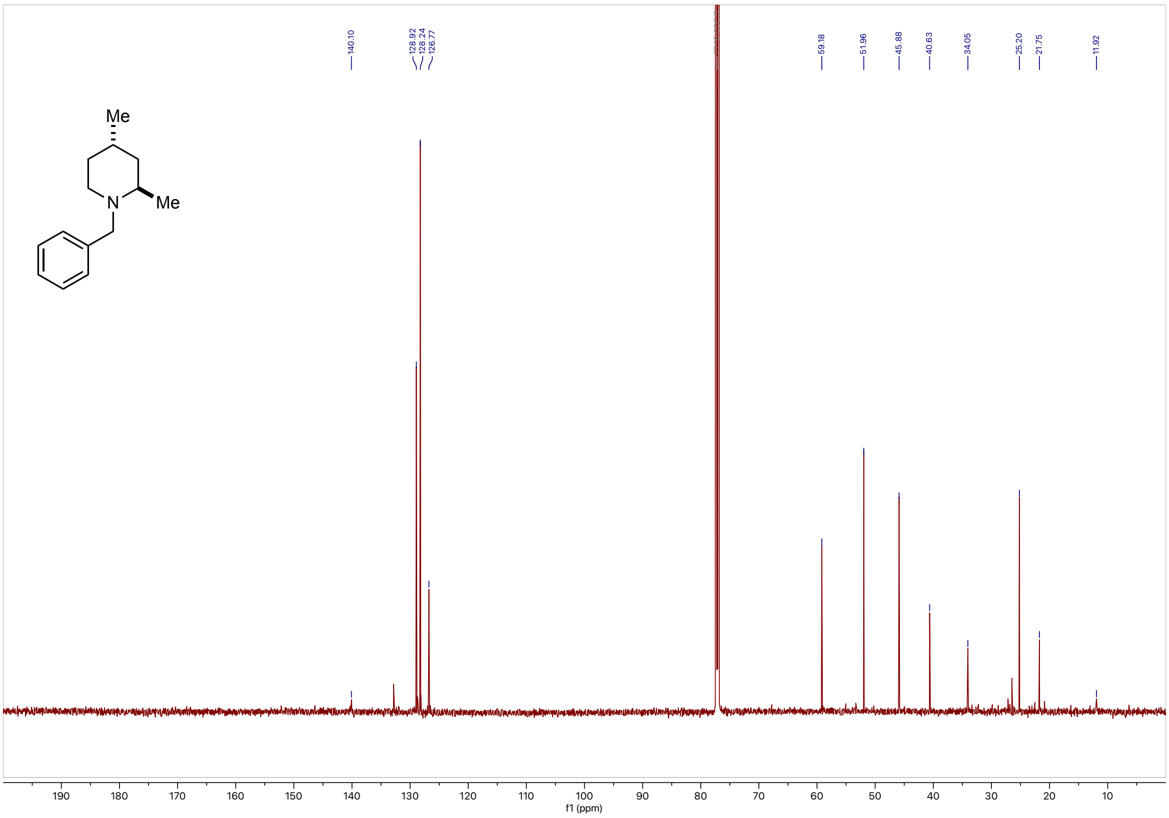

NOSEY (500 MHz, $CDCl_3$) of (*2R,4S*)-1-benzyl-2,4-dimethylpiperidine (**8b**):

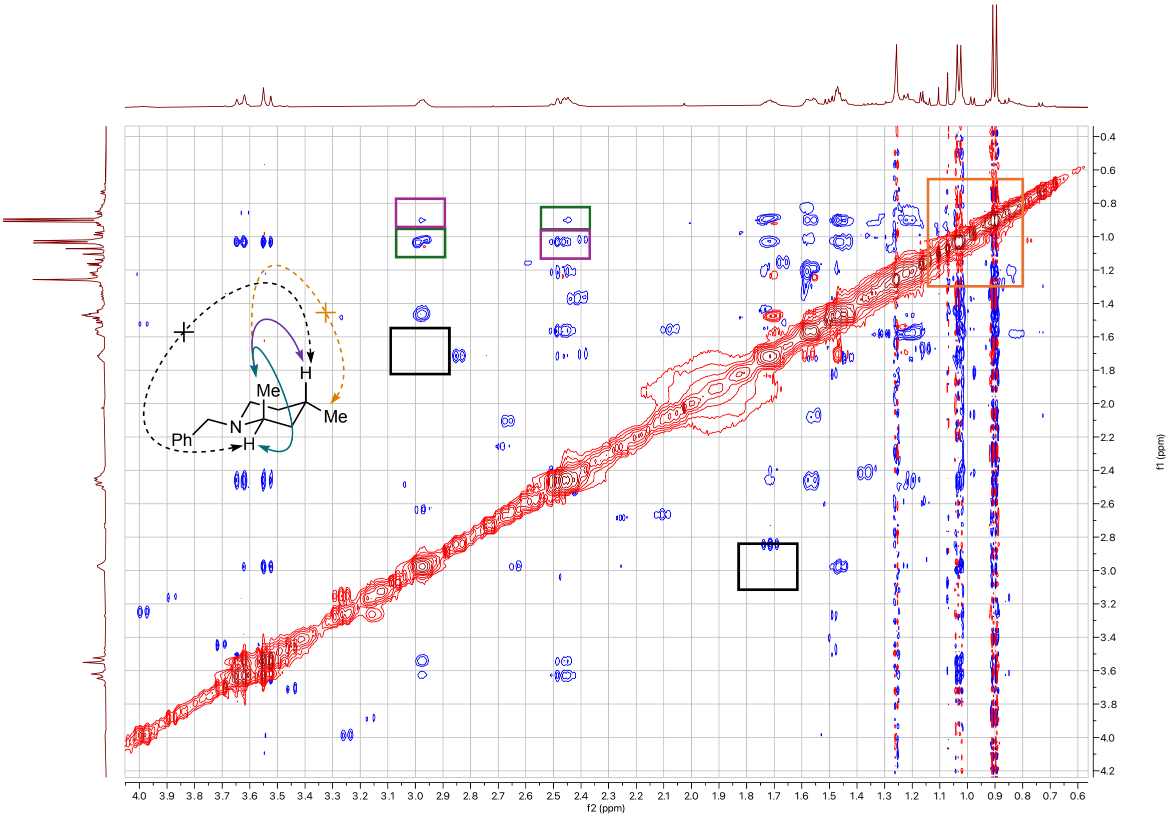

^{1}H NMR (700 MHz, $CDCl_3$) of 2-benzyl-1-methyl-1,2,3,4-tetrahydroisoquinoline (**8d**):

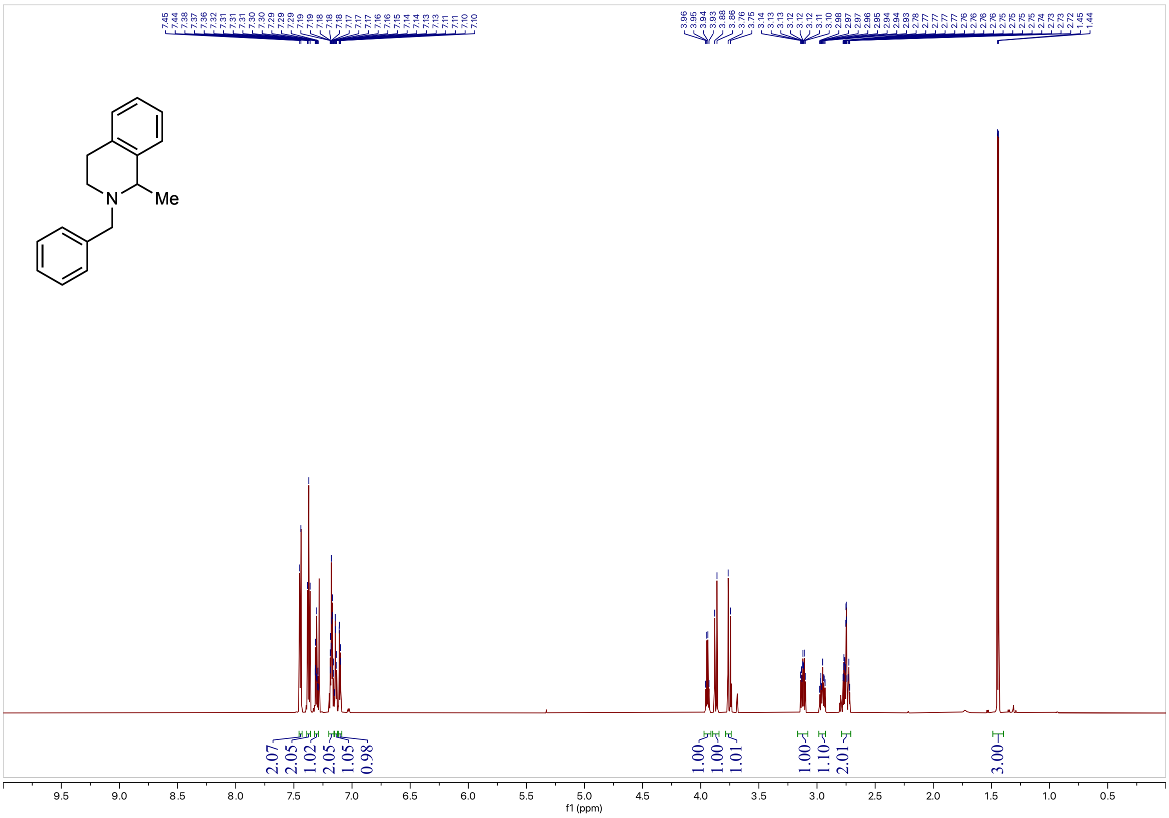

^{13}C{H} NMR (176 MHz, $CDCl_3$) of 2-benzyl-1-methyl-1,2,3,4-tetrahydroisoquinoline (**8d**):

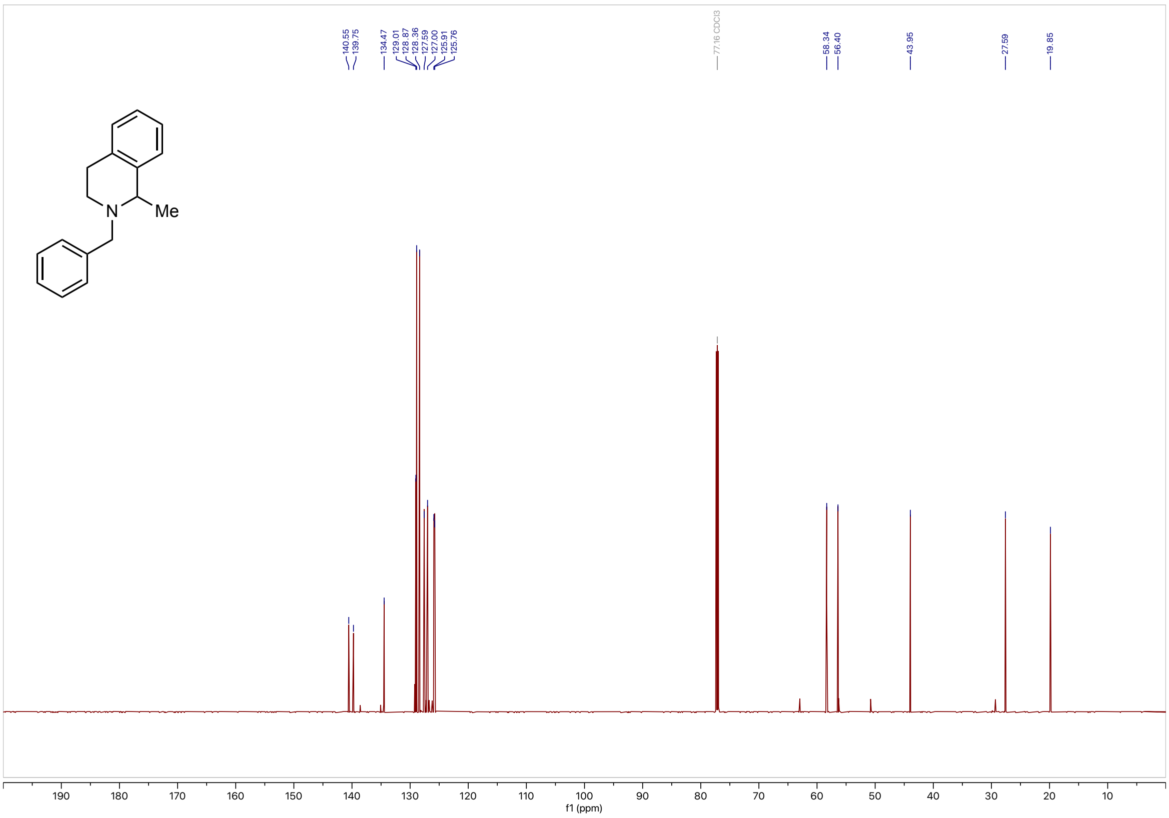

^{1}H NMR (700 MHz, $CDCl_3$) of 1-benzyl-4-methyl-2-(trifluoromethyl)piperidine (**9c**):

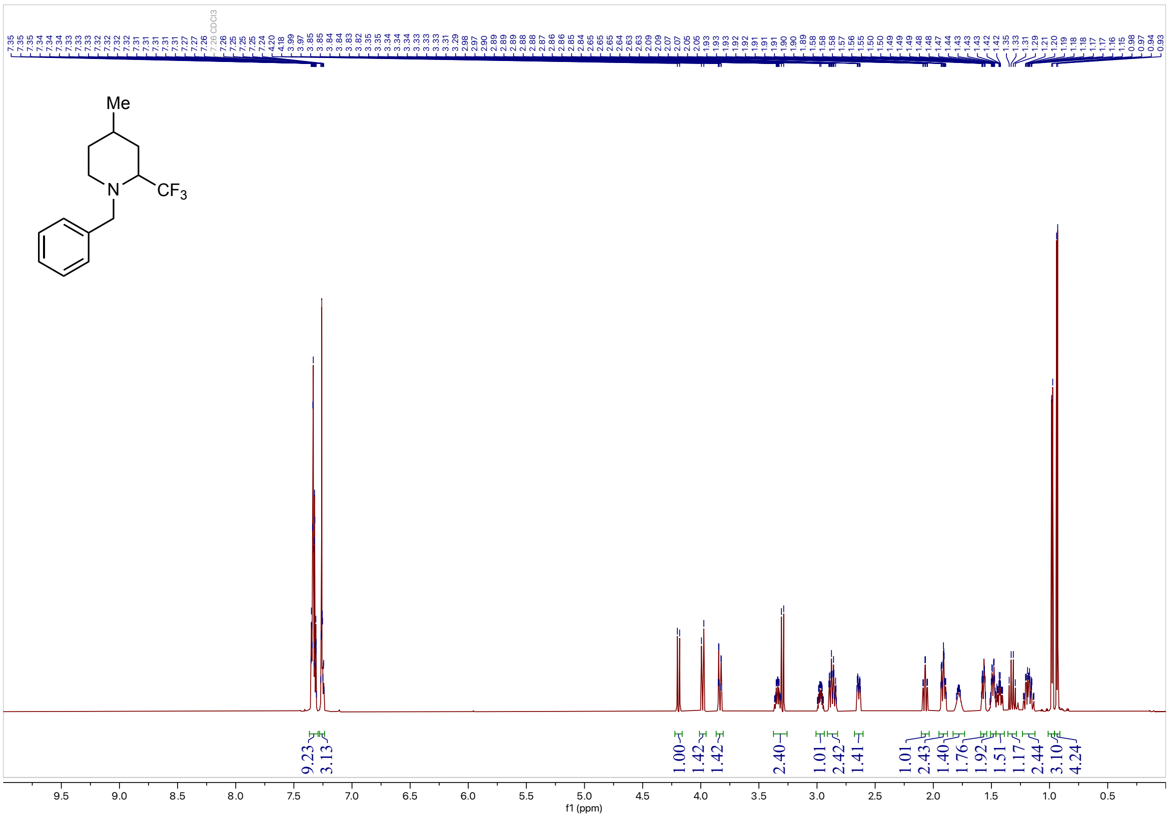

^{13}C{H} NMR (176 MHz, $CDCl_3$) of 1-benzyl-4-methyl-2-(trifluoromethyl)piperidine (**9c**):

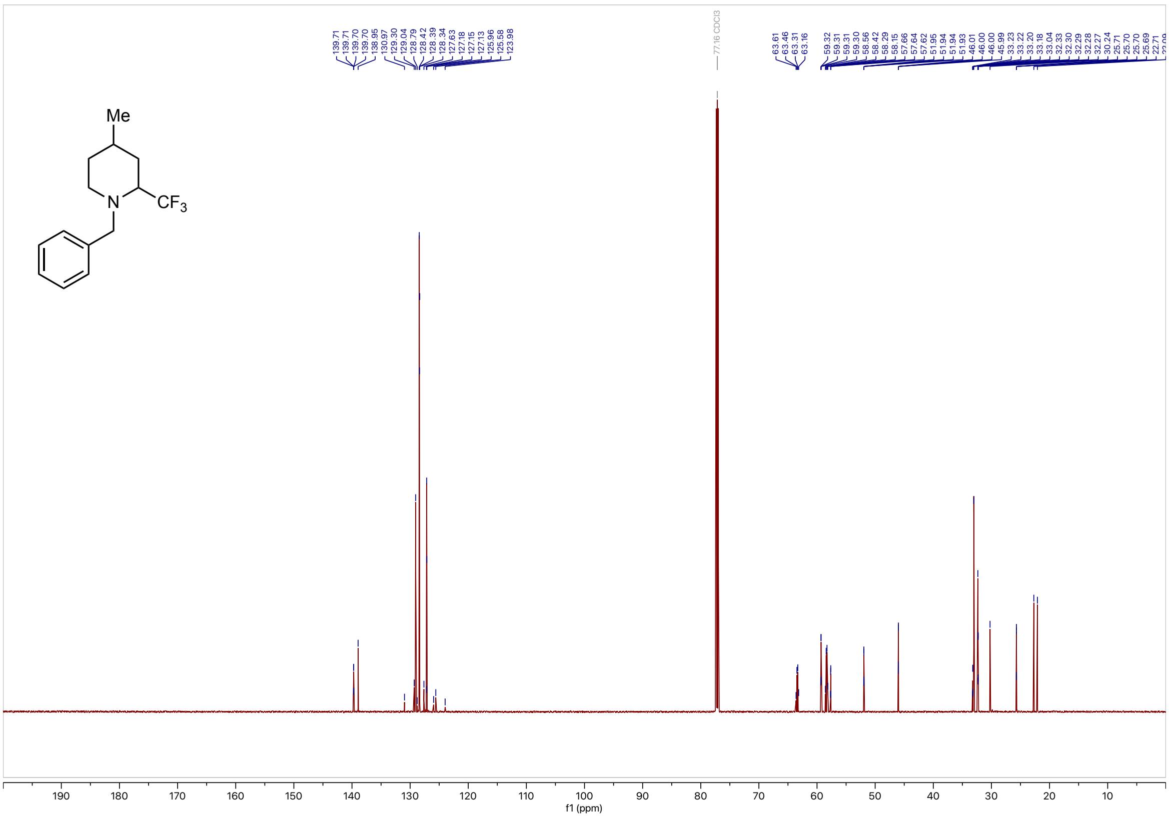

$^{19}F\{H\}$ NMR (376 MHz, $CDCl_3$) of 1-benzyl-4-methyl-2-(trifluoromethyl)piperidine (**9c**):

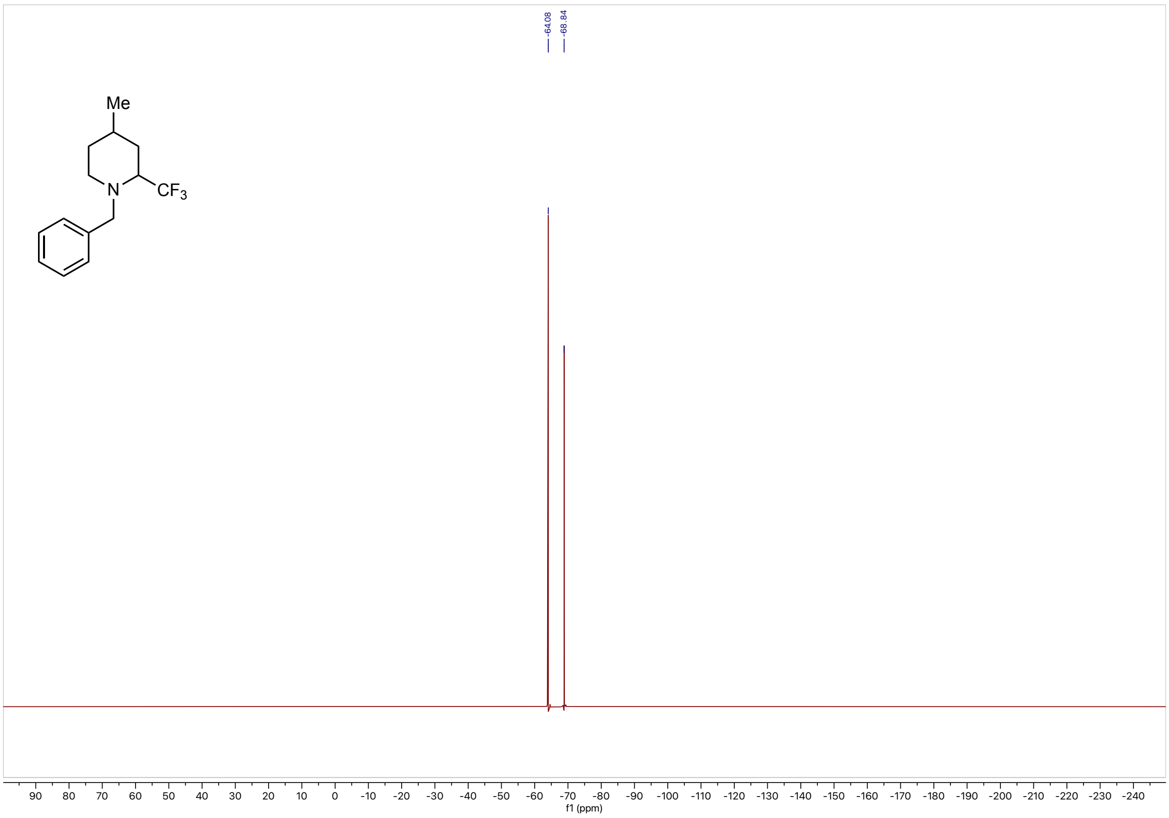

Heteronuclear ^{19}F-^{1}H NOSEY (500 MHz, $CDCl_3$) of (2*S*,4*S*)-1-benzyl-4-methyl-2-(trifluoromethyl)piperidine (**9c**):

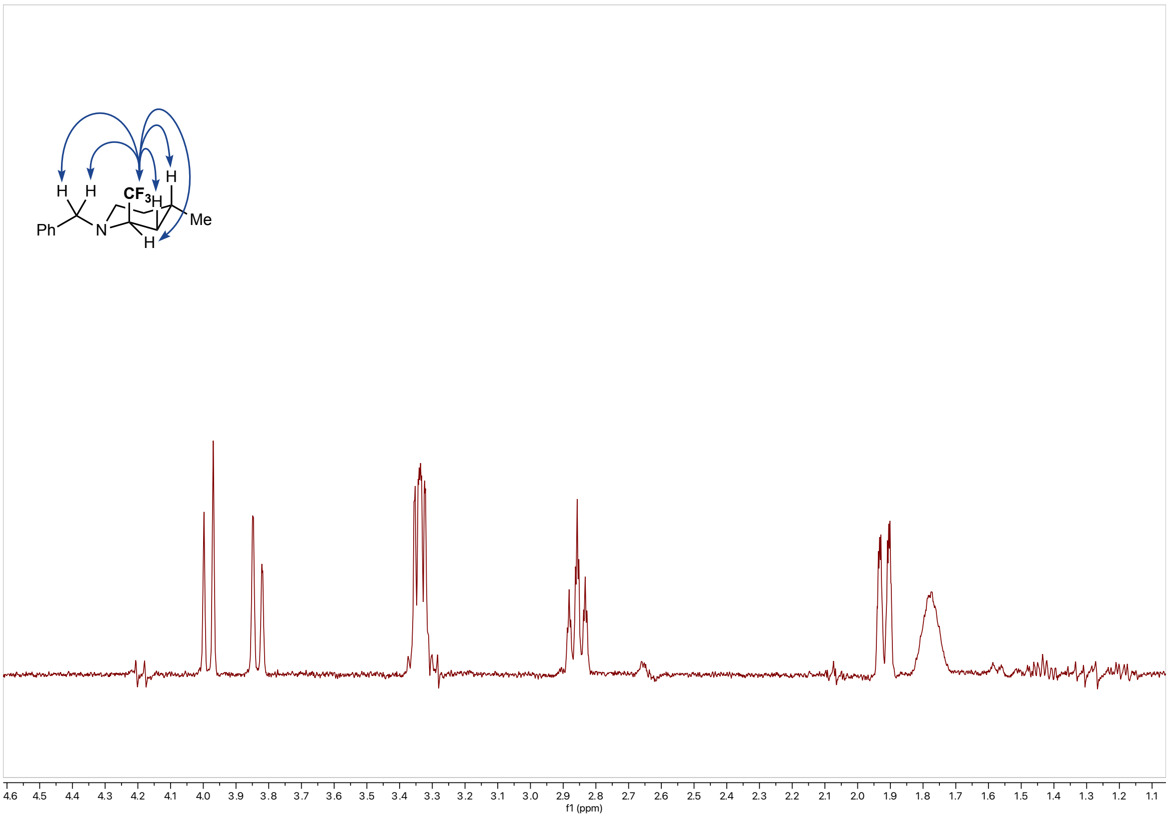

Heteronuclear ^{19}F-^{1}H NOSEY (500 MHz, $CDCl_3$) of (2*R*,4*S*)-1-benzyl-4-methyl-2-(trifluoromethyl)piperidine (**9c**):

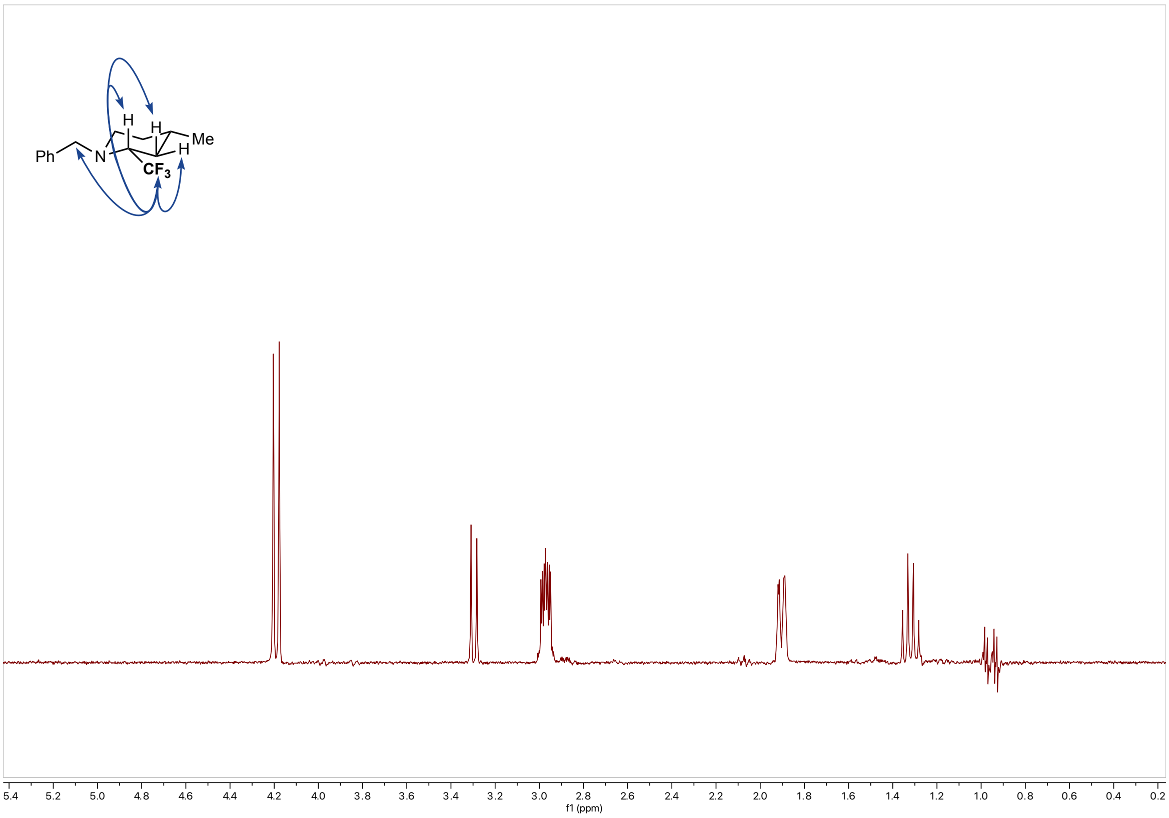

^{1}H NMR (500 MHz, $CDCl_3$) of 3-(1-benzylpiperidin-2-yl)pyridine (**10g**):

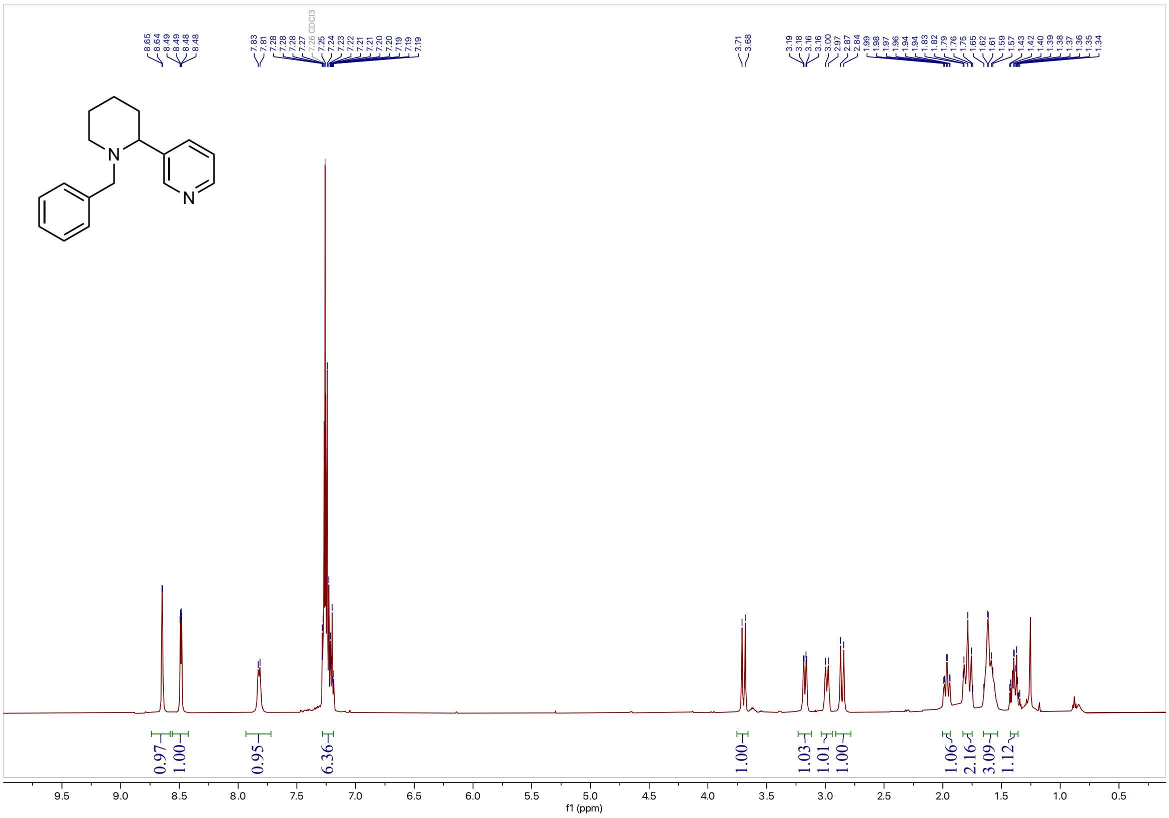

$^{13}C\{H\}$ NMR (126 MHz, $CDCl_3$) of 3-(1-benzylpiperidin-2-yl)pyridine (**10g**):

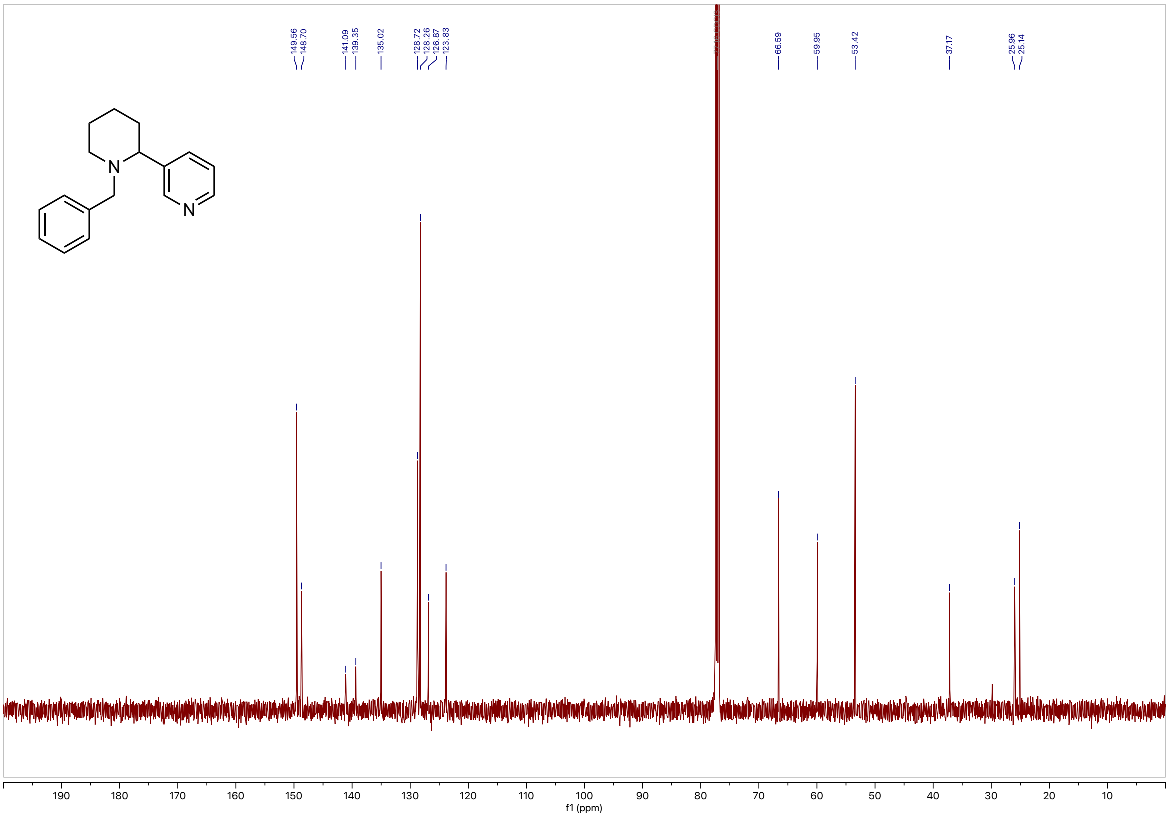

^{1}H NMR (500 MHz, $CDCl_3$) of 6-(1-benzylpiperidin-2-yl)nicotinonitrile (**10h**):

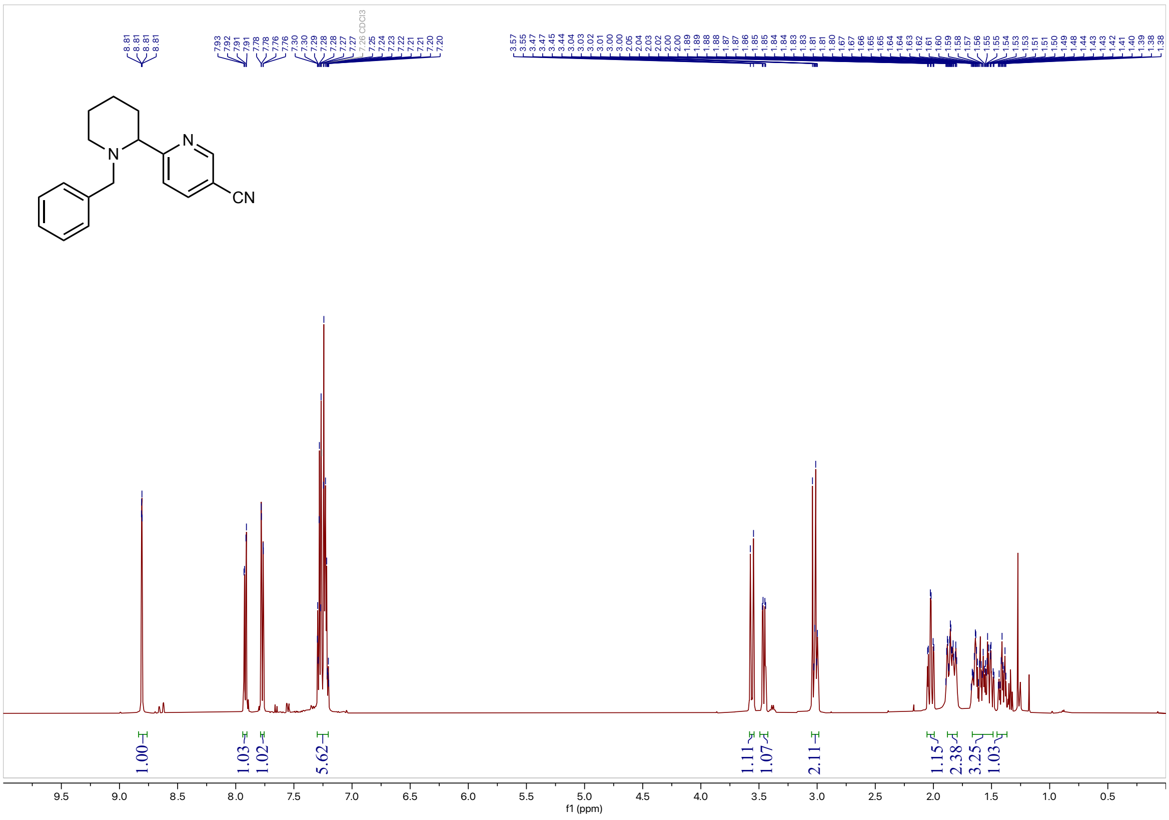

^{13}C{H} NMR (126 MHz, $CDCl_3$) of 6-(1-benzylpiperidin-2-yl)nicotinonitrile (**10h**):

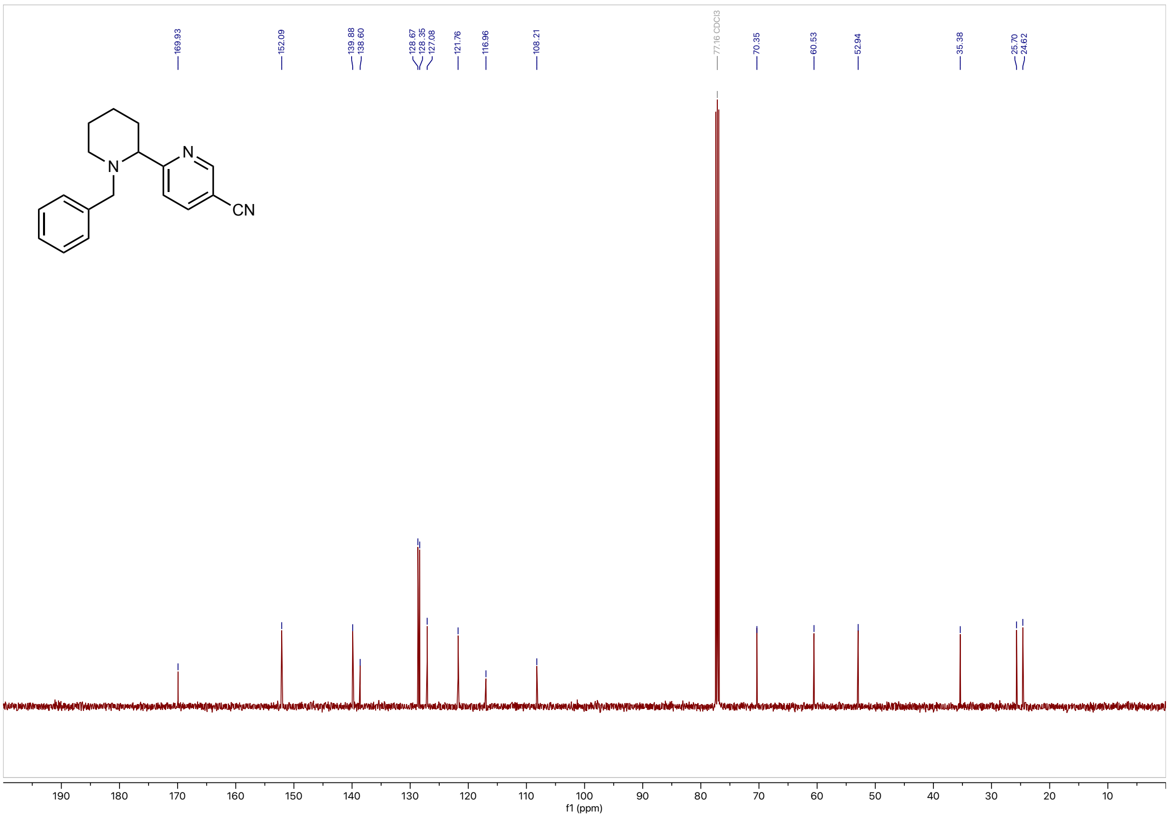

^{1}H NMR (500 MHz, $CDCl_3$) of 3-(1-benzylpiperidin-2-yl)-6-methylpyridazine (**10i**):

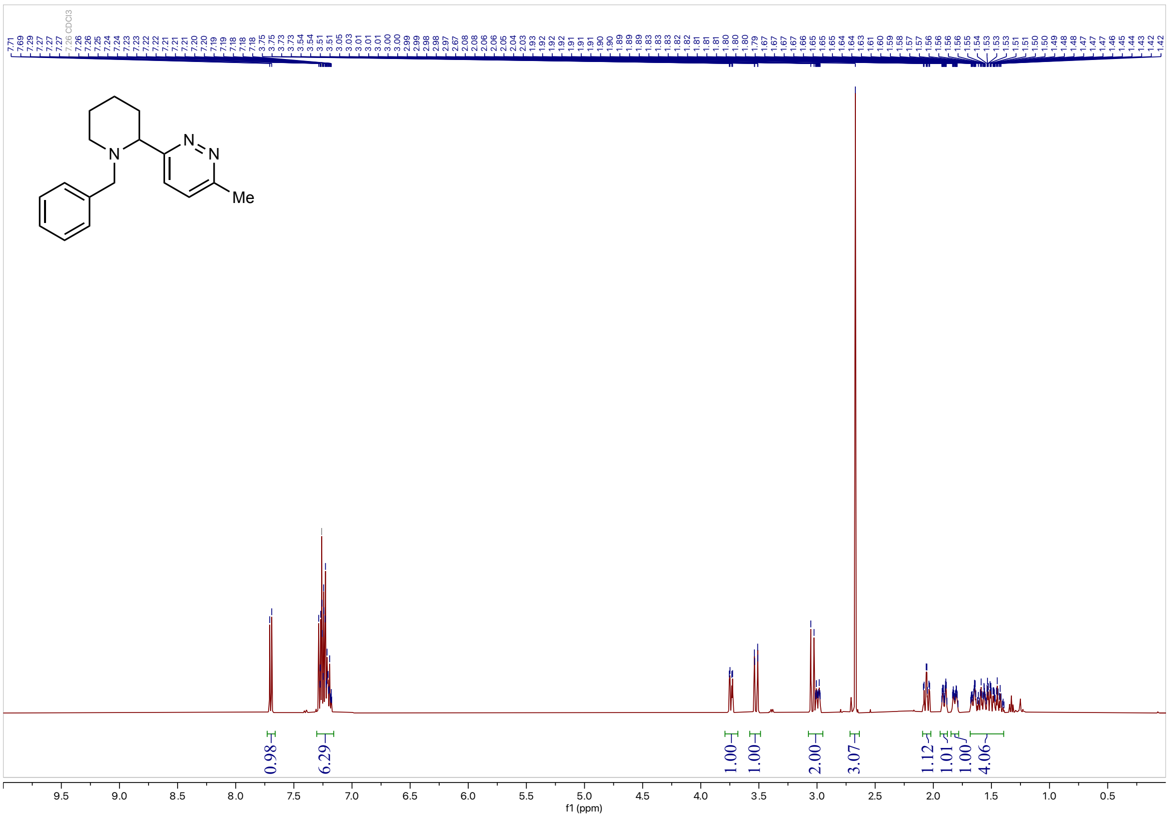

$^{13}C\{H\}$ NMR (126 MHz, $CDCl_3$) of 3-(1-benzylpiperidin-2-yl)-6-methylpyridazine (**10i**):

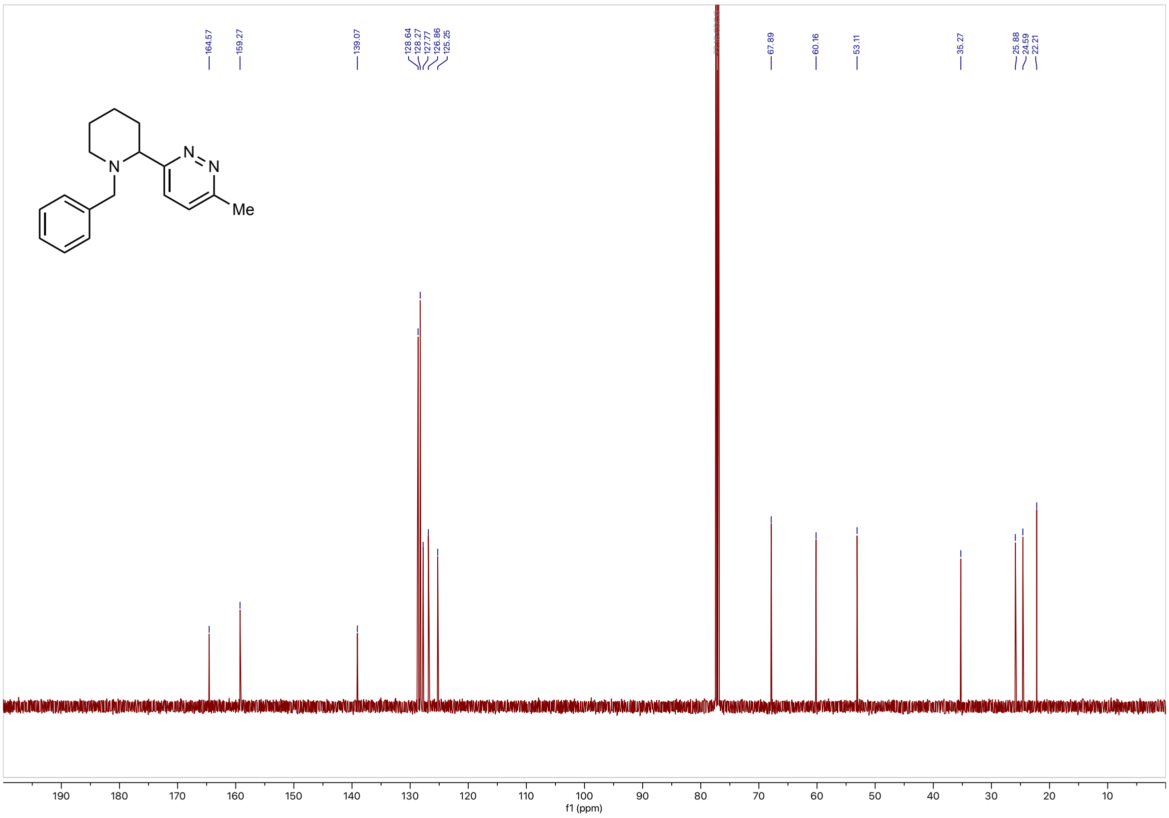

^{1}H NMR (500 MHz, $CDCl_3$) of 1-benzyl-2-(perfluorophenyl)-piperidine (**10j**):

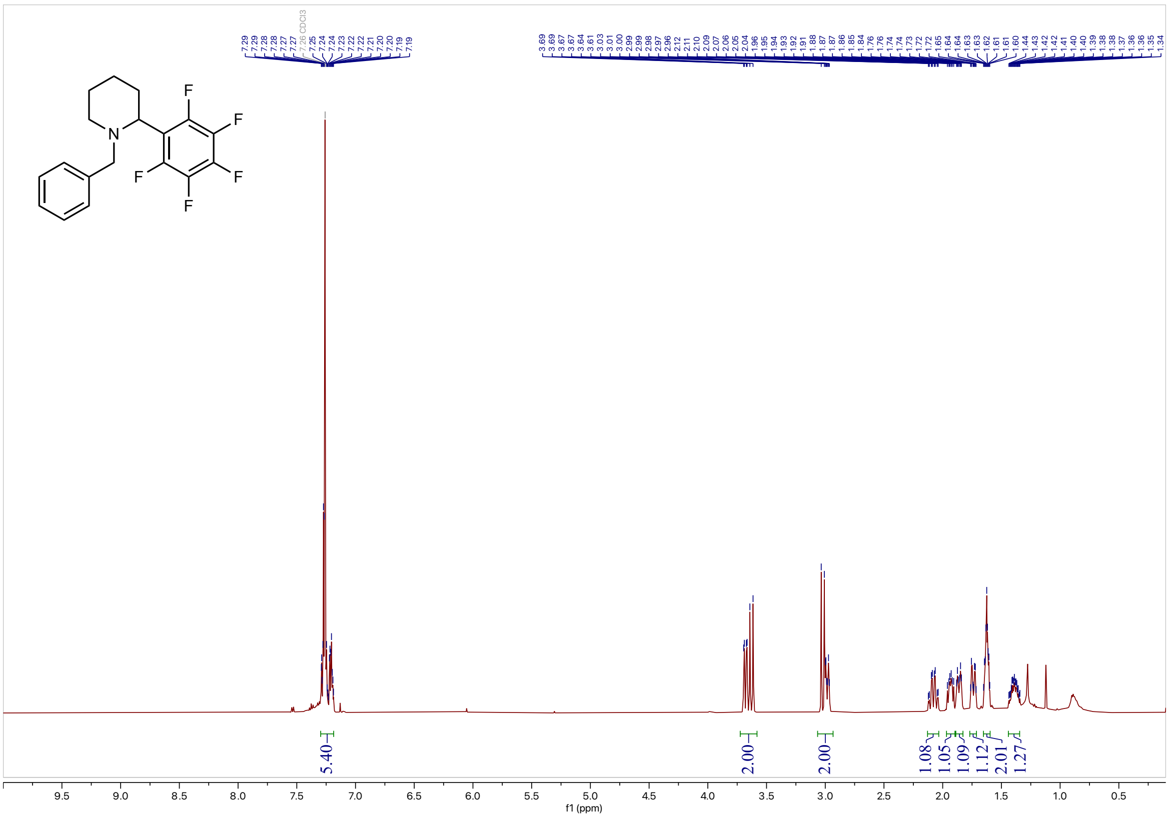

$^{13}C\{H\}$ NMR (126 MHz, $CDCl_3$) of 1-benzyl-2-(perfluorophenyl)-piperidine (**10j**):

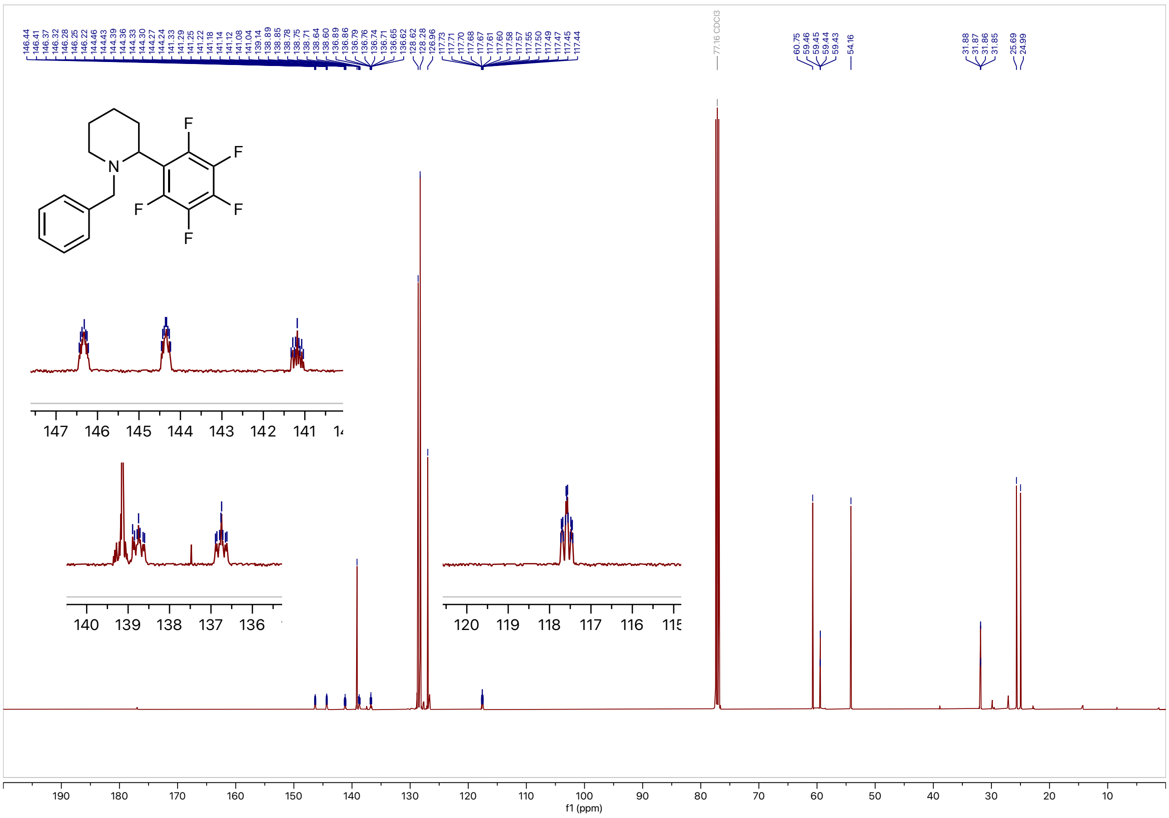

$^{19}F\{H\}$ NMR (471 MHz, $CDCl_3$) of 1-benzyl-2-(perfluorophenyl)-piperidine (**10j**):

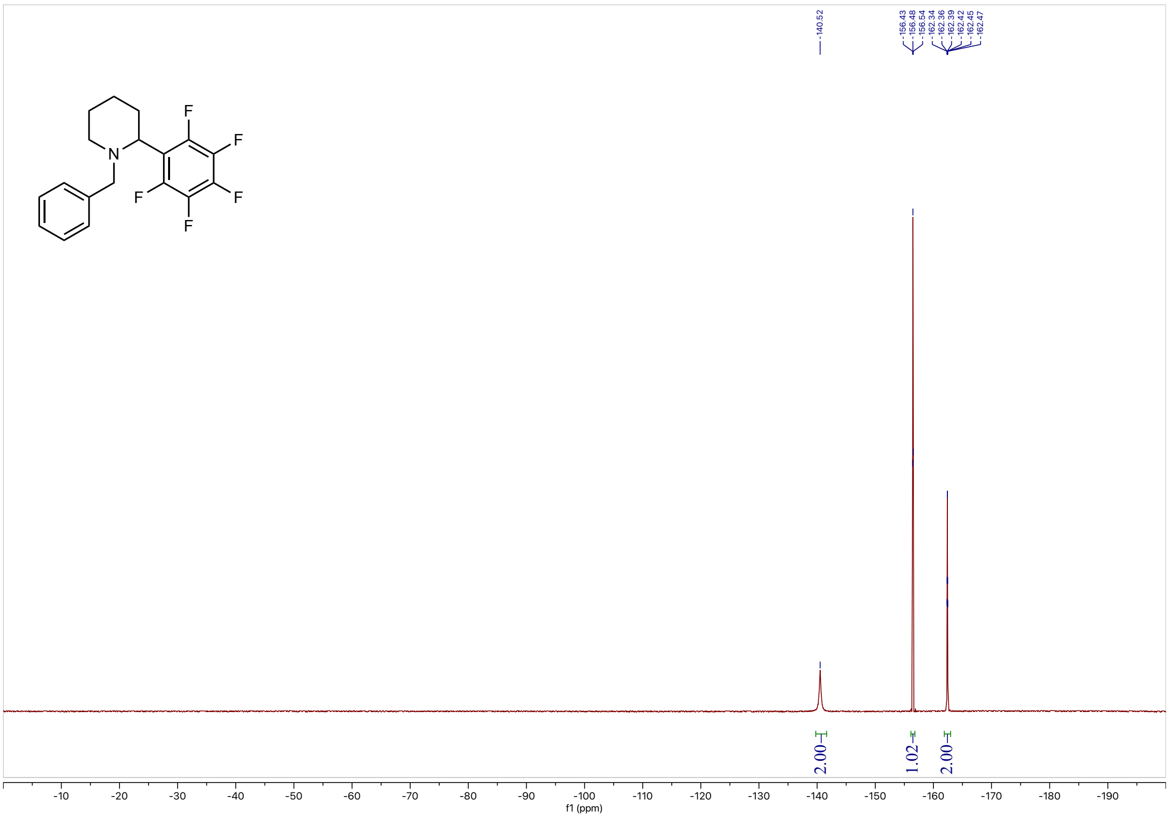

^{1}H NMR (400 MHz, $CDCl_3$) of *1*-benzyl-5-(*1*-benzylpiperidine-2-yl)-1,2,3,4-tetrahydropyridine (**24**):

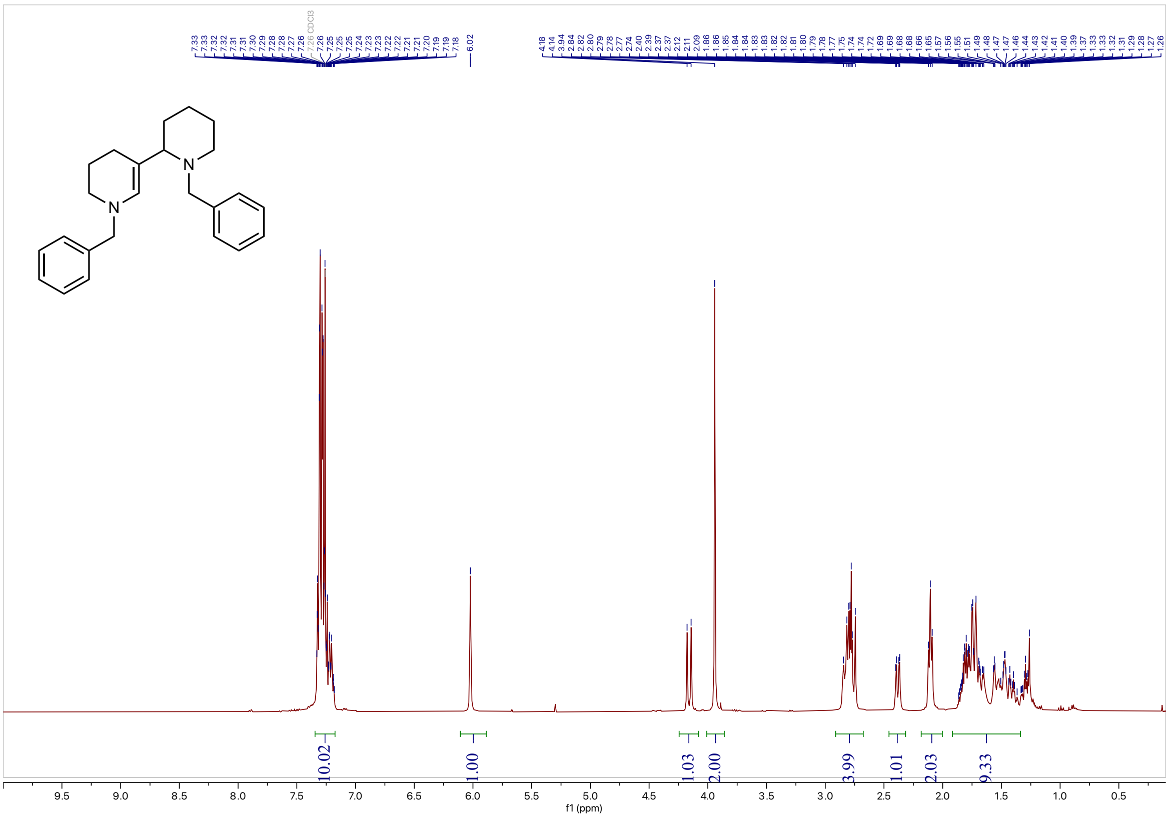

^{13}C{H} NMR (101 MHz, $CDCl_3$) of *1*-benzyl-5-(*1*-benzylpiperidine-2-yl)-1,2,3,4-tetrahydropyridine (**24**):

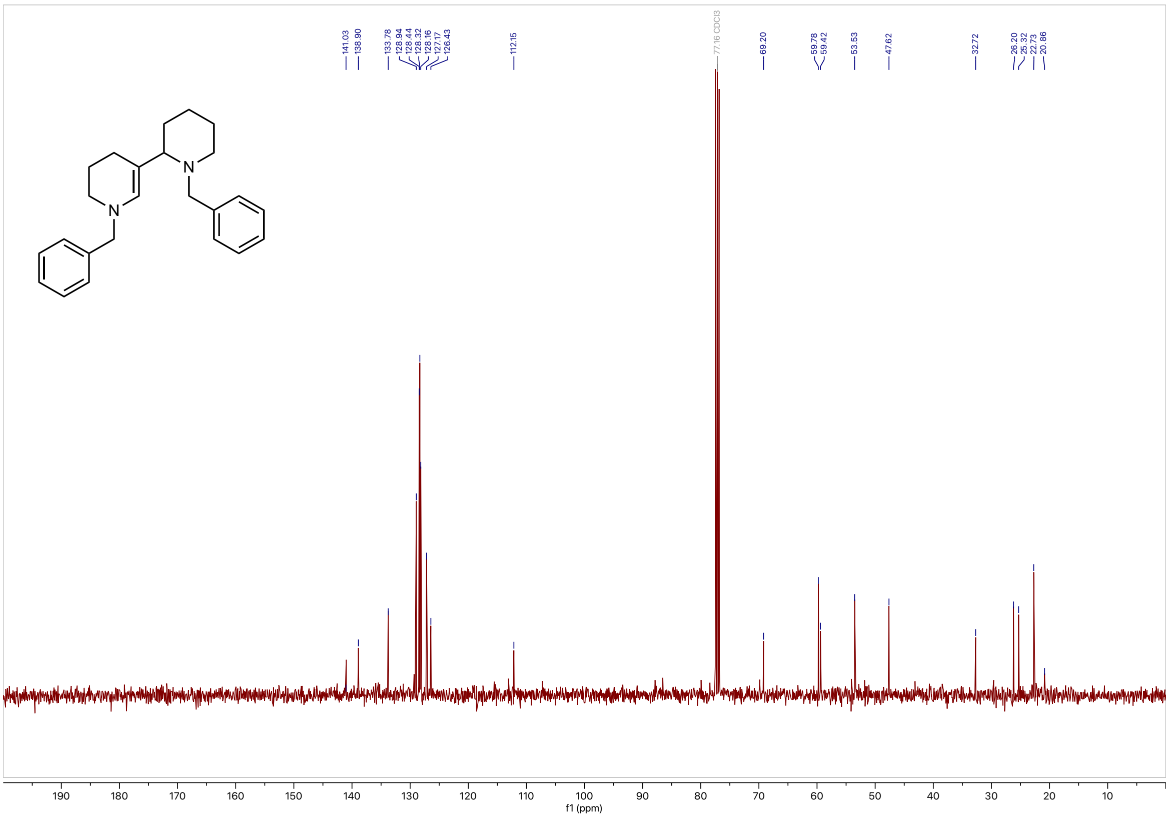

^{1}H NMR (700 MHz, $CDCl_3$) of *N*-((Z)-5-(2-((*1R,5S*)-bicyclo[3.3.1]nonan-3-yl)-1-phenylpiperidin-3-ylidene)pentyl)aniline (**25**)

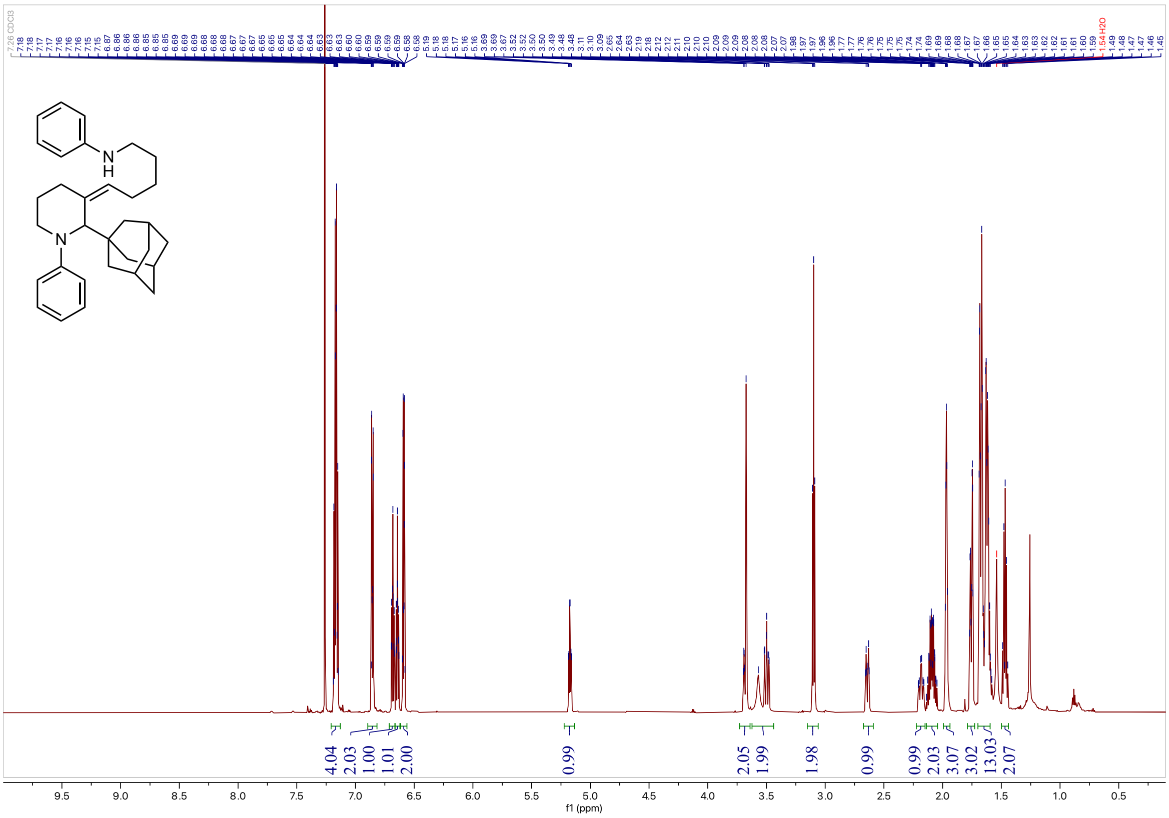

^{13}C{H} NMR (126 MHz, $CDCl_3$) of *N*-((Z)-5-(2-((*1R,5S*)-bicyclo[3.3.1]nonan-3-yl)-1-phenylpiperidin-3-ylidene)pentyl)aniline (**25**):

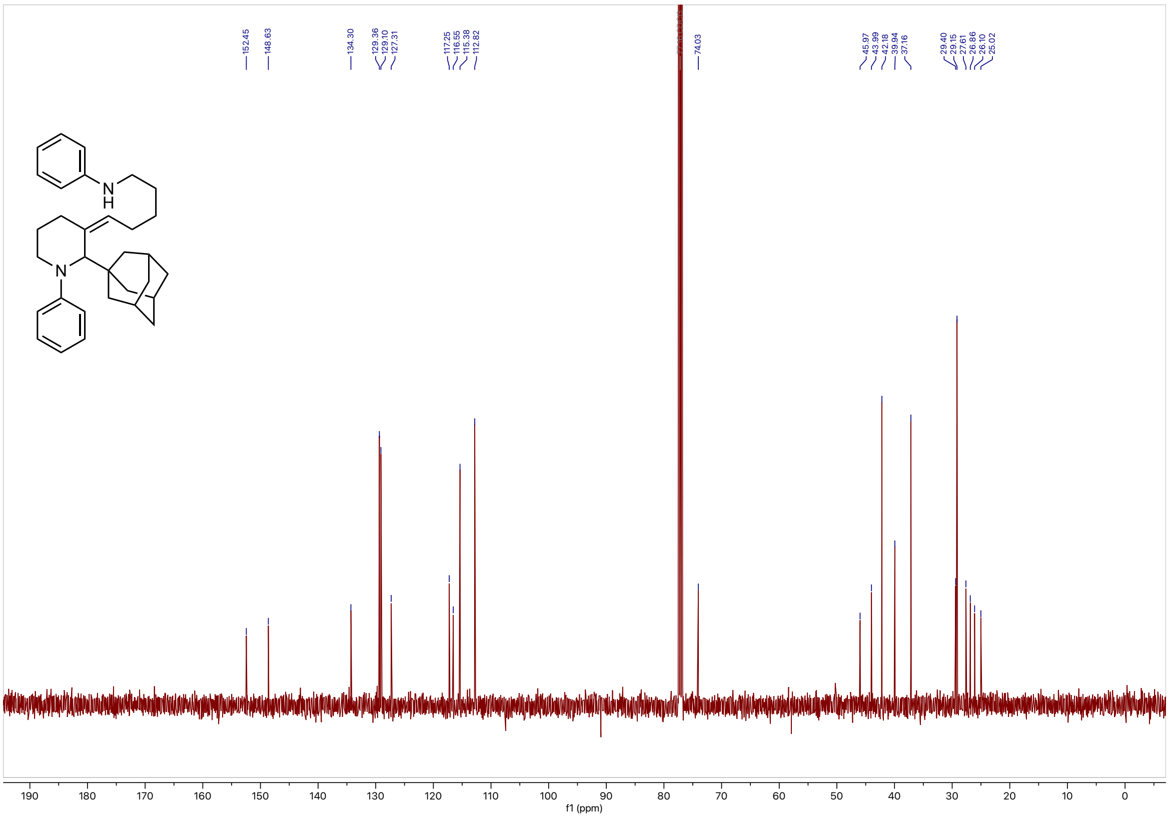

^{1}H NMR (500 MHz, CD_2Cl_2) of 1-(2-((3-methyl-4-oxo-2-phenyl-4*H*-chromene-8-carbonyl)oxy)ethyl)-2,3,4,5-tetrahydropyridin-1-ium chloride (**26**):

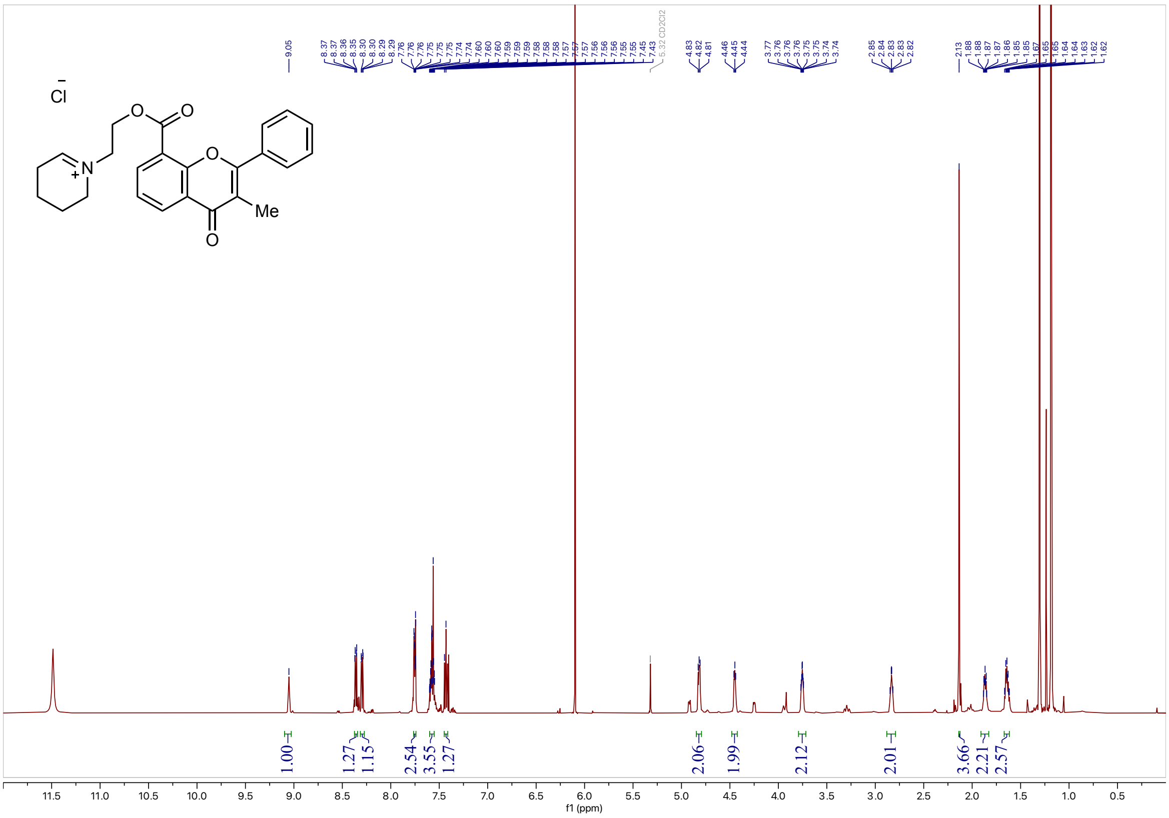

$^{13}C\{H\}$ NMR (126 MHz, CD_2Cl_2) of 1-(2-((3-methyl-4-oxo-2-phenyl-4*H*-chromene-8-carbonyl)oxy)ethyl)-2,3,4,5-tetrahydropyridin-1-ium chloride (**26**):

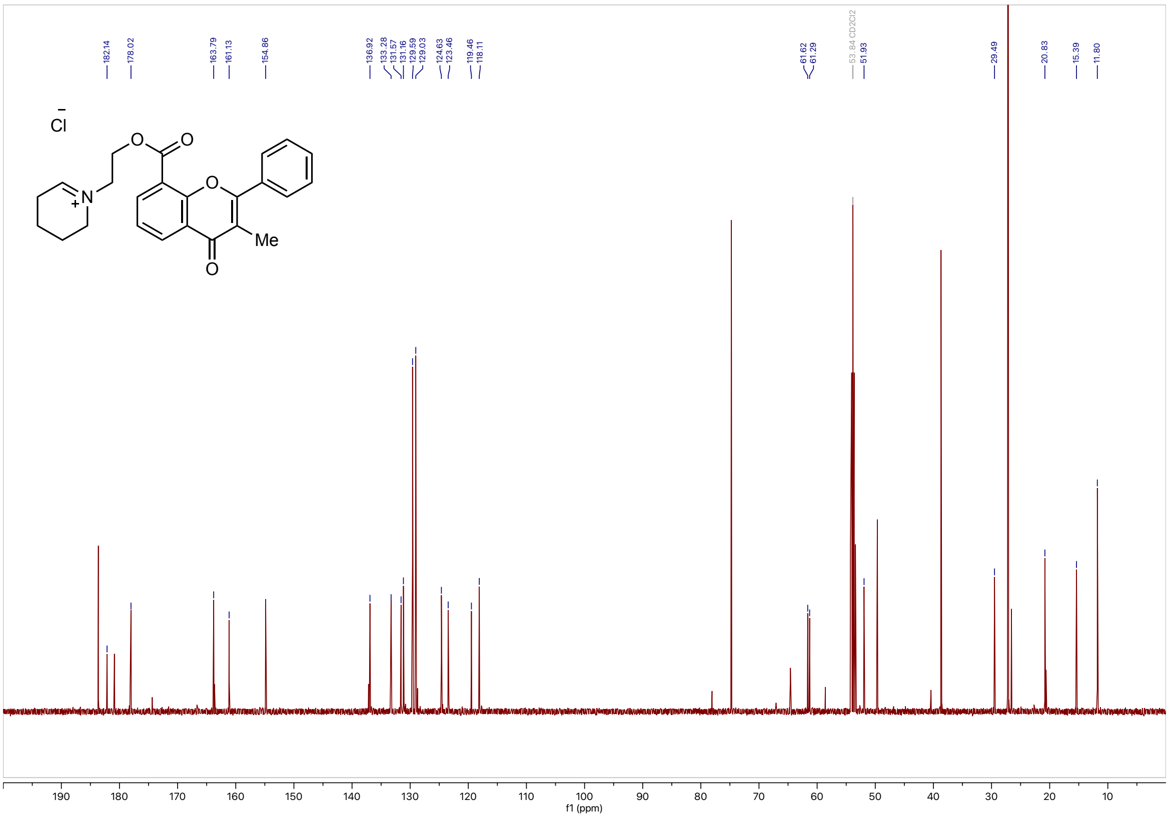

^{1}H NMR (700 MHz, $CDCl_3$) of 2-(2-methylpiperidin-1-yl)ethyl 3-methyl-4-oxo-2-phenyl-4*H*-chromene-8-carboxylate (**27a**):

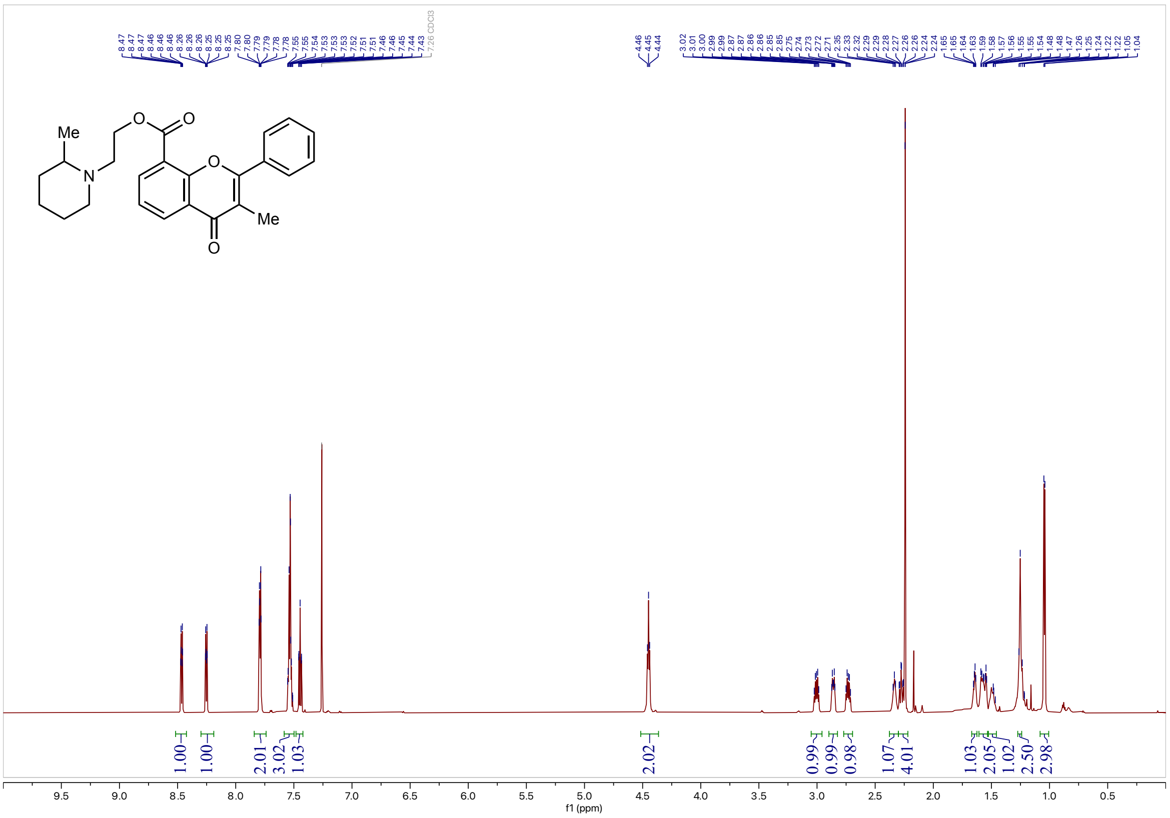

^{13}C{H} NMR (126 MHz, $CDCl_3$) of 2-(2-methylpiperidin-1-yl)ethyl 3-methyl-4-oxo-2-phenyl-4*H*-chromene-8-carboxylate (**27a**):

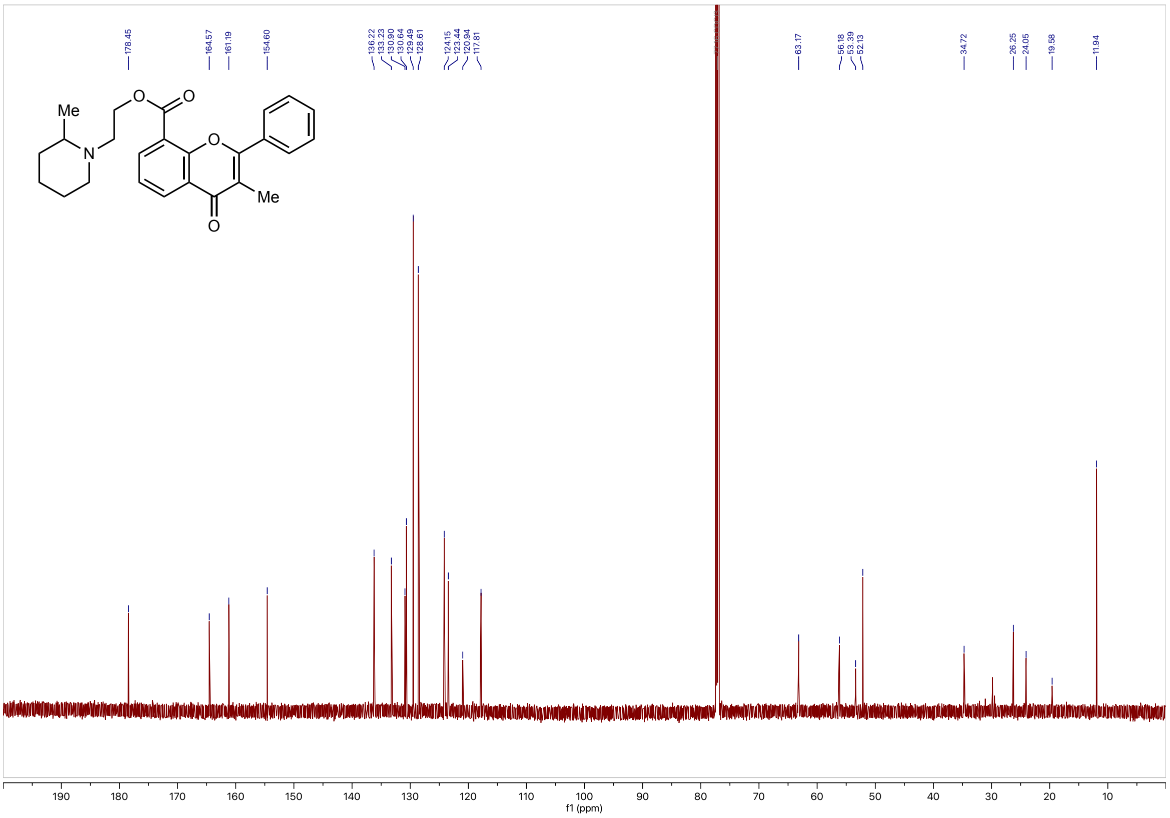

^{1}H NMR (500 MHz, $CDCl_3$) of 2-(2-(methyl-d_3)piperidin-1-yl)ethyl 3-methyl-4-oxo-2-phenyl-4*H*-chromene-8-carboxylate (***d*₃-27a**):

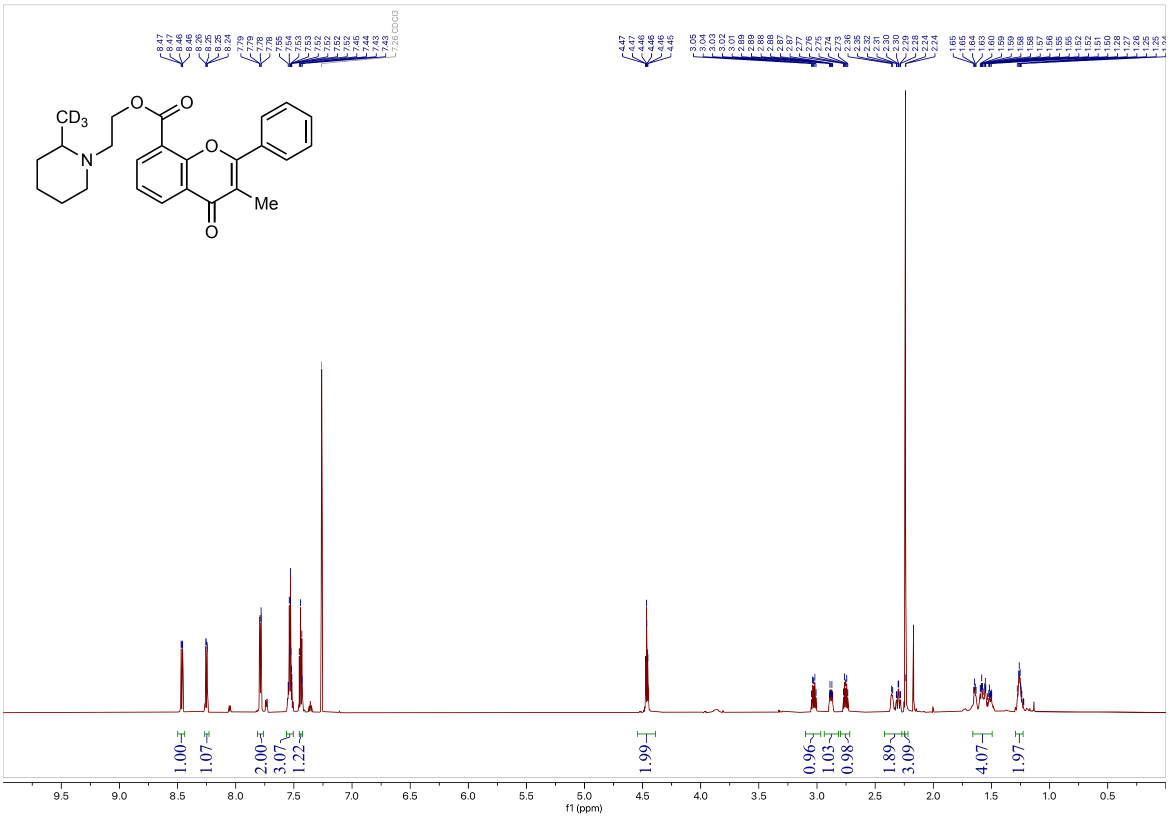

$^{13}C\{H\}$ NMR (126 MHz, $CDCl_3$) of 2-(2-(methyl-d_3)piperidin-1-yl)ethyl 3-methyl-4-oxo-2-phenyl-4*H*-chromene-8-carboxylate (***d*₃-27a**):

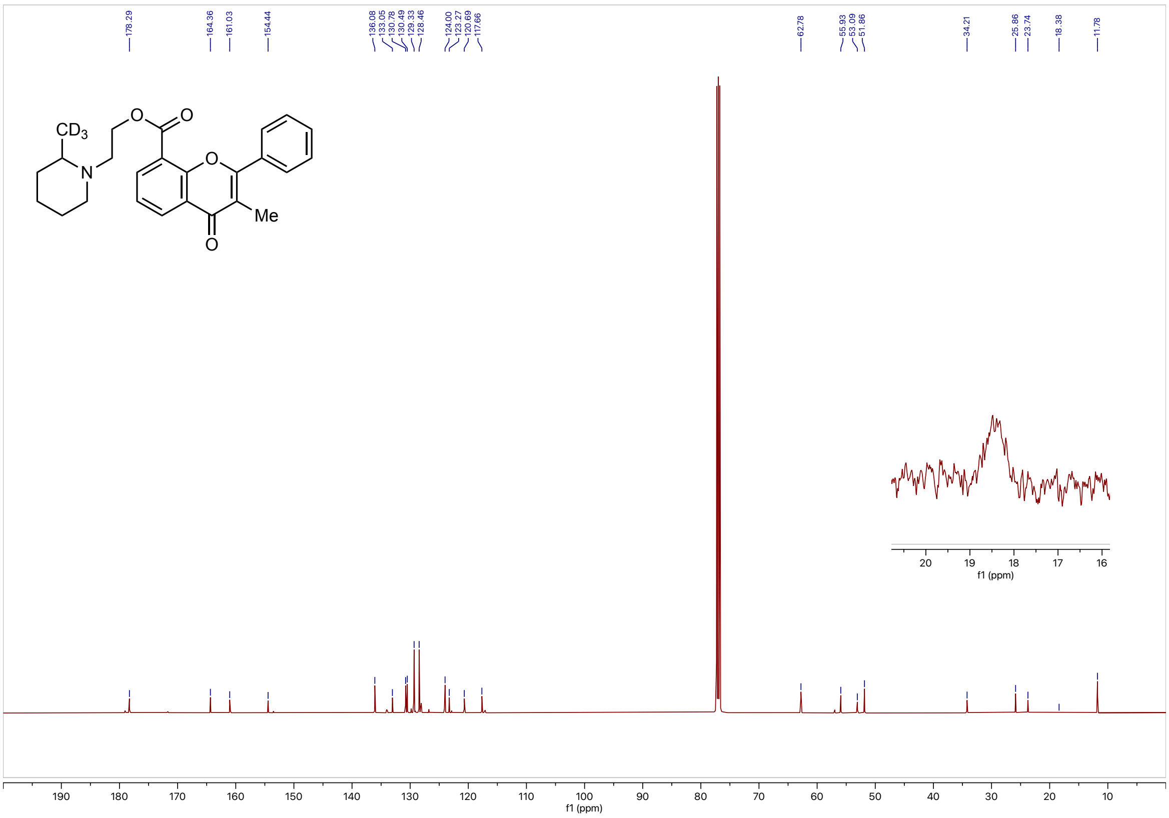

^{1}H NMR (500 MHz, $CDCl_3$) of 2-(2-((benzoyloxy)methyl)piperidin-1-yl)ethyl 3-methyl-4-oxo-2phenyl-4*H*-chromene-8-carboxylate (**27b**):

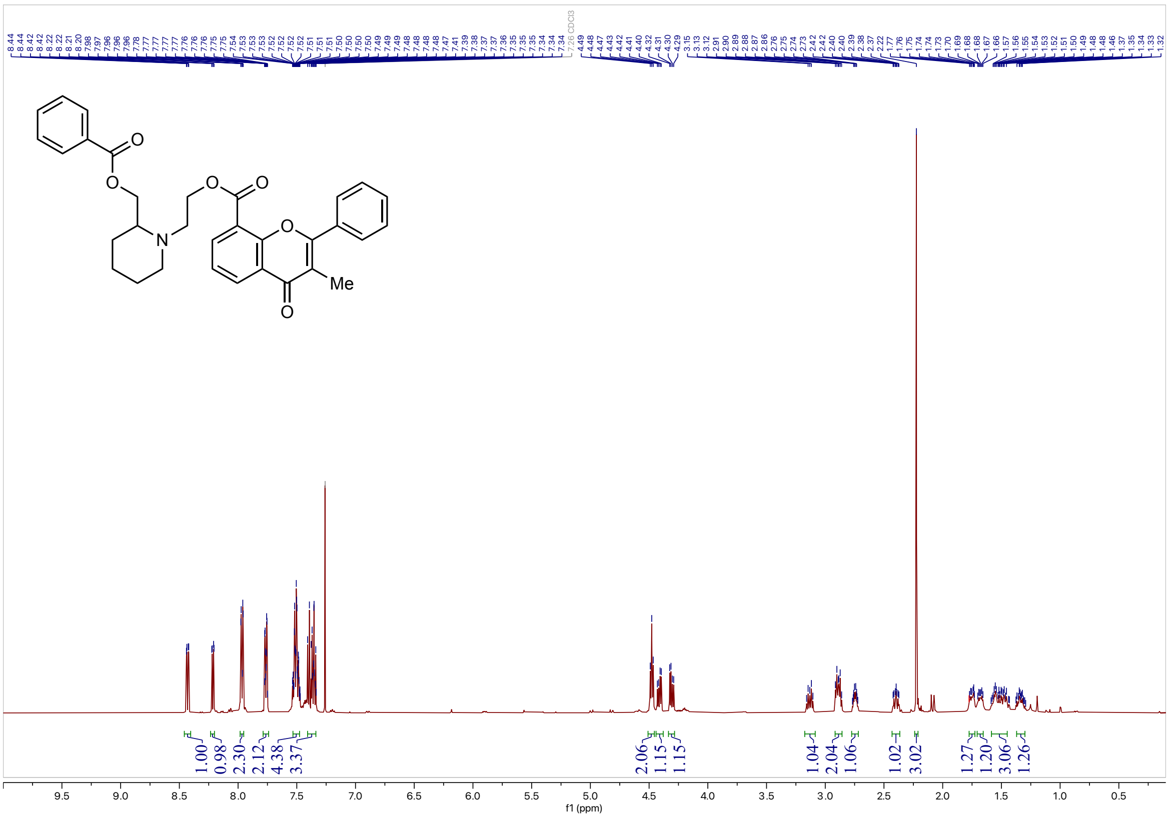

$^{13}C\{H\}$ NMR (126 MHz, $CDCl_3$) of 2-(2-((benzoyloxy)methyl)piperidin-1-yl)ethyl 3-methyl-4-oxo-2phenyl-4*H*-chromene-8-carboxylate (**27b**):

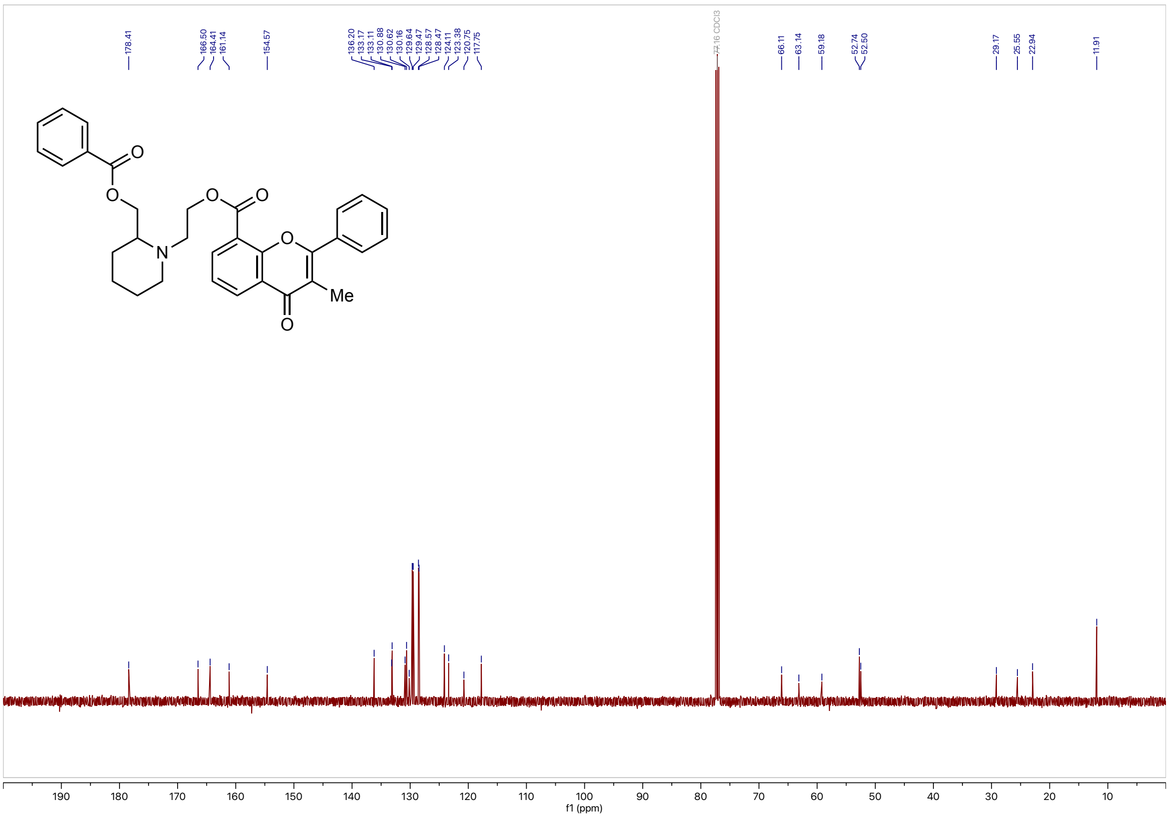

^{1}H NMR (700 MHz, $CDCl_3$) of 2-(2-fluoromethyl)piperidin-1-yl)ethyl 3-methyl-4-oxo-2-phenyl-4*H*-chromene-8-carboxylate (**27c**):

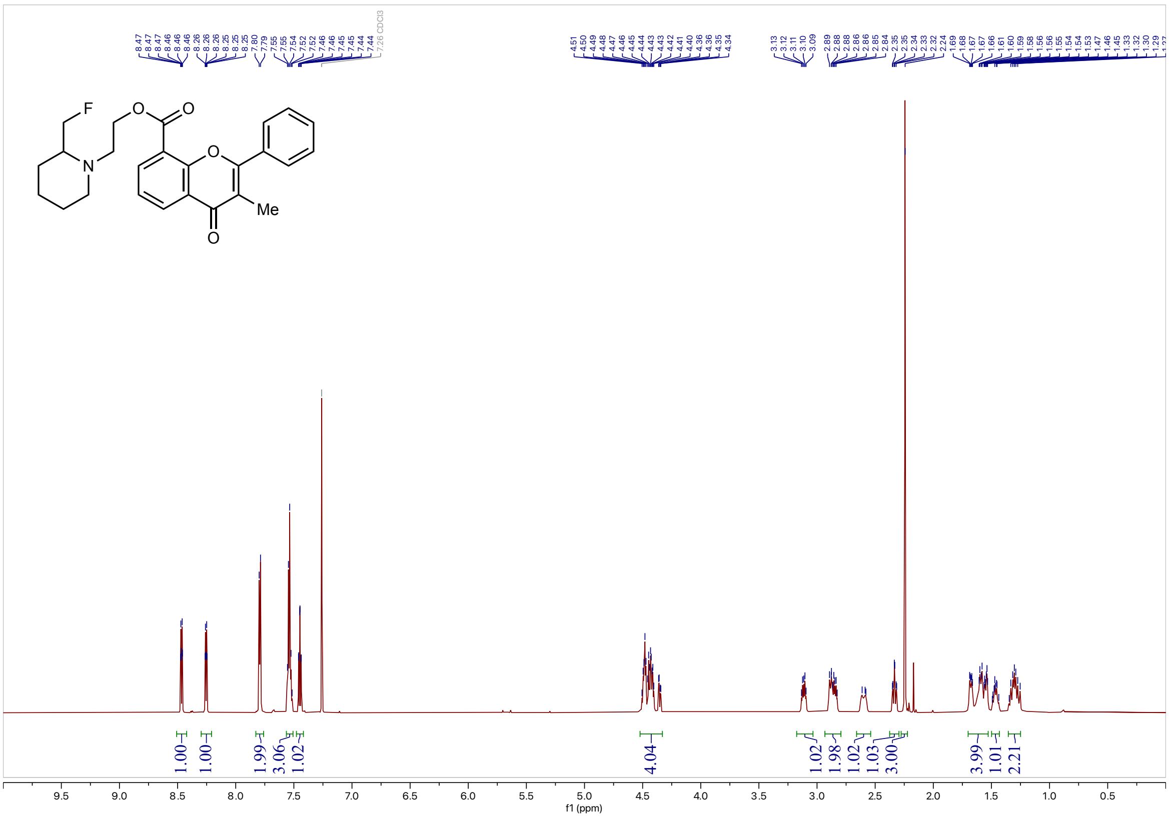

$^{13}C\{H\}$ NMR (126 MHz, $CDCl_3$) of 2-(2-fluoromethyl)piperidin-1-yl)ethyl 3-methyl-4-oxo-2-phenyl-4*H*-chromene-8-carboxylate (**27c**):

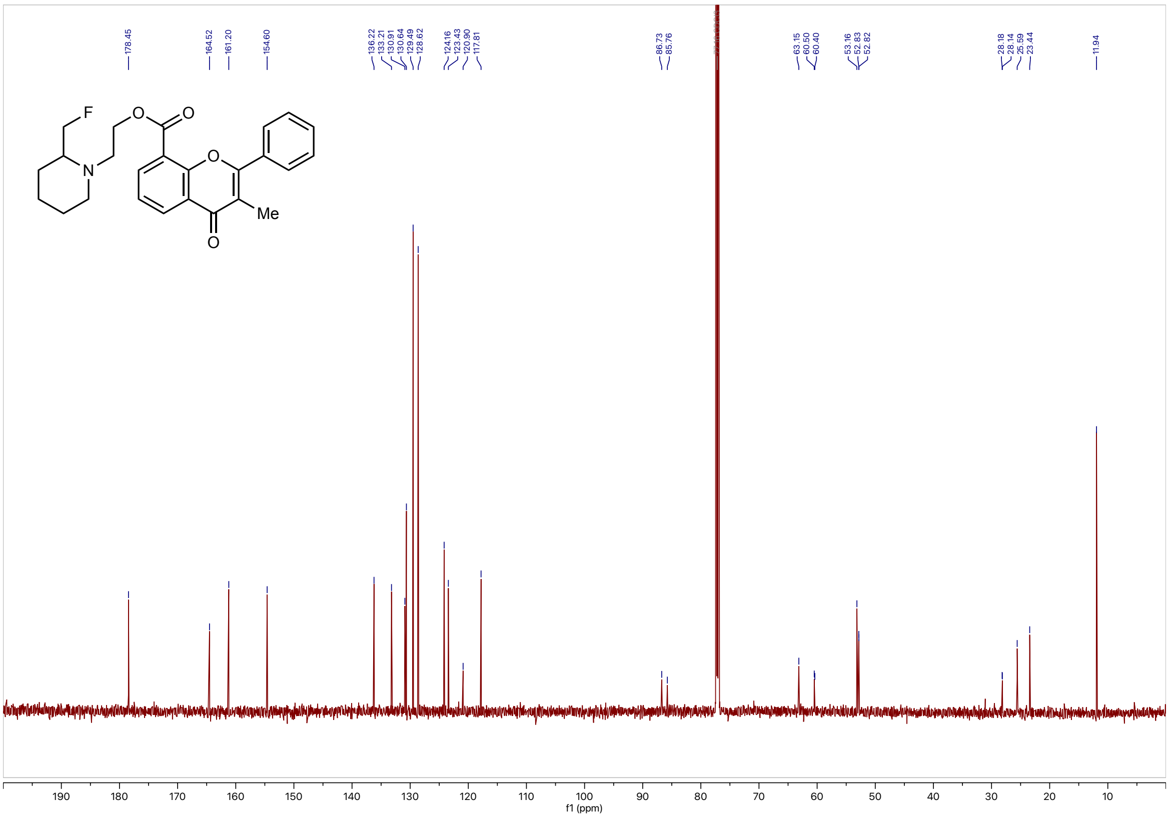

$^{19}F\{H\}$ NMR (471 MHz, $CDCl_3$) of 2-(2-fluoromethyl)piperidin-1-yl)ethyl 3-methyl-4-oxo-2-phenyl-4*H*-chromene-8-carboxylate (**27c**):

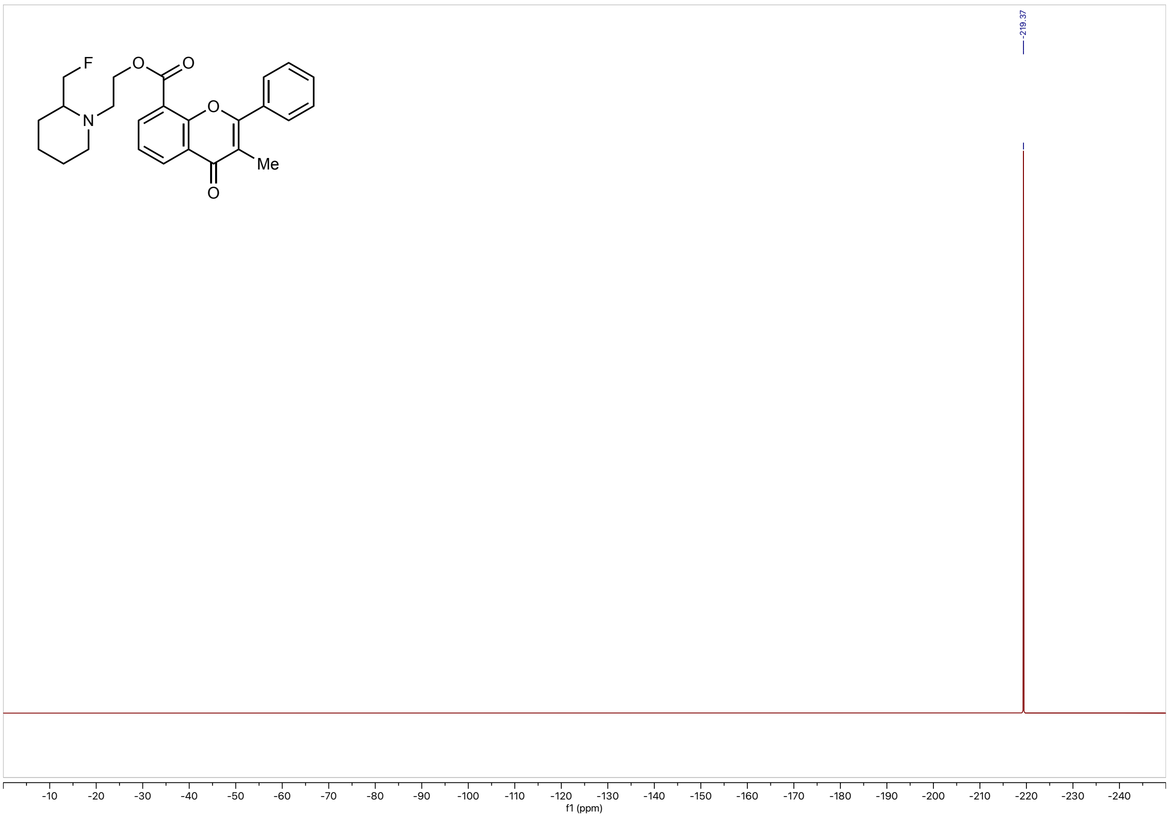

^{1}H NMR (700 MHz, $CDCl_3$) of 2-(2-isopropylpiperidin-1-yl)ethyl 3-methyl-4-oxo-2-phenyl-4*H*-chromene-8-carboxylate (**27d**):

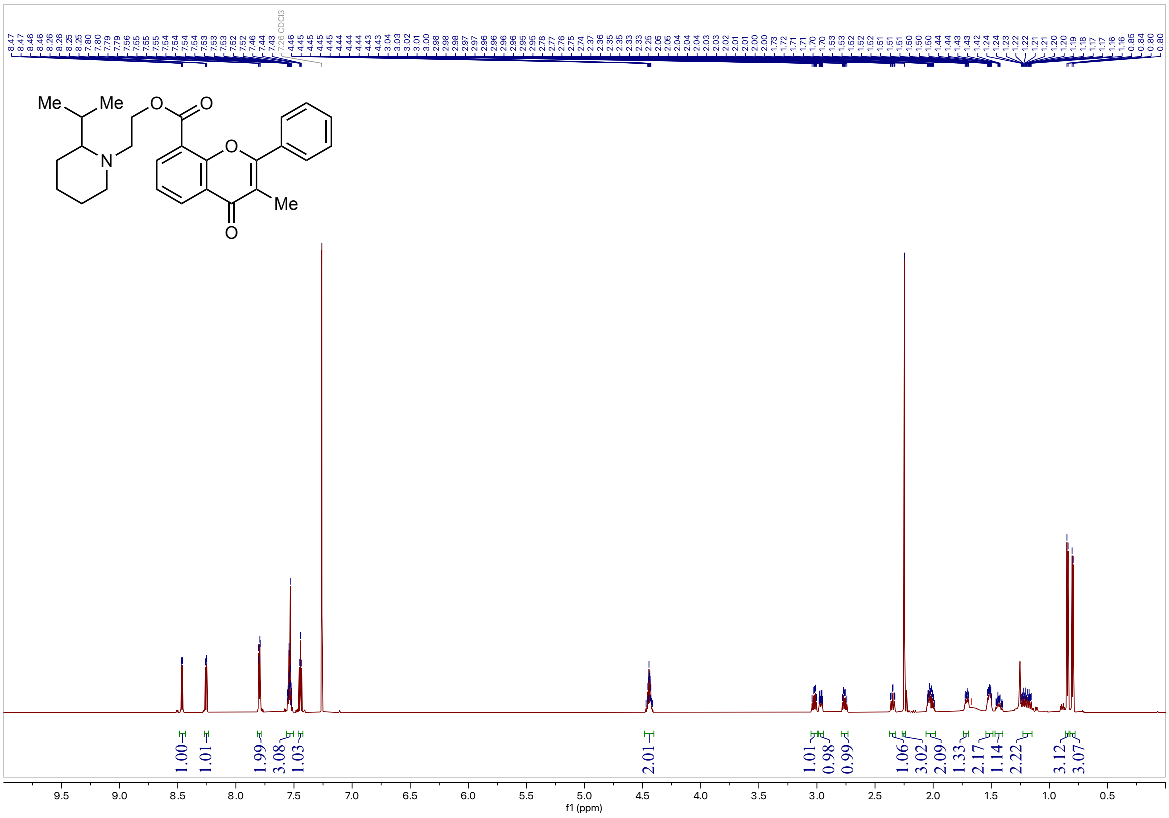

^{13}C{H} NMR (176 MHz, $CDCl_3$) of 2-(2-isopropylpiperidin-1-yl)ethyl 3-methyl-4-oxo-2-phenyl-4*H*-chromene-8-carboxylate (**27d**):

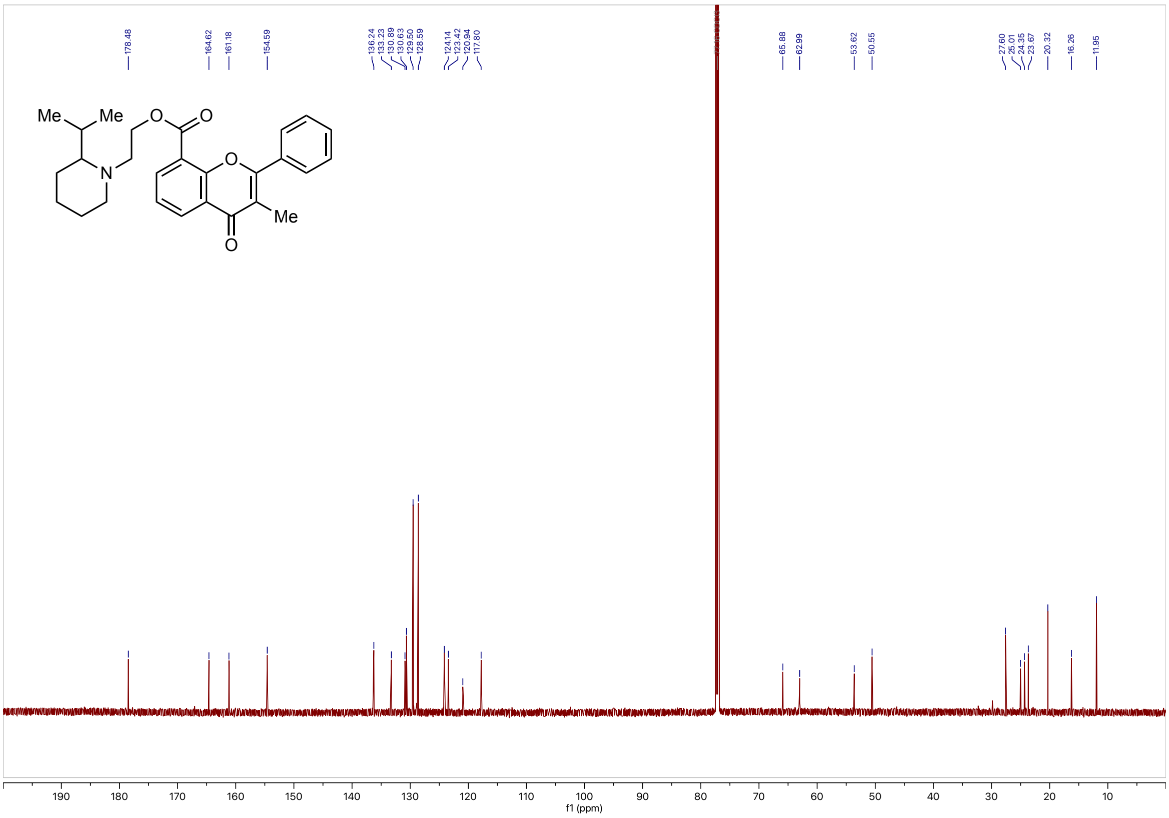

^{1}H NMR (700 MHz, $CDCl_3$) of 2-(2-ethylpiperidin-1-yl)ethyl 3-methyl-4-oxo-2-phenyl-4*H*-chromene-8-carboxylate (**27e**):

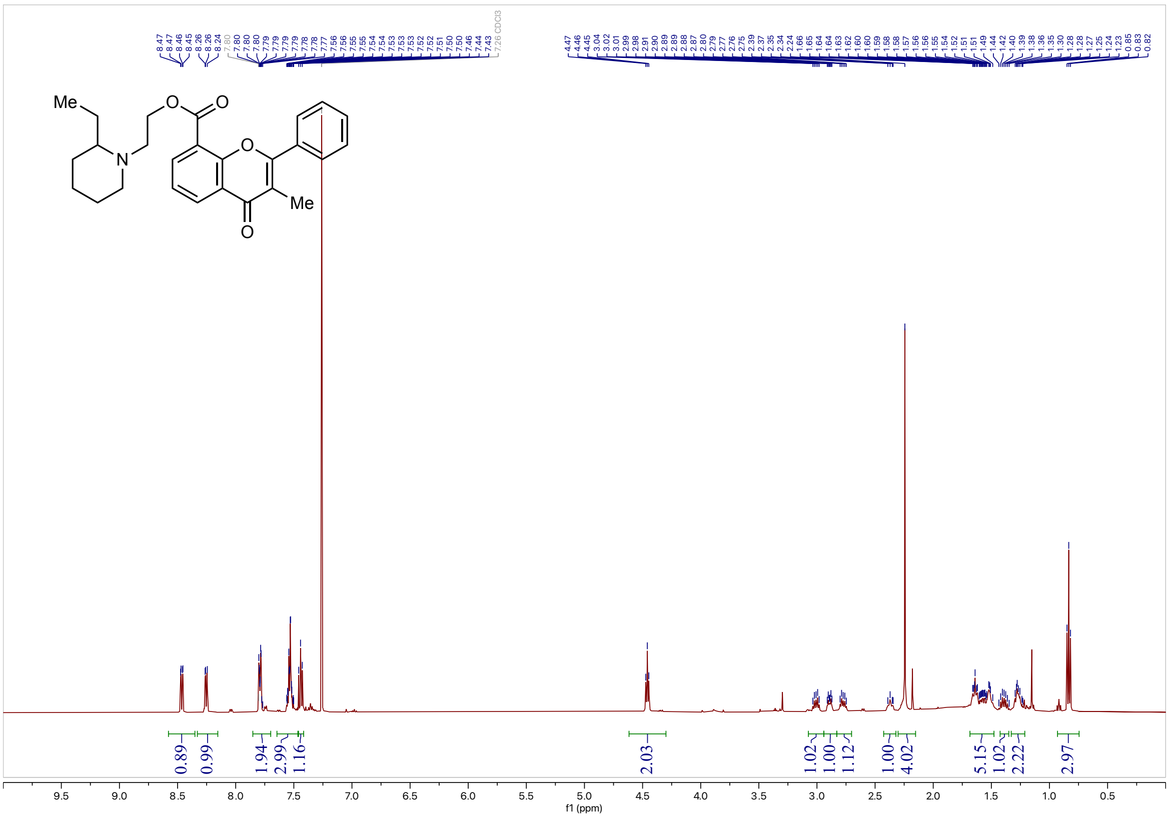

^{13}C{H} NMR (176 MHz, $CDCl_3$) of 2-(2-ethylpiperidin-1-yl)ethyl 3-methyl-4-oxo-2-phenyl-4*H*-chromene-8-carboxylate (**27e**):

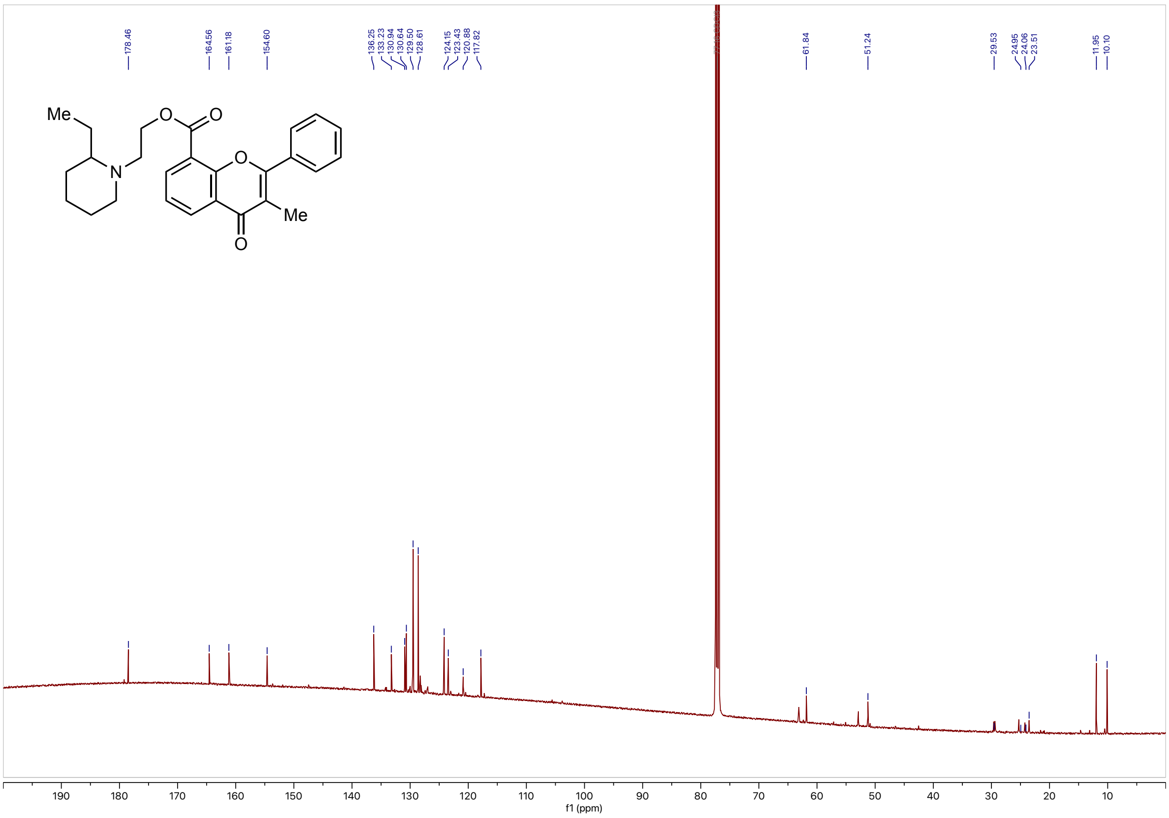

^{1}H NMR (700 MHz, $CDCl_3$) of 2-(2-(pyridin-2-yl)piperidin-1-yl)ethyl 3-methyl-4-oxo-2-phenyl-4*H*-chromene-8-carboxylate (**27f**):

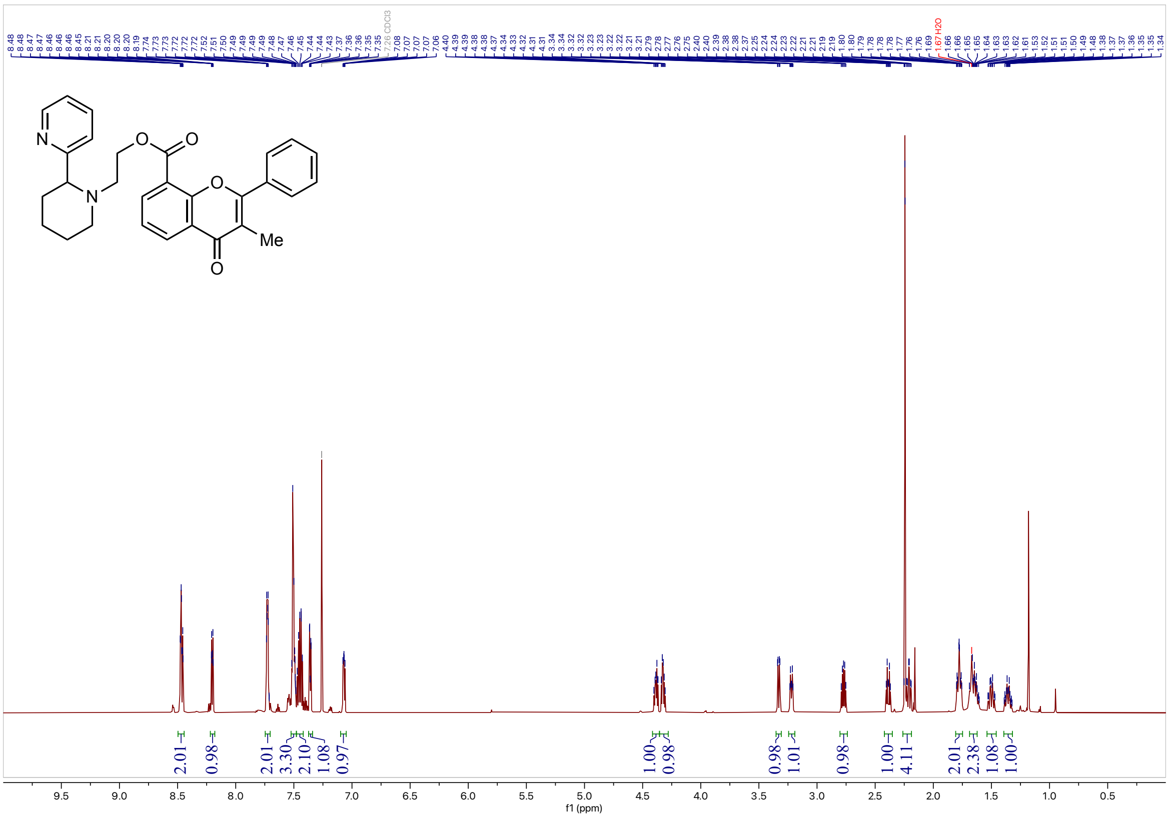

^{13}C{H} NMR (176 MHz, $CDCl_3$) of 2-(2-(pyridin-2-yl)piperidin-1-yl)ethyl 3-methyl-4-oxo-2-phenyl-4*H*-chromene-8-carboxylate (**27f**):

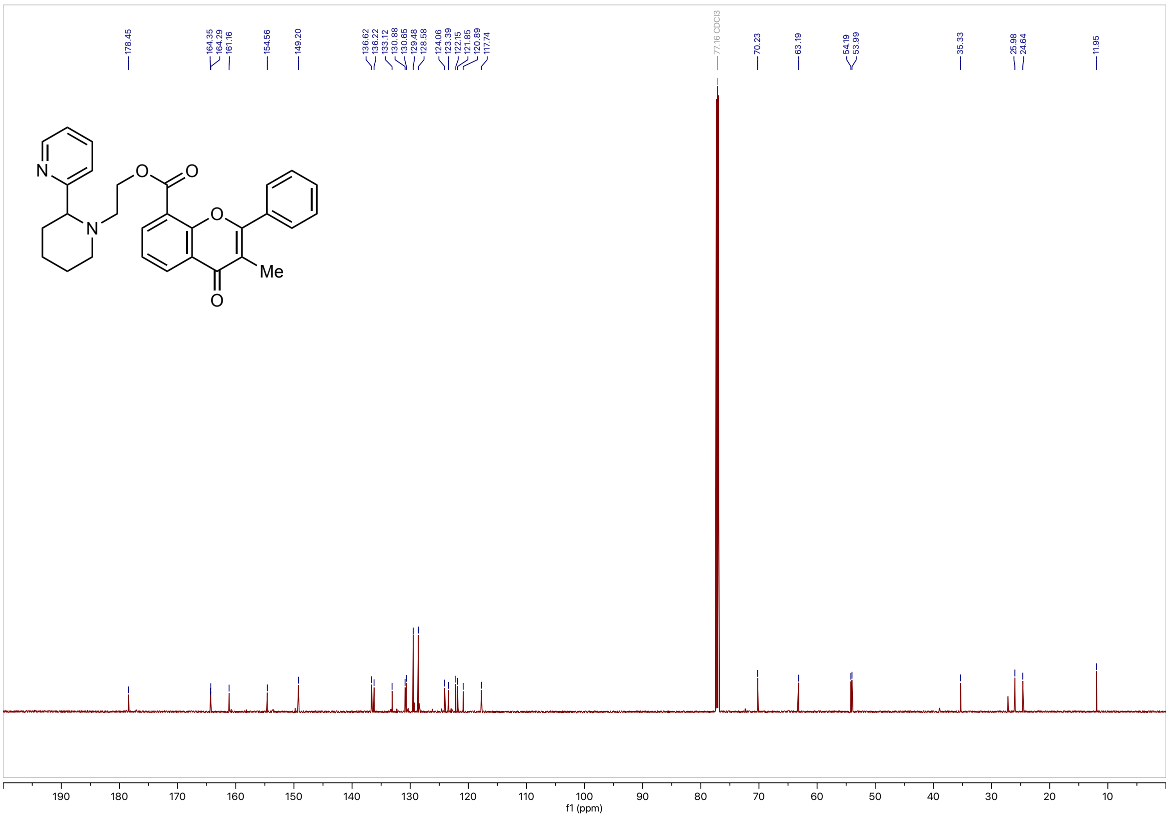

^{1}H NMR (500 MHz, $CDCl_3$) of *(2S,3S,4S)*-4-(5-chloro-2-methoxy-4-pivalamidobenzamido)-1-(3-(4-fluorophenoxy)propyl)-3-methoxy-2-(trifluoromethyl)piperidin-1-ium 2,2,2-trifluoroacetate (**28a**):

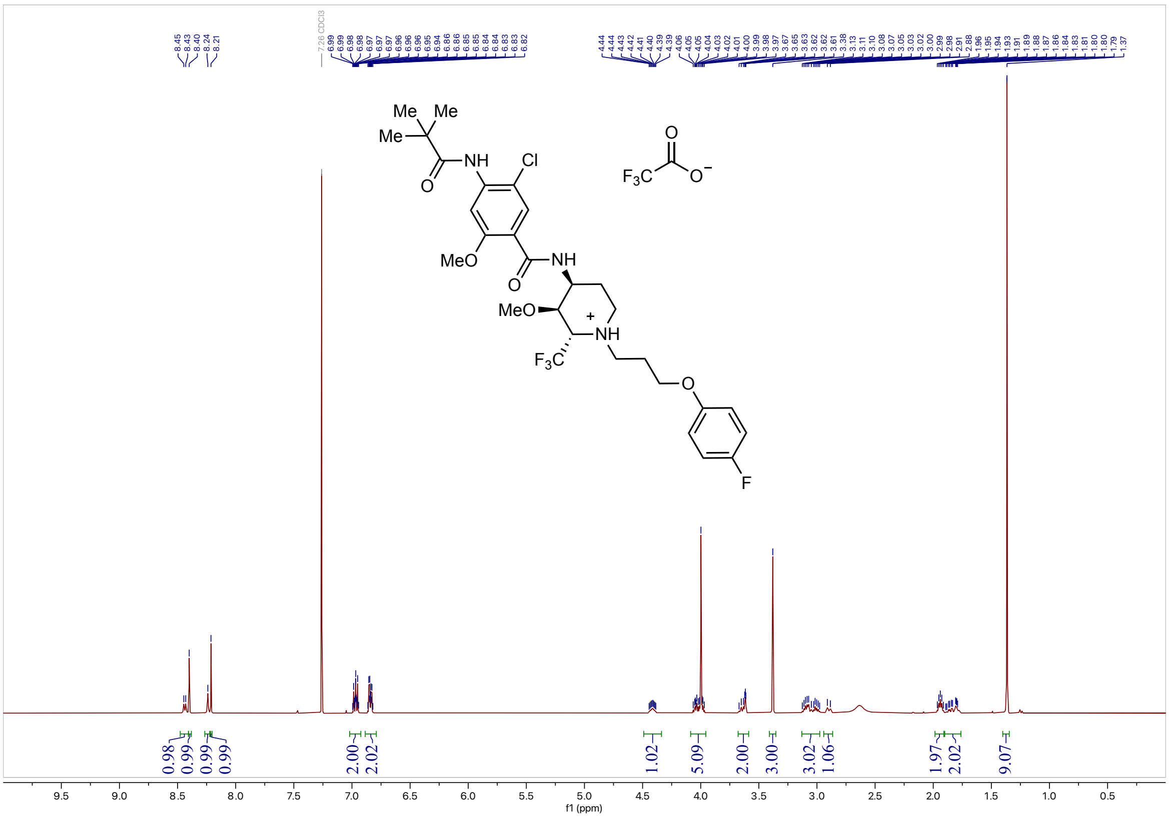

$^{13}C\{H\}$ NMR (126 MHz, $CDCl_3$) of *(2S,3S,4S)*-4-(5-chloro-2-methoxy-4-pivalamidobenzamido)-1-(3-(4-fluorophenoxy)propyl)-3-methoxy-2-(trifluoromethyl)piperidin-1-ium 2,2,2-trifluoroacetate (**28a**):

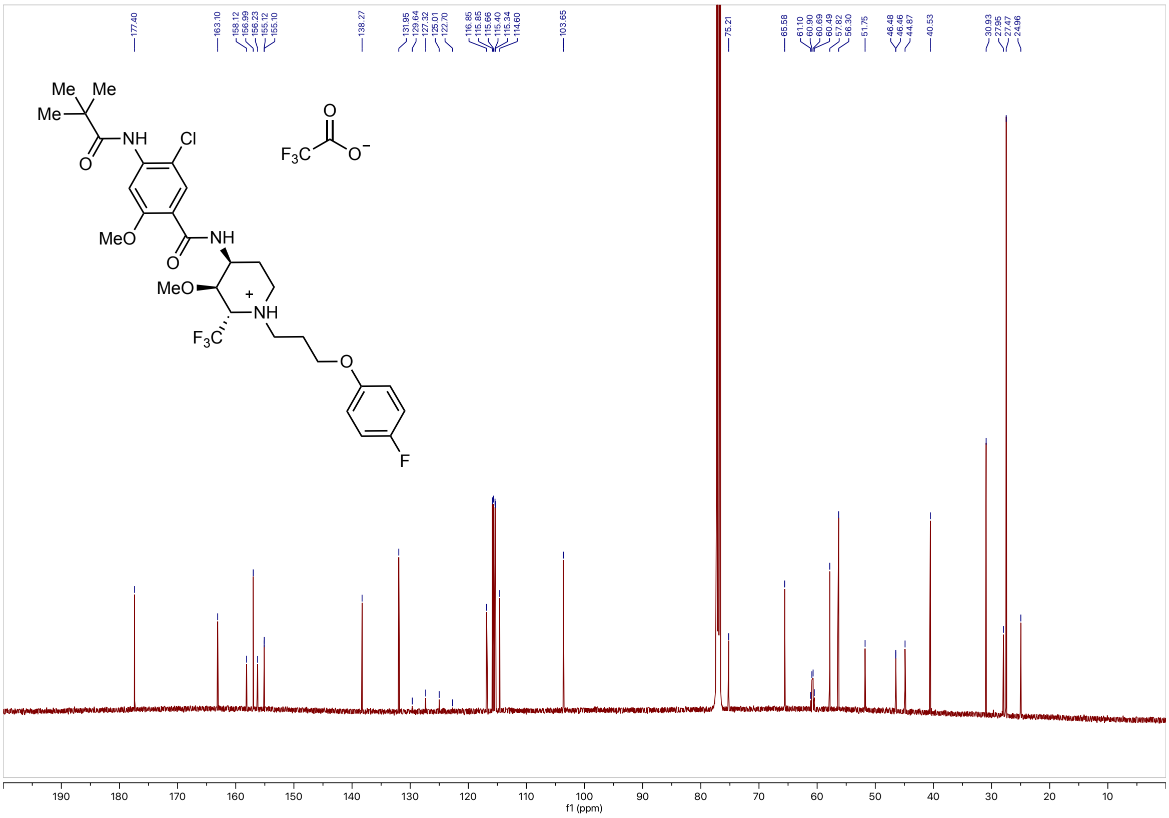

$^{19}F\{H\}$ NMR (376 MHz, $CDCl_3$) of *(2S,3S,4S)*-4-(5-chloro-2-methoxy-4-pivalamidobenzamido)-1-(3-(4-fluorophenoxy)propyl)-3-methoxy-2-(trifluoromethyl)piperidin-1-ium 2,2,2-trifluoroacetate (**28a**):

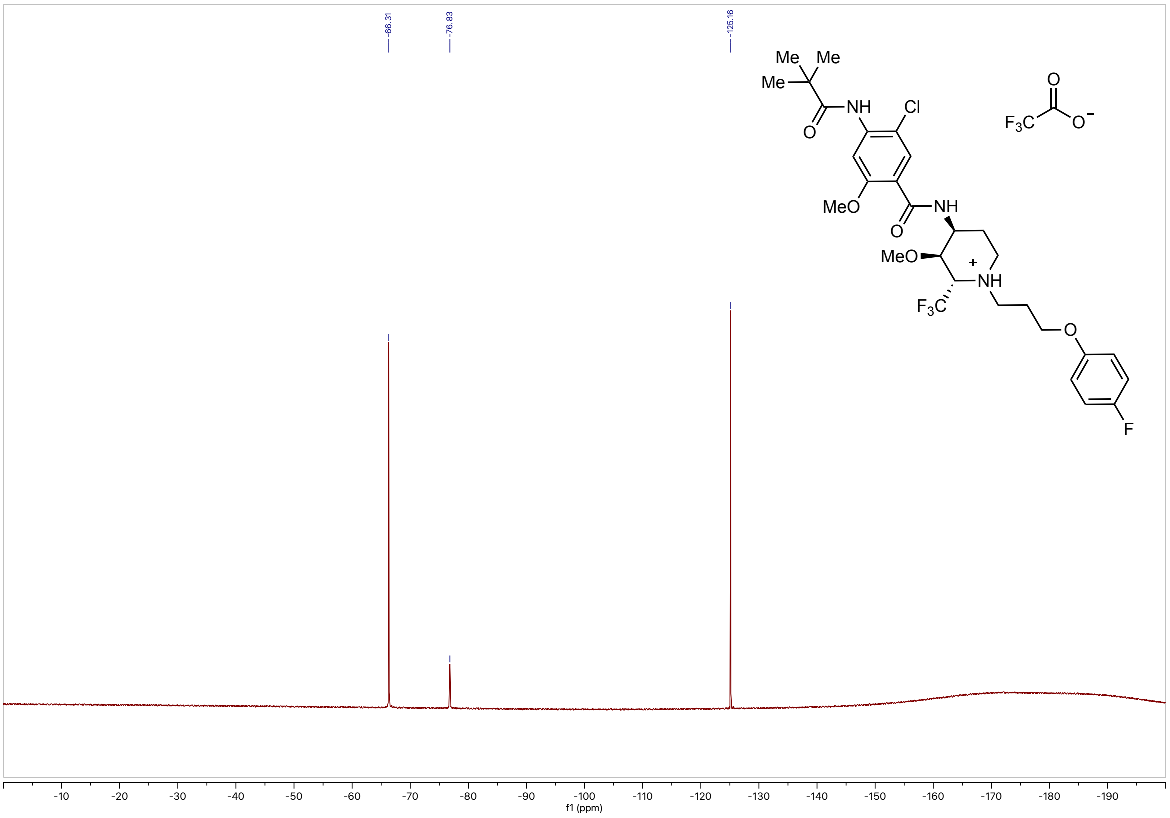

HMBC (500 MHz, $CDCl_3$) of (*2S,3S,4S*)-4-(5-chloro-2-methoxy-4-pivalamidobenzamido)-1-(3-(4- fluorophenoxy)propyl)-3-methoxy-2-(trifluoromethyl)piperidin-1-ium 2,2,2- trifluoroacetate (**28a**):

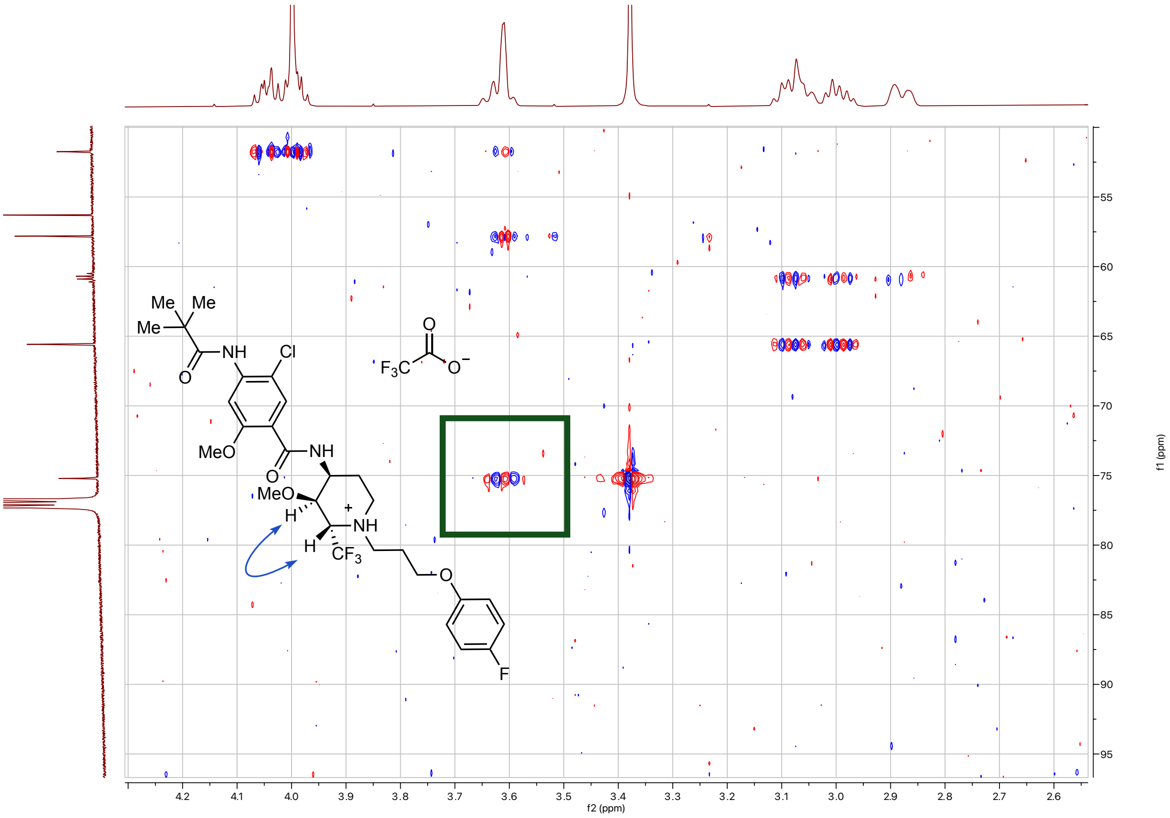

Heteronuclear NOSEY (500 MHz, $CDCl_3$) of (*2S,3S,4S*)-4-(5-chloro-2-methoxy-4-pivalamidobenzamido)-1-(3-(4- fluorophenoxy)propyl)-3-methoxy-2-(trifluoromethyl)piperidin-1-ium 2,2,2- trifluoroacetate (**28a**):

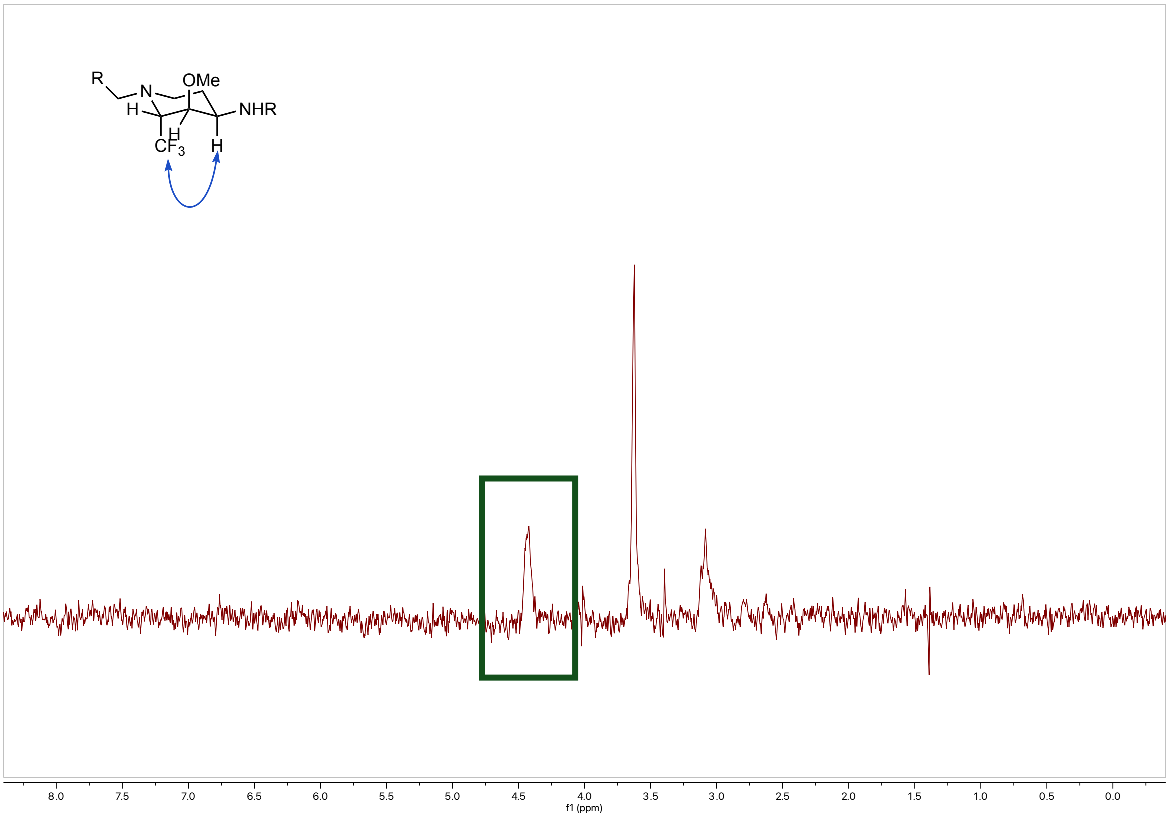

^{1}H NMR (500 MHz, $CDCl_3$) of (*2R,3S,4S*)-4-(5-chloro-2-methoxy-4-pivalamidobenzamido)-1-(3-(4-fluorophenoxy)propyl)-3-methoxy-2-(trifluoromethyl)piperidin-1-ium 2,2,2-trifluoroacetate (**28b**):

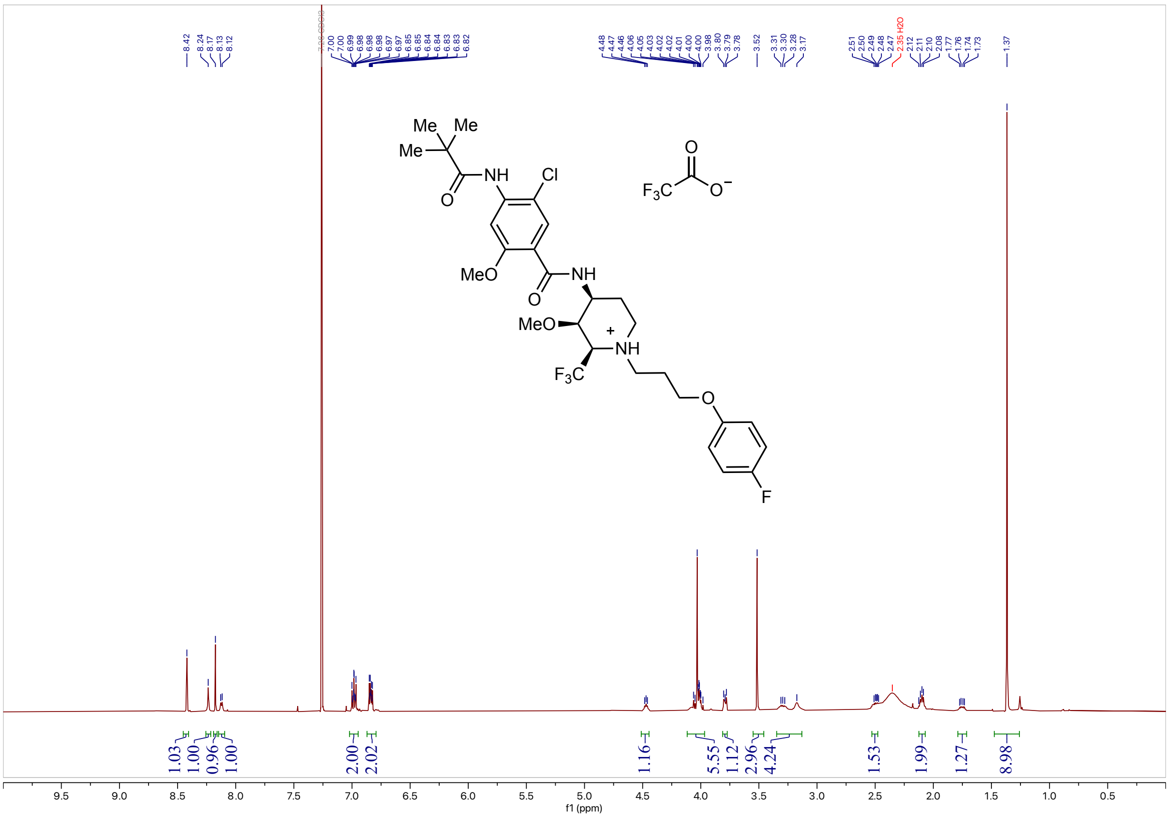

$^{13}C\{H\}$ NMR (126 MHz, $CDCl_3$) of (*2R,3S,4S*)-4-(5-chloro-2-methoxy-4-pivalamidobenzamido)-1-(3-(4-fluorophenoxy)propyl)-3-methoxy-2-(trifluoromethyl)piperidin-1-ium
2,2,2-trifluoroacetate (**28b**):

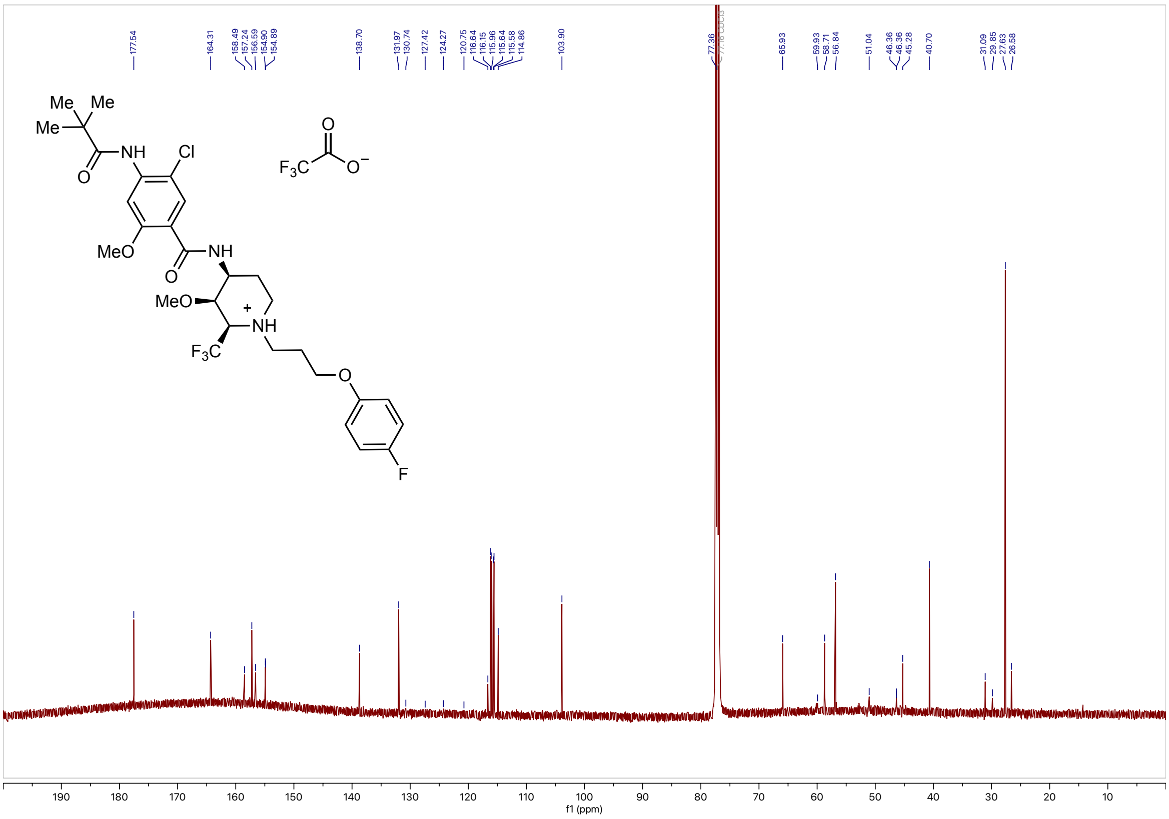

^{19}F{H} NMR (376 MHz, $CDCl_3$) of (*2R,3S,4S*)-4-(5-chloro-2-methoxy-4-pivalamidobenzamido)-1-(3-(4-fluorophenoxy)propyl)-3-methoxy-2-(trifluoromethyl)piperidin-1-ium
2,2,2-trifluoroacetate (**28b**):

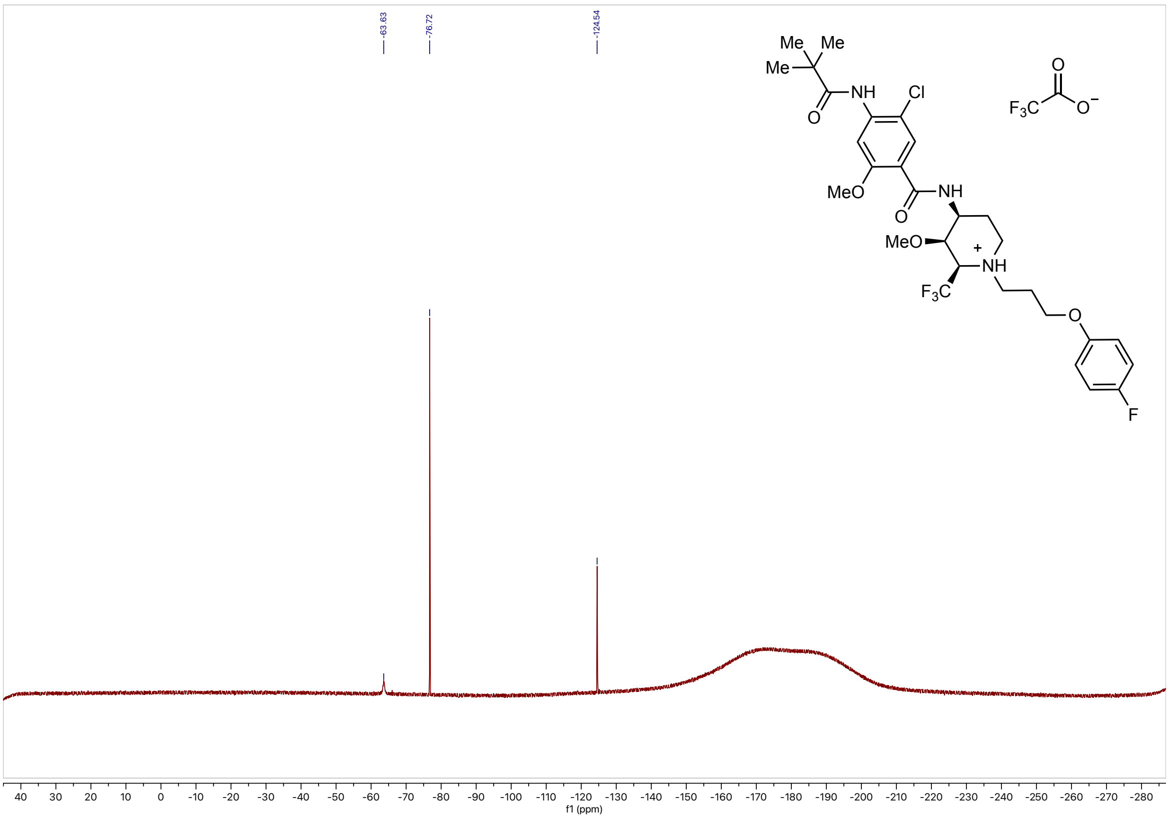

COSY (500 MHz, $CDCl_3$) of (*2R,3S,4S*)-4-(5-chloro-2-methoxy-4-pivalamidobenzamido)-1-(3-(4- fluorophenoxy)propyl)-3-methoxy-2-(trifluoromethyl)piperidin-1-ium 2,2,2-trifluoroacetate (**28b**):

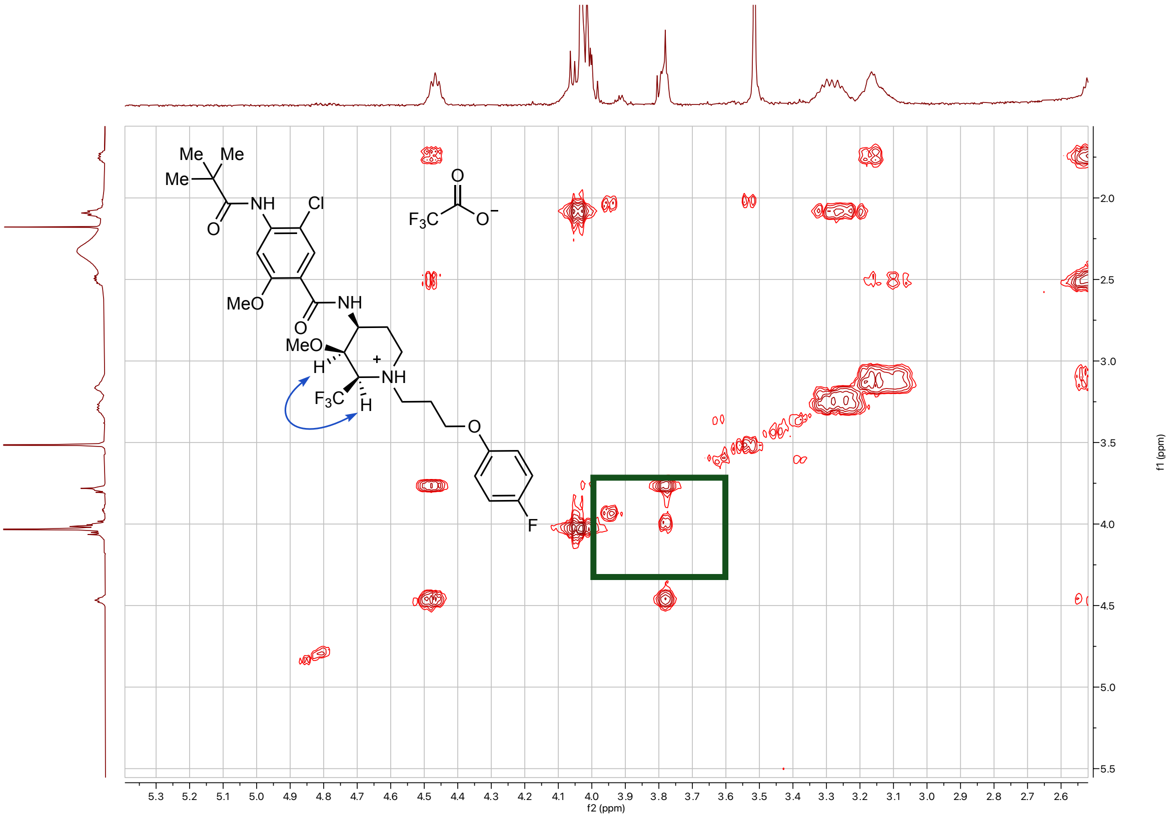

NOSEY (500 MHz, $CDCl_3$) of (*2R,3S,4S*)-4-(5-chloro-2-methoxy-4-pivalamidobenz amido)-1-(3-(4- fluorophenoxy)propyl)-3-methoxy-2-(trifluoromethyl)piperidin-1-ium 2,2,2-trifluoroacetate (**28b**):

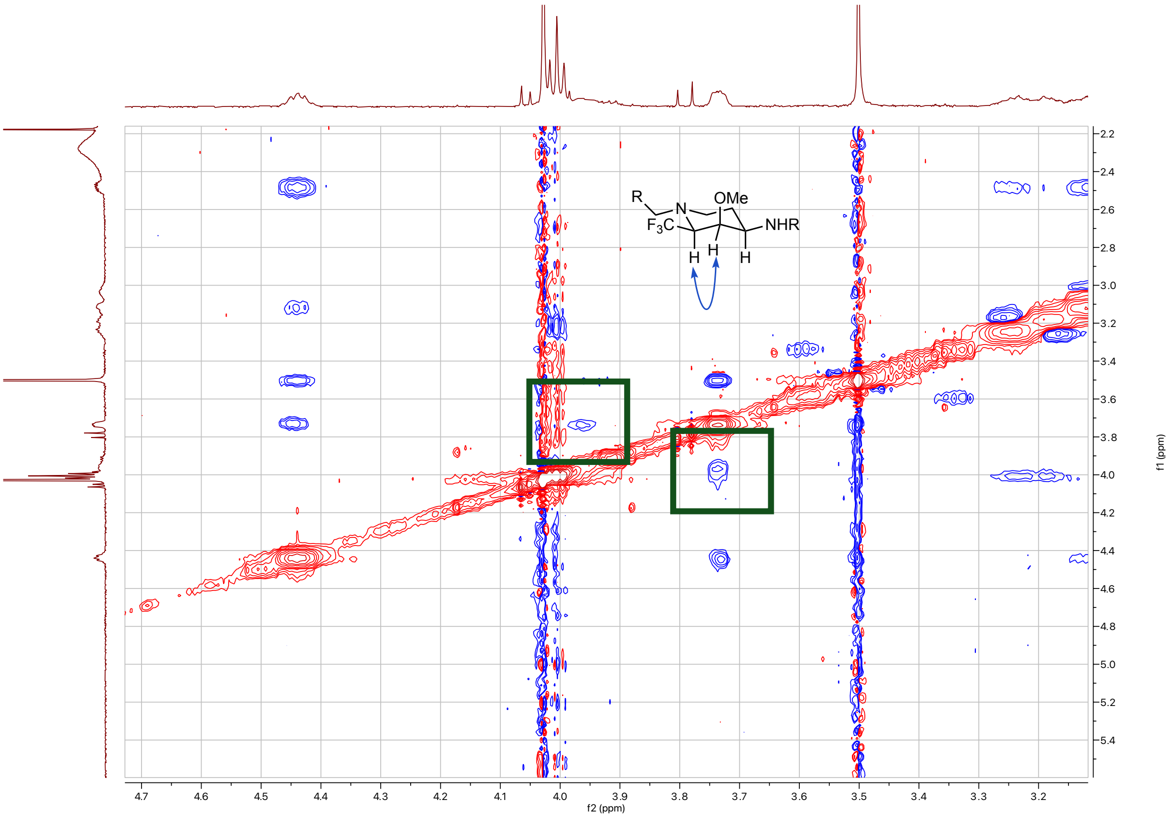

^{1}H NMR (500 MHz, $CDCl_3$) of (*2R,3R,4S*)-4-(5-chloro-2-methoxy-4-pivalamidobenzamido)-1-(3-(4-fluorophenoxy)propyl)-3-methoxy-2-(trifluoromethyl)piperidin-1-ium 2,2,2-trifluoroacetate (**28c**):

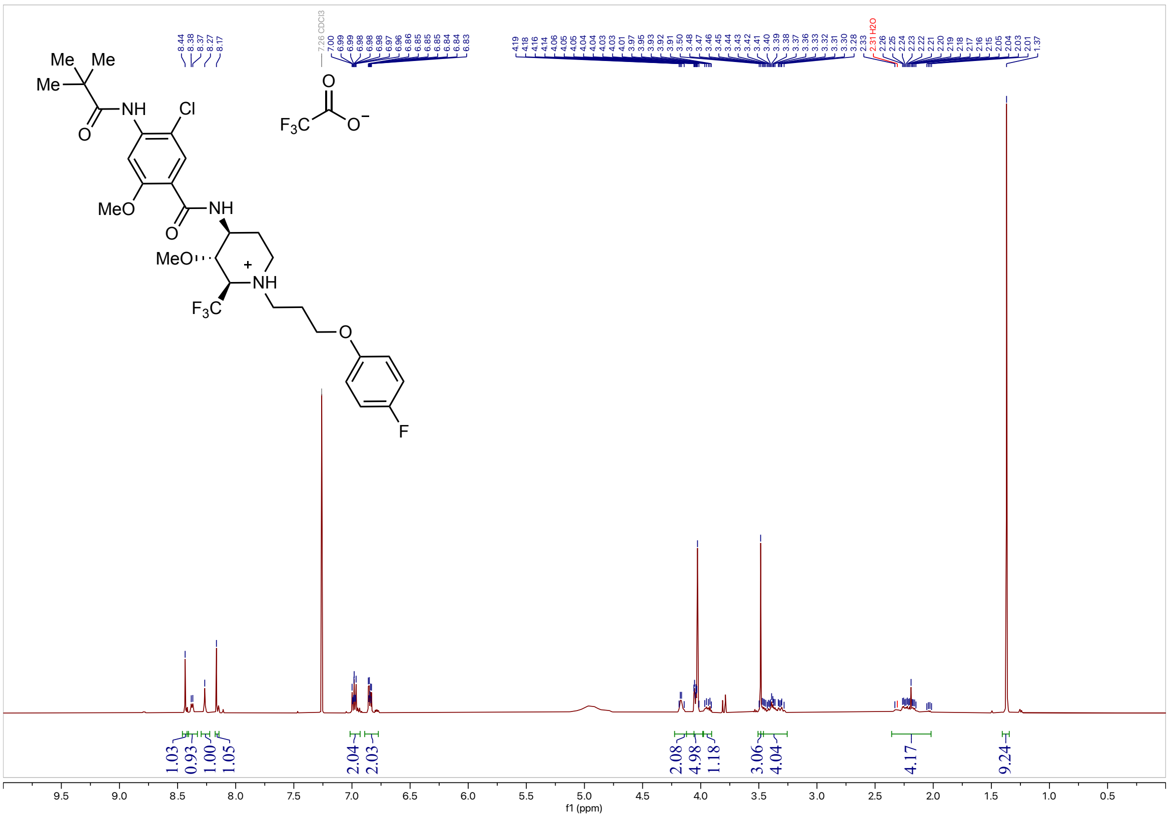

^{13}C{H} NMR (126 MHz, CDCl$_3$) of (*2R,3R,4S*)-4-(5-chloro-2-methoxy-4-pivalamidobenzamido)-1-(3-(4-fluorophenoxy)propyl)-3-methoxy-2-(trifluoromethyl)piperidin-1-ium 2,2,2-trifluoroacetate (**28c**):

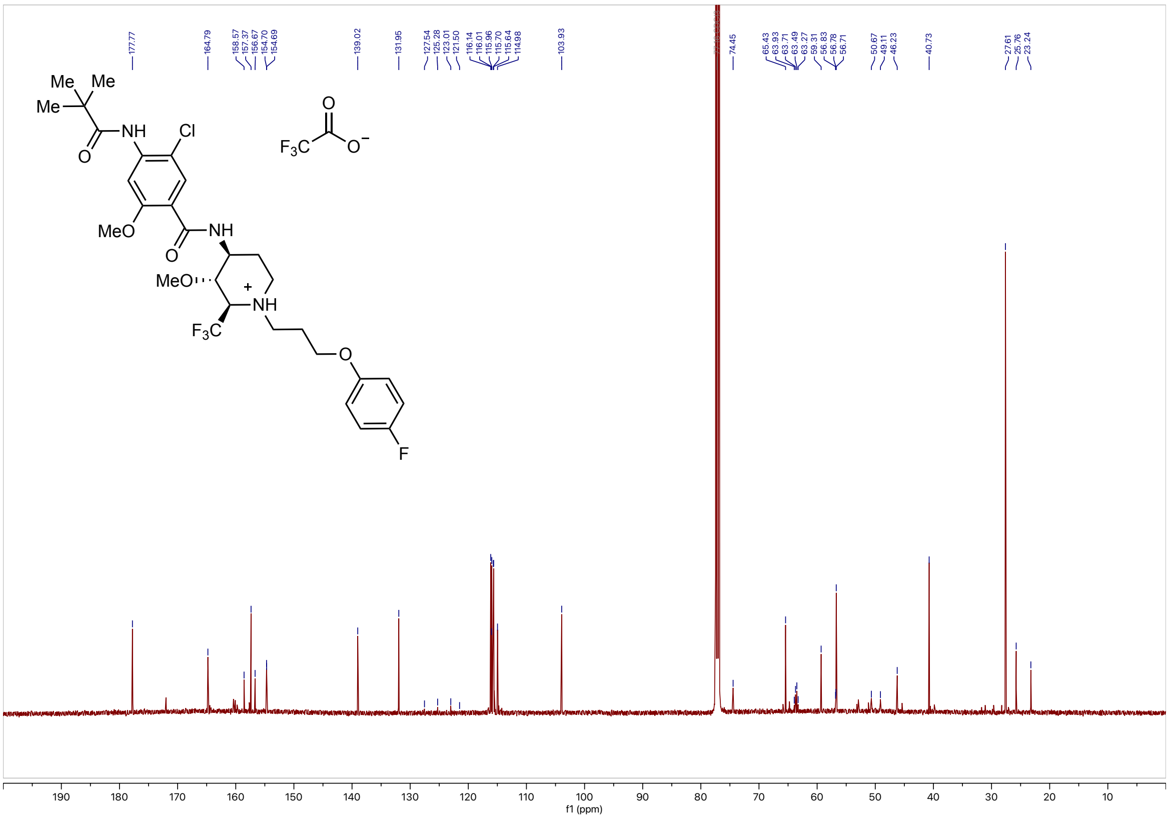

^{19}F{H} NMR (376 MHz, CDCl$_3$) of (*2R,3R,4S*)-4-(5-chloro-2-methoxy-4-pivalamidobenzamido)-1-(3-(4-fluorophenoxy)propyl)-3-methoxy-2-(trifluoromethyl)piperidin-1-ium 2,2,2-trifluoroacetate (**28c**):

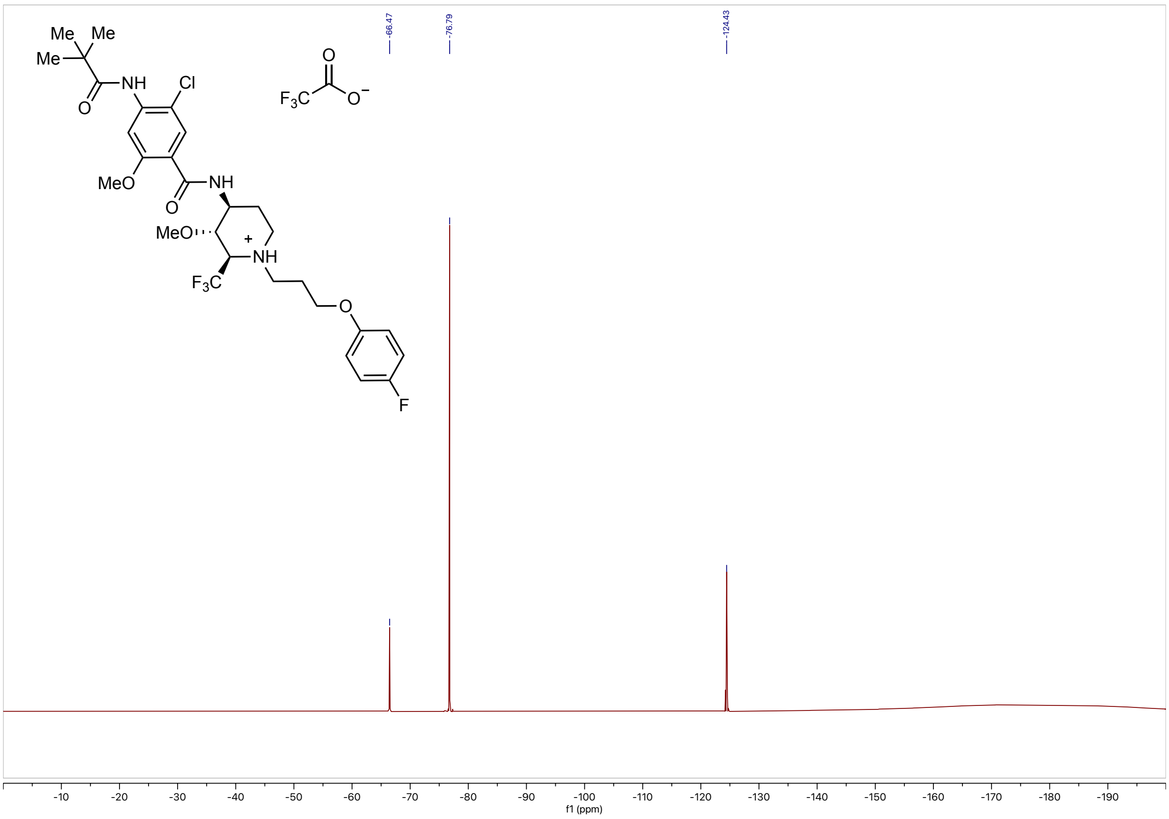

COSY (500 MHz, $CDCl_3$) of (*2R,3R,4S*)-4-(5-chloro-2-methoxy-4-pivalamidobenzamido)-1-(3-(4- fluorophenoxy)propyl)-3-methoxy-2-(trifluoromethyl)piperidin-1-ium 2,2,2-trifluoroacetate (**28c**):

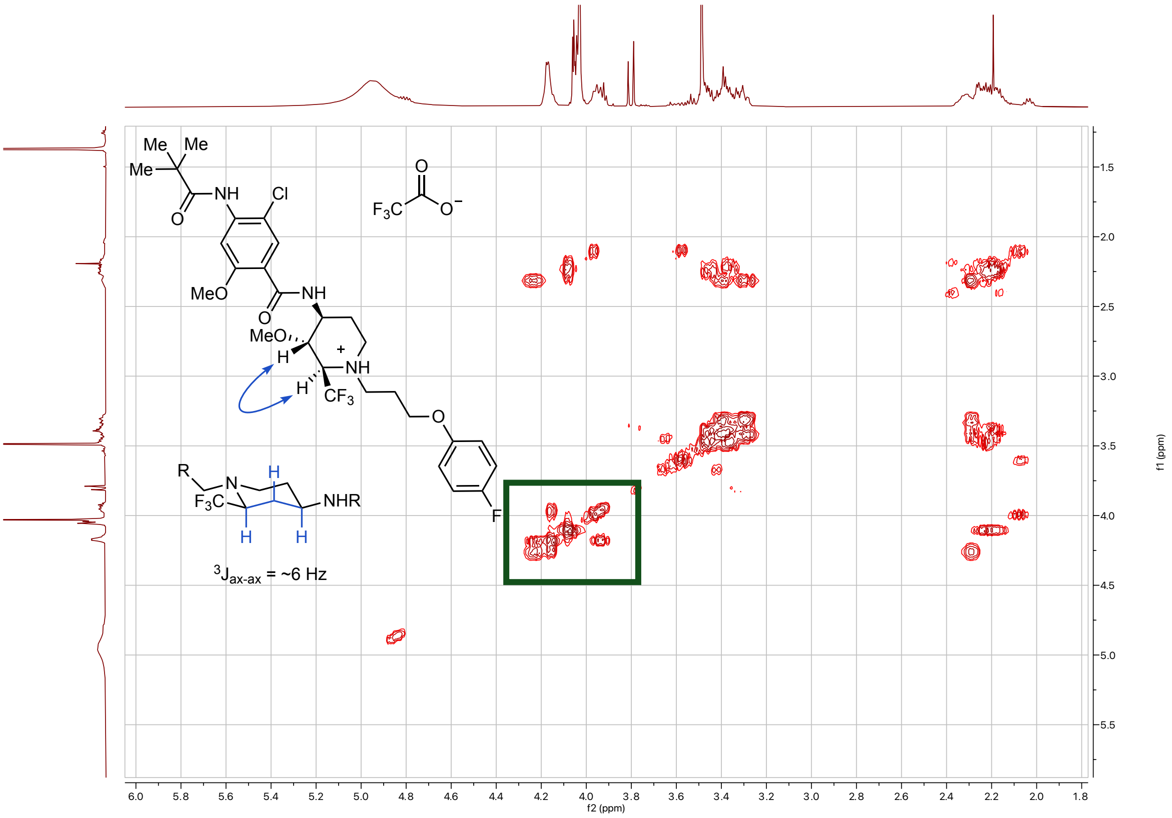

^{1}H NMR (500 MHz, $CDCl_3$) of 1-benzyl-3-methylpiperidine (**1g**):

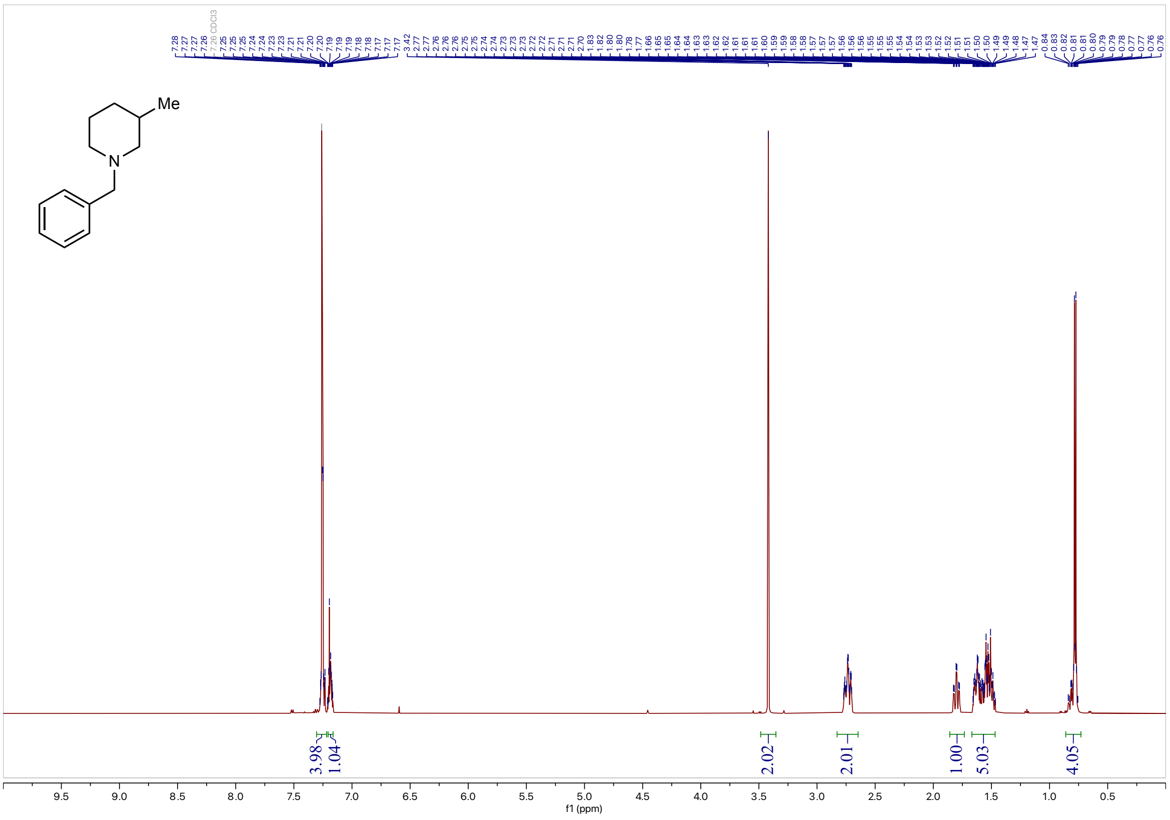

^{13}C{H} NMR (126 MHz, $CDCl_3$) of 1-benzyl-3-methylpiperidine (**1g**):

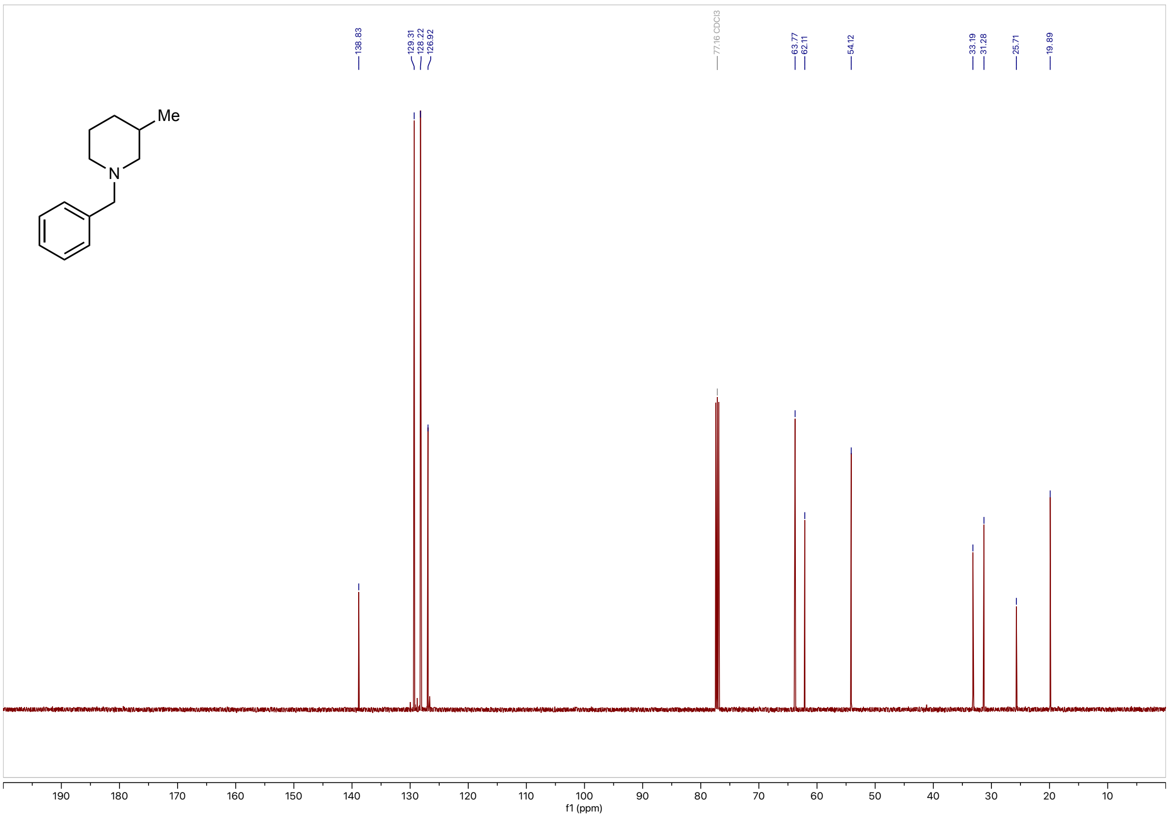

^{1}H NMR (400 MHz, $CDCl_3$) of 1-(4-phenylbenzyl)piperidine (**1r**):

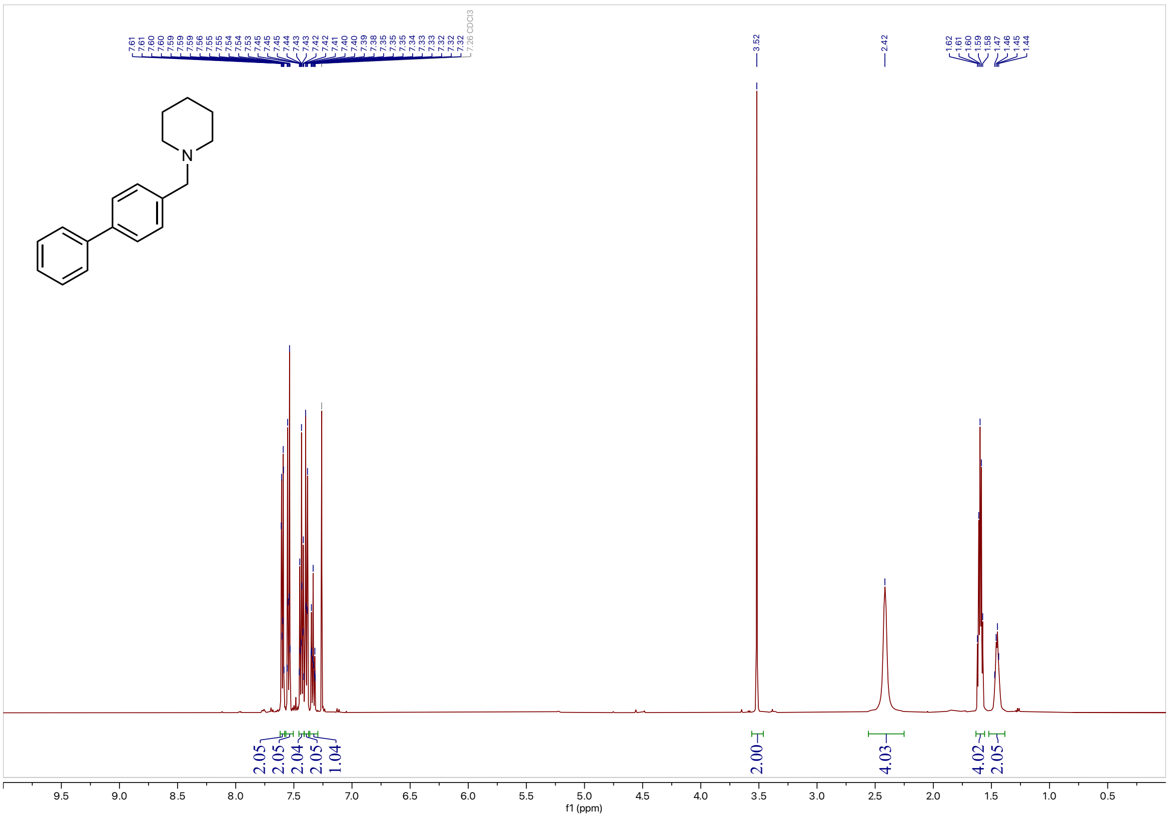

$^{13}C\{H\}$ NMR (126 MHz, $CDCl_3$) of 1-(4-phenylbenzyl)piperidine (**1r**):

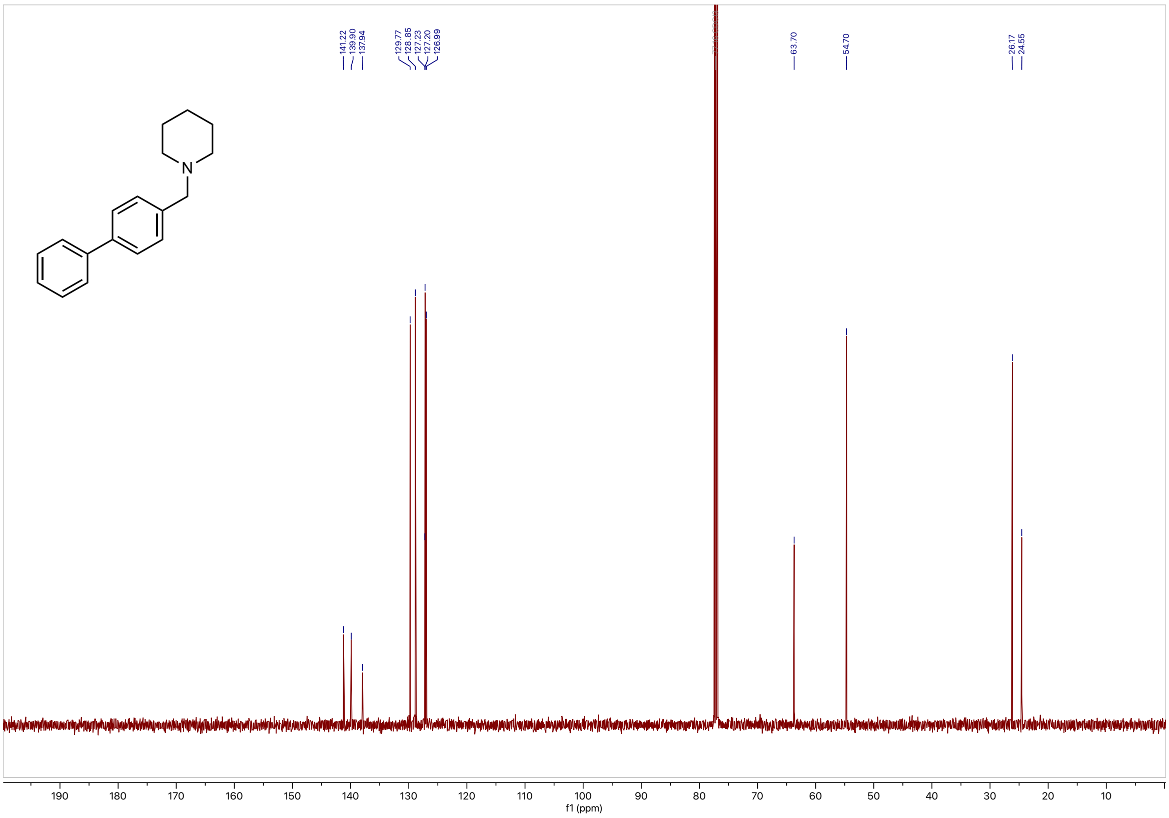

^{1}H NMR (700 MHz, $CDCl_3$) of dextromethorphan (**29**):

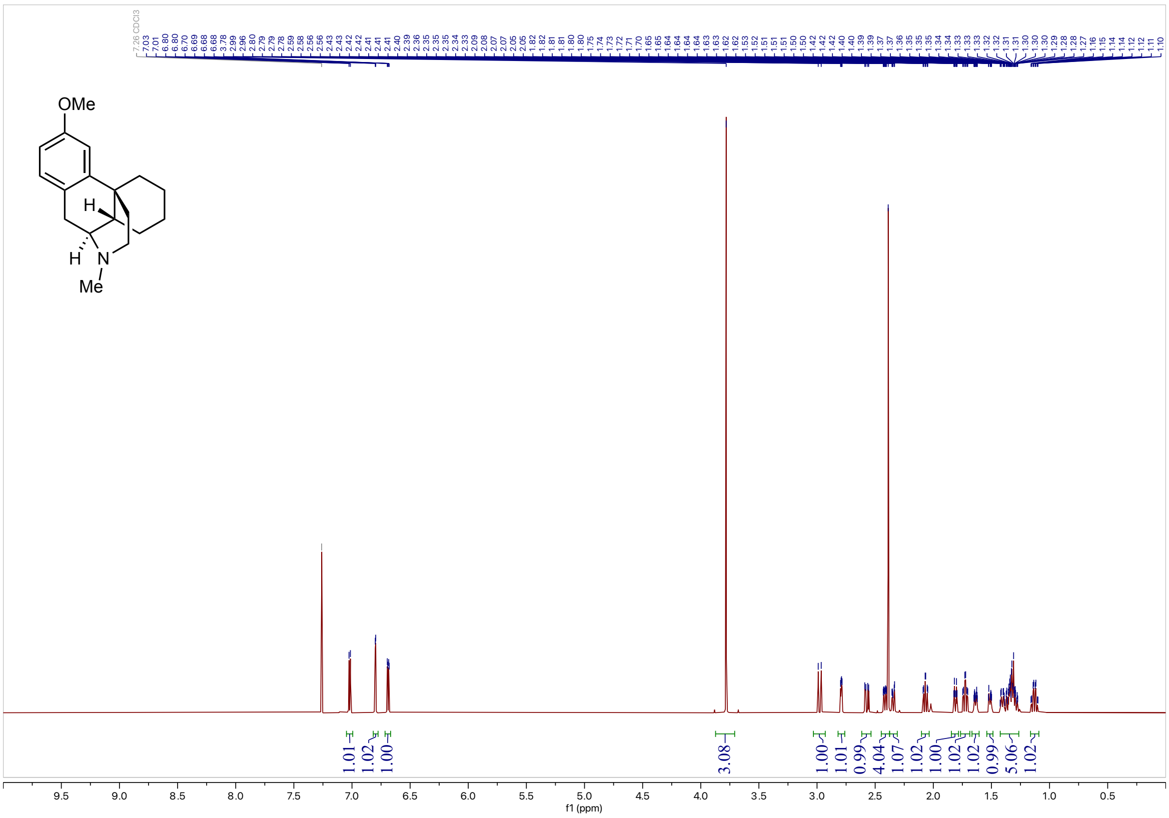

$^{13}C\{H\}$ NMR (176 MHz, $CDCl_3$) of dextromethorphan (**29**):

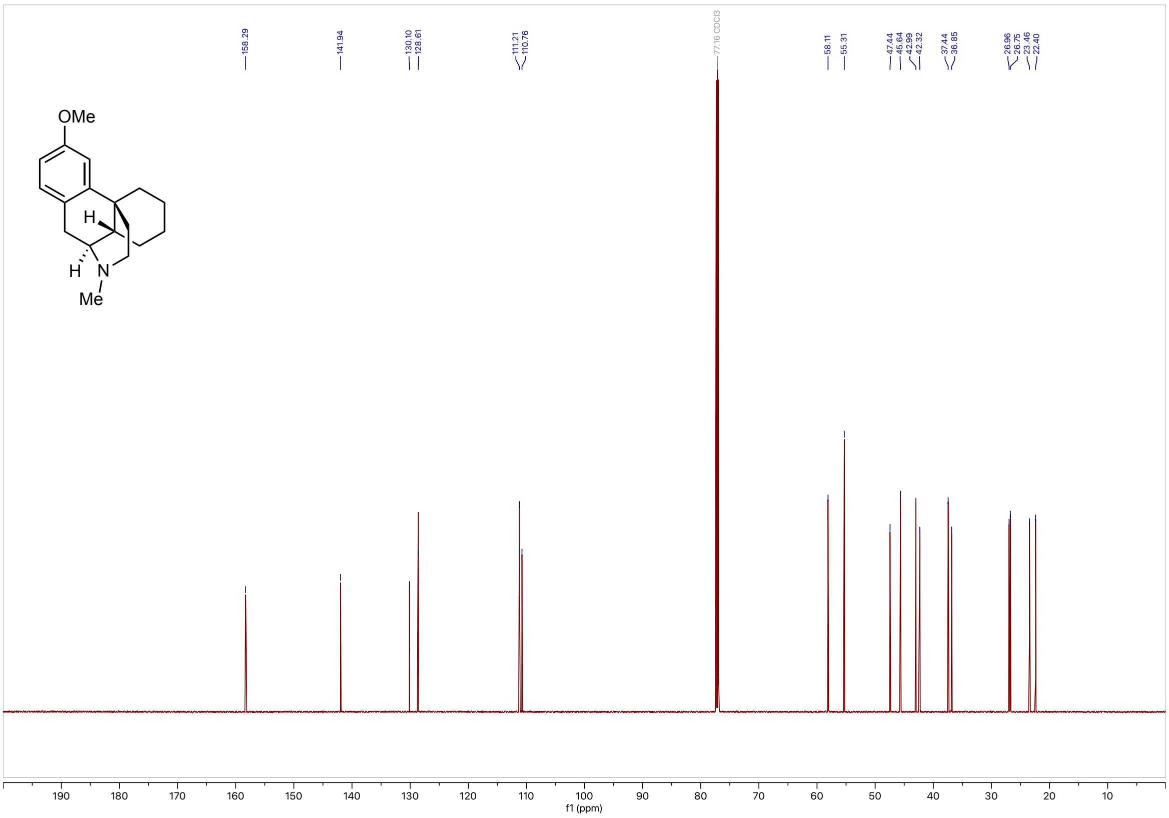

^{1}H NMR (500 MHz, $CDCl_3$) of cloperastine (**30**):

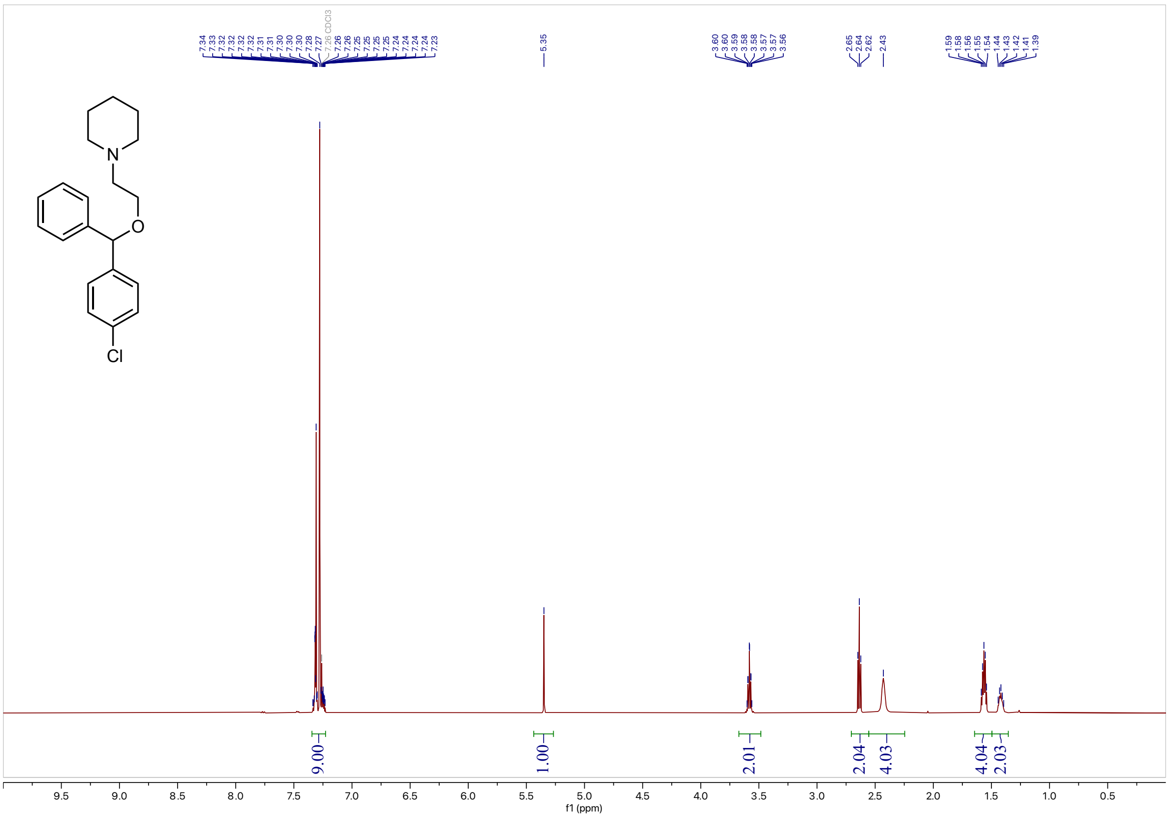

^{13}C{H} NMR (126 MHz, $CDCl_3$) of cloperastine (**30**):

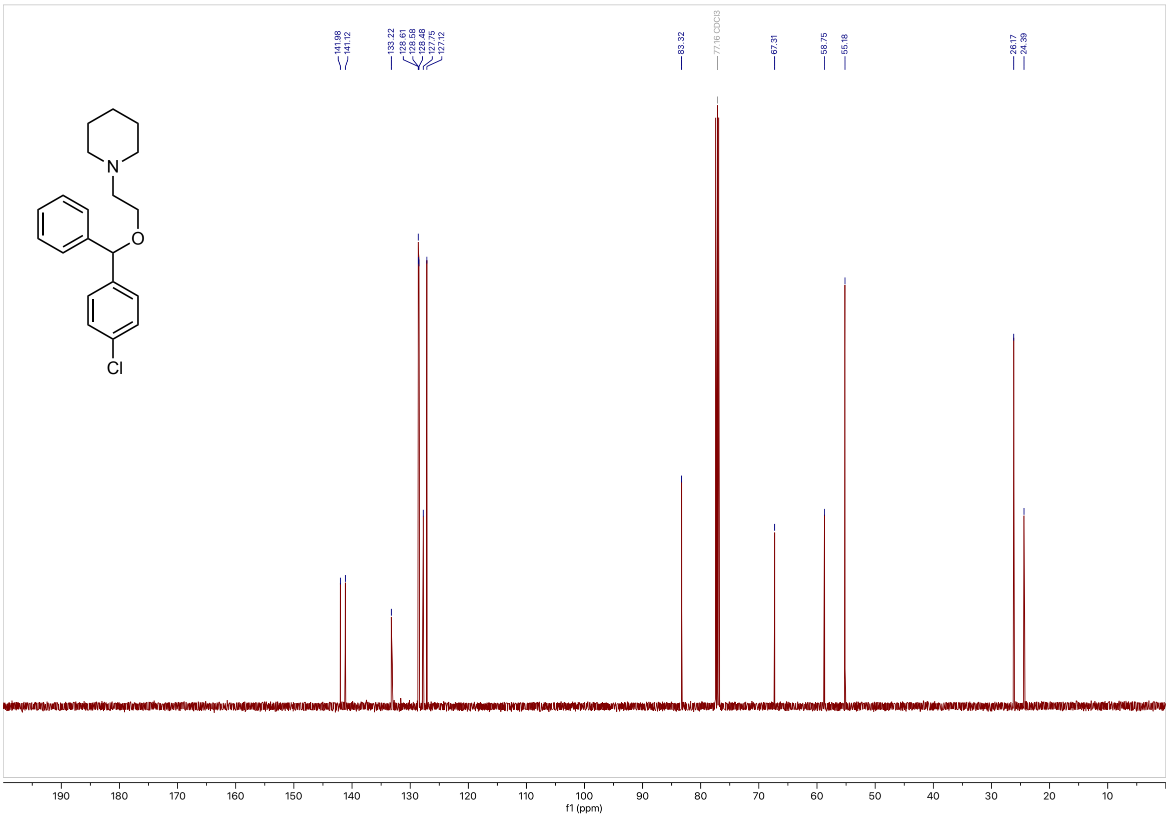

^{1}H NMR (700 MHz, $CDCl_3$) of ticlopidine (**31**):

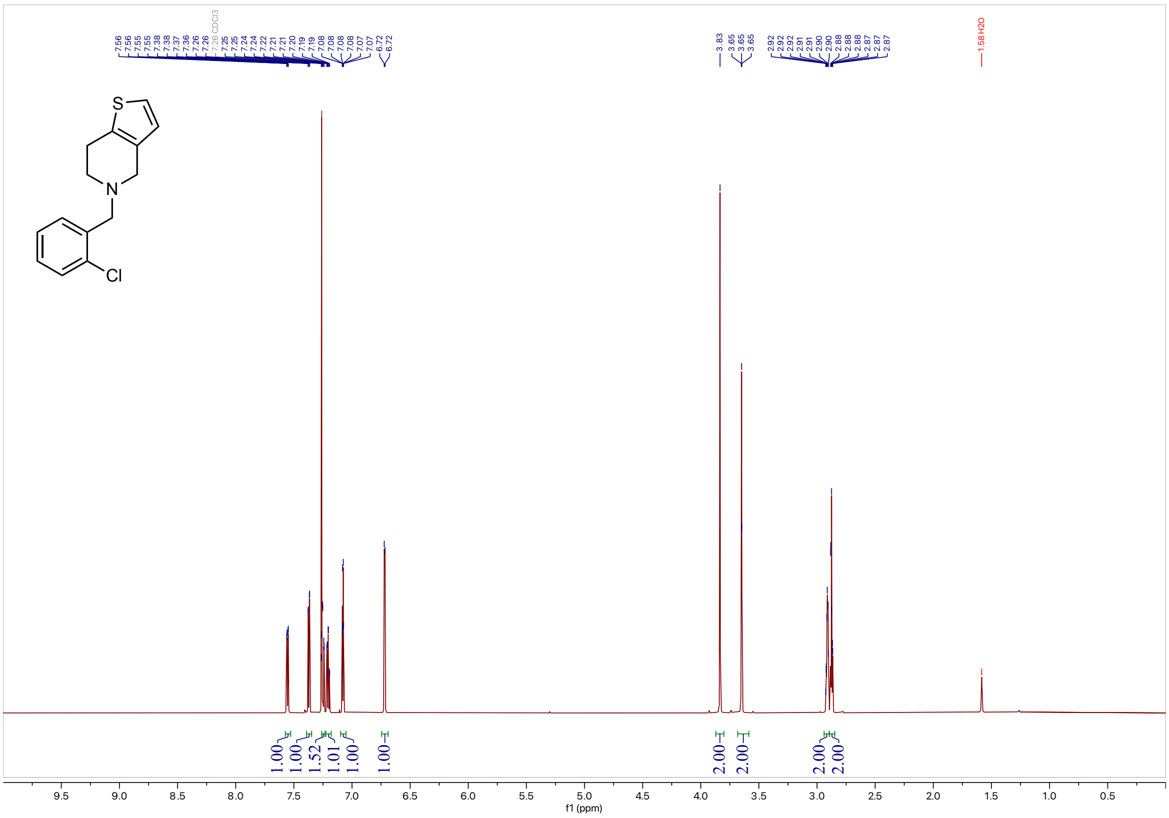

^{13}C{H} NMR (176 MHz, $CDCl_3$) of ticlopidine (**31**):

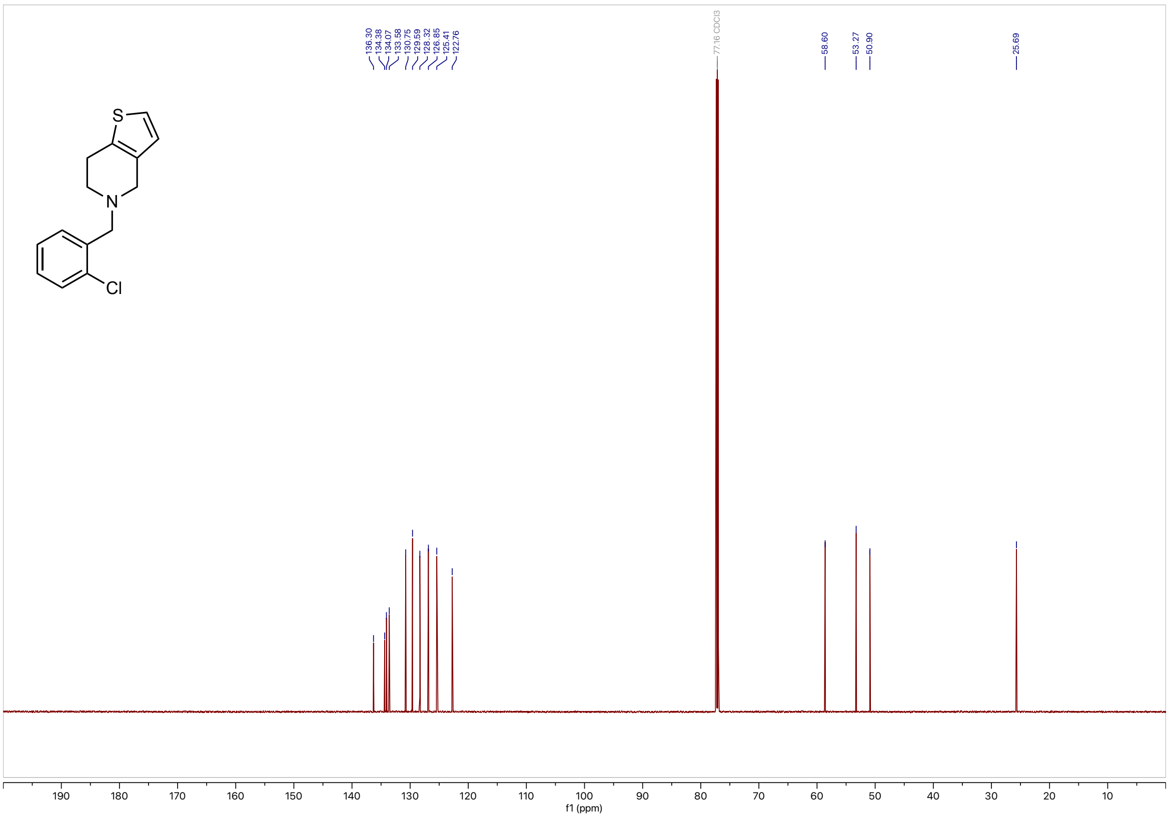

^{1}H NMR (400 MHz, $CDCl_3$) of *1*-benzylpiperidine *N*-oxide (**2a**):

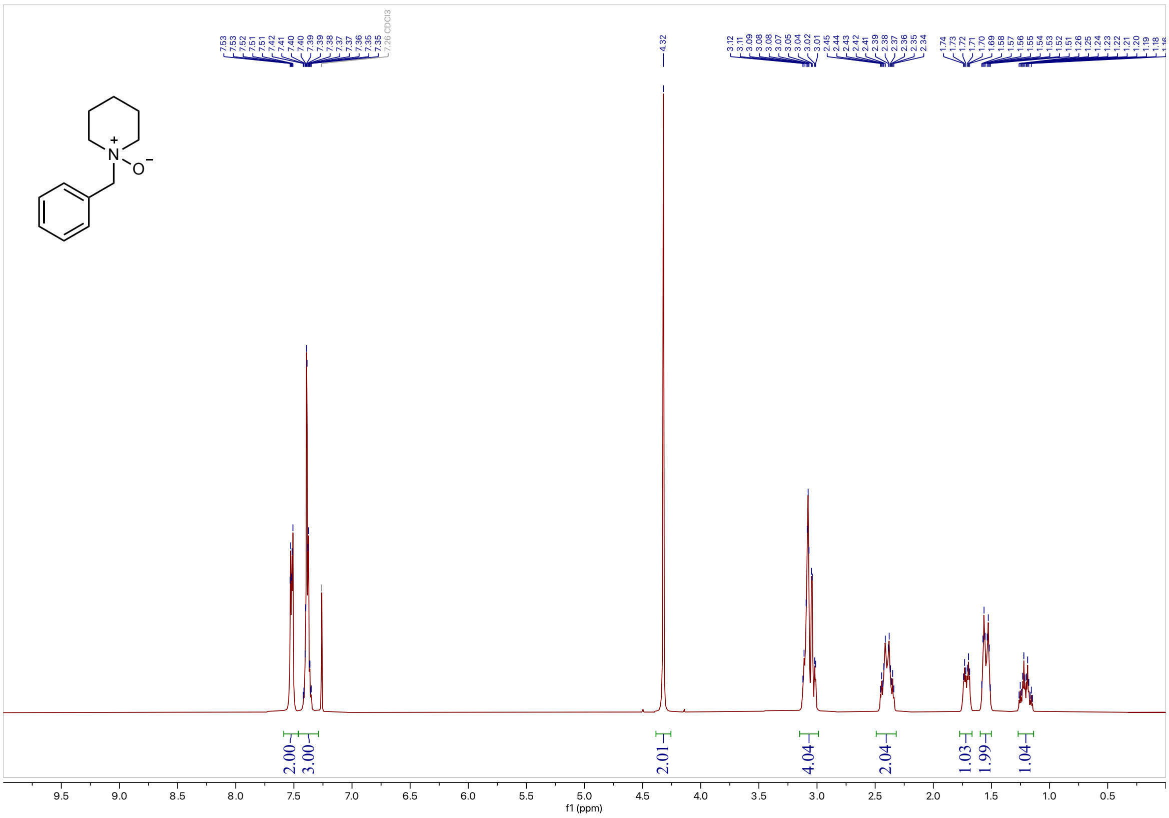

$^{13}C\{H\}$ NMR (101 MHz, $CDCl_3$) of *1*-benzylpiperidine *N*-oxide (**2a**):

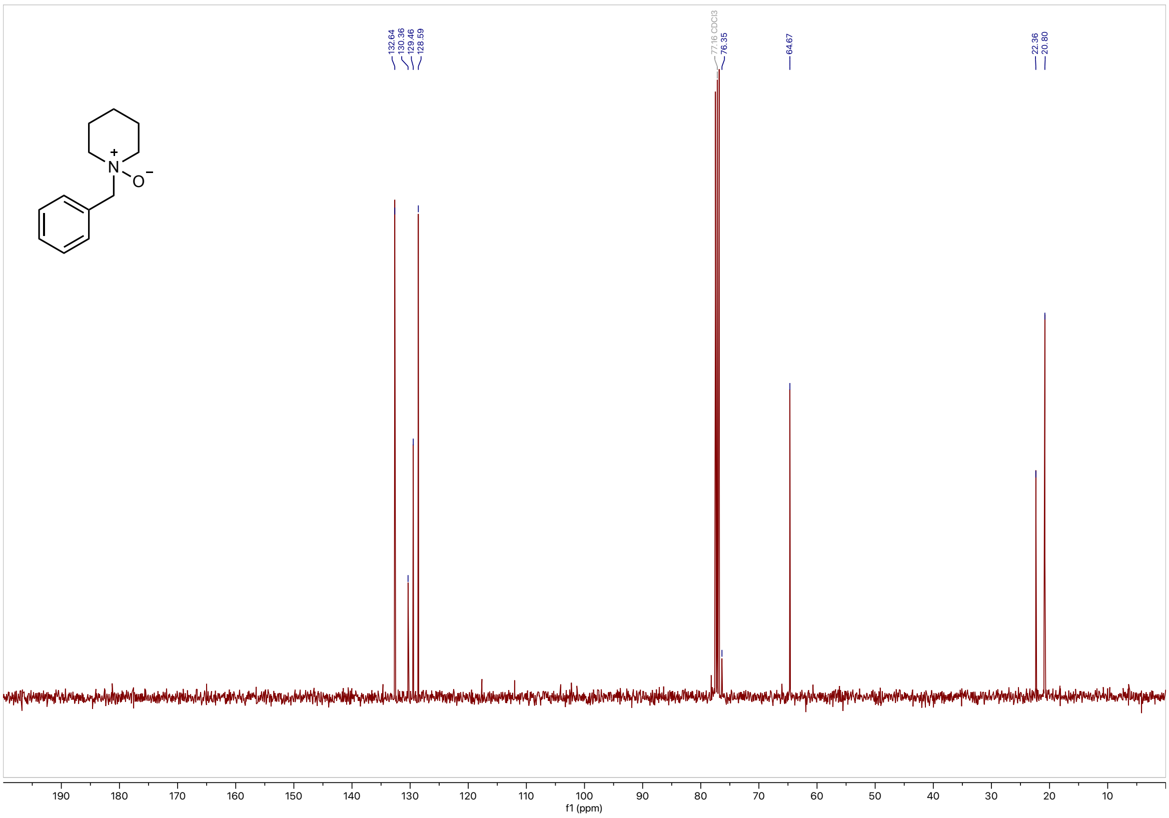

^{1}H NMR (400 MHz, $CDCl_3$) of *1*-(3-phenylpropyl)piperidine *N*-oxide (**2b**):

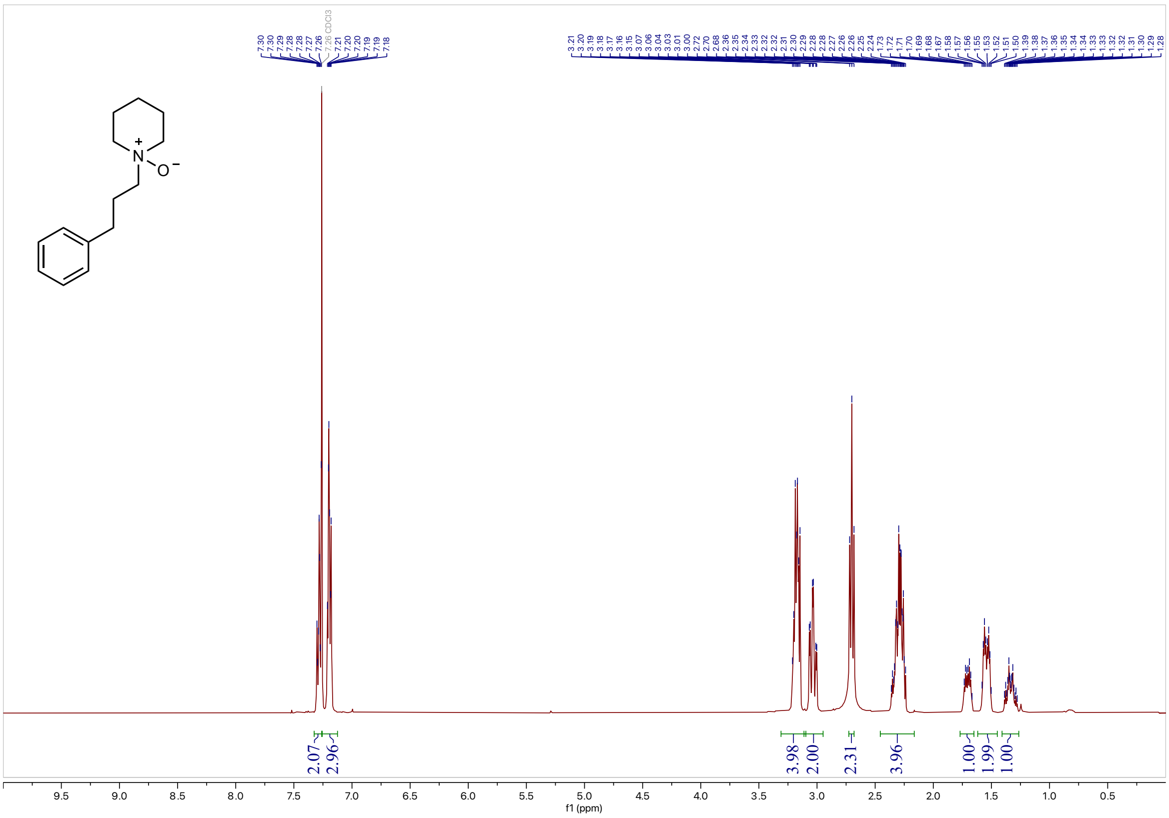

^{13}C{H} NMR (101 MHz, $CDCl_3$) of *1*-(3-phenylpropyl)piperidine *N*-oxide (**2b**):

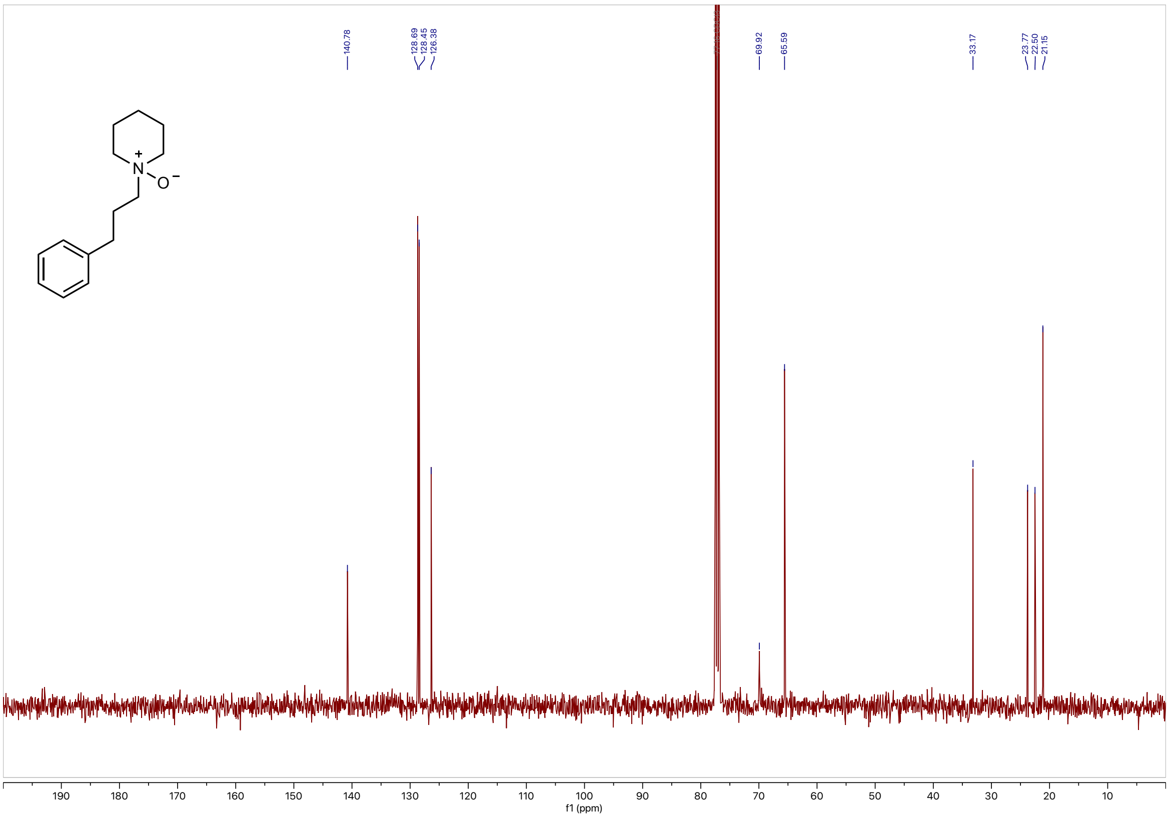

^{1}H NMR (400 MHz, $CDCl_3$) of *1*-methylpiperidine *N*-oxide (**2c**):

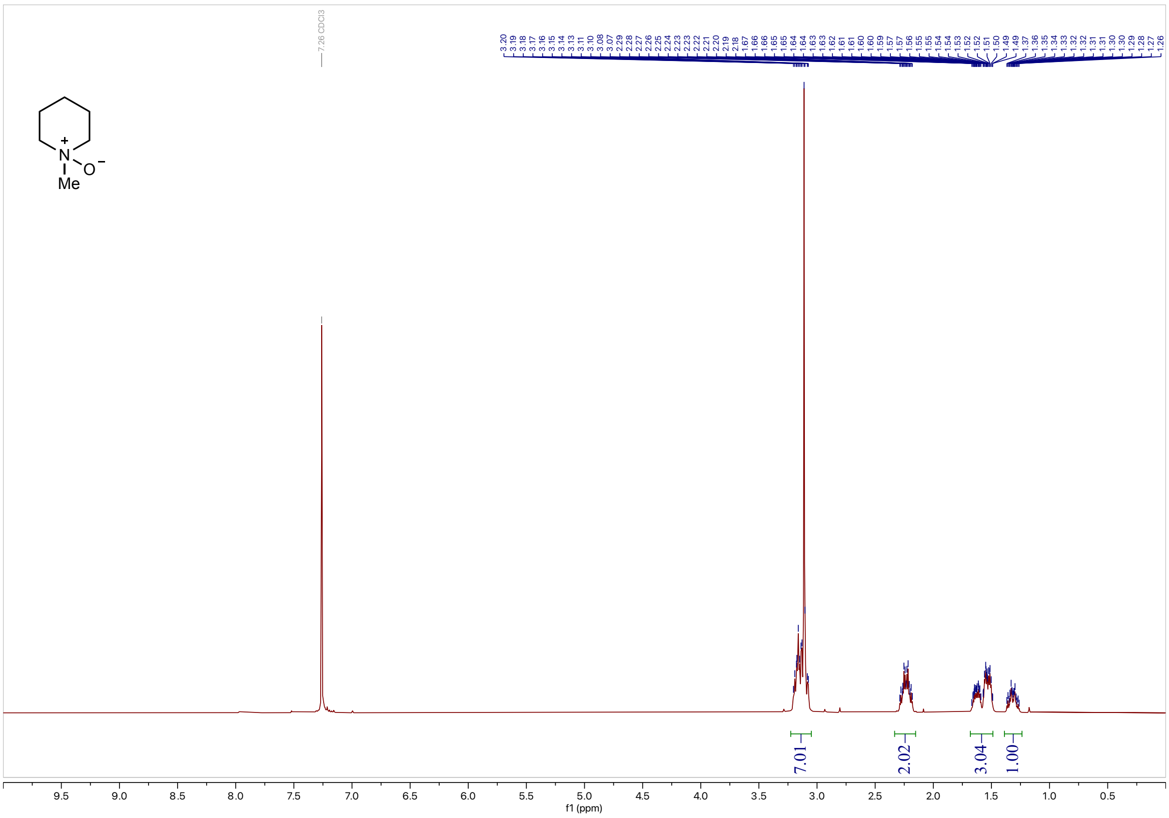

$^{13}C\{H\}$ NMR (126 MHz, $CDCl_3$) of *1*-methylpiperidine *N*-oxide (**2c**):

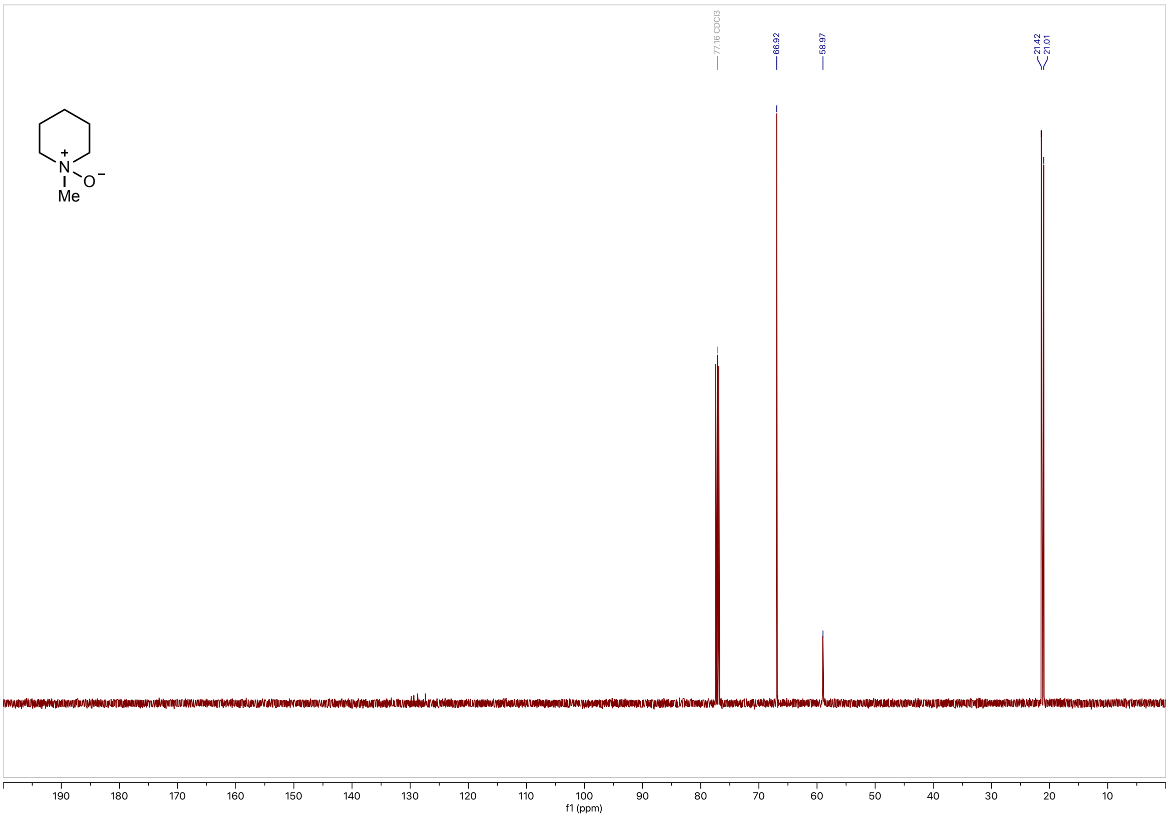

^{1}H NMR (500 MHz, $CDCl_3$) of *1*-cyclohexylpiperidine *N*-oxide (**2d**):

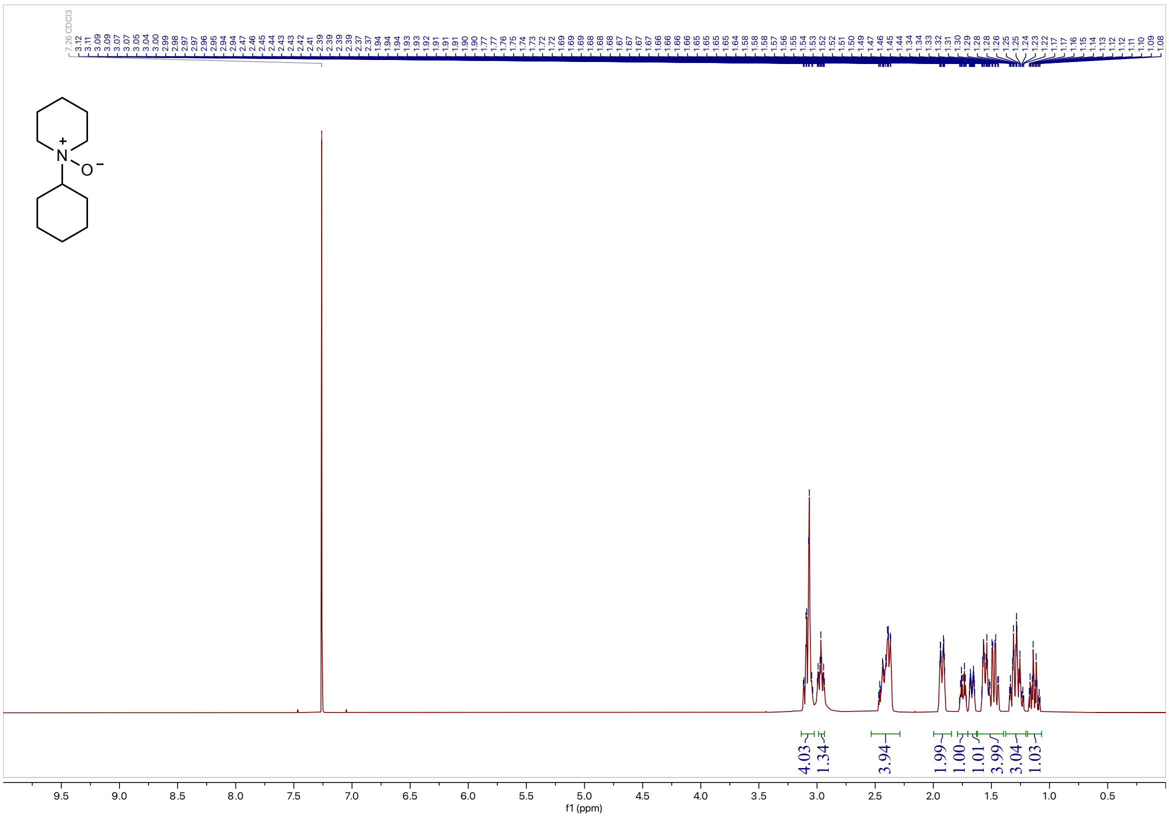

^{13}C{H} NMR (126 MHz, $CDCl_3$) of *1*-cyclohexylpiperidine *N*-oxide (**2d**):

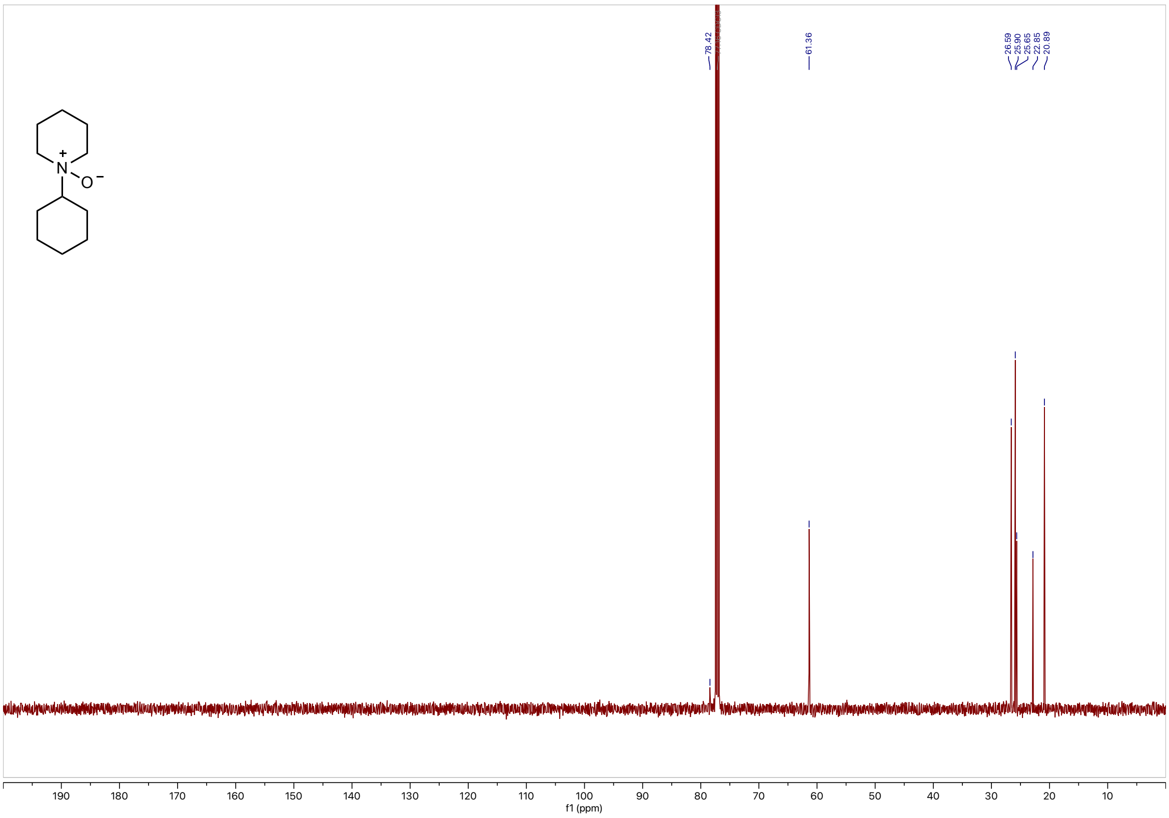

^{1}H NMR (500 MHz, $CDCl_3$) of *1*-phenylpiperidine *N*-oxide (**2e**):

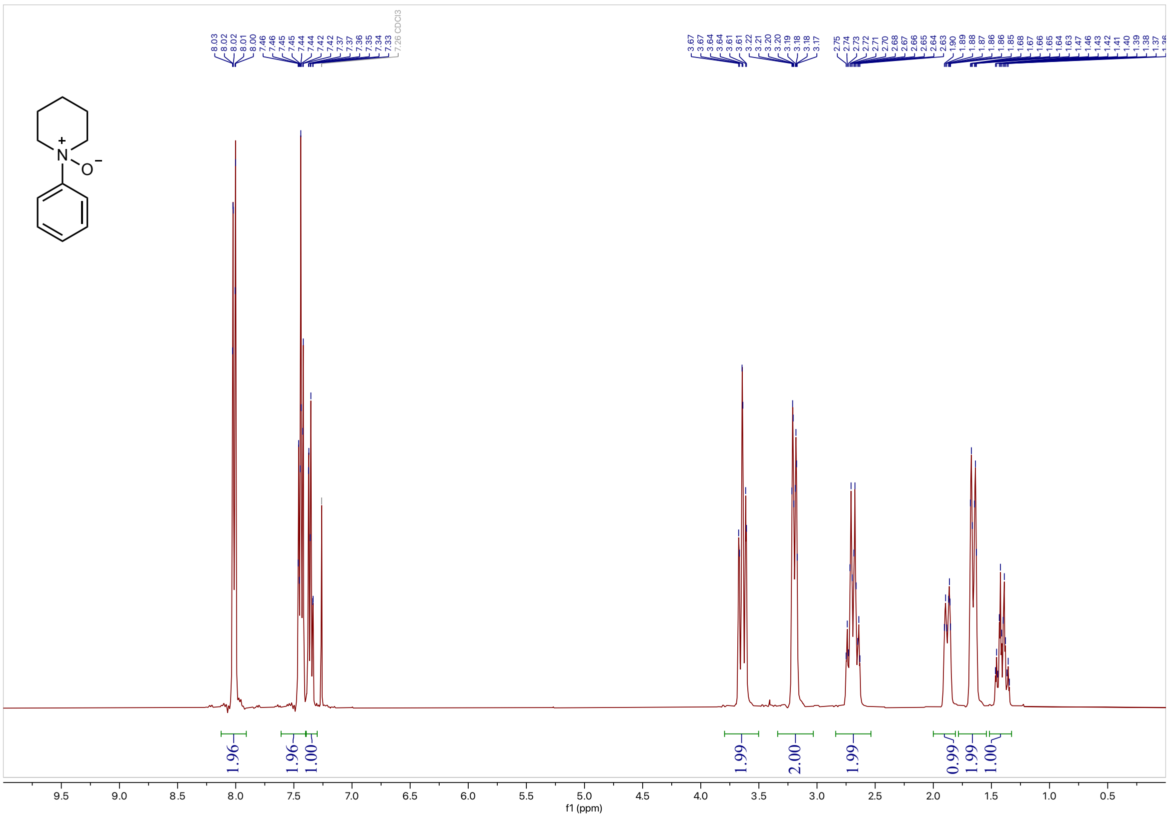

$^{13}C\{H\}$ NMR (101 MHz, $CDCl_3$) of *1*-phenylpiperidine *N*-oxide (**2e**):

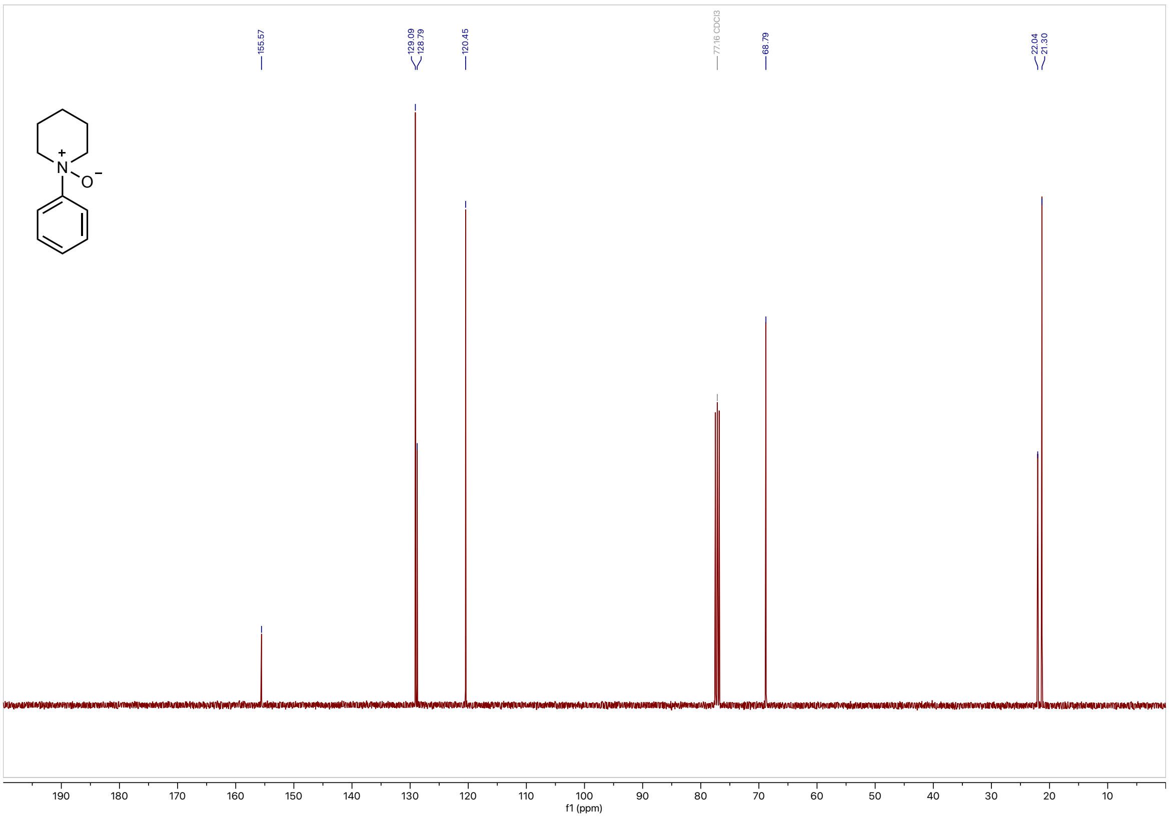

^{1}H NMR (500 MHz, $CDCl_3$) of *1*-benzyl-2-methylpiperidine *N*-oxide (**2f**):

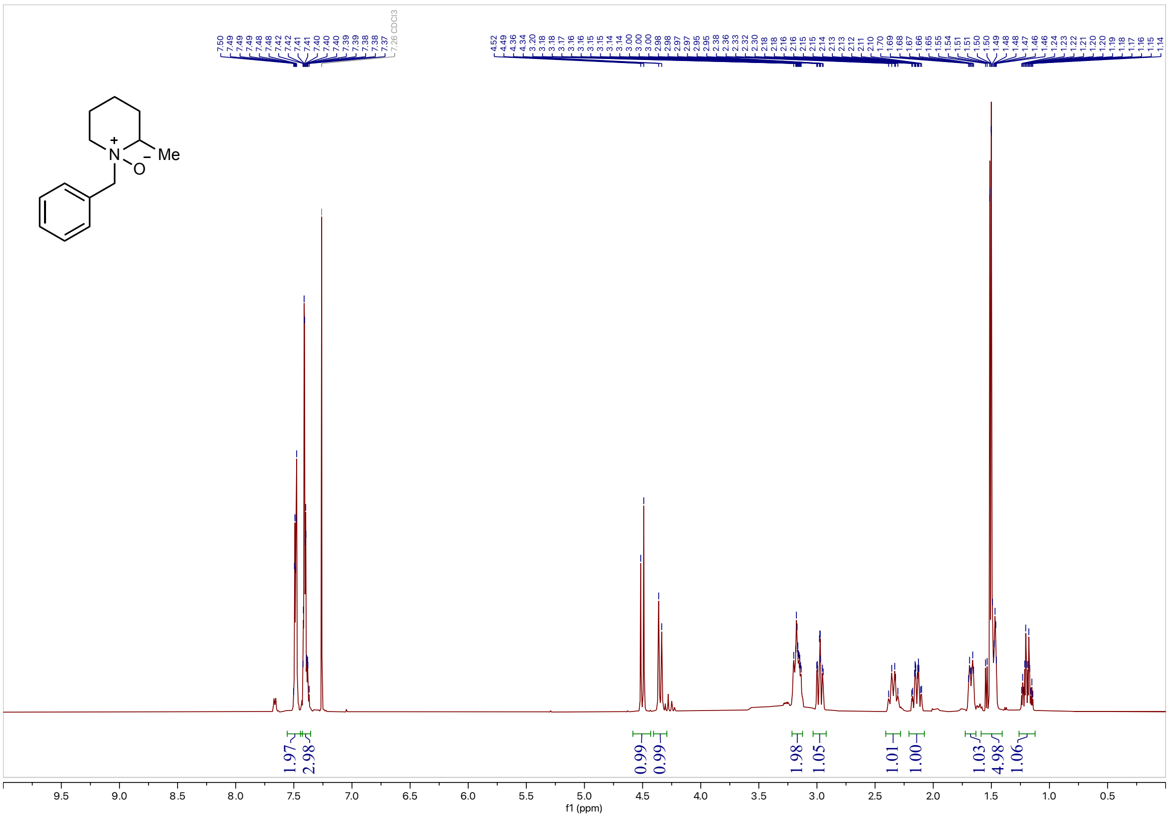

$^{13}C\{H\}$ NMR (126 MHz, $CDCl_3$) of *1*-benzyl-2-methylpiperidine *N*-oxide (**2f**):

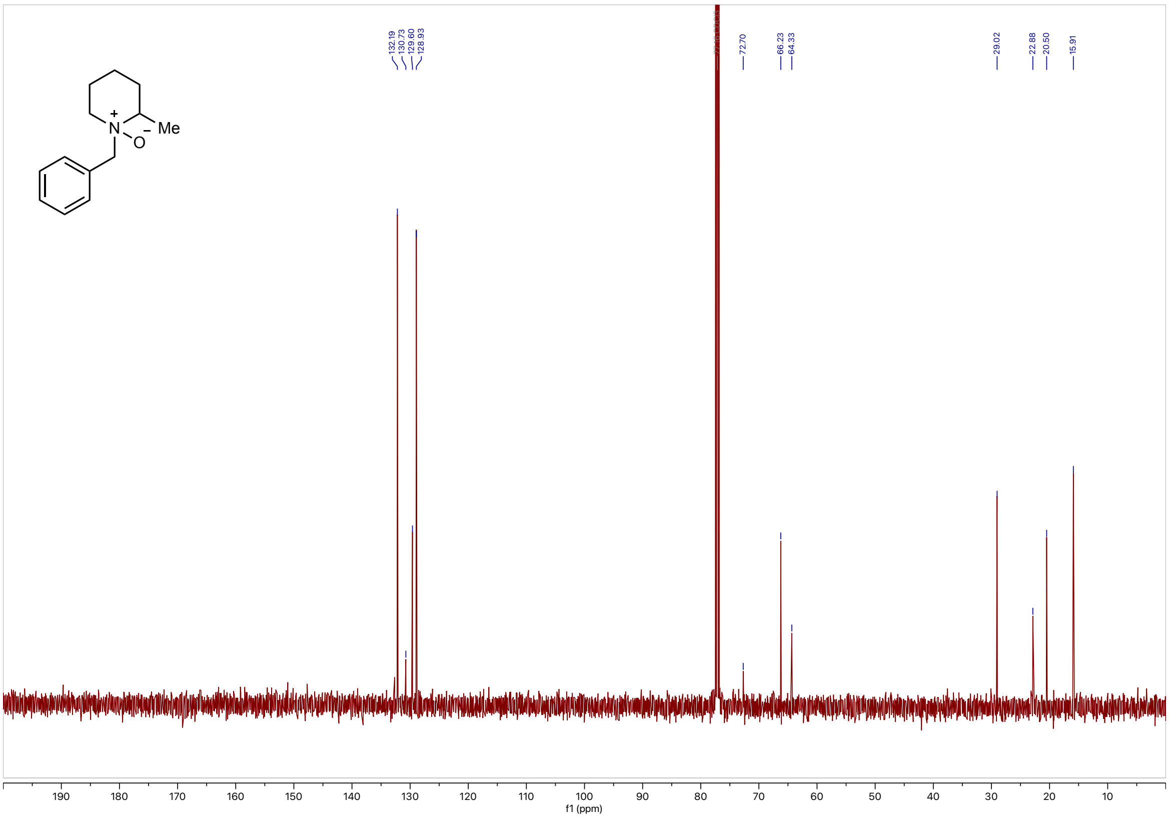

^{1}H NMR (500 MHz, $CDCl_3$) of *1*-benzyl-3-methylpiperidine *N*-oxide (**2g**):

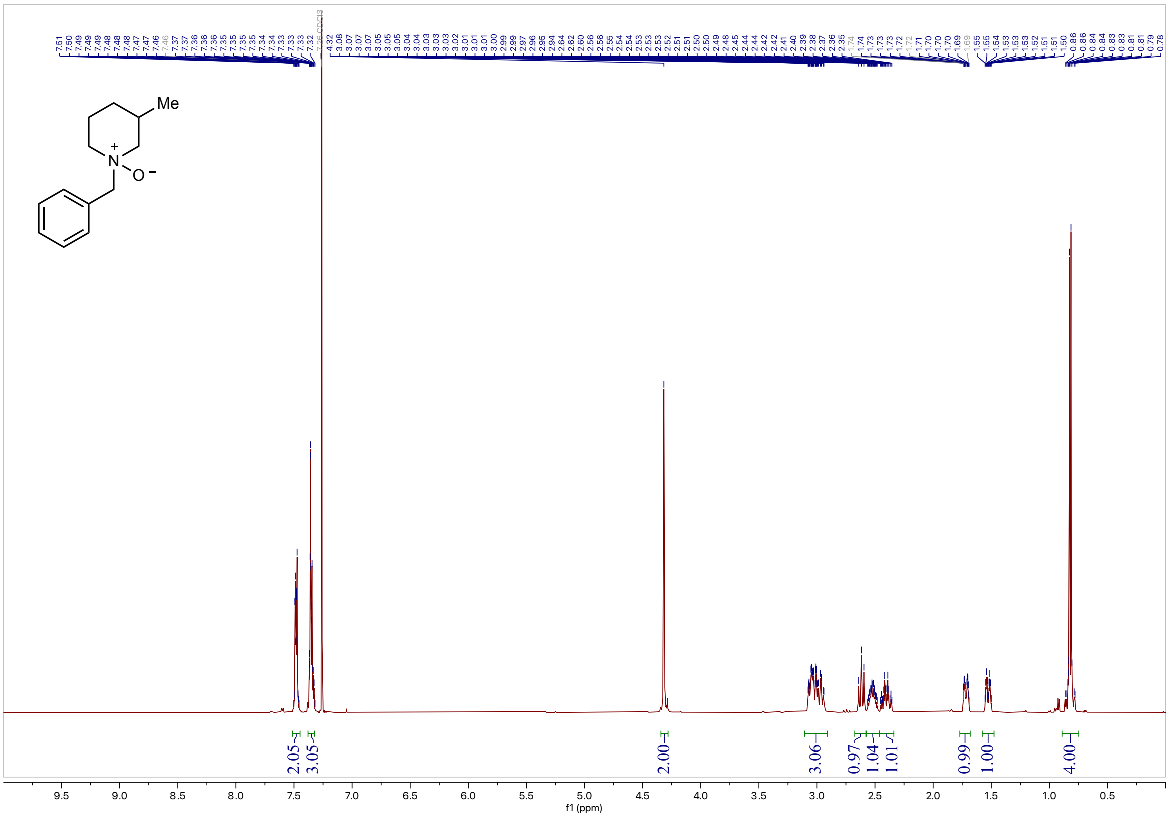

^{13}C{H} NMR (126 MHz, $CDCl_3$) of *1*-benzyl-3-methylpiperidine *N*-oxide (**2g**):

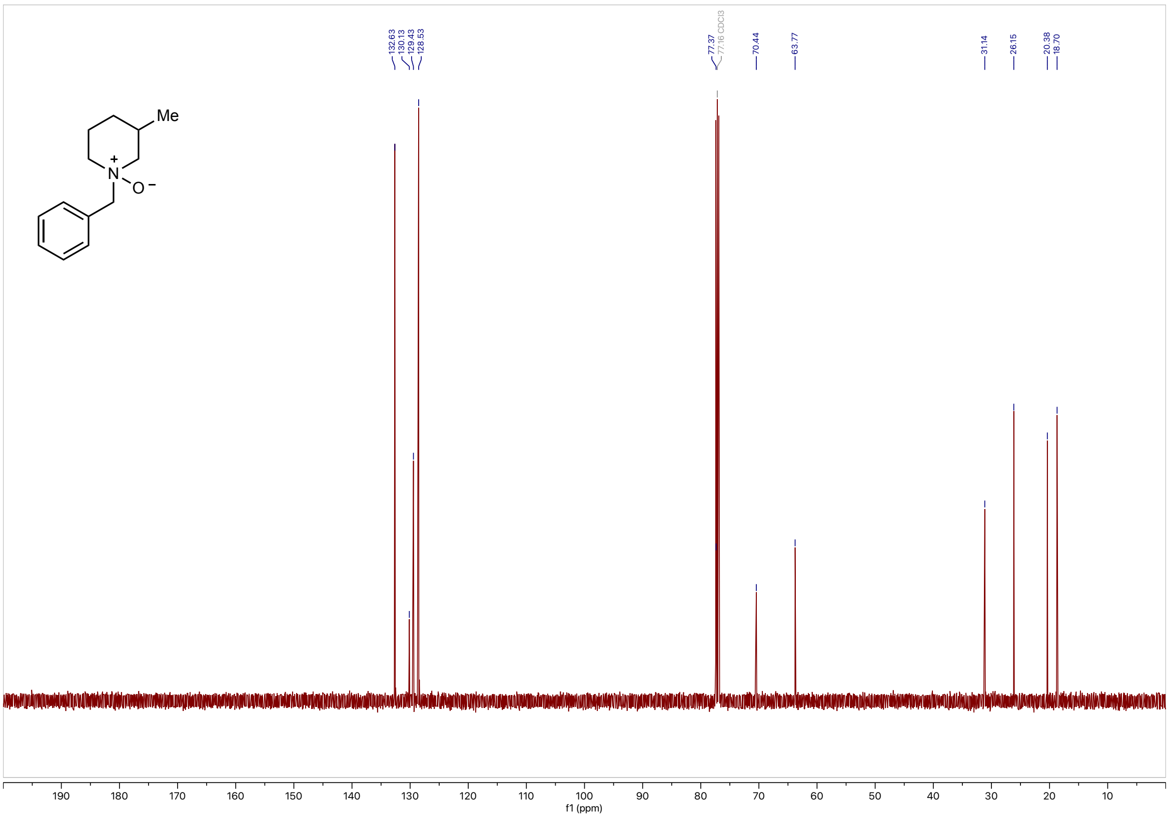

^{1}H NMR (500 MHz, $CDCl_3$) of *1*-benzyl-4-methylpiperidine *N*-oxide (**2h**):

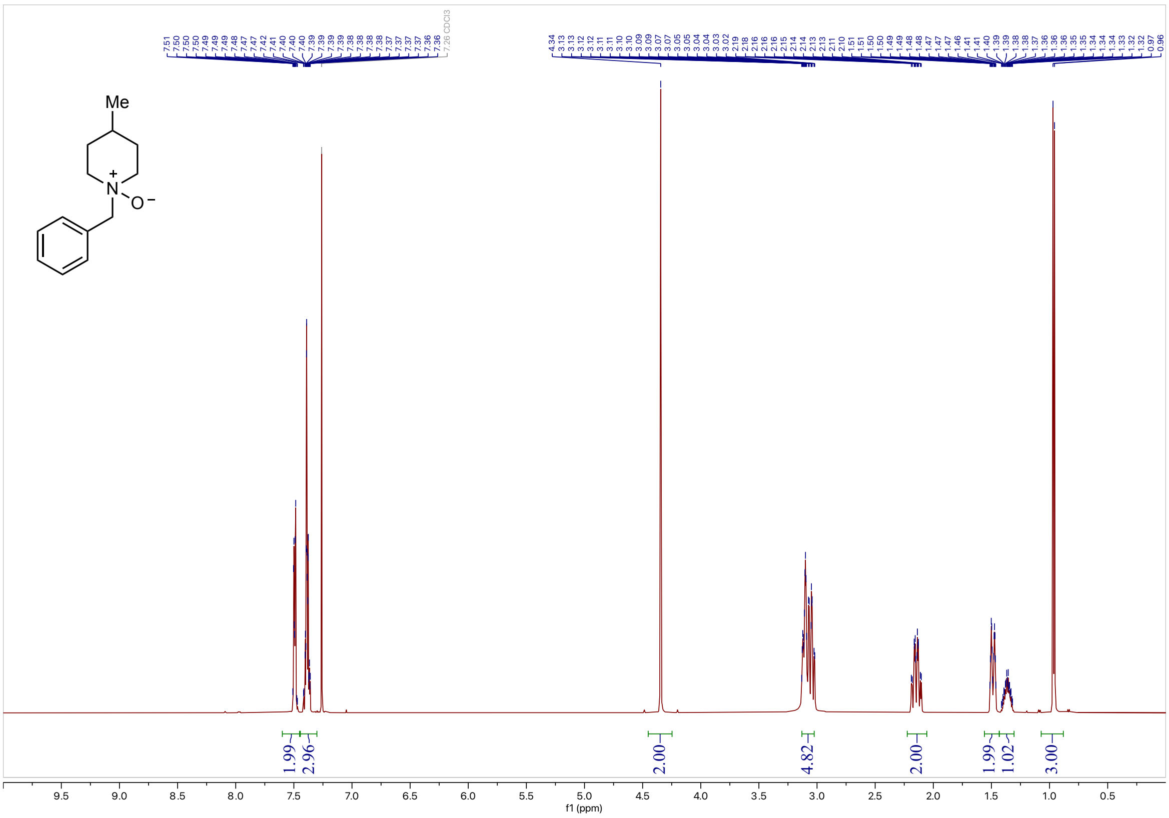

$^{13}C\{H\}$ NMR (126 MHz, $CDCl_3$) of *1*-benzyl-4-methylpiperidine *N*-oxide (**2h**):

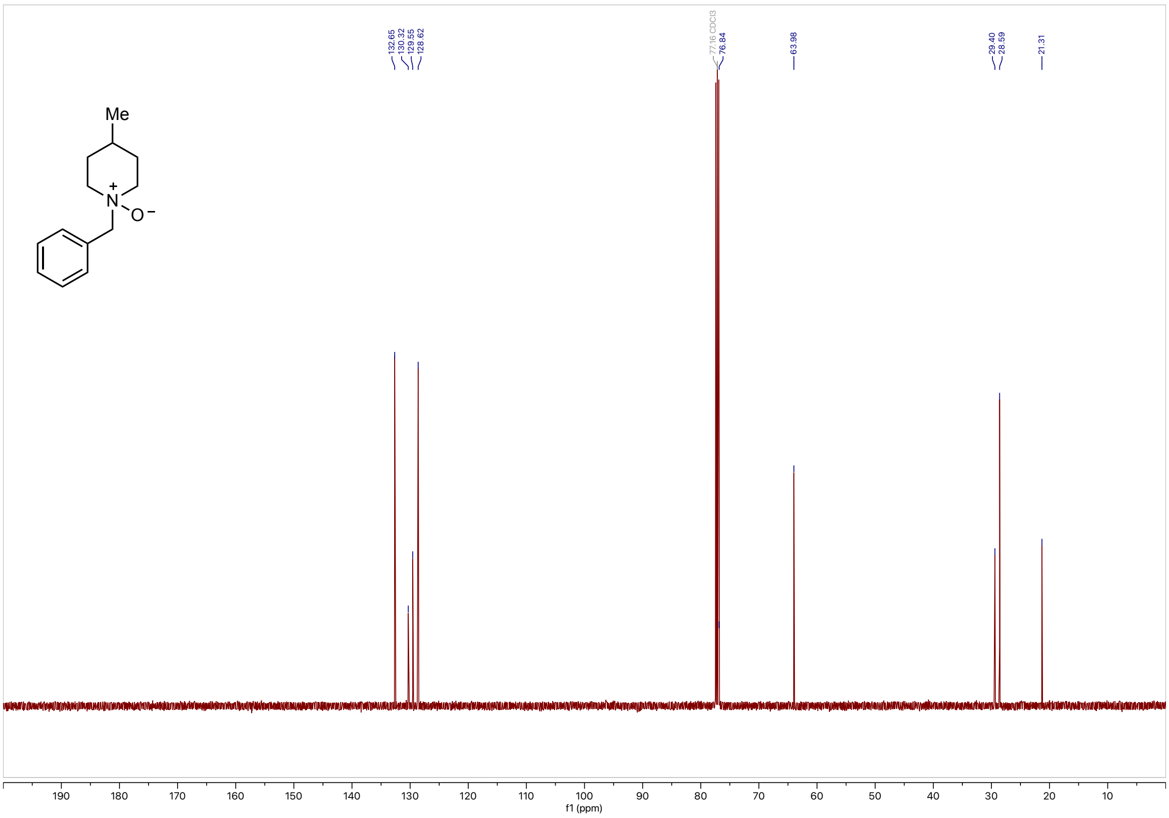

^{1}H NMR (500 MHz, $CDCl_3$) of 1-benzylmorpholine *N*-oxide (**2i**):

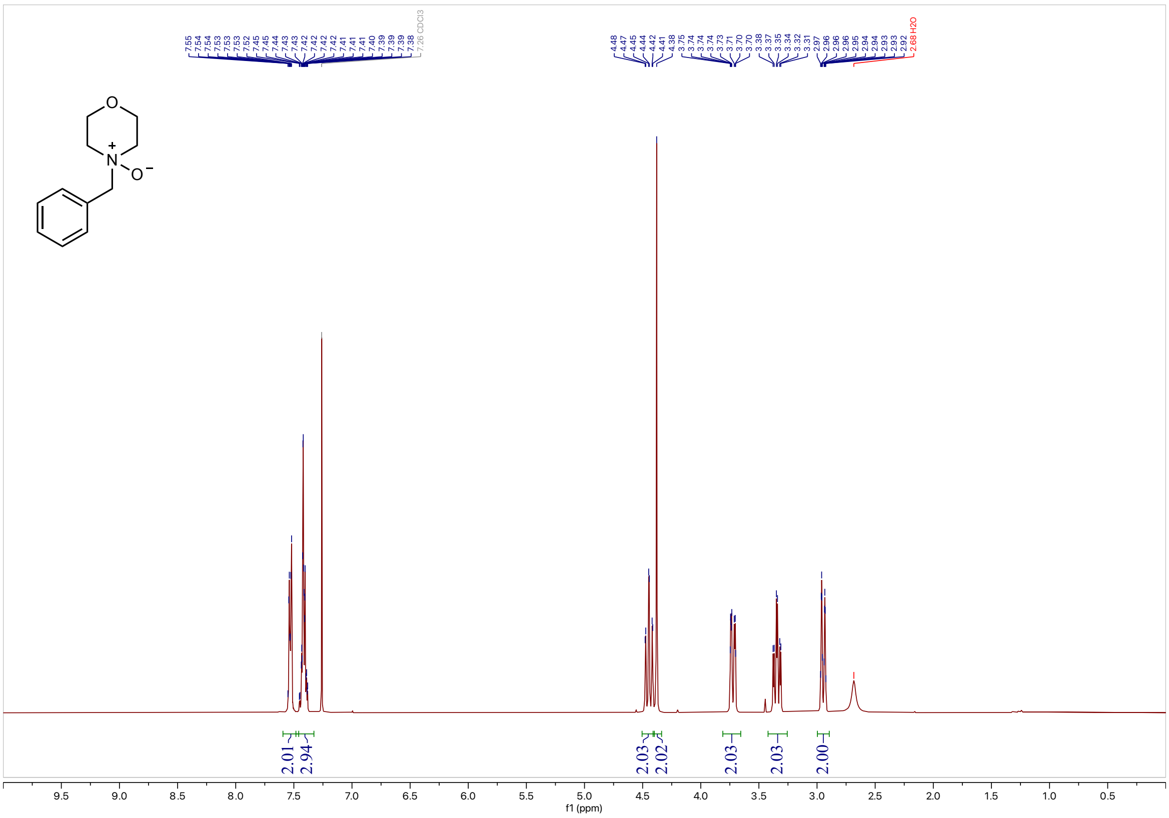

^{13}C{H} NMR (101 MHz, $CDCl_3$) of 1-benzylmorpholine *N*-oxide (**2i**):

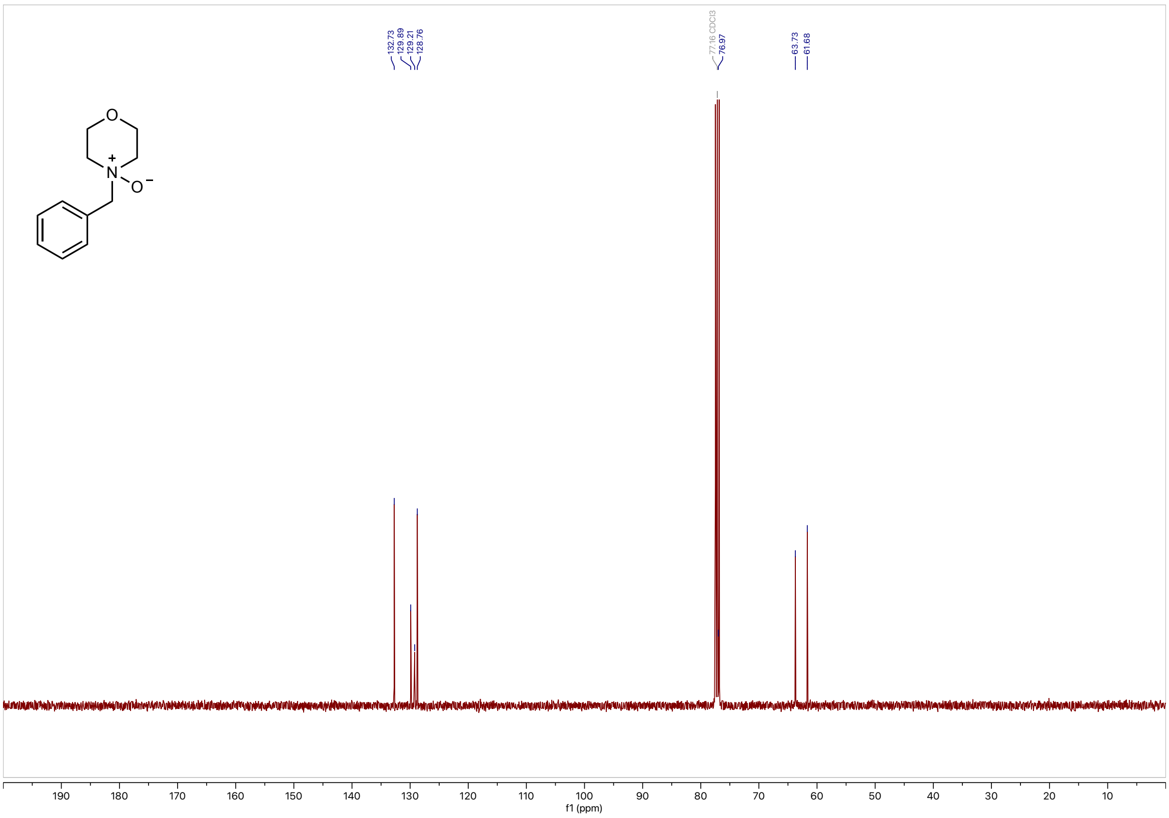

^{1}H NMR (500 MHz, $CDCl_3$) of benzyl 4-benzylpiperazine-1-carboxylate *N*-oxide (**2j**):

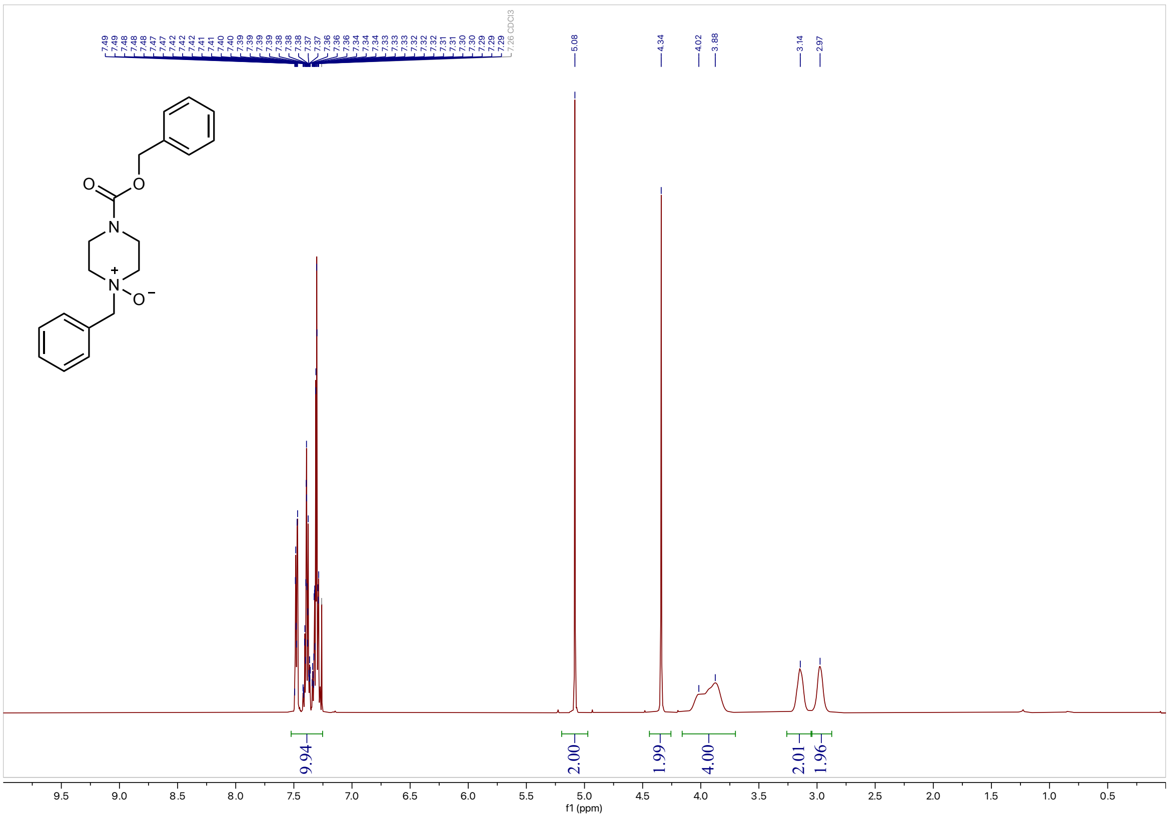

^{13}C{H} NMR (126 MHz, $CDCl_3$) of benzyl 4-benzylpiperazine-1-carboxylate *N*-oxide (**2j**):

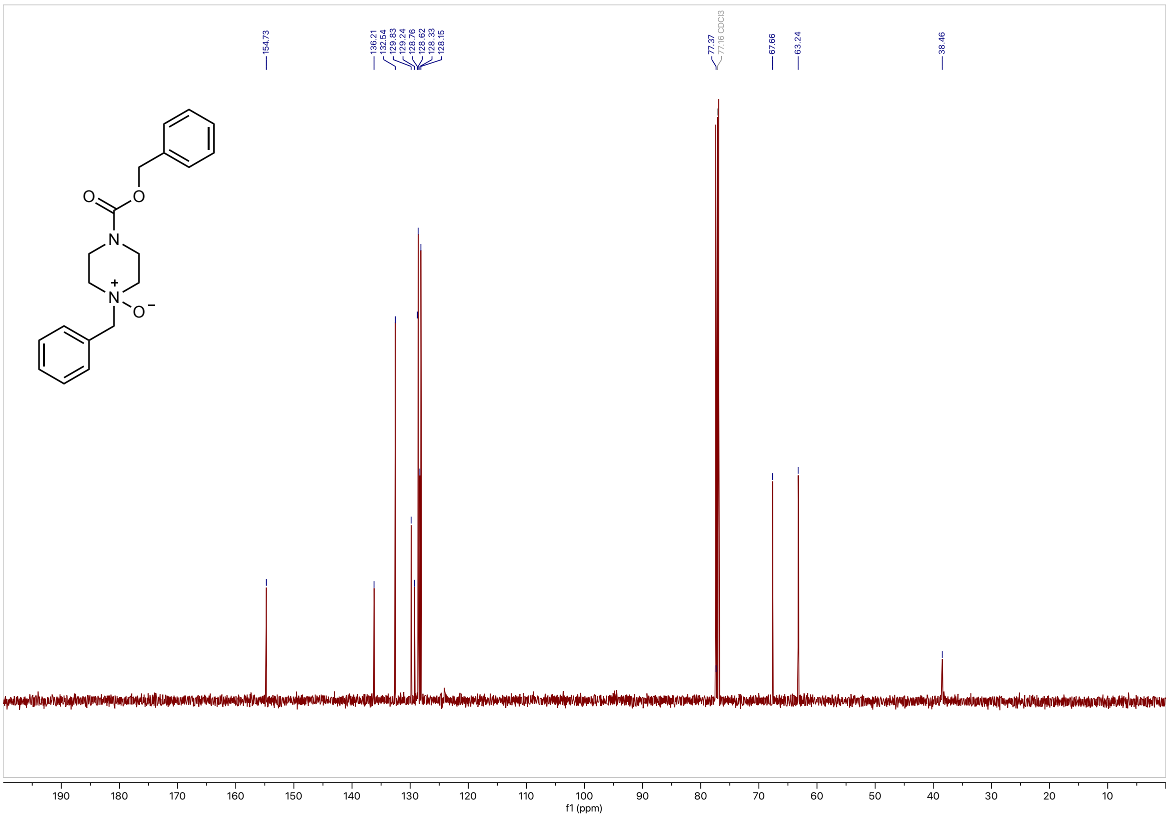

^{1}H NMR (500 MHz, $CDCl_3$) of *1*-benzylpyrrolidine *N*-oxide (**2k**):

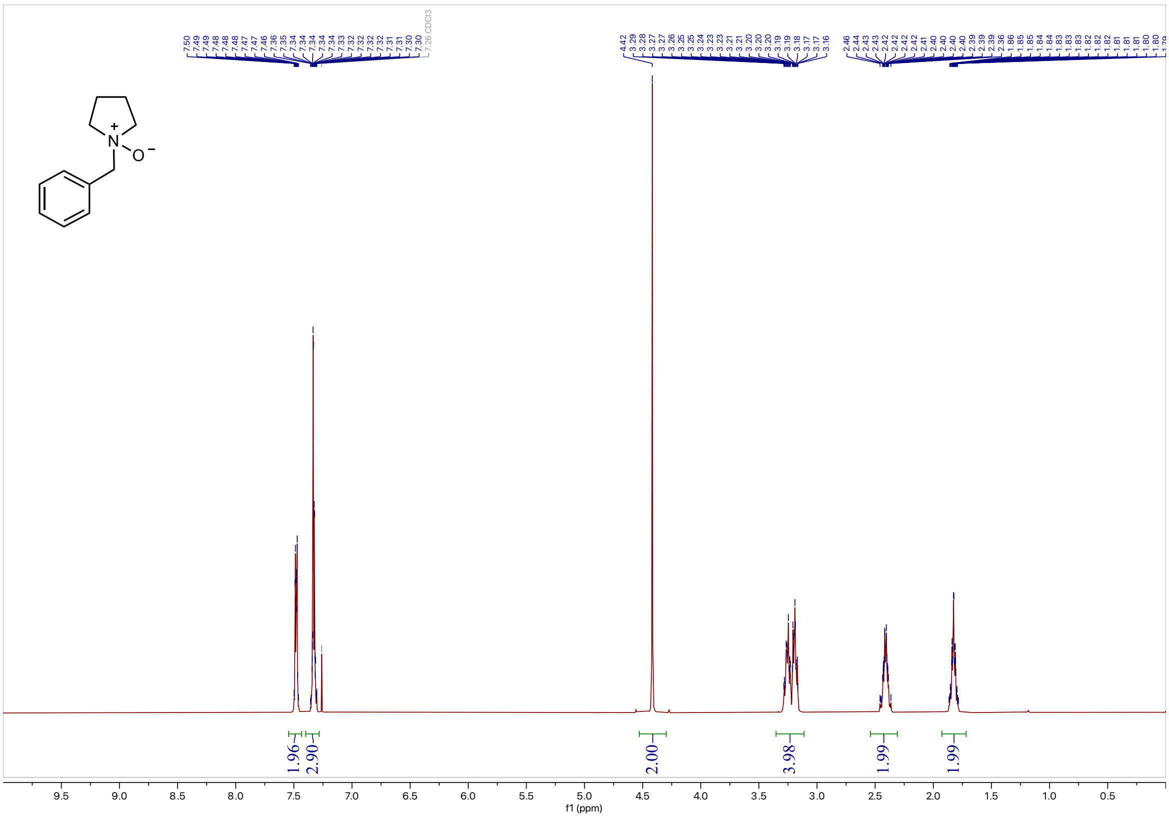

$^{13}C\{H\}$ NMR (126 MHz, $CDCl_3$) of *1*-benzylpyrrolidine *N*-oxide (**2k**):

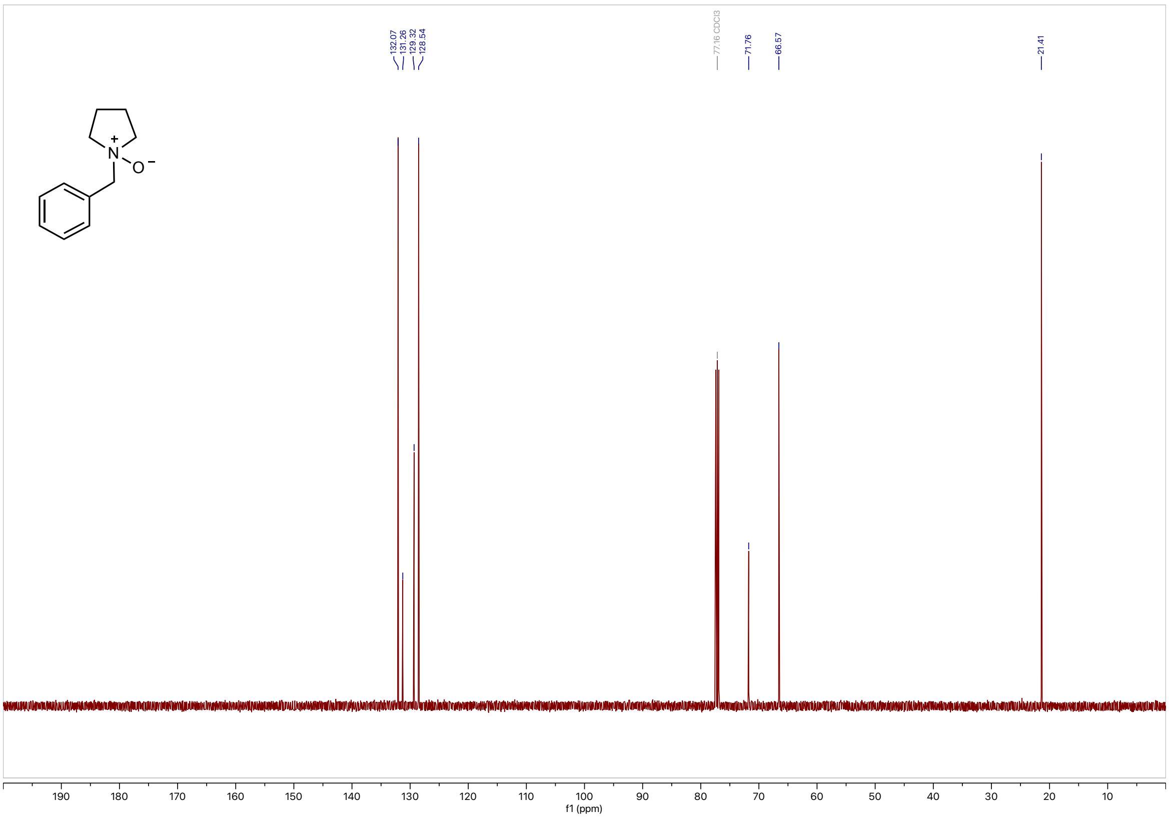

^{1}H NMR (500 MHz, $CDCl_3$) of *1*-benzylazepan *N*-oxide (**2l**):

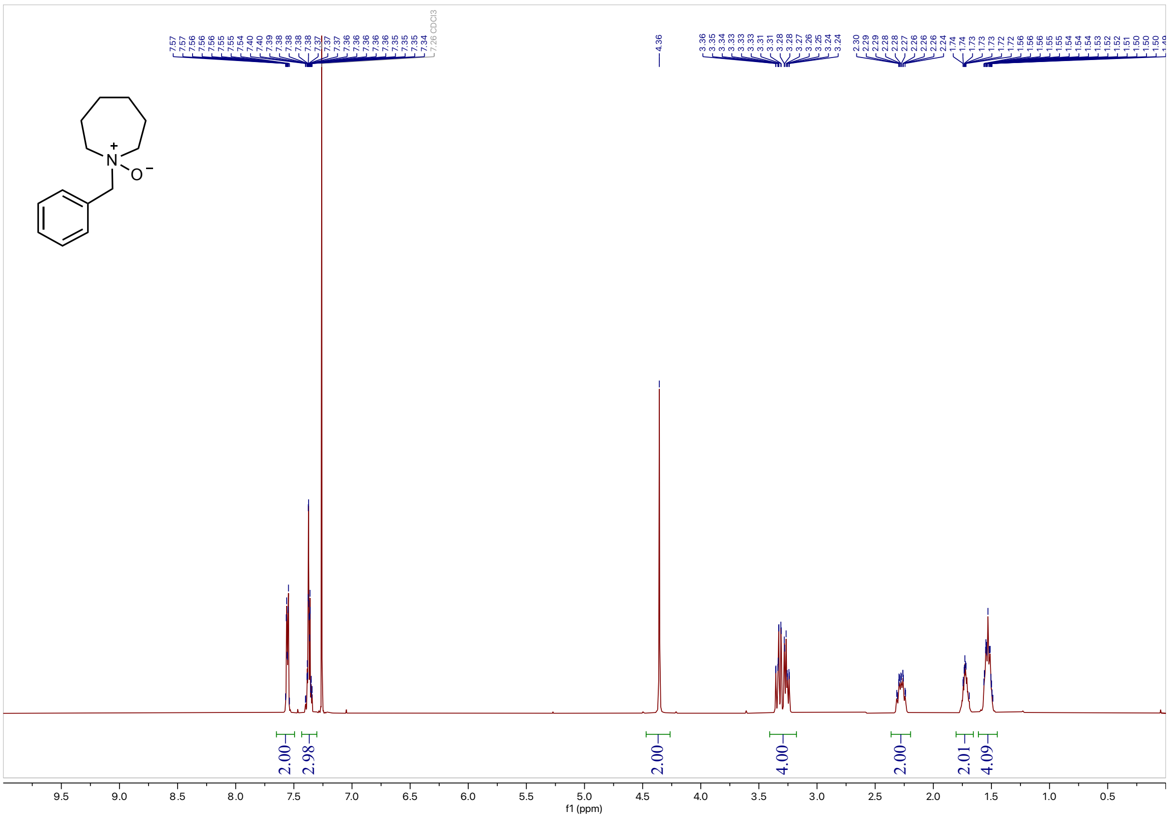

$^{13}C\{H\}$ NMR (126 MHz, $CDCl_3$) of *1*-benzylazepan *N*-oxide (**2l**):

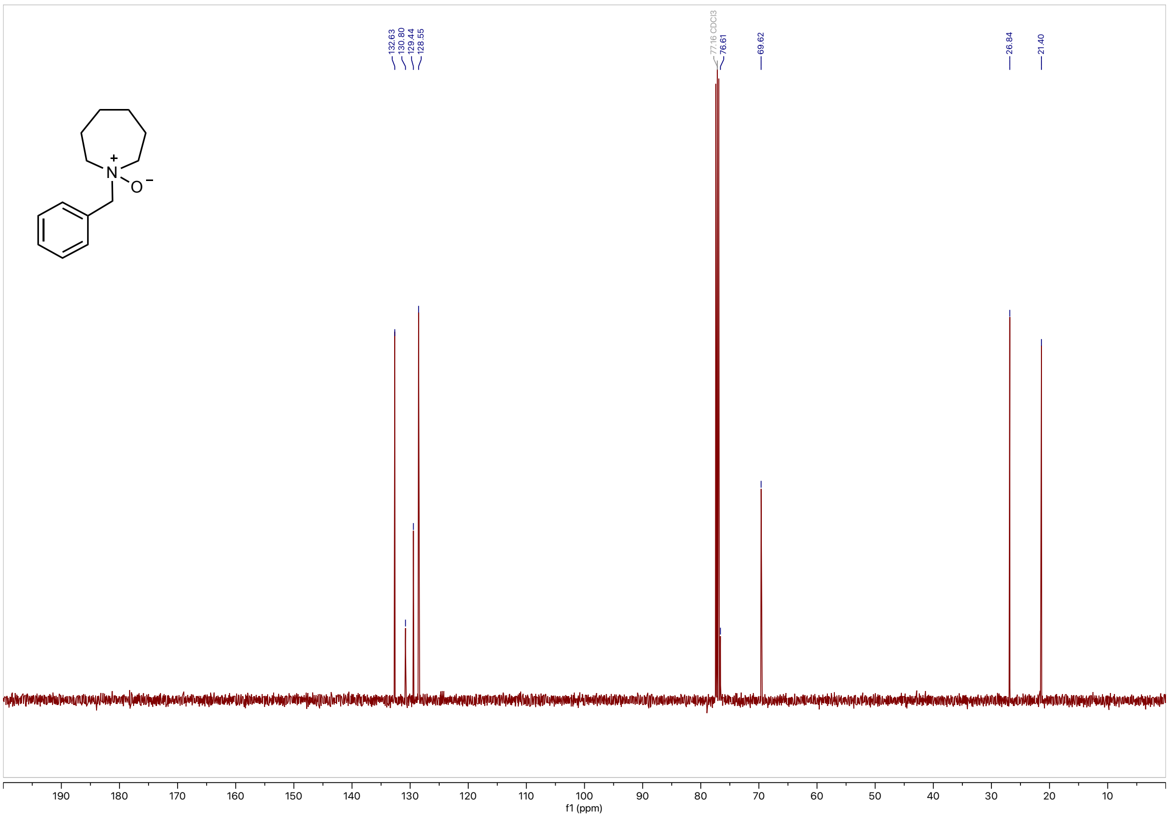

^{1}H NMR (500 MHz, $CDCl_3$) of *2*-benzyl-1,2,3,4-tetrahydroisoquinoline *N*-oxide (**2m**):

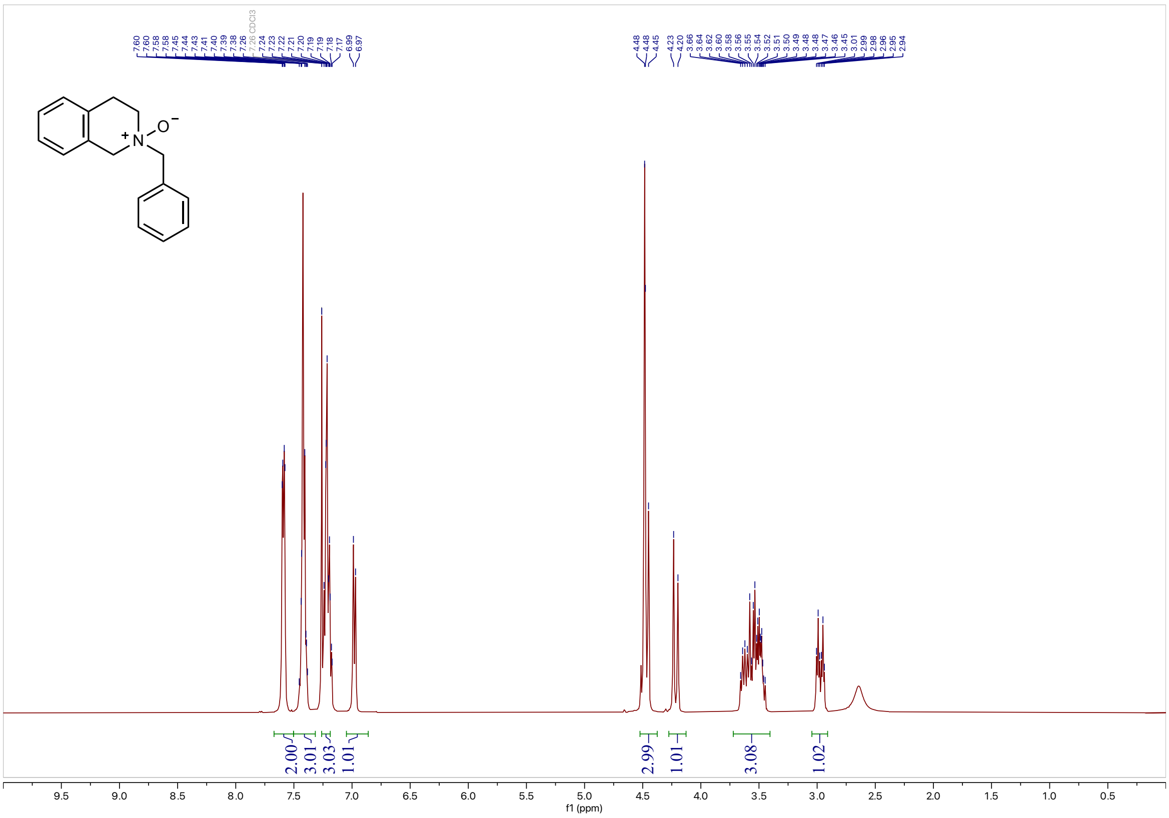

^{13}C{H} NMR (101 MHz, $CDCl_3$) of *2*-benzyl-1,2,3,4-tetrahydroisoquinoline *N*-oxide (**2m**):

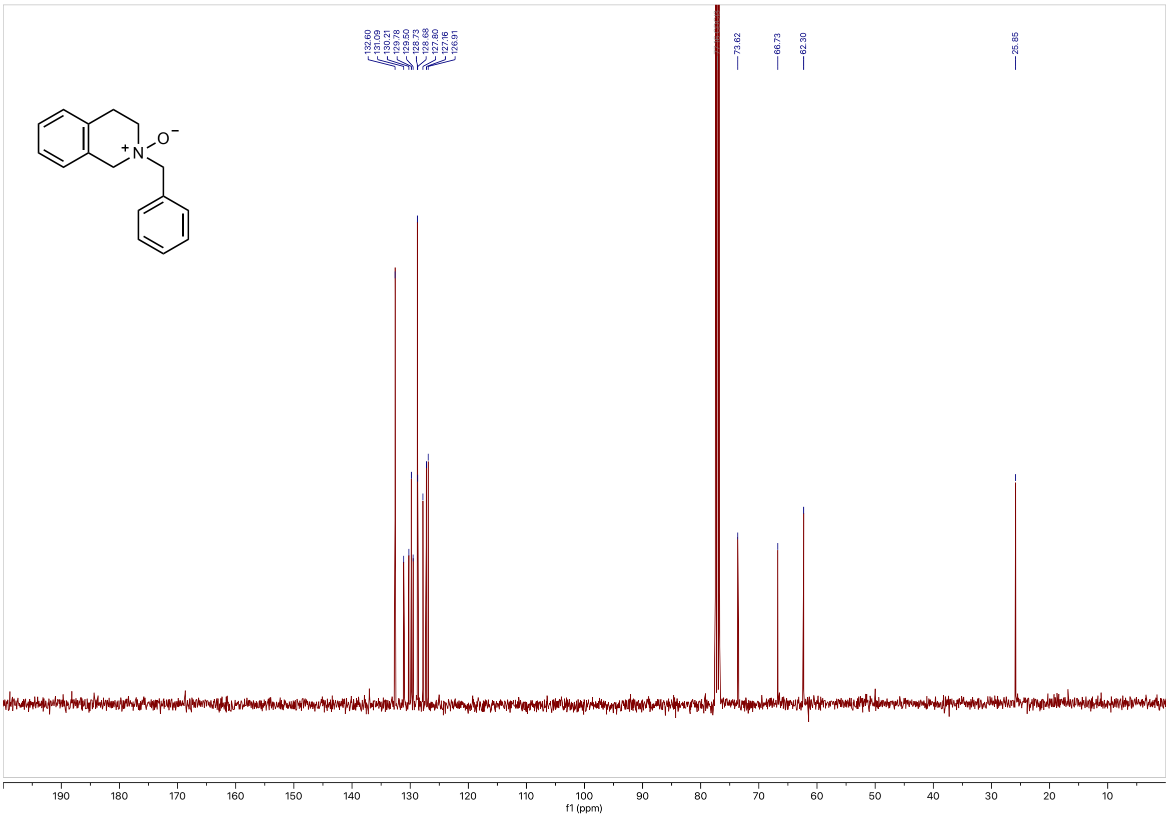

^{1}H NMR (500 MHz, $CDCl_3$) of *1*-benzyl-1,2,3,4-tetrahydroquinoline *N*-oxide (**2n**):

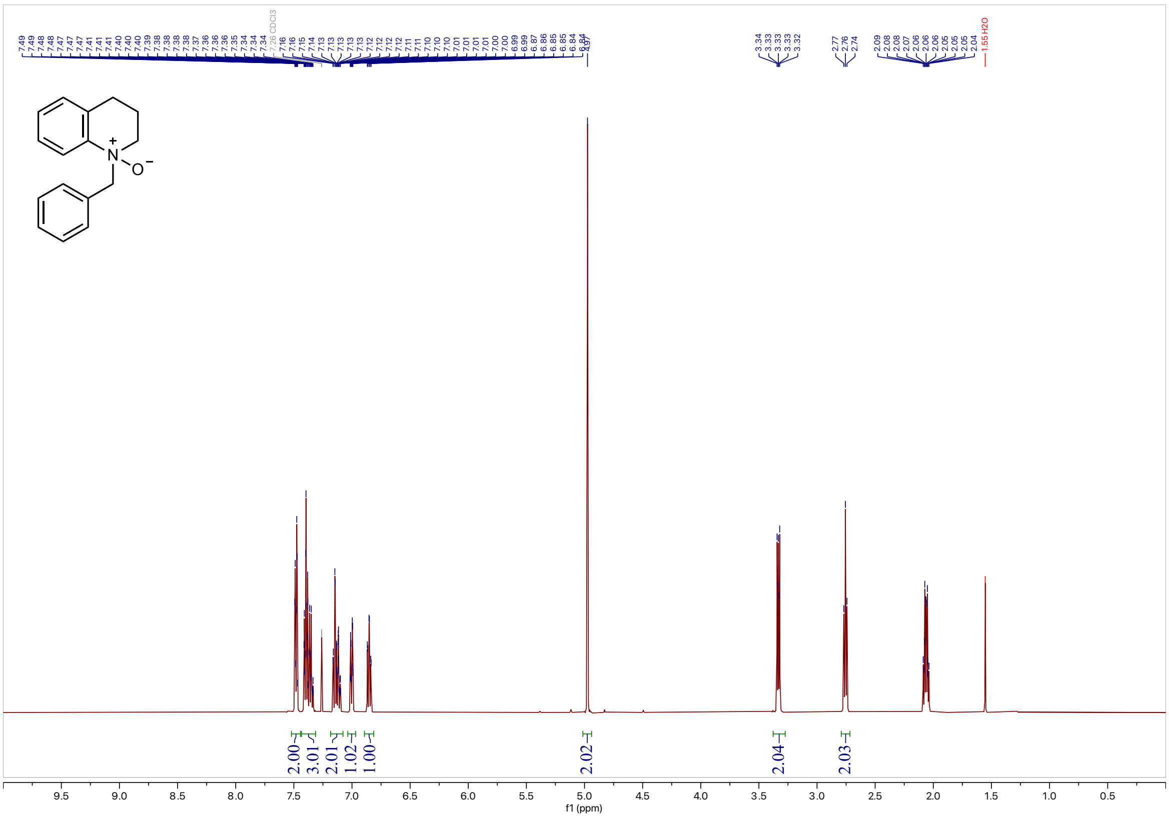

$^{13}C\{H\}$ NMR (126 MHz, $CDCl_3$) of 1-benzyl-1,2,3,4-tetrahydroquinoline *N*-oxide (**2n**):

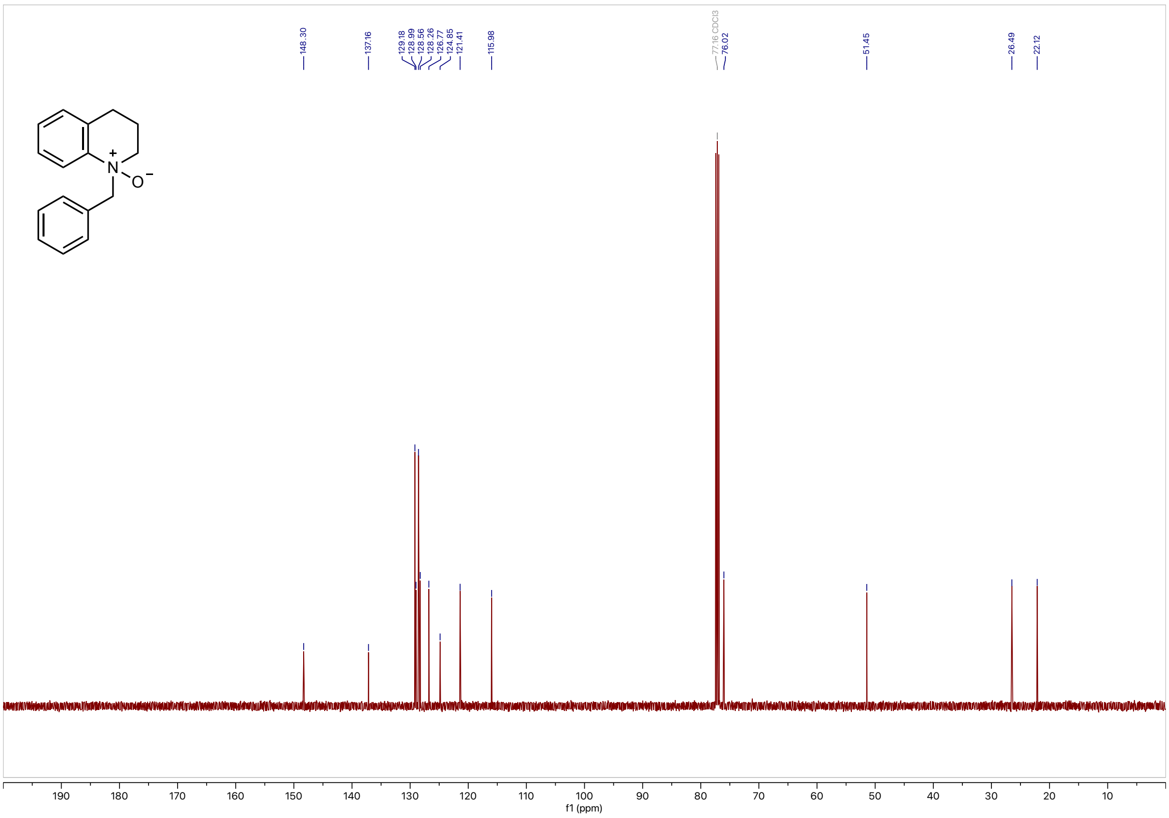

^{1}H NMR (700 MHz, $CDCl_3$) of 1-(pyridin-2-ylmethyl)piperidine *N*-oxide (**2o**):

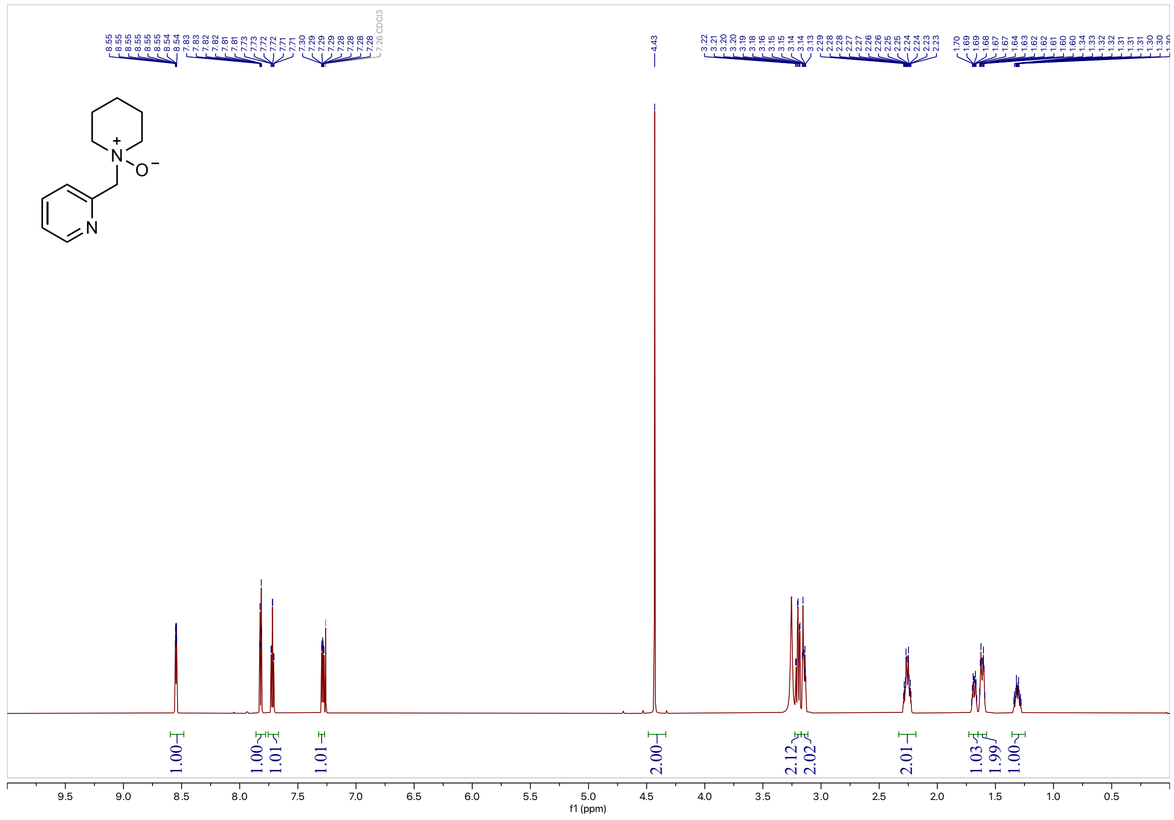

$^{13}C\{H\}$ NMR (176 MHz, $CDCl_3$) of 1-(pyridin-2-ylmethyl)piperidine *N*-oxide (**2o**):

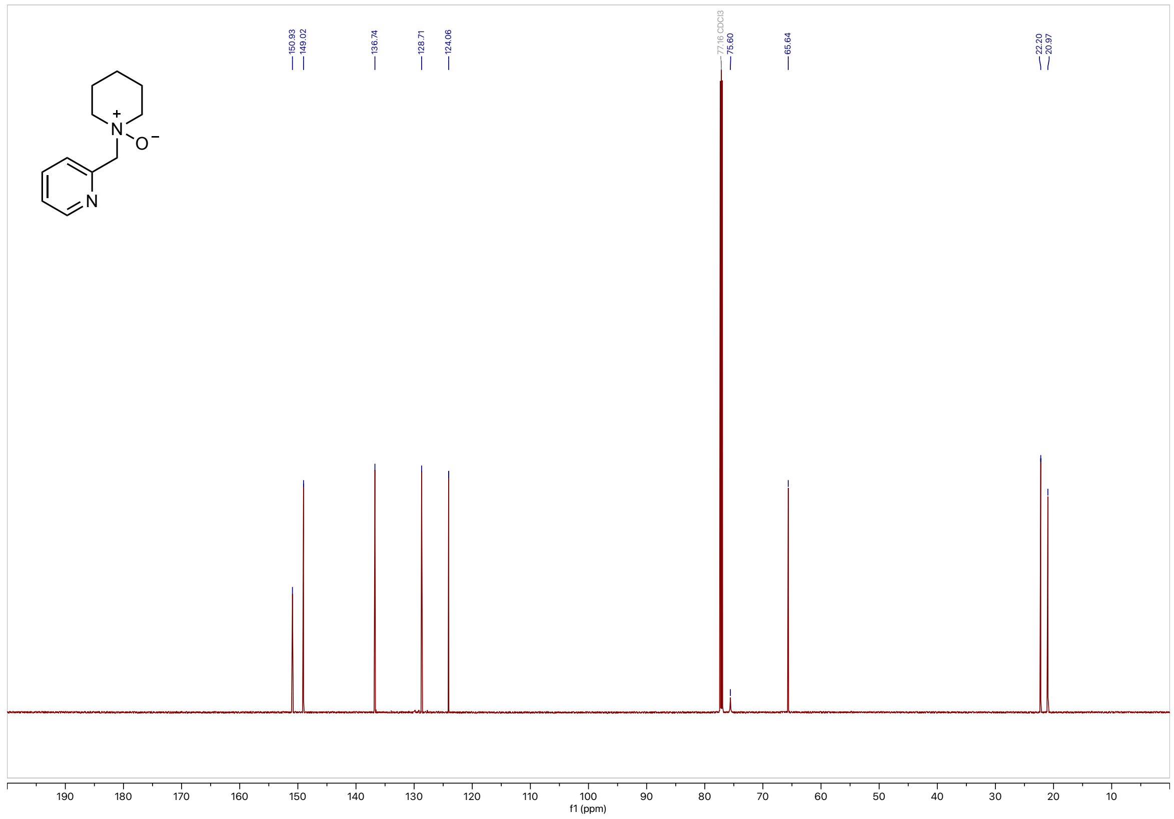

^{1}H NMR (500 MHz, $CDCl_3$) of *1*-(3-phenylpropyl)pyrrolidine *N*-oxide (**2p**):

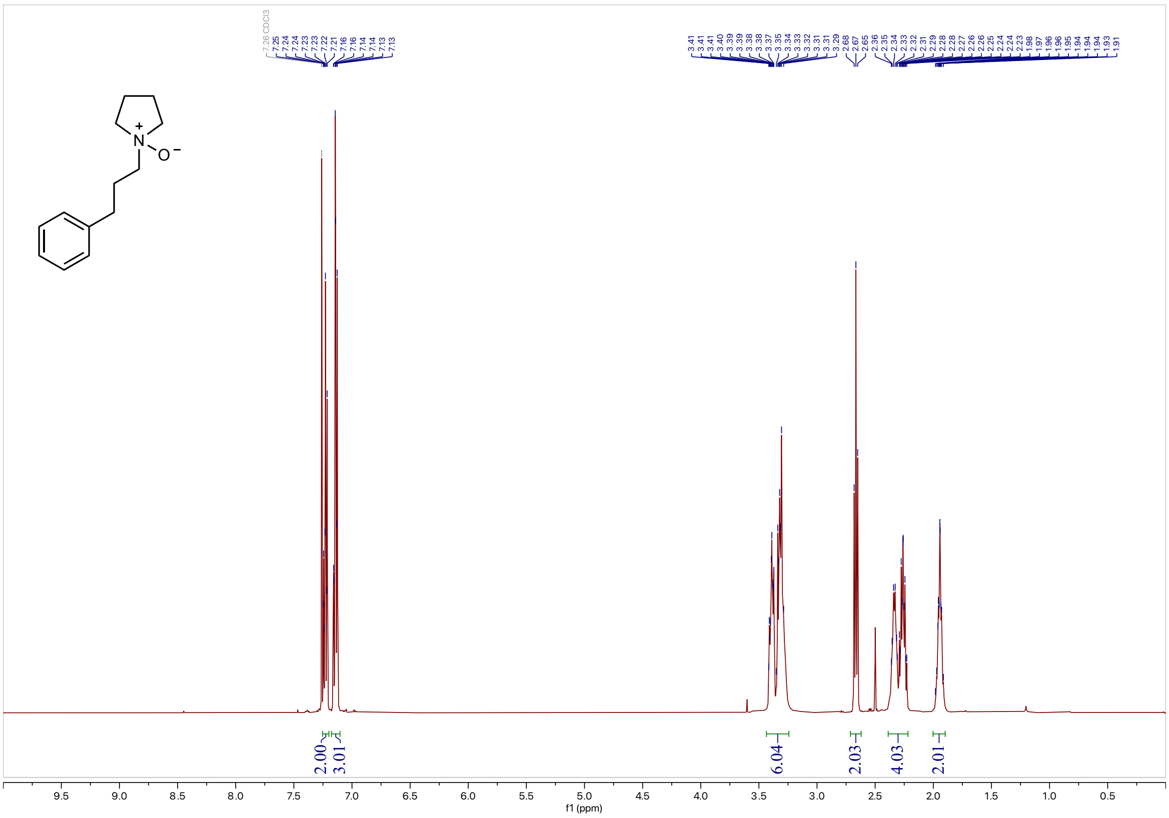

$^{13}C\{H\}$ NMR (126 MHz, $CDCl_3$) of *1*-(3-phenylpropyl)pyrrolidine *N*-oxide (**2p**):

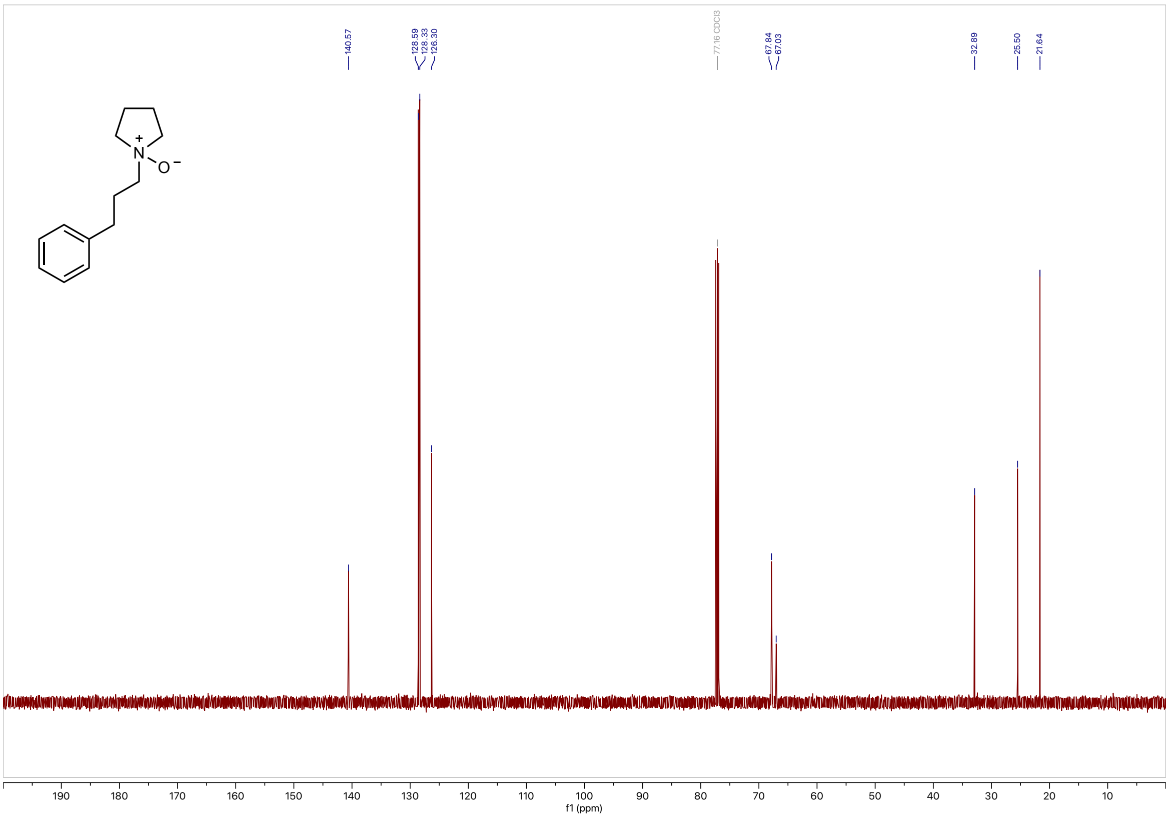

^{1}H NMR (500 MHz, $CDCl_3$) of *N*-cyclohexylpyrrolidine *N*-oxide (**2q**):

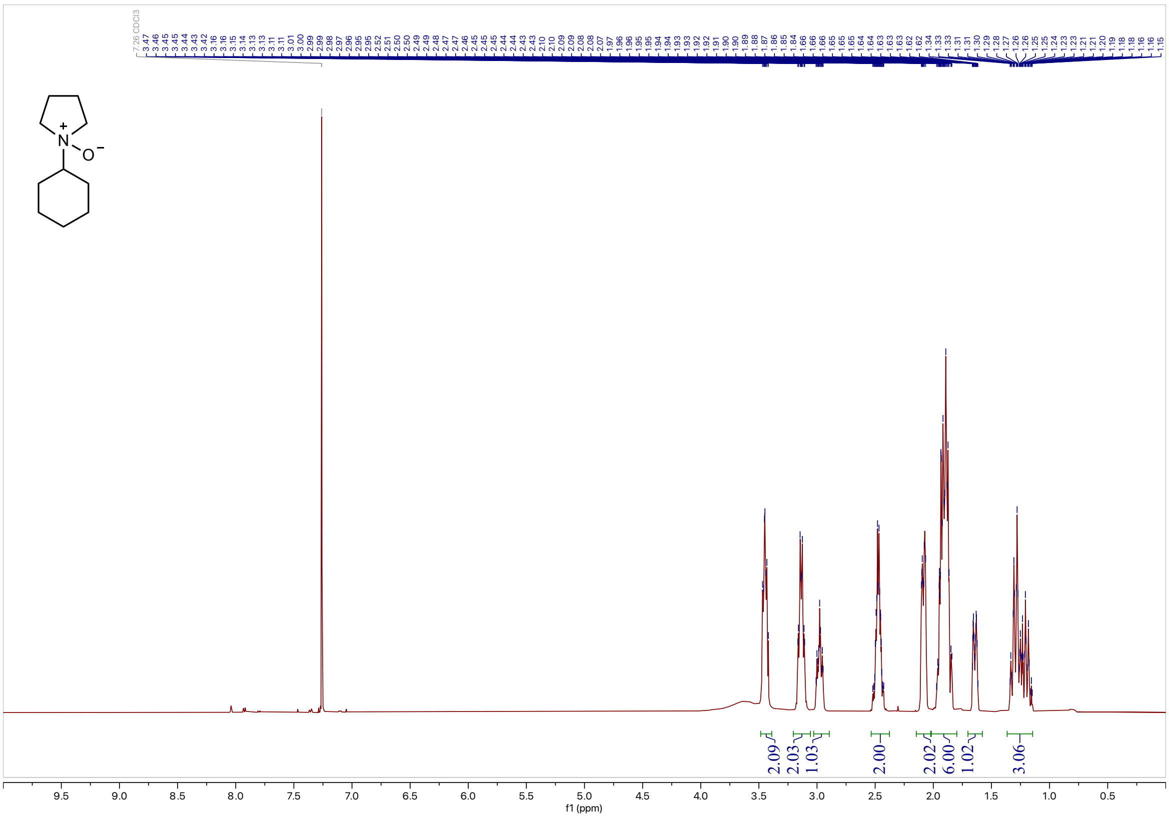

$^{13}C\{H\}$ NMR (126 MHz, $CDCl_3$) of *N*-cyclohexylpyrrolidine *N*-oxide (**2q**):

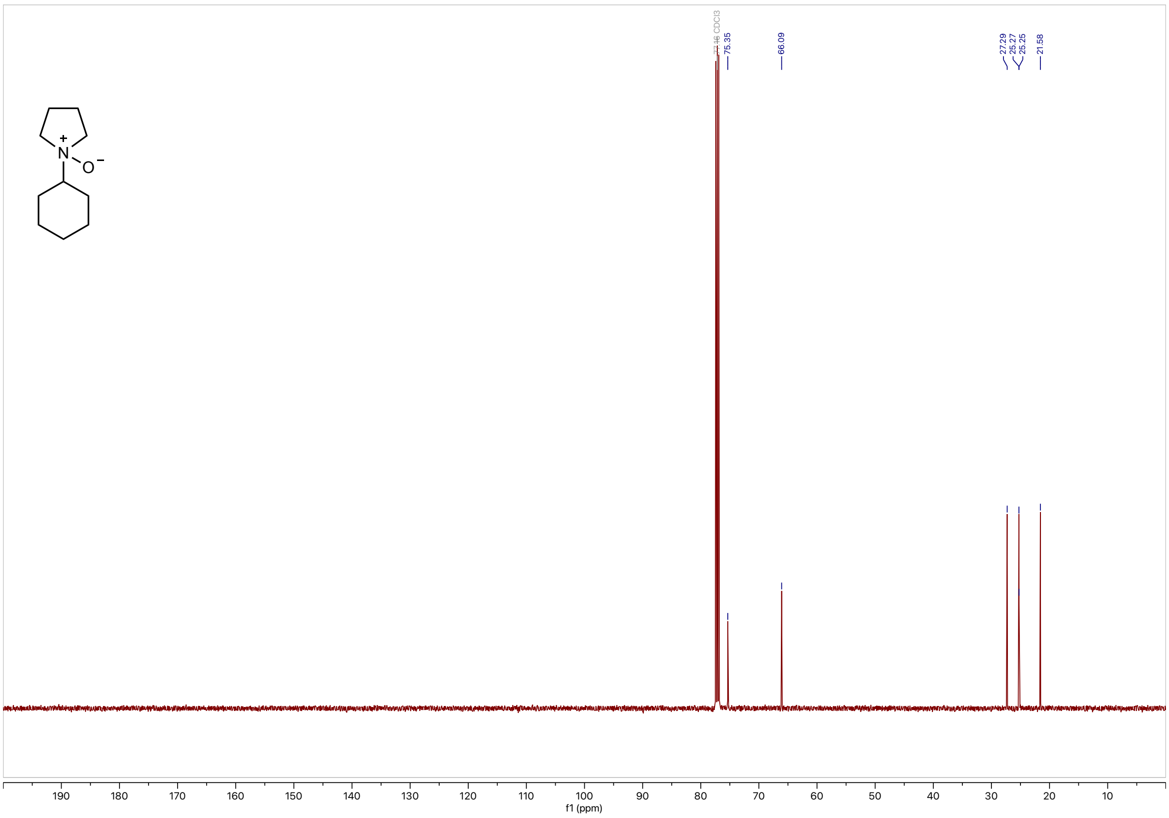

^{1}H NMR (500 MHz, $CDCl_3$) of 1-(3-phenylpropyl)morpholine *N*-oxide (**2r**)

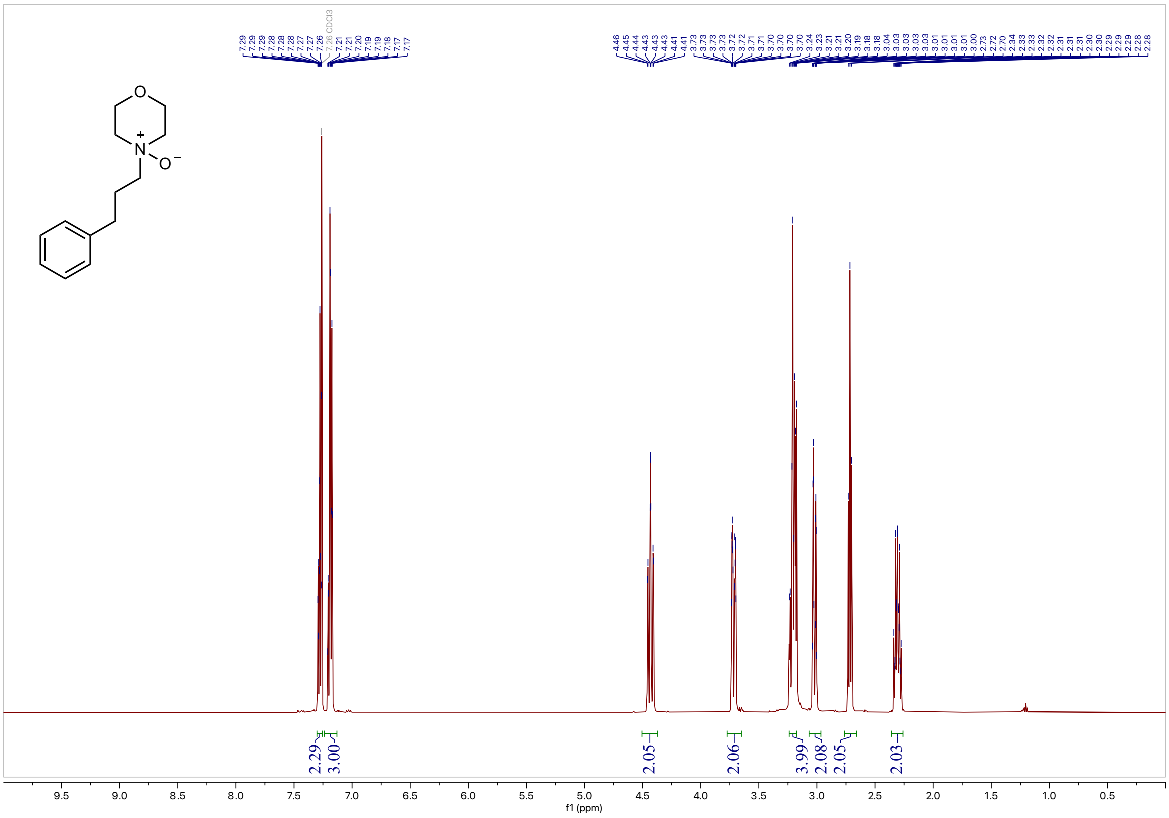

^{13}C{H} NMR (126 MHz, $CDCl_3$) of 1-(3-phenylpropyl)morpholine *N*-oxide (**2r**)

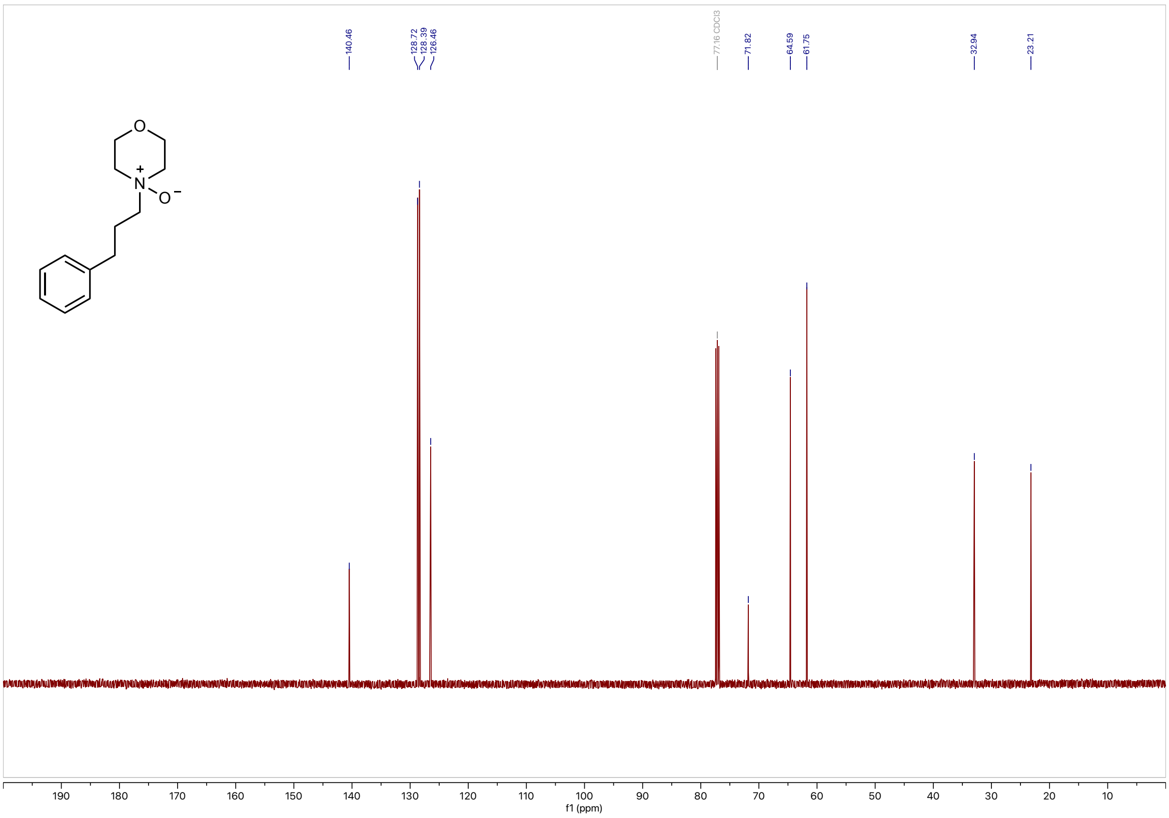

^{1}H NMR (500 MHz, $CDCl_3$) of *1*-(4-phenyl-benzyl)piperidine *N*-oxide (**7a**):

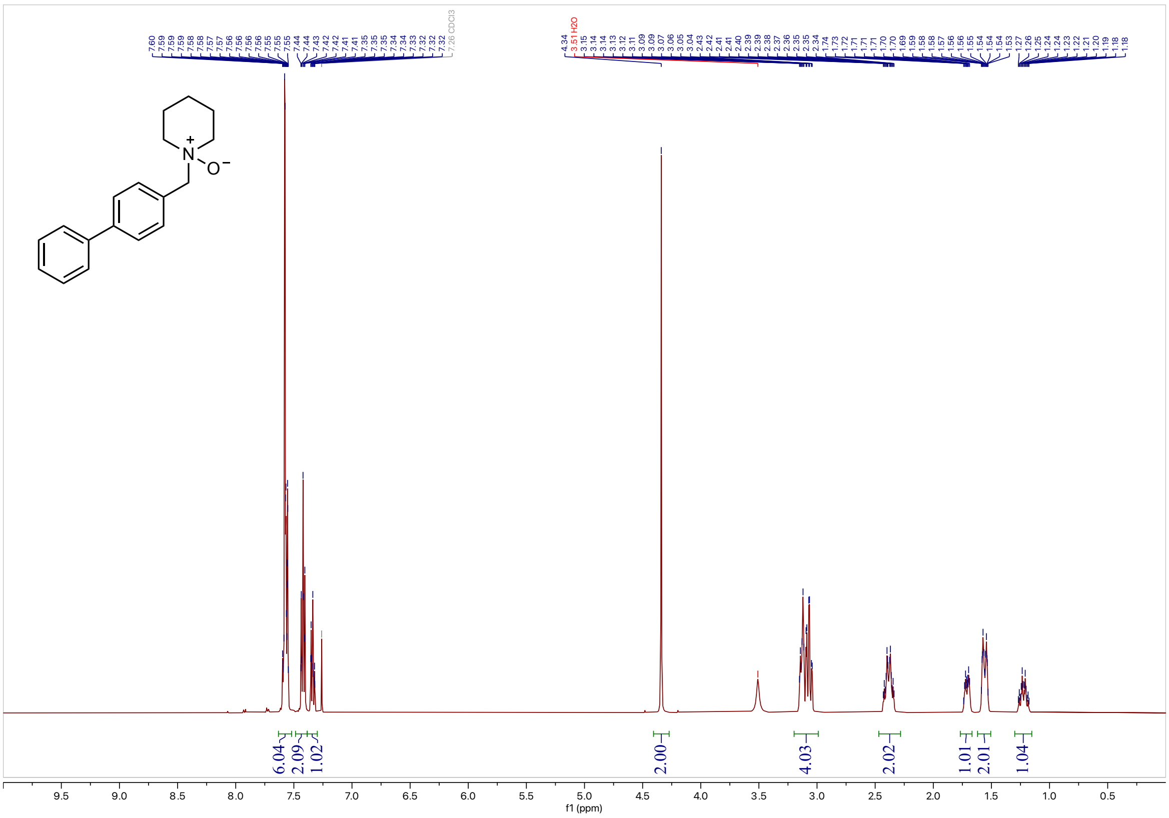

$^{13}C\{H\}$ NMR (126 MHz, $CDCl_3$) of *1*-(4-phenyl-benzyl)piperidine *N*-oxide (**7a**):

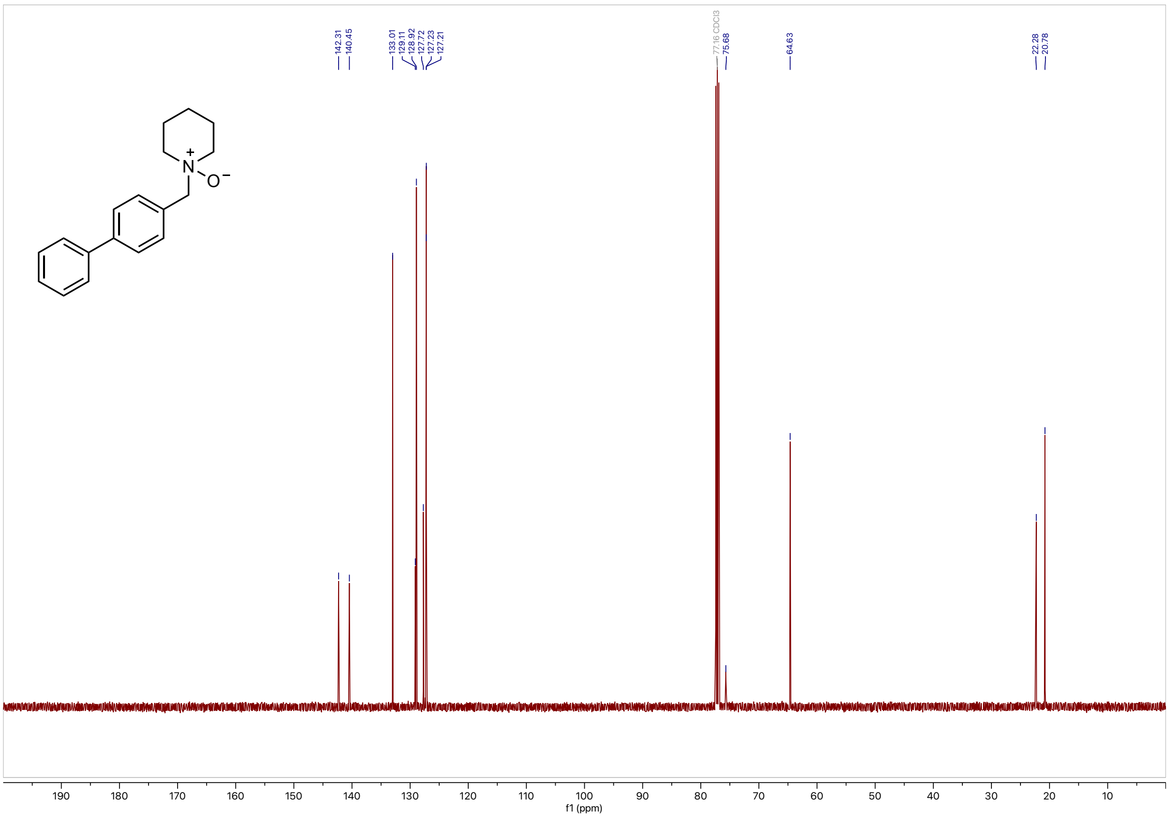

^{1}H NMR (700 MHz, $CDCl_3$) of 1-(4-phenylbenzyl)-2-methylpiperidine *N*-oxide (**7b**):

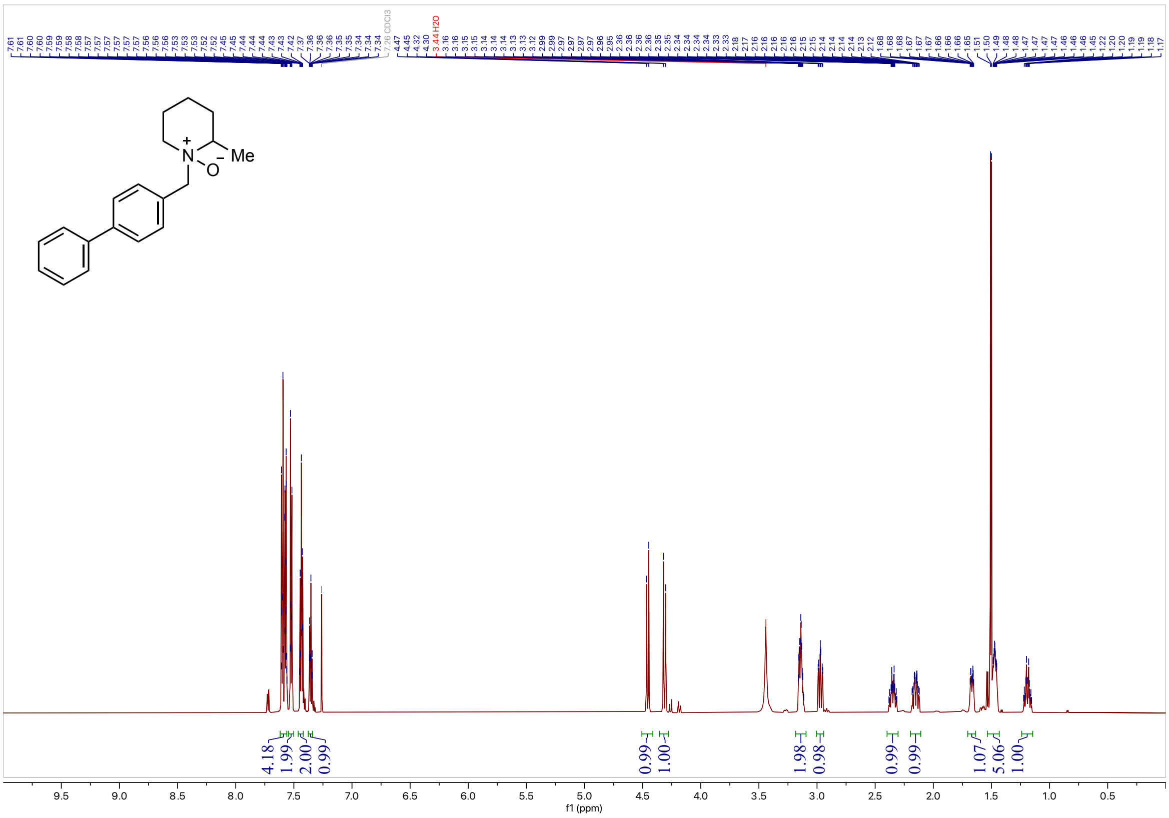

$^{13}C\{H\}$ NMR (176 MHz, $CDCl_3$) of 1-(4-phenylbenzyl)-2-methylpiperidine *N*-oxide (**7b**):

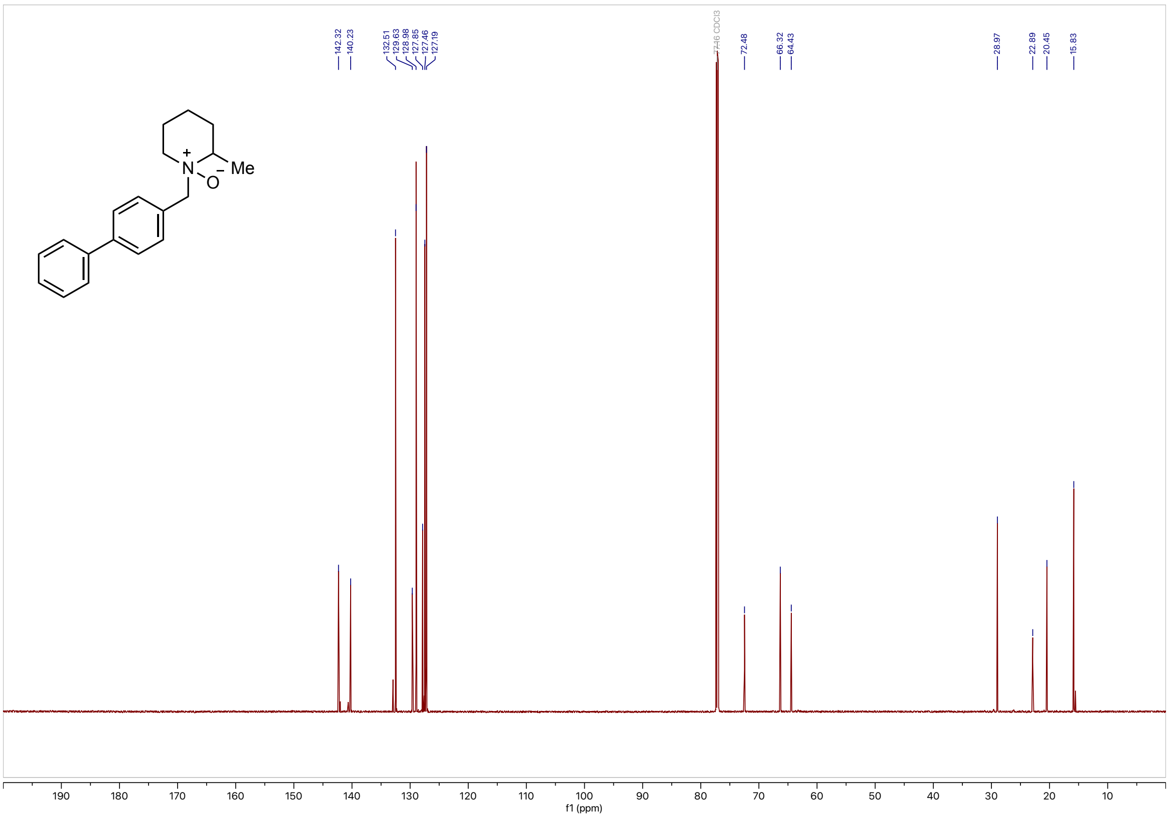

^{1}H NMR (500 MHz, MeOD) of dextromethorphan *N*-oxide (**33**):

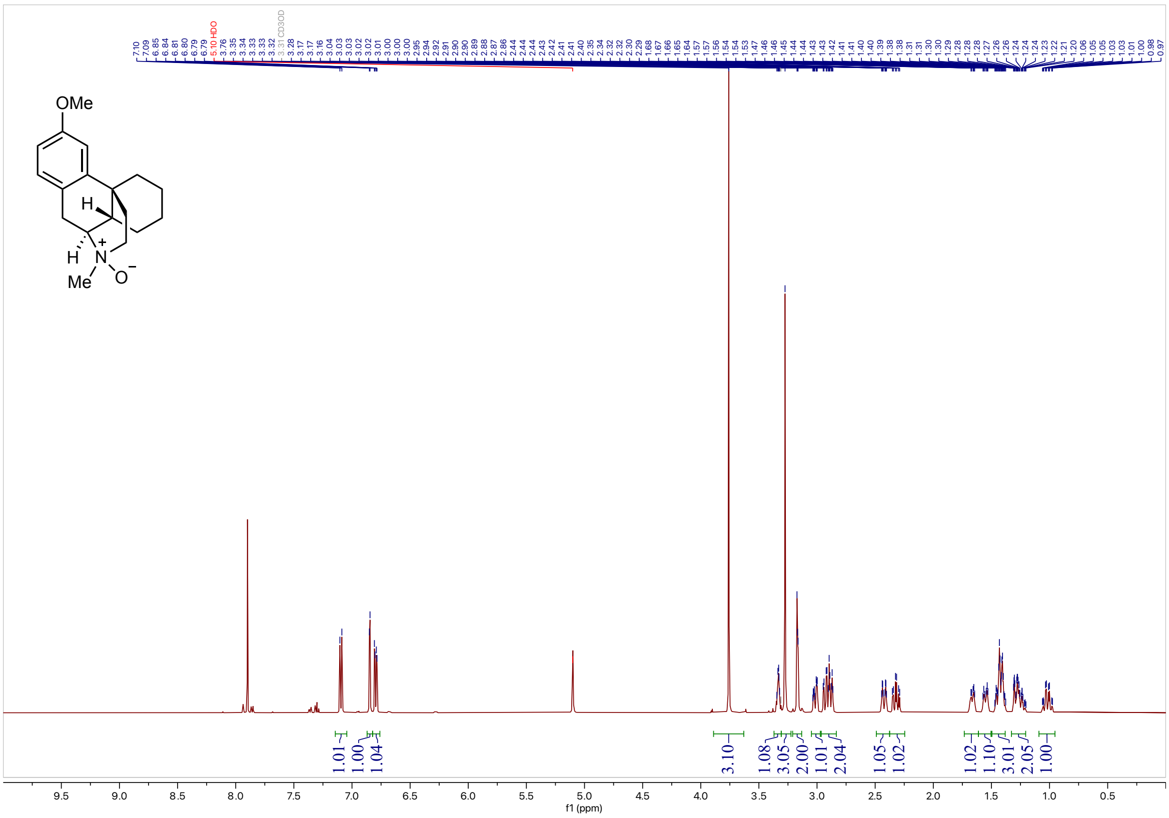

$^{13}C\{H\}$ NMR (126 MHz, MeOD) of dextromethorphan *N*-oxide (**33**):

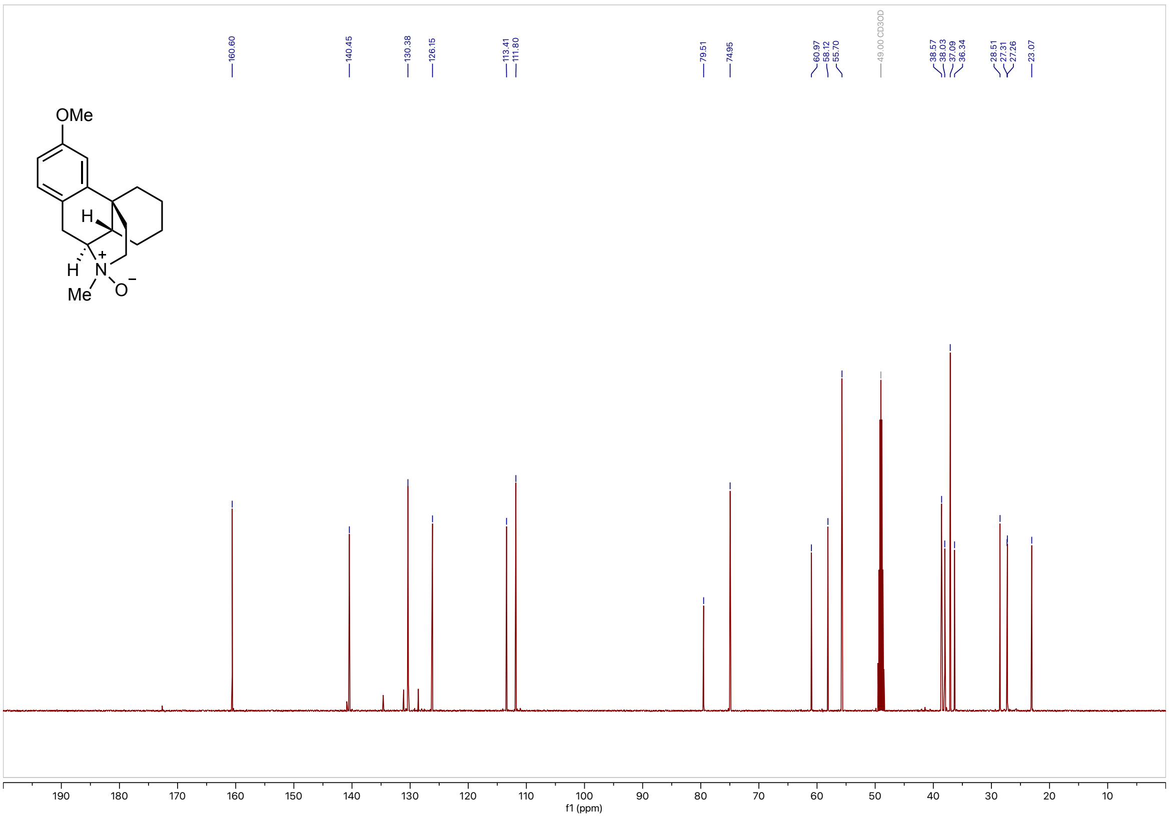

^{1}H NMR (700 MHz, $CDCl_3$) of reserpine *N*-oxide (**34**):

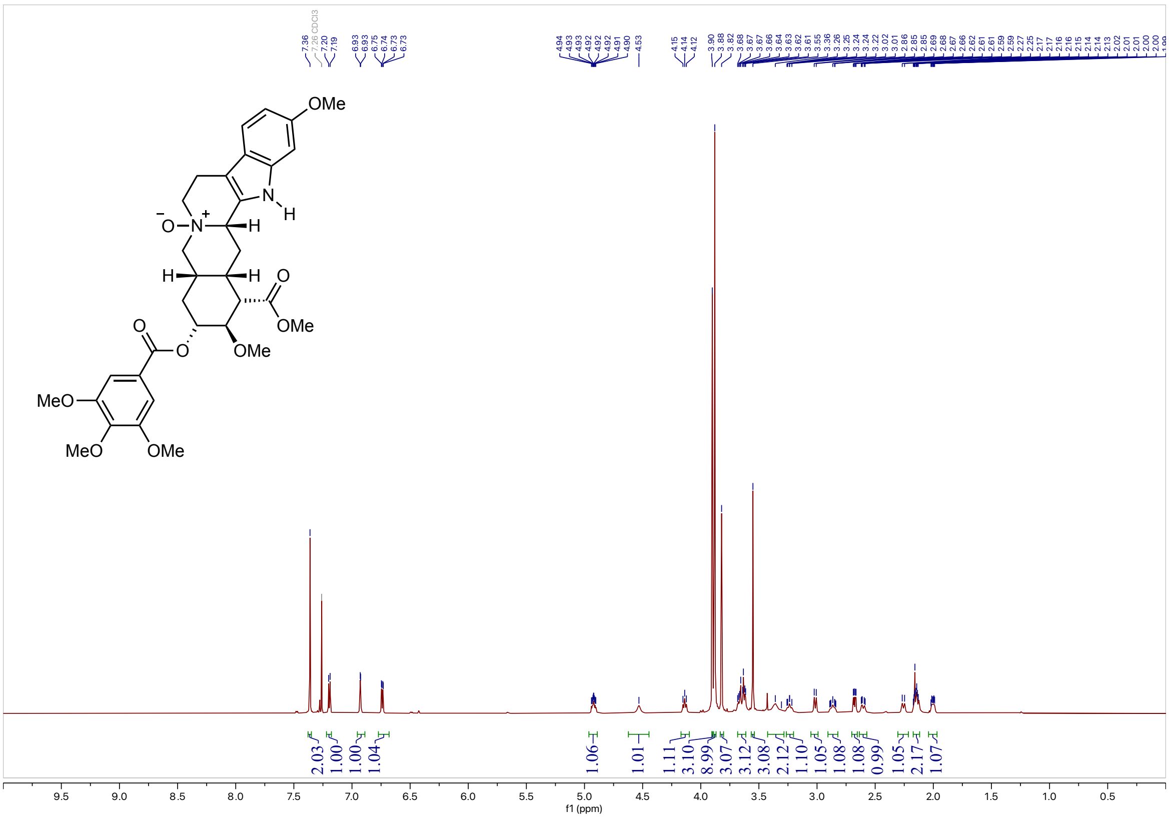

$^{13}C\{H\}$ NMR (176 MHz, $CDCl_3$) of reserpine *N*-oxide (**34**):

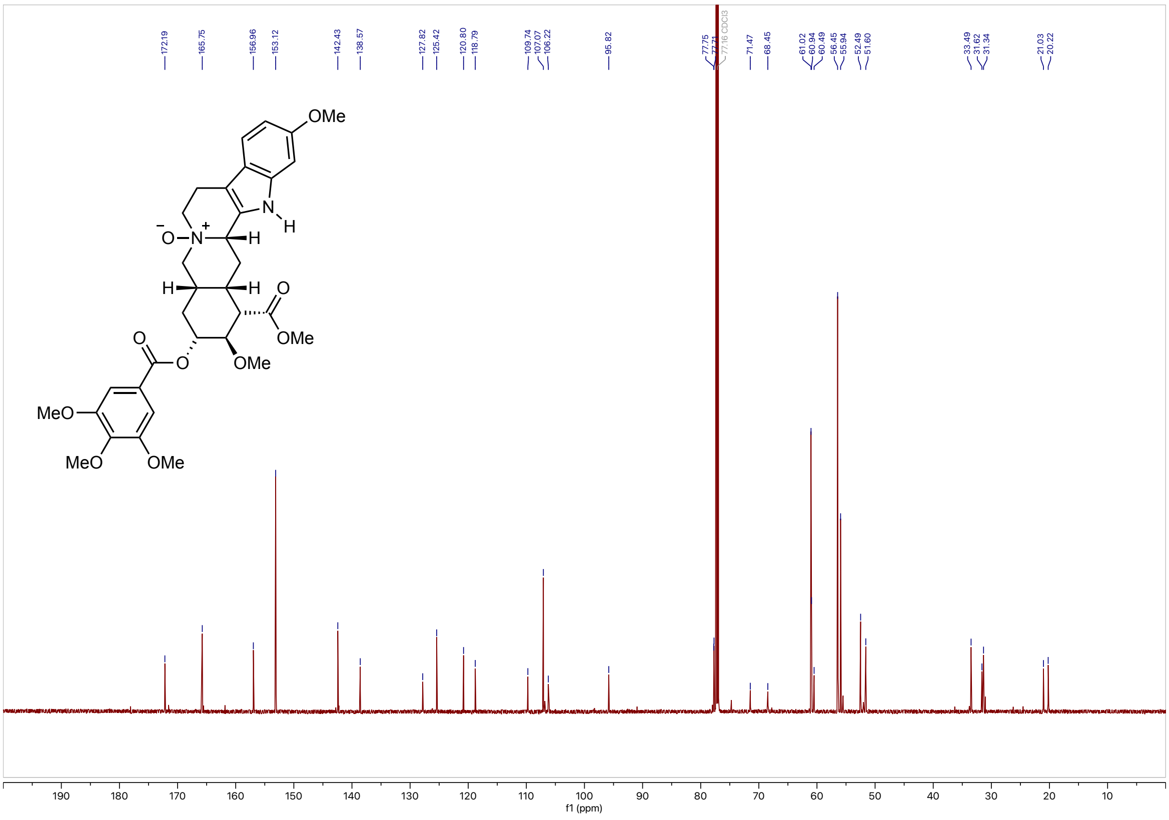

^{1}H NMR (500 MHz, $CDCl_3$) of cloperastine *N*-oxide (**35**):

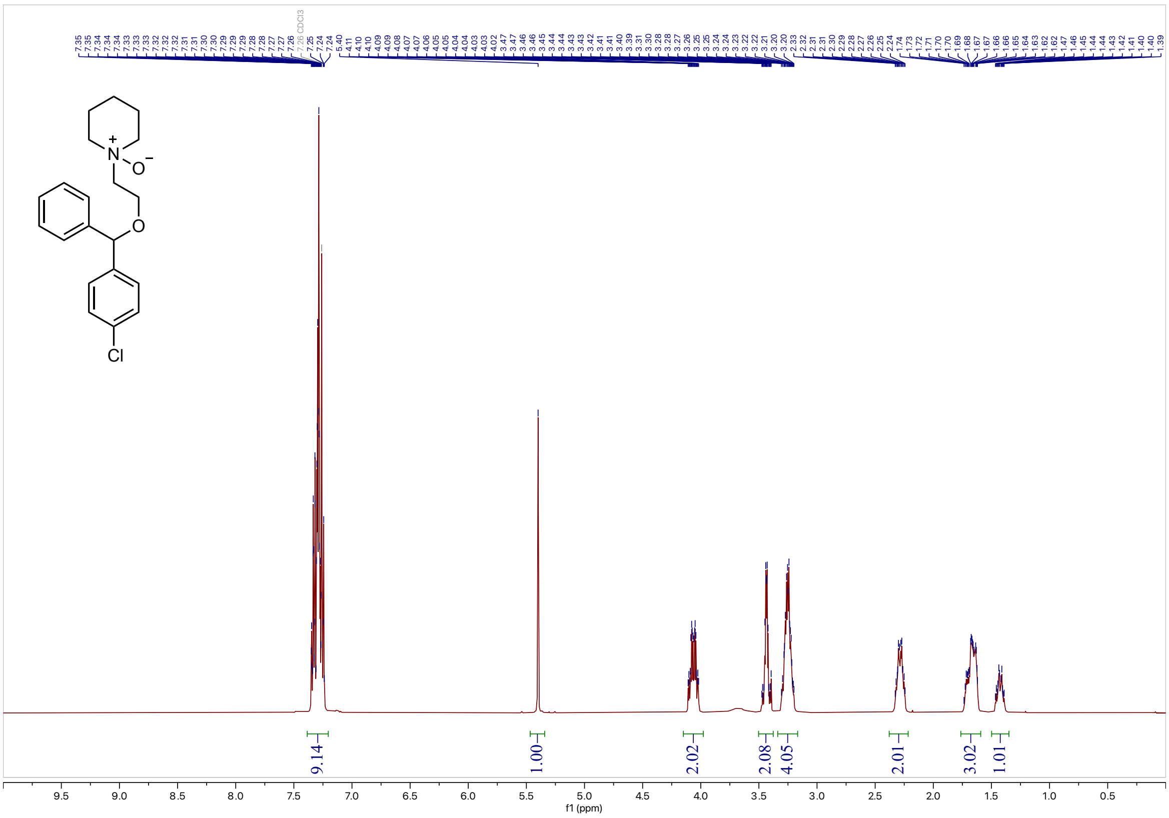

^{13}C{H} NMR (126 MHz, $CDCl_3$) of cloperastine *N*-oxide (**35**):

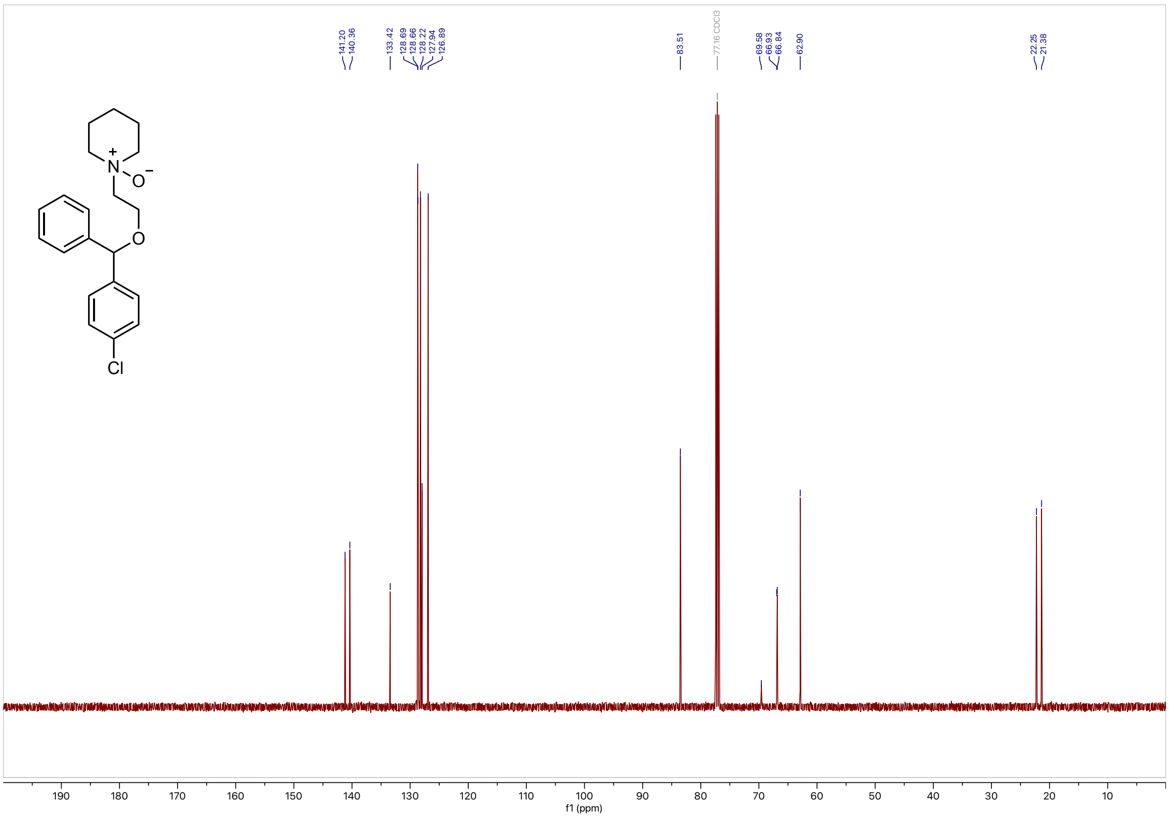

^{1}H NMR (400 MHz, MeOD) of ticlopidine *N*-oxide (**36**):

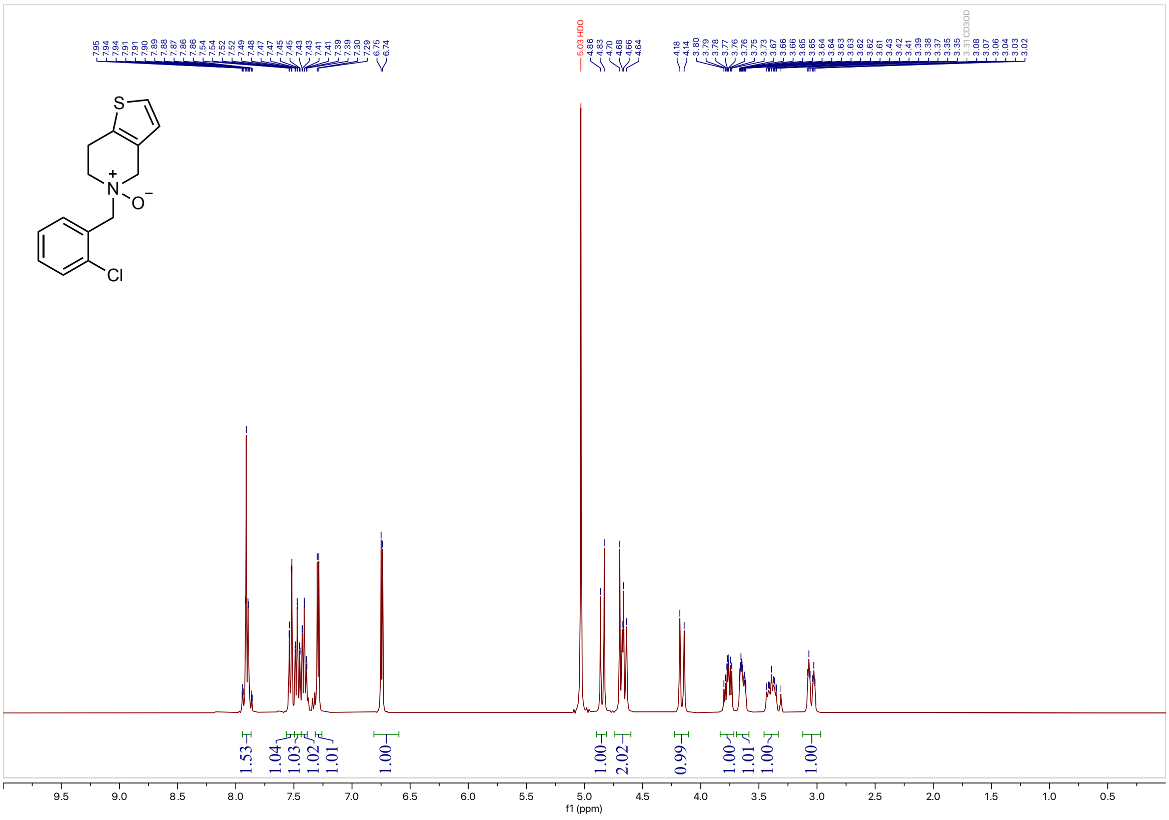

^{13}C{H} NMR (101 MHz, MeOD) of ticlopidine *N*-oxide (**36**):

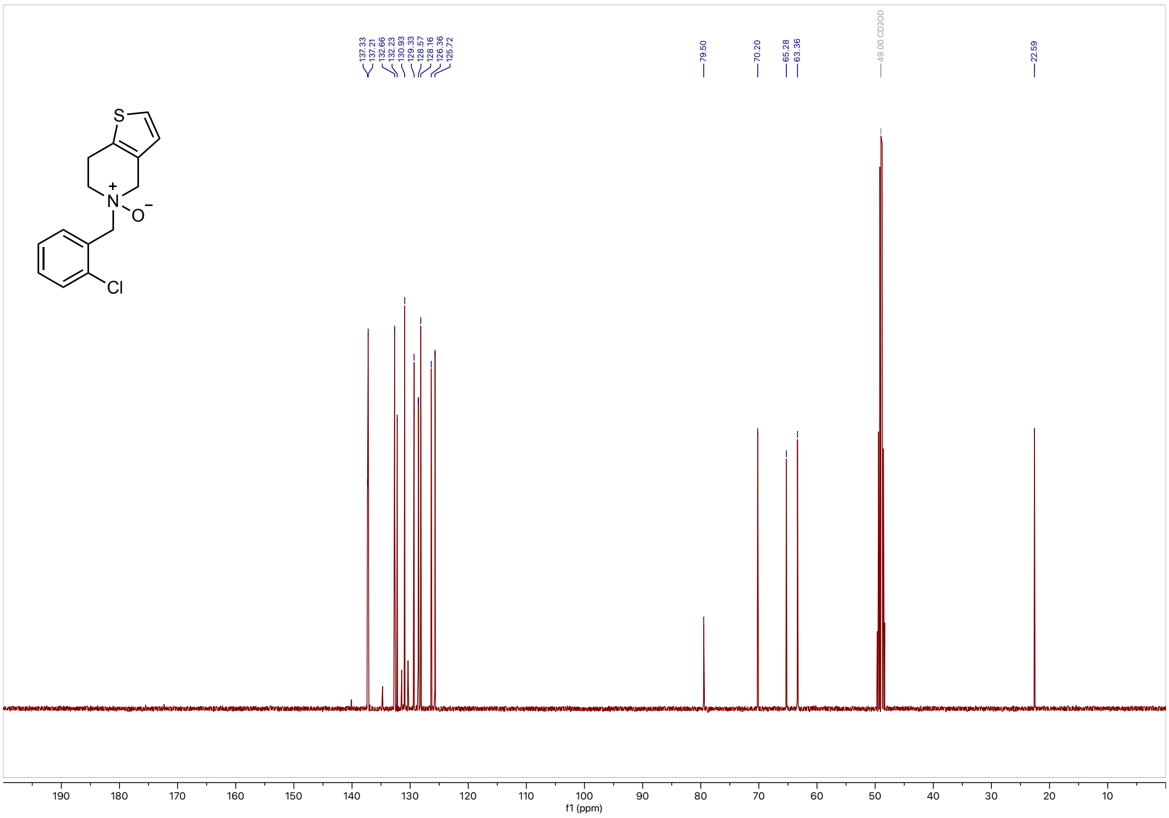

^{1}H NMR (700 MHz, $CDCl_3$) of matrine *N*-oxide (**37**)

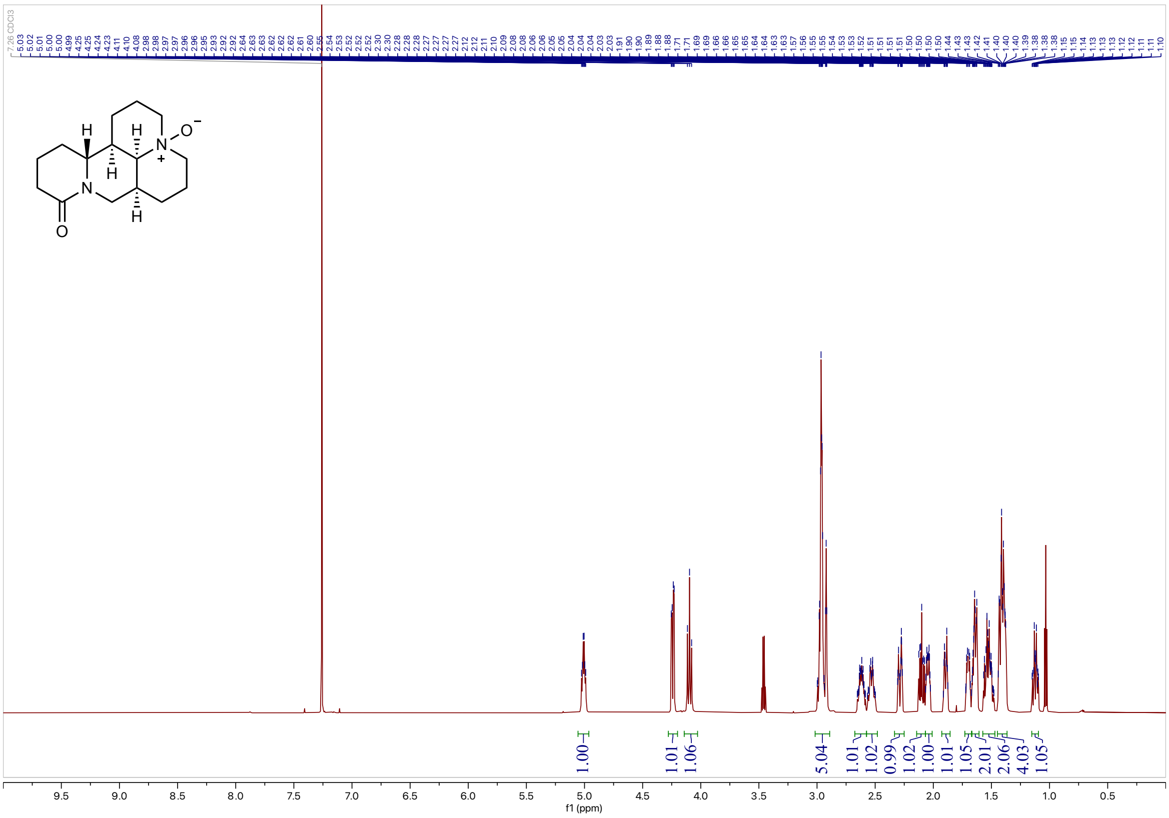

^{13}C{H} NMR (176 MHz, $CDCl_3$) of matrine *N*-oxide (**37**)

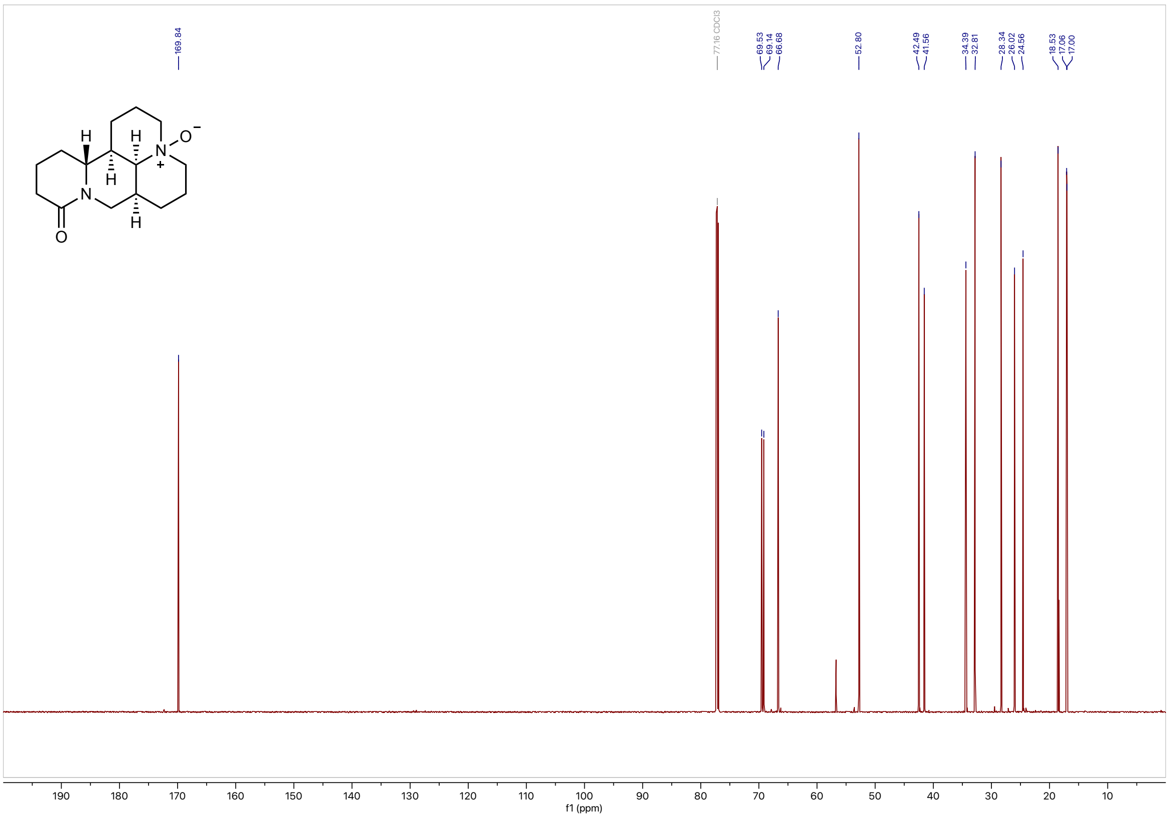

^{1}H NMR (400 MHz, $CDCl_3$) of flavoxate *N*-oxide (**38**):

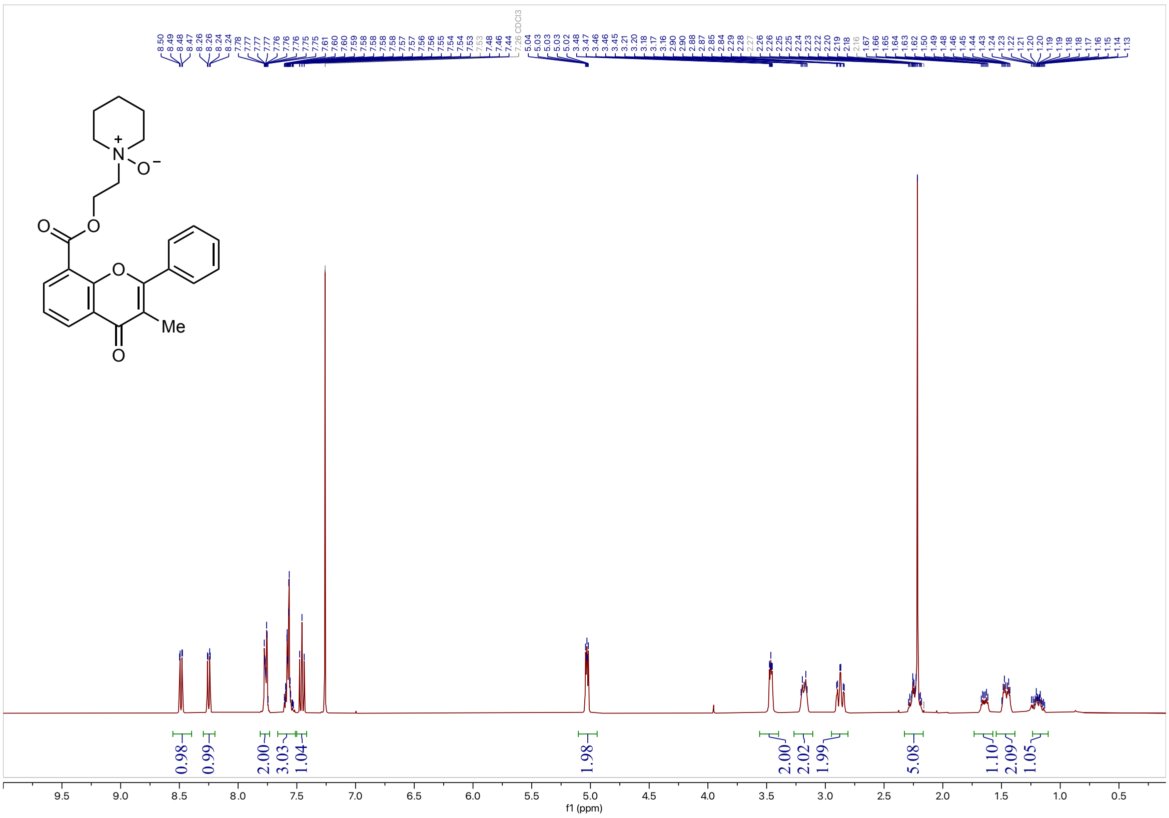

^{13}C{H} NMR (101 MHz, $CDCl_3$) of flavoxate *N*-oxide (**38**):

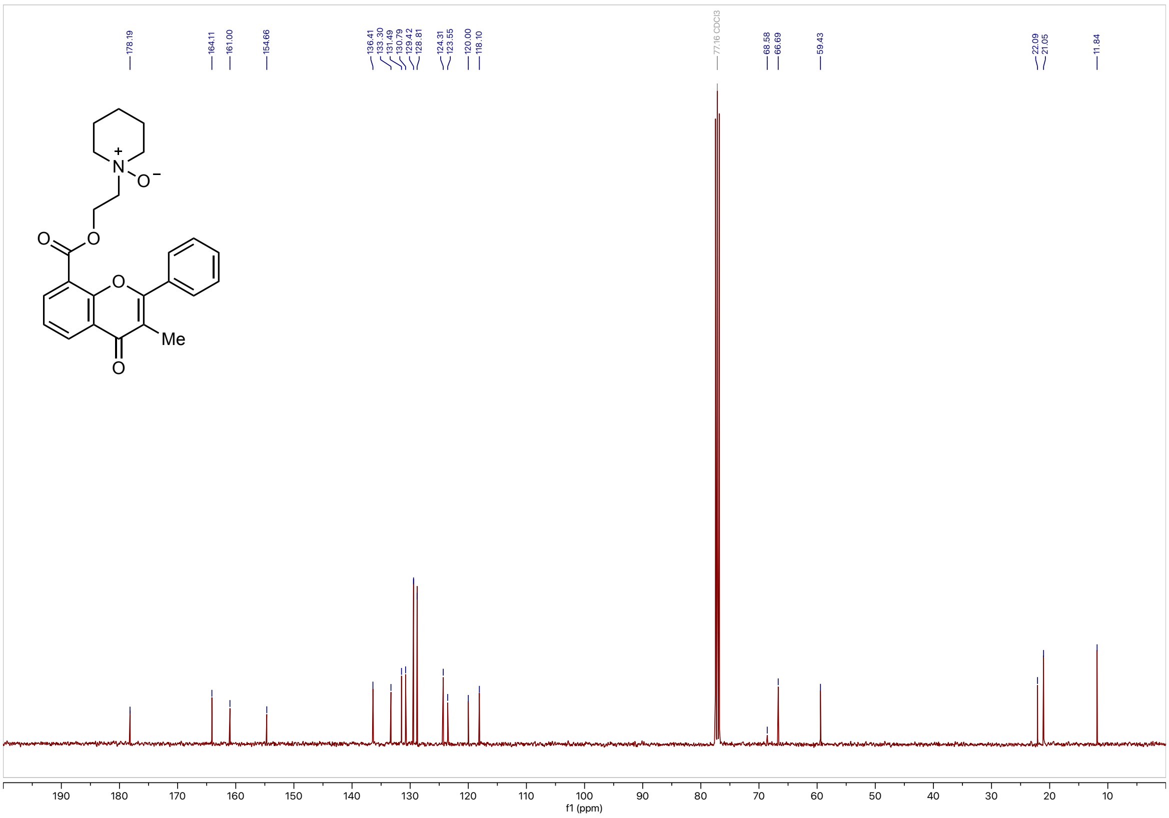

^{1}H NMR (500 MHz, $CDCl_3$) of risperidone *N*-oxide (**39**):

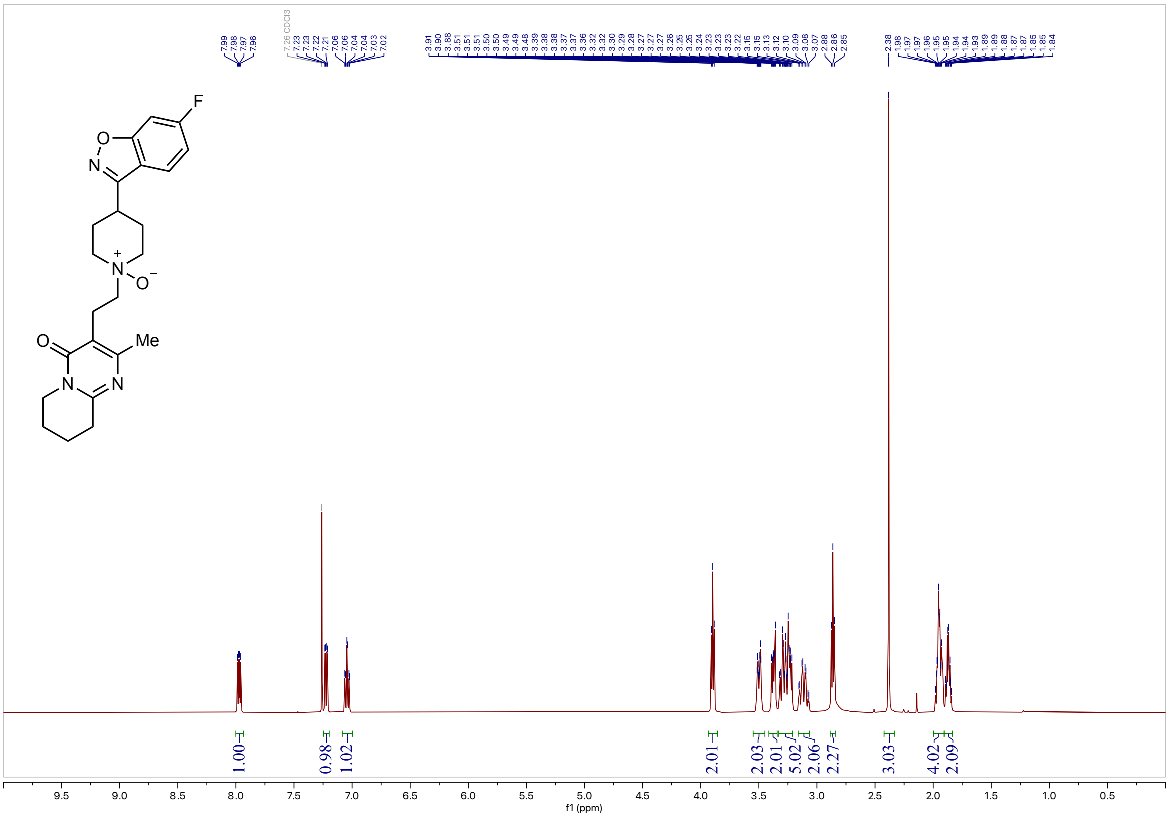

$^{13}C\{H\}$ NMR (126 MHz, $CDCl_3$) of risperidone *N*-oxide (**39**):

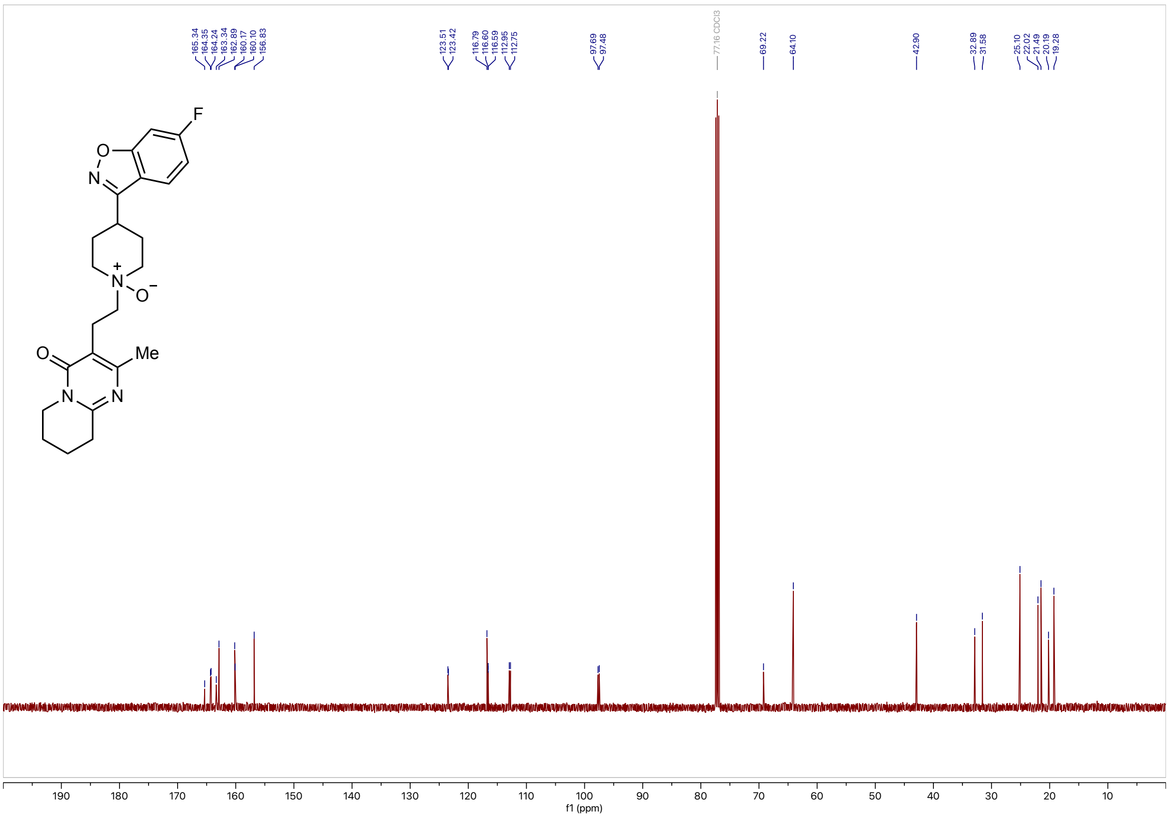

^{19}F{H} NMR (471 MHz, $CDCl_3$) of risperidone *N*-oxide (**39**):

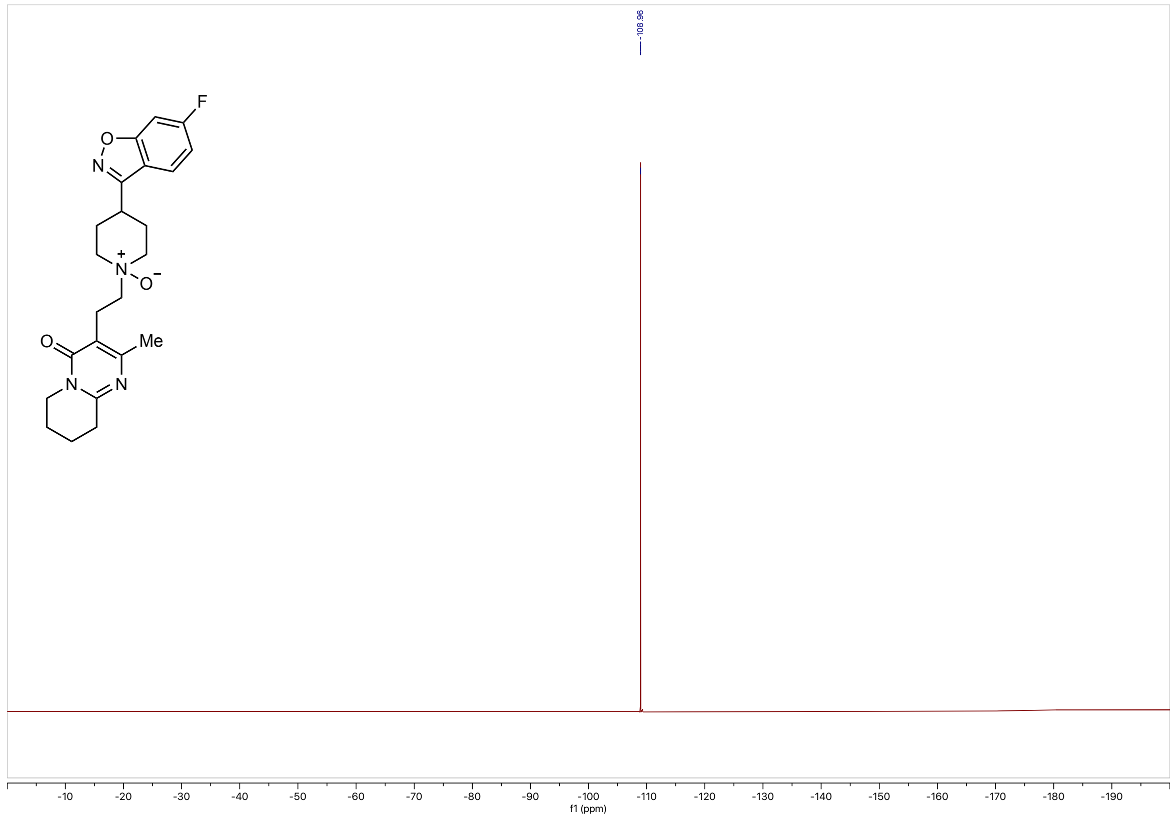

^{1}H NMR (700 MHz, MeOD) of cisapride *N*-oxide (**40**):

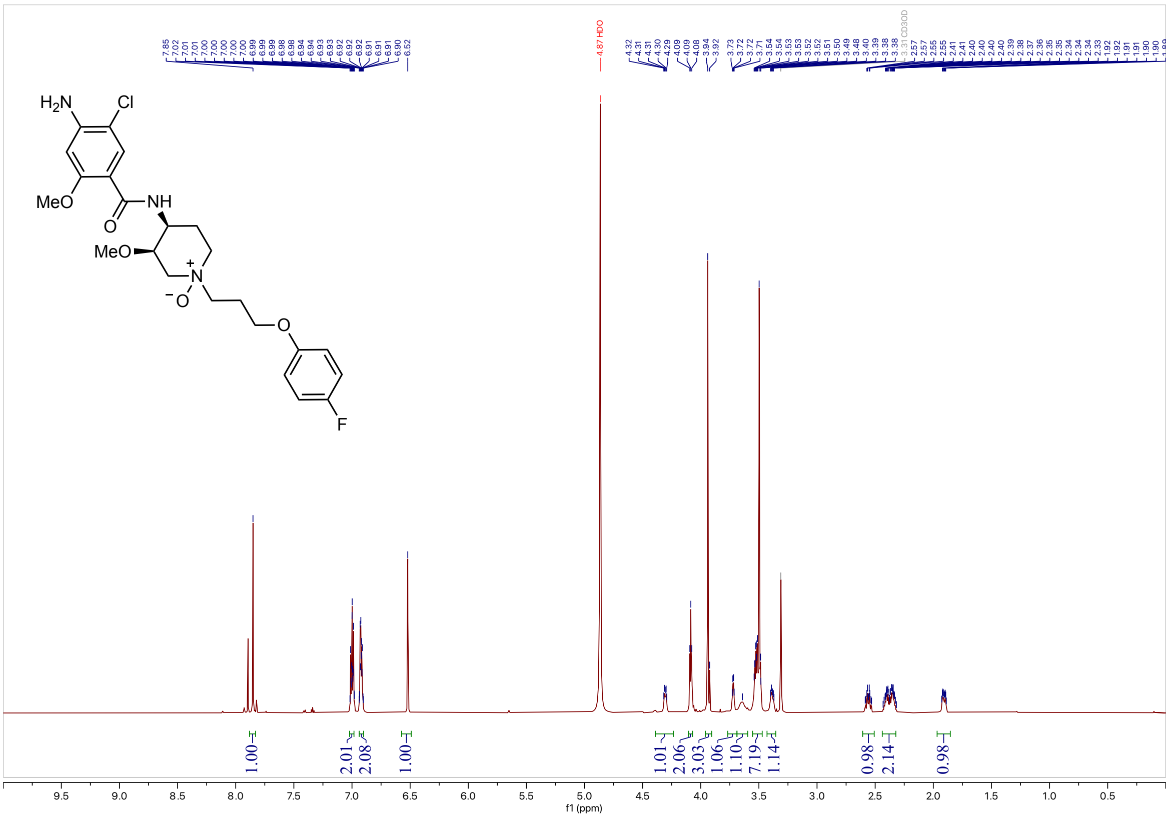

$^{13}C\{H\}$ NMR (176 MHz, MeOD) of cisapride *N*-oxide (**40**):

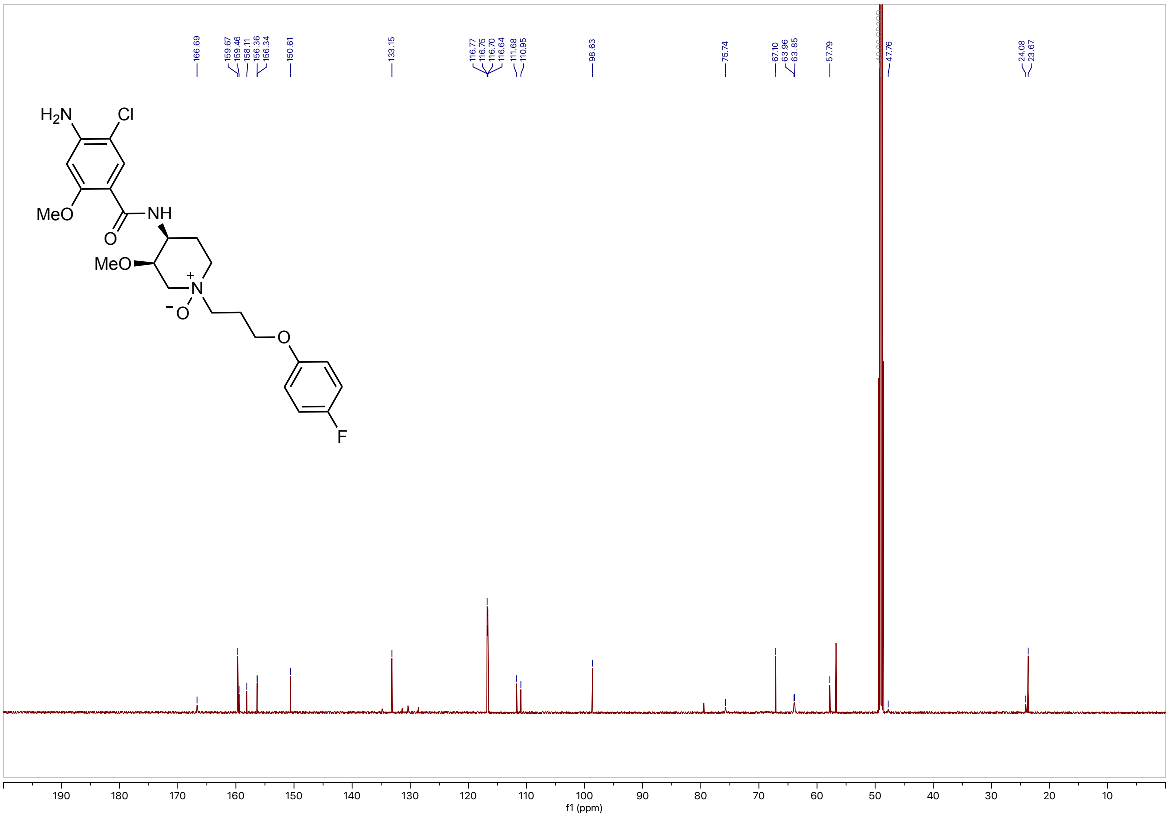

^{19}F{H} NMR (471 MHz, MeOD) of cisapride *N*-oxide (**40**):

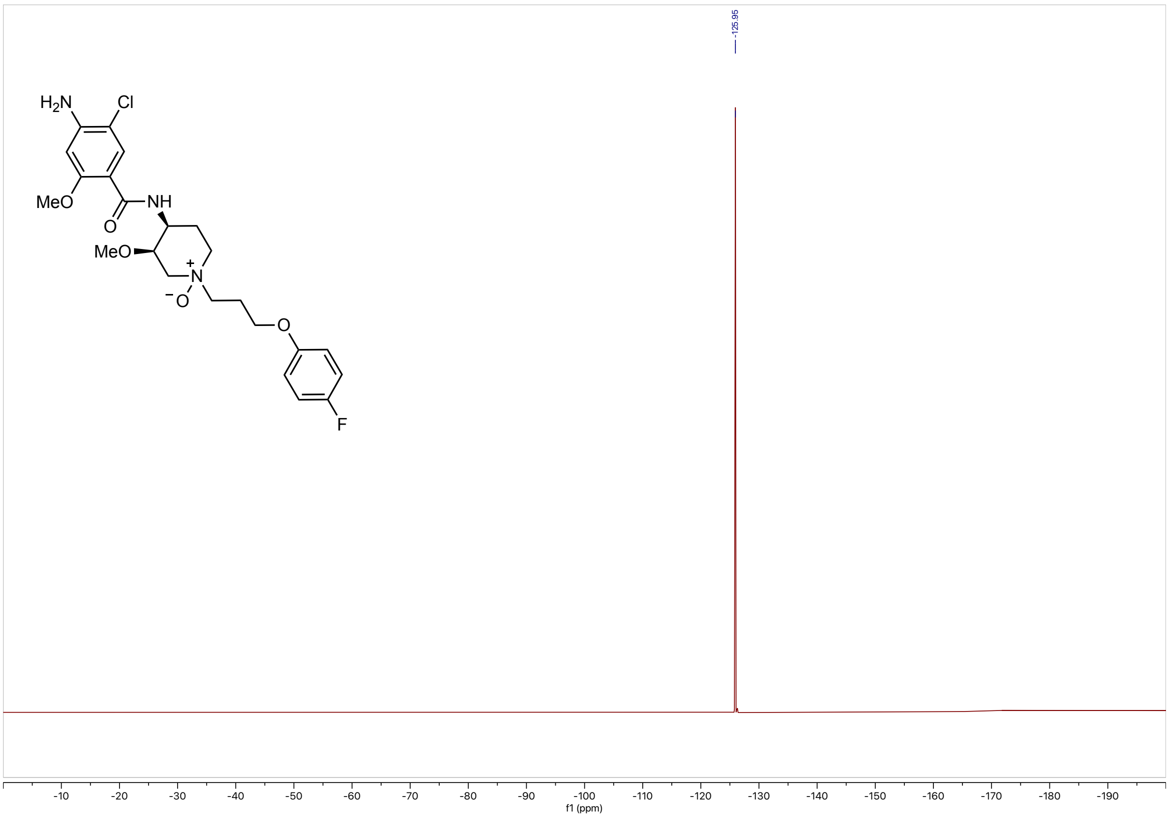

Supplement: Supplementary file 1 [file jo5c01742_si_001.pdf]
